# Perceived barriers to success for resident physicians interested in immigrant and refugee health

**DOI:** 10.1186/s12909-016-0696-z

**Published:** 2016-07-15

**Authors:** Jonathan D. Alpern, Cynthia S. Davey, John Song

**Affiliations:** Department of Infectious Disease, University of Minnesota, 800 North 3rd St. Apt 520, Minneapolis, MN 55401 USA; University of Minnesota, Biostatistical Design and Analysis Center, Clinical and Translational Science Institute, Minneapolis, MN USA; Department of Medicine, University of Minnesota, Center for Bioethics, Minneapolis, MN USA

## Abstract

**Background:**

Cross-cultural care is recognized by the ACGME as an important aspect of US residency training. Resident physicians' preparedness to deliver cross-cultural care has been well studied, while preparedness to provide care specifically to immigrant and refugee populations has not been.

**Methods:**

We administered a survey in October 2013 to 199 residents in Internal Medicine, Pediatrics, and Medicine/Pediatrics at the University of Minnesota, assessing perceived knowledge, attitudes, and experience with immigrant and refugee patients.

**Results:**

Eighty-three of 199 residents enrolled in Internal Medicine, Pediatrics and Medicine/Pediatrics programs at the University of Minnesota completed the survey (42 %). Most (*n* = 68, 82 %) enjoyed caring for immigrants and refugees. 54 (65 %) planned to care for this population after residency, though 45 (54 %) were not comfortable with their knowledge regarding immigrant and refugee health. Specific challenges were language (*n* = 81, 98 %), cultural barriers (*n* = 76, 92 %), time constraints (*n* = 60, 72 %), and limited knowledge of tropical medicine (*n* = 57, 69 %). 67 (82 %) wanted more training in refugee and immigrant health.

**Conclusions:**

The majority of residents enjoyed caring for immigrant and refugee patients and planned to continue after residency. Despite favorable attitudes, residents identified many barriers to providing good care. Some involved cultural and language barriers, while others were structural. Finally, most respondents felt they needed more education, did not feel comfortable with their knowledge, and wanted more training during residency. These data suggest that residency programs consider increasing training in these specific areas of concern.

**Electronic supplementary material:**

The online version of this article (doi:10.1186/s12909-016-0696-z) contains supplementary material, which is available to authorized users.

## Background

Cross-cultural care is a topic recognized by the Accreditation Council for Graduate Medical Education (ACGME) as an important aspect of U.S residency training [1]. Resident physicians’ preparedness to deliver cross-cultural medical care has been well studied [2–4], with research demonstrating that residents who received cross-cultural training had increased ability to deliver this care [5]. Competency in providing care specifically to immigrant and refugee populations has not been as well characterized. While incorporating the tenets and skills of cross-cultural care, the care of immigrant and refugee patients requires more unique considerations such as country of origin, refugee camp origin and conditions, cultural and language barriers, history of trauma, torture, travel and migration, and/or Post Traumatic Stress Disorder. For instance, a patient’s country of origin provides a more accurate view of one’s culture and disease risk, but may be overlooked in traditional cross-cultural care models. Or, for example, political refugees may suffer from health effects caused by torture or imprisonment. Learning to communicate effectively using a professional interpreter is an additional aspect that makes caring for this population unique.

With an estimated U.S foreign-born population of 41.3 million [6], and 69,926 refugee arrivals to the U.S in 2013 [7], the delivery of healthcare to immigrant and refugee patients in the U.S is commonplace and growing. While the ultimate number of Syrian refugees that will be admitted to the US is still in flux, crises such as these highlight the continued growth of this population. In Minnesota, the foreign-born population makes up 7 % of the general population [8], and Minnesota is a leader among U.S states in refugee arrivals from Burma (7^th^), Ethiopia (tied for 1st), and Somalia (1st) [9]. Interest in the topic of “global health” is increasing among medical students and many residency programs now offer some form of international elective or global health training to residents [10, 11]. One study found that a Refugee Health elective for pre-clinical medical students resulted in a greater awareness of health issues affecting refugees, comfort with interacting with foreign-born populations, and identifying cultural differences in understanding health conditions [12]. Another study found that medical students who participated in a training program improved their self-assessed cultural awareness [13].

However, few studies have evaluated graduate trainees’ knowledge, attitudes, and experiences caring for immigrant and refugee populations, with most evaluating specific educational interventions among small residency groups. One study of 32 resident psychiatrists found that using a virtual patient improved the confidence in providing care for traumatized refugee patients [14]. Favorable attitudes and self-assessed knowledge regarding immigrant and refugee health was seen following the implementation of a global health curriculum [15, 16]. Another study found that U.S residents had poor recognition of likely parasitic infections and the need for parasite screening [17]. Karp et al evaluated 27 pediatric residents’ attitudes, behavior, and knowledge about the rights of immigrant families [18], while another study found that participation in an American Society of Tropical Medicine & Hygiene (ASTMH)-global health curriculum improved medical knowledge of immigrants [19]. In this study, we evaluated Minnesota trainees’ self-assessed knowledge, attitudes, and experience providing care for immigrant and refugee patients.

## Methods

We performed a cross-sectional survey of resident physicians in Internal Medicine, Pediatrics, and Medicine/Pediatrics at the University of Minnesota. We created a 37-question survey with the help of faculty with expertise in immigrant and refugee health. (See Additional files 1 and 2). Despite other surveys having been done on the topic of residents’ attitude and preparedness in cross-cultural competency, our survey addressed immigrant and refugee health specifically, and therefore a new survey tool was created [2, 20]. For example, we included a question that addressed residents’ opinion of immigrant and refugee adherence to treatment plans which is more specific to these populations beyond general cultural competency. The survey was then revised after cognitive testing with medical students to improve comprehension and validity. Cognitive testing is an accepted first step in the development of a valid survey instrument [21]. In addition to demographic questions, we asked respondents a series of questions about their perceived knowledge, attitudes, and experience caring for immigrant and refugees.

The University of Minnesota Institutional Review Board reviewed and approved the survey (study number: 1306E36881). The survey instrument used was REDCap (Research Electronic Data Capture) electronic data capture tools hosted at UMN [22]. REDCap is a secure, web-based application designed to support data capture for research studies, providing 1) an intuitive interface for validated data entry; 2) audit trails for tracking data manipulation and export procedures; 3) automated export procedures for seamless data downloads to common statistical packages; and 4) procedures for importing data from external sources.

In October, 2013, we administered the survey to all residents in University of Minnesota residency programs. Residents in three primary care programs (Internal Medicine, Medicine/Pediatrics, and Pediatrics) were included in the analysis. Emails sent to all eligible residents contained a link to the anonymous survey located online using the REDCap platform (See Availability of Data and Materials section). We sent one reminder email. Participation was voluntary and there was no compensation for participating in the survey.

We calculated frequency distributions for all survey questions and demographic characteristics. Chi-Square tests were performed to identify characteristics associated with responses to four survey questions: “I would like to take care of more immigrant and refugee patients”, “I enjoy taking care of immigrant and refugee patients”, “Taking care of immigrant and refugee patients is more challenging than caring for U.S born patients, and “I feel comfortable with my fund of knowledge regarding immigrant and refugee health”. Fisher’s exact tests were performed if the Chi-square test was invalid due to small cell counts. All analyses were completed using SAS 9.3 (SAS Institute, Cary NC).

## Results

### Respondent demographics

A total of eighty-three of 199 residents enrolled in the Internal Medicine, Pediatrics and Medicine/Pediatrics programs at the University of Minnesota completed the survey (*n* = 83, 42 %). Approximately half of the respondents were female (*n* = 42, 51 %), 69 (84 %) were white, 9 (11 %) were Asian, and 11 (13 %) were born outside of the US. Of the 83 survey respondents, 75 (90 %) had a US degree and 8 (10 %) had a non-US degree. With a total of 178 (89 %) US graduates and 21 (11 %) international graduates comprising the Internal Medicine, Pediatrics, and Medicine/Pediatrics programs, the response rate was 42 % (75/178) for US graduates and 38 % (8/21) for international graduates.

Most respondents had an estimated educational debt > $100,000 (*n* = 54, 65 %) and planned to subspecialize (*n* = 48, 58 %). Nearly half were enrolled in the University of Minnesota Global Health Pathway elective (*n* = 36, 44 %), and spoke more than one language (*n* = 41, 49 %). The Global Health Pathway is a track available to residents that offers didactics and experience in the areas of immigrant and refugee health, international clinical rotations, and access to a Centers for Disease Control-sponsored Global Health course. Almost all respondents identified themselves as being politically liberal or moderate (*n* = 76, 92 %) (See Table 1).

**Table 1:** Demographics and Program Characteristics of Survey Respondents

Demographics and Program Characteristics		Demographics of Minnesota Physician Workforce, 2013-2014 [30]	
Age			
<30	51 (62 %)		
30 or older	32 (39 %)		
Gender		Gender	
Female	42 (51 %)	Female	7,041 (32.5 %)
Male	41 (49 %)	Male	14,623 (67.5 %)
Race		Race	
White	69 (84 %)	White	72.3 %
Asian	9 (11 %)	Asian	7.6 %
Other	4 (5 %)	Other	5.7 %
		Unknown	14.4 %
Born in the US		Foreign- trained	2,141 (14 %)
Yes	72 (87 %)		
No	11 (13 %)		
Program Year			
PGY1-3	76 (92 %)		
PGY4-5	7 (8 %)		
Political Ideology			
Conservative	7 (8 %)		
Moderate	19 (23 %)		
Liberal	57 (69 %)		
Estimated Educational Debt			
< $100,000	29 (35 %)		
$100,000 or more	54 (65 %)		
Plan to subspecialize			
Yes	48 (58 %)		
No	35 (42 %)		
In the Global Health Pathway			
Yes	36 (44 %)		
No	46 (56 %)		
Earned degree in the US			
Yes	75 (90 %)		
No	8 (10 %)		
Residency Program			
Internal Medicine	38 (46 %)		
Pediatrics	21 (25 %)		
Medicine/Pediatrics	24 (29 %)		
More than 1 language spoken			
Yes	41 (49 %)		
No	42 (51 %)		

Data as Number (% of respondents)

### Attitudes towards immigrant and refugee health

There were 68 (82 %) respondents that reported usually or always enjoying caring for immigrants and refugees. The most cited reasons included learning about tropical and other diseases (*n* = 59, 71 %) and about other cultures (*n* = 67, 81 %). Other common reasons included hearing their patients’ stories (*n* = 42, 51 %), patients being appreciative of the care that they receive (*n* = 47, 57 %), and the belief that these are vulnerable populations in need of care (*n* = 42, 51 %). Agreement with the statement: “I enjoy taking care of immigrant/refugee patients” was significantly associated with having received training in immigrant and refugee health (*p* < .001), being multilingual (*p* = .002), and being in the Global Health Pathway (*p* = .01). It was only marginally significantly associated with comfort with knowledge of immigrant and refugee health (*p* = .05).

Almost all respondents (*n* = 82, 99 %) felt that caring for immigrant or refugees was sometimes (*n* = 40, 48 %), usually (*n* = 36, 43 %) or always (*n* = 6, 7 %) more challenging than caring for US born patients. Many challenges were identified, with the most commonly cited including language barriers (*n* = 81, 98 %), cultural barriers (*n* = 76, 92 %), time constraints (*n* = 60, 72 %), limited knowledge of tropical medicine (*n* = 57, 69 %), and patients not understanding treatment plans (*n* = 54, 65 %). Finding an interpreter was a common challenge (*n* = 51, 62 %) while working with an interpreter was rarely (*n* = 8, 10 %) perceived as a challenge.

### Personal experience caring for immigrant and refugee patients

During inpatient rotations, 57 (69 %) respondents estimated that less than 10 % of hospitalized patients whom they care for are immigrants or refugees. During outpatient rotations, 52 (63 %) respondents estimated that less than 25 % of clinic patients they have treated are immigrants or refugees. However, almost half would like to care for more (*n* = 39, 47 %). Agreement with the statement: “I would like to care for more immigrant and refugee patients” was significantly associated with having received training in immigrant and refugee health (*p* = .005), comfort with knowledge of immigrant and refugee health (*p* = .003), and reporting “usually or always” enjoy caring for immigrant/refugee patients (*p* = .004). Most respondents planned to care for this population after residency (*n* = 54, 65 %), with approximately half planning to do short term international work (*n* = 40, 48 %), and one-quarter planning to do long-term international work (*n* = 21, 25 %).

### Medical education and immigrant and refugee health

However, 45 (54 %) were not comfortable with their knowledge of immigrant and refugee health, despite 53 (64 %) having received training in refugee and immigrant health. The majority of respondents who had received training reported training from more than one program (*n* = 33, 62 %) including during residency (*n* = 45, 85 %), during medical school (*n* = 27, 51 %), in a special program (*n* = 14, 26 %), as an undergraduate (*n* = 9, 17 %), or in a degree program (*n* = 8, 15 %). Those who had received training were significantly more likely than those who had not received training to 1) agree that they were comfortable with their knowledge regarding immigrant and refugee health (53 % vs 3 %), 2) usually to always enjoy caring for immigrant and refugee patients (93 % vs 63 %), 3) agree that they would like to care for more immigrant and refugee patients (59 % vs 27 %), and 4) estimate that more than 25 % of hospitalized patients they cared for were immigrant or refugee patients (51 % vs 13 %). There was no significant difference in estimated percent of clinic patients who were immigrant or refugee patients between those who had or had not received training (*p* = 0.094). (See Table 2).

**Table 2:** Associations with Training in Immigrant and Refugee health

Survey question *N* (%) with indicated responses for those with and without training in immigrant and refugee health are reported	“I have received training in Immigrant and Refugee Heath”		*p*-value*
	Yes	No^a^	
	*N* = 53	*N* = 30	
I am comfortable with my knowledge regarding Immigrant and refugee health			< 0.0001
Agree	28 (52.8 %)	1 (3.3 %)	
Disagree or No opinion	25 (47.2 %)	29 (96.7 %)	
I enjoy caring for Immigrant and refugee patients			0.0009
Usually – Always	49 (92.5 %)	19 (63.3 %)	
Never – Sometimes	4 (7.5 %)	11 (36.7 %)	
I would like to care for more immigrant and refugee patients			0.005
Agree	31 (58.5 %)	8 (26.7 %)	
Disagree or No opinion	22 (41.5 %)	22 (73.3 %)	
Estimated percent of clinic patients cared for who are Immigrant and refugee patients			0.094
0-10 %	33 (62.3 %)	24 (80 %)	
More than 10 %	20 (37.7 %)	6 (20 %)	
Estimated percent of hospitalized patients cared for who are Immigrant and refugee patients			0.0007
0-25 %	26 (49.1 %)	26 (86.7 %)	
More than 25 %	27 (50.9 %)	4 (13.3 %)	

*p-values are for Chi-square test of association between training received (Yes or No) and indicated survey responses

^a^Includes 5 respondents who answered ‘No opinion’ to the question about training

s

Factors associated with respondents not feeling comfortable with their knowledge of immigrant and refugee health were not planning to take care of immigrant and refugees after residency (*p* = .05), not having received training in immigrant and refugee health (*p* < .001), and younger respondents (*p* = 0.004). Finally, most residents would like more training in immigrant and refugee health (*n* = 67, 82 %), and for this training to be a part of residency (*n* = 62, 75 %).

## Discussion

This survey of resident physicians is the first study to our knowledge that comprehensively describes medical trainees’ attitudes, knowledge, and experience specifically with immigrant and refugee health. The majority of our residents enjoy caring for immigrant and refugee patients. However, despite favorable attitudes towards treating immigrants and refugees, residents identified many barriers to providing good care. Some of these involved cultural and language barriers, while others were structural, such as time constraints. Interestingly, finding an interpreter was identified as a common barrier while working with an interpreter was not.

While the majority of respondents reported having already received training in immigrant and refugee health, most did not feel comfortable with their knowledge of immigrant and refugee health and would like more training. Importantly, most respondents wanted this training to be part of residency training. The fact that respondents who reported receiving training in immigrant and refugee health were significantly more likely to report feeling comfortable with their knowledge in immigrant/refugee health supports the notion that additional training would likely result in improved knowledge in this area. More studies are needed to confirm this theory. Finally, despite the above challenges and indications of anti-immigrant and refugee sentiments in the general population most respondents would like to care for more immigrants and refugees [23]. And, even while the majority of residents report large educational debts, most plan to work with these populations after residency. It is noteworthy that the majority of respondents identified politically as being liberal. Further research is needed to determine what role political ideology may have on attitudes towards immigrant and refugee patients.

Our findings suggest a general feeling that resident physicians’ current training is inadequate, and highlights the need for targeted didactics that cover health issues that disproportionately and specifically affect immigrant and refugee populations. These findings are supported by previous studies, which showed that U.S residents’ knowledge of diseases affecting immigrants and refugees is poor, and that a targeted educational program improves knowledge in this area [15–17, 19]. Research in cross-cultural care training has similarly shown that despite an interest in cross-cultural care, training during residency remains inadequate [20]. Improving residents’ knowledge of immigrant and refugee health likely requires dedicated funds as well as the recruitment of full-time faculty with expertise in this area [15, 16, 24].

Our findings also suggest that residency programs could focus on quality improvement efforts that address access to interpreter services and encounter time. Previous studies have found that most residents do not regularly use professional interpreters for non-English speaking patients [25, 26] despite data suggesting the benefit of professional interpreters on patient communication, satisfaction, and outcomes [27]. Our finding that access to interpreter services was challenging has been described elsewhere [28], and emphasizes the need for programs to consider identifying barriers to interpreter services. Finally, programs might consider allotting more time for residents to see non-English speaking patients in clinic. A previous study similarly found that residents do not feel that they have enough time to treat diverse patients effectively [29]. Decreasing time constraints may improve both resident and patient satisfaction.

### Limitations

This study has several limitations. The data represent self-assessment, which is subject to social desirability bias; however, these were done anonymously which might mitigate some of these effects. Response bias may have self-selected residents who were more interested in the topic of immigrant and refugee health; unfortunately, we were not able to ascertain the demographics of those who did not participate. However, the similar response rates for U.S and international graduates indicate that the sample was not biased towards international residents. This survey has not been validated; however, it was constructed with the input of several physician-educators who specialize in immigrant and refugee health and was cognitively tested prior to administration. In addition, there is a strong emphasis on global medicine in these three programs, with access to a global health curriculum that includes opportunities for international electives, and participation in an ASTMH-accredited certificate course. The survey was only administered at one institution, and most respondents were white, identified politically as being liberal, and were born in the U.S, which might have skewed the data. Finally, our survey did not differentiate between refugees and migrants, who have very different experiences, especially with regards to trauma and geographic exposure. Future studies are needed and would benefit from a larger sample size, multiple specialties, and the inclusion of multiple institutions.

## Conclusion

These survey results demonstrate that most residents enjoy working with immigrants and refugees but identify many barriers to providing good care. Despite training in programs with a strong global health focus, most residents do not feel comfortable with their knowledge of immigrant and refugee health and would like more training during residency. These data suggest that residency programs should consider dedicated training to residents focused in the area of immigrant and refugee health, beyond the current training in cultural competency. In addition, identifying and addressing system issues that could affect access to interpreter services may improve the attitudes of trainees in this area.

## Abbreviations

ACGME, Accreditation Council for Graduate Medical Education; ASTMH, American Society of Tropical Medicine and Hygiene; REDCap, Research Electronic Data Capture

## Additional files

Additional file 1: Title of data: Raw Data Set. Description: Full raw data set via excel spreadsheet. (XLSX 93 kb) Additional file 2: Title of data: Data set in questionnaire format. Description: Full data set in survey questionnaire format via PDF. (PDF 2448 kb)
