# Supplementary material for: Perceived barriers to success for resident physicians interested in immigrant and refugee health
Source: BMC Med Educ. 2016 Jul 15;16:178. doi: 10.1186/s12909-016-0696-z (PMC4946089; doi:10.1186/s12909-016-0696-z)
Supplement: Additional file 2: — Title of data: Data set in questionnaire format. Description: Full data set in survey questionnaire format via PDF. (PDF 2448 kb) [file 12909_2016_696_MOESM2_ESM.pdf]

Medical Trainees' attitudes, knowledge, and experience with immigrant and refugee health

Response was added on 11/01/2013 2:50pm.

SECTION A: Personal experience with immigrant and refugee health care.

A. Please indicate your level of agreement with the following statements regarding your personal experience with immigrant and refugee health care by checking the box that best represents your experience.

a. During my inpatient rotations, I take care of the following percentage of immigrant and refugee patients:

- ◯ None
- ◯ 0 -5%
- ⊗ 5-10%
- ◯ 10-25%
- ◯ > 25%

b. During my outpatient rotations, I take care of the following percentage of immigrant and refugee patients:

- ◯ None
- ⊗ 0-10%
- ◯ 10 -25%
- ◯ 25-50%
- ◯ 50-75%
- ◯ >75%

c. I would like to take care of more immigrant and refugee patients.

- ◯ Strongly disagree
- ◯ Disagree
- ⊗ No opinion
- ◯ Agree
- ◯ Strongly agree

d. I plan to take care of immigrants and refugees when I finish residency.

- ◯ Strongly disagree
- ◯ Disagree
- ⊗ No opinion
- ◯ Agree
- ◯ Strongly agree

e. I plan to do short term (< 6 months) international work when I finish residency.

- ◯ Strongly disagree
- ⊗ Disagree
- ◯ No opinion
- ◯ Agree
- ◯ Strongly agree

f. I plan to do long term (>6 months) international work when I finish residency.

- ⊗ Strongly disagree
- ◯ Disagree
- ◯ No opinion
- ◯ Agree
- ◯ Strongly agree

g. I plan to work in health disparities in the following way after residency:

Unsure career path at this point

SECTION B: MEDICAL EDUCATION

A. Please indicate your level of agreement with the following statements regarding your medical education and knowledge about immigrants and refugees by checking the box that best represents your opinion.

a. I have received specialized training in immigrant and refugee health, tropical medicine, or cross-cultural health.

◯ Strongly disagree
⊗ Disagree
◯ No opinion
◯ Agree
◯ Strongly agree

c. I feel comfortable with my fund of knowledge regarding immigrant and refugee health.

◯ Strongly disagree
⊗ Disagree
◯ No opinion
◯ Agree
◯ Strongly agree

d. I would like to have further training in immigrant and refugee health.

◯ Strongly disagree
◯ Disagree
◯ No opinion
⊗ Agree
◯ Strongly agree

e. If you agree with the above, please indicate all the contexts in which you would like to receive this training:
□□

☒ As part of my residency.
☒ A special program.
☐ As part of my fellowship.

SECTION C: Attitudes towards immigrant health

A.□Please indicate your level of agreement with the following statements regarding immigrant and refugee health by checking the box that best represents your opinion.

a. I enjoy taking care of immigrants and refugees.

◯ Never
◯ Rarely
◯ Sometimes
⊗ Usually
◯ Always

b. Please indicate the reasons that you enjoy taking care of immigrants and refugees (may choose more than one).

☐ Tropical and other conditions not frequently diagnosed in US-born patients
☒ Learning about other cultures
☐ They don't complain as much
☐ Being able to hear their stories
☐ Their care is more complicated
☐ Their care is less complicated
☒ They are very appreciative of your help.
☐ They are extremely vulnerable
☐ Other:

c. Taking care of immigrants and refugees is more challenging than taking care of US born patients.

◯ Never
◯ Rarely
⊗ Sometimes
◯ Usually
◯ Always

d. Please mark all the challenges that you face as a provider when providing care to immigrants and refugees (may choose more than one):

- [x] Language barriers
- [ ] Insurance barriers
- [x] Cultural barriers
- [x] Finding a professional interpreter
- [ ] Knowing how to work with a professional interpreter
- [x] Time constraints
- [x] My own knowledge related to tropical and travel medicine
- [ ] Transportation problems for the patient
- [ ] Patients not understanding treatment plan
- [ ] Patients not following treatment plan
- [ ] My lack of knowledge regarding the patient's culture
- [ ] Bias or stereotyping
- [ ] Other:

e. Please mark all of the challenges faced by immigrant and refugee populations when receiving healthcare that you have perceived or witnessed (may choose more than one):

- [x] Language barriers
- [x] Insurance barriers
- [x] Cultural barriers
- [ ] Finding a professional interpreter
- [ ] Knowing how to work with a professional interpreter
- [ ] Time constraints
- [ ] Insufficiently trained health care providers
- [x] Transportation problems for the patient
- [ ] Food insufficiency
- [ ] Need for child care
- [x] Patients not understanding treatment plan
- [ ] Patients not following treatment plan
- [ ] My lack of knowledge regarding the patient's culture
- [ ] Bias or stereotyping
- [x] Trust issues
- [ ] Other...

f. Rank how well immigrants and refugees understand the healthcare that you are trying to provide.

- ( ) Significantly less than a US born individual
- (x) Less than a US born individual
- ( ) Equivalent to a US born individual
- ( ) More than a US born patient
- ( ) Significantly more than a US born individual

g. Immigrants and refugees adhere to treatment plans and follow my recommendations.

- ( ) Never
- ( ) Rarely
- ( ) Sometimes
- (x) Usually
- ( ) Always

h. Immigrants and refugees should receive the same care and insurance coverage as US born patients.

- ( ) Never
- ( ) Rarely
- ( ) Sometimes
- ( ) Usually
- (x) Always

i. Immigrants and refugees who are undocumented should receive the same care and insurance coverage as US born patients.

- ( ) Never
- ( ) Rarely
- ( ) Sometimes
- (x) Usually
- ( ) Always

j. Every physician is professionally obligated to care for immigrants and refugees if they present to your clinic or hospital.

- ( ) Strongly disagree
- ( ) Disagree
- ( ) No opinion
- (x) Agree
- ( ) Strongly agree

k. Is healthcare a human right?
- ◯ Yes
- ⊗ No

B. If you wish, please tell us about what you enjoy or do not enjoy about immigrant and refugee health care and the greatest challenges you face in caring for this population. ______________________________________

SECTION D: DEMOGRAPHIC INFORMATION
Please answer the following questions by checking the box in front of the response choice that best describes you.

a. Your age?
- ◯ 20 to 24
- ⊗ 25 to 29
- ◯ 30 to 34
- ◯ 35 to 39
- ◯ 40 or older

b. Your gender?
- ⊗ Female
- ◯ Male
- ◯ Other

c.Are you Hispanic or Latino?
- ◯ Yes
- ⊗ No

d. What is your race? (Select one or more responses)
- ◯ American Indian or Alaska Native
- ◯ Asian (Please specify):
- ◯ Black or African American
- ◯ Native Hawaiian or Other Pacific Islander
- ⊗ White
- ◯ Other (Please specify):

e.Were you born in the United States?
- ⊗ Yes
- ◯ No

g. Your residency year?
- ◯ PGY1
- ⊗ PGY2
- ◯ PGY3
- ◯ PGY4
- ◯ PGY5

h. How would you classify your political ideology?
- ◯ Conservative
- ◯ Somewhat conservative
- ◯ Moderate
- ⊗ Somewhat liberal
- ◯ Liberal
- ◯ Other (Please specify):

i. Estimated level of educational debt?
- ◯ None
- ◯ Less than $50,000
- ◯ $50,000 - $100,000
- ⊗ $100,000 - $200,000
- ◯ $200,000 or more

j.Do you plan to subspecialize?
- ◯ Yes
- ⊗ No

k. Languages spoken?

- ☒ English
- ☐ Spanish
- ☒ French
- ☐ Hmong
- ☐ Somali
- ☐ Japanese
- ☐ Chinese
- ☐ Russian
- ☐ Ethiopian
- ☐ Other ___________

l. Are you in the Global Health Pathway?

- ◯ Yes
- ⊗ No

m. Did you earn your degree in the US?

- ⊗ Yes
- ◯ No

n. What residency program are you in?

- ◯ Internal Medicine
- ◯ Med-Peds
- ⊗ Pediatrics
- ◯ Family Practice
- ◯ Neurology
- ◯ Psychiatry
- ◯ ObGyn
- ◯ Neurosurgery
- ◯ General Surgery
- ◯ Orthopedic Surgery
- ◯ Urology
- ◯ Surgical sub-specialty (please specify in text box below)
- ◯ Non-clinical specialty (radiology, pathology; please specify in text box below)

Medical Trainees' attitudes, knowledge, and experience with immigrant and refugee health

Response was added on 11/01/2013 2:55pm.

SECTION A: Personal experience with immigrant and refugee health care.

A. Please indicate your level of agreement with the following statements regarding your personal experience with immigrant and refugee health care by checking the box that best represents your experience.

a. During my inpatient rotations, I take care of the following percentage of immigrant and refugee patients:

- ◯ None
- ◯ 0 -5%
- ◯ 5-10%
- ⊗ 10-25%
- ◯ > 25%

b. During my outpatient rotations, I take care of the following percentage of immigrant and refugee patients:

- ◯ None
- ◯ 0-10%
- ◯ 10 -25%
- ⊗ 25-50%
- ◯ 50-75%
- ◯ >75%

c. I would like to take care of more immigrant and refugee patients.

- ◯ Strongly disagree
- ◯ Disagree
- ⊗ No opinion
- ◯ Agree
- ◯ Strongly agree

d. I plan to take care of immigrants and refugees when I finish residency.

- ◯ Strongly disagree
- ◯ Disagree
- ⊗ No opinion
- ◯ Agree
- ◯ Strongly agree

e. I plan to do short term (< 6 months) international work when I finish residency.

- ◯ Strongly disagree
- ⊗ Disagree
- ◯ No opinion
- ◯ Agree
- ◯ Strongly agree

f. I plan to do long term (>6 months) international work when I finish residency.

- ◯ Strongly disagree
- ⊗ Disagree
- ◯ No opinion
- ◯ Agree
- ◯ Strongly agree

g. I plan to work in health disparities in the following way after residency:

Not sure

SECTION B: MEDICAL EDUCATION

A. Please indicate your level of agreement with the following statements regarding your medical education and knowledge about immigrants and refugees by checking the box that best represents your opinion.

a. I have received specialized training in immigrant and refugee health, tropical medicine, or cross-cultural health.

- ◯ Strongly disagree
- ⊗ Disagree
- ◯ No opinion
- ◯ Agree
- ◯ Strongly agree

c. I feel comfortable with my fund of knowledge regarding immigrant and refugee health.

- ◯ Strongly disagree
- ◯ Disagree
- ⊗ No opinion
- ◯ Agree
- ◯ Strongly agree

d. I would like to have further training in immigrant and refugee health.

- ◯ Strongly disagree
- ◯ Disagree
- ◯ No opinion
- ⊗ Agree
- ◯ Strongly agree

e. If you agree with the above, please indicate all the contexts in which you would like to receive this training:

- ☒ As part of my residency.
- ☐ A special program.
- ☐ As part of my fellowship.

SECTION C: Attitudes towards immigrant health

A. Please indicate your level of agreement with the following statements regarding immigrant and refugee health by checking the box that best represents your opinion.

a. I enjoy taking care of immigrants and refugees.

- ◯ Never
- ◯ Rarely
- ⊗ Sometimes
- ◯ Usually
- ◯ Always

b. Please indicate the reasons that you enjoy taking care of immigrants and refugees (may choose more than one).

- ☐ Tropical and other conditions not frequently diagnosed in US-born patients
- ☒ Learning about other cultures
- ☐ They don't complain as much
- ☐ Being able to hear their stories
- ☐ Their care is more complicated
- ☐ Their care is less complicated
- ☒ They are very appreciative of your help.
- ☐ They are extremely vulnerable
- ☐ Other:

c. Taking care of immigrants and refugees is more challenging than taking care of US born patients.

- ◯ Never
- ◯ Rarely
- ◯ Sometimes
- ⊗ Usually
- ◯ Always

d. Please mark all the challenges that you face as a provider when providing care to immigrants and refugees (may choose more than one):

- ☒ Language barriers
- ☒ Insurance barriers
- ☒ Cultural barriers
- ☒ Finding a professional interpreter
- ☒ Knowing how to work with a professional interpreter
- ☒ Time constraints
- ☒ My own knowledge related to tropical and travel medicine
- ☒ Transportation problems for the patient
- ☒ Patients not understanding treatment plan
- ☒ Patients not following treatment plan
- ☒ My lack of knowledge regarding the patient's culture
- ☐ Bias or stereotyping
- ☐ Other:

e. Please mark all of the challenges faced by immigrant and refugee populations when receiving healthcare that you have perceived or witnessed (may choose more than one):

- ☒ Language barriers
- ☒ Insurance barriers
- ☒ Cultural barriers
- ☒ Finding a professional interpreter
- ☒ Knowing how to work with a professional interpreter
- ☐ Time constraints
- ☐ Insufficiently trained health care providers
- ☒ Transportation problems for the patient
- ☒ Food insufficiency
- ☒ Need for child care
- ☒ Patients not understanding treatment plan
- ☐ Patients not following treatment plan
- ☒ My lack of knowledge regarding the patient's culture
- ☒ Bias or stereotyping
- ☐ Trust issues
- ☐ Other...

f. Rank how well immigrants and refugees understand the healthcare that you are trying to provide.

- ⊗ Significantly less than a US born individual
- ◯ Less than a US born individual
- ◯ Equivalent to a US born individual
- ◯ More than a US born patient
- ◯ Significantly more than a US born individual

g. Immigrants and refugees adhere to treatment plans and follow my recommendations.

- ◯ Never
- ◯ Rarely
- ⊗ Sometimes
- ◯ Usually
- ◯ Always

h. Immigrants and refugees should receive the same care and insurance coverage as US born patients.

- ◯ Never
- ◯ Rarely
- ◯ Sometimes
- ◯ Usually
- ⊗ Always

i. Immigrants and refugees who are undocumented should receive the same care and insurance coverage as US born patients.

- ◯ Never
- ◯ Rarely
- ◯ Sometimes
- ◯ Usually
- ⊗ Always

j. Every physician is professionally obligated to care for immigrants and refugees if they present to your clinic or hospital.

- ◯ Strongly disagree
- ◯ Disagree
- ◯ No opinion
- ◯ Agree
- ⊗ Strongly agree

k. Is healthcare a human right?
⊗ Yes
○ No

B. If you wish, please tell us about what you enjoy or do not enjoy about immigrant and refugee health care and the greatest challenges you face in caring for this population.
____

SECTION D: DEMOGRAPHIC INFORMATION
Please answer the following questions by checking the box in front of the response choice that best describes you.

a. Your age?
○ 20 to 24
⊗ 25 to 29
○ 30 to 34
○ 35 to 39
○ 40 or older

b. Your gender?
⊗ Female
○ Male
○ Other

c. Are you Hispanic or Latino?
○ Yes
⊗ No

d. What is your race? (Select one or more responses)
○ American Indian or Alaska Native
○ Asian (Please specify):
○ Black or African American
○ Native Hawaiian or Other Pacific Islander
⊗ White
○ Other (Please specify):

e. Were you born in the United States?
⊗ Yes
○ No

g. Your residency year?
⊗ PGY1
○ PGY2
○ PGY3
○ PGY4
○ PGY5

h. How would you classify your political ideology?
○ Conservative
○ Somewhat conservative
○ Moderate
⊗ Somewhat liberal
○ Liberal
○ Other (Please specify):

i. Estimated level of educational debt?
○ None
⊗ Less than $50,000
○ $50,000 - $100,000
○ $100,000 - $200,000
○ $200,000 or more

j. Do you plan to subspecialize?
○ Yes
⊗ No

k. Languages spoken?

- ☒ English
- ☐ Spanish
- ☐ French
- ☐ Hmong
- ☐ Somali
- ☒ Japanese
- ☐ Chinese
- ☐ Russian
- ☐ Ethiopian
- ☐ Other __________

l. Are you in the Global Health Pathway?

- ◯ Yes
- ⊗ No

m.□ Did you earn your degree in the US?

- ⊗ Yes
- ◯ No

n. What residency program are you in?

- ◯ Internal Medicine
- ◯ Med-Peds
- ◯ Pediatrics
- ⊗ Family Practice
- ◯ Neurology
- ◯ Psychiatry
- ◯ ObGyn
- ◯ Neurosurgery
- ◯ General Surgery
- ◯ Orthopedic Surgery
- ◯ Urology
- ◯ Surgical sub-specialty (please specify in text box below)
- ◯ Non-clinical specialty (radiology, pathology; please specify in text box below)

Medical Trainees' attitudes, knowledge, and experience with immigrant and refugee health

Response was added on 11/01/2013 3:02pm.

SECTION A: Personal experience with immigrant and refugee health care.

A. Please indicate your level of agreement with the following statements regarding your personal experience with immigrant and refugee health care by checking the box that best represents your experience.

a. During my inpatient rotations, I take care of the following percentage of immigrant and refugee patients:

- ◯ None
- ◯ 0 -5%
- ◯ 5-10%
- ⊗ 10-25%
- ◯ > 25%

b. During my outpatient rotations, I take care of the following percentage of immigrant and refugee patients:

- ◯ None
- ◯ 0-10%
- ◯ 10 -25%
- ⊗ 25-50%
- ◯ 50-75%
- ◯ >75%

c. I would like to take care of more immigrant and refugee patients.

- ◯ Strongly disagree
- ◯ Disagree
- ⊗ No opinion
- ◯ Agree
- ◯ Strongly agree

d. I plan to take care of immigrants and refugees when I finish residency.

- ◯ Strongly disagree
- ◯ Disagree
- ◯ No opinion
- ⊗ Agree
- ◯ Strongly agree

e. I plan to do short term (< 6 months) international work when I finish residency.

- ◯ Strongly disagree
- ◯ Disagree
- ◯ No opinion
- ⊗ Agree
- ◯ Strongly agree

f. I plan to do long term (>6 months) international work when I finish residency.

- ◯ Strongly disagree
- ⊗ Disagree
- ◯ No opinion
- ◯ Agree
- ◯ Strongly agree

g. I plan to work in health disparities in the following way after residency:

Hospital care in MN, likely some volunteer work here and internationally

SECTION B: MEDICAL EDUCATION

A. Please indicate your level of agreement with the following statements regarding your medical education and knowledge about immigrants and refugees by checking the box that best represents your opinion.

a. I have received specialized training in immigrant and refugee health, tropical medicine, or cross-cultural health.

- ◯ Strongly disagree
- ◯ Disagree
- ◯ No opinion
- ⊗ Agree
- ◯ Strongly agree

b. If you have received specialized training in immigrant and refugee health, tropical medicine, or cross-cultural health, please indicate all the contexts in which you received this training:

- ☐ As an undergraduate.
- ☐ As a medical student.
- ☒ As part of my residency.
- ☒ A special program.
- ☐ As part of my fellowship.
- ☐ As part of a degree program (e.g. MPH)
- ☐ Other:

c. I feel comfortable with my fund of knowledge regarding immigrant and refugee health.

- ◯ Strongly disagree
- ⊗ Disagree
- ◯ No opinion
- ◯ Agree
- ◯ Strongly agree

d. I would like to have further training in immigrant and refugee health.

- ◯ Strongly disagree
- ◯ Disagree
- ◯ No opinion
- ⊗ Agree
- ◯ Strongly agree

e. If you agree with the above, please indicate all the contexts in which you would like to receive this training:

- ☐ As part of my residency.
- ☒ A special program.
- ☐ As part of my fellowship.

SECTION C: Attitudes towards immigrant health

A. Please indicate your level of agreement with the following statements regarding immigrant and refugee health by checking the box that best represents your opinion.

a. I enjoy taking care of immigrants and refugees.

- ◯ Never
- ◯ Rarely
- ◯ Sometimes
- ⊗ Usually
- ◯ Always

b. Please indicate the reasons that you enjoy taking care of immigrants and refugees (may choose more than one).

- ☒ Tropical and other conditions not frequently diagnosed in US-born patients
- ☒ Learning about other cultures
- ☐ They don't complain as much
- ☒ Being able to hear their stories
- ☐ Their care is more complicated
- ☐ Their care is less complicated
- ☐ They are very appreciative of your help.
- ☐ They are extremely vulnerable
- ☐ Other:

c. Taking care of immigrants and refugees is more challenging than taking care of US born patients.

- ◯ Never
- ◯ Rarely
- ◯ Sometimes
- ⊗ Usually
- ◯ Always

d. Please mark all the challenges that you face as a provider when providing care to immigrants and refugees (may choose more than one):

- ☒ Language barriers
- ☒ Insurance barriers
- ☒ Cultural barriers
- ☒ Finding a professional interpreter
- ☐ Knowing how to work with a professional interpreter
- ☒ Time constraints
- ☒ My own knowledge related to tropical and travel medicine
- ☒ Transportation problems for the patient
- ☒ Patients not understanding treatment plan
- ☒ Patients not following treatment plan
- ☒ My lack of knowledge regarding the patient's culture
- ☒ Bias or stereotyping
- ☐ Other:

e. Please mark all of the challenges faced by immigrant and refugee populations when receiving healthcare that you have perceived or witnessed (may choose more than one):

- ☒ Language barriers
- ☐ Insurance barriers
- ☐ Cultural barriers
- ☐ Finding a professional interpreter
- ☐ Knowing how to work with a professional interpreter
- ☐ Time constraints
- ☐ Insufficiently trained health care providers
- ☒ Transportation problems for the patient
- ☐ Food insufficiency
- ☐ Need for child care
- ☐ Patients not understanding treatment plan
- ☒ Patients not following treatment plan
- ☐ My lack of knowledge regarding the patient's culture
- ☐ Bias or stereotyping
- ☒ Trust issues
- ☒ Other...

Counter information provided by people within culture (Somali respresentatives speak to community against MRSA)

f. Rank how well immigrants and refugees understand the healthcare that you are trying to provide.

- ◯ Significantly less than a US born individual
- ⊗ Less than a US born individual
- ◯ Equivalent to a US born individual
- ◯ More than a US born patient
- ◯ Significantly more than a US born individual

g. Immigrants and refugees adhere to treatment plans and follow my recommendations.

- ◯ Never
- ◯ Rarely
- ⊗ Sometimes
- ◯ Usually
- ◯ Always

h. Immigrants and refugees should receive the same care and insurance coverage as US born patients.

- ◯ Never
- ◯ Rarely
- ◯ Sometimes
- ◯ Usually
- ⊗ Always

i. Immigrants and refugees who are undocumented should receive the same care and insurance coverage as US born patients.

- ⊗ Never
- ◯ Rarely
- ◯ Sometimes
- ◯ Usually
- ◯ Always

j. Every physician is professionally obligated to care for immigrants and refugees if they present to your clinic or hospital.

- ◯ Strongly disagree
- ◯ Disagree
- ◯ No opinion
- ◯ Agree
- ⊗ Strongly agree

k. Is healthcare a human right?

- ⊗ Yes
- ◯ No

B. If you wish, please tell us about what you enjoy or do not enjoy about immigrant and refugee health care and the greatest challenges you face in caring for this population.

I don't think insurance should cover undocumented individuals, but they should receive equal care.
I find more immegrants and refugee's over-utilizing emergency care and under utilizing or not using primary care.

SECTION D: DEMOGRAPHIC INFORMATION
Please answer the following questions by checking the box in front of the response choice that best describes you.

a. Your age?

- ◯ 20 to 24
- ⊗ 25 to 29
- ◯ 30 to 34
- ◯ 35 to 39
- ◯ 40 or older

b. Your gender?

- ⊗ Female
- ◯ Male
- ◯ Other

c.Are you Hispanic or Latino?

- ◯ Yes
- ⊗ No

d. What is your race? (Select one or more responses)

- ◯ American Indian or Alaska Native
- ◯ Asian (Please specify):
- ◯ Black or African American
- ◯ Native Hawaiian or Other Pacific Islander
- ⊗ White
- ◯ Other (Please specify):

e.Were you born in the United States?

- ⊗ Yes
- ◯ No

g. Your residency year?

- ◯ PGY1
- ◯ PGY2
- ⊗ PGY3
- ◯ PGY4
- ◯ PGY5

h. How would you classify your political ideology?

- ◯ Conservative
- ⊗ Somewhat conservative
- ◯ Moderate
- ◯ Somewhat liberal
- ◯ Liberal
- ◯ Other (Please specify):

i. Estimated level of educational debt?

- ◯ None
- ◯ Less than $50,000
- ◯ $50,000 - $100,000
- ◯ $100,000 - $200,000
- ⊗ $200,000 or more

j.Do you plan to subspecialize?

- ◯ Yes
- ⊗ No

k. Languages spoken?

- ☒ English
- ☐ Spanish
- ☐ French
- ☐ Hmong
- ☐ Somali
- ☐ Japanese
- ☐ Chinese
- ☐ Russian
- ☐ Ethiopian
- ☐ Other __________

l. Are you in the Global Health Pathway?

- ⊗ Yes
- ◯ No

m. Did you earn your degree in the US?

- ⊗ Yes
- ◯ No

n. What residency program are you in?

- ◯ Internal Medicine
- ◯ Med-Peds
- ⊗ Pediatrics
- ◯ Family Practice
- ◯ Neurology
- ◯ Psychiatry
- ◯ ObGyn
- ◯ Neurosurgery
- ◯ General Surgery
- ◯ Orthopedic Surgery
- ◯ Urology
- ◯ Surgical sub-specialty (please specify in text box below)
- ◯ Non-clinical specialty (radiology, pathology; please specify in text box below)

Medical Trainees' attitudes, knowledge, and experience with immigrant and refugee health

Response was added on 11/01/2013 3:07pm.

SECTION A: Personal experience with immigrant and refugee health care.

A. Please indicate your level of agreement with the following statements regarding your personal experience with immigrant and refugee health care by checking the box that best represents your experience.

a. During my inpatient rotations, I take care of the following percentage of immigrant and refugee patients:

- ◯ None
- ◯ 0 -5%
- ⊗ 5-10%
- ◯ 10-25%
- ◯ > 25%

b. During my outpatient rotations, I take care of the following percentage of immigrant and refugee patients:

- ◯ None
- ⊗ 0-10%
- ◯ 10 -25%
- ◯ 25-50%
- ◯ 50-75%
- ◯ >75%

c. I would like to take care of more immigrant and refugee patients.

- ◯ Strongly disagree
- ◯ Disagree
- ◯ No opinion
- ⊗ Agree
- ◯ Strongly agree

d. I plan to take care of immigrants and refugees when I finish residency.

- ◯ Strongly disagree
- ◯ Disagree
- ◯ No opinion
- ⊗ Agree
- ◯ Strongly agree

e. I plan to do short term (< 6 months) international work when I finish residency.

- ◯ Strongly disagree
- ◯ Disagree
- ◯ No opinion
- ⊗ Agree
- ◯ Strongly agree

f. I plan to do long term (>6 months) international work when I finish residency.

- ◯ Strongly disagree
- ⊗ Disagree
- ◯ No opinion
- ◯ Agree
- ◯ Strongly agree

g. I plan to work in health disparities in the following way after residency:

By providing the best possible care that I can to every patient presented to me.

SECTION B: MEDICAL EDUCATION

A. Please indicate your level of agreement with the following statements regarding your medical education and knowledge about immigrants and refugees by checking the box that best represents your opinion.

a. I have received specialized training in immigrant and refugee health, tropical medicine, or cross-cultural health.

- ◯ Strongly disagree
- ◯ Disagree
- ◯ No opinion
- ⊗ Agree
- ◯ Strongly agree

b. If you have received specialized training in immigrant and refugee health, tropical medicine, or cross-cultural health, please indicate all the contexts in which you received this training:

- ☐ As an undergraduate.
- ☒ As a medical student.
- ☒ As part of my residency.
- ☐ A special program.
- ☐ As part of my fellowship.
- ☐ As part of a degree program (e.g. MPH)
- ☐ Other:

c. I feel comfortable with my fund of knowledge regarding immigrant and refugee health.

- ◯ Strongly disagree
- ◯ Disagree
- ◯ No opinion
- ⊗ Agree
- ◯ Strongly agree

d. I would like to have further training in immigrant and refugee health.

- ◯ Strongly disagree
- ◯ Disagree
- ◯ No opinion
- ⊗ Agree
- ◯ Strongly agree

e. If you agree with the above, please indicate all the contexts in which you would like to receive this training:

- ☒ As part of my residency.
- ☐ A special program.
- ☐ As part of my fellowship.

SECTION C: Attitudes towards immigrant health

A. Please indicate your level of agreement with the following statements regarding immigrant and refugee health by checking the box that best represents your opinion.

a. I enjoy taking care of immigrants and refugees.

- ◯ Never
- ◯ Rarely
- ◯ Sometimes
- ⊗ Usually
- ◯ Always

b. Please indicate the reasons that you enjoy taking care of immigrants and refugees (may choose more than one).

- ☒ Tropical and other conditions not frequently diagnosed in US-born patients
- ☒ Learning about other cultures
- ☐ They don't complain as much
- ☐ Being able to hear their stories
- ☐ Their care is more complicated
- ☐ Their care is less complicated
- ☐ They are very appreciative of your help.
- ☐ They are extremely vulnerable
- ☐ Other:

c. Taking care of immigrants and refugees is more challenging than taking care of US born patients.

- ◯ Never
- ◯ Rarely
- ◯ Sometimes
- ⊗ Usually
- ◯ Always

d. Please mark all the challenges that you face as a provider when providing care to immigrants and refugees (may choose more than one):

- ☒ Language barriers
- ☒ Insurance barriers
- ☒ Cultural barriers
- ☒ Finding a professional interpreter
- ☐ Knowing how to work with a professional interpreter
- ☐ Time constraints
- ☐ My own knowledge related to tropical and travel medicine
- ☐ Transportation problems for the patient
- ☐ Patients not understanding treatment plan
- ☐ Patients not following treatment plan
- ☐ My lack of knowledge regarding the patient's culture
- ☐ Bias or stereotyping
- ☐ Other:

e. Please mark all of the challenges faced by immigrant and refugee populations when receiving healthcare that you have perceived or witnessed (may choose more than one):

- ☒ Language barriers
- ☒ Insurance barriers
- ☒ Cultural barriers
- ☐ Finding a professional interpreter
- ☐ Knowing how to work with a professional interpreter
- ☐ Time constraints
- ☐ Insufficiently trained health care providers
- ☐ Transportation problems for the patient
- ☐ Food insufficiency
- ☐ Need for child care
- ☐ Patients not understanding treatment plan
- ☐ Patients not following treatment plan
- ☐ My lack of knowledge regarding the patient's culture
- ☐ Bias or stereotyping
- ☐ Trust issues
- ☐ Other...

f. Rank how well immigrants and refugees understand the healthcare that you are trying to provide.

- ◯ Significantly less than a US born individual
- ◯ Less than a US born individual
- ⊗ Equivalent to a US born individual
- ◯ More than a US born patient
- ◯ Significantly more than a US born individual

g. Immigrants and refugees adhere to treatment plans and follow my recommendations.

- ◯ Never
- ◯ Rarely
- ⊗ Sometimes
- ◯ Usually
- ◯ Always

h. Immigrants and refugees should receive the same care and insurance coverage as US born patients.

- ◯ Never
- ◯ Rarely
- ◯ Sometimes
- ◯ Usually
- ⊗ Always

i. Immigrants and refugees who are undocumented should receive the same care and insurance coverage as US born patients.

- ◯ Never
- ◯ Rarely
- ◯ Sometimes
- ◯ Usually
- ⊗ Always

j. Every physician is professionally obligated to care for immigrants and refugees if they present to your clinic or hospital.

- ◯ Strongly disagree
- ◯ Disagree
- ◯ No opinion
- ⊗ Agree
- ◯ Strongly agree

k. Is healthcare a human right?
- ⊗ Yes
- ○ No

B. If you wish, please tell us about what you enjoy or do not enjoy about immigrant and refugee health care and the greatest challenges you face in caring for this population.

____

SECTION D: DEMOGRAPHIC INFORMATION
Please answer the following questions by checking the box in front of the response choice that best describes you.

a. Your age?
- ○ 20 to 24
- ⊗ 25 to 29
- ○ 30 to 34
- ○ 35 to 39
- ○ 40 or older

b. Your gender?
- ○ Female
- ⊗ Male
- ○ Other

c.Are you Hispanic or Latino?
- ○ Yes
- ⊗ No

d. What is your race? (Select one or more responses)
- ○ American Indian or Alaska Native
- ⊗ Asian (Please specify):
- ○ Black or African American
- ○ Native Hawaiian or Other Pacific Islander
- ○ White
- ○ Other (Please specify):

e.Were you born in the United States?
- ○ Yes
- ⊗ No

f.If not, in what country were you born?

Republic of China, Taiwan

g. Your residency year?
- ○ PGY1
- ○ PGY2
- ⊗ PGY3
- ○ PGY4
- ○ PGY5

h. How would you classify your political ideology?
- ○ Conservative
- ○ Somewhat conservative
- ⊗ Moderate
- ○ Somewhat liberal
- ○ Liberal
- ○ Other (Please specify):

i. Estimated level of educational debt?
- ⊗ None
- ○ Less than $50,000
- ○ $50,000 - $100,000
- ○ $100,000 - $200,000
- ○ $200,000 or more

j.Do you plan to subspecialize?
- ⊗ Yes
- ○ No

k. Languages spoken?

- ☒ English
- ☐ Spanish
- ☐ French
- ☐ Hmong
- ☐ Somali
- ☐ Japanese
- ☒ Chinese
- ☐ Russian
- ☐ Ethiopian
- ☐ Other __________

l. Are you in the Global Health Pathway?

- ○ Yes
- ⊗ No

m. Did you earn your degree in the US?

- ○ Yes
- ⊗ No

n. What residency program are you in?

- ⊗ Internal Medicine
- ○ Med-Peds
- ○ Pediatrics
- ○ Family Practice
- ○ Neurology
- ○ Psychiatry
- ○ ObGyn
- ○ Neurosurgery
- ○ General Surgery
- ○ Orthopedic Surgery
- ○ Urology
- ○ Surgical sub-specialty (please specify in text box below)
- ○ Non-clinical specialty (radiology, pathology; please specify in text box below)

Medical Trainees' attitudes, knowledge, and experience with immigrant and refugee health

Response was added on 11/01/2013 3:07pm.

SECTION A: Personal experience with immigrant and refugee health care.

A. Please indicate your level of agreement with the following statements regarding your personal experience with immigrant and refugee health care by checking the box that best represents your experience.

a. During my inpatient rotations, I take care of the following percentage of immigrant and refugee patients:

- ◯ None
- ◯ 0 -5%
- ◯ 5-10%
- ◯ 10-25%
- ⊗ > 25%

b. During my outpatient rotations, I take care of the following percentage of immigrant and refugee patients:

- ◯ None
- ◯ 0-10%
- ◯ 10 -25%
- ◯ 25-50%
- ⊗ 50-75%
- ◯ >75%

c. I would like to take care of more immigrant and refugee patients.

- ◯ Strongly disagree
- ◯ Disagree
- ◯ No opinion
- ⊗ Agree
- ◯ Strongly agree

d. I plan to take care of immigrants and refugees when I finish residency.

- ◯ Strongly disagree
- ◯ Disagree
- ◯ No opinion
- ⊗ Agree
- ◯ Strongly agree

e. I plan to do short term (< 6 months) international work when I finish residency.

- ◯ Strongly disagree
- ◯ Disagree
- ⊗ No opinion
- ◯ Agree
- ◯ Strongly agree

f. I plan to do long term (>6 months) international work when I finish residency.

- ◯ Strongly disagree
- ⊗ Disagree
- ◯ No opinion
- ◯ Agree
- ◯ Strongly agree

g. I plan to work in health disparities in the following way after residency: outpatient in underserved area

SECTION B: MEDICAL EDUCATION

A. Please indicate your level of agreement with the following statements regarding your medical education and knowledge about immigrants and refugees by checking the box that best represents your opinion.

a. I have received specialized training in immigrant and refugee health, tropical medicine, or cross-cultural health.

- ◯ Strongly disagree
- ◯ Disagree
- ◯ No opinion
- ⊗ Agree
- ◯ Strongly agree

b. If you have received specialized training in immigrant and refugee health, tropical medicine, or cross-cultural health, please indicate all the contexts in which you received this training:

- ☐ As an undergraduate.
- ☒ As a medical student.
- ☒ As part of my residency.
- ☐ A special program.
- ☐ As part of my fellowship.
- ☐ As part of a degree program (e.g. MPH)
- ☐ Other:

c. I feel comfortable with my fund of knowledge regarding immigrant and refugee health.

- ◯ Strongly disagree
- ◯ Disagree
- ⊗ No opinion
- ◯ Agree
- ◯ Strongly agree

d. I would like to have further training in immigrant and refugee health.

- ◯ Strongly disagree
- ◯ Disagree
- ◯ No opinion
- ⊗ Agree
- ◯ Strongly agree

e. If you agree with the above, please indicate all the contexts in which you would like to receive this training:
□□

- ☒ As part of my residency.
- ☐ A special program.
- ☐ As part of my fellowship.

SECTION C: Attitudes towards immigrant health

A.□Please indicate your level of agreement with the following statements regarding immigrant and refugee health by checking the box that best represents your opinion.

a. I enjoy taking care of immigrants and refugees.

- ◯ Never
- ◯ Rarely
- ◯ Sometimes
- ◯ Usually
- ⊗ Always

b. Please indicate the reasons that you enjoy taking care of immigrants and refugees (may choose more than one).

- ☒ Tropical and other conditions not frequently diagnosed in US-born patients
- ☐ Learning about other cultures
- ☐ They don't complain as much
- ☒ Being able to hear their stories
- ☒ Their care is more complicated
- ☐ Their care is less complicated
- ☒ They are very appreciative of your help.
- ☒ They are extremely vulnerable
- ☐ Other:

c. Taking care of immigrants and refugees is more challenging than taking care of US born patients.

- ◯ Never
- ◯ Rarely
- ◯ Sometimes
- ⊗ Usually
- ◯ Always

d. Please mark all the challenges that you face as a provider when providing care to immigrants and refugees (may choose more than one):

- ☒ Language barriers
- ☒ Insurance barriers
- ☒ Cultural barriers
- ☐ Finding a professional interpreter
- ☐ Knowing how to work with a professional interpreter
- ☒ Time constraints
- ☒ My own knowledge related to tropical and travel medicine
- ☒ Transportation problems for the patient
- ☒ Patients not understanding treatment plan
- ☒ Patients not following treatment plan
- ☒ My lack of knowledge regarding the patient's culture
- ☒ Bias or stereotyping
- ☐ Other:

e. Please mark all of the challenges faced by immigrant and refugee populations when receiving healthcare that you have perceived or witnessed (may choose more than one):

- ☒ Language barriers
- ☒ Insurance barriers
- ☒ Cultural barriers
- ☐ Finding a professional interpreter
- ☐ Knowing how to work with a professional interpreter
- ☐ Time constraints
- ☒ Insufficiently trained health care providers
- ☒ Transportation problems for the patient
- ☒ Food insufficiency
- ☒ Need for child care
- ☒ Patients not understanding treatment plan
- ☒ Patients not following treatment plan
- ☒ My lack of knowledge regarding the patient's culture
- ☒ Bias or stereotyping
- ☒ Trust issues
- ☐ Other...

f. Rank how well immigrants and refugees understand the healthcare that you are trying to provide.

- ○ Significantly less than a US born individual
- ⊗ Less than a US born individual
- ○ Equivalent to a US born individual
- ○ More than a US born patient
- ○ Significantly more than a US born individual

g. Immigrants and refugees adhere to treatment plans and follow my recommendations.

- ○ Never
- ○ Rarely
- ○ Sometimes
- ⊗ Usually
- ○ Always

h. Immigrants and refugees should receive the same care and insurance coverage as US born patients.

- ○ Never
- ○ Rarely
- ○ Sometimes
- ○ Usually
- ⊗ Always

i. Immigrants and refugees who are undocumented should receive the same care and insurance coverage as US born patients.

- ○ Never
- ○ Rarely
- ○ Sometimes
- ○ Usually
- ⊗ Always

j. Every physician is professionally obligated to care for immigrants and refugees if they present to your clinic or hospital.

- ○ Strongly disagree
- ○ Disagree
- ○ No opinion
- ○ Agree
- ⊗ Strongly agree

k. Is healthcare a human right?
- ⊗ Yes
- ◯ No

B. If you wish, please tell us about what you enjoy or do not enjoy about immigrant and refugee health care and the greatest challenges you face in caring for this population. ______________________________________

SECTION D: DEMOGRAPHIC INFORMATION
Please answer the following questions by checking the box in front of the response choice that best describes you.

a. Your age?
- ◯ 20 to 24
- ⊗ 25 to 29
- ◯ 30 to 34
- ◯ 35 to 39
- ◯ 40 or older

b. Your gender?
- ⊗ Female
- ◯ Male
- ◯ Other

c.Are you Hispanic or Latino?
- ◯ Yes
- ⊗ No

d. What is your race? (Select one or more responses)
- ◯ American Indian or Alaska Native
- ◯ Asian (Please specify):
- ◯ Black or African American
- ◯ Native Hawaiian or Other Pacific Islander
- ⊗ White
- ◯ Other (Please specify):

e.Were you born in the United States?
- ◯ Yes
- ⊗ No

f.If not, in what country were you born? ______________________________

g. Your residency year?
- ◯ PGY1
- ◯ PGY2
- ⊗ PGY3
- ◯ PGY4
- ◯ PGY5

h. How would you classify your political ideology?
- ◯ Conservative
- ◯ Somewhat conservative
- ◯ Moderate
- ◯ Somewhat liberal
- ⊗ Liberal
- ◯ Other (Please specify):

i. Estimated level of educational debt?
- ◯ None
- ◯ Less than $50,000
- ⊗ $50,000 - $100,000
- ◯ $100,000 - $200,000
- ◯ $200,000 or more

j.Do you plan to subspecialize?
- ◯ Yes
- ⊗ No

k. Languages spoken?

- ☒ English
- ☐ Spanish
- ☒ French
- ☐ Hmong
- ☐ Somali
- ☐ Japanese
- ☐ Chinese
- ☐ Russian
- ☐ Ethiopian
- ☐ Other ___________

l. Are you in the Global Health Pathway?

- ⊗ Yes
- ◯ No

m. Did you earn your degree in the US?

- ⊗ Yes
- ◯ No

n. What residency program are you in?

- ◯ Internal Medicine
- ◯ Med-Peds
- ⊗ Pediatrics
- ◯ Family Practice
- ◯ Neurology
- ◯ Psychiatry
- ◯ ObGyn
- ◯ Neurosurgery
- ◯ General Surgery
- ◯ Orthopedic Surgery
- ◯ Urology
- ◯ Surgical sub-specialty (please specify in text box below)
- ◯ Non-clinical specialty (radiology, pathology; please specify in text box below)

Medical Trainees' attitudes, knowledge, and experience with immigrant and refugee health

Response was added on 11/01/2013 3:10pm.

SECTION A: Personal experience with immigrant and refugee health care.

A. Please indicate your level of agreement with the following statements regarding your personal experience with immigrant and refugee health care by checking the box that best represents your experience.

a. During my inpatient rotations, I take care of the following percentage of immigrant and refugee patients:

- ◯ None
- ◯ 0 -5%
- ⊗ 5-10%
- ◯ 10-25%
- ◯ > 25%

b. During my outpatient rotations, I take care of the following percentage of immigrant and refugee patients:

- ◯ None
- ⊗ 0-10%
- ◯ 10 -25%
- ◯ 25-50%
- ◯ 50-75%
- ◯ >75%

c. I would like to take care of more immigrant and refugee patients.

- ◯ Strongly disagree
- ◯ Disagree
- ⊗ No opinion
- ◯ Agree
- ◯ Strongly agree

d. I plan to take care of immigrants and refugees when I finish residency.

- ◯ Strongly disagree
- ◯ Disagree
- ◯ No opinion
- ⊗ Agree
- ◯ Strongly agree

e. I plan to do short term (< 6 months) international work when I finish residency.

- ◯ Strongly disagree
- ◯ Disagree
- ◯ No opinion
- ⊗ Agree
- ◯ Strongly agree

f. I plan to do long term (>6 months) international work when I finish residency.

- ⊗ Strongly disagree
- ◯ Disagree
- ◯ No opinion
- ◯ Agree
- ◯ Strongly agree

g. I plan to work in health disparities in the following way after residency: no

SECTION B: MEDICAL EDUCATION

A. Please indicate your level of agreement with the following statements regarding your medical education and knowledge about immigrants and refugees by checking the box that best represents your opinion.

a. I have received specialized training in immigrant and refugee health, tropical medicine, or cross-cultural health.

- ◯ Strongly disagree
- ◯ Disagree
- ◯ No opinion
- ⊗ Agree
- ◯ Strongly agree

b. If you have received specialized training in immigrant and refugee health, tropical medicine, or cross-cultural health, please indicate all the contexts in which you received this training:

- ☐ As an undergraduate.
- ☒ As a medical student.
- ☐ As part of my residency.
- ☐ A special program.
- ☐ As part of my fellowship.
- ☐ As part of a degree program (e.g. MPH)
- ☐ Other:

c. I feel comfortable with my fund of knowledge regarding immigrant and refugee health.

- ◯ Strongly disagree
- ◯ Disagree
- ⊗ No opinion
- ◯ Agree
- ◯ Strongly agree

d. I would like to have further training in immigrant and refugee health.

- ◯ Strongly disagree
- ◯ Disagree
- ◯ No opinion
- ⊗ Agree
- ◯ Strongly agree

e. If you agree with the above, please indicate all the contexts in which you would like to receive this training:
□□

- ☒ As part of my residency.
- ☐ A special program.
- ☒ As part of my fellowship.

SECTION C: Attitudes towards immigrant health

A.□Please indicate your level of agreement with the following statements regarding immigrant and refugee health by checking the box that best represents your opinion.

a. I enjoy taking care of immigrants and refugees.

- ◯ Never
- ◯ Rarely
- ◯ Sometimes
- ⊗ Usually
- ◯ Always

b. Please indicate the reasons that you enjoy taking care of immigrants and refugees (may choose more than one).

- ☐ Tropical and other conditions not frequently diagnosed in US-born patients
- ☒ Learning about other cultures
- ☐ They don't complain as much
- ☐ Being able to hear their stories
- ☐ Their care is more complicated
- ☐ Their care is less complicated
- ☐ They are very appreciative of your help.
- ☐ They are extremely vulnerable
- ☐ Other:

c. Taking care of immigrants and refugees is more challenging than taking care of US born patients.

- ◯ Never
- ◯ Rarely
- ◯ Sometimes
- ⊗ Usually
- ◯ Always

d. Please mark all the challenges that you face as a provider when providing care to immigrants and refugees (may choose more than one):

- ☒ Language barriers
- ☒ Insurance barriers
- ☒ Cultural barriers
- ☒ Finding a professional interpreter
- ☐ Knowing how to work with a professional interpreter
- ☒ Time constraints
- ☒ My own knowledge related to tropical and travel medicine
- ☒ Transportation problems for the patient
- ☒ Patients not understanding treatment plan
- ☒ Patients not following treatment plan
- ☒ My lack of knowledge regarding the patient's culture
- ☐ Bias or stereotyping
- ☐ Other:

e. Please mark all of the challenges faced by immigrant and refugee populations when receiving healthcare that you have perceived or witnessed (may choose more than one):

- ☒ Language barriers
- ☒ Insurance barriers
- ☒ Cultural barriers
- ☒ Finding a professional interpreter
- ☒ Knowing how to work with a professional interpreter
- ☒ Time constraints
- ☒ Insufficiently trained health care providers
- ☒ Transportation problems for the patient
- ☒ Food insufficiency
- ☒ Need for child care
- ☒ Patients not understanding treatment plan
- ☒ Patients not following treatment plan
- ☒ My lack of knowledge regarding the patient's culture
- ☒ Bias or stereotyping
- ☒ Trust issues
- ☐ Other...

f. Rank how well immigrants and refugees understand the healthcare that you are trying to provide.

- ⊗ Significantly less than a US born individual
- ○ Less than a US born individual
- ○ Equivalent to a US born individual
- ○ More than a US born patient
- ○ Significantly more than a US born individual

g. Immigrants and refugees adhere to treatment plans and follow my recommendations.

- ○ Never
- ○ Rarely
- ⊗ Sometimes
- ○ Usually
- ○ Always

h. Immigrants and refugees should receive the same care and insurance coverage as US born patients.

- ○ Never
- ○ Rarely
- ○ Sometimes
- ○ Usually
- ⊗ Always

i. Immigrants and refugees who are undocumented should receive the same care and insurance coverage as US born patients.

- ○ Never
- ○ Rarely
- ○ Sometimes
- ○ Usually
- ⊗ Always

j. Every physician is professionally obligated to care for immigrants and refugees if they present to your clinic or hospital.

- ○ Strongly disagree
- ○ Disagree
- ○ No opinion
- ○ Agree
- ⊗ Strongly agree

k. Is healthcare a human right?
- ⊗ Yes
- ◯ No

B. If you wish, please tell us about what you enjoy or do not enjoy about immigrant and refugee health care and the greatest challenges you face in caring for this population.

____

SECTION D: DEMOGRAPHIC INFORMATION
Please answer the following questions by checking the box in front of the response choice that best describes you.

a. Your age?
- ◯ 20 to 24
- ◯ 25 to 29
- ◯ 30 to 34
- ⊗ 35 to 39
- ◯ 40 or older

b. Your gender?
- ◯ Female
- ⊗ Male
- ◯ Other

c.Are you Hispanic or Latino?
- ◯ Yes
- ⊗ No

d. What is your race? (Select one or more responses)
- ◯ American Indian or Alaska Native
- ◯ Asian (Please specify):
- ◯ Black or African American
- ◯ Native Hawaiian or Other Pacific Islander
- ◯ White
- ⊗ Other (Please specify):

Nordic

e.Were you born in the United States?
- ⊗ Yes
- ◯ No

g. Your residency year?
- ◯ PGY1
- ◯ PGY2
- ◯ PGY3
- ⊗ PGY4
- ◯ PGY5

h. How would you classify your political ideology?
- ◯ Conservative
- ◯ Somewhat conservative
- ◯ Moderate
- ◯ Somewhat liberal
- ◯ Liberal
- ⊗ Other (Please specify):

Socialist

i. Estimated level of educational debt?
- ◯ None
- ◯ Less than $50,000
- ◯ $50,000 - $100,000
- ⊗ $100,000 - $200,000
- ◯ $200,000 or more

j.Do you plan to subspecialize?
- ⊗ Yes
- ◯ No

k. Languages spoken?

☒ English
☒ Spanish
☐ French
☐ Hmong
☐ Somali
☐ Japanese
☐ Chinese
☒ Russian
☐ Ethiopian
☐ Other ___________

l. Are you in the Global Health Pathway?

◯ Yes
⊗ No

m. Did you earn your degree in the US?

⊗ Yes
◯ No

n. What residency program are you in?

◯ Internal Medicine
◯ Med-Peds
◯ Pediatrics
◯ Family Practice
◯ Neurology
◯ Psychiatry
◯ ObGyn
◯ Neurosurgery
◯ General Surgery
◯ Orthopedic Surgery
◯ Urology
◯ Surgical sub-specialty (please specify in text box below)
⊗ Non-clinical specialty (radiology, pathology; please specify in text box below)

PM&R; Pain medicine fellowship matched for next year

Medical Trainees' attitudes, knowledge, and experience with immigrant and refugee health

Response was added on 11/01/2013 3:20pm.

SECTION A: Personal experience with immigrant and refugee health care.

A. Please indicate your level of agreement with the following statements regarding your personal experience with immigrant and refugee health care by checking the box that best represents your experience.

a. During my inpatient rotations, I take care of the following percentage of immigrant and refugee patients:
- ◯ None
- ◯ 0 -5%
- ⊗ 5-10%
- ◯ 10-25%
- ◯ > 25%

b. During my outpatient rotations, I take care of the following percentage of immigrant and refugee patients:
- ◯ None
- ⊗ 0-10%
- ◯ 10 -25%
- ◯ 25-50%
- ◯ 50-75%
- ◯ >75%

c. I would like to take care of more immigrant and refugee patients.
- ◯ Strongly disagree
- ◯ Disagree
- ⊗ No opinion
- ◯ Agree
- ◯ Strongly agree

d. I plan to take care of immigrants and refugees when I finish residency.
- ◯ Strongly disagree
- ◯ Disagree
- ◯ No opinion
- ⊗ Agree
- ◯ Strongly agree

e. I plan to do short term (< 6 months) international work when I finish residency.
- ◯ Strongly disagree
- ◯ Disagree
- ⊗ No opinion
- ◯ Agree
- ◯ Strongly agree

f. I plan to do long term (>6 months) international work when I finish residency.
- ◯ Strongly disagree
- ◯ Disagree
- ⊗ No opinion
- ◯ Agree
- ◯ Strongly agree

g. I plan to work in health disparities in the following way after residency:
n/a

SECTION B: MEDICAL EDUCATION

A. Please indicate your level of agreement with the following statements regarding your medical education and knowledge about immigrants and refugees by checking the box that best represents your opinion.

a. I have received specialized training in immigrant and refugee health, tropical medicine, or cross-cultural health.

- ◯ Strongly disagree
- ◯ Disagree
- ⊗ No opinion
- ◯ Agree
- ◯ Strongly agree

c. I feel comfortable with my fund of knowledge regarding immigrant and refugee health.

- ◯ Strongly disagree
- ⊗ Disagree
- ◯ No opinion
- ◯ Agree
- ◯ Strongly agree

d. I would like to have further training in immigrant and refugee health.

- ◯ Strongly disagree
- ◯ Disagree
- ⊗ No opinion
- ◯ Agree
- ◯ Strongly agree

SECTION C: Attitudes towards immigrant health

A.Please indicate your level of agreement with the following statements regarding immigrant and refugee health by checking the box that best represents your opinion.

a. I enjoy taking care of immigrants and refugees.

- ◯ Never
- ◯ Rarely
- ◯ Sometimes
- ⊗ Usually
- ◯ Always

b. Please indicate the reasons that you enjoy taking care of immigrants and refugees (may choose more than one).

- ☒ Tropical and other conditions not frequently diagnosed in US-born patients
- ☒ Learning about other cultures
- ☐ They don't complain as much
- ☐ Being able to hear their stories
- ☐ Their care is more complicated
- ☐ Their care is less complicated
- ☒ They are very appreciative of your help.
- ☐ They are extremely vulnerable
- ☐ Other:

c. Taking care of immigrants and refugees is more challenging than taking care of US born patients.

- ◯ Never
- ◯ Rarely
- ⊗ Sometimes
- ◯ Usually
- ◯ Always

d. Please mark all the challenges that you face as a provider when providing care to immigrants and refugees (may choose more than one):

- ☒ Language barriers
- ☐ Insurance barriers
- ☒ Cultural barriers
- ☒ Finding a professional interpreter
- ☐ Knowing how to work with a professional interpreter
- ☒ Time constraints
- ☒ My own knowledge related to tropical and travel medicine
- ☐ Transportation problems for the patient
- ☒ Patients not understanding treatment plan
- ☒ Patients not following treatment plan
- ☒ My lack of knowledge regarding the patient's culture
- ☐ Bias or stereotyping
- ☐ Other:

e. Please mark all of the challenges faced by immigrant and refugee populations when receiving healthcare that you have perceived or witnessed (may choose more than one):

- ☒ Language barriers
- ☒ Insurance barriers
- ☒ Cultural barriers
- ☒ Finding a professional interpreter
- ☒ Knowing how to work with a professional interpreter
- ☒ Time constraints
- ☒ Insufficiently trained health care providers
- ☒ Transportation problems for the patient
- ☐ Food insufficiency
- ☐ Need for child care
- ☒ Patients not understanding treatment plan
- ☒ Patients not following treatment plan
- ☒ My lack of knowledge regarding the patient's culture
- ☐ Bias or stereotyping
- ☒ Trust issues
- ☐ Other...

f. Rank how well immigrants and refugees understand the healthcare that you are trying to provide.

- ◯ Significantly less than a US born individual
- ◯ Less than a US born individual
- ⊗ Equivalent to a US born individual
- ◯ More than a US born patient
- ◯ Significantly more than a US born individual

g. Immigrants and refugees adhere to treatment plans and follow my recommendations.

- ◯ Never
- ◯ Rarely
- ◯ Sometimes
- ⊗ Usually
- ◯ Always

h. Immigrants and refugees should receive the same care and insurance coverage as US born patients.

- ◯ Never
- ◯ Rarely
- ◯ Sometimes
- ◯ Usually
- ⊗ Always

i. Immigrants and refugees who are undocumented should receive the same care and insurance coverage as US born patients.

- ◯ Never
- ◯ Rarely
- ◯ Sometimes
- ◯ Usually
- ⊗ Always

j. Every physician is professionally obligated to care for immigrants and refugees if they present to your clinic or hospital.

- ◯ Strongly disagree
- ◯ Disagree
- ◯ No opinion
- ◯ Agree
- ⊗ Strongly agree

k. Is healthcare a human right?

- ⊗ Yes
- ◯ No

B. If you wish, please tell us about what you enjoy or do not enjoy about immigrant and refugee health care and the greatest challenges you face in caring for this population.

____

SECTION D: DEMOGRAPHIC INFORMATION
Please answer the following questions by checking the box in front of the response choice that best describes you.

a. Your age?
- ◯ 20 to 24
- ⊗ 25 to 29
- ◯ 30 to 34
- ◯ 35 to 39
- ◯ 40 or older

b. Your gender?
- ◯ Female
- ⊗ Male
- ◯ Other

c. Are you Hispanic or Latino?
- ◯ Yes
- ⊗ No

d. What is your race? (Select one or more responses)
- ◯ American Indian or Alaska Native
- ◯ Asian (Please specify):
- ◯ Black or African American
- ◯ Native Hawaiian or Other Pacific Islander
- ⊗ White
- ◯ Other (Please specify):

e. Were you born in the United States?
- ⊗ Yes
- ◯ No

g. Your residency year?
- ◯ PGY1
- ◯ PGY2
- ⊗ PGY3
- ◯ PGY4
- ◯ PGY5

h. How would you classify your political ideology?
- ◯ Conservative
- ◯ Somewhat conservative
- ⊗ Moderate
- ◯ Somewhat liberal
- ◯ Liberal
- ◯ Other (Please specify):

i. Estimated level of educational debt?
- ◯ None
- ◯ Less than $50,000
- ◯ $50,000 - $100,000
- ⊗ $100,000 - $200,000
- ◯ $200,000 or more

j. Do you plan to subspecialize?
- ⊗ Yes
- ◯ No

k. Languages spoken?
- ☒ English
- ☒ Spanish
- ☐ French
- ☐ Hmong
- ☐ Somali
- ☐ Japanese
- ☐ Chinese
- ☐ Russian
- ☐ Ethiopian
- ☐ Other ___________

l. Are you in the Global Health Pathway?
- ◯ Yes
- ⊗ No

m. Did you earn your degree in the US?

- ⊗ Yes
- ◯ No

n. What residency program are you in?

- ⊗ Internal Medicine
- ◯ Med-Peds
- ◯ Pediatrics
- ◯ Family Practice
- ◯ Neurology
- ◯ Psychiatry
- ◯ ObGyn
- ◯ Neurosurgery
- ◯ General Surgery
- ◯ Orthopedic Surgery
- ◯ Urology
- ◯ Surgical sub-specialty (please specify in text box below)
- ◯ Non-clinical specialty (radiology, pathology; please specify in text box below)

Medical Trainees' attitudes, knowledge, and experience with immigrant and refugee health

Response was added on 11/01/2013 3:34pm.

SECTION A: Personal experience with immigrant and refugee health care.

A. Please indicate your level of agreement with the following statements regarding your personal experience with immigrant and refugee health care by checking the box that best represents your experience.

a. During my inpatient rotations, I take care of the following percentage of immigrant and refugee patients:

- ◯ None
- ◯ 0 -5%
- ⊗ 5-10%
- ◯ 10-25%
- ◯ > 25%

b. During my outpatient rotations, I take care of the following percentage of immigrant and refugee patients:

- ◯ None
- ⊗ 0-10%
- ◯ 10 -25%
- ◯ 25-50%
- ◯ 50-75%
- ◯ >75%

c. I would like to take care of more immigrant and refugee patients.

- ⊗ Strongly disagree
- ◯ Disagree
- ◯ No opinion
- ◯ Agree
- ◯ Strongly agree

d. I plan to take care of immigrants and refugees when I finish residency.

- ◯ Strongly disagree
- ◯ Disagree
- ⊗ No opinion
- ◯ Agree
- ◯ Strongly agree

e. I plan to do short term (< 6 months) international work when I finish residency.

- ◯ Strongly disagree
- ⊗ Disagree
- ◯ No opinion
- ◯ Agree
- ◯ Strongly agree

f. I plan to do long term (>6 months) international work when I finish residency.

- ⊗ Strongly disagree
- ◯ Disagree
- ◯ No opinion
- ◯ Agree
- ◯ Strongly agree

g. I plan to work in health disparities in the following way after residency:

na

SECTION B: MEDICAL EDUCATION

A. Please indicate your level of agreement with the following statements regarding your medical education and knowledge about immigrants and refugees by checking the box that best represents your opinion.

a. I have received specialized training in immigrant and refugee health, tropical medicine, or cross-cultural health.

- ⊗ Strongly disagree
- ◯ Disagree
- ◯ No opinion
- ◯ Agree
- ◯ Strongly agree

c. I feel comfortable with my fund of knowledge regarding immigrant and refugee health.

- ⊗ Strongly disagree
- ◯ Disagree
- ◯ No opinion
- ◯ Agree
- ◯ Strongly agree

d. I would like to have further training in immigrant and refugee health.

- ⊗ Strongly disagree
- ◯ Disagree
- ◯ No opinion
- ◯ Agree
- ◯ Strongly agree

SECTION C: Attitudes towards immigrant health

A.Please indicate your level of agreement with the following statements regarding immigrant and refugee health by checking the box that best represents your opinion.

a. I enjoy taking care of immigrants and refugees.

- ◯ Never
- ◯ Rarely
- ◯ Sometimes
- ⊗ Usually
- ◯ Always

b. Please indicate the reasons that you enjoy taking care of immigrants and refugees (may choose more than one).

- ☐ Tropical and other conditions not frequently diagnosed in US-born patients
- ☐ Learning about other cultures
- ☐ They don't complain as much
- ☐ Being able to hear their stories
- ☒ Their care is more complicated
- ☐ Their care is less complicated
- ☐ They are very appreciative of your help.
- ☐ They are extremely vulnerable
- ☐ Other:

c. Taking care of immigrants and refugees is more challenging than taking care of US born patients.

- ◯ Never
- ◯ Rarely
- ◯ Sometimes
- ⊗ Usually
- ◯ Always

d. Please mark all the challenges that you face as a provider when providing care to immigrants and refugees (may choose more than one):

- ☒ Language barriers
- ☒ Insurance barriers
- ☒ Cultural barriers
- ☐ Finding a professional interpreter
- ☐ Knowing how to work with a professional interpreter
- ☐ Time constraints
- ☒ My own knowledge related to tropical and travel medicine
- ☒ Transportation problems for the patient
- ☐ Patients not understanding treatment plan
- ☒ Patients not following treatment plan
- ☒ My lack of knowledge regarding the patient's culture
- ☐ Bias or stereotyping
- ☐ Other:

e. Please mark all of the challenges faced by immigrant and refugee populations when receiving healthcare that you have perceived or witnessed (may choose more than one):

- ☒ Language barriers
- ☒ Insurance barriers
- ☒ Cultural barriers
- ☒ Finding a professional interpreter
- ☒ Knowing how to work with a professional interpreter
- ☒ Time constraints
- ☒ Insufficiently trained health care providers
- ☒ Transportation problems for the patient
- ☒ Food insufficiency
- ☒ Need for child care
- ☒ Patients not understanding treatment plan
- ☒ Patients not following treatment plan
- ☒ My lack of knowledge regarding the patient's culture
- ☒ Bias or stereotyping
- ☒ Trust issues
- ☐ Other...

f. Rank how well immigrants and refugees understand the healthcare that you are trying to provide.

- ◯ Significantly less than a US born individual
- ⊗ Less than a US born individual
- ◯ Equivalent to a US born individual
- ◯ More than a US born patient
- ◯ Significantly more than a US born individual

g. Immigrants and refugees adhere to treatment plans and follow my recommendations.

- ◯ Never
- ◯ Rarely
- ◯ Sometimes
- ⊗ Usually
- ◯ Always

h. Immigrants and refugees should receive the same care and insurance coverage as US born patients.

- ◯ Never
- ⊗ Rarely
- ◯ Sometimes
- ◯ Usually
- ◯ Always

i. Immigrants and refugees who are undocumented should receive the same care and insurance coverage as US born patients.

- ◯ Never
- ⊗ Rarely
- ◯ Sometimes
- ◯ Usually
- ◯ Always

j. Every physician is professionally obligated to care for immigrants and refugees if they present to your clinic or hospital.

- ◯ Strongly disagree
- ⊗ Disagree
- ◯ No opinion
- ◯ Agree
- ◯ Strongly agree

k. Is healthcare a human right?

- ◯ Yes
- ⊗ No

B. If you wish, please tell us about what you enjoy or do not enjoy about immigrant and refugee health care and the greatest challenges you face in caring for this population.

____

SECTION D: DEMOGRAPHIC INFORMATION
Please answer the following questions by checking the box in front of the response choice that best describes you.

a. Your age?

◯ 20 to 24
◯ 25 to 29
⊗ 30 to 34
◯ 35 to 39
◯ 40 or older

b. Your gender?

◯ Female
⊗ Male
◯ Other

c. Are you Hispanic or Latino?

◯ Yes
⊗ No

d. What is your race? (Select one or more responses)

◯ American Indian or Alaska Native
◯ Asian (Please specify):
◯ Black or African American
◯ Native Hawaiian or Other Pacific Islander
⊗ White
◯ Other (Please specify):

e. Were you born in the United States?

⊗ Yes
◯ No

g. Your residency year?

◯ PGY1
◯ PGY2
◯ PGY3
⊗ PGY4
◯ PGY5

h. How would you classify your political ideology?

◯ Conservative
◯ Somewhat conservative
⊗ Moderate
◯ Somewhat liberal
◯ Liberal
◯ Other (Please specify):

i. Estimated level of educational debt?

◯ None
◯ Less than $50,000
◯ $50,000 - $100,000
◯ $100,000 - $200,000
⊗ $200,000 or more

j. Do you plan to subspecialize?

◯ Yes
⊗ No

k. Languages spoken?

☒ English
☐ Spanish
☐ French
☐ Hmong
☐ Somali
☐ Japanese
☐ Chinese
☐ Russian
☐ Ethiopian
☐ Other ___________

l. Are you in the Global Health Pathway?

◯ Yes
⊗ No

m. Did you earn your degree in the US?

⊗ Yes
◯ No

n. What residency program are you in?

◯ Internal Medicine
◯ Med-Peds
◯ Pediatrics
◯ Family Practice
◯ Neurology
◯ Psychiatry
◯ ObGyn
◯ Neurosurgery
◯ General Surgery
◯ Orthopedic Surgery
◯ Urology
◯ Surgical sub-specialty (please specify in text box below)
⊗ Non-clinical specialty (radiology, pathology; please specify in text box below)

bvf

Medical Trainees' attitudes, knowledge, and experience with immigrant and refugee health

Response was added on 11/01/2013 4:14pm.

SECTION A: Personal experience with immigrant and refugee health care.

A. Please indicate your level of agreement with the following statements regarding your personal experience with immigrant and refugee health care by checking the box that best represents your experience.

a. During my inpatient rotations, I take care of the following percentage of immigrant and refugee patients:

- ◯ None
- ◯ 0 -5%
- ⊗ 5-10%
- ◯ 10-25%
- ◯ > 25%

b. During my outpatient rotations, I take care of the following percentage of immigrant and refugee patients:

- ◯ None
- ◯ 0-10%
- ⊗ 10 -25%
- ◯ 25-50%
- ◯ 50-75%
- ◯ >75%

c. I would like to take care of more immigrant and refugee patients.

- ⊗ Strongly disagree
- ◯ Disagree
- ◯ No opinion
- ◯ Agree
- ◯ Strongly agree

d. I plan to take care of immigrants and refugees when I finish residency.

- ◯ Strongly disagree
- ◯ Disagree
- ⊗ No opinion
- ◯ Agree
- ◯ Strongly agree

e. I plan to do short term (< 6 months) international work when I finish residency.

- ◯ Strongly disagree
- ⊗ Disagree
- ◯ No opinion
- ◯ Agree
- ◯ Strongly agree

f. I plan to do long term (>6 months) international work when I finish residency.

- ◯ Strongly disagree
- ⊗ Disagree
- ◯ No opinion
- ◯ Agree
- ◯ Strongly agree

g. I plan to work in health disparities in the following way after residency:

Not

SECTION B: MEDICAL EDUCATION

A. Please indicate your level of agreement with the following statements regarding your medical education and knowledge about immigrants and refugees by checking the box that best represents your opinion.

a. I have received specialized training in immigrant and refugee health, tropical medicine, or cross-cultural health.

- ◯ Strongly disagree
- ◯ Disagree
- ◯ No opinion
- ⊗ Agree
- ◯ Strongly agree

b. If you have received specialized training in immigrant and refugee health, tropical medicine, or cross-cultural health, please indicate all the contexts in which you received this training:

- ☐ As an undergraduate.
- ☒ As a medical student.
- ☐ As part of my residency.
- ☐ A special program.
- ☐ As part of my fellowship.
- ☐ As part of a degree program (e.g. MPH)
- ☐ Other:

c. I feel comfortable with my fund of knowledge regarding immigrant and refugee health.

- ◯ Strongly disagree
- ◯ Disagree
- ◯ No opinion
- ⊗ Agree
- ◯ Strongly agree

d. I would like to have further training in immigrant and refugee health.

- ◯ Strongly disagree
- ⊗ Disagree
- ◯ No opinion
- ◯ Agree
- ◯ Strongly agree

SECTION C: Attitudes towards immigrant health

A.Please indicate your level of agreement with the following statements regarding immigrant and refugee health by checking the box that best represents your opinion.

a. I enjoy taking care of immigrants and refugees.

- ◯ Never
- ◯ Rarely
- ⊗ Sometimes
- ◯ Usually
- ◯ Always

b. Please indicate the reasons that you enjoy taking care of immigrants and refugees (may choose more than one).

- ☐ Tropical and other conditions not frequently diagnosed in US-born patients
- ☐ Learning about other cultures
- ☐ They don't complain as much
- ☒ Being able to hear their stories
- ☐ Their care is more complicated
- ☐ Their care is less complicated
- ☐ They are very appreciative of your help.
- ☐ They are extremely vulnerable
- ☐ Other:

c. Taking care of immigrants and refugees is more challenging than taking care of US born patients.

- ◯ Never
- ◯ Rarely
- ◯ Sometimes
- ⊗ Usually
- ◯ Always

d. Please mark all the challenges that you face as a provider when providing care to immigrants and refugees (may choose more than one):

- ☒ Language barriers
- ☒ Insurance barriers
- ☒ Cultural barriers
- ☒ Finding a professional interpreter
- ☒ Knowing how to work with a professional interpreter
- ☒ Time constraints
- ☒ My own knowledge related to tropical and travel medicine
- ☒ Transportation problems for the patient
- ☒ Patients not understanding treatment plan
- ☒ Patients not following treatment plan
- ☐ My lack of knowledge regarding the patient's culture
- ☐ Bias or stereotyping
- ☐ Other:

e. Please mark all of the challenges faced by immigrant and refugee populations when receiving healthcare that you have perceived or witnessed (may choose more than one):

- ☒ Language barriers
- ☒ Insurance barriers
- ☒ Cultural barriers
- ☒ Finding a professional interpreter
- ☒ Knowing how to work with a professional interpreter
- ☒ Time constraints
- ☒ Insufficiently trained health care providers
- ☒ Transportation problems for the patient
- ☒ Food insufficiency
- ☒ Need for child care
- ☒ Patients not understanding treatment plan
- ☒ Patients not following treatment plan
- ☒ My lack of knowledge regarding the patient's culture
- ☒ Bias or stereotyping
- ☒ Trust issues
- ☐ Other...

f. Rank how well immigrants and refugees understand the healthcare that you are trying to provide.

- ◯ Significantly less than a US born individual
- ⊗ Less than a US born individual
- ◯ Equivalent to a US born individual
- ◯ More than a US born patient
- ◯ Significantly more than a US born individual

g. Immigrants and refugees adhere to treatment plans and follow my recommendations.

- ◯ Never
- ◯ Rarely
- ⊗ Sometimes
- ◯ Usually
- ◯ Always

h. Immigrants and refugees should receive the same care and insurance coverage as US born patients.

- ◯ Never
- ◯ Rarely
- ⊗ Sometimes
- ◯ Usually
- ◯ Always

i. Immigrants and refugees who are undocumented should receive the same care and insurance coverage as US born patients.

- ◯ Never
- ◯ Rarely
- ⊗ Sometimes
- ◯ Usually
- ◯ Always

j. Every physician is professionally obligated to care for immigrants and refugees if they present to your clinic or hospital.

- ◯ Strongly disagree
- ◯ Disagree
- ◯ No opinion
- ◯ Agree
- ⊗ Strongly agree

k. Is healthcare a human right?
- ◯ Yes
- ⊗ No

B. If you wish, please tell us about what you enjoy or do not enjoy about immigrant and refugee health care and the greatest challenges you face in caring for this population.

____

SECTION D: DEMOGRAPHIC INFORMATION
Please answer the following questions by checking the box in front of the response choice that best describes you.

a. Your age?
- ◯ 20 to 24
- ⊗ 25 to 29
- ◯ 30 to 34
- ◯ 35 to 39
- ◯ 40 or older

b. Your gender?
- ◯ Female
- ⊗ Male
- ◯ Other

c. Are you Hispanic or Latino?
- ◯ Yes
- ⊗ No

d. What is your race? (Select one or more responses)
- ◯ American Indian or Alaska Native
- ◯ Asian (Please specify):
- ◯ Black or African American
- ◯ Native Hawaiian or Other Pacific Islander
- ⊗ White
- ◯ Other (Please specify):

e. Were you born in the United States?
- ⊗ Yes
- ◯ No

g. Your residency year?
- ⊗ PGY1
- ◯ PGY2
- ◯ PGY3
- ◯ PGY4
- ◯ PGY5

h. How would you classify your political ideology?
- ◯ Conservative
- ◯ Somewhat conservative
- ⊗ Moderate
- ◯ Somewhat liberal
- ◯ Liberal
- ◯ Other (Please specify):

i. Estimated level of educational debt?
- ◯ None
- ◯ Less than $50,000
- ◯ $50,000 - $100,000
- ⊗ $100,000 - $200,000
- ◯ $200,000 or more

j. Do you plan to subspecialize?
- ⊗ Yes
- ◯ No

k. Languages spoken?

- ☒ English
- ☐ Spanish
- ☐ French
- ☐ Hmong
- ☐ Somali
- ☐ Japanese
- ☐ Chinese
- ☐ Russian
- ☐ Ethiopian
- ☐ Other ___________

l. Are you in the Global Health Pathway?

- ◯ Yes
- ⊗ No

m. Did you earn your degree in the US?

- ⊗ Yes
- ◯ No

n. What residency program are you in?

- ◯ Internal Medicine
- ◯ Med-Peds
- ◯ Pediatrics
- ◯ Family Practice
- ◯ Neurology
- ◯ Psychiatry
- ◯ ObGyn
- ◯ Neurosurgery
- ◯ General Surgery
- ◯ Orthopedic Surgery
- ◯ Urology
- ◯ Surgical sub-specialty (please specify in text box below)
- ⊗ Non-clinical specialty (radiology, pathology; please specify in text box below)

Radiology

Medical Trainees' attitudes, knowledge, and experience with immigrant and refugee health

Response was added on 11/01/2013 4:21pm.

SECTION A: Personal experience with immigrant and refugee health care.

A. Please indicate your level of agreement with the following statements regarding your personal experience with immigrant and refugee health care by checking the box that best represents your experience.

a. During my inpatient rotations, I take care of the following percentage of immigrant and refugee patients:

- ◯ None
- ◯ 0 -5%
- ◯ 5-10%
- ⊗ 10-25%
- ◯ > 25%

b. During my outpatient rotations, I take care of the following percentage of immigrant and refugee patients:

- ◯ None
- ◯ 0-10%
- ◯ 10 -25%
- ⊗ 25-50%
- ◯ 50-75%
- ◯ >75%

c. I would like to take care of more immigrant and refugee patients.

- ◯ Strongly disagree
- ⊗ Disagree
- ◯ No opinion
- ◯ Agree
- ◯ Strongly agree

d. I plan to take care of immigrants and refugees when I finish residency.

- ◯ Strongly disagree
- ◯ Disagree
- ◯ No opinion
- ⊗ Agree
- ◯ Strongly agree

e. I plan to do short term (< 6 months) international work when I finish residency.

- ◯ Strongly disagree
- ◯ Disagree
- ◯ No opinion
- ⊗ Agree
- ◯ Strongly agree

f. I plan to do long term (>6 months) international work when I finish residency.

- ◯ Strongly disagree
- ⊗ Disagree
- ◯ No opinion
- ◯ Agree
- ◯ Strongly agree

g. I plan to work in health disparities in the following way after residency:

donate money to organizations that participate in humanitarian causes.

SECTION B: MEDICAL EDUCATION

A. Please indicate your level of agreement with the following statements regarding your medical education and knowledge about immigrants and refugees by checking the box that best represents your opinion.

a. I have received specialized training in immigrant and refugee health, tropical medicine, or cross-cultural health.

- ◯ Strongly disagree
- ◯ Disagree
- ◯ No opinion
- ⊗ Agree
- ◯ Strongly agree

b. If you have received specialized training in immigrant and refugee health, tropical medicine, or cross-cultural health, please indicate all the contexts in which you received this training:

- ☒ As an undergraduate.
- ☒ As a medical student.
- ☒ As part of my residency.
- ☐ A special program.
- ☐ As part of my fellowship.
- ☐ As part of a degree program (e.g. MPH)
- ☐ Other:

c. I feel comfortable with my fund of knowledge regarding immigrant and refugee health.

- ◯ Strongly disagree
- ⊗ Disagree
- ◯ No opinion
- ◯ Agree
- ◯ Strongly agree

d. I would like to have further training in immigrant and refugee health.

- ◯ Strongly disagree
- ◯ Disagree
- ◯ No opinion
- ⊗ Agree
- ◯ Strongly agree

e. If you agree with the above, please indicate all the contexts in which you would like to receive this training:
□□

- ☒ As part of my residency.
- ☐ A special program.
- ☐ As part of my fellowship.

SECTION C: Attitudes towards immigrant health

A.□Please indicate your level of agreement with the following statements regarding immigrant and refugee health by checking the box that best represents your opinion.

a. I enjoy taking care of immigrants and refugees.

- ◯ Never
- ◯ Rarely
- ⊗ Sometimes
- ◯ Usually
- ◯ Always

b. Please indicate the reasons that you enjoy taking care of immigrants and refugees (may choose more than one).

- ☐ Tropical and other conditions not frequently diagnosed in US-born patients
- ☒ Learning about other cultures
- ☐ They don't complain as much
- ☒ Being able to hear their stories
- ☐ Their care is more complicated
- ☐ Their care is less complicated
- ☐ They are very appreciative of your help.
- ☐ They are extremely vulnerable
- ☐ Other:

c. Taking care of immigrants and refugees is more challenging than taking care of US born patients.

- ◯ Never
- ◯ Rarely
- ◯ Sometimes
- ◯ Usually
- ⊗ Always

d. Please mark all the challenges that you face as a provider when providing care to immigrants and refugees (may choose more than one):

☒ Language barriers
☒ Insurance barriers
☒ Cultural barriers
☒ Finding a professional interpreter
☐ Knowing how to work with a professional interpreter
☒ Time constraints
☐ My own knowledge related to tropical and travel medicine
☐ Transportation problems for the patient
☒ Patients not understanding treatment plan
☒ Patients not following treatment plan
☒ My lack of knowledge regarding the patient's culture
☐ Bias or stereotyping
☐ Other:

e. Please mark all of the challenges faced by immigrant and refugee populations when receiving healthcare that you have perceived or witnessed (may choose more than one):

☒ Language barriers
☐ Insurance barriers
☐ Cultural barriers
☐ Finding a professional interpreter
☐ Knowing how to work with a professional interpreter
☐ Time constraints
☐ Insufficiently trained health care providers
☐ Transportation problems for the patient
☐ Food insufficiency
☐ Need for child care
☒ Patients not understanding treatment plan
☒ Patients not following treatment plan
☒ My lack of knowledge regarding the patient's culture
☒ Bias or stereotyping
☒ Trust issues
☐ Other...

f. Rank how well immigrants and refugees understand the healthcare that you are trying to provide.

⊗ Significantly less than a US born individual
◯ Less than a US born individual
◯ Equivalent to a US born individual
◯ More than a US born patient
◯ Significantly more than a US born individual

g. Immigrants and refugees adhere to treatment plans and follow my recommendations.

◯ Never
◯ Rarely
⊗ Sometimes
◯ Usually
◯ Always

h. Immigrants and refugees should receive the same care and insurance coverage as US born patients.

◯ Never
⊗ Rarely
◯ Sometimes
◯ Usually
◯ Always

i. Immigrants and refugees who are undocumented should receive the same care and insurance coverage as US born patients.

⊗ Never
◯ Rarely
◯ Sometimes
◯ Usually
◯ Always

j. Every physician is professionally obligated to care for immigrants and refugees if they present to your clinic or hospital.

⊗ Strongly disagree
◯ Disagree
◯ No opinion
◯ Agree
◯ Strongly agree

k. Is healthcare a human right?
◯ Yes
⊗ No

B. If you wish, please tell us about what you enjoy or do not enjoy about immigrant and refugee health care and the greatest challenges you face in caring for this population.
____

SECTION D: DEMOGRAPHIC INFORMATION
Please answer the following questions by checking the box in front of the response choice that best describes you.

a. Your age?
◯ 20 to 24
⊗ 25 to 29
◯ 30 to 34
◯ 35 to 39
◯ 40 or older

b. Your gender?
⊗ Female
◯ Male
◯ Other

c.Are you Hispanic or Latino?
◯ Yes
⊗ No

d. What is your race? (Select one or more responses)
◯ American Indian or Alaska Native
◯ Asian (Please specify):
◯ Black or African American
◯ Native Hawaiian or Other Pacific Islander
◯ White
◯ Other (Please specify):

e.Were you born in the United States?
⊗ Yes
◯ No

g. Your residency year?
◯ PGY1
⊗ PGY2
◯ PGY3
◯ PGY4
◯ PGY5

h. How would you classify your political ideology?
◯ Conservative
◯ Somewhat conservative
◯ Moderate
◯ Somewhat liberal
◯ Liberal
◯ Other (Please specify):

i. Estimated level of educational debt?
◯ None
◯ Less than $50,000
◯ $50,000 - $100,000
⊗ $100,000 - $200,000
◯ $200,000 or more

j.Do you plan to subspecialize?
⊗ Yes
◯ No

k. Languages spoken?

- [x] English
- [ ] Spanish
- [ ] French
- [ ] Hmong
- [ ] Somali
- [ ] Japanese
- [ ] Chinese
- [ ] Russian
- [ ] Ethiopian
- [ ] Other ___________

l. Are you in the Global Health Pathway?

- ○ Yes
- ⊗ No

m. Did you earn your degree in the US?

- ⊗ Yes
- ○ No

n. What residency program are you in?

- ○ Internal Medicine
- ○ Med-Peds
- ○ Pediatrics
- ○ Family Practice
- ○ Neurology
- ○ Psychiatry
- ○ ObGyn
- ○ Neurosurgery
- ○ General Surgery
- ○ Orthopedic Surgery
- ○ Urology
- ○ Surgical sub-specialty (please specify in text box below)
- ○ Non-clinical specialty (radiology, pathology; please specify in text box below)

Medical Trainees' attitudes, knowledge, and experience with immigrant and refugee health

Response was added on 11/01/2013 5:49pm.

SECTION A: Personal experience with immigrant and refugee health care.

A. Please indicate your level of agreement with the following statements regarding your personal experience with immigrant and refugee health care by checking the box that best represents your experience.

a. During my inpatient rotations, I take care of the following percentage of immigrant and refugee patients:	◯ None<br>◯ 0 -5%<br>⊗ 5-10%<br>◯ 10-25%<br>◯ > 25%
b. During my outpatient rotations, I take care of the following percentage of immigrant and refugee patients:	◯ None<br>◯ 0-10%<br>◯ 10 -25%<br>⊗ 25-50%<br>◯ 50-75%<br>◯ >75%
c. I would like to take care of more immigrant and refugee patients.	◯ Strongly disagree<br>◯ Disagree<br>⊗ No opinion<br>◯ Agree<br>◯ Strongly agree
d. I plan to take care of immigrants and refugees when I finish residency.	◯ Strongly disagree<br>◯ Disagree<br>⊗ No opinion<br>◯ Agree<br>◯ Strongly agree
e. I plan to do short term (< 6 months) international work when I finish residency.	◯ Strongly disagree<br>⊗ Disagree<br>◯ No opinion<br>◯ Agree<br>◯ Strongly agree
f. I plan to do long term (>6 months) international work when I finish residency.	◯ Strongly disagree<br>⊗ Disagree<br>◯ No opinion<br>◯ Agree<br>◯ Strongly agree
g. I plan to work in health disparities in the following way after residency:	Unsure

SECTION B: MEDICAL EDUCATION

A. Please indicate your level of agreement with the following statements regarding your medical education and knowledge about immigrants and refugees by checking the box that best represents your opinion.

a. I have received specialized training in immigrant and refugee health, tropical medicine, or cross-cultural health.

- ◯ Strongly disagree
- ◯ Disagree
- ◯ No opinion
- ⊗ Agree
- ◯ Strongly agree

b. If you have received specialized training in immigrant and refugee health, tropical medicine, or cross-cultural health, please indicate all the contexts in which you received this training:

- ☐ As an undergraduate.
- ☒ As a medical student.
- ☐ As part of my residency.
- ☐ A special program.
- ☐ As part of my fellowship.
- ☐ As part of a degree program (e.g. MPH)
- ☐ Other:

c. I feel comfortable with my fund of knowledge regarding immigrant and refugee health.

- ◯ Strongly disagree
- ◯ Disagree
- ⊗ No opinion
- ◯ Agree
- ◯ Strongly agree

d. I would like to have further training in immigrant and refugee health.

- ◯ Strongly disagree
- ◯ Disagree
- ◯ No opinion
- ⊗ Agree
- ◯ Strongly agree

e. If you agree with the above, please indicate all the contexts in which you would like to receive this training:

- ☒ As part of my residency.
- ☒ A special program.
- ☐ As part of my fellowship.

SECTION C: Attitudes towards immigrant health

A. Please indicate your level of agreement with the following statements regarding immigrant and refugee health by checking the box that best represents your opinion.

a. I enjoy taking care of immigrants and refugees.

- ◯ Never
- ◯ Rarely
- ⊗ Sometimes
- ◯ Usually
- ◯ Always

b. Please indicate the reasons that you enjoy taking care of immigrants and refugees (may choose more than one).

- ☐ Tropical and other conditions not frequently diagnosed in US-born patients
- ☒ Learning about other cultures
- ☐ They don't complain as much
- ☐ Being able to hear their stories
- ☐ Their care is more complicated
- ☐ Their care is less complicated
- ☐ They are very appreciative of your help.
- ☒ They are extremely vulnerable
- ☐ Other:

c. Taking care of immigrants and refugees is more challenging than taking care of US born patients.

- ◯ Never
- ◯ Rarely
- ◯ Sometimes
- ⊗ Usually
- ◯ Always

d. Please mark all the challenges that you face as a provider when providing care to immigrants and refugees (may choose more than one):

- [x] Language barriers
- [ ] Insurance barriers
- [x] Cultural barriers
- [x] Finding a professional interpreter
- [ ] Knowing how to work with a professional interpreter
- [ ] Time constraints
- [x] My own knowledge related to tropical and travel medicine
- [x] Transportation problems for the patient
- [ ] Patients not understanding treatment plan
- [x] Patients not following treatment plan
- [ ] My lack of knowledge regarding the patient's culture
- [ ] Bias or stereotyping
- [ ] Other:

e. Please mark all of the challenges faced by immigrant and refugee populations when receiving healthcare that you have perceived or witnessed (may choose more than one):

- [x] Language barriers
- [ ] Insurance barriers
- [x] Cultural barriers
- [x] Finding a professional interpreter
- [ ] Knowing how to work with a professional interpreter
- [ ] Time constraints
- [x] Insufficiently trained health care providers
- [x] Transportation problems for the patient
- [ ] Food insufficiency
- [ ] Need for child care
- [ ] Patients not understanding treatment plan
- [x] Patients not following treatment plan
- [ ] My lack of knowledge regarding the patient's culture
- [ ] Bias or stereotyping
- [ ] Trust issues
- [ ] Other...

f. Rank how well immigrants and refugees understand the healthcare that you are trying to provide.

- ( ) Significantly less than a US born individual
- ( ) Less than a US born individual
- (x) Equivalent to a US born individual
- ( ) More than a US born patient
- ( ) Significantly more than a US born individual

g. Immigrants and refugees adhere to treatment plans and follow my recommendations.

- ( ) Never
- ( ) Rarely
- (x) Sometimes
- ( ) Usually
- ( ) Always

h. Immigrants and refugees should receive the same care and insurance coverage as US born patients.

- ( ) Never
- ( ) Rarely
- ( ) Sometimes
- ( ) Usually
- (x) Always

i. Immigrants and refugees who are undocumented should receive the same care and insurance coverage as US born patients.

- ( ) Never
- ( ) Rarely
- ( ) Sometimes
- ( ) Usually
- (x) Always

j. Every physician is professionally obligated to care for immigrants and refugees if they present to your clinic or hospital.

- ( ) Strongly disagree
- ( ) Disagree
- ( ) No opinion
- ( ) Agree
- (x) Strongly agree

k. Is healthcare a human right?

- ⊗ Yes
- ◯ No

B. If you wish, please tell us about what you enjoy or do not enjoy about immigrant and refugee health care and the greatest challenges you face in caring for this population.

____

SECTION D: DEMOGRAPHIC INFORMATION
Please answer the following questions by checking the box in front of the response choice that best describes you.

a. Your age?

- ◯ 20 to 24
- ⊗ 25 to 29
- ◯ 30 to 34
- ◯ 35 to 39
- ◯ 40 or older

b. Your gender?

- ⊗ Female
- ◯ Male
- ◯ Other

c.Are you Hispanic or Latino?

- ◯ Yes
- ⊗ No

d. What is your race? (Select one or more responses)

- ◯ American Indian or Alaska Native
- ◯ Asian (Please specify):
- ◯ Black or African American
- ◯ Native Hawaiian or Other Pacific Islander
- ⊗ White
- ◯ Other (Please specify):

e.Were you born in the United States?

- ⊗ Yes
- ◯ No

g. Your residency year?

- ⊗ PGY1
- ◯ PGY2
- ◯ PGY3
- ◯ PGY4
- ◯ PGY5

h. How would you classify your political ideology?

- ◯ Conservative
- ◯ Somewhat conservative
- ◯ Moderate
- ⊗ Somewhat liberal
- ◯ Liberal
- ◯ Other (Please specify):

i. Estimated level of educational debt?

- ◯ None
- ◯ Less than $50,000
- ◯ $50,000 - $100,000
- ◯ $100,000 - $200,000
- ⊗ $200,000 or more

j.Do you plan to subspecialize?

- ◯ Yes
- ⊗ No

k. Languages spoken?

- ☒ English
- ☐ Spanish
- ☐ French
- ☐ Hmong
- ☐ Somali
- ☐ Japanese
- ☐ Chinese
- ☐ Russian
- ☐ Ethiopian
- ☐ Other ___________

l. Are you in the Global Health Pathway?

- ◯ Yes
- ⊗ No

m. Did you earn your degree in the US?

- ⊗ Yes
- ◯ No

n. What residency program are you in?

- ◯ Internal Medicine
- ◯ Med-Peds
- ⊗ Pediatrics
- ◯ Family Practice
- ◯ Neurology
- ◯ Psychiatry
- ◯ ObGyn
- ◯ Neurosurgery
- ◯ General Surgery
- ◯ Orthopedic Surgery
- ◯ Urology
- ◯ Surgical sub-specialty (please specify in text box below)
- ◯ Non-clinical specialty (radiology, pathology; please specify in text box below)

Medical Trainees' attitudes, knowledge, and experience with immigrant and refugee health

Response was added on 11/01/2013 5:53pm.

SECTION A: Personal experience with immigrant and refugee health care.

A. Please indicate your level of agreement with the following statements regarding your personal experience with immigrant and refugee health care by checking the box that best represents your experience.

a. During my inpatient rotations, I take care of the following percentage of immigrant and refugee patients:

- ◯ None
- ◯ 0 -5%
- ◯ 5-10%
- ◯ 10-25%
- ⊗ > 25%

b. During my outpatient rotations, I take care of the following percentage of immigrant and refugee patients:

- ◯ None
- ◯ 0-10%
- ◯ 10 -25%
- ◯ 25-50%
- ◯ 50-75%
- ⊗ >75%

c. I would like to take care of more immigrant and refugee patients.

- ◯ Strongly disagree
- ◯ Disagree
- ◯ No opinion
- ⊗ Agree
- ◯ Strongly agree

d. I plan to take care of immigrants and refugees when I finish residency.

- ◯ Strongly disagree
- ◯ Disagree
- ◯ No opinion
- ◯ Agree
- ⊗ Strongly agree

e. I plan to do short term (< 6 months) international work when I finish residency.

- ◯ Strongly disagree
- ◯ Disagree
- ◯ No opinion
- ◯ Agree
- ⊗ Strongly agree

f. I plan to do long term (>6 months) international work when I finish residency.

- ◯ Strongly disagree
- ◯ Disagree
- ◯ No opinion
- ◯ Agree
- ⊗ Strongly agree

g. I plan to work in health disparities in the following way after residency:

serve under-served population, hopefully an urban group with a good number of immigrants

SECTION B: MEDICAL EDUCATION

A. Please indicate your level of agreement with the following statements regarding your medical education and knowledge about immigrants and refugees by checking the box that best represents your opinion.

a. I have received specialized training in immigrant and refugee health, tropical medicine, or cross-cultural health.

- ◯ Strongly disagree
- ◯ Disagree
- ◯ No opinion
- ◯ Agree
- ⊗ Strongly agree

b. If you have received specialized training in immigrant and refugee health, tropical medicine, or cross-cultural health, please indicate all the contexts in which you received this training:

- ☐ As an undergraduate.
- ☒ As a medical student.
- ☒ As part of my residency.
- ☒ A special program.
- ☐ As part of my fellowship.
- ☒ As part of a degree program (e.g. MPH)
- ☐ Other:

c. I feel comfortable with my fund of knowledge regarding immigrant and refugee health.

- ◯ Strongly disagree
- ◯ Disagree
- ◯ No opinion
- ◯ Agree
- ⊗ Strongly agree

d. I would like to have further training in immigrant and refugee health.

- ◯ Strongly disagree
- ◯ Disagree
- ◯ No opinion
- ⊗ Agree
- ◯ Strongly agree

e. If you agree with the above, please indicate all the contexts in which you would like to receive this training:
□□

- ☒ As part of my residency.
- ☐ A special program.
- ☐ As part of my fellowship.

SECTION C: Attitudes towards immigrant health

A.□Please indicate your level of agreement with the following statements regarding immigrant and refugee health by checking the box that best represents your opinion.

a. I enjoy taking care of immigrants and refugees.

- ◯ Never
- ◯ Rarely
- ◯ Sometimes
- ◯ Usually
- ⊗ Always

b. Please indicate the reasons that you enjoy taking care of immigrants and refugees (may choose more than one).

- ☒ Tropical and other conditions not frequently diagnosed in US-born patients
- ☒ Learning about other cultures
- ☐ They don't complain as much
- ☒ Being able to hear their stories
- ☒ Their care is more complicated
- ☐ Their care is less complicated
- ☒ They are very appreciative of your help.
- ☒ They are extremely vulnerable
- ☐ Other:

c. Taking care of immigrants and refugees is more challenging than taking care of US born patients.

- ◯ Never
- ◯ Rarely
- ⊗ Sometimes
- ◯ Usually
- ◯ Always

d. Please mark all the challenges that you face as a provider when providing care to immigrants and refugees (may choose more than one):

- ☒ Language barriers
- ☒ Insurance barriers
- ☒ Cultural barriers
- ☐ Finding a professional interpreter
- ☐ Knowing how to work with a professional interpreter
- ☒ Time constraints
- ☐ My own knowledge related to tropical and travel medicine
- ☒ Transportation problems for the patient
- ☐ Patients not understanding treatment plan
- ☐ Patients not following treatment plan
- ☒ My lack of knowledge regarding the patient's culture
- ☐ Bias or stereotyping
- ☐ Other:

e. Please mark all of the challenges faced by immigrant and refugee populations when receiving healthcare that you have perceived or witnessed (may choose more than one):

- ☒ Language barriers
- ☒ Insurance barriers
- ☒ Cultural barriers
- ☒ Finding a professional interpreter
- ☒ Knowing how to work with a professional interpreter
- ☐ Time constraints
- ☐ Insufficiently trained health care providers
- ☐ Transportation problems for the patient
- ☐ Food insufficiency
- ☐ Need for child care
- ☐ Patients not understanding treatment plan
- ☐ Patients not following treatment plan
- ☐ My lack of knowledge regarding the patient's culture
- ☐ Bias or stereotyping
- ☒ Trust issues
- ☐ Other...

f. Rank how well immigrants and refugees understand the healthcare that you are trying to provide.

- ◯ Significantly less than a US born individual
- ⊗ Less than a US born individual
- ◯ Equivalent to a US born individual
- ◯ More than a US born patient
- ◯ Significantly more than a US born individual

g. Immigrants and refugees adhere to treatment plans and follow my recommendations.

- ◯ Never
- ◯ Rarely
- ⊗ Sometimes
- ◯ Usually
- ◯ Always

h. Immigrants and refugees should receive the same care and insurance coverage as US born patients.

- ◯ Never
- ◯ Rarely
- ◯ Sometimes
- ◯ Usually
- ⊗ Always

i. Immigrants and refugees who are undocumented should receive the same care and insurance coverage as US born patients.

- ◯ Never
- ◯ Rarely
- ⊗ Sometimes
- ◯ Usually
- ◯ Always

j. Every physician is professionally obligated to care for immigrants and refugees if they present to your clinic or hospital.

- ◯ Strongly disagree
- ◯ Disagree
- ◯ No opinion
- ◯ Agree
- ⊗ Strongly agree

k. Is healthcare a human right?

⊗ Yes
◯ No

B. If you wish, please tell us about what you enjoy or do not enjoy about immigrant and refugee health care and the greatest challenges you face in caring for this population.

____

SECTION D: DEMOGRAPHIC INFORMATION
Please answer the following questions by checking the box in front of the response choice that best describes you.

a. Your age?

◯ 20 to 24
◯ 25 to 29
⊗ 30 to 34
◯ 35 to 39
◯ 40 or older

b. Your gender?

◯ Female
⊗ Male
◯ Other

c. Are you Hispanic or Latino?

◯ Yes
⊗ No

d. What is your race? (Select one or more responses)

◯ American Indian or Alaska Native
◯ Asian (Please specify):
◯ Black or African American
◯ Native Hawaiian or Other Pacific Islander
⊗ White
◯ Other (Please specify):

e. Were you born in the United States?

⊗ Yes
◯ No

g. Your residency year?

◯ PGY1
⊗ PGY2
◯ PGY3
◯ PGY4
◯ PGY5

h. How would you classify your political ideology?

◯ Conservative
◯ Somewhat conservative
◯ Moderate
◯ Somewhat liberal
⊗ Liberal
◯ Other (Please specify):

i. Estimated level of educational debt?

◯ None
◯ Less than $50,000
◯ $50,000 - $100,000
◯ $100,000 - $200,000
⊗ $200,000 or more

j. Do you plan to subspecialize?

◯ Yes
⊗ No

k. Languages spoken?

- ☒ English
- ☒ Spanish
- ☒ French
- ☐ Hmong
- ☐ Somali
- ☐ Japanese
- ☐ Chinese
- ☐ Russian
- ☐ Ethiopian
- ☐ Other ___________

l. Are you in the Global Health Pathway?

- ⊗ Yes
- ◯ No

m. Did you earn your degree in the US?

- ⊗ Yes
- ◯ No

n. What residency program are you in?

- ⊗ Internal Medicine
- ◯ Med-Peds
- ◯ Pediatrics
- ◯ Family Practice
- ◯ Neurology
- ◯ Psychiatry
- ◯ ObGyn
- ◯ Neurosurgery
- ◯ General Surgery
- ◯ Orthopedic Surgery
- ◯ Urology
- ◯ Surgical sub-specialty (please specify in text box below)
- ◯ Non-clinical specialty (radiology, pathology; please specify in text box below)

Medical Trainees' attitudes, knowledge, and experience with immigrant and refugee health

Response was added on 11/01/2013 6:54pm.

SECTION A: Personal experience with immigrant and refugee health care.

A. Please indicate your level of agreement with the following statements regarding your personal experience with immigrant and refugee health care by checking the box that best represents your experience.

a. During my inpatient rotations, I take care of the following percentage of immigrant and refugee patients:	◯ None<br>⊗ 0 -5%<br>◯ 5-10%<br>◯ 10-25%<br>◯ > 25%
b. During my outpatient rotations, I take care of the following percentage of immigrant and refugee patients:	⊗ None<br>◯ 0-10%<br>◯ 10 -25%<br>◯ 25-50%<br>◯ 50-75%<br>◯ >75%
c. I would like to take care of more immigrant and refugee patients.	◯ Strongly disagree<br>◯ Disagree<br>◯ No opinion<br>⊗ Agree<br>◯ Strongly agree
d. I plan to take care of immigrants and refugees when I finish residency.	◯ Strongly disagree<br>◯ Disagree<br>◯ No opinion<br>⊗ Agree<br>◯ Strongly agree
e. I plan to do short term (< 6 months) international work when I finish residency.	◯ Strongly disagree<br>◯ Disagree<br>⊗ No opinion<br>◯ Agree<br>◯ Strongly agree
f. I plan to do long term (>6 months) international work when I finish residency.	◯ Strongly disagree<br>⊗ Disagree<br>◯ No opinion<br>◯ Agree<br>◯ Strongly agree
g. I plan to work in health disparities in the following way after residency:	Caring for under served

SECTION B: MEDICAL EDUCATION

A. Please indicate your level of agreement with the following statements regarding your medical education and knowledge about immigrants and refugees by checking the box that best represents your opinion.

a. I have received specialized training in immigrant and refugee health, tropical medicine, or cross-cultural health.

- ◯ Strongly disagree
- ⊗ Disagree
- ◯ No opinion
- ◯ Agree
- ◯ Strongly agree

c. I feel comfortable with my fund of knowledge regarding immigrant and refugee health.

- ◯ Strongly disagree
- ⊗ Disagree
- ◯ No opinion
- ◯ Agree
- ◯ Strongly agree

d. I would like to have further training in immigrant and refugee health.

- ◯ Strongly disagree
- ◯ Disagree
- ◯ No opinion
- ⊗ Agree
- ◯ Strongly agree

e. If you agree with the above, please indicate all the contexts in which you would like to receive this training:
□□

- ☒ As part of my residency.
- ☒ A special program.
- ☒ As part of my fellowship.

SECTION C: Attitudes towards immigrant health

A.□Please indicate your level of agreement with the following statements regarding immigrant and refugee health by checking the box that best represents your opinion.

a. I enjoy taking care of immigrants and refugees.

- ◯ Never
- ◯ Rarely
- ◯ Sometimes
- ⊗ Usually
- ◯ Always

b. Please indicate the reasons that you enjoy taking care of immigrants and refugees (may choose more than one).

- ☐ Tropical and other conditions not frequently diagnosed in US-born patients
- ☒ Learning about other cultures
- ☐ They don't complain as much
- ☒ Being able to hear their stories
- ☐ Their care is more complicated
- ☐ Their care is less complicated
- ☒ They are very appreciative of your help.
- ☐ They are extremely vulnerable
- ☐ Other:

c. Taking care of immigrants and refugees is more challenging than taking care of US born patients.

- ◯ Never
- ◯ Rarely
- ⊗ Sometimes
- ◯ Usually
- ◯ Always

d. Please mark all the challenges that you face as a provider when providing care to immigrants and refugees (may choose more than one):

- ☒ Language barriers
- ☒ Insurance barriers
- ☒ Cultural barriers
- ☒ Finding a professional interpreter
- ☒ Knowing how to work with a professional interpreter
- ☒ Time constraints
- ☒ My own knowledge related to tropical and travel medicine
- ☐ Transportation problems for the patient
- ☒ Patients not understanding treatment plan
- ☒ Patients not following treatment plan
- ☒ My lack of knowledge regarding the patient's culture
- ☐ Bias or stereotyping
- ☐ Other:

e. Please mark all of the challenges faced by immigrant and refugee populations when receiving healthcare that you have perceived or witnessed (may choose more than one):

- ☒ Language barriers
- ☒ Insurance barriers
- ☒ Cultural barriers
- ☒ Finding a professional interpreter
- ☒ Knowing how to work with a professional interpreter
- ☒ Time constraints
- ☒ Insufficiently trained health care providers
- ☒ Transportation problems for the patient
- ☒ Food insufficiency
- ☒ Need for child care
- ☒ Patients not understanding treatment plan
- ☒ Patients not following treatment plan
- ☒ My lack of knowledge regarding the patient's culture
- ☒ Bias or stereotyping
- ☒ Trust issues
- ☐ Other...

f. Rank how well immigrants and refugees understand the healthcare that you are trying to provide.

- ◯ Significantly less than a US born individual
- ⊗ Less than a US born individual
- ◯ Equivalent to a US born individual
- ◯ More than a US born patient
- ◯ Significantly more than a US born individual

g. Immigrants and refugees adhere to treatment plans and follow my recommendations.

- ◯ Never
- ◯ Rarely
- ⊗ Sometimes
- ◯ Usually
- ◯ Always

h. Immigrants and refugees should receive the same care and insurance coverage as US born patients.

- ◯ Never
- ◯ Rarely
- ⊗ Sometimes
- ◯ Usually
- ◯ Always

i. Immigrants and refugees who are undocumented should receive the same care and insurance coverage as US born patients.

- ◯ Never
- ⊗ Rarely
- ◯ Sometimes
- ◯ Usually
- ◯ Always

j. Every physician is professionally obligated to care for immigrants and refugees if they present to your clinic or hospital.

- ◯ Strongly disagree
- ◯ Disagree
- ◯ No opinion
- ⊗ Agree
- ◯ Strongly agree

k. Is healthcare a human right?

◯ Yes
⊗ No

B. If you wish, please tell us about what you enjoy or do not enjoy about immigrant and refugee health care and the greatest challenges you face in caring for this population.

____

SECTION D: DEMOGRAPHIC INFORMATION
Please answer the following questions by checking the box in front of the response choice that best describes you.

a. Your age?

◯ 20 to 24
⊗ 25 to 29
◯ 30 to 34
◯ 35 to 39
◯ 40 or older

b. Your gender?

⊗ Female
◯ Male
◯ Other

c. Are you Hispanic or Latino?

◯ Yes
⊗ No

d. What is your race? (Select one or more responses)

◯ American Indian or Alaska Native
◯ Asian (Please specify):
◯ Black or African American
◯ Native Hawaiian or Other Pacific Islander
⊗ White
◯ Other (Please specify):

e. Were you born in the United States?

⊗ Yes
◯ No

g. Your residency year?

⊗ PGY1
◯ PGY2
◯ PGY3
◯ PGY4
◯ PGY5

h. How would you classify your political ideology?

◯ Conservative
⊗ Somewhat conservative
◯ Moderate
◯ Somewhat liberal
◯ Liberal
◯ Other (Please specify):

i. Estimated level of educational debt?

◯ None
◯ Less than $50,000
◯ $50,000 - $100,000
◯ $100,000 - $200,000
⊗ $200,000 or more

j. Do you plan to subspecialize?

⊗ Yes
◯ No

k. Languages spoken?

- ☒ English
- ☒ Spanish
- ☐ French
- ☐ Hmong
- ☐ Somali
- ☐ Japanese
- ☐ Chinese
- ☐ Russian
- ☐ Ethiopian
- ☐ Other ___________

l. Are you in the Global Health Pathway?

- ⊗ Yes
- ◯ No

m. Did you earn your degree in the US?

- ⊗ Yes
- ◯ No

n. What residency program are you in?

- ⊗ Internal Medicine
- ◯ Med-Peds
- ◯ Pediatrics
- ◯ Family Practice
- ◯ Neurology
- ◯ Psychiatry
- ◯ ObGyn
- ◯ Neurosurgery
- ◯ General Surgery
- ◯ Orthopedic Surgery
- ◯ Urology
- ◯ Surgical sub-specialty (please specify in text box below)
- ◯ Non-clinical specialty (radiology, pathology; please specify in text box below)

Medical Trainees' attitudes, knowledge, and experience with immigrant and refugee health

Response was added on 11/01/2013 6:54pm.

SECTION A: Personal experience with immigrant and refugee health care.

A. Please indicate your level of agreement with the following statements regarding your personal experience with immigrant and refugee health care by checking the box that best represents your experience.

a. During my inpatient rotations, I take care of the following percentage of immigrant and refugee patients:
- ◯ None
- ◯ 0 -5%
- ◯ 5-10%
- ⊗ 10-25%
- ◯ > 25%

b. During my outpatient rotations, I take care of the following percentage of immigrant and refugee patients:
- ◯ None
- ◯ 0-10%
- ⊗ 10 -25%
- ◯ 25-50%
- ◯ 50-75%
- ◯ >75%

c. I would like to take care of more immigrant and refugee patients.
- ◯ Strongly disagree
- ⊗ Disagree
- ◯ No opinion
- ◯ Agree
- ◯ Strongly agree

d. I plan to take care of immigrants and refugees when I finish residency.
- ◯ Strongly disagree
- ◯ Disagree
- ◯ No opinion
- ⊗ Agree
- ◯ Strongly agree

e. I plan to do short term (< 6 months) international work when I finish residency.
- ◯ Strongly disagree
- ⊗ Disagree
- ◯ No opinion
- ◯ Agree
- ◯ Strongly agree

f. I plan to do long term (>6 months) international work when I finish residency.
- ◯ Strongly disagree
- ⊗ Disagree
- ◯ No opinion
- ◯ Agree
- ◯ Strongly agree

g. I plan to work in health disparities in the following way after residency:
Rural setting

SECTION B: MEDICAL EDUCATION

A. Please indicate your level of agreement with the following statements regarding your medical education and knowledge about immigrants and refugees by checking the box that best represents your opinion.

a. I have received specialized training in immigrant and refugee health, tropical medicine, or cross-cultural health.
- ◯ Strongly disagree
- ◯ Disagree
- ⊗ No opinion
- ◯ Agree
- ◯ Strongly agree

c. I feel comfortable with my fund of knowledge regarding immigrant and refugee health.
- ◯ Strongly disagree
- ◯ Disagree
- ◯ No opinion
- ⊗ Agree
- ◯ Strongly agree

d. I would like to have further training in immigrant and refugee health.
- ◯ Strongly disagree
- ⊗ Disagree
- ◯ No opinion
- ◯ Agree
- ◯ Strongly agree

SECTION C: Attitudes towards immigrant health

A.Please indicate your level of agreement with the following statements regarding immigrant and refugee health by checking the box that best represents your opinion.

a. I enjoy taking care of immigrants and refugees.
- ◯ Never
- ◯ Rarely
- ⊗ Sometimes
- ◯ Usually
- ◯ Always

b. Please indicate the reasons that you enjoy taking care of immigrants and refugees (may choose more than one).
- ☐ Tropical and other conditions not frequently diagnosed in US-born patients
- ☐ Learning about other cultures
- ☐ They don't complain as much
- ☐ Being able to hear their stories
- ☐ Their care is more complicated
- ☐ Their care is less complicated
- ☐ They are very appreciative of your help.
- ☐ They are extremely vulnerable
- ☐ Other:

c. Taking care of immigrants and refugees is more challenging than taking care of US born patients.
- ◯ Never
- ◯ Rarely
- ◯ Sometimes
- ⊗ Usually
- ◯ Always

d. Please mark all the challenges that you face as a provider when providing care to immigrants and refugees (may choose more than one):
- ☒ Language barriers
- ☐ Insurance barriers
- ☒ Cultural barriers
- ☒ Finding a professional interpreter
- ☐ Knowing how to work with a professional interpreter
- ☒ Time constraints
- ☐ My own knowledge related to tropical and travel medicine
- ☐ Transportation problems for the patient
- ☒ Patients not understanding treatment plan
- ☒ Patients not following treatment plan
- ☒ My lack of knowledge regarding the patient's culture
- ☐ Bias or stereotyping
- ☐ Other:

e. Please mark all of the challenges faced by immigrant and refugee populations when receiving healthcare that you have perceived or witnessed (may choose more than one):

- ☒ Language barriers
- ☐ Insurance barriers
- ☒ Cultural barriers
- ☒ Finding a professional interpreter
- ☐ Knowing how to work with a professional interpreter
- ☐ Time constraints
- ☐ Insufficiently trained health care providers
- ☐ Transportation problems for the patient
- ☐ Food insufficiency
- ☐ Need for child care
- ☒ Patients not understanding treatment plan
- ☐ Patients not following treatment plan
- ☐ My lack of knowledge regarding the patient's culture
- ☐ Bias or stereotyping
- ☐ Trust issues
- ☐ Other...

f. Rank how well immigrants and refugees understand the healthcare that you are trying to provide.

- ⊗ Significantly less than a US born individual
- ◯ Less than a US born individual
- ◯ Equivalent to a US born individual
- ◯ More than a US born patient
- ◯ Significantly more than a US born individual

g. Immigrants and refugees adhere to treatment plans and follow my recommendations.

- ◯ Never
- ◯ Rarely
- ⊗ Sometimes
- ◯ Usually
- ◯ Always

h. Immigrants and refugees should receive the same care and insurance coverage as US born patients.

- ◯ Never
- ◯ Rarely
- ◯ Sometimes
- ⊗ Usually
- ◯ Always

i. Immigrants and refugees who are undocumented should receive the same care and insurance coverage as US born patients.

- ◯ Never
- ⊗ Rarely
- ◯ Sometimes
- ◯ Usually
- ◯ Always

j. Every physician is professionally obligated to care for immigrants and refugees if they present to your clinic or hospital.

- ◯ Strongly disagree
- ◯ Disagree
- ◯ No opinion
- ⊗ Agree
- ◯ Strongly agree

k. Is healthcare a human right?

- ⊗ Yes
- ◯ No

B. If you wish, please tell us about what you enjoy or do not enjoy about immigrant and refugee health care and the greatest challenges you face in caring for this population.

____

SECTION D: DEMOGRAPHIC INFORMATION
Please answer the following questions by checking the box in front of the response choice that best describes you.

a. Your age?
- ◯ 20 to 24
- ⊗ 25 to 29
- ◯ 30 to 34
- ◯ 35 to 39
- ◯ 40 or older

b. Your gender?
- ⊗ Female
- ◯ Male
- ◯ Other

c. Are you Hispanic or Latino?
- ◯ Yes
- ⊗ No

d. What is your race? (Select one or more responses)
- ◯ American Indian or Alaska Native
- ◯ Asian (Please specify):
- ◯ Black or African American
- ◯ Native Hawaiian or Other Pacific Islander
- ⊗ White
- ◯ Other (Please specify):

e. Were you born in the United States?
- ⊗ Yes
- ◯ No

g. Your residency year?
- ◯ PGY1
- ⊗ PGY2
- ◯ PGY3
- ◯ PGY4
- ◯ PGY5

h. How would you classify your political ideology?
- ⊗ Conservative
- ◯ Somewhat conservative
- ◯ Moderate
- ◯ Somewhat liberal
- ◯ Liberal
- ◯ Other (Please specify):

i. Estimated level of educational debt?
- ◯ None
- ◯ Less than $50,000
- ◯ $50,000 - $100,000
- ⊗ $100,000 - $200,000
- ◯ $200,000 or more

j. Do you plan to subspecialize?
- ◯ Yes
- ⊗ No

k. Languages spoken?
- ☒ English
- ☐ Spanish
- ☐ French
- ☐ Hmong
- ☐ Somali
- ☐ Japanese
- ☐ Chinese
- ☐ Russian
- ☐ Ethiopian
- ☐ Other ___________

l. Are you in the Global Health Pathway?
- ◯ Yes
- ⊗ No

m. Did you earn your degree in the US?

- ⊗ Yes
- ◯ No

n. What residency program are you in?

- ◯ Internal Medicine
- ◯ Med-Peds
- ◯ Pediatrics
- ◯ Family Practice
- ◯ Neurology
- ◯ Psychiatry
- ◯ ObGyn
- ◯ Neurosurgery
- ⊗ General Surgery
- ◯ Orthopedic Surgery
- ◯ Urology
- ◯ Surgical sub-specialty (please specify in text box below)
- ◯ Non-clinical specialty (radiology, pathology; please specify in text box below)

Medical Trainees' attitudes, knowledge, and experience with immigrant and refugee health

Response was added on 11/01/2013 7:19pm.

SECTION A: Personal experience with immigrant and refugee health care.

A. Please indicate your level of agreement with the following statements regarding your personal experience with immigrant and refugee health care by checking the box that best represents your experience.

a. During my inpatient rotations, I take care of the following percentage of immigrant and refugee patients:

- ◯ None
- ◯ 0 -5%
- ◯ 5-10%
- ⊗ 10-25%
- ◯ > 25%

b. During my outpatient rotations, I take care of the following percentage of immigrant and refugee patients:

- ◯ None
- ◯ 0-10%
- ◯ 10 -25%
- ◯ 25-50%
- ⊗ 50-75%
- ◯ >75%

c. I would like to take care of more immigrant and refugee patients.

- ◯ Strongly disagree
- ◯ Disagree
- ⊗ No opinion
- ◯ Agree
- ◯ Strongly agree

d. I plan to take care of immigrants and refugees when I finish residency.

- ◯ Strongly disagree
- ◯ Disagree
- ◯ No opinion
- ◯ Agree
- ⊗ Strongly agree

e. I plan to do short term (< 6 months) international work when I finish residency.

- ◯ Strongly disagree
- ⊗ Disagree
- ◯ No opinion
- ◯ Agree
- ◯ Strongly agree

f. I plan to do long term (>6 months) international work when I finish residency.

- ◯ Strongly disagree
- ⊗ Disagree
- ◯ No opinion
- ◯ Agree
- ◯ Strongly agree

g. I plan to work in health disparities in the following way after residency:

I hope to continue to working in primary care in an urban area and clinic with similar immigrant make up after graduation. I would love to research and address public health issues that relate to immigrants

SECTION B: MEDICAL EDUCATION

A. Please indicate your level of agreement with the following statements regarding your medical education and knowledge about immigrants and refugees by checking the box that best represents your opinion.

a. I have received specialized training in immigrant and refugee health, tropical medicine, or cross-cultural health.

- ◯ Strongly disagree
- ◯ Disagree
- ◯ No opinion
- ⊗ Agree
- ◯ Strongly agree

b. If you have received specialized training in immigrant and refugee health, tropical medicine, or cross-cultural health, please indicate all the contexts in which you received this training:

- ☐ As an undergraduate.
- ☐ As a medical student.
- ☒ As part of my residency.
- ☐ A special program.
- ☐ As part of my fellowship.
- ☐ As part of a degree program (e.g. MPH)
- ☐ Other:

c. I feel comfortable with my fund of knowledge regarding immigrant and refugee health.

- ◯ Strongly disagree
- ⊗ Disagree
- ◯ No opinion
- ◯ Agree
- ◯ Strongly agree

d. I would like to have further training in immigrant and refugee health.

- ◯ Strongly disagree
- ◯ Disagree
- ◯ No opinion
- ⊗ Agree
- ◯ Strongly agree

e. If you agree with the above, please indicate all the contexts in which you would like to receive this training:

- ☒ As part of my residency.
- ☒ A special program.
- ☐ As part of my fellowship.

SECTION C: Attitudes towards immigrant health

A. Please indicate your level of agreement with the following statements regarding immigrant and refugee health by checking the box that best represents your opinion.

a. I enjoy taking care of immigrants and refugees.

- ◯ Never
- ◯ Rarely
- ◯ Sometimes
- ⊗ Usually
- ◯ Always

b. Please indicate the reasons that you enjoy taking care of immigrants and refugees (may choose more than one).

- ☒ Tropical and other conditions not frequently diagnosed in US-born patients
- ☒ Learning about other cultures
- ☒ They don't complain as much
- ☒ Being able to hear their stories
- ☐ Their care is more complicated
- ☐ Their care is less complicated
- ☒ They are very appreciative of your help.
- ☐ They are extremely vulnerable
- ☐ Other:

c. Taking care of immigrants and refugees is more challenging than taking care of US born patients.

- ◯ Never
- ◯ Rarely
- ◯ Sometimes
- ⊗ Usually
- ◯ Always

d. Please mark all the challenges that you face as a provider when providing care to immigrants and refugees (may choose more than one):

- ☒ Language barriers
- ☐ Insurance barriers
- ☒ Cultural barriers
- ☐ Finding a professional interpreter
- ☐ Knowing how to work with a professional interpreter
- ☒ Time constraints
- ☒ My own knowledge related to tropical and travel medicine
- ☒ Transportation problems for the patient
- ☒ Patients not understanding treatment plan
- ☒ Patients not following treatment plan
- ☐ My lack of knowledge regarding the patient's culture
- ☐ Bias or stereotyping
- ☐ Other:

e. Please mark all of the challenges faced by immigrant and refugee populations when receiving healthcare that you have perceived or witnessed (may choose more than one):

- ☒ Language barriers
- ☒ Insurance barriers
- ☒ Cultural barriers
- ☒ Finding a professional interpreter
- ☒ Knowing how to work with a professional interpreter
- ☒ Time constraints
- ☒ Insufficiently trained health care providers
- ☒ Transportation problems for the patient
- ☒ Food insufficiency
- ☒ Need for child care
- ☒ Patients not understanding treatment plan
- ☒ Patients not following treatment plan
- ☒ My lack of knowledge regarding the patient's culture
- ☒ Bias or stereotyping
- ☒ Trust issues
- ☐ Other...

f. Rank how well immigrants and refugees understand the healthcare that you are trying to provide.

- ◯ Significantly less than a US born individual
- ⊗ Less than a US born individual
- ◯ Equivalent to a US born individual
- ◯ More than a US born patient
- ◯ Significantly more than a US born individual

g. Immigrants and refugees adhere to treatment plans and follow my recommendations.

- ◯ Never
- ◯ Rarely
- ⊗ Sometimes
- ◯ Usually
- ◯ Always

h. Immigrants and refugees should receive the same care and insurance coverage as US born patients.

- ◯ Never
- ◯ Rarely
- ◯ Sometimes
- ◯ Usually
- ⊗ Always

i. Immigrants and refugees who are undocumented should receive the same care and insurance coverage as US born patients.

- ◯ Never
- ◯ Rarely
- ◯ Sometimes
- ◯ Usually
- ⊗ Always

j. Every physician is professionally obligated to care for immigrants and refugees if they present to your clinic or hospital.

- ◯ Strongly disagree
- ◯ Disagree
- ◯ No opinion
- ⊗ Agree
- ◯ Strongly agree

k. Is healthcare a human right?

- ⊗ Yes
- ◯ No

B. If you wish, please tell us about what you enjoy or do not enjoy about immigrant and refugee health care and the greatest challenges you face in caring for this population.

____

SECTION D: DEMOGRAPHIC INFORMATION
Please answer the following questions by checking the box in front of the response choice that best describes you.

a. Your age?

- ◯ 20 to 24
- ◯ 25 to 29
- ⊗ 30 to 34
- ◯ 35 to 39
- ◯ 40 or older

b. Your gender?

- ⊗ Female
- ◯ Male
- ◯ Other

c.Are you Hispanic or Latino?

- ◯ Yes
- ⊗ No

d. What is your race? (Select one or more responses)

- ◯ American Indian or Alaska Native
- ◯ Asian (Please specify):
- ◯ Black or African American
- ◯ Native Hawaiian or Other Pacific Islander
- ⊗ White
- ◯ Other (Please specify):

e.Were you born in the United States?

- ⊗ Yes
- ◯ No

g. Your residency year?

- ◯ PGY1
- ◯ PGY2
- ⊗ PGY3
- ◯ PGY4
- ◯ PGY5

h. How would you classify your political ideology?

- ◯ Conservative
- ◯ Somewhat conservative
- ◯ Moderate
- ◯ Somewhat liberal
- ⊗ Liberal
- ◯ Other (Please specify):

i. Estimated level of educational debt?

- ◯ None
- ◯ Less than $50,000
- ◯ $50,000 - $100,000
- ⊗ $100,000 - $200,000
- ◯ $200,000 or more

j.Do you plan to subspecialize?
- ◯ Yes
- ⊗ No

k. Languages spoken?
- ☒ English
- ☒ Spanish
- ☐ French
- ☐ Hmong
- ☐ Somali
- ☐ Japanese
- ☐ Chinese
- ☐ Russian
- ☐ Ethiopian
- ☐ Other ___________

l. Are you in the Global Health Pathway?
- ◯ Yes
- ⊗ No

m. Did you earn your degree in the US?
- ⊗ Yes
- ◯ No

n. What residency program are you in?
- ◯ Internal Medicine
- ◯ Med-Peds
- ◯ Pediatrics
- ⊗ Family Practice
- ◯ Neurology
- ◯ Psychiatry
- ◯ ObGyn
- ◯ Neurosurgery
- ◯ General Surgery
- ◯ Orthopedic Surgery
- ◯ Urology
- ◯ Surgical sub-specialty (please specify in text box below)
- ◯ Non-clinical specialty (radiology, pathology; please specify in text box below)

Medical Trainees' attitudes, knowledge, and experience with immigrant and refugee health

Response was added on 11/01/2013 7:26pm.

SECTION A: Personal experience with immigrant and refugee health care.

A. Please indicate your level of agreement with the following statements regarding your personal experience with immigrant and refugee health care by checking the box that best represents your experience.

a. During my inpatient rotations, I take care of the following percentage of immigrant and refugee patients:
- ◯ None
- ◯ 0 -5%
- ⊗ 5-10%
- ◯ 10-25%
- ◯ > 25%

b. During my outpatient rotations, I take care of the following percentage of immigrant and refugee patients:
- ◯ None
- ◯ 0-10%
- ⊗ 10 -25%
- ◯ 25-50%
- ◯ 50-75%
- ◯ >75%

c. I would like to take care of more immigrant and refugee patients.
- ◯ Strongly disagree
- ◯ Disagree
- ◯ No opinion
- ⊗ Agree
- ◯ Strongly agree

d. I plan to take care of immigrants and refugees when I finish residency.
- ◯ Strongly disagree
- ◯ Disagree
- ◯ No opinion
- ⊗ Agree
- ◯ Strongly agree

e. I plan to do short term (< 6 months) international work when I finish residency.
- ◯ Strongly disagree
- ◯ Disagree
- ◯ No opinion
- ⊗ Agree
- ◯ Strongly agree

f. I plan to do long term (>6 months) international work when I finish residency.
- ◯ Strongly disagree
- ⊗ Disagree
- ◯ No opinion
- ◯ Agree
- ◯ Strongly agree

g. I plan to work in health disparities in the following way after residency:

Ob gyn care and training in mn and abroad

SECTION B: MEDICAL EDUCATION

A. Please indicate your level of agreement with the following statements regarding your medical education and knowledge about immigrants and refugees by checking the box that best represents your opinion.

a. I have received specialized training in immigrant and refugee health, tropical medicine, or cross-cultural health.

◯ Strongly disagree
◯ Disagree
◯ No opinion
⊗ Agree
◯ Strongly agree

b. If you have received specialized training in immigrant and refugee health, tropical medicine, or cross-cultural health, please indicate all the contexts in which you received this training:

☐ As an undergraduate.
☒ As a medical student.
☒ As part of my residency.
☐ A special program.
☐ As part of my fellowship.
☒ As part of a degree program (e.g. MPH)
☐ Other:

c. I feel comfortable with my fund of knowledge regarding immigrant and refugee health.

◯ Strongly disagree
◯ Disagree
◯ No opinion
⊗ Agree
◯ Strongly agree

d. I would like to have further training in immigrant and refugee health.

◯ Strongly disagree
◯ Disagree
◯ No opinion
⊗ Agree
◯ Strongly agree

e. If you agree with the above, please indicate all the contexts in which you would like to receive this training:
□□

☒ As part of my residency.
☐ A special program.
☐ As part of my fellowship.

SECTION C: Attitudes towards immigrant health

A.□Please indicate your level of agreement with the following statements regarding immigrant and refugee health by checking the box that best represents your opinion.

a. I enjoy taking care of immigrants and refugees.

◯ Never
◯ Rarely
◯ Sometimes
⊗ Usually
◯ Always

b. Please indicate the reasons that you enjoy taking care of immigrants and refugees (may choose more than one).

☐ Tropical and other conditions not frequently diagnosed in US-born patients
☒ Learning about other cultures
☐ They don't complain as much
☒ Being able to hear their stories
☐ Their care is more complicated
☐ Their care is less complicated
☒ They are very appreciative of your help.
☒ They are extremely vulnerable
☐ Other:

c. Taking care of immigrants and refugees is more challenging than taking care of US born patients.

◯ Never
◯ Rarely
⊗ Sometimes
◯ Usually
◯ Always

d. Please mark all the challenges that you face as a provider when providing care to immigrants and refugees (may choose more than one):

- ☒ Language barriers
- ☒ Insurance barriers
- ☒ Cultural barriers
- ☒ Finding a professional interpreter
- ☐ Knowing how to work with a professional interpreter
- ☒ Time constraints
- ☒ My own knowledge related to tropical and travel medicine
- ☒ Transportation problems for the patient
- ☒ Patients not understanding treatment plan
- ☒ Patients not following treatment plan
- ☒ My lack of knowledge regarding the patient's culture
- ☐ Bias or stereotyping
- ☐ Other:

e. Please mark all of the challenges faced by immigrant and refugee populations when receiving healthcare that you have perceived or witnessed (may choose more than one):

- ☒ Language barriers
- ☒ Insurance barriers
- ☒ Cultural barriers
- ☐ Finding a professional interpreter
- ☐ Knowing how to work with a professional interpreter
- ☐ Time constraints
- ☐ Insufficiently trained health care providers
- ☒ Transportation problems for the patient
- ☐ Food insufficiency
- ☐ Need for child care
- ☒ Patients not understanding treatment plan
- ☐ Patients not following treatment plan
- ☐ My lack of knowledge regarding the patient's culture
- ☐ Bias or stereotyping
- ☒ Trust issues
- ☐ Other...

f. Rank how well immigrants and refugees understand the healthcare that you are trying to provide.

- ◯ Significantly less than a US born individual
- ⊗ Less than a US born individual
- ◯ Equivalent to a US born individual
- ◯ More than a US born patient
- ◯ Significantly more than a US born individual

g. Immigrants and refugees adhere to treatment plans and follow my recommendations.

- ◯ Never
- ◯ Rarely
- ⊗ Sometimes
- ◯ Usually
- ◯ Always

h. Immigrants and refugees should receive the same care and insurance coverage as US born patients.

- ◯ Never
- ◯ Rarely
- ◯ Sometimes
- ⊗ Usually
- ◯ Always

i. Immigrants and refugees who are undocumented should receive the same care and insurance coverage as US born patients.

- ◯ Never
- ◯ Rarely
- ⊗ Sometimes
- ◯ Usually
- ◯ Always

j. Every physician is professionally obligated to care for immigrants and refugees if they present to your clinic or hospital.

- ◯ Strongly disagree
- ◯ Disagree
- ◯ No opinion
- ◯ Agree
- ⊗ Strongly agree

k. Is healthcare a human right?
- ⊗ Yes
- ◯ No

B. If you wish, please tell us about what you enjoy or do not enjoy about immigrant and refugee health care and the greatest challenges you face in caring for this population.
____

SECTION D: DEMOGRAPHIC INFORMATION
Please answer the following questions by checking the box in front of the response choice that best describes you.

a. Your age?
- ◯ 20 to 24
- ◯ 25 to 29
- ⊗ 30 to 34
- ◯ 35 to 39
- ◯ 40 or older

b. Your gender?
- ⊗ Female
- ◯ Male
- ◯ Other

c. Are you Hispanic or Latino?
- ◯ Yes
- ⊗ No

d. What is your race? (Select one or more responses)
- ◯ American Indian or Alaska Native
- ◯ Asian (Please specify):
- ◯ Black or African American
- ◯ Native Hawaiian or Other Pacific Islander
- ⊗ White
- ◯ Other (Please specify):

e. Were you born in the United States?
- ⊗ Yes
- ◯ No

g. Your residency year?
- ◯ PGY1
- ◯ PGY2
- ◯ PGY3
- ⊗ PGY4
- ◯ PGY5

h. How would you classify your political ideology?
- ◯ Conservative
- ◯ Somewhat conservative
- ◯ Moderate
- ◯ Somewhat liberal
- ⊗ Liberal
- ◯ Other (Please specify):

i. Estimated level of educational debt?
- ◯ None
- ◯ Less than $50,000
- ◯ $50,000 - $100,000
- ⊗ $100,000 - $200,000
- ◯ $200,000 or more

j. Do you plan to subspecialize?
- ◯ Yes
- ⊗ No

k. Languages spoken?

- [x] English
- [x] Spanish
- [ ] French
- [ ] Hmong
- [ ] Somali
- [ ] Japanese
- [ ] Chinese
- [ ] Russian
- [ ] Ethiopian
- [ ] Other ___________

l. Are you in the Global Health Pathway?

- ◯ Yes
- ⊗ No

m. Did you earn your degree in the US?

- ⊗ Yes
- ◯ No

n. What residency program are you in?

- ◯ Internal Medicine
- ◯ Med-Peds
- ◯ Pediatrics
- ◯ Family Practice
- ◯ Neurology
- ◯ Psychiatry
- ⊗ ObGyn
- ◯ Neurosurgery
- ◯ General Surgery
- ◯ Orthopedic Surgery
- ◯ Urology
- ◯ Surgical sub-specialty (please specify in text box below)
- ◯ Non-clinical specialty (radiology, pathology; please specify in text box below)

Medical Trainees' attitudes, knowledge, and experience with immigrant and refugee health

Response was added on 11/01/2013 7:27pm.

SECTION A: Personal experience with immigrant and refugee health care.

A. Please indicate your level of agreement with the following statements regarding your personal experience with immigrant and refugee health care by checking the box that best represents your experience.

a. During my inpatient rotations, I take care of the following percentage of immigrant and refugee patients:

○ None
○ 0 -5%
⊗ 5-10%
○ 10-25%
○ > 25%

b. During my outpatient rotations, I take care of the following percentage of immigrant and refugee patients:

○ None
⊗ 0-10%
○ 10 -25%
○ 25-50%
○ 50-75%
○ >75%

c. I would like to take care of more immigrant and refugee patients.

○ Strongly disagree
○ Disagree
⊗ No opinion
○ Agree
○ Strongly agree

d. I plan to take care of immigrants and refugees when I finish residency.

○ Strongly disagree
⊗ Disagree
○ No opinion
○ Agree
○ Strongly agree

e. I plan to do short term (< 6 months) international work when I finish residency.

○ Strongly disagree
⊗ Disagree
○ No opinion
○ Agree
○ Strongly agree

f. I plan to do long term (>6 months) international work when I finish residency.

○ Strongly disagree
⊗ Disagree
○ No opinion
○ Agree
○ Strongly agree

g. I plan to work in health disparities in the following way after residency:

None

SECTION B: MEDICAL EDUCATION

A. Please indicate your level of agreement with the following statements regarding your medical education and knowledge about immigrants and refugees by checking the box that best represents your opinion.

a. I have received specialized training in immigrant and refugee health, tropical medicine, or cross-cultural health.

⊗ Strongly disagree
○ Disagree
○ No opinion
○ Agree
○ Strongly agree

c. I feel comfortable with my fund of knowledge regarding immigrant and refugee health.

⊗ Strongly disagree
○ Disagree
○ No opinion
○ Agree
○ Strongly agree

d. I would like to have further training in immigrant and refugee health.

⊗ Strongly disagree
○ Disagree
○ No opinion
○ Agree
○ Strongly agree

SECTION C: Attitudes towards immigrant health

A.Please indicate your level of agreement with the following statements regarding immigrant and refugee health by checking the box that best represents your opinion.

a. I enjoy taking care of immigrants and refugees.

○ Never
○ Rarely
○ Sometimes
⊗ Usually
○ Always

b. Please indicate the reasons that you enjoy taking care of immigrants and refugees (may choose more than one).

☐ Tropical and other conditions not frequently diagnosed in US-born patients
☒ Learning about other cultures
☐ They don't complain as much
☐ Being able to hear their stories
☐ Their care is more complicated
☐ Their care is less complicated
☒ They are very appreciative of your help.
☐ They are extremely vulnerable
☐ Other:

c. Taking care of immigrants and refugees is more challenging than taking care of US born patients.

○ Never
⊗ Rarely
○ Sometimes
○ Usually
○ Always

d. Please mark all the challenges that you face as a provider when providing care to immigrants and refugees (may choose more than one):

☒ Language barriers
☐ Insurance barriers
☒ Cultural barriers
☒ Finding a professional interpreter
☐ Knowing how to work with a professional interpreter
☒ Time constraints
☐ My own knowledge related to tropical and travel medicine
☐ Transportation problems for the patient
☒ Patients not understanding treatment plan
☒ Patients not following treatment plan
☒ My lack of knowledge regarding the patient's culture
☐ Bias or stereotyping
☐ Other:

e. Please mark all of the challenges faced by immigrant and refugee populations when receiving healthcare that you have perceived or witnessed (may choose more than one):

- ☒ Language barriers
- ☐ Insurance barriers
- ☐ Cultural barriers
- ☐ Finding a professional interpreter
- ☒ Knowing how to work with a professional interpreter
- ☐ Time constraints
- ☐ Insufficiently trained health care providers
- ☒ Transportation problems for the patient
- ☐ Food insufficiency
- ☐ Need for child care
- ☐ Patients not understanding treatment plan
- ☐ Patients not following treatment plan
- ☐ My lack of knowledge regarding the patient's culture
- ☐ Bias or stereotyping
- ☐ Trust issues
- ☐ Other...

f. Rank how well immigrants and refugees understand the healthcare that you are trying to provide.

- ◯ Significantly less than a US born individual
- ⊗ Less than a US born individual
- ◯ Equivalent to a US born individual
- ◯ More than a US born patient
- ◯ Significantly more than a US born individual

g. Immigrants and refugees adhere to treatment plans and follow my recommendations.

- ◯ Never
- ◯ Rarely
- ⊗ Sometimes
- ◯ Usually
- ◯ Always

h. Immigrants and refugees should receive the same care and insurance coverage as US born patients.

- ◯ Never
- ◯ Rarely
- ◯ Sometimes
- ◯ Usually
- ⊗ Always

i. Immigrants and refugees who are undocumented should receive the same care and insurance coverage as US born patients.

- ◯ Never
- ◯ Rarely
- ◯ Sometimes
- ◯ Usually
- ⊗ Always

j. Every physician is professionally obligated to care for immigrants and refugees if they present to your clinic or hospital.

- ◯ Strongly disagree
- ◯ Disagree
- ◯ No opinion
- ◯ Agree
- ⊗ Strongly agree

k. Is healthcare a human right?

- ⊗ Yes
- ◯ No

B. If you wish, please tell us about what you enjoy or do not enjoy about immigrant and refugee health care and the greatest challenges you face in caring for this population.

____

SECTION D: DEMOGRAPHIC INFORMATION
Please answer the following questions by checking the box in front of the response choice that best describes you.

a. Your age?
- ◯ 20 to 24
- ◯ 25 to 29
- ◯ 30 to 34
- ⊗ 35 to 39
- ◯ 40 or older

b. Your gender?
- ◯ Female
- ⊗ Male
- ◯ Other

c. Are you Hispanic or Latino?
- ◯ Yes
- ⊗ No

d. What is your race? (Select one or more responses)
- ◯ American Indian or Alaska Native
- ◯ Asian (Please specify):
- ◯ Black or African American
- ◯ Native Hawaiian or Other Pacific Islander
- ⊗ White
- ◯ Other (Please specify):

e. Were you born in the United States?
- ⊗ Yes
- ◯ No

g. Your residency year?
- ◯ PGY1
- ◯ PGY2
- ◯ PGY3
- ◯ PGY4
- ⊗ PGY5

h. How would you classify your political ideology?
- ◯ Conservative
- ◯ Somewhat conservative
- ⊗ Moderate
- ◯ Somewhat liberal
- ◯ Liberal
- ◯ Other (Please specify):

i. Estimated level of educational debt?
- ◯ None
- ◯ Less than $50,000
- ◯ $50,000 - $100,000
- ⊗ $100,000 - $200,000
- ◯ $200,000 or more

j. Do you plan to subspecialize?
- ◯ Yes
- ⊗ No

k. Languages spoken?
- ☒ English
- ☐ Spanish
- ☐ French
- ☐ Hmong
- ☐ Somali
- ☐ Japanese
- ☐ Chinese
- ☐ Russian
- ☐ Ethiopian
- ☐ Other ___________

l. Are you in the Global Health Pathway?
- ◯ Yes
- ⊗ No

m. Did you earn your degree in the US?

- ⊗ Yes
- ◯ No

n. What residency program are you in?

- ◯ Internal Medicine
- ◯ Med-Peds
- ◯ Pediatrics
- ◯ Family Practice
- ◯ Neurology
- ◯ Psychiatry
- ◯ ObGyn
- ◯ Neurosurgery
- ⊗ General Surgery
- ◯ Orthopedic Surgery
- ◯ Urology
- ◯ Surgical sub-specialty (please specify in text box below)
- ◯ Non-clinical specialty (radiology, pathology; please specify in text box below)

Medical Trainees' attitudes, knowledge, and experience with immigrant and refugee health

Response was added on 11/01/2013 7:32pm.

SECTION A: Personal experience with immigrant and refugee health care.

A. Please indicate your level of agreement with the following statements regarding your personal experience with immigrant and refugee health care by checking the box that best represents your experience.

a. During my inpatient rotations, I take care of the following percentage of immigrant and refugee patients:	⊗ None<br>○ 0 -5%<br>○ 5-10%<br>○ 10-25%<br>○ > 25%
b. During my outpatient rotations, I take care of the following percentage of immigrant and refugee patients:	⊗ None<br>○ 0-10%<br>○ 10 -25%<br>○ 25-50%<br>○ 50-75%<br>○ >75%
c. I would like to take care of more immigrant and refugee patients.	○ Strongly disagree<br>○ Disagree<br>⊗ No opinion<br>○ Agree<br>○ Strongly agree
d. I plan to take care of immigrants and refugees when I finish residency.	○ Strongly disagree<br>○ Disagree<br>○ No opinion<br>○ Agree<br>⊗ Strongly agree
e. I plan to do short term (< 6 months) international work when I finish residency.	○ Strongly disagree<br>○ Disagree<br>○ No opinion<br>○ Agree<br>⊗ Strongly agree
f. I plan to do long term (>6 months) international work when I finish residency.	○ Strongly disagree<br>⊗ Disagree<br>○ No opinion<br>○ Agree<br>○ Strongly agree
g. I plan to work in health disparities in the following way after residency:	10Forensic pathology

SECTION B: MEDICAL EDUCATION

A. Please indicate your level of agreement with the following statements regarding your medical education and knowledge about immigrants and refugees by checking the box that best represents your opinion.

a. I have received specialized training in immigrant and refugee health, tropical medicine, or cross-cultural health.

- ◯ Strongly disagree
- ◯ Disagree
- ◯ No opinion
- ⊗ Agree
- ◯ Strongly agree

b. If you have received specialized training in immigrant and refugee health, tropical medicine, or cross-cultural health, please indicate all the contexts in which you received this training:

- ☐ As an undergraduate.
- ☒ As a medical student.
- ☐ As part of my residency.
- ☐ A special program.
- ☐ As part of my fellowship.
- ☐ As part of a degree program (e.g. MPH)
- ☐ Other:

c. I feel comfortable with my fund of knowledge regarding immigrant and refugee health.

- ◯ Strongly disagree
- ◯ Disagree
- ◯ No opinion
- ⊗ Agree
- ◯ Strongly agree

d. I would like to have further training in immigrant and refugee health.

- ◯ Strongly disagree
- ◯ Disagree
- ◯ No opinion
- ⊗ Agree
- ◯ Strongly agree

e. If you agree with the above, please indicate all the contexts in which you would like to receive this training:
□□

- ☒ As part of my residency.
- ☐ A special program.
- ☐ As part of my fellowship.

SECTION C: Attitudes towards immigrant health

A.□Please indicate your level of agreement with the following statements regarding immigrant and refugee health by checking the box that best represents your opinion.

a. I enjoy taking care of immigrants and refugees.

- ◯ Never
- ◯ Rarely
- ◯ Sometimes
- ◯ Usually
- ⊗ Always

b. Please indicate the reasons that you enjoy taking care of immigrants and refugees (may choose more than one).

- ☒ Tropical and other conditions not frequently diagnosed in US-born patients
- ☒ Learning about other cultures
- ☐ They don't complain as much
- ☒ Being able to hear their stories
- ☐ Their care is more complicated
- ☐ Their care is less complicated
- ☒ They are very appreciative of your help.
- ☐ They are extremely vulnerable
- ☐ Other:

c. Taking care of immigrants and refugees is more challenging than taking care of US born patients.

- ◯ Never
- ◯ Rarely
- ⊗ Sometimes
- ◯ Usually
- ◯ Always

d. Please mark all the challenges that you face as a provider when providing care to immigrants and refugees (may choose more than one):

☒ Language barriers
☐ Insurance barriers
☒ Cultural barriers
☐ Finding a professional interpreter
☒ Knowing how to work with a professional interpreter
☐ Time constraints
☐ My own knowledge related to tropical and travel medicine
☐ Transportation problems for the patient
☐ Patients not understanding treatment plan
☒ Patients not following treatment plan
☒ My lack of knowledge regarding the patient's culture
☐ Bias or stereotyping
☐ Other:

e. Please mark all of the challenges faced by immigrant and refugee populations when receiving healthcare that you have perceived or witnessed (may choose more than one):

☒ Language barriers
☒ Insurance barriers
☒ Cultural barriers
☒ Finding a professional interpreter
☒ Knowing how to work with a professional interpreter
☒ Time constraints
☒ Insufficiently trained health care providers
☒ Transportation problems for the patient
☒ Food insufficiency
☒ Need for child care
☒ Patients not understanding treatment plan
☒ Patients not following treatment plan
☒ My lack of knowledge regarding the patient's culture
☒ Bias or stereotyping
☒ Trust issues
☐ Other...

f. Rank how well immigrants and refugees understand the healthcare that you are trying to provide.

◯ Significantly less than a US born individual
◯ Less than a US born individual
⊗ Equivalent to a US born individual
◯ More than a US born patient
◯ Significantly more than a US born individual

g. Immigrants and refugees adhere to treatment plans and follow my recommendations.

◯ Never
◯ Rarely
⊗ Sometimes
◯ Usually
◯ Always

h. Immigrants and refugees should receive the same care and insurance coverage as US born patients.

◯ Never
◯ Rarely
◯ Sometimes
⊗ Usually
◯ Always

i. Immigrants and refugees who are undocumented should receive the same care and insurance coverage as US born patients.

◯ Never
◯ Rarely
◯ Sometimes
⊗ Usually
◯ Always

j. Every physician is professionally obligated to care for immigrants and refugees if they present to your clinic or hospital.

◯ Strongly disagree
◯ Disagree
◯ No opinion
◯ Agree
⊗ Strongly agree

k. Is healthcare a human right?
- ⊗ Yes
- ◯ No

B. If you wish, please tell us about what you enjoy or do not enjoy about immigrant and refugee health care and the greatest challenges you face in caring for this population.

____

SECTION D: DEMOGRAPHIC INFORMATION
Please answer the following questions by checking the box in front of the response choice that best describes you.

a. Your age?
- ◯ 20 to 24
- ◯ 25 to 29
- ◯ 30 to 34
- ⊗ 35 to 39
- ◯ 40 or older

b. Your gender?
- ⊗ Female
- ◯ Male
- ◯ Other

c.Are you Hispanic or Latino?
- ◯ Yes
- ⊗ No

d. What is your race? (Select one or more responses)
- ◯ American Indian or Alaska Native
- ◯ Asian (Please specify):
- ◯ Black or African American
- ◯ Native Hawaiian or Other Pacific Islander
- ⊗ White
- ◯ Other (Please specify):

e.Were you born in the United States?
- ⊗ Yes
- ◯ No

g. Your residency year?
- ◯ PGY1
- ⊗ PGY2
- ◯ PGY3
- ◯ PGY4
- ◯ PGY5

h. How would you classify your political ideology?
- ◯ Conservative
- ◯ Somewhat conservative
- ◯ Moderate
- ◯ Somewhat liberal
- ⊗ Liberal
- ◯ Other (Please specify):

i. Estimated level of educational debt?
- ◯ None
- ◯ Less than $50,000
- ◯ $50,000 - $100,000
- ◯ $100,000 - $200,000
- ⊗ $200,000 or more

j.Do you plan to subspecialize?
- ⊗ Yes
- ◯ No

k. Languages spoken?

- ☒ English
- ☒ Spanish
- ☐ French
- ☐ Hmong
- ☐ Somali
- ☐ Japanese
- ☐ Chinese
- ☐ Russian
- ☐ Ethiopian
- ☐ Other ___________

l. Are you in the Global Health Pathway?

- ◯ Yes
- ⊗ No

m. Did you earn your degree in the US?

- ◯ Yes
- ⊗ No

n. What residency program are you in?

- ◯ Internal Medicine
- ◯ Med-Peds
- ◯ Pediatrics
- ◯ Family Practice
- ◯ Neurology
- ◯ Psychiatry
- ◯ ObGyn
- ◯ Neurosurgery
- ◯ General Surgery
- ◯ Orthopedic Surgery
- ◯ Urology
- ◯ Surgical sub-specialty (please specify in text box below)
- ⊗ Non-clinical specialty (radiology, pathology; please specify in text box below)

Pathology

Medical Trainees' attitudes, knowledge, and experience with immigrant and refugee health

Response was added on 11/01/2013 8:20pm.

SECTION A: Personal experience with immigrant and refugee health care.

A. Please indicate your level of agreement with the following statements regarding your personal experience with immigrant and refugee health care by checking the box that best represents your experience.

a. During my inpatient rotations, I take care of the following percentage of immigrant and refugee patients:

- ◯ None
- ◯ 0 -5%
- ⊗ 5-10%
- ◯ 10-25%
- ◯ > 25%

b. During my outpatient rotations, I take care of the following percentage of immigrant and refugee patients:

- ◯ None
- ◯ 0-10%
- ◯ 10 -25%
- ◯ 25-50%
- ◯ 50-75%
- ⊗ >75%

c. I would like to take care of more immigrant and refugee patients.

- ◯ Strongly disagree
- ◯ Disagree
- ⊗ No opinion
- ◯ Agree
- ◯ Strongly agree

d. I plan to take care of immigrants and refugees when I finish residency.

- ◯ Strongly disagree
- ◯ Disagree
- ◯ No opinion
- ◯ Agree
- ⊗ Strongly agree

e. I plan to do short term (< 6 months) international work when I finish residency.

- ◯ Strongly disagree
- ◯ Disagree
- ◯ No opinion
- ⊗ Agree
- ◯ Strongly agree

f. I plan to do long term (>6 months) international work when I finish residency.

- ⊗ Strongly disagree
- ◯ Disagree
- ◯ No opinion
- ◯ Agree
- ◯ Strongly agree

g. I plan to work in health disparities in the following way after residency:

Political advocacy

SECTION B: MEDICAL EDUCATION

A. Please indicate your level of agreement with the following statements regarding your medical education and knowledge about immigrants and refugees by checking the box that best represents your opinion.

a. I have received specialized training in immigrant and refugee health, tropical medicine, or cross-cultural health.

- ⊗ Strongly disagree
- ○ Disagree
- ○ No opinion
- ○ Agree
- ○ Strongly agree

c. I feel comfortable with my fund of knowledge regarding immigrant and refugee health.

- ⊗ Strongly disagree
- ○ Disagree
- ○ No opinion
- ○ Agree
- ○ Strongly agree

d. I would like to have further training in immigrant and refugee health.

- ○ Strongly disagree
- ○ Disagree
- ○ No opinion
- ○ Agree
- ⊗ Strongly agree

e. If you agree with the above, please indicate all the contexts in which you would like to receive this training:

- ☒ As part of my residency.
- ☐ A special program.
- ☐ As part of my fellowship.

SECTION C: Attitudes towards immigrant health

A. Please indicate your level of agreement with the following statements regarding immigrant and refugee health by checking the box that best represents your opinion.

a. I enjoy taking care of immigrants and refugees.

- ○ Never
- ○ Rarely
- ○ Sometimes
- ⊗ Usually
- ○ Always

b. Please indicate the reasons that you enjoy taking care of immigrants and refugees (may choose more than one).

- ☒ Tropical and other conditions not frequently diagnosed in US-born patients
- ☒ Learning about other cultures
- ☐ They don't complain as much
- ☒ Being able to hear their stories
- ☒ Their care is more complicated
- ☐ Their care is less complicated
- ☒ They are very appreciative of your help.
- ☒ They are extremely vulnerable
- ☐ Other:

c. Taking care of immigrants and refugees is more challenging than taking care of US born patients.

- ○ Never
- ○ Rarely
- ⊗ Sometimes
- ○ Usually
- ○ Always

d. Please mark all the challenges that you face as a provider when providing care to immigrants and refugees (may choose more than one):

- ☒ Language barriers
- ☐ Insurance barriers
- ☒ Cultural barriers
- ☒ Finding a professional interpreter
- ☐ Knowing how to work with a professional interpreter
- ☒ Time constraints
- ☒ My own knowledge related to tropical and travel medicine
- ☐ Transportation problems for the patient
- ☒ Patients not understanding treatment plan
- ☐ Patients not following treatment plan
- ☒ My lack of knowledge regarding the patient's culture
- ☒ Bias or stereotyping
- ☐ Other:

e. Please mark all of the challenges faced by immigrant and refugee populations when receiving healthcare that you have perceived or witnessed (may choose more than one):

- ☒ Language barriers
- ☒ Insurance barriers
- ☒ Cultural barriers
- ☒ Finding a professional interpreter
- ☒ Knowing how to work with a professional interpreter
- ☐ Time constraints
- ☒ Insufficiently trained health care providers
- ☒ Transportation problems for the patient
- ☒ Food insufficiency
- ☒ Need for child care
- ☒ Patients not understanding treatment plan
- ☒ Patients not following treatment plan
- ☒ My lack of knowledge regarding the patient's culture
- ☒ Bias or stereotyping
- ☒ Trust issues
- ☐ Other...

f. Rank how well immigrants and refugees understand the healthcare that you are trying to provide.

- ◯ Significantly less than a US born individual
- ⊗ Less than a US born individual
- ◯ Equivalent to a US born individual
- ◯ More than a US born patient
- ◯ Significantly more than a US born individual

g. Immigrants and refugees adhere to treatment plans and follow my recommendations.

- ◯ Never
- ◯ Rarely
- ⊗ Sometimes
- ◯ Usually
- ◯ Always

h. Immigrants and refugees should receive the same care and insurance coverage as US born patients.

- ◯ Never
- ◯ Rarely
- ◯ Sometimes
- ◯ Usually
- ⊗ Always

i. Immigrants and refugees who are undocumented should receive the same care and insurance coverage as US born patients.

- ◯ Never
- ◯ Rarely
- ◯ Sometimes
- ◯ Usually
- ⊗ Always

j. Every physician is professionally obligated to care for immigrants and refugees if they present to your clinic or hospital.

- ◯ Strongly disagree
- ◯ Disagree
- ◯ No opinion
- ◯ Agree
- ⊗ Strongly agree

k. Is healthcare a human right?
- ⊗ Yes
- ◯ No

B. If you wish, please tell us about what you enjoy or do not enjoy about immigrant and refugee health care and the greatest challenges you face in caring for this population.

____

SECTION D: DEMOGRAPHIC INFORMATION
Please answer the following questions by checking the box in front of the response choice that best describes you.

a. Your age?
- ◯ 20 to 24
- ⊗ 25 to 29
- ◯ 30 to 34
- ◯ 35 to 39
- ◯ 40 or older

b. Your gender?
- ⊗ Female
- ◯ Male
- ◯ Other

c.Are you Hispanic or Latino?
- ◯ Yes
- ⊗ No

d. What is your race? (Select one or more responses)
- ◯ American Indian or Alaska Native
- ◯ Asian (Please specify):
- ◯ Black or African American
- ◯ Native Hawaiian or Other Pacific Islander
- ⊗ White
- ◯ Other (Please specify):

e.Were you born in the United States?
- ⊗ Yes
- ◯ No

g. Your residency year?
- ⊗ PGY1
- ◯ PGY2
- ◯ PGY3
- ◯ PGY4
- ◯ PGY5

h. How would you classify your political ideology?
- ◯ Conservative
- ◯ Somewhat conservative
- ◯ Moderate
- ◯ Somewhat liberal
- ⊗ Liberal
- ◯ Other (Please specify):

i. Estimated level of educational debt?
- ◯ None
- ⊗ Less than $50,000
- ◯ $50,000 - $100,000
- ◯ $100,000 - $200,000
- ◯ $200,000 or more

j.Do you plan to subspecialize?
- ◯ Yes
- ⊗ No

k. Languages spoken?

- ☒ English
- ☒ Spanish
- ☐ French
- ☐ Hmong
- ☐ Somali
- ☐ Japanese
- ☐ Chinese
- ☐ Russian
- ☐ Ethiopian
- ☐ Other ___________

l. Are you in the Global Health Pathway?

- ⊗ Yes
- ◯ No

m. Did you earn your degree in the US?

- ⊗ Yes
- ◯ No

n. What residency program are you in?

- ◯ Internal Medicine
- ⊗ Med-Peds
- ◯ Pediatrics
- ◯ Family Practice
- ◯ Neurology
- ◯ Psychiatry
- ◯ ObGyn
- ◯ Neurosurgery
- ◯ General Surgery
- ◯ Orthopedic Surgery
- ◯ Urology
- ◯ Surgical sub-specialty (please specify in text box below)
- ◯ Non-clinical specialty (radiology, pathology; please specify in text box below)

Medical Trainees' attitudes, knowledge, and experience with immigrant and refugee health

Response was added on 11/01/2013 8:30pm.

SECTION A: Personal experience with immigrant and refugee health care.

A. Please indicate your level of agreement with the following statements regarding your personal experience with immigrant and refugee health care by checking the box that best represents your experience.

a. During my inpatient rotations, I take care of the following percentage of immigrant and refugee patients:
- ◯ None
- ◯ 0 -5%
- ◯ 5-10%
- ⊗ 10-25%
- ◯ > 25%

b. During my outpatient rotations, I take care of the following percentage of immigrant and refugee patients:
- ◯ None
- ◯ 0-10%
- ◯ 10 -25%
- ⊗ 25-50%
- ◯ 50-75%
- ◯ >75%

c. I would like to take care of more immigrant and refugee patients.
- ◯ Strongly disagree
- ◯ Disagree
- ◯ No opinion
- ⊗ Agree
- ◯ Strongly agree

d. I plan to take care of immigrants and refugees when I finish residency.
- ◯ Strongly disagree
- ◯ Disagree
- ◯ No opinion
- ⊗ Agree
- ◯ Strongly agree

e. I plan to do short term (< 6 months) international work when I finish residency.
- ◯ Strongly disagree
- ◯ Disagree
- ◯ No opinion
- ⊗ Agree
- ◯ Strongly agree

f. I plan to do long term (>6 months) international work when I finish residency.
- ◯ Strongly disagree
- ◯ Disagree
- ◯ No opinion
- ⊗ Agree
- ◯ Strongly agree

g. I plan to work in health disparities in the following way after residency:

Patient care, public health interventions

SECTION B: MEDICAL EDUCATION

A. Please indicate your level of agreement with the following statements regarding your medical education and knowledge about immigrants and refugees by checking the box that best represents your opinion.

a. I have received specialized training in immigrant and refugee health, tropical medicine, or cross-cultural health.

◯ Strongly disagree
◯ Disagree
◯ No opinion
⊗ Agree
◯ Strongly agree

b. If you have received specialized training in immigrant and refugee health, tropical medicine, or cross-cultural health, please indicate all the contexts in which you received this training:

☐ As an undergraduate.
☒ As a medical student.
☐ As part of my residency.
☐ A special program.
☐ As part of my fellowship.
☐ As part of a degree program (e.g. MPH)
☐ Other:

c. I feel comfortable with my fund of knowledge regarding immigrant and refugee health.

◯ Strongly disagree
⊗ Disagree
◯ No opinion
◯ Agree
◯ Strongly agree

d. I would like to have further training in immigrant and refugee health.

◯ Strongly disagree
◯ Disagree
◯ No opinion
◯ Agree
⊗ Strongly agree

e. If you agree with the above, please indicate all the contexts in which you would like to receive this training:
□□

☒ As part of my residency.
☒ A special program.
☐ As part of my fellowship.

SECTION C: Attitudes towards immigrant health

A.□Please indicate your level of agreement with the following statements regarding immigrant and refugee health by checking the box that best represents your opinion.

a. I enjoy taking care of immigrants and refugees.

◯ Never
◯ Rarely
◯ Sometimes
⊗ Usually
◯ Always

b. Please indicate the reasons that you enjoy taking care of immigrants and refugees (may choose more than one).

☒ Tropical and other conditions not frequently diagnosed in US-born patients
☒ Learning about other cultures
☐ They don't complain as much
☒ Being able to hear their stories
☐ Their care is more complicated
☐ Their care is less complicated
☐ They are very appreciative of your help.
☐ They are extremely vulnerable
☐ Other:

c. Taking care of immigrants and refugees is more challenging than taking care of US born patients.

◯ Never
◯ Rarely
⊗ Sometimes
◯ Usually
◯ Always

d. Please mark all the challenges that you face as a provider when providing care to immigrants and refugees (may choose more than one):

- ☒ Language barriers
- ☒ Insurance barriers
- ☒ Cultural barriers
- ☒ Finding a professional interpreter
- ☒ Knowing how to work with a professional interpreter
- ☒ Time constraints
- ☒ My own knowledge related to tropical and travel medicine
- ☒ Transportation problems for the patient
- ☒ Patients not understanding treatment plan
- ☒ Patients not following treatment plan
- ☒ My lack of knowledge regarding the patient's culture
- ☒ Bias or stereotyping
- ☐ Other:

e. Please mark all of the challenges faced by immigrant and refugee populations when receiving healthcare that you have perceived or witnessed (may choose more than one):

- ☒ Language barriers
- ☒ Insurance barriers
- ☒ Cultural barriers
- ☒ Finding a professional interpreter
- ☒ Knowing how to work with a professional interpreter
- ☒ Time constraints
- ☒ Insufficiently trained health care providers
- ☒ Transportation problems for the patient
- ☒ Food insufficiency
- ☒ Need for child care
- ☒ Patients not understanding treatment plan
- ☒ Patients not following treatment plan
- ☒ My lack of knowledge regarding the patient's culture
- ☒ Bias or stereotyping
- ☒ Trust issues
- ☐ Other...

f. Rank how well immigrants and refugees understand the healthcare that you are trying to provide.

- ◯ Significantly less than a US born individual
- ◯ Less than a US born individual
- ◯ Equivalent to a US born individual
- ◯ More than a US born patient
- ◯ Significantly more than a US born individual

g. Immigrants and refugees adhere to treatment plans and follow my recommendations.

- ◯ Never
- ◯ Rarely
- ◯ Sometimes
- ⊗ Usually
- ◯ Always

h. Immigrants and refugees should receive the same care and insurance coverage as US born patients.

- ◯ Never
- ◯ Rarely
- ◯ Sometimes
- ◯ Usually
- ⊗ Always

i. Immigrants and refugees who are undocumented should receive the same care and insurance coverage as US born patients.

- ◯ Never
- ◯ Rarely
- ◯ Sometimes
- ⊗ Usually
- ◯ Always

j. Every physician is professionally obligated to care for immigrants and refugees if they present to your clinic or hospital.

- ◯ Strongly disagree
- ◯ Disagree
- ◯ No opinion
- ◯ Agree
- ⊗ Strongly agree

k. Is healthcare a human right?

- ◯ Yes
- ◯ No

B. If you wish, please tell us about what you enjoy or do not enjoy about immigrant and refugee health care and the greatest challenges you face in caring for this population.

____

SECTION D: DEMOGRAPHIC INFORMATION
Please answer the following questions by checking the box in front of the response choice that best describes you.

a. Your age?

- ◯ 20 to 24
- ◯ 25 to 29
- ⊗ 30 to 34
- ◯ 35 to 39
- ◯ 40 or older

b. Your gender?

- ⊗ Female
- ◯ Male
- ◯ Other

c. Are you Hispanic or Latino?

- ◯ Yes
- ⊗ No

d. What is your race? (Select one or more responses)

- ◯ American Indian or Alaska Native
- ◯ Asian (Please specify):
- ◯ Black or African American
- ◯ Native Hawaiian or Other Pacific Islander
- ◯ White
- ◯ Other (Please specify):

e. Were you born in the United States?

- ⊗ Yes
- ◯ No

g. Your residency year?

- ◯ PGY1
- ⊗ PGY2
- ◯ PGY3
- ◯ PGY4
- ◯ PGY5

h. How would you classify your political ideology?

- ◯ Conservative
- ◯ Somewhat conservative
- ◯ Moderate
- ◯ Somewhat liberal
- ⊗ Liberal
- ◯ Other (Please specify):

i. Estimated level of educational debt?

- ◯ None
- ◯ Less than $50,000
- ◯ $50,000 - $100,000
- ⊗ $100,000 - $200,000
- ◯ $200,000 or more

j. Do you plan to subspecialize?

- ◯ Yes
- ⊗ No

k. Languages spoken?

- ☒ English
- ☒ Spanish
- ☒ French
- ☐ Hmong
- ☐ Somali
- ☐ Japanese
- ☐ Chinese
- ☐ Russian
- ☐ Ethiopian
- ☐ Other ___________

l. Are you in the Global Health Pathway?

- ○ Yes
- ⊗ No

m. Did you earn your degree in the US?

- ⊗ Yes
- ○ No

n. What residency program are you in?

- ⊗ Internal Medicine
- ○ Med-Peds
- ○ Pediatrics
- ○ Family Practice
- ○ Neurology
- ○ Psychiatry
- ○ ObGyn
- ○ Neurosurgery
- ○ General Surgery
- ○ Orthopedic Surgery
- ○ Urology
- ○ Surgical sub-specialty (please specify in text box below)
- ○ Non-clinical specialty (radiology, pathology; please specify in text box below)

Medical Trainees' attitudes, knowledge, and experience with immigrant and refugee health

Response was added on 11/01/2013 8:36pm.

SECTION A: Personal experience with immigrant and refugee health care.

A. Please indicate your level of agreement with the following statements regarding your personal experience with immigrant and refugee health care by checking the box that best represents your experience.

a. During my inpatient rotations, I take care of the following percentage of immigrant and refugee patients:
- ◯ None
- ◯ 0 -5%
- ◯ 5-10%
- ⊗ 10-25%
- ◯ > 25%

b. During my outpatient rotations, I take care of the following percentage of immigrant and refugee patients:
- ⊗ None
- ◯ 0-10%
- ◯ 10 -25%
- ◯ 25-50%
- ◯ 50-75%
- ◯ >75%

c. I would like to take care of more immigrant and refugee patients.
- ⊗ Strongly disagree
- ◯ Disagree
- ◯ No opinion
- ◯ Agree
- ◯ Strongly agree

d. I plan to take care of immigrants and refugees when I finish residency.
- ⊗ Strongly disagree
- ◯ Disagree
- ◯ No opinion
- ◯ Agree
- ◯ Strongly agree

e. I plan to do short term (< 6 months) international work when I finish residency.
- ⊗ Strongly disagree
- ◯ Disagree
- ◯ No opinion
- ◯ Agree
- ◯ Strongly agree

f. I plan to do long term (>6 months) international work when I finish residency.
- ⊗ Strongly disagree
- ◯ Disagree
- ◯ No opinion
- ◯ Agree
- ◯ Strongly agree

g. I plan to work in health disparities in the following way after residency:

Low income and under insured does not only mean immigrant population. I'm not sure my practice plans after residency and fellowship, but it will not involve global health.

SECTION B: MEDICAL EDUCATION

A. Please indicate your level of agreement with the following statements regarding your medical education and knowledge about immigrants and refugees by checking the box that best represents your opinion.

a. I have received specialized training in immigrant and refugee health, tropical medicine, or cross-cultural health.

- ◯ Strongly disagree
- ⊗ Disagree
- ◯ No opinion
- ◯ Agree
- ◯ Strongly agree

c. I feel comfortable with my fund of knowledge regarding immigrant and refugee health.

- ◯ Strongly disagree
- ◯ Disagree
- ◯ No opinion
- ⊗ Agree
- ◯ Strongly agree

d. I would like to have further training in immigrant and refugee health.

- ⊗ Strongly disagree
- ◯ Disagree
- ◯ No opinion
- ◯ Agree
- ◯ Strongly agree

SECTION C: Attitudes towards immigrant health

A.Please indicate your level of agreement with the following statements regarding immigrant and refugee health by checking the box that best represents your opinion.

a. I enjoy taking care of immigrants and refugees.

- ◯ Never
- ⊗ Rarely
- ◯ Sometimes
- ◯ Usually
- ◯ Always

b. Please indicate the reasons that you enjoy taking care of immigrants and refugees (may choose more than one).

- ☐ Tropical and other conditions not frequently diagnosed in US-born patients
- ☐ Learning about other cultures
- ☐ They don't complain as much
- ☐ Being able to hear their stories
- ☐ Their care is more complicated
- ☐ Their care is less complicated
- ☐ They are very appreciative of your help.
- ☐ They are extremely vulnerable
- ☐ Other:

c. Taking care of immigrants and refugees is more challenging than taking care of US born patients.

- ◯ Never
- ◯ Rarely
- ◯ Sometimes
- ⊗ Usually
- ◯ Always

d. Please mark all the challenges that you face as a provider when providing care to immigrants and refugees (may choose more than one):

- ☒ Language barriers
- ☐ Insurance barriers
- ☒ Cultural barriers
- ☒ Finding a professional interpreter
- ☐ Knowing how to work with a professional interpreter
- ☒ Time constraints
- ☒ My own knowledge related to tropical and travel medicine
- ☐ Transportation problems for the patient
- ☒ Patients not understanding treatment plan
- ☒ Patients not following treatment plan
- ☒ My lack of knowledge regarding the patient's culture
- ☒ Bias or stereotyping
- ☐ Other:

e. Please mark all of the challenges faced by immigrant and refugee populations when receiving healthcare that you have perceived or witnessed (may choose more than one):

- ☒ Language barriers
- ☐ Insurance barriers
- ☒ Cultural barriers
- ☐ Finding a professional interpreter
- ☐ Knowing how to work with a professional interpreter
- ☐ Time constraints
- ☐ Insufficiently trained health care providers
- ☐ Transportation problems for the patient
- ☐ Food insufficiency
- ☐ Need for child care
- ☒ Patients not understanding treatment plan
- ☐ Patients not following treatment plan
- ☐ My lack of knowledge regarding the patient's culture
- ☐ Bias or stereotyping
- ☒ Trust issues
- ☐ Other...

f. Rank how well immigrants and refugees understand the healthcare that you are trying to provide.

- ○ Significantly less than a US born individual
- ○ Less than a US born individual
- ⊗ Equivalent to a US born individual
- ○ More than a US born patient
- ○ Significantly more than a US born individual

g. Immigrants and refugees adhere to treatment plans and follow my recommendations.

- ○ Never
- ○ Rarely
- ⊗ Sometimes
- ○ Usually
- ○ Always

h. Immigrants and refugees should receive the same care and insurance coverage as US born patients.

- ○ Never
- ○ Rarely
- ⊗ Sometimes
- ○ Usually
- ○ Always

i. Immigrants and refugees who are undocumented should receive the same care and insurance coverage as US born patients.

- ○ Never
- ○ Rarely
- ○ Sometimes
- ⊗ Usually
- ○ Always

j. Every physician is professionally obligated to care for immigrants and refugees if they present to your clinic or hospital.

- ○ Strongly disagree
- ○ Disagree
- ○ No opinion
- ⊗ Agree
- ○ Strongly agree

k. Is healthcare a human right?
- ⊗ Yes
- ◯ No

B. If you wish, please tell us about what you enjoy or do not enjoy about immigrant and refugee health care and the greatest challenges you face in caring for this population.
____

SECTION D: DEMOGRAPHIC INFORMATION
Please answer the following questions by checking the box in front of the response choice that best describes you.

a. Your age?
- ◯ 20 to 24
- ⊗ 25 to 29
- ◯ 30 to 34
- ◯ 35 to 39
- ◯ 40 or older

b. Your gender?
- ⊗ Female
- ◯ Male
- ◯ Other

c. Are you Hispanic or Latino?
- ◯ Yes
- ⊗ No

d. What is your race? (Select one or more responses)
- ◯ American Indian or Alaska Native
- ◯ Asian (Please specify):
- ◯ Black or African American
- ◯ Native Hawaiian or Other Pacific Islander
- ⊗ White
- ◯ Other (Please specify):

e. Were you born in the United States?
- ⊗ Yes
- ◯ No

g. Your residency year?
- ⊗ PGY1
- ◯ PGY2
- ◯ PGY3
- ◯ PGY4
- ◯ PGY5

h. How would you classify your political ideology?
- ◯ Conservative
- ◯ Somewhat conservative
- ◯ Moderate
- ◯ Somewhat liberal
- ⊗ Liberal
- ◯ Other (Please specify):

i. Estimated level of educational debt?
- ◯ None
- ◯ Less than $50,000
- ◯ $50,000 - $100,000
- ◯ $100,000 - $200,000
- ⊗ $200,000 or more

j. Do you plan to subspecialize?
- ⊗ Yes
- ◯ No

k. Languages spoken?

- ☒ English
- ☐ Spanish
- ☐ French
- ☐ Hmong
- ☐ Somali
- ☐ Japanese
- ☐ Chinese
- ☐ Russian
- ☐ Ethiopian
- ☐ Other ___________

l. Are you in the Global Health Pathway?

- ◯ Yes
- ⊗ No

m. Did you earn your degree in the US?

- ⊗ Yes
- ◯ No

n. What residency program are you in?

- ⊗ Internal Medicine
- ◯ Med-Peds
- ◯ Pediatrics
- ◯ Family Practice
- ◯ Neurology
- ◯ Psychiatry
- ◯ ObGyn
- ◯ Neurosurgery
- ◯ General Surgery
- ◯ Orthopedic Surgery
- ◯ Urology
- ◯ Surgical sub-specialty (please specify in text box below)
- ◯ Non-clinical specialty (radiology, pathology; please specify in text box below)

Medical Trainees' attitudes, knowledge, and experience with immigrant and refugee health

Response was added on 11/01/2013 9:05pm.

SECTION A: Personal experience with immigrant and refugee health care.

A. Please indicate your level of agreement with the following statements regarding your personal experience with immigrant and refugee health care by checking the box that best represents your experience.

a. During my inpatient rotations, I take care of the following percentage of immigrant and refugee patients:

- ◯ None
- ◯ 0 -5%
- ⊗ 5-10%
- ◯ 10-25%
- ◯ > 25%

b. During my outpatient rotations, I take care of the following percentage of immigrant and refugee patients:

- ◯ None
- ◯ 0-10%
- ⊗ 10 -25%
- ◯ 25-50%
- ◯ 50-75%
- ◯ >75%

c. I would like to take care of more immigrant and refugee patients.

- ◯ Strongly disagree
- ◯ Disagree
- ◯ No opinion
- ⊗ Agree
- ◯ Strongly agree

d. I plan to take care of immigrants and refugees when I finish residency.

- ◯ Strongly disagree
- ◯ Disagree
- ◯ No opinion
- ◯ Agree
- ⊗ Strongly agree

e. I plan to do short term (< 6 months) international work when I finish residency.

- ◯ Strongly disagree
- ◯ Disagree
- ⊗ No opinion
- ◯ Agree
- ◯ Strongly agree

f. I plan to do long term (>6 months) international work when I finish residency.

- ◯ Strongly disagree
- ⊗ Disagree
- ◯ No opinion
- ◯ Agree
- ◯ Strongly agree

g. I plan to work in health disparities in the following way after residency:

Hopefully community health center

SECTION B: MEDICAL EDUCATION

A. Please indicate your level of agreement with the following statements regarding your medical education and knowledge about immigrants and refugees by checking the box that best represents your opinion.

a. I have received specialized training in immigrant and refugee health, tropical medicine, or cross-cultural health.

- ◯ Strongly disagree
- ◯ Disagree
- ◯ No opinion
- ⊗ Agree
- ◯ Strongly agree

b. If you have received specialized training in immigrant and refugee health, tropical medicine, or cross-cultural health, please indicate all the contexts in which you received this training:

- ☐ As an undergraduate.
- ☒ As a medical student.
- ☒ As part of my residency.
- ☐ A special program.
- ☐ As part of my fellowship.
- ☐ As part of a degree program (e.g. MPH)
- ☐ Other:

c. I feel comfortable with my fund of knowledge regarding immigrant and refugee health.

- ◯ Strongly disagree
- ⊗ Disagree
- ◯ No opinion
- ◯ Agree
- ◯ Strongly agree

d. I would like to have further training in immigrant and refugee health.

- ◯ Strongly disagree
- ◯ Disagree
- ◯ No opinion
- ⊗ Agree
- ◯ Strongly agree

e. If you agree with the above, please indicate all the contexts in which you would like to receive this training:

- ☐ As part of my residency.
- ☒ A special program.
- ☐ As part of my fellowship.

SECTION C: Attitudes towards immigrant health

A. Please indicate your level of agreement with the following statements regarding immigrant and refugee health by checking the box that best represents your opinion.

a. I enjoy taking care of immigrants and refugees.

- ◯ Never
- ◯ Rarely
- ◯ Sometimes
- ⊗ Usually
- ◯ Always

b. Please indicate the reasons that you enjoy taking care of immigrants and refugees (may choose more than one).

- ☒ Tropical and other conditions not frequently diagnosed in US-born patients
- ☒ Learning about other cultures
- ☒ They don't complain as much
- ☐ Being able to hear their stories
- ☐ Their care is more complicated
- ☐ Their care is less complicated
- ☒ They are very appreciative of your help.
- ☐ They are extremely vulnerable
- ☐ Other:

c. Taking care of immigrants and refugees is more challenging than taking care of US born patients.

- ◯ Never
- ⊗ Rarely
- ◯ Sometimes
- ◯ Usually
- ◯ Always

d. Please mark all the challenges that you face as a provider when providing care to immigrants and refugees (may choose more than one):

- ☒ Language barriers
- ☐ Insurance barriers
- ☒ Cultural barriers
- ☒ Finding a professional interpreter
- ☐ Knowing how to work with a professional interpreter
- ☒ Time constraints
- ☐ My own knowledge related to tropical and travel medicine
- ☐ Transportation problems for the patient
- ☒ Patients not understanding treatment plan
- ☒ Patients not following treatment plan
- ☒ My lack of knowledge regarding the patient's culture
- ☒ Bias or stereotyping
- ☐ Other:

e. Please mark all of the challenges faced by immigrant and refugee populations when receiving healthcare that you have perceived or witnessed (may choose more than one):

- ☒ Language barriers
- ☐ Insurance barriers
- ☒ Cultural barriers
- ☒ Finding a professional interpreter
- ☐ Knowing how to work with a professional interpreter
- ☒ Time constraints
- ☒ Insufficiently trained health care providers
- ☐ Transportation problems for the patient
- ☐ Food insufficiency
- ☐ Need for child care
- ☒ Patients not understanding treatment plan
- ☒ Patients not following treatment plan
- ☒ My lack of knowledge regarding the patient's culture
- ☒ Bias or stereotyping
- ☒ Trust issues
- ☐ Other...

f. Rank how well immigrants and refugees understand the healthcare that you are trying to provide.

- ○ Significantly less than a US born individual
- ⊗ Less than a US born individual
- ○ Equivalent to a US born individual
- ○ More than a US born patient
- ○ Significantly more than a US born individual

g. Immigrants and refugees adhere to treatment plans and follow my recommendations.

- ○ Never
- ○ Rarely
- ⊗ Sometimes
- ○ Usually
- ○ Always

h. Immigrants and refugees should receive the same care and insurance coverage as US born patients.

- ○ Never
- ○ Rarely
- ○ Sometimes
- ○ Usually
- ⊗ Always

i. Immigrants and refugees who are undocumented should receive the same care and insurance coverage as US born patients.

- ○ Never
- ○ Rarely
- ○ Sometimes
- ○ Usually
- ⊗ Always

j. Every physician is professionally obligated to care for immigrants and refugees if they present to your clinic or hospital.

- ○ Strongly disagree
- ○ Disagree
- ○ No opinion
- ○ Agree
- ⊗ Strongly agree

k. Is healthcare a human right?
- ⊗ Yes
- ◯ No

B. If you wish, please tell us about what you enjoy or do not enjoy about immigrant and refugee health care and the greatest challenges you face in caring for this population.

Interpreter = time

SECTION D: DEMOGRAPHIC INFORMATION
Please answer the following questions by checking the box in front of the response choice that best describes you.

a. Your age?
- ◯ 20 to 24
- ⊗ 25 to 29
- ◯ 30 to 34
- ◯ 35 to 39
- ◯ 40 or older

b. Your gender?
- ◯ Female
- ⊗ Male
- ◯ Other

c.Are you Hispanic or Latino?
- ◯ Yes
- ⊗ No

d. What is your race? (Select one or more responses)
- ◯ American Indian or Alaska Native
- ◯ Asian (Please specify):
- ◯ Black or African American
- ◯ Native Hawaiian or Other Pacific Islander
- ⊗ White
- ◯ Other (Please specify):

e.Were you born in the United States?
- ⊗ Yes
- ◯ No

g. Your residency year?
- ◯ PGY1
- ⊗ PGY2
- ◯ PGY3
- ◯ PGY4
- ◯ PGY5

h. How would you classify your political ideology?
- ◯ Conservative
- ◯ Somewhat conservative
- ◯ Moderate
- ◯ Somewhat liberal
- ⊗ Liberal
- ◯ Other (Please specify):

i. Estimated level of educational debt?
- ◯ None
- ◯ Less than $50,000
- ◯ $50,000 - $100,000
- ⊗ $100,000 - $200,000
- ◯ $200,000 or more

j.Do you plan to subspecialize?
- ◯ Yes
- ⊗ No

k. Languages spoken?

- ☒ English
- ☐ Spanish
- ☐ French
- ☐ Hmong
- ☐ Somali
- ☐ Japanese
- ☐ Chinese
- ☐ Russian
- ☐ Ethiopian
- ☐ Other ___________

l. Are you in the Global Health Pathway?

- ◯ Yes
- ⊗ No

m. Did you earn your degree in the US?

- ⊗ Yes
- ◯ No

n. What residency program are you in?

- ◯ Internal Medicine
- ◯ Med-Peds
- ◯ Pediatrics
- ⊗ Family Practice
- ◯ Neurology
- ◯ Psychiatry
- ◯ ObGyn
- ◯ Neurosurgery
- ◯ General Surgery
- ◯ Orthopedic Surgery
- ◯ Urology
- ◯ Surgical sub-specialty (please specify in text box below)
- ◯ Non-clinical specialty (radiology, pathology; please specify in text box below)

Medical Trainees' attitudes, knowledge, and experience with immigrant and refugee health

Response was added on 11/01/2013 9:13pm.

SECTION A: Personal experience with immigrant and refugee health care.

A. Please indicate your level of agreement with the following statements regarding your personal experience with immigrant and refugee health care by checking the box that best represents your experience.

a. During my inpatient rotations, I take care of the following percentage of immigrant and refugee patients:

- ◯ None
- ⊗ 0 -5%
- ◯ 5-10%
- ◯ 10-25%
- ◯ > 25%

b. During my outpatient rotations, I take care of the following percentage of immigrant and refugee patients:

- ◯ None
- ◯ 0-10%
- ⊗ 10 -25%
- ◯ 25-50%
- ◯ 50-75%
- ◯ >75%

c. I would like to take care of more immigrant and refugee patients.

- ◯ Strongly disagree
- ◯ Disagree
- ⊗ No opinion
- ◯ Agree
- ◯ Strongly agree

d. I plan to take care of immigrants and refugees when I finish residency.

- ◯ Strongly disagree
- ◯ Disagree
- ◯ No opinion
- ⊗ Agree
- ◯ Strongly agree

e. I plan to do short term (< 6 months) international work when I finish residency.

- ◯ Strongly disagree
- ⊗ Disagree
- ◯ No opinion
- ◯ Agree
- ◯ Strongly agree

f. I plan to do long term (>6 months) international work when I finish residency.

- ◯ Strongly disagree
- ⊗ Disagree
- ◯ No opinion
- ◯ Agree
- ◯ Strongly agree

g. I plan to work in health disparities in the following way after residency:

Diverse patient population served in clinic. Possible international work as well.

SECTION B: MEDICAL EDUCATION

A. Please indicate your level of agreement with the following statements regarding your medical education and knowledge about immigrants and refugees by checking the box that best represents your opinion.

a. I have received specialized training in immigrant and refugee health, tropical medicine, or cross-cultural health.

◯ Strongly disagree
◯ Disagree
◯ No opinion
◯ Agree
⊗ Strongly agree

b. If you have received specialized training in immigrant and refugee health, tropical medicine, or cross-cultural health, please indicate all the contexts in which you received this training:

☐ As an undergraduate.
☒ As a medical student.
☒ As part of my residency.
☒ A special program.
☐ As part of my fellowship.
☐ As part of a degree program (e.g. MPH)
☐ Other:

c. I feel comfortable with my fund of knowledge regarding immigrant and refugee health.

◯ Strongly disagree
◯ Disagree
◯ No opinion
⊗ Agree
◯ Strongly agree

d. I would like to have further training in immigrant and refugee health.

◯ Strongly disagree
◯ Disagree
◯ No opinion
⊗ Agree
◯ Strongly agree

e. If you agree with the above, please indicate all the contexts in which you would like to receive this training:
□□

☒ As part of my residency.
☒ A special program.
☐ As part of my fellowship.

SECTION C: Attitudes towards immigrant health

A.□Please indicate your level of agreement with the following statements regarding immigrant and refugee health by checking the box that best represents your opinion.

a. I enjoy taking care of immigrants and refugees.

◯ Never
◯ Rarely
◯ Sometimes
⊗ Usually
◯ Always

b. Please indicate the reasons that you enjoy taking care of immigrants and refugees (may choose more than one).

☒ Tropical and other conditions not frequently diagnosed in US-born patients
☒ Learning about other cultures
☐ They don't complain as much
☒ Being able to hear their stories
☐ Their care is more complicated
☐ Their care is less complicated
☒ They are very appreciative of your help.
☐ They are extremely vulnerable
☐ Other:

c. Taking care of immigrants and refugees is more challenging than taking care of US born patients.

◯ Never
◯ Rarely
◯ Sometimes
⊗ Usually
◯ Always

d. Please mark all the challenges that you face as a provider when providing care to immigrants and refugees (may choose more than one):

- ☒ Language barriers
- ☐ Insurance barriers
- ☒ Cultural barriers
- ☒ Finding a professional interpreter
- ☐ Knowing how to work with a professional interpreter
- ☒ Time constraints
- ☒ My own knowledge related to tropical and travel medicine
- ☒ Transportation problems for the patient
- ☒ Patients not understanding treatment plan
- ☒ Patients not following treatment plan
- ☒ My lack of knowledge regarding the patient's culture
- ☒ Bias or stereotyping
- ☐ Other:

e. Please mark all of the challenges faced by immigrant and refugee populations when receiving healthcare that you have perceived or witnessed (may choose more than one):

- ☒ Language barriers
- ☒ Insurance barriers
- ☒ Cultural barriers
- ☒ Finding a professional interpreter
- ☒ Knowing how to work with a professional interpreter
- ☒ Time constraints
- ☐ Insufficiently trained health care providers
- ☒ Transportation problems for the patient
- ☐ Food insufficiency
- ☒ Need for child care
- ☒ Patients not understanding treatment plan
- ☒ Patients not following treatment plan
- ☒ My lack of knowledge regarding the patient's culture
- ☒ Bias or stereotyping
- ☒ Trust issues
- ☐ Other...

f. Rank how well immigrants and refugees understand the healthcare that you are trying to provide.

- ○ Significantly less than a US born individual
- ○ Less than a US born individual
- ○ Equivalent to a US born individual
- ○ More than a US born patient
- ○ Significantly more than a US born individual

g. Immigrants and refugees adhere to treatment plans and follow my recommendations.

- ○ Never
- ○ Rarely
- ⊗ Sometimes
- ○ Usually
- ○ Always

h. Immigrants and refugees should receive the same care and insurance coverage as US born patients.

- ○ Never
- ○ Rarely
- ○ Sometimes
- ⊗ Usually
- ○ Always

i. Immigrants and refugees who are undocumented should receive the same care and insurance coverage as US born patients.

- ○ Never
- ⊗ Rarely
- ○ Sometimes
- ○ Usually
- ○ Always

j. Every physician is professionally obligated to care for immigrants and refugees if they present to your clinic or hospital.

- ○ Strongly disagree
- ○ Disagree
- ○ No opinion
- ⊗ Agree
- ○ Strongly agree

k. Is healthcare a human right?

◯ Yes
⊗ No

B. If you wish, please tell us about what you enjoy or do not enjoy about immigrant and refugee health care and the greatest challenges you face in caring for this population.

____

SECTION D: DEMOGRAPHIC INFORMATION
Please answer the following questions by checking the box in front of the response choice that best describes you.

a. Your age?

◯ 20 to 24
⊗ 25 to 29
◯ 30 to 34
◯ 35 to 39
◯ 40 or older

b. Your gender?

⊗ Female
◯ Male
◯ Other

c.Are you Hispanic or Latino?

◯ Yes
⊗ No

d. What is your race? (Select one or more responses)

◯ American Indian or Alaska Native
◯ Asian (Please specify):
◯ Black or African American
◯ Native Hawaiian or Other Pacific Islander
⊗ White
◯ Other (Please specify):

e.Were you born in the United States?

⊗ Yes
◯ No

g. Your residency year?

⊗ PGY1
◯ PGY2
◯ PGY3
◯ PGY4
◯ PGY5

h. How would you classify your political ideology?

◯ Conservative
◯ Somewhat conservative
◯ Moderate
⊗ Somewhat liberal
◯ Liberal
◯ Other (Please specify):

i. Estimated level of educational debt?

◯ None
⊗ Less than $50,000
◯ $50,000 - $100,000
◯ $100,000 - $200,000
◯ $200,000 or more

j.Do you plan to subspecialize?

◯ Yes
⊗ No

k. Languages spoken?

- ☒ English
- ☒ Spanish
- ☐ French
- ☐ Hmong
- ☐ Somali
- ☐ Japanese
- ☐ Chinese
- ☐ Russian
- ☐ Ethiopian
- ☐ Other ___________

l. Are you in the Global Health Pathway?

- ⊗ Yes
- ◯ No

m. Did you earn your degree in the US?

- ⊗ Yes
- ◯ No

n. What residency program are you in?

- ◯ Internal Medicine
- ◯ Med-Peds
- ⊗ Pediatrics
- ◯ Family Practice
- ◯ Neurology
- ◯ Psychiatry
- ◯ ObGyn
- ◯ Neurosurgery
- ◯ General Surgery
- ◯ Orthopedic Surgery
- ◯ Urology
- ◯ Surgical sub-specialty (please specify in text box below)
- ◯ Non-clinical specialty (radiology, pathology; please specify in text box below)

Medical Trainees' attitudes, knowledge, and experience with immigrant and refugee health

Response was added on 11/01/2013 9:17pm.

SECTION A: Personal experience with immigrant and refugee health care.

A. Please indicate your level of agreement with the following statements regarding your personal experience with immigrant and refugee health care by checking the box that best represents your experience.

a. During my inpatient rotations, I take care of the following percentage of immigrant and refugee patients:

- ◯ None
- ◯ 0 -5%
- ◯ 5-10%
- ⊗ 10-25%
- ◯ > 25%

b. During my outpatient rotations, I take care of the following percentage of immigrant and refugee patients:

- ◯ None
- ◯ 0-10%
- ◯ 10 -25%
- ⊗ 25-50%
- ◯ 50-75%
- ◯ >75%

c. I would like to take care of more immigrant and refugee patients.

- ◯ Strongly disagree
- ◯ Disagree
- ⊗ No opinion
- ◯ Agree
- ◯ Strongly agree

d. I plan to take care of immigrants and refugees when I finish residency.

- ◯ Strongly disagree
- ◯ Disagree
- ◯ No opinion
- ⊗ Agree
- ◯ Strongly agree

e. I plan to do short term (< 6 months) international work when I finish residency.

- ◯ Strongly disagree
- ⊗ Disagree
- ◯ No opinion
- ◯ Agree
- ◯ Strongly agree

f. I plan to do long term (>6 months) international work when I finish residency.

- ◯ Strongly disagree
- ⊗ Disagree
- ◯ No opinion
- ◯ Agree
- ◯ Strongly agree

g. I plan to work in health disparities in the following way after residency:

n/a

SECTION B: MEDICAL EDUCATION

A. Please indicate your level of agreement with the following statements regarding your medical education and knowledge about immigrants and refugees by checking the box that best represents your opinion.

a. I have received specialized training in immigrant and refugee health, tropical medicine, or cross-cultural health.

- ◯ Strongly disagree
- ⊗ Disagree
- ◯ No opinion
- ◯ Agree
- ◯ Strongly agree

c. I feel comfortable with my fund of knowledge regarding immigrant and refugee health.

- ◯ Strongly disagree
- ⊗ Disagree
- ◯ No opinion
- ◯ Agree
- ◯ Strongly agree

d. I would like to have further training in immigrant and refugee health.

- ◯ Strongly disagree
- ◯ Disagree
- ⊗ No opinion
- ◯ Agree
- ◯ Strongly agree

SECTION C: Attitudes towards immigrant health

A.Please indicate your level of agreement with the following statements regarding immigrant and refugee health by checking the box that best represents your opinion.

a. I enjoy taking care of immigrants and refugees.

- ◯ Never
- ◯ Rarely
- ⊗ Sometimes
- ◯ Usually
- ◯ Always

b. Please indicate the reasons that you enjoy taking care of immigrants and refugees (may choose more than one).

- ☐ Tropical and other conditions not frequently diagnosed in US-born patients
- ☒ Learning about other cultures
- ☒ They don't complain as much
- ☒ Being able to hear their stories
- ☐ Their care is more complicated
- ☐ Their care is less complicated
- ☒ They are very appreciative of your help.
- ☐ They are extremely vulnerable
- ☐ Other:

c. Taking care of immigrants and refugees is more challenging than taking care of US born patients.

- ◯ Never
- ◯ Rarely
- ◯ Sometimes
- ⊗ Usually
- ◯ Always

d. Please mark all the challenges that you face as a provider when providing care to immigrants and refugees (may choose more than one):

- ☒ Language barriers
- ☐ Insurance barriers
- ☒ Cultural barriers
- ☒ Finding a professional interpreter
- ☐ Knowing how to work with a professional interpreter
- ☒ Time constraints
- ☐ My own knowledge related to tropical and travel medicine
- ☐ Transportation problems for the patient
- ☒ Patients not understanding treatment plan
- ☒ Patients not following treatment plan
- ☒ My lack of knowledge regarding the patient's culture
- ☒ Bias or stereotyping
- ☐ Other:

e. Please mark all of the challenges faced by immigrant and refugee populations when receiving healthcare that you have perceived or witnessed (may choose more than one):

- ☒ Language barriers
- ☐ Insurance barriers
- ☒ Cultural barriers
- ☒ Finding a professional interpreter
- ☒ Knowing how to work with a professional interpreter
- ☐ Time constraints
- ☐ Insufficiently trained health care providers
- ☐ Transportation problems for the patient
- ☐ Food insufficiency
- ☐ Need for child care
- ☒ Patients not understanding treatment plan
- ☒ Patients not following treatment plan
- ☒ My lack of knowledge regarding the patient's culture
- ☐ Bias or stereotyping
- ☒ Trust issues
- ☐ Other...

f. Rank how well immigrants and refugees understand the healthcare that you are trying to provide.

- ◯ Significantly less than a US born individual
- ⊗ Less than a US born individual
- ◯ Equivalent to a US born individual
- ◯ More than a US born patient
- ◯ Significantly more than a US born individual

g. Immigrants and refugees adhere to treatment plans and follow my recommendations.

- ◯ Never
- ◯ Rarely
- ⊗ Sometimes
- ◯ Usually
- ◯ Always

h. Immigrants and refugees should receive the same care and insurance coverage as US born patients.

- ◯ Never
- ◯ Rarely
- ◯ Sometimes
- ◯ Usually
- ⊗ Always

i. Immigrants and refugees who are undocumented should receive the same care and insurance coverage as US born patients.

- ◯ Never
- ◯ Rarely
- ◯ Sometimes
- ◯ Usually
- ⊗ Always

j. Every physician is professionally obligated to care for immigrants and refugees if they present to your clinic or hospital.

- ◯ Strongly disagree
- ◯ Disagree
- ◯ No opinion
- ◯ Agree
- ⊗ Strongly agree

k. Is healthcare a human right?

- ⊗ Yes
- ◯ No

B. If you wish, please tell us about what you enjoy or do not enjoy about immigrant and refugee health care and the greatest challenges you face in caring for this population.

____

SECTION D: DEMOGRAPHIC INFORMATION
Please answer the following questions by checking the box in front of the response choice that best describes you.

a. Your age?
- ◯ 20 to 24
- ⊗ 25 to 29
- ◯ 30 to 34
- ◯ 35 to 39
- ◯ 40 or older

b. Your gender?
- ◯ Female
- ⊗ Male
- ◯ Other

c. Are you Hispanic or Latino?
- ◯ Yes
- ⊗ No

d. What is your race? (Select one or more responses)
- ◯ American Indian or Alaska Native
- ◯ Asian (Please specify):
- ◯ Black or African American
- ◯ Native Hawaiian or Other Pacific Islander
- ⊗ White
- ◯ Other (Please specify):

e. Were you born in the United States?
- ⊗ Yes
- ◯ No

g. Your residency year?
- ⊗ PGY1
- ◯ PGY2
- ◯ PGY3
- ◯ PGY4
- ◯ PGY5

h. How would you classify your political ideology?
- ◯ Conservative
- ◯ Somewhat conservative
- ◯ Moderate
- ◯ Somewhat liberal
- ⊗ Liberal
- ◯ Other (Please specify):

i. Estimated level of educational debt?
- ◯ None
- ◯ Less than $50,000
- ◯ $50,000 - $100,000
- ⊗ $100,000 - $200,000
- ◯ $200,000 or more

j. Do you plan to subspecialize?
- ◯ Yes
- ⊗ No

k. Languages spoken?
- ☒ English
- ☐ Spanish
- ☐ French
- ☐ Hmong
- ☐ Somali
- ☐ Japanese
- ☐ Chinese
- ☐ Russian
- ☐ Ethiopian
- ☐ Other ___________

l. Are you in the Global Health Pathway?
- ◯ Yes
- ⊗ No

m. Did you earn your degree in the US?

- ⊗ Yes
- ◯ No

n. What residency program are you in?

- ◯ Internal Medicine
- ◯ Med-Peds
- ◯ Pediatrics
- ⊗ Family Practice
- ◯ Neurology
- ◯ Psychiatry
- ◯ ObGyn
- ◯ Neurosurgery
- ◯ General Surgery
- ◯ Orthopedic Surgery
- ◯ Urology
- ◯ Surgical sub-specialty (please specify in text box below)
- ◯ Non-clinical specialty (radiology, pathology; please specify in text box below)

Medical Trainees' attitudes, knowledge, and experience with immigrant and refugee health

Response was added on 11/01/2013 9:27pm.

SECTION A: Personal experience with immigrant and refugee health care.

A. Please indicate your level of agreement with the following statements regarding your personal experience with immigrant and refugee health care by checking the box that best represents your experience.

a. During my inpatient rotations, I take care of the following percentage of immigrant and refugee patients:	◯ None<br>◯ 0 -5%<br>⊗ 5-10%<br>⊗ 10-25%<br>◯ > 25%
b. During my outpatient rotations, I take care of the following percentage of immigrant and refugee patients:	◯ None<br>◯ 0-10%<br>◯ 10 -25%<br>◯ 25-50%<br>◯ 50-75%<br>⊗ >75%
c. I would like to take care of more immigrant and refugee patients.	◯ Strongly disagree<br>◯ Disagree<br>⊗ No opinion<br>◯ Agree<br>◯ Strongly agree
d. I plan to take care of immigrants and refugees when I finish residency.	◯ Strongly disagree<br>◯ Disagree<br>⊗ No opinion<br>◯ Agree<br>◯ Strongly agree
e. I plan to do short term (< 6 months) international work when I finish residency.	◯ Strongly disagree<br>◯ Disagree<br>◯ No opinion<br>⊗ Agree<br>◯ Strongly agree
f. I plan to do long term (>6 months) international work when I finish residency.	◯ Strongly disagree<br>◯ Disagree<br>⊗ No opinion<br>◯ Agree<br>◯ Strongly agree
g. I plan to work in health disparities in the following way after residency:	Don't know yet

SECTION B: MEDICAL EDUCATION

A. Please indicate your level of agreement with the following statements regarding your medical education and knowledge about immigrants and refugees by checking the box that best represents your opinion.

a. I have received specialized training in immigrant and refugee health, tropical medicine, or cross-cultural health.

- ◯ Strongly disagree
- ⊗ Disagree
- ◯ No opinion
- ◯ Agree
- ◯ Strongly agree

c. I feel comfortable with my fund of knowledge regarding immigrant and refugee health.

- ◯ Strongly disagree
- ⊗ Disagree
- ◯ No opinion
- ◯ Agree
- ◯ Strongly agree

d. I would like to have further training in immigrant and refugee health.

- ◯ Strongly disagree
- ◯ Disagree
- ◯ No opinion
- ◯ Agree
- ⊗ Strongly agree

e. If you agree with the above, please indicate all the contexts in which you would like to receive this training:

- ☒ As part of my residency.
- ☐ A special program.
- ☐ As part of my fellowship.

SECTION C: Attitudes towards immigrant health

A. Please indicate your level of agreement with the following statements regarding immigrant and refugee health by checking the box that best represents your opinion.

a. I enjoy taking care of immigrants and refugees.

- ◯ Never
- ◯ Rarely
- ⊗ Sometimes
- ◯ Usually
- ◯ Always

b. Please indicate the reasons that you enjoy taking care of immigrants and refugees (may choose more than one).

- ☒ Tropical and other conditions not frequently diagnosed in US-born patients
- ☒ Learning about other cultures
- ☐ They don't complain as much
- ☐ Being able to hear their stories
- ☐ Their care is more complicated
- ☐ Their care is less complicated
- ☒ They are very appreciative of your help.
- ☒ They are extremely vulnerable
- ☐ Other:

c. Taking care of immigrants and refugees is more challenging than taking care of US born patients.

- ◯ Never
- ◯ Rarely
- ◯ Sometimes
- ⊗ Usually
- ◯ Always

d. Please mark all the challenges that you face as a provider when providing care to immigrants and refugees (may choose more than one):

- ☒ Language barriers
- ☐ Insurance barriers
- ☒ Cultural barriers
- ☒ Finding a professional interpreter
- ☐ Knowing how to work with a professional interpreter
- ☒ Time constraints
- ☒ My own knowledge related to tropical and travel medicine
- ☐ Transportation problems for the patient
- ☒ Patients not understanding treatment plan
- ☒ Patients not following treatment plan
- ☒ My lack of knowledge regarding the patient's culture
- ☒ Bias or stereotyping
- ☐ Other:

e. Please mark all of the challenges faced by immigrant and refugee populations when receiving healthcare that you have perceived or witnessed (may choose more than one):

- ☒ Language barriers
- ☒ Insurance barriers
- ☒ Cultural barriers
- ☐ Finding a professional interpreter
- ☐ Knowing how to work with a professional interpreter
- ☐ Time constraints
- ☒ Insufficiently trained health care providers
- ☒ Transportation problems for the patient
- ☒ Food insufficiency
- ☒ Need for child care
- ☒ Patients not understanding treatment plan
- ☒ Patients not following treatment plan
- ☒ My lack of knowledge regarding the patient's culture
- ☒ Bias or stereotyping
- ☒ Trust issues
- ☐ Other...

f. Rank how well immigrants and refugees understand the healthcare that you are trying to provide.

- ◯ Significantly less than a US born individual
- ⊗ Less than a US born individual
- ◯ Equivalent to a US born individual
- ◯ More than a US born patient
- ◯ Significantly more than a US born individual

g. Immigrants and refugees adhere to treatment plans and follow my recommendations.

- ◯ Never
- ◯ Rarely
- ⊗ Sometimes
- ◯ Usually
- ◯ Always

h. Immigrants and refugees should receive the same care and insurance coverage as US born patients.

- ◯ Never
- ◯ Rarely
- ◯ Sometimes
- ◯ Usually
- ⊗ Always

i. Immigrants and refugees who are undocumented should receive the same care and insurance coverage as US born patients.

- ◯ Never
- ◯ Rarely
- ◯ Sometimes
- ◯ Usually
- ⊗ Always

j. Every physician is professionally obligated to care for immigrants and refugees if they present to your clinic or hospital.

- ◯ Strongly disagree
- ◯ Disagree
- ⊗ No opinion
- ◯ Agree
- ◯ Strongly agree

k. Is healthcare a human right?
- ⊗ Yes
- ◯ No

B. If you wish, please tell us about what you enjoy or do not enjoy about immigrant and refugee health care and the greatest challenges you face in caring for this population.

____

SECTION D: DEMOGRAPHIC INFORMATION
Please answer the following questions by checking the box in front of the response choice that best describes you.

a. Your age?
- ◯ 20 to 24
- ⊗ 25 to 29
- ◯ 30 to 34
- ◯ 35 to 39
- ◯ 40 or older

b. Your gender?
- ◯ Female
- ⊗ Male
- ◯ Other

c. Are you Hispanic or Latino?
- ◯ Yes
- ⊗ No

d. What is your race? (Select one or more responses)
- ◯ American Indian or Alaska Native
- ◯ Asian (Please specify):
- ◯ Black or African American
- ◯ Native Hawaiian or Other Pacific Islander
- ⊗ White
- ◯ Other (Please specify):

e. Were you born in the United States?
- ⊗ Yes
- ◯ No

g. Your residency year?
- ⊗ PGY1
- ◯ PGY2
- ◯ PGY3
- ◯ PGY4
- ◯ PGY5

h. How would you classify your political ideology?
- ◯ Conservative
- ◯ Somewhat conservative
- ⊗ Moderate
- ◯ Somewhat liberal
- ◯ Liberal
- ◯ Other (Please specify):

i. Estimated level of educational debt?
- ◯ None
- ◯ Less than $50,000
- ◯ $50,000 - $100,000
- ◯ $100,000 - $200,000
- ⊗ $200,000 or more

j. Do you plan to subspecialize?
- ◯ Yes
- ⊗ No

k. Languages spoken?

☒ English
☐ Spanish
☐ French
☐ Hmong
☐ Somali
☐ Japanese
☐ Chinese
☐ Russian
☐ Ethiopian
☐ Other ___________

l. Are you in the Global Health Pathway?

⊗ Yes
◯ No

m. Did you earn your degree in the US?

⊗ Yes
◯ No

n. What residency program are you in?

◯ Internal Medicine
⊗ Med-Peds
◯ Pediatrics
◯ Family Practice
◯ Neurology
◯ Psychiatry
◯ ObGyn
◯ Neurosurgery
◯ General Surgery
◯ Orthopedic Surgery
◯ Urology
◯ Surgical sub-specialty (please specify in text box below)
◯ Non-clinical specialty (radiology, pathology; please specify in text box below)

Medical Trainees' attitudes, knowledge, and experience with immigrant and refugee health

Response was added on 11/01/2013 10:22pm.

SECTION A: Personal experience with immigrant and refugee health care.

A. Please indicate your level of agreement with the following statements regarding your personal experience with immigrant and refugee health care by checking the box that best represents your experience.

a. During my inpatient rotations, I take care of the following percentage of immigrant and refugee patients:	◯ None<br>◯ 0 -5%<br>◯ 5-10%<br>⊗ 10-25%<br>◯ > 25%
b. During my outpatient rotations, I take care of the following percentage of immigrant and refugee patients:	◯ None<br>⊗ 0-10%<br>◯ 10 -25%<br>◯ 25-50%<br>◯ 50-75%<br>◯ >75%
c. I would like to take care of more immigrant and refugee patients.	◯ Strongly disagree<br>◯ Disagree<br>◯ No opinion<br>⊗ Agree<br>◯ Strongly agree
d. I plan to take care of immigrants and refugees when I finish residency.	◯ Strongly disagree<br>◯ Disagree<br>⊗ No opinion<br>◯ Agree<br>◯ Strongly agree
e. I plan to do short term (< 6 months) international work when I finish residency.	⊗ Strongly disagree<br>◯ Disagree<br>◯ No opinion<br>◯ Agree<br>◯ Strongly agree
f. I plan to do long term (>6 months) international work when I finish residency.	⊗ Strongly disagree<br>◯ Disagree<br>◯ No opinion<br>◯ Agree<br>◯ Strongly agree
g. I plan to work in health disparities in the following way after residency:	Working to provide equal care for low SES

SECTION B: MEDICAL EDUCATION

A. Please indicate your level of agreement with the following statements regarding your medical education and knowledge about immigrants and refugees by checking the box that best represents your opinion.

a. I have received specialized training in immigrant and refugee health, tropical medicine, or cross-cultural health.

- ○ Strongly disagree
- ⊗ Disagree
- ○ No opinion
- ○ Agree
- ○ Strongly agree

c. I feel comfortable with my fund of knowledge regarding immigrant and refugee health.

- ○ Strongly disagree
- ⊗ Disagree
- ○ No opinion
- ○ Agree
- ○ Strongly agree

d. I would like to have further training in immigrant and refugee health.

- ○ Strongly disagree
- ○ Disagree
- ○ No opinion
- ⊗ Agree
- ○ Strongly agree

e. If you agree with the above, please indicate all the contexts in which you would like to receive this training:
□□

- ☒ As part of my residency.
- ☐ A special program.
- ☐ As part of my fellowship.

SECTION C: Attitudes towards immigrant health

A.□Please indicate your level of agreement with the following statements regarding immigrant and refugee health by checking the box that best represents your opinion.

a. I enjoy taking care of immigrants and refugees.

- ○ Never
- ○ Rarely
- ○ Sometimes
- ⊗ Usually
- ○ Always

b. Please indicate the reasons that you enjoy taking care of immigrants and refugees (may choose more than one).

- ☐ Tropical and other conditions not frequently diagnosed in US-born patients
- ☒ Learning about other cultures
- ☐ They don't complain as much
- ☐ Being able to hear their stories
- ☒ Their care is more complicated
- ☐ Their care is less complicated
- ☒ They are very appreciative of your help.
- ☐ They are extremely vulnerable
- ☐ Other:

c. Taking care of immigrants and refugees is more challenging than taking care of US born patients.

- ○ Never
- ○ Rarely
- ⊗ Sometimes
- ○ Usually
- ○ Always

d. Please mark all the challenges that you face as a provider when providing care to immigrants and refugees (may choose more than one):

- [x] Language barriers
- [x] Insurance barriers
- [x] Cultural barriers
- [ ] Finding a professional interpreter
- [ ] Knowing how to work with a professional interpreter
- [ ] Time constraints
- [x] My own knowledge related to tropical and travel medicine
- [ ] Transportation problems for the patient
- [x] Patients not understanding treatment plan
- [ ] Patients not following treatment plan
- [x] My lack of knowledge regarding the patient's culture
- [ ] Bias or stereotyping
- [ ] Other:

e. Please mark all of the challenges faced by immigrant and refugee populations when receiving healthcare that you have perceived or witnessed (may choose more than one):

- [x] Language barriers
- [x] Insurance barriers
- [x] Cultural barriers
- [ ] Finding a professional interpreter
- [ ] Knowing how to work with a professional interpreter
- [ ] Time constraints
- [x] Insufficiently trained health care providers
- [ ] Transportation problems for the patient
- [ ] Food insufficiency
- [ ] Need for child care
- [x] Patients not understanding treatment plan
- [ ] Patients not following treatment plan
- [ ] My lack of knowledge regarding the patient's culture
- [x] Bias or stereotyping
- [x] Trust issues
- [ ] Other...

f. Rank how well immigrants and refugees understand the healthcare that you are trying to provide.

- ◯ Significantly less than a US born individual
- ⊗ Less than a US born individual
- ◯ Equivalent to a US born individual
- ◯ More than a US born patient
- ◯ Significantly more than a US born individual

g. Immigrants and refugees adhere to treatment plans and follow my recommendations.

- ◯ Never
- ◯ Rarely
- ◯ Sometimes
- ⊗ Usually
- ◯ Always

h. Immigrants and refugees should receive the same care and insurance coverage as US born patients.

- ◯ Never
- ◯ Rarely
- ⊗ Sometimes
- ◯ Usually
- ◯ Always

i. Immigrants and refugees who are undocumented should receive the same care and insurance coverage as US born patients.

- ◯ Never
- ◯ Rarely
- ⊗ Sometimes
- ◯ Usually
- ◯ Always

j. Every physician is professionally obligated to care for immigrants and refugees if they present to your clinic or hospital.

- ◯ Strongly disagree
- ◯ Disagree
- ◯ No opinion
- ◯ Agree
- ⊗ Strongly agree

k. Is healthcare a human right?
- ⊗ Yes
- ◯ No

B. If you wish, please tell us about what you enjoy or do not enjoy about immigrant and refugee health care and the greatest challenges you face in caring for this population.

____

SECTION D: DEMOGRAPHIC INFORMATION
Please answer the following questions by checking the box in front of the response choice that best describes you.

a. Your age?
- ◯ 20 to 24
- ⊗ 25 to 29
- ◯ 30 to 34
- ◯ 35 to 39
- ◯ 40 or older

b. Your gender?
- ◯ Female
- ⊗ Male
- ◯ Other

c.Are you Hispanic or Latino?
- ◯ Yes
- ⊗ No

d. What is your race? (Select one or more responses)
- ◯ American Indian or Alaska Native
- ◯ Asian (Please specify):
- ◯ Black or African American
- ◯ Native Hawaiian or Other Pacific Islander
- ⊗ White
- ◯ Other (Please specify):

e.Were you born in the United States?
- ⊗ Yes
- ◯ No

g. Your residency year?
- ◯ PGY1
- ⊗ PGY2
- ◯ PGY3
- ◯ PGY4
- ◯ PGY5

h. How would you classify your political ideology?
- ◯ Conservative
- ◯ Somewhat conservative
- ◯ Moderate
- ⊗ Somewhat liberal
- ◯ Liberal
- ◯ Other (Please specify):

i. Estimated level of educational debt?
- ◯ None
- ◯ Less than $50,000
- ⊗ $50,000 - $100,000
- ◯ $100,000 - $200,000
- ◯ $200,000 or more

j.Do you plan to subspecialize?
- ⊗ Yes
- ◯ No

k. Languages spoken?

- ☒ English
- ☐ Spanish
- ☐ French
- ☐ Hmong
- ☐ Somali
- ☐ Japanese
- ☐ Chinese
- ☐ Russian
- ☐ Ethiopian
- ☐ Other ___________

l. Are you in the Global Health Pathway?

- ◯ Yes
- ⊗ No

m. Did you earn your degree in the US?

- ⊗ Yes
- ◯ No

n. What residency program are you in?

- ◯ Internal Medicine
- ⊗ Med-Peds
- ◯ Pediatrics
- ◯ Family Practice
- ◯ Neurology
- ◯ Psychiatry
- ◯ ObGyn
- ◯ Neurosurgery
- ◯ General Surgery
- ◯ Orthopedic Surgery
- ◯ Urology
- ◯ Surgical sub-specialty (please specify in text box below)
- ◯ Non-clinical specialty (radiology, pathology; please specify in text box below)

Medical Trainees' attitudes, knowledge, and experience with immigrant and refugee health

Response was added on 11/02/2013 3:17am.

SECTION A: Personal experience with immigrant and refugee health care.

A. Please indicate your level of agreement with the following statements regarding your personal experience with immigrant and refugee health care by checking the box that best represents your experience.

a. During my inpatient rotations, I take care of the following percentage of immigrant and refugee patients:

- ◯ None
- ◯ 0 -5%
- ⊗ 5-10%
- ◯ 10-25%
- ◯ > 25%

b. During my outpatient rotations, I take care of the following percentage of immigrant and refugee patients:

- ◯ None
- ◯ 0-10%
- ◯ 10 -25%
- ⊗ 25-50%
- ◯ 50-75%
- ◯ >75%

c. I would like to take care of more immigrant and refugee patients.

- ◯ Strongly disagree
- ◯ Disagree
- ⊗ No opinion
- ◯ Agree
- ◯ Strongly agree

d. I plan to take care of immigrants and refugees when I finish residency.

- ◯ Strongly disagree
- ◯ Disagree
- ◯ No opinion
- ⊗ Agree
- ◯ Strongly agree

e. I plan to do short term (< 6 months) international work when I finish residency.

- ⊗ Strongly disagree
- ◯ Disagree
- ◯ No opinion
- ◯ Agree
- ◯ Strongly agree

f. I plan to do long term (>6 months) international work when I finish residency.

- ⊗ Strongly disagree
- ◯ Disagree
- ◯ No opinion
- ◯ Agree
- ◯ Strongly agree

g. I plan to work in health disparities in the following way after residency:

Hope to work in the city, so I can provide care to the underserved. Hope to do political advocacy to expand health care access.

SECTION B: MEDICAL EDUCATION

A. Please indicate your level of agreement with the following statements regarding your medical education and knowledge about immigrants and refugees by checking the box that best represents your opinion.

a. I have received specialized training in immigrant and refugee health, tropical medicine, or cross-cultural health.

- ○ Strongly disagree
- ○ Disagree
- ○ No opinion
- ⊗ Agree
- ○ Strongly agree

b. If you have received specialized training in immigrant and refugee health, tropical medicine, or cross-cultural health, please indicate all the contexts in which you received this training:

- ☐ As an undergraduate.
- ☐ As a medical student.
- ☒ As part of my residency.
- ☐ A special program.
- ☐ As part of my fellowship.
- ☐ As part of a degree program (e.g. MPH)
- ☐ Other:

c. I feel comfortable with my fund of knowledge regarding immigrant and refugee health.

- ○ Strongly disagree
- ⊗ Disagree
- ○ No opinion
- ○ Agree
- ○ Strongly agree

d. I would like to have further training in immigrant and refugee health.

- ○ Strongly disagree
- ○ Disagree
- ○ No opinion
- ⊗ Agree
- ○ Strongly agree

e. If you agree with the above, please indicate all the contexts in which you would like to receive this training:

- ☒ As part of my residency.
- ☐ A special program.
- ☐ As part of my fellowship.

SECTION C: Attitudes towards immigrant health

A. Please indicate your level of agreement with the following statements regarding immigrant and refugee health by checking the box that best represents your opinion.

a. I enjoy taking care of immigrants and refugees.

- ○ Never
- ○ Rarely
- ○ Sometimes
- ⊗ Usually
- ○ Always

b. Please indicate the reasons that you enjoy taking care of immigrants and refugees (may choose more than one).

- ☐ Tropical and other conditions not frequently diagnosed in US-born patients
- ☒ Learning about other cultures
- ☐ They don't complain as much
- ☒ Being able to hear their stories
- ☐ Their care is more complicated
- ☐ Their care is less complicated
- ☒ They are very appreciative of your help.
- ☐ They are extremely vulnerable
- ☐ Other:

c. Taking care of immigrants and refugees is more challenging than taking care of US born patients.

- ○ Never
- ○ Rarely
- ⊗ Sometimes
- ○ Usually
- ○ Always

d. Please mark all the challenges that you face as a provider when providing care to immigrants and refugees (may choose more than one):

- ☒ Language barriers
- ☐ Insurance barriers
- ☒ Cultural barriers
- ☐ Finding a professional interpreter
- ☐ Knowing how to work with a professional interpreter
- ☐ Time constraints
- ☒ My own knowledge related to tropical and travel medicine
- ☒ Transportation problems for the patient
- ☒ Patients not understanding treatment plan
- ☐ Patients not following treatment plan
- ☒ My lack of knowledge regarding the patient's culture
- ☐ Bias or stereotyping
- ☐ Other:

e. Please mark all of the challenges faced by immigrant and refugee populations when receiving healthcare that you have perceived or witnessed (may choose more than one):

- ☒ Language barriers
- ☒ Insurance barriers
- ☒ Cultural barriers
- ☐ Finding a professional interpreter
- ☐ Knowing how to work with a professional interpreter
- ☒ Time constraints
- ☐ Insufficiently trained health care providers
- ☒ Transportation problems for the patient
- ☒ Food insufficiency
- ☒ Need for child care
- ☒ Patients not understanding treatment plan
- ☒ Patients not following treatment plan
- ☐ My lack of knowledge regarding the patient's culture
- ☐ Bias or stereotyping
- ☒ Trust issues
- ☐ Other...

f. Rank how well immigrants and refugees understand the healthcare that you are trying to provide.

- ◯ Significantly less than a US born individual
- ⊗ Less than a US born individual
- ◯ Equivalent to a US born individual
- ◯ More than a US born patient
- ◯ Significantly more than a US born individual

g. Immigrants and refugees adhere to treatment plans and follow my recommendations.

- ◯ Never
- ◯ Rarely
- ⊗ Sometimes
- ◯ Usually
- ◯ Always

h. Immigrants and refugees should receive the same care and insurance coverage as US born patients.

- ◯ Never
- ◯ Rarely
- ◯ Sometimes
- ◯ Usually
- ⊗ Always

i. Immigrants and refugees who are undocumented should receive the same care and insurance coverage as US born patients.

- ◯ Never
- ◯ Rarely
- ◯ Sometimes
- ◯ Usually
- ⊗ Always

j. Every physician is professionally obligated to care for immigrants and refugees if they present to your clinic or hospital.

- ◯ Strongly disagree
- ◯ Disagree
- ◯ No opinion
- ◯ Agree
- ⊗ Strongly agree

k. Is healthcare a human right?

⊗ Yes
◯ No

B. If you wish, please tell us about what you enjoy or do not enjoy about immigrant and refugee health care and the greatest challenges you face in caring for this population.

____

SECTION D: DEMOGRAPHIC INFORMATION
Please answer the following questions by checking the box in front of the response choice that best describes you.

a. Your age?

◯ 20 to 24
⊗ 25 to 29
◯ 30 to 34
◯ 35 to 39
◯ 40 or older

b. Your gender?

⊗ Female
◯ Male
◯ Other

c. Are you Hispanic or Latino?

◯ Yes
⊗ No

d. What is your race? (Select one or more responses)

◯ American Indian or Alaska Native
◯ Asian (Please specify):
◯ Black or African American
◯ Native Hawaiian or Other Pacific Islander
⊗ White
◯ Other (Please specify):

e. Were you born in the United States?

⊗ Yes
◯ No

g. Your residency year?

⊗ PGY1
◯ PGY2
◯ PGY3
◯ PGY4
◯ PGY5

h. How would you classify your political ideology?

◯ Conservative
◯ Somewhat conservative
◯ Moderate
◯ Somewhat liberal
⊗ Liberal
◯ Other (Please specify):

i. Estimated level of educational debt?

⊗ None
◯ Less than $50,000
◯ $50,000 - $100,000
◯ $100,000 - $200,000
◯ $200,000 or more

j. Do you plan to subspecialize?

⊗ Yes
◯ No

k. Languages spoken?

- ☒ English
- ☐ Spanish
- ☐ French
- ☐ Hmong
- ☐ Somali
- ☐ Japanese
- ☐ Chinese
- ☐ Russian
- ☐ Ethiopian
- ☐ Other ___________

l. Are you in the Global Health Pathway?

- ◯ Yes
- ⊗ No

m. Did you earn your degree in the US?

- ⊗ Yes
- ◯ No

n. What residency program are you in?

- ◯ Internal Medicine
- ◯ Med-Peds
- ⊗ Pediatrics
- ◯ Family Practice
- ◯ Neurology
- ◯ Psychiatry
- ◯ ObGyn
- ◯ Neurosurgery
- ◯ General Surgery
- ◯ Orthopedic Surgery
- ◯ Urology
- ◯ Surgical sub-specialty (please specify in text box below)
- ◯ Non-clinical specialty (radiology, pathology; please specify in text box below)

Medical Trainees' attitudes, knowledge, and experience with immigrant and refugee health

Response was added on 11/02/2013 3:46am.

SECTION A: Personal experience with immigrant and refugee health care.

A. Please indicate your level of agreement with the following statements regarding your personal experience with immigrant and refugee health care by checking the box that best represents your experience.

a. During my inpatient rotations, I take care of the following percentage of immigrant and refugee patients:
- ◯ None
- ◯ 0 -5%
- ⊗ 5-10%
- ◯ 10-25%
- ◯ > 25%

b. During my outpatient rotations, I take care of the following percentage of immigrant and refugee patients:
- ◯ None
- ◯ 0-10%
- ◯ 10 -25%
- ◯ 25-50%
- ◯ 50-75%
- ⊗ >75%

c. I would like to take care of more immigrant and refugee patients.
- ◯ Strongly disagree
- ◯ Disagree
- ◯ No opinion
- ⊗ Agree
- ◯ Strongly agree

d. I plan to take care of immigrants and refugees when I finish residency.
- ◯ Strongly disagree
- ◯ Disagree
- ◯ No opinion
- ◯ Agree
- ⊗ Strongly agree

e. I plan to do short term (< 6 months) international work when I finish residency.
- ◯ Strongly disagree
- ◯ Disagree
- ◯ No opinion
- ◯ Agree
- ⊗ Strongly agree

f. I plan to do long term (>6 months) international work when I finish residency.
- ◯ Strongly disagree
- ◯ Disagree
- ◯ No opinion
- ◯ Agree
- ⊗ Strongly agree

g. I plan to work in health disparities in the following way after residency:

Spanish speaking populations and immigrants

SECTION B: MEDICAL EDUCATION

A. Please indicate your level of agreement with the following statements regarding your medical education and knowledge about immigrants and refugees by checking the box that best represents your opinion.

a. I have received specialized training in immigrant and refugee health, tropical medicine, or cross-cultural health.

- ◯ Strongly disagree
- ◯ Disagree
- ◯ No opinion
- ⊗ Agree
- ◯ Strongly agree

b. If you have received specialized training in immigrant and refugee health, tropical medicine, or cross-cultural health, please indicate all the contexts in which you received this training:

- ☐ As an undergraduate.
- ☐ As a medical student.
- ☒ As part of my residency.
- ☐ A special program.
- ☐ As part of my fellowship.
- ☐ As part of a degree program (e.g. MPH)
- ☐ Other:

c. I feel comfortable with my fund of knowledge regarding immigrant and refugee health.

- ◯ Strongly disagree
- ⊗ Disagree
- ◯ No opinion
- ◯ Agree
- ◯ Strongly agree

d. I would like to have further training in immigrant and refugee health.

- ◯ Strongly disagree
- ◯ Disagree
- ◯ No opinion
- ◯ Agree
- ⊗ Strongly agree

e. If you agree with the above, please indicate all the contexts in which you would like to receive this training:

- ☐ As part of my residency.
- ☐ A special program.
- ☐ As part of my fellowship.

SECTION C: Attitudes towards immigrant health

A. Please indicate your level of agreement with the following statements regarding immigrant and refugee health by checking the box that best represents your opinion.

a. I enjoy taking care of immigrants and refugees.

- ◯ Never
- ◯ Rarely
- ◯ Sometimes
- ⊗ Usually
- ◯ Always

b. Please indicate the reasons that you enjoy taking care of immigrants and refugees (may choose more than one).

- ☒ Tropical and other conditions not frequently diagnosed in US-born patients
- ☒ Learning about other cultures
- ☐ They don't complain as much
- ☐ Being able to hear their stories
- ☐ Their care is more complicated
- ☐ Their care is less complicated
- ☒ They are very appreciative of your help.
- ☒ They are extremely vulnerable
- ☐ Other:

c. Taking care of immigrants and refugees is more challenging than taking care of US born patients.

- ◯ Never
- ◯ Rarely
- ◯ Sometimes
- ◯ Usually
- ⊗ Always

d. Please mark all the challenges that you face as a provider when providing care to immigrants and refugees (may choose more than one):

- ☒ Language barriers
- ☐ Insurance barriers
- ☐ Cultural barriers
- ☒ Finding a professional interpreter
- ☐ Knowing how to work with a professional interpreter
- ☒ Time constraints
- ☐ My own knowledge related to tropical and travel medicine
- ☒ Transportation problems for the patient
- ☒ Patients not understanding treatment plan
- ☐ Patients not following treatment plan
- ☐ My lack of knowledge regarding the patient's culture
- ☐ Bias or stereotyping
- ☐ Other:

e. Please mark all of the challenges faced by immigrant and refugee populations when receiving healthcare that you have perceived or witnessed (may choose more than one):

- ☒ Language barriers
- ☒ Insurance barriers
- ☒ Cultural barriers
- ☐ Finding a professional interpreter
- ☐ Knowing how to work with a professional interpreter
- ☐ Time constraints
- ☒ Insufficiently trained health care providers
- ☒ Transportation problems for the patient
- ☐ Food insufficiency
- ☒ Need for child care
- ☒ Patients not understanding treatment plan
- ☐ Patients not following treatment plan
- ☐ My lack of knowledge regarding the patient's culture
- ☐ Bias or stereotyping
- ☐ Trust issues
- ☐ Other...

f. Rank how well immigrants and refugees understand the healthcare that you are trying to provide.

- ⊗ Significantly less than a US born individual
- ○ Less than a US born individual
- ○ Equivalent to a US born individual
- ○ More than a US born patient
- ○ Significantly more than a US born individual

g. Immigrants and refugees adhere to treatment plans and follow my recommendations.

- ○ Never
- ○ Rarely
- ⊗ Sometimes
- ○ Usually
- ○ Always

h. Immigrants and refugees should receive the same care and insurance coverage as US born patients.

- ○ Never
- ○ Rarely
- ○ Sometimes
- ○ Usually
- ⊗ Always

i. Immigrants and refugees who are undocumented should receive the same care and insurance coverage as US born patients.

- ○ Never
- ○ Rarely
- ○ Sometimes
- ⊗ Usually
- ○ Always

j. Every physician is professionally obligated to care for immigrants and refugees if they present to your clinic or hospital.

- ○ Strongly disagree
- ○ Disagree
- ○ No opinion
- ⊗ Agree
- ○ Strongly agree

k. Is healthcare a human right?
- ⊗ Yes
- ◯ No

B. If you wish, please tell us about what you enjoy or do not enjoy about immigrant and refugee health care and the greatest challenges you face in caring for this population.

____

SECTION D: DEMOGRAPHIC INFORMATION
Please answer the following questions by checking the box in front of the response choice that best describes you.

a. Your age?
- ◯ 20 to 24
- ⊗ 25 to 29
- ◯ 30 to 34
- ◯ 35 to 39
- ◯ 40 or older

b. Your gender?
- ⊗ Female
- ◯ Male
- ◯ Other

c.Are you Hispanic or Latino?
- ◯ Yes
- ⊗ No

d. What is your race? (Select one or more responses)
- ◯ American Indian or Alaska Native
- ⊗ Asian (Please specify):
- ◯ Black or African American
- ◯ Native Hawaiian or Other Pacific Islander
- ◯ White
- ◯ Other (Please specify):

e.Were you born in the United States?
- ⊗ Yes
- ◯ No

g. Your residency year?
- ◯ PGY1
- ⊗ PGY2
- ◯ PGY3
- ◯ PGY4
- ◯ PGY5

h. How would you classify your political ideology?
- ◯ Conservative
- ◯ Somewhat conservative
- ◯ Moderate
- ◯ Somewhat liberal
- ⊗ Liberal
- ◯ Other (Please specify):

i. Estimated level of educational debt?
- ⊗ None
- ◯ Less than $50,000
- ◯ $50,000 - $100,000
- ◯ $100,000 - $200,000
- ◯ $200,000 or more

j.Do you plan to subspecialize?
- ⊗ Yes
- ◯ No

k. Languages spoken?

☒ English
☒ Spanish
☐ French
☐ Hmong
☐ Somali
☐ Japanese
☐ Chinese
☐ Russian
☐ Ethiopian
☒ Other ___________

Gujarati

l. Are you in the Global Health Pathway?

⊗ Yes
◯ No

m. Did you earn your degree in the US?

⊗ Yes
◯ No

n. What residency program are you in?

⊗ Internal Medicine
◯ Med-Peds
◯ Pediatrics
◯ Family Practice
◯ Neurology
◯ Psychiatry
◯ ObGyn
◯ Neurosurgery
◯ General Surgery
◯ Orthopedic Surgery
◯ Urology
◯ Surgical sub-specialty (please specify in text box below)
◯ Non-clinical specialty (radiology, pathology; please specify in text box below)

Medical Trainees' attitudes, knowledge, and experience with immigrant and refugee health

Response was added on 11/02/2013 3:58am.

SECTION A: Personal experience with immigrant and refugee health care.

A. Please indicate your level of agreement with the following statements regarding your personal experience with immigrant and refugee health care by checking the box that best represents your experience.

a. During my inpatient rotations, I take care of the following percentage of immigrant and refugee patients:

- ◯ None
- ◯ 0 -5%
- ⊗ 5-10%
- ◯ 10-25%
- ◯ > 25%

b. During my outpatient rotations, I take care of the following percentage of immigrant and refugee patients:

- ◯ None
- ⊗ 0-10%
- ◯ 10 -25%
- ◯ 25-50%
- ◯ 50-75%
- ◯ >75%

c. I would like to take care of more immigrant and refugee patients.

- ◯ Strongly disagree
- ◯ Disagree
- ⊗ No opinion
- ◯ Agree
- ◯ Strongly agree

d. I plan to take care of immigrants and refugees when I finish residency.

- ◯ Strongly disagree
- ◯ Disagree
- ⊗ No opinion
- ◯ Agree
- ◯ Strongly agree

e. I plan to do short term (< 6 months) international work when I finish residency.

- ◯ Strongly disagree
- ⊗ Disagree
- ◯ No opinion
- ◯ Agree
- ◯ Strongly agree

f. I plan to do long term (>6 months) international work when I finish residency.

- ⊗ Strongly disagree
- ◯ Disagree
- ◯ No opinion
- ◯ Agree
- ◯ Strongly agree

g. I plan to work in health disparities in the following way after residency: no

SECTION B: MEDICAL EDUCATION

A. Please indicate your level of agreement with the following statements regarding your medical education and knowledge about immigrants and refugees by checking the box that best represents your opinion.

a. I have received specialized training in immigrant and refugee health, tropical medicine, or cross-cultural health.

- ◯ Strongly disagree
- ⊗ Disagree
- ◯ No opinion
- ◯ Agree
- ◯ Strongly agree

c. I feel comfortable with my fund of knowledge regarding immigrant and refugee health.

- ⊗ Strongly disagree
- ◯ Disagree
- ◯ No opinion
- ◯ Agree
- ◯ Strongly agree

d. I would like to have further training in immigrant and refugee health.

- ◯ Strongly disagree
- ◯ Disagree
- ⊗ No opinion
- ◯ Agree
- ◯ Strongly agree

SECTION C: Attitudes towards immigrant health

A.Please indicate your level of agreement with the following statements regarding immigrant and refugee health by checking the box that best represents your opinion.

a. I enjoy taking care of immigrants and refugees.

- ◯ Never
- ◯ Rarely
- ◯ Sometimes
- ◯ Usually
- ⊗ Always

b. Please indicate the reasons that you enjoy taking care of immigrants and refugees (may choose more than one).

- ☒ Tropical and other conditions not frequently diagnosed in US-born patients
- ☒ Learning about other cultures
- ☐ They don't complain as much
- ☒ Being able to hear their stories
- ☐ Their care is more complicated
- ☐ Their care is less complicated
- ☒ They are very appreciative of your help.
- ☐ They are extremely vulnerable
- ☐ Other:

c. Taking care of immigrants and refugees is more challenging than taking care of US born patients.

- ◯ Never
- ◯ Rarely
- ◯ Sometimes
- ◯ Usually
- ⊗ Always

d. Please mark all the challenges that you face as a provider when providing care to immigrants and refugees (may choose more than one):

- ☒ Language barriers
- ☐ Insurance barriers
- ☒ Cultural barriers
- ☒ Finding a professional interpreter
- ☐ Knowing how to work with a professional interpreter
- ☒ Time constraints
- ☒ My own knowledge related to tropical and travel medicine
- ☐ Transportation problems for the patient
- ☐ Patients not understanding treatment plan
- ☐ Patients not following treatment plan
- ☒ My lack of knowledge regarding the patient's culture
- ☐ Bias or stereotyping
- ☐ Other:

e. Please mark all of the challenges faced by immigrant and refugee populations when receiving healthcare that you have perceived or witnessed (may choose more than one):

- ☒ Language barriers
- ☒ Insurance barriers
- ☒ Cultural barriers
- ☒ Finding a professional interpreter
- ☐ Knowing how to work with a professional interpreter
- ☐ Time constraints
- ☐ Insufficiently trained health care providers
- ☒ Transportation problems for the patient
- ☒ Food insufficiency
- ☒ Need for child care
- ☐ Patients not understanding treatment plan
- ☐ Patients not following treatment plan
- ☒ My lack of knowledge regarding the patient's culture
- ☐ Bias or stereotyping
- ☒ Trust issues
- ☐ Other...

f. Rank how well immigrants and refugees understand the healthcare that you are trying to provide.

- ◯ Significantly less than a US born individual
- ◯ Less than a US born individual
- ⊗ Equivalent to a US born individual
- ◯ More than a US born patient
- ◯ Significantly more than a US born individual

g. Immigrants and refugees adhere to treatment plans and follow my recommendations.

- ◯ Never
- ◯ Rarely
- ◯ Sometimes
- ◯ Usually
- ⊗ Always

h. Immigrants and refugees should receive the same care and insurance coverage as US born patients.

- ◯ Never
- ◯ Rarely
- ◯ Sometimes
- ⊗ Usually
- ◯ Always

i. Immigrants and refugees who are undocumented should receive the same care and insurance coverage as US born patients.

- ◯ Never
- ◯ Rarely
- ⊗ Sometimes
- ◯ Usually
- ◯ Always

j. Every physician is professionally obligated to care for immigrants and refugees if they present to your clinic or hospital.

- ◯ Strongly disagree
- ◯ Disagree
- ◯ No opinion
- ◯ Agree
- ⊗ Strongly agree

k. Is healthcare a human right?

- ⊗ Yes
- ◯ No

B. If you wish, please tell us about what you enjoy or do not enjoy about immigrant and refugee health care and the greatest challenges you face in caring for this population.

____

SECTION D: DEMOGRAPHIC INFORMATION
Please answer the following questions by checking the box in front of the response choice that best describes you.

a. Your age?
- ◯ 20 to 24
- ⊗ 25 to 29
- ◯ 30 to 34
- ◯ 35 to 39
- ◯ 40 or older

b. Your gender?
- ◯ Female
- ⊗ Male
- ◯ Other

c. Are you Hispanic or Latino?
- ◯ Yes
- ⊗ No

d. What is your race? (Select one or more responses)
- ◯ American Indian or Alaska Native
- ◯ Asian (Please specify):
- ◯ Black or African American
- ⊗ Native Hawaiian or Other Pacific Islander
- ◯ White
- ◯ Other (Please specify):

e. Were you born in the United States?
- ⊗ Yes
- ◯ No

g. Your residency year?
- ◯ PGY1
- ◯ PGY2
- ⊗ PGY3
- ◯ PGY4
- ◯ PGY5

h. How would you classify your political ideology?
- ◯ Conservative
- ◯ Somewhat conservative
- ◯ Moderate
- ⊗ Somewhat liberal
- ◯ Liberal
- ◯ Other (Please specify):

i. Estimated level of educational debt?
- ⊗ None
- ◯ Less than $50,000
- ◯ $50,000 - $100,000
- ◯ $100,000 - $200,000
- ◯ $200,000 or more

j. Do you plan to subspecialize?
- ⊗ Yes
- ◯ No

k. Languages spoken?
- ☒ English
- ☐ Spanish
- ☒ French
- ☐ Hmong
- ☐ Somali
- ☐ Japanese
- ☒ Chinese
- ☐ Russian
- ☐ Ethiopian
- ☐ Other ___________

l. Are you in the Global Health Pathway?
- ◯ Yes
- ⊗ No

m.􀀀 Did you earn your degree in the US?

- ⊗ Yes
- ◯ No

n. What residency program are you in?

- ⊗ Internal Medicine
- ◯ Med-Peds
- ◯ Pediatrics
- ◯ Family Practice
- ◯ Neurology
- ◯ Psychiatry
- ◯ ObGyn
- ◯ Neurosurgery
- ◯ General Surgery
- ◯ Orthopedic Surgery
- ◯ Urology
- ◯ Surgical sub-specialty (please specify in text box below)
- ◯ Non-clinical specialty (radiology, pathology; please specify in text box below)

Medical Trainees' attitudes, knowledge, and experience with immigrant and refugee health

Response was added on 11/02/2013 6:28am.

SECTION A: Personal experience with immigrant and refugee health care.

A. Please indicate your level of agreement with the following statements regarding your personal experience with immigrant and refugee health care by checking the box that best represents your experience.

a. During my inpatient rotations, I take care of the following percentage of immigrant and refugee patients:

- ◯ None
- ◯ 0 -5%
- ◯ 5-10%
- ⊗ 10-25%
- ◯ > 25%

b. During my outpatient rotations, I take care of the following percentage of immigrant and refugee patients:

- ◯ None
- ◯ 0-10%
- ⊗ 10 -25%
- ◯ 25-50%
- ◯ 50-75%
- ◯ >75%

c. I would like to take care of more immigrant and refugee patients.

- ◯ Strongly disagree
- ◯ Disagree
- ⊗ No opinion
- ◯ Agree
- ◯ Strongly agree

d. I plan to take care of immigrants and refugees when I finish residency.

- ◯ Strongly disagree
- ◯ Disagree
- ◯ No opinion
- ⊗ Agree
- ◯ Strongly agree

e. I plan to do short term (< 6 months) international work when I finish residency.

- ◯ Strongly disagree
- ◯ Disagree
- ◯ No opinion
- ⊗ Agree
- ◯ Strongly agree

f. I plan to do long term (>6 months) international work when I finish residency.

- ◯ Strongly disagree
- ◯ Disagree
- ⊗ No opinion
- ◯ Agree
- ◯ Strongly agree

g. I plan to work in health disparities in the following way after residency:

Expect immigrants/refugees to make up part of my patient population

SECTION B: MEDICAL EDUCATION

A. Please indicate your level of agreement with the following statements regarding your medical education and knowledge about immigrants and refugees by checking the box that best represents your opinion.

a. I have received specialized training in immigrant and refugee health, tropical medicine, or cross-cultural health.

- ◯ Strongly disagree
- ◯ Disagree
- ◯ No opinion
- ⊗ Agree
- ◯ Strongly agree

b. If you have received specialized training in immigrant and refugee health, tropical medicine, or cross-cultural health, please indicate all the contexts in which you received this training:

- ☐ As an undergraduate.
- ☒ As a medical student.
- ☐ As part of my residency.
- ☐ A special program.
- ☐ As part of my fellowship.
- ☐ As part of a degree program (e.g. MPH)
- ☐ Other:

c. I feel comfortable with my fund of knowledge regarding immigrant and refugee health.

- ◯ Strongly disagree
- ⊗ Disagree
- ◯ No opinion
- ◯ Agree
- ◯ Strongly agree

d. I would like to have further training in immigrant and refugee health.

- ◯ Strongly disagree
- ◯ Disagree
- ◯ No opinion
- ⊗ Agree
- ◯ Strongly agree

e. If you agree with the above, please indicate all the contexts in which you would like to receive this training:
□□

- ☐ As part of my residency.
- ☒ A special program.
- ☐ As part of my fellowship.

SECTION C: Attitudes towards immigrant health

A.□Please indicate your level of agreement with the following statements regarding immigrant and refugee health by checking the box that best represents your opinion.

a. I enjoy taking care of immigrants and refugees.

- ◯ Never
- ◯ Rarely
- ⊗ Sometimes
- ◯ Usually
- ◯ Always

b. Please indicate the reasons that you enjoy taking care of immigrants and refugees (may choose more than one).

- ☐ Tropical and other conditions not frequently diagnosed in US-born patients
- ☒ Learning about other cultures
- ☐ They don't complain as much
- ☐ Being able to hear their stories
- ☐ Their care is more complicated
- ☐ Their care is less complicated
- ☐ They are very appreciative of your help.
- ☒ They are extremely vulnerable
- ☐ Other:

c. Taking care of immigrants and refugees is more challenging than taking care of US born patients.

- ◯ Never
- ◯ Rarely
- ◯ Sometimes
- ⊗ Usually
- ◯ Always

d. Please mark all the challenges that you face as a provider when providing care to immigrants and refugees (may choose more than one):

- [x] Language barriers
- [x] Insurance barriers
- [x] Cultural barriers
- [x] Finding a professional interpreter
- [ ] Knowing how to work with a professional interpreter
- [x] Time constraints
- [ ] My own knowledge related to tropical and travel medicine
- [ ] Transportation problems for the patient
- [x] Patients not understanding treatment plan
- [x] Patients not following treatment plan
- [x] My lack of knowledge regarding the patient's culture
- [ ] Bias or stereotyping
- [ ] Other:

e. Please mark all of the challenges faced by immigrant and refugee populations when receiving healthcare that you have perceived or witnessed (may choose more than one):

- [x] Language barriers
- [x] Insurance barriers
- [ ] Cultural barriers
- [x] Finding a professional interpreter
- [ ] Knowing how to work with a professional interpreter
- [x] Time constraints
- [ ] Insufficiently trained health care providers
- [x] Transportation problems for the patient
- [ ] Food insufficiency
- [x] Need for child care
- [x] Patients not understanding treatment plan
- [ ] Patients not following treatment plan
- [ ] My lack of knowledge regarding the patient's culture
- [ ] Bias or stereotyping
- [ ] Trust issues
- [ ] Other...

f. Rank how well immigrants and refugees understand the healthcare that you are trying to provide.

- ○ Significantly less than a US born individual
- ⊗ Less than a US born individual
- ○ Equivalent to a US born individual
- ○ More than a US born patient
- ○ Significantly more than a US born individual

g. Immigrants and refugees adhere to treatment plans and follow my recommendations.

- ○ Never
- ○ Rarely
- ⊗ Sometimes
- ○ Usually
- ○ Always

h. Immigrants and refugees should receive the same care and insurance coverage as US born patients.

- ○ Never
- ○ Rarely
- ○ Sometimes
- ⊗ Usually
- ○ Always

i. Immigrants and refugees who are undocumented should receive the same care and insurance coverage as US born patients.

- ○ Never
- ○ Rarely
- ⊗ Sometimes
- ○ Usually
- ○ Always

j. Every physician is professionally obligated to care for immigrants and refugees if they present to your clinic or hospital.

- ○ Strongly disagree
- ○ Disagree
- ○ No opinion
- ⊗ Agree
- ○ Strongly agree

k. Is healthcare a human right?
- ◯ Yes
- ⊗ No

B. If you wish, please tell us about what you enjoy or do not enjoy about immigrant and refugee health care and the greatest challenges you face in caring for this population.

____

SECTION D: DEMOGRAPHIC INFORMATION
Please answer the following questions by checking the box in front of the response choice that best describes you.

a. Your age?
- ◯ 20 to 24
- ◯ 25 to 29
- ⊗ 30 to 34
- ◯ 35 to 39
- ◯ 40 or older

b. Your gender?
- ⊗ Female
- ◯ Male
- ◯ Other

c. Are you Hispanic or Latino?
- ◯ Yes
- ⊗ No

d. What is your race? (Select one or more responses)
- ◯ American Indian or Alaska Native
- ◯ Asian (Please specify):
- ◯ Black or African American
- ◯ Native Hawaiian or Other Pacific Islander
- ⊗ White
- ◯ Other (Please specify):

e. Were you born in the United States?
- ⊗ Yes
- ◯ No

g. Your residency year?
- ◯ PGY1
- ◯ PGY2
- ⊗ PGY3
- ◯ PGY4
- ◯ PGY5

h. How would you classify your political ideology?
- ◯ Conservative
- ◯ Somewhat conservative
- ⊗ Moderate
- ◯ Somewhat liberal
- ◯ Liberal
- ◯ Other (Please specify):

i. Estimated level of educational debt?
- ◯ None
- ◯ Less than $50,000
- ◯ $50,000 - $100,000
- ◯ $100,000 - $200,000
- ⊗ $200,000 or more

j. Do you plan to subspecialize?
- ⊗ Yes
- ◯ No

k. Languages spoken?

- ☒ English
- ☐ Spanish
- ☐ French
- ☐ Hmong
- ☐ Somali
- ☐ Japanese
- ☐ Chinese
- ☐ Russian
- ☐ Ethiopian
- ☐ Other ___________

l. Are you in the Global Health Pathway?

- ◯ Yes
- ⊗ No

m.□ Did you earn your degree in the US?

- ⊗ Yes
- ◯ No

n. What residency program are you in?

- ◯ Internal Medicine
- ◯ Med-Peds
- ◯ Pediatrics
- ◯ Family Practice
- ◯ Neurology
- ◯ Psychiatry
- ◯ ObGyn
- ◯ Neurosurgery
- ◯ General Surgery
- ⊗ Orthopedic Surgery
- ◯ Urology
- ◯ Surgical sub-specialty (please specify in text box below)
- ◯ Non-clinical specialty (radiology, pathology; please specify in text box below)

Medical Trainees' attitudes, knowledge, and experience with immigrant and refugee health

Response was added on 11/02/2013 7:27am.

SECTION A: Personal experience with immigrant and refugee health care.

A. Please indicate your level of agreement with the following statements regarding your personal experience with immigrant and refugee health care by checking the box that best represents your experience.

a. During my inpatient rotations, I take care of the following percentage of immigrant and refugee patients:

- ◯ None
- ◯ 0 -5%
- ⊗ 5-10%
- ◯ 10-25%
- ◯ > 25%

b. During my outpatient rotations, I take care of the following percentage of immigrant and refugee patients:

- ◯ None
- ⊗ 0-10%
- ◯ 10 -25%
- ◯ 25-50%
- ◯ 50-75%
- ◯ >75%

c. I would like to take care of more immigrant and refugee patients.

- ◯ Strongly disagree
- ◯ Disagree
- ⊗ No opinion
- ◯ Agree
- ◯ Strongly agree

d. I plan to take care of immigrants and refugees when I finish residency.

- ◯ Strongly disagree
- ◯ Disagree
- ◯ No opinion
- ⊗ Agree
- ◯ Strongly agree

e. I plan to do short term (< 6 months) international work when I finish residency.

- ◯ Strongly disagree
- ◯ Disagree
- ⊗ No opinion
- ◯ Agree
- ◯ Strongly agree

f. I plan to do long term (>6 months) international work when I finish residency.

- ◯ Strongly disagree
- ⊗ Disagree
- ◯ No opinion
- ◯ Agree
- ◯ Strongly agree

g. I plan to work in health disparities in the following way after residency:

General

SECTION B: MEDICAL EDUCATION

A. Please indicate your level of agreement with the following statements regarding your medical education and knowledge about immigrants and refugees by checking the box that best represents your opinion.

a. I have received specialized training in immigrant and refugee health, tropical medicine, or cross-cultural health.

- ○ Strongly disagree
- ⊗ Disagree
- ○ No opinion
- ○ Agree
- ○ Strongly agree

c. I feel comfortable with my fund of knowledge regarding immigrant and refugee health.

- ○ Strongly disagree
- ⊗ Disagree
- ○ No opinion
- ○ Agree
- ○ Strongly agree

d. I would like to have further training in immigrant and refugee health.

- ○ Strongly disagree
- ○ Disagree
- ○ No opinion
- ⊗ Agree
- ○ Strongly agree

e. If you agree with the above, please indicate all the contexts in which you would like to receive this training:

- ☐ As part of my residency.
- ☐ A special program.
- ☐ As part of my fellowship.

SECTION C: Attitudes towards immigrant health

A. Please indicate your level of agreement with the following statements regarding immigrant and refugee health by checking the box that best represents your opinion.

a. I enjoy taking care of immigrants and refugees.

- ○ Never
- ○ Rarely
- ○ Sometimes
- ⊗ Usually
- ○ Always

b. Please indicate the reasons that you enjoy taking care of immigrants and refugees (may choose more than one).

- ☐ Tropical and other conditions not frequently diagnosed in US-born patients
- ☒ Learning about other cultures
- ☐ They don't complain as much
- ☒ Being able to hear their stories
- ☐ Their care is more complicated
- ☐ Their care is less complicated
- ☐ They are very appreciative of your help.
- ☐ They are extremely vulnerable
- ☐ Other:

c. Taking care of immigrants and refugees is more challenging than taking care of US born patients.

- ○ Never
- ○ Rarely
- ⊗ Sometimes
- ○ Usually
- ○ Always

d. Please mark all the challenges that you face as a provider when providing care to immigrants and refugees (may choose more than one):

- ☒ Language barriers
- ☐ Insurance barriers
- ☒ Cultural barriers
- ☒ Finding a professional interpreter
- ☐ Knowing how to work with a professional interpreter
- ☒ Time constraints
- ☐ My own knowledge related to tropical and travel medicine
- ☐ Transportation problems for the patient
- ☐ Patients not understanding treatment plan
- ☐ Patients not following treatment plan
- ☐ My lack of knowledge regarding the patient's culture
- ☐ Bias or stereotyping
- ☐ Other:

e. Please mark all of the challenges faced by immigrant and refugee populations when receiving healthcare that you have perceived or witnessed (may choose more than one):

- ☒ Language barriers
- ☐ Insurance barriers
- ☐ Cultural barriers
- ☐ Finding a professional interpreter
- ☐ Knowing how to work with a professional interpreter
- ☐ Time constraints
- ☐ Insufficiently trained health care providers
- ☐ Transportation problems for the patient
- ☐ Food insufficiency
- ☐ Need for child care
- ☒ Patients not understanding treatment plan
- ☐ Patients not following treatment plan
- ☐ My lack of knowledge regarding the patient's culture
- ☐ Bias or stereotyping
- ☒ Trust issues
- ☐ Other...

f. Rank how well immigrants and refugees understand the healthcare that you are trying to provide.

- ○ Significantly less than a US born individual
- ○ Less than a US born individual
- ⊗ Equivalent to a US born individual
- ○ More than a US born patient
- ○ Significantly more than a US born individual

g. Immigrants and refugees adhere to treatment plans and follow my recommendations.

- ○ Never
- ○ Rarely
- ○ Sometimes
- ⊗ Usually
- ○ Always

h. Immigrants and refugees should receive the same care and insurance coverage as US born patients.

- ○ Never
- ○ Rarely
- ○ Sometimes
- ○ Usually
- ⊗ Always

i. Immigrants and refugees who are undocumented should receive the same care and insurance coverage as US born patients.

- ○ Never
- ○ Rarely
- ○ Sometimes
- ○ Usually
- ⊗ Always

j. Every physician is professionally obligated to care for immigrants and refugees if they present to your clinic or hospital.

- ○ Strongly disagree
- ○ Disagree
- ○ No opinion
- ○ Agree
- ⊗ Strongly agree

k. Is healthcare a human right?
- ⊗ Yes
- ◯ No

B. If you wish, please tell us about what you enjoy or do not enjoy about immigrant and refugee health care and the greatest challenges you face in caring for this population.

____

SECTION D: DEMOGRAPHIC INFORMATION
Please answer the following questions by checking the box in front of the response choice that best describes you.

a. Your age?
- ◯ 20 to 24
- ◯ 25 to 29
- ◯ 30 to 34
- ⊗ 35 to 39
- ◯ 40 or older

b. Your gender?
- ⊗ Female
- ◯ Male
- ◯ Other

c. Are you Hispanic or Latino?
- ⊗ Yes
- ◯ No

d. What is your race? (Select one or more responses)
- ◯ American Indian or Alaska Native
- ◯ Asian (Please specify):
- ◯ Black or African American
- ◯ Native Hawaiian or Other Pacific Islander
- ⊗ White
- ◯ Other (Please specify):

e. Were you born in the United States?
- ⊗ Yes
- ◯ No

g. Your residency year?
- ◯ PGY1
- ◯ PGY2
- ⊗ PGY3
- ◯ PGY4
- ◯ PGY5

h. How would you classify your political ideology?
- ◯ Conservative
- ◯ Somewhat conservative
- ◯ Moderate
- ⊗ Somewhat liberal
- ◯ Liberal
- ◯ Other (Please specify):

i. Estimated level of educational debt?
- ◯ None
- ◯ Less than $50,000
- ◯ $50,000 - $100,000
- ⊗ $100,000 - $200,000
- ◯ $200,000 or more

j. Do you plan to subspecialize?
- ◯ Yes
- ⊗ No

k. Languages spoken?

- [x] English
- [x] Spanish
- [ ] French
- [ ] Hmong
- [ ] Somali
- [ ] Japanese
- [ ] Chinese
- [ ] Russian
- [ ] Ethiopian
- [ ] Other ___________

l. Are you in the Global Health Pathway?

- ○ Yes
- ⊗ No

m.▯ Did you earn your degree in the US?

- ⊗ Yes
- ○ No

n. What residency program are you in?

- ○ Internal Medicine
- ○ Med-Peds
- ○ Pediatrics
- ○ Family Practice
- ○ Neurology
- ○ Psychiatry
- ○ ObGyn
- ○ Neurosurgery
- ○ General Surgery
- ○ Orthopedic Surgery
- ○ Urology
- ○ Surgical sub-specialty (please specify in text box below)
- ⊗ Non-clinical specialty (radiology, pathology; please specify in text box below)

Anesthesiology

Medical Trainees' attitudes, knowledge, and experience with immigrant and refugee health

Response was added on 11/02/2013 9:05am.

SECTION A: Personal experience with immigrant and refugee health care.

A. Please indicate your level of agreement with the following statements regarding your personal experience with immigrant and refugee health care by checking the box that best represents your experience.

a. During my inpatient rotations, I take care of the following percentage of immigrant and refugee patients:

- ◯ None
- ◯ 0 -5%
- ⊗ 5-10%
- ◯ 10-25%
- ◯ > 25%

b. During my outpatient rotations, I take care of the following percentage of immigrant and refugee patients:

- ◯ None
- ◯ 0-10%
- ⊗ 10 -25%
- ◯ 25-50%
- ◯ 50-75%
- ◯ >75%

c. I would like to take care of more immigrant and refugee patients.

- ◯ Strongly disagree
- ◯ Disagree
- ⊗ No opinion
- ◯ Agree
- ◯ Strongly agree

d. I plan to take care of immigrants and refugees when I finish residency.

- ◯ Strongly disagree
- ◯ Disagree
- ◯ No opinion
- ⊗ Agree
- ◯ Strongly agree

e. I plan to do short term (< 6 months) international work when I finish residency.

- ◯ Strongly disagree
- ◯ Disagree
- ◯ No opinion
- ⊗ Agree
- ◯ Strongly agree

f. I plan to do long term (>6 months) international work when I finish residency.

- ◯ Strongly disagree
- ◯ Disagree
- ◯ No opinion
- ◯ Agree
- ⊗ Strongly agree

g. I plan to work in health disparities in the following way after residency:

Fellowship

SECTION B: MEDICAL EDUCATION

A. Please indicate your level of agreement with the following statements regarding your medical education and knowledge about immigrants and refugees by checking the box that best represents your opinion.

a. I have received specialized training in immigrant and refugee health, tropical medicine, or cross-cultural health.

- ◯ Strongly disagree
- ◯ Disagree
- ◯ No opinion
- ⊗ Agree
- ◯ Strongly agree

b. If you have received specialized training in immigrant and refugee health, tropical medicine, or cross-cultural health, please indicate all the contexts in which you received this training:

- ☐ As an undergraduate.
- ☒ As a medical student.
- ☒ As part of my residency.
- ☐ A special program.
- ☐ As part of my fellowship.
- ☐ As part of a degree program (e.g. MPH)
- ☐ Other:

c. I feel comfortable with my fund of knowledge regarding immigrant and refugee health.

- ◯ Strongly disagree
- ◯ Disagree
- ⊗ No opinion
- ◯ Agree
- ◯ Strongly agree

d. I would like to have further training in immigrant and refugee health.

- ◯ Strongly disagree
- ◯ Disagree
- ◯ No opinion
- ⊗ Agree
- ◯ Strongly agree

e. If you agree with the above, please indicate all the contexts in which you would like to receive this training:
□□

- ☒ As part of my residency.
- ☐ A special program.
- ☒ As part of my fellowship.

SECTION C: Attitudes towards immigrant health

A.□Please indicate your level of agreement with the following statements regarding immigrant and refugee health by checking the box that best represents your opinion.

a. I enjoy taking care of immigrants and refugees.

- ◯ Never
- ◯ Rarely
- ◯ Sometimes
- ⊗ Usually
- ◯ Always

b. Please indicate the reasons that you enjoy taking care of immigrants and refugees (may choose more than one).

- ☐ Tropical and other conditions not frequently diagnosed in US-born patients
- ☒ Learning about other cultures
- ☐ They don't complain as much
- ☐ Being able to hear their stories
- ☐ Their care is more complicated
- ☐ Their care is less complicated
- ☐ They are very appreciative of your help.
- ☐ They are extremely vulnerable
- ☐ Other:

c. Taking care of immigrants and refugees is more challenging than taking care of US born patients.

- ◯ Never
- ◯ Rarely
- ◯ Sometimes
- ⊗ Usually
- ◯ Always

d. Please mark all the challenges that you face as a provider when providing care to immigrants and refugees (may choose more than one):

- [x] Language barriers
- [ ] Insurance barriers
- [x] Cultural barriers
- [x] Finding a professional interpreter
- [ ] Knowing how to work with a professional interpreter
- [x] Time constraints
- [ ] My own knowledge related to tropical and travel medicine
- [ ] Transportation problems for the patient
- [ ] Patients not understanding treatment plan
- [ ] Patients not following treatment plan
- [x] My lack of knowledge regarding the patient's culture
- [ ] Bias or stereotyping
- [ ] Other:

e. Please mark all of the challenges faced by immigrant and refugee populations when receiving healthcare that you have perceived or witnessed (may choose more than one):

- [x] Language barriers
- [x] Insurance barriers
- [x] Cultural barriers
- [x] Finding a professional interpreter
- [x] Knowing how to work with a professional interpreter
- [x] Time constraints
- [ ] Insufficiently trained health care providers
- [x] Transportation problems for the patient
- [ ] Food insufficiency
- [x] Need for child care
- [x] Patients not understanding treatment plan
- [x] Patients not following treatment plan
- [x] My lack of knowledge regarding the patient's culture
- [ ] Bias or stereotyping
- [x] Trust issues
- [ ] Other...

f. Rank how well immigrants and refugees understand the healthcare that you are trying to provide.

- ( ) Significantly less than a US born individual
- (x) Less than a US born individual
- ( ) Equivalent to a US born individual
- ( ) More than a US born patient
- ( ) Significantly more than a US born individual

g. Immigrants and refugees adhere to treatment plans and follow my recommendations.

- ( ) Never
- ( ) Rarely
- (x) Sometimes
- ( ) Usually
- ( ) Always

h. Immigrants and refugees should receive the same care and insurance coverage as US born patients.

- ( ) Never
- ( ) Rarely
- (x) Sometimes
- ( ) Usually
- ( ) Always

i. Immigrants and refugees who are undocumented should receive the same care and insurance coverage as US born patients.

- ( ) Never
- ( ) Rarely
- (x) Sometimes
- ( ) Usually
- ( ) Always

j. Every physician is professionally obligated to care for immigrants and refugees if they present to your clinic or hospital.

- ( ) Strongly disagree
- ( ) Disagree
- (x) No opinion
- ( ) Agree
- ( ) Strongly agree

k. Is healthcare a human right?
- ⊗ Yes
- ◯ No

B. If you wish, please tell us about what you enjoy or do not enjoy about immigrant and refugee health care and the greatest challenges you face in caring for this population. __________________________________

SECTION D: DEMOGRAPHIC INFORMATION
Please answer the following questions by checking the box in front of the response choice that best describes you.

a. Your age?
- ◯ 20 to 24
- ⊗ 25 to 29
- ◯ 30 to 34
- ◯ 35 to 39
- ◯ 40 or older

b. Your gender?
- ◯ Female
- ⊗ Male
- ◯ Other

c. Are you Hispanic or Latino?
- ◯ Yes
- ⊗ No

d. What is your race? (Select one or more responses)
- ⊗ American Indian or Alaska Native
- ◯ Asian (Please specify):
- ◯ Black or African American
- ◯ Native Hawaiian or Other Pacific Islander
- ◯ White
- ◯ Other (Please specify):

e. Were you born in the United States?
- ⊗ Yes
- ◯ No

g. Your residency year?
- ◯ PGY1
- ◯ PGY2
- ⊗ PGY3
- ◯ PGY4
- ◯ PGY5

h. How would you classify your political ideology?
- ◯ Conservative
- ◯ Somewhat conservative
- ⊗ Moderate
- ◯ Somewhat liberal
- ◯ Liberal
- ◯ Other (Please specify):

i. Estimated level of educational debt?
- ◯ None
- ◯ Less than $50,000
- ◯ $50,000 - $100,000
- ◯ $100,000 - $200,000
- ⊗ $200,000 or more

j. Do you plan to subspecialize?
- ⊗ Yes
- ◯ No

k. Languages spoken?

☒ English
☐ Spanish
☐ French
☐ Hmong
☐ Somali
☐ Japanese
☐ Chinese
☐ Russian
☐ Ethiopian
☐ Other ___________

l. Are you in the Global Health Pathway?

○ Yes
⊗ No

m.□ Did you earn your degree in the US?

⊗ Yes
○ No

n. What residency program are you in?

○ Internal Medicine
○ Med-Peds
○ Pediatrics
○ Family Practice
○ Neurology
○ Psychiatry
○ ObGyn
○ Neurosurgery
○ General Surgery
○ Orthopedic Surgery
○ Urology
○ Surgical sub-specialty (please specify in text box below)
○ Non-clinical specialty (radiology, pathology; please specify in text box below)

Medical Trainees' attitudes, knowledge, and experience with immigrant and refugee health

Response was added on 11/02/2013 9:29am.

SECTION A: Personal experience with immigrant and refugee health care.

A. Please indicate your level of agreement with the following statements regarding your personal experience with immigrant and refugee health care by checking the box that best represents your experience.

a. During my inpatient rotations, I take care of the following percentage of immigrant and refugee patients:

- ◯ None
- ◯ 0 -5%
- ⊗ 5-10%
- ◯ 10-25%
- ◯ > 25%

b. During my outpatient rotations, I take care of the following percentage of immigrant and refugee patients:

- ◯ None
- ⊗ 0-10%
- ◯ 10 -25%
- ◯ 25-50%
- ◯ 50-75%
- ◯ >75%

c. I would like to take care of more immigrant and refugee patients.

- ◯ Strongly disagree
- ◯ Disagree
- ⊗ No opinion
- ◯ Agree
- ◯ Strongly agree

d. I plan to take care of immigrants and refugees when I finish residency.

- ◯ Strongly disagree
- ◯ Disagree
- ◯ No opinion
- ⊗ Agree
- ◯ Strongly agree

e. I plan to do short term (< 6 months) international work when I finish residency.

- ◯ Strongly disagree
- ◯ Disagree
- ◯ No opinion
- ◯ Agree
- ⊗ Strongly agree

f. I plan to do long term (>6 months) international work when I finish residency.

- ◯ Strongly disagree
- ◯ Disagree
- ⊗ No opinion
- ◯ Agree
- ◯ Strongly agree

g. I plan to work in health disparities in the following way after residency:

Serving within the underserved population. Short term international work.

SECTION B: MEDICAL EDUCATION

A. Please indicate your level of agreement with the following statements regarding your medical education and knowledge about immigrants and refugees by checking the box that best represents your opinion.

a. I have received specialized training in immigrant and refugee health, tropical medicine, or cross-cultural health.

◯ Strongly disagree
◯ Disagree
⊗ No opinion
◯ Agree
◯ Strongly agree

c. I feel comfortable with my fund of knowledge regarding immigrant and refugee health.

◯ Strongly disagree
⊗ Disagree
◯ No opinion
◯ Agree
◯ Strongly agree

d. I would like to have further training in immigrant and refugee health.

◯ Strongly disagree
◯ Disagree
◯ No opinion
⊗ Agree
◯ Strongly agree

e. If you agree with the above, please indicate all the contexts in which you would like to receive this training:

☒ As part of my residency.
☐ A special program.
☐ As part of my fellowship.

SECTION C: Attitudes towards immigrant health

A. Please indicate your level of agreement with the following statements regarding immigrant and refugee health by checking the box that best represents your opinion.

a. I enjoy taking care of immigrants and refugees.

◯ Never
◯ Rarely
⊗ Sometimes
◯ Usually
◯ Always

b. Please indicate the reasons that you enjoy taking care of immigrants and refugees (may choose more than one).

☒ Tropical and other conditions not frequently diagnosed in US-born patients
☒ Learning about other cultures
☐ They don't complain as much
☐ Being able to hear their stories
☐ Their care is more complicated
☐ Their care is less complicated
☒ They are very appreciative of your help.
☒ They are extremely vulnerable
☐ Other:

c. Taking care of immigrants and refugees is more challenging than taking care of US born patients.

◯ Never
◯ Rarely
◯ Sometimes
⊗ Usually
◯ Always

d. Please mark all the challenges that you face as a provider when providing care to immigrants and refugees (may choose more than one):

- ☒ Language barriers
- ☐ Insurance barriers
- ☒ Cultural barriers
- ☒ Finding a professional interpreter
- ☐ Knowing how to work with a professional interpreter
- ☒ Time constraints
- ☒ My own knowledge related to tropical and travel medicine
- ☐ Transportation problems for the patient
- ☐ Patients not understanding treatment plan
- ☐ Patients not following treatment plan
- ☒ My lack of knowledge regarding the patient's culture
- ☐ Bias or stereotyping
- ☐ Other:

e. Please mark all of the challenges faced by immigrant and refugee populations when receiving healthcare that you have perceived or witnessed (may choose more than one):

- ☒ Language barriers
- ☒ Insurance barriers
- ☒ Cultural barriers
- ☒ Finding a professional interpreter
- ☒ Knowing how to work with a professional interpreter
- ☐ Time constraints
- ☒ Insufficiently trained health care providers
- ☐ Transportation problems for the patient
- ☐ Food insufficiency
- ☐ Need for child care
- ☒ Patients not understanding treatment plan
- ☒ Patients not following treatment plan
- ☐ My lack of knowledge regarding the patient's culture
- ☐ Bias or stereotyping
- ☒ Trust issues
- ☐ Other...

f. Rank how well immigrants and refugees understand the healthcare that you are trying to provide.

- ○ Significantly less than a US born individual
- ⊗ Less than a US born individual
- ○ Equivalent to a US born individual
- ○ More than a US born patient
- ○ Significantly more than a US born individual

g. Immigrants and refugees adhere to treatment plans and follow my recommendations.

- ○ Never
- ○ Rarely
- ⊗ Sometimes
- ○ Usually
- ○ Always

h. Immigrants and refugees should receive the same care and insurance coverage as US born patients.

- ○ Never
- ○ Rarely
- ○ Sometimes
- ○ Usually
- ⊗ Always

i. Immigrants and refugees who are undocumented should receive the same care and insurance coverage as US born patients.

- ○ Never
- ○ Rarely
- ○ Sometimes
- ○ Usually
- ○ Always

j. Every physician is professionally obligated to care for immigrants and refugees if they present to your clinic or hospital.

- ○ Strongly disagree
- ○ Disagree
- ○ No opinion
- ○ Agree
- ⊗ Strongly agree

k. Is healthcare a human right?	⊗ Yes<br>◯ No
B. If you wish, please tell us about what you enjoy or do not enjoy about immigrant and refugee health care and the greatest challenges you face in caring for this population.	I think one of the challenges that I face is an inner struggle with the fact that using an interpreter takes more time, which makes their care inherently more challenging. Which means when I get an admit that is a non-english speaking immigrant, I have to fight the feeling of wishing they spoke English.

SECTION D: DEMOGRAPHIC INFORMATION
Please answer the following questions by checking the box in front of the response choice that best describes you.

a. Your age?	◯ 20 to 24<br>⊗ 25 to 29<br>◯ 30 to 34<br>◯ 35 to 39<br>◯ 40 or older
b. Your gender?	◯ Female<br>⊗ Male<br>◯ Other
c.Are you Hispanic or Latino?	◯ Yes<br>⊗ No
d. What is your race? (Select one or more responses)	◯ American Indian or Alaska Native<br>◯ Asian (Please specify):<br>◯ Black or African American<br>◯ Native Hawaiian or Other Pacific Islander<br>⊗ White<br>◯ Other (Please specify):
e.Were you born in the United States?	⊗ Yes<br>◯ No
g. Your residency year?	◯ PGY1<br>⊗ PGY2<br>◯ PGY3<br>◯ PGY4<br>◯ PGY5
h. How would you classify your political ideology?	◯ Conservative<br>◯ Somewhat conservative<br>⊗ Moderate<br>◯ Somewhat liberal<br>◯ Liberal<br>◯ Other (Please specify):
i. Estimated level of educational debt?	◯ None<br>◯ Less than $50,000<br>◯ $50,000 - $100,000<br>⊗ $100,000 - $200,000<br>◯ $200,000 or more
j.Do you plan to subspecialize?	◯ Yes<br>⊗ No

k. Languages spoken?

- ☒ English
- ☐ Spanish
- ☐ French
- ☐ Hmong
- ☐ Somali
- ☐ Japanese
- ☐ Chinese
- ☐ Russian
- ☐ Ethiopian
- ☐ Other __________

l. Are you in the Global Health Pathway?

- ○ Yes
- ⊗ No

m. Did you earn your degree in the US?

- ⊗ Yes
- ○ No

n. What residency program are you in?

- ⊗ Internal Medicine
- ○ Med-Peds
- ○ Pediatrics
- ○ Family Practice
- ○ Neurology
- ○ Psychiatry
- ○ ObGyn
- ○ Neurosurgery
- ○ General Surgery
- ○ Orthopedic Surgery
- ○ Urology
- ○ Surgical sub-specialty (please specify in text box below)
- ○ Non-clinical specialty (radiology, pathology; please specify in text box below)

Medical Trainees' attitudes, knowledge, and experience with immigrant and refugee health

Response was added on 11/02/2013 9:34am.

SECTION A: Personal experience with immigrant and refugee health care.

A. Please indicate your level of agreement with the following statements regarding your personal experience with immigrant and refugee health care by checking the box that best represents your experience.

a. During my inpatient rotations, I take care of the following percentage of immigrant and refugee patients:
- ○ None
- ○ 0 -5%
- ⊗ 5-10%
- ○ 10-25%
- ○ > 25%

b. During my outpatient rotations, I take care of the following percentage of immigrant and refugee patients:
- ○ None
- ○ 0-10%
- ⊗ 10 -25%
- ○ 25-50%
- ○ 50-75%
- ○ >75%

c. I would like to take care of more immigrant and refugee patients.
- ○ Strongly disagree
- ⊗ Disagree
- ○ No opinion
- ○ Agree
- ○ Strongly agree

d. I plan to take care of immigrants and refugees when I finish residency.
- ○ Strongly disagree
- ⊗ Disagree
- ○ No opinion
- ○ Agree
- ○ Strongly agree

e. I plan to do short term (< 6 months) international work when I finish residency.
- ○ Strongly disagree
- ○ Disagree
- ○ No opinion
- ⊗ Agree
- ○ Strongly agree

f. I plan to do long term (>6 months) international work when I finish residency.
- ○ Strongly disagree
- ⊗ Disagree
- ○ No opinion
- ○ Agree
- ○ Strongly agree

g. I plan to work in health disparities in the following way after residency: hospital based practice

SECTION B: MEDICAL EDUCATION

A. Please indicate your level of agreement with the following statements regarding your medical education and knowledge about immigrants and refugees by checking the box that best represents your opinion.

a. I have received specialized training in immigrant and refugee health, tropical medicine, or cross-cultural health.

- ◯ Strongly disagree
- ⊗ Disagree
- ◯ No opinion
- ◯ Agree
- ◯ Strongly agree

c. I feel comfortable with my fund of knowledge regarding immigrant and refugee health.

- ◯ Strongly disagree
- ◯ Disagree
- ◯ No opinion
- ⊗ Agree
- ◯ Strongly agree

d. I would like to have further training in immigrant and refugee health.

- ◯ Strongly disagree
- ◯ Disagree
- ⊗ No opinion
- ◯ Agree
- ◯ Strongly agree

SECTION C: Attitudes towards immigrant health

A.Please indicate your level of agreement with the following statements regarding immigrant and refugee health by checking the box that best represents your opinion.

a. I enjoy taking care of immigrants and refugees.

- ◯ Never
- ◯ Rarely
- ⊗ Sometimes
- ◯ Usually
- ◯ Always

b. Please indicate the reasons that you enjoy taking care of immigrants and refugees (may choose more than one).

- ☐ Tropical and other conditions not frequently diagnosed in US-born patients
- ☐ Learning about other cultures
- ☐ They don't complain as much
- ☐ Being able to hear their stories
- ☐ Their care is more complicated
- ☐ Their care is less complicated
- ☒ They are very appreciative of your help.
- ☐ They are extremely vulnerable
- ☐ Other:

c. Taking care of immigrants and refugees is more challenging than taking care of US born patients.

- ◯ Never
- ◯ Rarely
- ◯ Sometimes
- ⊗ Usually
- ◯ Always

d. Please mark all the challenges that you face as a provider when providing care to immigrants and refugees (may choose more than one):

- ☒ Language barriers
- ☒ Insurance barriers
- ☒ Cultural barriers
- ☒ Finding a professional interpreter
- ☐ Knowing how to work with a professional interpreter
- ☒ Time constraints
- ☐ My own knowledge related to tropical and travel medicine
- ☒ Transportation problems for the patient
- ☒ Patients not understanding treatment plan
- ☒ Patients not following treatment plan
- ☐ My lack of knowledge regarding the patient's culture
- ☒ Bias or stereotyping
- ☒ Other:

usually complicated social situation that takes much time to fix rhater then focusing on medical problem

e. Please mark all of the challenges faced by immigrant and refugee populations when receiving healthcare that you have perceived or witnessed (may choose more than one):

- ☒ Language barriers
- ☒ Insurance barriers
- ☒ Cultural barriers
- ☐ Finding a professional interpreter
- ☐ Knowing how to work with a professional interpreter
- ☒ Time constraints
- ☐ Insufficiently trained health care providers
- ☐ Transportation problems for the patient
- ☐ Food insufficiency
- ☐ Need for child care
- ☐ Patients not understanding treatment plan
- ☒ Patients not following treatment plan
- ☐ My lack of knowledge regarding the patient's culture
- ☒ Bias or stereotyping
- ☐ Trust issues
- ☐ Other...

f. Rank how well immigrants and refugees understand the healthcare that you are trying to provide.

- ○ Significantly less than a US born individual
- ⊗ Less than a US born individual
- ○ Equivalent to a US born individual
- ○ More than a US born patient
- ○ Significantly more than a US born individual

g. Immigrants and refugees adhere to treatment plans and follow my recommendations.

- ○ Never
- ○ Rarely
- ⊗ Sometimes
- ○ Usually
- ○ Always

h. Immigrants and refugees should receive the same care and insurance coverage as US born patients.

- ○ Never
- ○ Rarely
- ○ Sometimes
- ⊗ Usually
- ○ Always

i. Immigrants and refugees who are undocumented should receive the same care and insurance coverage as US born patients.

- ○ Never
- ○ Rarely
- ○ Sometimes
- ⊗ Usually
- ○ Always

j. Every physician is professionally obligated to care for immigrants and refugees if they present to your clinic or hospital.

- ○ Strongly disagree
- ○ Disagree
- ○ No opinion
- ○ Agree
- ⊗ Strongly agree

k. Is healthcare a human right?

- ⊗ Yes
- ○ No

B. If you wish, please tell us about what you enjoy or do not enjoy about immigrant and refugee health care and the greatest challenges you face in caring for this population.

____

SECTION D: DEMOGRAPHIC INFORMATION
Please answer the following questions by checking the box in front of the response choice that best describes you.

a. Your age?
- ◯ 20 to 24
- ◯ 25 to 29
- ◯ 30 to 34
- ⊗ 35 to 39
- ◯ 40 or older

b. Your gender?
- ⊗ Female
- ◯ Male
- ◯ Other

c. Are you Hispanic or Latino?
- ◯ Yes
- ⊗ No

d. What is your race? (Select one or more responses)
- ◯ American Indian or Alaska Native
- ◯ Asian (Please specify):
- ◯ Black or African American
- ◯ Native Hawaiian or Other Pacific Islander
- ◯ White
- ⊗ Other (Please specify):

____

e. Were you born in the United States?
- ◯ Yes
- ⊗ No

f. If not, in what country were you born? __________________________________

g. Your residency year?
- ⊗ PGY1
- ◯ PGY2
- ◯ PGY3
- ◯ PGY4
- ◯ PGY5

h. How would you classify your political ideology?
- ◯ Conservative
- ◯ Somewhat conservative
- ◯ Moderate
- ◯ Somewhat liberal
- ⊗ Liberal
- ◯ Other (Please specify):

i. Estimated level of educational debt?
- ◯ None
- ◯ Less than $50,000
- ⊗ $50,000 - $100,000
- ◯ $100,000 - $200,000
- ◯ $200,000 or more

j. Do you plan to subspecialize?
- ⊗ Yes
- ◯ No

k. Languages spoken?
- ☒ English
- ☐ Spanish
- ☒ French
- ☐ Hmong
- ☐ Somali
- ☐ Japanese
- ☐ Chinese
- ☒ Russian
- ☐ Ethiopian
- ☒ Other ___________

arabic

l. Are you in the Global Health Pathway?

- ◯ Yes
- ⊗ No

m. Did you earn your degree in the US?

- ◯ Yes
- ⊗ No

n. What residency program are you in?

- ◯ Internal Medicine
- ◯ Med-Peds
- ◯ Pediatrics
- ◯ Family Practice
- ⊗ Neurology
- ◯ Psychiatry
- ◯ ObGyn
- ◯ Neurosurgery
- ◯ General Surgery
- ◯ Orthopedic Surgery
- ◯ Urology
- ◯ Surgical sub-specialty (please specify in text box below)
- ◯ Non-clinical specialty (radiology, pathology; please specify in text box below)

Medical Trainees' attitudes, knowledge, and experience with immigrant and refugee health

Response was added on 11/02/2013 9:47am.

SECTION A: Personal experience with immigrant and refugee health care.

A. Please indicate your level of agreement with the following statements regarding your personal experience with immigrant and refugee health care by checking the box that best represents your experience.

a. During my inpatient rotations, I take care of the following percentage of immigrant and refugee patients:

- ○ None
- ○ 0 -5%
- ○ 5-10%
- ⊗ 10-25%
- ○ > 25%

b. During my outpatient rotations, I take care of the following percentage of immigrant and refugee patients:

- ○ None
- ⊗ 0-10%
- ○ 10 -25%
- ○ 25-50%
- ○ 50-75%
- ○ >75%

c. I would like to take care of more immigrant and refugee patients.

- ○ Strongly disagree
- ○ Disagree
- ⊗ No opinion
- ○ Agree
- ○ Strongly agree

d. I plan to take care of immigrants and refugees when I finish residency.

- ○ Strongly disagree
- ○ Disagree
- ⊗ No opinion
- ○ Agree
- ○ Strongly agree

e. I plan to do short term (< 6 months) international work when I finish residency.

- ⊗ Strongly disagree
- ○ Disagree
- ○ No opinion
- ○ Agree
- ○ Strongly agree

f. I plan to do long term (>6 months) international work when I finish residency.

- ⊗ Strongly disagree
- ○ Disagree
- ○ No opinion
- ○ Agree
- ○ Strongly agree

g. I plan to work in health disparities in the following way after residency:

Rural

SECTION B: MEDICAL EDUCATION

A. Please indicate your level of agreement with the following statements regarding your medical education and knowledge about immigrants and refugees by checking the box that best represents your opinion.

a. I have received specialized training in immigrant and refugee health, tropical medicine, or cross-cultural health.

- ◯ Strongly disagree
- ⊗ Disagree
- ◯ No opinion
- ◯ Agree
- ◯ Strongly agree

c. I feel comfortable with my fund of knowledge regarding immigrant and refugee health.

- ◯ Strongly disagree
- ◯ Disagree
- ⊗ No opinion
- ◯ Agree
- ◯ Strongly agree

d. I would like to have further training in immigrant and refugee health.

- ◯ Strongly disagree
- ◯ Disagree
- ◯ No opinion
- ⊗ Agree
- ◯ Strongly agree

e. If you agree with the above, please indicate all the contexts in which you would like to receive this training:
□□

- ☒ As part of my residency.
- ☐ A special program.
- ☐ As part of my fellowship.

SECTION C: Attitudes towards immigrant health

A.□Please indicate your level of agreement with the following statements regarding immigrant and refugee health by checking the box that best represents your opinion.

a. I enjoy taking care of immigrants and refugees.

- ◯ Never
- ◯ Rarely
- ⊗ Sometimes
- ◯ Usually
- ◯ Always

b. Please indicate the reasons that you enjoy taking care of immigrants and refugees (may choose more than one).

- ☐ Tropical and other conditions not frequently diagnosed in US-born patients
- ☒ Learning about other cultures
- ☐ They don't complain as much
- ☐ Being able to hear their stories
- ☐ Their care is more complicated
- ☐ Their care is less complicated
- ☐ They are very appreciative of your help.
- ☒ They are extremely vulnerable
- ☐ Other:

c. Taking care of immigrants and refugees is more challenging than taking care of US born patients.

- ◯ Never
- ◯ Rarely
- ◯ Sometimes
- ⊗ Usually
- ◯ Always

d. Please mark all the challenges that you face as a provider when providing care to immigrants and refugees (may choose more than one):

☒ Language barriers
☐ Insurance barriers
☒ Cultural barriers
☒ Finding a professional interpreter
☐ Knowing how to work with a professional interpreter
☐ Time constraints
☒ My own knowledge related to tropical and travel medicine
☐ Transportation problems for the patient
☒ Patients not understanding treatment plan
☒ Patients not following treatment plan
☒ My lack of knowledge regarding the patient's culture
☐ Bias or stereotyping
☐ Other:

e. Please mark all of the challenges faced by immigrant and refugee populations when receiving healthcare that you have perceived or witnessed (may choose more than one):

☒ Language barriers
☒ Insurance barriers
☒ Cultural barriers
☒ Finding a professional interpreter
☐ Knowing how to work with a professional interpreter
☒ Time constraints
☒ Insufficiently trained health care providers
☒ Transportation problems for the patient
☒ Food insufficiency
☒ Need for child care
☒ Patients not understanding treatment plan
☒ Patients not following treatment plan
☒ My lack of knowledge regarding the patient's culture
☒ Bias or stereotyping
☐ Trust issues
☐ Other...

f. Rank how well immigrants and refugees understand the healthcare that you are trying to provide.

○ Significantly less than a US born individual
⊗ Less than a US born individual
○ Equivalent to a US born individual
○ More than a US born patient
○ Significantly more than a US born individual

g. Immigrants and refugees adhere to treatment plans and follow my recommendations.

○ Never
○ Rarely
⊗ Sometimes
○ Usually
○ Always

h. Immigrants and refugees should receive the same care and insurance coverage as US born patients.

○ Never
○ Rarely
○ Sometimes
⊗ Usually
○ Always

i. Immigrants and refugees who are undocumented should receive the same care and insurance coverage as US born patients.

○ Never
⊗ Rarely
○ Sometimes
○ Usually
○ Always

j. Every physician is professionally obligated to care for immigrants and refugees if they present to your clinic or hospital.

○ Strongly disagree
○ Disagree
○ No opinion
⊗ Agree
○ Strongly agree

k. Is healthcare a human right?

⊗ Yes
◯ No

B. If you wish, please tell us about what you enjoy or do not enjoy about immigrant and refugee health care and the greatest challenges you face in caring for this population.

____

SECTION D: DEMOGRAPHIC INFORMATION
Please answer the following questions by checking the box in front of the response choice that best describes you.

a. Your age?

◯ 20 to 24
⊗ 25 to 29
◯ 30 to 34
◯ 35 to 39
◯ 40 or older

b. Your gender?

⊗ Female
◯ Male
◯ Other

c. Are you Hispanic or Latino?

◯ Yes
⊗ No

d. What is your race? (Select one or more responses)

◯ American Indian or Alaska Native
◯ Asian (Please specify):
◯ Black or African American
◯ Native Hawaiian or Other Pacific Islander
⊗ White
◯ Other (Please specify):

e. Were you born in the United States?

⊗ Yes
◯ No

g. Your residency year?

◯ PGY1
⊗ PGY2
◯ PGY3
◯ PGY4
◯ PGY5

h. How would you classify your political ideology?

◯ Conservative
◯ Somewhat conservative
◯ Moderate
⊗ Somewhat liberal
◯ Liberal
◯ Other (Please specify):

i. Estimated level of educational debt?

◯ None
◯ Less than $50,000
◯ $50,000 - $100,000
⊗ $100,000 - $200,000
◯ $200,000 or more

j. Do you plan to subspecialize?

◯ Yes
⊗ No

k. Languages spoken?

- [x] English
- [x] Spanish
- [ ] French
- [ ] Hmong
- [ ] Somali
- [ ] Japanese
- [ ] Chinese
- [ ] Russian
- [ ] Ethiopian
- [ ] Other ___________

l. Are you in the Global Health Pathway?

- ○ Yes
- ⊗ No

m. Did you earn your degree in the US?

- ⊗ Yes
- ○ No

n. What residency program are you in?

- ○ Internal Medicine
- ○ Med-Peds
- ○ Pediatrics
- ⊗ Family Practice
- ○ Neurology
- ○ Psychiatry
- ○ ObGyn
- ○ Neurosurgery
- ○ General Surgery
- ○ Orthopedic Surgery
- ○ Urology
- ○ Surgical sub-specialty (please specify in text box below)
- ○ Non-clinical specialty (radiology, pathology; please specify in text box below)

Medical Trainees' attitudes, knowledge, and experience with immigrant and refugee health

Response was added on 11/02/2013 10:18am.

SECTION A: Personal experience with immigrant and refugee health care.

A. Please indicate your level of agreement with the following statements regarding your personal experience with immigrant and refugee health care by checking the box that best represents your experience.

a. During my inpatient rotations, I take care of the following percentage of immigrant and refugee patients:	◯ None<br>◯ 0 -5%<br>⊗ 5-10%<br>⊗ 10-25%<br>◯ > 25%
b. During my outpatient rotations, I take care of the following percentage of immigrant and refugee patients:	◯ None<br>⊗ 0-10%<br>◯ 10 -25%<br>◯ 25-50%<br>◯ 50-75%<br>◯ >75%
c. I would like to take care of more immigrant and refugee patients.	◯ Strongly disagree<br>◯ Disagree<br>⊗ No opinion<br>◯ Agree<br>◯ Strongly agree
d. I plan to take care of immigrants and refugees when I finish residency.	◯ Strongly disagree<br>◯ Disagree<br>⊗ No opinion<br>◯ Agree<br>◯ Strongly agree
e. I plan to do short term (< 6 months) international work when I finish residency.	◯ Strongly disagree<br>◯ Disagree<br>⊗ No opinion<br>◯ Agree<br>◯ Strongly agree
f. I plan to do long term (>6 months) international work when I finish residency.	◯ Strongly disagree<br>⊗ Disagree<br>◯ No opinion<br>◯ Agree<br>◯ Strongly agree
g. I plan to work in health disparities in the following way after residency:	undecided

SECTION B: MEDICAL EDUCATION

A. Please indicate your level of agreement with the following statements regarding your medical education and knowledge about immigrants and refugees by checking the box that best represents your opinion.

a. I have received specialized training in immigrant and refugee health, tropical medicine, or cross-cultural health.

- ◯ Strongly disagree
- ◯ Disagree
- ◯ No opinion
- ⊗ Agree
- ◯ Strongly agree

b. If you have received specialized training in immigrant and refugee health, tropical medicine, or cross-cultural health, please indicate all the contexts in which you received this training:

- ☐ As an undergraduate.
- ☐ As a medical student.
- ☒ As part of my residency.
- ☐ A special program.
- ☐ As part of my fellowship.
- ☐ As part of a degree program (e.g. MPH)
- ☐ Other:

c. I feel comfortable with my fund of knowledge regarding immigrant and refugee health.

- ◯ Strongly disagree
- ⊗ Disagree
- ◯ No opinion
- ◯ Agree
- ◯ Strongly agree

d. I would like to have further training in immigrant and refugee health.

- ◯ Strongly disagree
- ◯ Disagree
- ◯ No opinion
- ⊗ Agree
- ◯ Strongly agree

e. If you agree with the above, please indicate all the contexts in which you would like to receive this training:
□□

- ☒ As part of my residency.
- ☐ A special program.
- ☐ As part of my fellowship.

SECTION C: Attitudes towards immigrant health

A.□Please indicate your level of agreement with the following statements regarding immigrant and refugee health by checking the box that best represents your opinion.

a. I enjoy taking care of immigrants and refugees.

- ◯ Never
- ◯ Rarely
- ◯ Sometimes
- ⊗ Usually
- ◯ Always

b. Please indicate the reasons that you enjoy taking care of immigrants and refugees (may choose more than one).

- ☒ Tropical and other conditions not frequently diagnosed in US-born patients
- ☒ Learning about other cultures
- ☐ They don't complain as much
- ☒ Being able to hear their stories
- ☐ Their care is more complicated
- ☐ Their care is less complicated
- ☐ They are very appreciative of your help.
- ☐ They are extremely vulnerable
- ☐ Other:

c. Taking care of immigrants and refugees is more challenging than taking care of US born patients.

- ◯ Never
- ◯ Rarely
- ◯ Sometimes
- ⊗ Usually
- ◯ Always

d. Please mark all the challenges that you face as a provider when providing care to immigrants and refugees (may choose more than one):

- ☒ Language barriers
- ☐ Insurance barriers
- ☒ Cultural barriers
- ☒ Finding a professional interpreter
- ☐ Knowing how to work with a professional interpreter
- ☒ Time constraints
- ☐ My own knowledge related to tropical and travel medicine
- ☐ Transportation problems for the patient
- ☐ Patients not understanding treatment plan
- ☐ Patients not following treatment plan
- ☐ My lack of knowledge regarding the patient's culture
- ☐ Bias or stereotyping
- ☐ Other:

e. Please mark all of the challenges faced by immigrant and refugee populations when receiving healthcare that you have perceived or witnessed (may choose more than one):

- ☒ Language barriers
- ☒ Insurance barriers
- ☒ Cultural barriers
- ☒ Finding a professional interpreter
- ☒ Knowing how to work with a professional interpreter
- ☐ Time constraints
- ☐ Insufficiently trained health care providers
- ☒ Transportation problems for the patient
- ☐ Food insufficiency
- ☐ Need for child care
- ☒ Patients not understanding treatment plan
- ☐ Patients not following treatment plan
- ☐ My lack of knowledge regarding the patient's culture
- ☐ Bias or stereotyping
- ☐ Trust issues
- ☐ Other...

f. Rank how well immigrants and refugees understand the healthcare that you are trying to provide.

- ◯ Significantly less than a US born individual
- ◯ Less than a US born individual
- ⊗ Equivalent to a US born individual
- ◯ More than a US born patient
- ◯ Significantly more than a US born individual

g. Immigrants and refugees adhere to treatment plans and follow my recommendations.

- ◯ Never
- ◯ Rarely
- ◯ Sometimes
- ⊗ Usually
- ◯ Always

h. Immigrants and refugees should receive the same care and insurance coverage as US born patients.

- ◯ Never
- ◯ Rarely
- ◯ Sometimes
- ⊗ Usually
- ◯ Always

i. Immigrants and refugees who are undocumented should receive the same care and insurance coverage as US born patients.

- ◯ Never
- ◯ Rarely
- ⊗ Sometimes
- ◯ Usually
- ◯ Always

j. Every physician is professionally obligated to care for immigrants and refugees if they present to your clinic or hospital.

- ◯ Strongly disagree
- ◯ Disagree
- ◯ No opinion
- ⊗ Agree
- ◯ Strongly agree

k. Is healthcare a human right?

⊗ Yes
◯ No

B. If you wish, please tell us about what you enjoy or do not enjoy about immigrant and refugee health care and the greatest challenges you face in caring for this population.

____

SECTION D: DEMOGRAPHIC INFORMATION
Please answer the following questions by checking the box in front of the response choice that best describes you.

a. Your age?

◯ 20 to 24
⊗ 25 to 29
◯ 30 to 34
◯ 35 to 39
◯ 40 or older

b. Your gender?

⊗ Female
◯ Male
◯ Other

c. Are you Hispanic or Latino?

◯ Yes
⊗ No

d. What is your race? (Select one or more responses)

◯ American Indian or Alaska Native
◯ Asian (Please specify):
◯ Black or African American
◯ Native Hawaiian or Other Pacific Islander
⊗ White
◯ Other (Please specify):

e. Were you born in the United States?

⊗ Yes
◯ No

g. Your residency year?

⊗ PGY1
◯ PGY2
◯ PGY3
◯ PGY4
◯ PGY5

h. How would you classify your political ideology?

◯ Conservative
◯ Somewhat conservative
⊗ Moderate
◯ Somewhat liberal
◯ Liberal
◯ Other (Please specify):

i. Estimated level of educational debt?

◯ None
◯ Less than $50,000
◯ $50,000 - $100,000
◯ $100,000 - $200,000
⊗ $200,000 or more

j. Do you plan to subspecialize?

⊗ Yes
◯ No

k. Languages spoken?

- ☒ English
- ☐ Spanish
- ☐ French
- ☐ Hmong
- ☐ Somali
- ☐ Japanese
- ☐ Chinese
- ☐ Russian
- ☐ Ethiopian
- ☐ Other ___________

l. Are you in the Global Health Pathway?

- ◯ Yes
- ⊗ No

m. Did you earn your degree in the US?

- ⊗ Yes
- ◯ No

n. What residency program are you in?

- ⊗ Internal Medicine
- ◯ Med-Peds
- ◯ Pediatrics
- ◯ Family Practice
- ◯ Neurology
- ◯ Psychiatry
- ◯ ObGyn
- ◯ Neurosurgery
- ◯ General Surgery
- ◯ Orthopedic Surgery
- ◯ Urology
- ◯ Surgical sub-specialty (please specify in text box below)
- ◯ Non-clinical specialty (radiology, pathology; please specify in text box below)

Medical Trainees' attitudes, knowledge, and experience with immigrant and refugee health

Response was added on 11/02/2013 1:39pm.

SECTION A: Personal experience with immigrant and refugee health care.

A. Please indicate your level of agreement with the following statements regarding your personal experience with immigrant and refugee health care by checking the box that best represents your experience.

a. During my inpatient rotations, I take care of the following percentage of immigrant and refugee patients:

- ◯ None
- ⊗ 0 -5%
- ◯ 5-10%
- ◯ 10-25%
- ◯ > 25%

b. During my outpatient rotations, I take care of the following percentage of immigrant and refugee patients:

- ◯ None
- ◯ 0-10%
- ⊗ 10 -25%
- ◯ 25-50%
- ◯ 50-75%
- ◯ >75%

c. I would like to take care of more immigrant and refugee patients.

- ◯ Strongly disagree
- ◯ Disagree
- ◯ No opinion
- ⊗ Agree
- ◯ Strongly agree

d. I plan to take care of immigrants and refugees when I finish residency.

- ◯ Strongly disagree
- ◯ Disagree
- ◯ No opinion
- ◯ Agree
- ⊗ Strongly agree

e. I plan to do short term (< 6 months) international work when I finish residency.

- ◯ Strongly disagree
- ◯ Disagree
- ◯ No opinion
- ⊗ Agree
- ◯ Strongly agree

f. I plan to do long term (>6 months) international work when I finish residency.

- ◯ Strongly disagree
- ◯ Disagree
- ◯ No opinion
- ⊗ Agree
- ◯ Strongly agree

g. I plan to work in health disparities in the following way after residency:

Clinical care for the underserved

SECTION B: MEDICAL EDUCATION

A. Please indicate your level of agreement with the following statements regarding your medical education and knowledge about immigrants and refugees by checking the box that best represents your opinion.

a. I have received specialized training in immigrant and refugee health, tropical medicine, or cross-cultural health.

- ◯ Strongly disagree
- ◯ Disagree
- ◯ No opinion
- ⊗ Agree
- ◯ Strongly agree

b. If you have received specialized training in immigrant and refugee health, tropical medicine, or cross-cultural health, please indicate all the contexts in which you received this training:

- ☐ As an undergraduate.
- ☒ As a medical student.
- ☒ As part of my residency.
- ☐ A special program.
- ☐ As part of my fellowship.
- ☐ As part of a degree program (e.g. MPH)
- ☐ Other:

c. I feel comfortable with my fund of knowledge regarding immigrant and refugee health.

- ◯ Strongly disagree
- ⊗ Disagree
- ◯ No opinion
- ◯ Agree
- ◯ Strongly agree

d. I would like to have further training in immigrant and refugee health.

- ◯ Strongly disagree
- ◯ Disagree
- ◯ No opinion
- ⊗ Agree
- ◯ Strongly agree

e. If you agree with the above, please indicate all the contexts in which you would like to receive this training:
□□

- ☒ As part of my residency.
- ☒ A special program.
- ☐ As part of my fellowship.

SECTION C: Attitudes towards immigrant health

A.□Please indicate your level of agreement with the following statements regarding immigrant and refugee health by checking the box that best represents your opinion.

a. I enjoy taking care of immigrants and refugees.

- ◯ Never
- ◯ Rarely
- ◯ Sometimes
- ⊗ Usually
- ◯ Always

b. Please indicate the reasons that you enjoy taking care of immigrants and refugees (may choose more than one).

- ☒ Tropical and other conditions not frequently diagnosed in US-born patients
- ☒ Learning about other cultures
- ☐ They don't complain as much
- ☒ Being able to hear their stories
- ☐ Their care is more complicated
- ☐ Their care is less complicated
- ☒ They are very appreciative of your help.
- ☒ They are extremely vulnerable
- ☐ Other:

c. Taking care of immigrants and refugees is more challenging than taking care of US born patients.

- ◯ Never
- ◯ Rarely
- ◯ Sometimes
- ⊗ Usually
- ◯ Always

d. Please mark all the challenges that you face as a provider when providing care to immigrants and refugees (may choose more than one):

- ☒ Language barriers
- ☒ Insurance barriers
- ☒ Cultural barriers
- ☒ Finding a professional interpreter
- ☐ Knowing how to work with a professional interpreter
- ☒ Time constraints
- ☒ My own knowledge related to tropical and travel medicine
- ☒ Transportation problems for the patient
- ☒ Patients not understanding treatment plan
- ☒ Patients not following treatment plan
- ☒ My lack of knowledge regarding the patient's culture
- ☒ Bias or stereotyping
- ☐ Other:

e. Please mark all of the challenges faced by immigrant and refugee populations when receiving healthcare that you have perceived or witnessed (may choose more than one):

- ☒ Language barriers
- ☒ Insurance barriers
- ☒ Cultural barriers
- ☒ Finding a professional interpreter
- ☒ Knowing how to work with a professional interpreter
- ☒ Time constraints
- ☐ Insufficiently trained health care providers
- ☒ Transportation problems for the patient
- ☒ Food insufficiency
- ☒ Need for child care
- ☒ Patients not understanding treatment plan
- ☒ Patients not following treatment plan
- ☒ My lack of knowledge regarding the patient's culture
- ☒ Bias or stereotyping
- ☒ Trust issues
- ☐ Other...

f. Rank how well immigrants and refugees understand the healthcare that you are trying to provide.

- ⊗ Significantly less than a US born individual
- ○ Less than a US born individual
- ○ Equivalent to a US born individual
- ○ More than a US born patient
- ○ Significantly more than a US born individual

g. Immigrants and refugees adhere to treatment plans and follow my recommendations.

- ○ Never
- ○ Rarely
- ⊗ Sometimes
- ○ Usually
- ○ Always

h. Immigrants and refugees should receive the same care and insurance coverage as US born patients.

- ○ Never
- ○ Rarely
- ○ Sometimes
- ○ Usually
- ⊗ Always

i. Immigrants and refugees who are undocumented should receive the same care and insurance coverage as US born patients.

- ○ Never
- ○ Rarely
- ○ Sometimes
- ○ Usually
- ⊗ Always

j. Every physician is professionally obligated to care for immigrants and refugees if they present to your clinic or hospital.

- ○ Strongly disagree
- ○ Disagree
- ○ No opinion
- ⊗ Agree
- ○ Strongly agree

k. Is healthcare a human right?
- ⊗ Yes
- ◯ No

B. If you wish, please tell us about what you enjoy or do not enjoy about immigrant and refugee health care and the greatest challenges you face in caring for this population.

It is enjoyable to experience other cultures and help the vulnerable. However, system issues make it less enjoyable, such as waiting for interpreters, arranging follow up, and spending enough time to understand barriers to understanding

SECTION D: DEMOGRAPHIC INFORMATION
Please answer the following questions by checking the box in front of the response choice that best describes you.

a. Your age?
- ◯ 20 to 24
- ⊗ 25 to 29
- ◯ 30 to 34
- ◯ 35 to 39
- ◯ 40 or older

b. Your gender?
- ◯ Female
- ⊗ Male
- ◯ Other

c. Are you Hispanic or Latino?
- ◯ Yes
- ⊗ No

d. What is your race? (Select one or more responses)
- ◯ American Indian or Alaska Native
- ◯ Asian (Please specify):
- ◯ Black or African American
- ◯ Native Hawaiian or Other Pacific Islander
- ⊗ White
- ◯ Other (Please specify):

e. Were you born in the United States?
- ⊗ Yes
- ◯ No

g. Your residency year?
- ◯ PGY1
- ◯ PGY2
- ⊗ PGY3
- ◯ PGY4
- ◯ PGY5

h. How would you classify your political ideology?
- ◯ Conservative
- ◯ Somewhat conservative
- ◯ Moderate
- ⊗ Somewhat liberal
- ◯ Liberal
- ◯ Other (Please specify):

i. Estimated level of educational debt?
- ⊗ None
- ◯ Less than $50,000
- ◯ $50,000 - $100,000
- ◯ $100,000 - $200,000
- ◯ $200,000 or more

j. Do you plan to subspecialize?
- ◯ Yes
- ⊗ No

k. Languages spoken?

- ☒ English
- ☒ Spanish
- ☐ French
- ☐ Hmong
- ☐ Somali
- ☐ Japanese
- ☐ Chinese
- ☐ Russian
- ☐ Ethiopian
- ☐ Other ___________

l. Are you in the Global Health Pathway?

- ⊗ Yes
- ◯ No

m. Did you earn your degree in the US?

- ⊗ Yes
- ◯ No

n. What residency program are you in?

- ◯ Internal Medicine
- ⊗ Med-Peds
- ◯ Pediatrics
- ◯ Family Practice
- ◯ Neurology
- ◯ Psychiatry
- ◯ ObGyn
- ◯ Neurosurgery
- ◯ General Surgery
- ◯ Orthopedic Surgery
- ◯ Urology
- ◯ Surgical sub-specialty (please specify in text box below)
- ◯ Non-clinical specialty (radiology, pathology; please specify in text box below)

Medical Trainees' attitudes, knowledge, and experience with immigrant and refugee health

Response was added on 11/02/2013 2:11pm.

SECTION A: Personal experience with immigrant and refugee health care.

A. Please indicate your level of agreement with the following statements regarding your personal experience with immigrant and refugee health care by checking the box that best represents your experience.

a. During my inpatient rotations, I take care of the following percentage of immigrant and refugee patients:

- ◯ None
- ◯ 0 -5%
- ◯ 5-10%
- ◯ 10-25%
- ⊗ > 25%

b. During my outpatient rotations, I take care of the following percentage of immigrant and refugee patients:

- ◯ None
- ◯ 0-10%
- ◯ 10 -25%
- ⊗ 25-50%
- ◯ 50-75%
- ◯ >75%

c. I would like to take care of more immigrant and refugee patients.

- ◯ Strongly disagree
- ◯ Disagree
- ⊗ No opinion
- ◯ Agree
- ◯ Strongly agree

d. I plan to take care of immigrants and refugees when I finish residency.

- ◯ Strongly disagree
- ◯ Disagree
- ◯ No opinion
- ⊗ Agree
- ◯ Strongly agree

e. I plan to do short term (< 6 months) international work when I finish residency.

- ◯ Strongly disagree
- ⊗ Disagree
- ◯ No opinion
- ◯ Agree
- ◯ Strongly agree

f. I plan to do long term (>6 months) international work when I finish residency.

- ◯ Strongly disagree
- ⊗ Disagree
- ◯ No opinion
- ◯ Agree
- ◯ Strongly agree

g. I plan to work in health disparities in the following way after residency:

Would like to practice in a county hospital/other underserved facility

SECTION B: MEDICAL EDUCATION

A. Please indicate your level of agreement with the following statements regarding your medical education and knowledge about immigrants and refugees by checking the box that best represents your opinion.

a. I have received specialized training in immigrant and refugee health, tropical medicine, or cross-cultural health.

- ◯ Strongly disagree
- ◯ Disagree
- ◯ No opinion
- ⊗ Agree
- ◯ Strongly agree

b. If you have received specialized training in immigrant and refugee health, tropical medicine, or cross-cultural health, please indicate all the contexts in which you received this training:

- ☐ As an undergraduate.
- ☒ As a medical student.
- ☐ As part of my residency.
- ☐ A special program.
- ☐ As part of my fellowship.
- ☐ As part of a degree program (e.g. MPH)
- ☐ Other:

c. I feel comfortable with my fund of knowledge regarding immigrant and refugee health.

- ◯ Strongly disagree
- ⊗ Disagree
- ◯ No opinion
- ◯ Agree
- ◯ Strongly agree

d. I would like to have further training in immigrant and refugee health.

- ◯ Strongly disagree
- ◯ Disagree
- ◯ No opinion
- ⊗ Agree
- ◯ Strongly agree

e. If you agree with the above, please indicate all the contexts in which you would like to receive this training:
□□

- ☒ As part of my residency.
- ☐ A special program.
- ☐ As part of my fellowship.

SECTION C: Attitudes towards immigrant health

A.□Please indicate your level of agreement with the following statements regarding immigrant and refugee health by checking the box that best represents your opinion.

a. I enjoy taking care of immigrants and refugees.

- ◯ Never
- ◯ Rarely
- ◯ Sometimes
- ◯ Usually
- ⊗ Always

b. Please indicate the reasons that you enjoy taking care of immigrants and refugees (may choose more than one).

- ☐ Tropical and other conditions not frequently diagnosed in US-born patients
- ☐ Learning about other cultures
- ☐ They don't complain as much
- ☐ Being able to hear their stories
- ☐ Their care is more complicated
- ☐ Their care is less complicated
- ☒ They are very appreciative of your help.
- ☒ They are extremely vulnerable
- ☐ Other:

c. Taking care of immigrants and refugees is more challenging than taking care of US born patients.

- ◯ Never
- ◯ Rarely
- ⊗ Sometimes
- ◯ Usually
- ◯ Always

d. Please mark all the challenges that you face as a provider when providing care to immigrants and refugees (may choose more than one):

- ☒ Language barriers
- ☐ Insurance barriers
- ☒ Cultural barriers
- ☒ Finding a professional interpreter
- ☐ Knowing how to work with a professional interpreter
- ☒ Time constraints
- ☐ My own knowledge related to tropical and travel medicine
- ☐ Transportation problems for the patient
- ☐ Patients not understanding treatment plan
- ☒ Patients not following treatment plan
- ☒ My lack of knowledge regarding the patient's culture
- ☐ Bias or stereotyping
- ☐ Other:

e. Please mark all of the challenges faced by immigrant and refugee populations when receiving healthcare that you have perceived or witnessed (may choose more than one):

- ☒ Language barriers
- ☐ Insurance barriers
- ☒ Cultural barriers
- ☒ Finding a professional interpreter
- ☒ Knowing how to work with a professional interpreter
- ☒ Time constraints
- ☒ Insufficiently trained health care providers
- ☐ Transportation problems for the patient
- ☐ Food insufficiency
- ☐ Need for child care
- ☒ Patients not understanding treatment plan
- ☒ Patients not following treatment plan
- ☐ My lack of knowledge regarding the patient's culture
- ☒ Bias or stereotyping
- ☒ Trust issues
- ☐ Other...

f. Rank how well immigrants and refugees understand the healthcare that you are trying to provide.

- ◯ Significantly less than a US born individual
- ◯ Less than a US born individual
- ⊗ Equivalent to a US born individual
- ◯ More than a US born patient
- ◯ Significantly more than a US born individual

g. Immigrants and refugees adhere to treatment plans and follow my recommendations.

- ◯ Never
- ◯ Rarely
- ◯ Sometimes
- ⊗ Usually
- ◯ Always

h. Immigrants and refugees should receive the same care and insurance coverage as US born patients.

- ◯ Never
- ◯ Rarely
- ◯ Sometimes
- ◯ Usually
- ⊗ Always

i. Immigrants and refugees who are undocumented should receive the same care and insurance coverage as US born patients.

- ◯ Never
- ◯ Rarely
- ◯ Sometimes
- ◯ Usually
- ⊗ Always

j. Every physician is professionally obligated to care for immigrants and refugees if they present to your clinic or hospital.

- ◯ Strongly disagree
- ◯ Disagree
- ◯ No opinion
- ◯ Agree
- ⊗ Strongly agree

k. Is healthcare a human right?

⊗ Yes
◯ No

B. If you wish, please tell us about what you enjoy or do not enjoy about immigrant and refugee health care and the greatest challenges you face in caring for this population.

____

SECTION D: DEMOGRAPHIC INFORMATION
Please answer the following questions by checking the box in front of the response choice that best describes you.

a. Your age?

◯ 20 to 24
⊗ 25 to 29
◯ 30 to 34
◯ 35 to 39
◯ 40 or older

b. Your gender?

⊗ Female
◯ Male
◯ Other

c.Are you Hispanic or Latino?

◯ Yes
⊗ No

d. What is your race? (Select one or more responses)

◯ American Indian or Alaska Native
◯ Asian (Please specify):
◯ Black or African American
◯ Native Hawaiian or Other Pacific Islander
⊗ White
◯ Other (Please specify):

e.Were you born in the United States?

⊗ Yes
◯ No

g. Your residency year?

⊗ PGY1
◯ PGY2
◯ PGY3
◯ PGY4
◯ PGY5

h. How would you classify your political ideology?

◯ Conservative
◯ Somewhat conservative
◯ Moderate
◯ Somewhat liberal
⊗ Liberal
◯ Other (Please specify):

i. Estimated level of educational debt?

◯ None
◯ Less than $50,000
◯ $50,000 - $100,000
◯ $100,000 - $200,000
⊗ $200,000 or more

j.Do you plan to subspecialize?

⊗ Yes
◯ No

k. Languages spoken?

- ☒ English
- ☒ Spanish
- ☐ French
- ☐ Hmong
- ☐ Somali
- ☐ Japanese
- ☐ Chinese
- ☐ Russian
- ☐ Ethiopian
- ☐ Other ___________

l. Are you in the Global Health Pathway?

- ◯ Yes
- ⊗ No

m. Did you earn your degree in the US?

- ⊗ Yes
- ◯ No

n. What residency program are you in?

- ◯ Internal Medicine
- ◯ Med-Peds
- ◯ Pediatrics
- ◯ Family Practice
- ◯ Neurology
- ◯ Psychiatry
- ⊗ ObGyn
- ◯ Neurosurgery
- ◯ General Surgery
- ◯ Orthopedic Surgery
- ◯ Urology
- ◯ Surgical sub-specialty (please specify in text box below)
- ◯ Non-clinical specialty (radiology, pathology; please specify in text box below)

Medical Trainees' attitudes, knowledge, and experience with immigrant and refugee health

Response was added on 11/02/2013 2:15pm.

SECTION A: Personal experience with immigrant and refugee health care.

A. Please indicate your level of agreement with the following statements regarding your personal experience with immigrant and refugee health care by checking the box that best represents your experience.

a. During my inpatient rotations, I take care of the following percentage of immigrant and refugee patients:

- ◯ None
- ◯ 0 -5%
- ⊗ 5-10%
- ◯ 10-25%
- ◯ > 25%

b. During my outpatient rotations, I take care of the following percentage of immigrant and refugee patients:

- ◯ None
- ◯ 0-10%
- ◯ 10 -25%
- ⊗ 25-50%
- ◯ 50-75%
- ◯ >75%

c. I would like to take care of more immigrant and refugee patients.

- ◯ Strongly disagree
- ◯ Disagree
- ⊗ No opinion
- ◯ Agree
- ◯ Strongly agree

d. I plan to take care of immigrants and refugees when I finish residency.

- ◯ Strongly disagree
- ◯ Disagree
- ◯ No opinion
- ⊗ Agree
- ◯ Strongly agree

e. I plan to do short term (< 6 months) international work when I finish residency.

- ◯ Strongly disagree
- ◯ Disagree
- ◯ No opinion
- ⊗ Agree
- ◯ Strongly agree

f. I plan to do long term (>6 months) international work when I finish residency.

- ◯ Strongly disagree
- ⊗ Disagree
- ◯ No opinion
- ◯ Agree
- ◯ Strongly agree

g. I plan to work in health disparities in the following way after residency:

Working with African American populations American Indian and pursuing degree in public health

SECTION B: MEDICAL EDUCATION

A. Please indicate your level of agreement with the following statements regarding your medical education and knowledge about immigrants and refugees by checking the box that best represents your opinion.

a. I have received specialized training in immigrant and refugee health, tropical medicine, or cross-cultural health.

- ◯ Strongly disagree
- ◯ Disagree
- ◯ No opinion
- ⊗ Agree
- ◯ Strongly agree

b. If you have received specialized training in immigrant and refugee health, tropical medicine, or cross-cultural health, please indicate all the contexts in which you received this training:

- ☐ As an undergraduate.
- ☒ As a medical student.
- ☒ As part of my residency.
- ☐ A special program.
- ☐ As part of my fellowship.
- ☐ As part of a degree program (e.g. MPH)
- ☐ Other:

c. I feel comfortable with my fund of knowledge regarding immigrant and refugee health.

- ◯ Strongly disagree
- ◯ Disagree
- ◯ No opinion
- ⊗ Agree
- ◯ Strongly agree

d. I would like to have further training in immigrant and refugee health.

- ◯ Strongly disagree
- ◯ Disagree
- ◯ No opinion
- ◯ Agree
- ⊗ Strongly agree

e. If you agree with the above, please indicate all the contexts in which you would like to receive this training:
□□

- ☒ As part of my residency.
- ☐ A special program.
- ☒ As part of my fellowship.

SECTION C: Attitudes towards immigrant health

A.□Please indicate your level of agreement with the following statements regarding immigrant and refugee health by checking the box that best represents your opinion.

a. I enjoy taking care of immigrants and refugees.

- ◯ Never
- ◯ Rarely
- ◯ Sometimes
- ⊗ Usually
- ◯ Always

b. Please indicate the reasons that you enjoy taking care of immigrants and refugees (may choose more than one).

- ☒ Tropical and other conditions not frequently diagnosed in US-born patients
- ☒ Learning about other cultures
- ☒ They don't complain as much
- ☒ Being able to hear their stories
- ☐ Their care is more complicated
- ☐ Their care is less complicated
- ☒ They are very appreciative of your help.
- ☒ They are extremely vulnerable
- ☐ Other:

c. Taking care of immigrants and refugees is more challenging than taking care of US born patients.

- ◯ Never
- ◯ Rarely
- ◯ Sometimes
- ⊗ Usually
- ◯ Always

d. Please mark all the challenges that you face as a provider when providing care to immigrants and refugees (may choose more than one):

- ☒ Language barriers
- ☐ Insurance barriers
- ☒ Cultural barriers
- ☒ Finding a professional interpreter
- ☐ Knowing how to work with a professional interpreter
- ☒ Time constraints
- ☒ My own knowledge related to tropical and travel medicine
- ☒ Transportation problems for the patient
- ☒ Patients not understanding treatment plan
- ☒ Patients not following treatment plan
- ☐ My lack of knowledge regarding the patient's culture
- ☐ Bias or stereotyping
- ☐ Other:

e. Please mark all of the challenges faced by immigrant and refugee populations when receiving healthcare that you have perceived or witnessed (may choose more than one):

- ☒ Language barriers
- ☒ Insurance barriers
- ☒ Cultural barriers
- ☒ Finding a professional interpreter
- ☒ Knowing how to work with a professional interpreter
- ☒ Time constraints
- ☒ Insufficiently trained health care providers
- ☒ Transportation problems for the patient
- ☒ Food insufficiency
- ☒ Need for child care
- ☒ Patients not understanding treatment plan
- ☒ Patients not following treatment plan
- ☒ My lack of knowledge regarding the patient's culture
- ☒ Bias or stereotyping
- ☒ Trust issues
- ☐ Other...

f. Rank how well immigrants and refugees understand the healthcare that you are trying to provide.

- ◯ Significantly less than a US born individual
- ⊗ Less than a US born individual
- ◯ Equivalent to a US born individual
- ◯ More than a US born patient
- ◯ Significantly more than a US born individual

g. Immigrants and refugees adhere to treatment plans and follow my recommendations.

- ◯ Never
- ◯ Rarely
- ⊗ Sometimes
- ◯ Usually
- ◯ Always

h. Immigrants and refugees should receive the same care and insurance coverage as US born patients.

- ◯ Never
- ◯ Rarely
- ◯ Sometimes
- ◯ Usually
- ⊗ Always

i. Immigrants and refugees who are undocumented should receive the same care and insurance coverage as US born patients.

- ◯ Never
- ◯ Rarely
- ⊗ Sometimes
- ◯ Usually
- ◯ Always

j. Every physician is professionally obligated to care for immigrants and refugees if they present to your clinic or hospital.

- ◯ Strongly disagree
- ◯ Disagree
- ◯ No opinion
- ◯ Agree
- ⊗ Strongly agree

k. Is healthcare a human right?
- ⊗ Yes
- ◯ No

B. If you wish, please tell us about what you enjoy or do not enjoy about immigrant and refugee health care and the greatest challenges you face in caring for this population. ____

SECTION D: DEMOGRAPHIC INFORMATION
Please answer the following questions by checking the box in front of the response choice that best describes you.

a. Your age?
- ◯ 20 to 24
- ⊗ 25 to 29
- ◯ 30 to 34
- ◯ 35 to 39
- ◯ 40 or older

b. Your gender?
- ◯ Female
- ⊗ Male
- ◯ Other

c. Are you Hispanic or Latino?
- ◯ Yes
- ⊗ No

d. What is your race? (Select one or more responses)
- ◯ American Indian or Alaska Native
- ◯ Asian (Please specify):
- ◯ Black or African American
- ◯ Native Hawaiian or Other Pacific Islander
- ⊗ White
- ◯ Other (Please specify):

e. Were you born in the United States?
- ⊗ Yes
- ◯ No

g. Your residency year?
- ◯ PGY1
- ◯ PGY2
- ⊗ PGY3
- ◯ PGY4
- ◯ PGY5

h. How would you classify your political ideology?
- ◯ Conservative
- ◯ Somewhat conservative
- ◯ Moderate
- ◯ Somewhat liberal
- ⊗ Liberal
- ◯ Other (Please specify):

i. Estimated level of educational debt?
- ◯ None
- ◯ Less than $50,000
- ◯ $50,000 - $100,000
- ◯ $100,000 - $200,000
- ⊗ $200,000 or more

j. Do you plan to subspecialize?
- ◯ Yes
- ⊗ No

k. Languages spoken?

☒ English
☒ Spanish
☐ French
☐ Hmong
☐ Somali
☐ Japanese
☐ Chinese
☐ Russian
☐ Ethiopian
☐ Other ___________

l. Are you in the Global Health Pathway?

○ Yes
⊗ No

m. Did you earn your degree in the US?

⊗ Yes
○ No

n. What residency program are you in?

○ Internal Medicine
⊗ Med-Peds
○ Pediatrics
○ Family Practice
○ Neurology
○ Psychiatry
○ ObGyn
○ Neurosurgery
○ General Surgery
○ Orthopedic Surgery
○ Urology
○ Surgical sub-specialty (please specify in text box below)
○ Non-clinical specialty (radiology, pathology; please specify in text box below)

Medical Trainees' attitudes, knowledge, and experience with immigrant and refugee health

Response was added on 11/02/2013 3:41pm.

SECTION A: Personal experience with immigrant and refugee health care.

A. Please indicate your level of agreement with the following statements regarding your personal experience with immigrant and refugee health care by checking the box that best represents your experience.

a. During my inpatient rotations, I take care of the following percentage of immigrant and refugee patients:
- ◯ None
- ◯ 0 -5%
- ◯ 5-10%
- ⊗ 10-25%
- ◯ > 25%

b. During my outpatient rotations, I take care of the following percentage of immigrant and refugee patients:
- ⊗ None
- ◯ 0-10%
- ◯ 10 -25%
- ◯ 25-50%
- ◯ 50-75%
- ◯ >75%

c. I would like to take care of more immigrant and refugee patients.
- ◯ Strongly disagree
- ◯ Disagree
- ⊗ No opinion
- ◯ Agree
- ◯ Strongly agree

d. I plan to take care of immigrants and refugees when I finish residency.
- ◯ Strongly disagree
- ◯ Disagree
- ⊗ No opinion
- ◯ Agree
- ◯ Strongly agree

e. I plan to do short term (< 6 months) international work when I finish residency.
- ◯ Strongly disagree
- ◯ Disagree
- ◯ No opinion
- ⊗ Agree
- ◯ Strongly agree

f. I plan to do long term (>6 months) international work when I finish residency.
- ◯ Strongly disagree
- ◯ Disagree
- ◯ No opinion
- ⊗ Agree
- ◯ Strongly agree

g. I plan to work in health disparities in the following way after residency:

I dont

SECTION B: MEDICAL EDUCATION

A. Please indicate your level of agreement with the following statements regarding your medical education and knowledge about immigrants and refugees by checking the box that best represents your opinion.

a. I have received specialized training in immigrant and refugee health, tropical medicine, or cross-cultural health.

- ◯ Strongly disagree
- ⊗ Disagree
- ◯ No opinion
- ◯ Agree
- ◯ Strongly agree

c. I feel comfortable with my fund of knowledge regarding immigrant and refugee health.

- ◯ Strongly disagree
- ◯ Disagree
- ◯ No opinion
- ⊗ Agree
- ◯ Strongly agree

d. I would like to have further training in immigrant and refugee health.

- ◯ Strongly disagree
- ⊗ Disagree
- ◯ No opinion
- ◯ Agree
- ◯ Strongly agree

SECTION C: Attitudes towards immigrant health

A.□Please indicate your level of agreement with the following statements regarding immigrant and refugee health by checking the box that best represents your opinion.

a. I enjoy taking care of immigrants and refugees.

- ◯ Never
- ◯ Rarely
- ◯ Sometimes
- ⊗ Usually
- ◯ Always

b. Please indicate the reasons that you enjoy taking care of immigrants and refugees (may choose more than one).

- ☐ Tropical and other conditions not frequently diagnosed in US-born patients
- ☐ Learning about other cultures
- ☐ They don't complain as much
- ☐ Being able to hear their stories
- ☐ Their care is more complicated
- ☐ Their care is less complicated
- ☐ They are very appreciative of your help.
- ☐ They are extremely vulnerable
- ☒ Other:

I don't enjoy it any more than any other patient

c. Taking care of immigrants and refugees is more challenging than taking care of US born patients.

- ◯ Never
- ◯ Rarely
- ⊗ Sometimes
- ◯ Usually
- ◯ Always

d. Please mark all the challenges that you face as a provider when providing care to immigrants and refugees (may choose more than one):

- ☒ Language barriers
- ☐ Insurance barriers
- ☒ Cultural barriers
- ☐ Finding a professional interpreter
- ☐ Knowing how to work with a professional interpreter
- ☐ Time constraints
- ☐ My own knowledge related to tropical and travel medicine
- ☐ Transportation problems for the patient
- ☐ Patients not understanding treatment plan
- ☐ Patients not following treatment plan
- ☐ My lack of knowledge regarding the patient's culture
- ☐ Bias or stereotyping
- ☐ Other:

e. Please mark all of the challenges faced by immigrant and refugee populations when receiving healthcare that you have perceived or witnessed (may choose more than one):

- ☒ Language barriers
- ☐ Insurance barriers
- ☒ Cultural barriers
- ☒ Finding a professional interpreter
- ☐ Knowing how to work with a professional interpreter
- ☒ Time constraints
- ☐ Insufficiently trained health care providers
- ☐ Transportation problems for the patient
- ☐ Food insufficiency
- ☐ Need for child care
- ☐ Patients not understanding treatment plan
- ☐ Patients not following treatment plan
- ☐ My lack of knowledge regarding the patient's culture
- ☐ Bias or stereotyping
- ☐ Trust issues
- ☐ Other...

f. Rank how well immigrants and refugees understand the healthcare that you are trying to provide.

- ○ Significantly less than a US born individual
- ⊗ Less than a US born individual
- ○ Equivalent to a US born individual
- ○ More than a US born patient
- ○ Significantly more than a US born individual

g. Immigrants and refugees adhere to treatment plans and follow my recommendations.

- ○ Never
- ○ Rarely
- ○ Sometimes
- ⊗ Usually
- ○ Always

h. Immigrants and refugees should receive the same care and insurance coverage as US born patients.

- ○ Never
- ○ Rarely
- ○ Sometimes
- ○ Usually
- ⊗ Always

i. Immigrants and refugees who are undocumented should receive the same care and insurance coverage as US born patients.

- ○ Never
- ○ Rarely
- ○ Sometimes
- ○ Usually
- ⊗ Always

j. Every physician is professionally obligated to care for immigrants and refugees if they present to your clinic or hospital.

- ○ Strongly disagree
- ○ Disagree
- ○ No opinion
- ○ Agree
- ⊗ Strongly agree

k. Is healthcare a human right?
- ⊗ Yes
- ◯ No

B. If you wish, please tell us about what you enjoy or do not enjoy about immigrant and refugee health care and the greatest challenges you face in caring for this population.

____

SECTION D: DEMOGRAPHIC INFORMATION
Please answer the following questions by checking the box in front of the response choice that best describes you.

a. Your age?
- ◯ 20 to 24
- ⊗ 25 to 29
- ◯ 30 to 34
- ◯ 35 to 39
- ◯ 40 or older

b. Your gender?
- ◯ Female
- ⊗ Male
- ◯ Other

c.Are you Hispanic or Latino?
- ◯ Yes
- ⊗ No

d. What is your race? (Select one or more responses)
- ◯ American Indian or Alaska Native
- ◯ Asian (Please specify):
- ◯ Black or African American
- ◯ Native Hawaiian or Other Pacific Islander
- ⊗ White
- ◯ Other (Please specify):

e.Were you born in the United States?
- ⊗ Yes
- ◯ No

g. Your residency year?
- ◯ PGY1
- ◯ PGY2
- ⊗ PGY3
- ◯ PGY4
- ◯ PGY5

h. How would you classify your political ideology?
- ◯ Conservative
- ◯ Somewhat conservative
- ◯ Moderate
- ◯ Somewhat liberal
- ◯ Liberal
- ◯ Other (Please specify):

i. Estimated level of educational debt?
- ◯ None
- ◯ Less than $50,000
- ◯ $50,000 - $100,000
- ⊗ $100,000 - $200,000
- ◯ $200,000 or more

j.Do you plan to subspecialize?
- ⊗ Yes
- ◯ No

k. Languages spoken?

- ☐ English
- ☐ Spanish
- ☐ French
- ☐ Hmong
- ☐ Somali
- ☐ Japanese
- ☐ Chinese
- ☐ Russian
- ☐ Ethiopian
- ☐ Other ___________

l. Are you in the Global Health Pathway?

- ◯ Yes
- ⊗ No

m. Did you earn your degree in the US?

- ⊗ Yes
- ◯ No

n. What residency program are you in?

- ◯ Internal Medicine
- ◯ Med-Peds
- ◯ Pediatrics
- ◯ Family Practice
- ◯ Neurology
- ◯ Psychiatry
- ◯ ObGyn
- ◯ Neurosurgery
- ◯ General Surgery
- ◯ Orthopedic Surgery
- ⊗ Urology
- ◯ Surgical sub-specialty (please specify in text box below)
- ◯ Non-clinical specialty (radiology, pathology; please specify in text box below)

Medical Trainees' attitudes, knowledge, and experience with immigrant and refugee health

Response was added on 11/02/2013 6:01pm.

SECTION A: Personal experience with immigrant and refugee health care.

A. Please indicate your level of agreement with the following statements regarding your personal experience with immigrant and refugee health care by checking the box that best represents your experience.

a. During my inpatient rotations, I take care of the following percentage of immigrant and refugee patients:

- ◯ None
- ◯ 0 -5%
- ⊗ 5-10%
- ◯ 10-25%
- ◯ > 25%

b. During my outpatient rotations, I take care of the following percentage of immigrant and refugee patients:

- ◯ None
- ◯ 0-10%
- ⊗ 10 -25%
- ◯ 25-50%
- ◯ 50-75%
- ◯ >75%

c. I would like to take care of more immigrant and refugee patients.

- ◯ Strongly disagree
- ◯ Disagree
- ⊗ No opinion
- ◯ Agree
- ◯ Strongly agree

d. I plan to take care of immigrants and refugees when I finish residency.

- ◯ Strongly disagree
- ◯ Disagree
- ◯ No opinion
- ⊗ Agree
- ◯ Strongly agree

e. I plan to do short term (< 6 months) international work when I finish residency.

- ⊗ Strongly disagree
- ◯ Disagree
- ◯ No opinion
- ◯ Agree
- ◯ Strongly agree

f. I plan to do long term (>6 months) international work when I finish residency.

- ⊗ Strongly disagree
- ◯ Disagree
- ◯ No opinion
- ◯ Agree
- ◯ Strongly agree

g. I plan to work in health disparities in the following way after residency:

If they come into my clinic

SECTION B: MEDICAL EDUCATION

A. Please indicate your level of agreement with the following statements regarding your medical education and knowledge about immigrants and refugees by checking the box that best represents your opinion.

a. I have received specialized training in immigrant and refugee health, tropical medicine, or cross-cultural health.

- ◯ Strongly disagree
- ◯ Disagree
- ◯ No opinion
- ⊗ Agree
- ◯ Strongly agree

b. If you have received specialized training in immigrant and refugee health, tropical medicine, or cross-cultural health, please indicate all the contexts in which you received this training:

- ☐ As an undergraduate.
- ☒ As a medical student.
- ☒ As part of my residency.
- ☐ A special program.
- ☐ As part of my fellowship.
- ☐ As part of a degree program (e.g. MPH)
- ☐ Other:

c. I feel comfortable with my fund of knowledge regarding immigrant and refugee health.

- ◯ Strongly disagree
- ◯ Disagree
- ◯ No opinion
- ⊗ Agree
- ◯ Strongly agree

d. I would like to have further training in immigrant and refugee health.

- ◯ Strongly disagree
- ◯ Disagree
- ⊗ No opinion
- ◯ Agree
- ◯ Strongly agree

SECTION C: Attitudes towards immigrant health

A.Please indicate your level of agreement with the following statements regarding immigrant and refugee health by checking the box that best represents your opinion.

a. I enjoy taking care of immigrants and refugees.

- ◯ Never
- ◯ Rarely
- ◯ Sometimes
- ⊗ Usually
- ◯ Always

b. Please indicate the reasons that you enjoy taking care of immigrants and refugees (may choose more than one).

- ☐ Tropical and other conditions not frequently diagnosed in US-born patients
- ☒ Learning about other cultures
- ☐ They don't complain as much
- ☐ Being able to hear their stories
- ☐ Their care is more complicated
- ☐ Their care is less complicated
- ☐ They are very appreciative of your help.
- ☐ They are extremely vulnerable
- ☐ Other:

c. Taking care of immigrants and refugees is more challenging than taking care of US born patients.

- ◯ Never
- ◯ Rarely
- ⊗ Sometimes
- ◯ Usually
- ◯ Always

d. Please mark all the challenges that you face as a provider when providing care to immigrants and refugees (may choose more than one):

- ☒ Language barriers
- ☒ Insurance barriers
- ☒ Cultural barriers
- ☐ Finding a professional interpreter
- ☐ Knowing how to work with a professional interpreter
- ☒ Time constraints
- ☒ My own knowledge related to tropical and travel medicine
- ☒ Transportation problems for the patient
- ☐ Patients not understanding treatment plan
- ☐ Patients not following treatment plan
- ☐ My lack of knowledge regarding the patient's culture
- ☐ Bias or stereotyping
- ☐ Other:

e. Please mark all of the challenges faced by immigrant and refugee populations when receiving healthcare that you have perceived or witnessed (may choose more than one):

- ☒ Language barriers
- ☒ Insurance barriers
- ☒ Cultural barriers
- ☐ Finding a professional interpreter
- ☐ Knowing how to work with a professional interpreter
- ☒ Time constraints
- ☒ Insufficiently trained health care providers
- ☒ Transportation problems for the patient
- ☐ Food insufficiency
- ☐ Need for child care
- ☐ Patients not understanding treatment plan
- ☐ Patients not following treatment plan
- ☐ My lack of knowledge regarding the patient's culture
- ☐ Bias or stereotyping
- ☐ Trust issues
- ☐ Other...

f. Rank how well immigrants and refugees understand the healthcare that you are trying to provide.

- ○ Significantly less than a US born individual
- ○ Less than a US born individual
- ⊗ Equivalent to a US born individual
- ○ More than a US born patient
- ○ Significantly more than a US born individual

g. Immigrants and refugees adhere to treatment plans and follow my recommendations.

- ○ Never
- ○ Rarely
- ○ Sometimes
- ⊗ Usually
- ○ Always

h. Immigrants and refugees should receive the same care and insurance coverage as US born patients.

- ○ Never
- ○ Rarely
- ○ Sometimes
- ○ Usually
- ⊗ Always

i. Immigrants and refugees who are undocumented should receive the same care and insurance coverage as US born patients.

- ○ Never
- ○ Rarely
- ○ Sometimes
- ○ Usually
- ⊗ Always

j. Every physician is professionally obligated to care for immigrants and refugees if they present to your clinic or hospital.

- ○ Strongly disagree
- ○ Disagree
- ○ No opinion
- ○ Agree
- ⊗ Strongly agree

k. Is healthcare a human right?
- ⊗ Yes
- ◯ No

B. If you wish, please tell us about what you enjoy or do not enjoy about immigrant and refugee health care and the greatest challenges you face in caring for this population. __________________________________

SECTION D: DEMOGRAPHIC INFORMATION
Please answer the following questions by checking the box in front of the response choice that best describes you.

a. Your age?
- ◯ 20 to 24
- ⊗ 25 to 29
- ◯ 30 to 34
- ◯ 35 to 39
- ◯ 40 or older

b. Your gender?
- ◯ Female
- ⊗ Male
- ◯ Other

c.Are you Hispanic or Latino?
- ◯ Yes
- ⊗ No

d. What is your race? (Select one or more responses)
- ◯ American Indian or Alaska Native
- ◯ Asian (Please specify):
- ◯ Black or African American
- ◯ Native Hawaiian or Other Pacific Islander
- ⊗ White
- ◯ Other (Please specify):

e.Were you born in the United States?
- ⊗ Yes
- ◯ No

g. Your residency year?
- ◯ PGY1
- ⊗ PGY2
- ◯ PGY3
- ◯ PGY4
- ◯ PGY5

h. How would you classify your political ideology?
- ◯ Conservative
- ◯ Somewhat conservative
- ◯ Moderate
- ⊗ Somewhat liberal
- ◯ Liberal
- ◯ Other (Please specify):

i. Estimated level of educational debt?
- ◯ None
- ◯ Less than $50,000
- ◯ $50,000 - $100,000
- ◯ $100,000 - $200,000
- ⊗ $200,000 or more

j.Do you plan to subspecialize?
- ◯ Yes
- ⊗ No

k. Languages spoken?

- ☒ English
- ☐ Spanish
- ☐ French
- ☐ Hmong
- ☐ Somali
- ☐ Japanese
- ☐ Chinese
- ☐ Russian
- ☐ Ethiopian
- ☐ Other ___________

l. Are you in the Global Health Pathway?

- ◯ Yes
- ⊗ No

m.□ Did you earn your degree in the US?

- ⊗ Yes
- ◯ No

n. What residency program are you in?

- ◯ Internal Medicine
- ◯ Med-Peds
- ◯ Pediatrics
- ⊗ Family Practice
- ◯ Neurology
- ◯ Psychiatry
- ◯ ObGyn
- ◯ Neurosurgery
- ◯ General Surgery
- ◯ Orthopedic Surgery
- ◯ Urology
- ◯ Surgical sub-specialty (please specify in text box below)
- ◯ Non-clinical specialty (radiology, pathology; please specify in text box below)

Medical Trainees' attitudes, knowledge, and experience with immigrant and refugee health

Response was added on 11/02/2013 6:16pm.

SECTION A: Personal experience with immigrant and refugee health care.

A. Please indicate your level of agreement with the following statements regarding your personal experience with immigrant and refugee health care by checking the box that best represents your experience.

a. During my inpatient rotations, I take care of the following percentage of immigrant and refugee patients:

- ◯ None
- ⊗ 0 -5%
- ◯ 5-10%
- ◯ 10-25%
- ◯ > 25%

b. During my outpatient rotations, I take care of the following percentage of immigrant and refugee patients:

- ◯ None
- ◯ 0-10%
- ◯ 10 -25%
- ⊗ 25-50%
- ◯ 50-75%
- ◯ >75%

c. I would like to take care of more immigrant and refugee patients.

- ◯ Strongly disagree
- ◯ Disagree
- ⊗ No opinion
- ◯ Agree
- ◯ Strongly agree

d. I plan to take care of immigrants and refugees when I finish residency.

- ◯ Strongly disagree
- ◯ Disagree
- ◯ No opinion
- ◯ Agree
- ⊗ Strongly agree

e. I plan to do short term (< 6 months) international work when I finish residency.

- ◯ Strongly disagree
- ◯ Disagree
- ◯ No opinion
- ◯ Agree
- ⊗ Strongly agree

f. I plan to do long term (>6 months) international work when I finish residency.

- ◯ Strongly disagree
- ◯ Disagree
- ⊗ No opinion
- ◯ Agree
- ◯ Strongly agree

g. I plan to work in health disparities in the following way after residency:

Research on diseases relevant to under-served populations, advocacy, direct patient care of under-served populations

SECTION B: MEDICAL EDUCATION

A. Please indicate your level of agreement with the following statements regarding your medical education and knowledge about immigrants and refugees by checking the box that best represents your opinion.

a. I have received specialized training in immigrant and refugee health, tropical medicine, or cross-cultural health.

- ◯ Strongly disagree
- ◯ Disagree
- ◯ No opinion
- ◯ Agree
- ⊗ Strongly agree

b. If you have received specialized training in immigrant and refugee health, tropical medicine, or cross-cultural health, please indicate all the contexts in which you received this training:

- ☐ As an undergraduate.
- ☐ As a medical student.
- ☒ As part of my residency.
- ☒ A special program.
- ☐ As part of my fellowship.
- ☒ As part of a degree program (e.g. MPH)
- ☐ Other:

c. I feel comfortable with my fund of knowledge regarding immigrant and refugee health.

- ◯ Strongly disagree
- ◯ Disagree
- ◯ No opinion
- ⊗ Agree
- ◯ Strongly agree

d. I would like to have further training in immigrant and refugee health.

- ◯ Strongly disagree
- ◯ Disagree
- ◯ No opinion
- ◯ Agree
- ⊗ Strongly agree

e. If you agree with the above, please indicate all the contexts in which you would like to receive this training:

- ☒ As part of my residency.
- ☒ A special program.
- ☒ As part of my fellowship.

SECTION C: Attitudes towards immigrant health

A. Please indicate your level of agreement with the following statements regarding immigrant and refugee health by checking the box that best represents your opinion.

a. I enjoy taking care of immigrants and refugees.

- ◯ Never
- ◯ Rarely
- ◯ Sometimes
- ⊗ Usually
- ◯ Always

b. Please indicate the reasons that you enjoy taking care of immigrants and refugees (may choose more than one).

- ☒ Tropical and other conditions not frequently diagnosed in US-born patients
- ☒ Learning about other cultures
- ☐ They don't complain as much
- ☒ Being able to hear their stories
- ☐ Their care is more complicated
- ☐ Their care is less complicated
- ☒ They are very appreciative of your help.
- ☒ They are extremely vulnerable
- ☐ Other:

c. Taking care of immigrants and refugees is more challenging than taking care of US born patients.

- ◯ Never
- ◯ Rarely
- ⊗ Sometimes
- ◯ Usually
- ◯ Always

d. Please mark all the challenges that you face as a provider when providing care to immigrants and refugees (may choose more than one):

- ☒ Language barriers
- ☐ Insurance barriers
- ☒ Cultural barriers
- ☐ Finding a professional interpreter
- ☐ Knowing how to work with a professional interpreter
- ☒ Time constraints
- ☐ My own knowledge related to tropical and travel medicine
- ☒ Transportation problems for the patient
- ☒ Patients not understanding treatment plan
- ☒ Patients not following treatment plan
- ☒ My lack of knowledge regarding the patient's culture
- ☐ Bias or stereotyping
- ☐ Other:

e. Please mark all of the challenges faced by immigrant and refugee populations when receiving healthcare that you have perceived or witnessed (may choose more than one):

- ☒ Language barriers
- ☒ Insurance barriers
- ☒ Cultural barriers
- ☒ Finding a professional interpreter
- ☐ Knowing how to work with a professional interpreter
- ☒ Time constraints
- ☒ Insufficiently trained health care providers
- ☒ Transportation problems for the patient
- ☒ Food insufficiency
- ☒ Need for child care
- ☒ Patients not understanding treatment plan
- ☒ Patients not following treatment plan
- ☒ My lack of knowledge regarding the patient's culture
- ☒ Bias or stereotyping
- ☒ Trust issues
- ☐ Other...

f. Rank how well immigrants and refugees understand the healthcare that you are trying to provide.

- ◯ Significantly less than a US born individual
- ⊗ Less than a US born individual
- ◯ Equivalent to a US born individual
- ◯ More than a US born patient
- ◯ Significantly more than a US born individual

g. Immigrants and refugees adhere to treatment plans and follow my recommendations.

- ◯ Never
- ◯ Rarely
- ⊗ Sometimes
- ◯ Usually
- ◯ Always

h. Immigrants and refugees should receive the same care and insurance coverage as US born patients.

- ◯ Never
- ◯ Rarely
- ◯ Sometimes
- ◯ Usually
- ⊗ Always

i. Immigrants and refugees who are undocumented should receive the same care and insurance coverage as US born patients.

- ◯ Never
- ◯ Rarely
- ◯ Sometimes
- ◯ Usually
- ⊗ Always

j. Every physician is professionally obligated to care for immigrants and refugees if they present to your clinic or hospital.

- ◯ Strongly disagree
- ◯ Disagree
- ◯ No opinion
- ◯ Agree
- ⊗ Strongly agree

k. Is healthcare a human right?
⊗ Yes
○ No

B. If you wish, please tell us about what you enjoy or do not enjoy about immigrant and refugee health care and the greatest challenges you face in caring for this population.

I enjoy the variety of problems that they bring to clinic. I enjoy the aspect of creative problem solving to come up with solutions they can use. I find it challenging because that takes time and time is usually lacking in short clinic appointments and on busy inpatient services.

SECTION D: DEMOGRAPHIC INFORMATION
Please answer the following questions by checking the box in front of the response choice that best describes you.

a. Your age?
○ 20 to 24
○ 25 to 29
⊗ 30 to 34
○ 35 to 39
○ 40 or older

b. Your gender?
⊗ Female
○ Male
○ Other

c. Are you Hispanic or Latino?
○ Yes
⊗ No

d. What is your race? (Select one or more responses)
○ American Indian or Alaska Native
○ Asian (Please specify):
○ Black or African American
○ Native Hawaiian or Other Pacific Islander
⊗ White
○ Other (Please specify):

e. Were you born in the United States?
⊗ Yes
○ No

g. Your residency year?
○ PGY1
⊗ PGY2
○ PGY3
○ PGY4
○ PGY5

h. How would you classify your political ideology?
○ Conservative
○ Somewhat conservative
○ Moderate
○ Somewhat liberal
⊗ Liberal
○ Other (Please specify):

i. Estimated level of educational debt?
⊗ None
○ Less than $50,000
○ $50,000 - $100,000
○ $100,000 - $200,000
○ $200,000 or more

j. Do you plan to subspecialize?
⊗ Yes
○ No

k. Languages spoken?

- ☒ English
- ☐ Spanish
- ☐ French
- ☐ Hmong
- ☐ Somali
- ☐ Japanese
- ☐ Chinese
- ☒ Russian
- ☐ Ethiopian
- ☐ Other ___________

l. Are you in the Global Health Pathway?

- ⊗ Yes
- ◯ No

m. Did you earn your degree in the US?

- ⊗ Yes
- ◯ No

n. What residency program are you in?

- ◯ Internal Medicine
- ⊗ Med-Peds
- ◯ Pediatrics
- ◯ Family Practice
- ◯ Neurology
- ◯ Psychiatry
- ◯ ObGyn
- ◯ Neurosurgery
- ◯ General Surgery
- ◯ Orthopedic Surgery
- ◯ Urology
- ◯ Surgical sub-specialty (please specify in text box below)
- ◯ Non-clinical specialty (radiology, pathology; please specify in text box below)

Medical Trainees' attitudes, knowledge, and experience with immigrant and refugee health

Response was added on 11/02/2013 7:32pm.

SECTION A: Personal experience with immigrant and refugee health care.

A. Please indicate your level of agreement with the following statements regarding your personal experience with immigrant and refugee health care by checking the box that best represents your experience.

a. During my inpatient rotations, I take care of the following percentage of immigrant and refugee patients:	◯ None<br>◯ 0 -5%<br>⊗ 5-10%<br>◯ 10-25%<br>◯ > 25%
b. During my outpatient rotations, I take care of the following percentage of immigrant and refugee patients:	◯ None<br>◯ 0-10%<br>◯ 10 -25%<br>◯ 25-50%<br>⊗ 50-75%<br>◯ >75%
c. I would like to take care of more immigrant and refugee patients.	◯ Strongly disagree<br>◯ Disagree<br>◯ No opinion<br>⊗ Agree<br>◯ Strongly agree
d. I plan to take care of immigrants and refugees when I finish residency.	◯ Strongly disagree<br>◯ Disagree<br>◯ No opinion<br>◯ Agree<br>⊗ Strongly agree
e. I plan to do short term (< 6 months) international work when I finish residency.	◯ Strongly disagree<br>◯ Disagree<br>◯ No opinion<br>◯ Agree<br>⊗ Strongly agree
f. I plan to do long term (>6 months) international work when I finish residency.	◯ Strongly disagree<br>◯ Disagree<br>◯ No opinion<br>◯ Agree<br>⊗ Strongly agree
g. I plan to work in health disparities in the following way after residency:	research on developing health care infrastructure in developing countries. refugee care stateside. finding ways to get care to the unreachables.

SECTION B: MEDICAL EDUCATION

A. Please indicate your level of agreement with the following statements regarding your medical education and knowledge about immigrants and refugees by checking the box that best represents your opinion.

a. I have received specialized training in immigrant and refugee health, tropical medicine, or cross-cultural health.

- ◯ Strongly disagree
- ◯ Disagree
- ◯ No opinion
- ⊗ Agree
- ◯ Strongly agree

b. If you have received specialized training in immigrant and refugee health, tropical medicine, or cross-cultural health, please indicate all the contexts in which you received this training:

- ☒ As an undergraduate.
- ☒ As a medical student.
- ☒ As part of my residency.
- ☐ A special program.
- ☐ As part of my fellowship.
- ☐ As part of a degree program (e.g. MPH)
- ☐ Other:

c. I feel comfortable with my fund of knowledge regarding immigrant and refugee health.

- ◯ Strongly disagree
- ◯ Disagree
- ◯ No opinion
- ⊗ Agree
- ◯ Strongly agree

d. I would like to have further training in immigrant and refugee health.

- ◯ Strongly disagree
- ◯ Disagree
- ◯ No opinion
- ◯ Agree
- ⊗ Strongly agree

e. If you agree with the above, please indicate all the contexts in which you would like to receive this training:

- ☒ As part of my residency.
- ☒ A special program.
- ☒ As part of my fellowship.

SECTION C: Attitudes towards immigrant health

A. Please indicate your level of agreement with the following statements regarding immigrant and refugee health by checking the box that best represents your opinion.

a. I enjoy taking care of immigrants and refugees.

- ◯ Never
- ◯ Rarely
- ◯ Sometimes
- ◯ Usually
- ⊗ Always

b. Please indicate the reasons that you enjoy taking care of immigrants and refugees (may choose more than one).

- ☒ Tropical and other conditions not frequently diagnosed in US-born patients
- ☒ Learning about other cultures
- ☐ They don't complain as much
- ☐ Being able to hear their stories
- ☒ Their care is more complicated
- ☐ Their care is less complicated
- ☒ They are very appreciative of your help.
- ☒ They are extremely vulnerable
- ☐ Other:

c. Taking care of immigrants and refugees is more challenging than taking care of US born patients.

- ◯ Never
- ◯ Rarely
- ⊗ Sometimes
- ◯ Usually
- ◯ Always

d. Please mark all the challenges that you face as a provider when providing care to immigrants and refugees (may choose more than one):

- ☒ Language barriers
- ☒ Insurance barriers
- ☐ Cultural barriers
- ☒ Finding a professional interpreter
- ☐ Knowing how to work with a professional interpreter
- ☐ Time constraints
- ☐ My own knowledge related to tropical and travel medicine
- ☐ Transportation problems for the patient
- ☐ Patients not understanding treatment plan
- ☐ Patients not following treatment plan
- ☒ My lack of knowledge regarding the patient's culture
- ☐ Bias or stereotyping
- ☐ Other:

e. Please mark all of the challenges faced by immigrant and refugee populations when receiving healthcare that you have perceived or witnessed (may choose more than one):

- ☒ Language barriers
- ☐ Insurance barriers
- ☐ Cultural barriers
- ☐ Finding a professional interpreter
- ☐ Knowing how to work with a professional interpreter
- ☒ Time constraints
- ☐ Insufficiently trained health care providers
- ☒ Transportation problems for the patient
- ☒ Food insufficiency
- ☒ Need for child care
- ☐ Patients not understanding treatment plan
- ☐ Patients not following treatment plan
- ☐ My lack of knowledge regarding the patient's culture
- ☐ Bias or stereotyping
- ☐ Trust issues
- ☐ Other...

f. Rank how well immigrants and refugees understand the healthcare that you are trying to provide.

- ○ Significantly less than a US born individual
- ○ Less than a US born individual
- ⊗ Equivalent to a US born individual
- ○ More than a US born patient
- ○ Significantly more than a US born individual

g. Immigrants and refugees adhere to treatment plans and follow my recommendations.

- ○ Never
- ○ Rarely
- ○ Sometimes
- ⊗ Usually
- ○ Always

h. Immigrants and refugees should receive the same care and insurance coverage as US born patients.

- ○ Never
- ○ Rarely
- ○ Sometimes
- ○ Usually
- ⊗ Always

i. Immigrants and refugees who are undocumented should receive the same care and insurance coverage as US born patients.

- ○ Never
- ○ Rarely
- ○ Sometimes
- ○ Usually
- ⊗ Always

j. Every physician is professionally obligated to care for immigrants and refugees if they present to your clinic or hospital.

- ○ Strongly disagree
- ○ Disagree
- ○ No opinion
- ○ Agree
- ⊗ Strongly agree

k. Is healthcare a human right?
- ⊗ Yes
- ◯ No

B. If you wish, please tell us about what you enjoy or do not enjoy about immigrant and refugee health care and the greatest challenges you face in caring for this population.

____

SECTION D: DEMOGRAPHIC INFORMATION
Please answer the following questions by checking the box in front of the response choice that best describes you.

a. Your age?
- ◯ 20 to 24
- ⊗ 25 to 29
- ◯ 30 to 34
- ◯ 35 to 39
- ◯ 40 or older

b. Your gender?
- ◯ Female
- ⊗ Male
- ◯ Other

c. Are you Hispanic or Latino?
- ◯ Yes
- ⊗ No

d. What is your race? (Select one or more responses)
- ◯ American Indian or Alaska Native
- ◯ Asian (Please specify):
- ◯ Black or African American
- ◯ Native Hawaiian or Other Pacific Islander
- ⊗ White
- ◯ Other (Please specify):

e. Were you born in the United States?
- ⊗ Yes
- ◯ No

g. Your residency year?
- ◯ PGY1
- ⊗ PGY2
- ◯ PGY3
- ◯ PGY4
- ◯ PGY5

h. How would you classify your political ideology?
- ◯ Conservative
- ◯ Somewhat conservative
- ◯ Moderate
- ◯ Somewhat liberal
- ⊗ Liberal
- ◯ Other (Please specify):

i. Estimated level of educational debt?
- ◯ None
- ◯ Less than $50,000
- ◯ $50,000 - $100,000
- ⊗ $100,000 - $200,000
- ◯ $200,000 or more

j. Do you plan to subspecialize?
- ◯ Yes
- ⊗ No

k. Languages spoken?

- ☒ English
- ☒ Spanish
- ☒ French
- ☐ Hmong
- ☐ Somali
- ☐ Japanese
- ☐ Chinese
- ☐ Russian
- ☐ Ethiopian
- ☐ Other ___________

l. Are you in the Global Health Pathway?

- ⊗ Yes
- ◯ No

m. Did you earn your degree in the US?

- ⊗ Yes
- ◯ No

n. What residency program are you in?

- ◯ Internal Medicine
- ◯ Med-Peds
- ⊗ Pediatrics
- ◯ Family Practice
- ◯ Neurology
- ◯ Psychiatry
- ◯ ObGyn
- ◯ Neurosurgery
- ◯ General Surgery
- ◯ Orthopedic Surgery
- ◯ Urology
- ◯ Surgical sub-specialty (please specify in text box below)
- ◯ Non-clinical specialty (radiology, pathology; please specify in text box below)

Medical Trainees' attitudes, knowledge, and experience with immigrant and refugee health

Response was added on 11/03/2013 8:29am.

SECTION A: Personal experience with immigrant and refugee health care.

A. Please indicate your level of agreement with the following statements regarding your personal experience with immigrant and refugee health care by checking the box that best represents your experience.

a. During my inpatient rotations, I take care of the following percentage of immigrant and refugee patients:

- ◯ None
- ◯ 0 -5%
- ◯ 5-10%
- ⊗ 10-25%
- ◯ > 25%

b. During my outpatient rotations, I take care of the following percentage of immigrant and refugee patients:

- ◯ None
- ⊗ 0-10%
- ◯ 10 -25%
- ◯ 25-50%
- ◯ 50-75%
- ◯ >75%

c. I would like to take care of more immigrant and refugee patients.

- ◯ Strongly disagree
- ◯ Disagree
- ⊗ No opinion
- ◯ Agree
- ◯ Strongly agree

d. I plan to take care of immigrants and refugees when I finish residency.

- ◯ Strongly disagree
- ⊗ Disagree
- ◯ No opinion
- ◯ Agree
- ◯ Strongly agree

e. I plan to do short term (< 6 months) international work when I finish residency.

- ◯ Strongly disagree
- ◯ Disagree
- ◯ No opinion
- ◯ Agree
- ⊗ Strongly agree

f. I plan to do long term (>6 months) international work when I finish residency.

- ◯ Strongly disagree
- ⊗ Disagree
- ◯ No opinion
- ◯ Agree
- ◯ Strongly agree

g. I plan to work in health disparities in the following way after residency:

No

SECTION B: MEDICAL EDUCATION

A. Please indicate your level of agreement with the following statements regarding your medical education and knowledge about immigrants and refugees by checking the box that best represents your opinion.

a. I have received specialized training in immigrant and refugee health, tropical medicine, or cross-cultural health.

- ◯ Strongly disagree
- ◯ Disagree
- ◯ No opinion
- ⊗ Agree
- ◯ Strongly agree

b. If you have received specialized training in immigrant and refugee health, tropical medicine, or cross-cultural health, please indicate all the contexts in which you received this training:

- ☐ As an undergraduate.
- ☒ As a medical student.
- ☒ As part of my residency.
- ☐ A special program.
- ☐ As part of my fellowship.
- ☐ As part of a degree program (e.g. MPH)
- ☐ Other:

c. I feel comfortable with my fund of knowledge regarding immigrant and refugee health.

- ◯ Strongly disagree
- ◯ Disagree
- ◯ No opinion
- ⊗ Agree
- ◯ Strongly agree

d. I would like to have further training in immigrant and refugee health.

- ◯ Strongly disagree
- ◯ Disagree
- ◯ No opinion
- ⊗ Agree
- ◯ Strongly agree

e. If you agree with the above, please indicate all the contexts in which you would like to receive this training:

- ☒ As part of my residency.
- ☒ A special program.
- ☐ As part of my fellowship.

SECTION C: Attitudes towards immigrant health

A. Please indicate your level of agreement with the following statements regarding immigrant and refugee health by checking the box that best represents your opinion.

a. I enjoy taking care of immigrants and refugees.

- ◯ Never
- ◯ Rarely
- ◯ Sometimes
- ⊗ Usually
- ◯ Always

b. Please indicate the reasons that you enjoy taking care of immigrants and refugees (may choose more than one).

- ☒ Tropical and other conditions not frequently diagnosed in US-born patients
- ☒ Learning about other cultures
- ☐ They don't complain as much
- ☒ Being able to hear their stories
- ☐ Their care is more complicated
- ☐ Their care is less complicated
- ☒ They are very appreciative of your help.
- ☒ They are extremely vulnerable
- ☐ Other:

c. Taking care of immigrants and refugees is more challenging than taking care of US born patients.

- ◯ Never
- ◯ Rarely
- ⊗ Sometimes
- ◯ Usually
- ◯ Always

d. Please mark all the challenges that you face as a provider when providing care to immigrants and refugees (may choose more than one):

☒ Language barriers
☒ Insurance barriers
☒ Cultural barriers
☒ Finding a professional interpreter
☒ Knowing how to work with a professional interpreter
☒ Time constraints
☒ My own knowledge related to tropical and travel medicine
☒ Transportation problems for the patient
☒ Patients not understanding treatment plan
☒ Patients not following treatment plan
☒ My lack of knowledge regarding the patient's culture
☒ Bias or stereotyping
☐ Other:

e. Please mark all of the challenges faced by immigrant and refugee populations when receiving healthcare that you have perceived or witnessed (may choose more than one):

☒ Language barriers
☒ Insurance barriers
☒ Cultural barriers
☒ Finding a professional interpreter
☐ Knowing how to work with a professional interpreter
☒ Time constraints
☐ Insufficiently trained health care providers
☒ Transportation problems for the patient
☐ Food insufficiency
☐ Need for child care
☒ Patients not understanding treatment plan
☒ Patients not following treatment plan
☐ My lack of knowledge regarding the patient's culture
☐ Bias or stereotyping
☐ Trust issues
☐ Other...

f. Rank how well immigrants and refugees understand the healthcare that you are trying to provide.

◯ Significantly less than a US born individual
⊗ Less than a US born individual
◯ Equivalent to a US born individual
◯ More than a US born patient
◯ Significantly more than a US born individual

g. Immigrants and refugees adhere to treatment plans and follow my recommendations.

◯ Never
◯ Rarely
⊗ Sometimes
◯ Usually
◯ Always

h. Immigrants and refugees should receive the same care and insurance coverage as US born patients.

◯ Never
◯ Rarely
◯ Sometimes
◯ Usually
⊗ Always

i. Immigrants and refugees who are undocumented should receive the same care and insurance coverage as US born patients.

◯ Never
◯ Rarely
◯ Sometimes
◯ Usually
⊗ Always

j. Every physician is professionally obligated to care for immigrants and refugees if they present to your clinic or hospital.

◯ Strongly disagree
◯ Disagree
◯ No opinion
◯ Agree
⊗ Strongly agree

k. Is healthcare a human right?
⊗ Yes
◯ No

B. If you wish, please tell us about what you enjoy or do not enjoy about immigrant and refugee health care and the greatest challenges you face in caring for this population.
____

SECTION D: DEMOGRAPHIC INFORMATION
Please answer the following questions by checking the box in front of the response choice that best describes you.

a. Your age?
◯ 20 to 24
◯ 25 to 29
⊗ 30 to 34
◯ 35 to 39
◯ 40 or older

b. Your gender?
◯ Female
⊗ Male
◯ Other

c.Are you Hispanic or Latino?
◯ Yes
⊗ No

d. What is your race? (Select one or more responses)
◯ American Indian or Alaska Native
◯ Asian (Please specify):
◯ Black or African American
◯ Native Hawaiian or Other Pacific Islander
⊗ White
◯ Other (Please specify):

e.Were you born in the United States?
⊗ Yes
◯ No

g. Your residency year?
◯ PGY1
⊗ PGY2
◯ PGY3
◯ PGY4
◯ PGY5

h. How would you classify your political ideology?
◯ Conservative
◯ Somewhat conservative
◯ Moderate
⊗ Somewhat liberal
◯ Liberal
◯ Other (Please specify):

i. Estimated level of educational debt?
◯ None
◯ Less than $50,000
◯ $50,000 - $100,000
◯ $100,000 - $200,000
⊗ $200,000 or more

j.Do you plan to subspecialize?
◯ Yes
⊗ No

k. Languages spoken?

- ☒ English
- ☐ Spanish
- ☐ French
- ☐ Hmong
- ☐ Somali
- ☐ Japanese
- ☐ Chinese
- ☐ Russian
- ☐ Ethiopian
- ☐ Other ___________

l. Are you in the Global Health Pathway?

- ○ Yes
- ⊗ No

m. Did you earn your degree in the US?

- ⊗ Yes
- ○ No

n. What residency program are you in?

- ○ Internal Medicine
- ○ Med-Peds
- ○ Pediatrics
- ⊗ Family Practice
- ○ Neurology
- ○ Psychiatry
- ○ ObGyn
- ○ Neurosurgery
- ○ General Surgery
- ○ Orthopedic Surgery
- ○ Urology
- ○ Surgical sub-specialty (please specify in text box below)
- ○ Non-clinical specialty (radiology, pathology; please specify in text box below)

Medical Trainees' attitudes, knowledge, and experience with immigrant and refugee health

Response was added on 11/03/2013 8:44am.

SECTION A: Personal experience with immigrant and refugee health care.

A. Please indicate your level of agreement with the following statements regarding your personal experience with immigrant and refugee health care by checking the box that best represents your experience.

a. During my inpatient rotations, I take care of the following percentage of immigrant and refugee patients:

- ◯ None
- ◯ 0 -5%
- ◯ 5-10%
- ⊗ 10-25%
- ◯ > 25%

b. During my outpatient rotations, I take care of the following percentage of immigrant and refugee patients:

- ◯ None
- ⊗ 0-10%
- ◯ 10 -25%
- ◯ 25-50%
- ◯ 50-75%
- ◯ >75%

c. I would like to take care of more immigrant and refugee patients.

- ◯ Strongly disagree
- ◯ Disagree
- ⊗ No opinion
- ◯ Agree
- ◯ Strongly agree

d. I plan to take care of immigrants and refugees when I finish residency.

- ◯ Strongly disagree
- ◯ Disagree
- ⊗ No opinion
- ◯ Agree
- ◯ Strongly agree

e. I plan to do short term (< 6 months) international work when I finish residency.

- ◯ Strongly disagree
- ◯ Disagree
- ◯ No opinion
- ⊗ Agree
- ◯ Strongly agree

f. I plan to do long term (>6 months) international work when I finish residency.

- ◯ Strongly disagree
- ◯ Disagree
- ⊗ No opinion
- ◯ Agree
- ◯ Strongly agree

g. I plan to work in health disparities in the following way after residency:

international work

SECTION B: MEDICAL EDUCATION

A. Please indicate your level of agreement with the following statements regarding your medical education and knowledge about immigrants and refugees by checking the box that best represents your opinion.

a. I have received specialized training in immigrant and refugee health, tropical medicine, or cross-cultural health.

- ◯ Strongly disagree
- ⊗ Disagree
- ◯ No opinion
- ◯ Agree
- ◯ Strongly agree

c. I feel comfortable with my fund of knowledge regarding immigrant and refugee health.

- ◯ Strongly disagree
- ⊗ Disagree
- ◯ No opinion
- ◯ Agree
- ◯ Strongly agree

d. I would like to have further training in immigrant and refugee health.

- ◯ Strongly disagree
- ◯ Disagree
- ◯ No opinion
- ⊗ Agree
- ◯ Strongly agree

e. If you agree with the above, please indicate all the contexts in which you would like to receive this training:
□□

- ☒ As part of my residency.
- ☐ A special program.
- ☐ As part of my fellowship.

SECTION C: Attitudes towards immigrant health

A.□Please indicate your level of agreement with the following statements regarding immigrant and refugee health by checking the box that best represents your opinion.

a. I enjoy taking care of immigrants and refugees.

- ◯ Never
- ◯ Rarely
- ⊗ Sometimes
- ◯ Usually
- ◯ Always

b. Please indicate the reasons that you enjoy taking care of immigrants and refugees (may choose more than one).

- ☐ Tropical and other conditions not frequently diagnosed in US-born patients
- ☒ Learning about other cultures
- ☐ They don't complain as much
- ☒ Being able to hear their stories
- ☐ Their care is more complicated
- ☐ Their care is less complicated
- ☐ They are very appreciative of your help.
- ☐ They are extremely vulnerable
- ☐ Other:

c. Taking care of immigrants and refugees is more challenging than taking care of US born patients.

- ◯ Never
- ◯ Rarely
- ⊗ Sometimes
- ◯ Usually
- ◯ Always

d. Please mark all the challenges that you face as a provider when providing care to immigrants and refugees (may choose more than one):

- ☒ Language barriers
- ☐ Insurance barriers
- ☒ Cultural barriers
- ☐ Finding a professional interpreter
- ☐ Knowing how to work with a professional interpreter
- ☐ Time constraints
- ☐ My own knowledge related to tropical and travel medicine
- ☐ Transportation problems for the patient
- ☐ Patients not understanding treatment plan
- ☒ Patients not following treatment plan
- ☒ My lack of knowledge regarding the patient's culture
- ☒ Bias or stereotyping
- ☐ Other:

e. Please mark all of the challenges faced by immigrant and refugee populations when receiving healthcare that you have perceived or witnessed (may choose more than one):

- ☒ Language barriers
- ☒ Insurance barriers
- ☐ Cultural barriers
- ☒ Finding a professional interpreter
- ☐ Knowing how to work with a professional interpreter
- ☒ Time constraints
- ☐ Insufficiently trained health care providers
- ☐ Transportation problems for the patient
- ☒ Food insufficiency
- ☐ Need for child care
- ☒ Patients not understanding treatment plan
- ☒ Patients not following treatment plan
- ☐ My lack of knowledge regarding the patient's culture
- ☐ Bias or stereotyping
- ☐ Trust issues
- ☐ Other...

f. Rank how well immigrants and refugees understand the healthcare that you are trying to provide.

- ◯ Significantly less than a US born individual
- ⊗ Less than a US born individual
- ◯ Equivalent to a US born individual
- ◯ More than a US born patient
- ◯ Significantly more than a US born individual

g. Immigrants and refugees adhere to treatment plans and follow my recommendations.

- ◯ Never
- ◯ Rarely
- ⊗ Sometimes
- ◯ Usually
- ◯ Always

h. Immigrants and refugees should receive the same care and insurance coverage as US born patients.

- ◯ Never
- ◯ Rarely
- ◯ Sometimes
- ⊗ Usually
- ◯ Always

i. Immigrants and refugees who are undocumented should receive the same care and insurance coverage as US born patients.

- ◯ Never
- ⊗ Rarely
- ◯ Sometimes
- ◯ Usually
- ◯ Always

j. Every physician is professionally obligated to care for immigrants and refugees if they present to your clinic or hospital.

- ◯ Strongly disagree
- ◯ Disagree
- ⊗ No opinion
- ◯ Agree
- ◯ Strongly agree

k. Is healthcare a human right?
⊗ Yes
◯ No

B. If you wish, please tell us about what you enjoy or do not enjoy about immigrant and refugee health care and the greatest challenges you face in caring for this population.
____

SECTION D: DEMOGRAPHIC INFORMATION
Please answer the following questions by checking the box in front of the response choice that best describes you.

a. Your age?
◯ 20 to 24
◯ 25 to 29
⊗ 30 to 34
◯ 35 to 39
◯ 40 or older

b. Your gender?
⊗ Female
◯ Male
◯ Other

c. Are you Hispanic or Latino?
◯ Yes
⊗ No

d. What is your race? (Select one or more responses)
◯ American Indian or Alaska Native
◯ Asian (Please specify):
◯ Black or African American
◯ Native Hawaiian or Other Pacific Islander
⊗ White
◯ Other (Please specify):

e. Were you born in the United States?
⊗ Yes
◯ No

g. Your residency year?
◯ PGY1
◯ PGY2
◯ PGY3
⊗ PGY4
◯ PGY5

h. How would you classify your political ideology?
◯ Conservative
◯ Somewhat conservative
⊗ Moderate
◯ Somewhat liberal
◯ Liberal
◯ Other (Please specify):

i. Estimated level of educational debt?
◯ None
◯ Less than $50,000
◯ $50,000 - $100,000
⊗ $100,000 - $200,000
◯ $200,000 or more

j. Do you plan to subspecialize?
◯ Yes
⊗ No

k. Languages spoken?

- ☒ English
- ☐ Spanish
- ☐ French
- ☐ Hmong
- ☐ Somali
- ☐ Japanese
- ☐ Chinese
- ☐ Russian
- ☐ Ethiopian
- ☐ Other ___________

l. Are you in the Global Health Pathway?

- ○ Yes
- ⊗ No

m.&#xFFFD; Did you earn your degree in the US?

- ⊗ Yes
- ○ No

n. What residency program are you in?

- ○ Internal Medicine
- ○ Med-Peds
- ○ Pediatrics
- ○ Family Practice
- ○ Neurology
- ○ Psychiatry
- ⊗ ObGyn
- ○ Neurosurgery
- ○ General Surgery
- ○ Orthopedic Surgery
- ○ Urology
- ○ Surgical sub-specialty (please specify in text box below)
- ○ Non-clinical specialty (radiology, pathology; please specify in text box below)

Medical Trainees' attitudes, knowledge, and experience with immigrant and refugee health

Response was added on 11/03/2013 11:32am.

SECTION A: Personal experience with immigrant and refugee health care.

A. Please indicate your level of agreement with the following statements regarding your personal experience with immigrant and refugee health care by checking the box that best represents your experience.

a. During my inpatient rotations, I take care of the following percentage of immigrant and refugee patients:

- ◯ None
- ◯ 0 -5%
- ⊗ 5-10%
- ◯ 10-25%
- ◯ > 25%

b. During my outpatient rotations, I take care of the following percentage of immigrant and refugee patients:

- ◯ None
- ◯ 0-10%
- ◯ 10 -25%
- ◯ 25-50%
- ◯ 50-75%
- ◯ >75%

c. I would like to take care of more immigrant and refugee patients.

- ◯ Strongly disagree
- ◯ Disagree
- ⊗ No opinion
- ◯ Agree
- ◯ Strongly agree

d. I plan to take care of immigrants and refugees when I finish residency.

- ◯ Strongly disagree
- ◯ Disagree
- ◯ No opinion
- ⊗ Agree
- ◯ Strongly agree

e. I plan to do short term (< 6 months) international work when I finish residency.

- ◯ Strongly disagree
- ◯ Disagree
- ◯ No opinion
- ⊗ Agree
- ◯ Strongly agree

f. I plan to do long term (>6 months) international work when I finish residency.

- ◯ Strongly disagree
- ⊗ Disagree
- ◯ No opinion
- ◯ Agree
- ◯ Strongly agree

g. I plan to work in health disparities in the following way after residency:

In Anesthesia we provide health care to all comers; plan to work internationally to to do trips to undeserved areas but realistically will do the bulk of my work in a larger hospital

SECTION B: MEDICAL EDUCATION

A. Please indicate your level of agreement with the following statements regarding your medical education and knowledge about immigrants and refugees by checking the box that best represents your opinion.

a. I have received specialized training in immigrant and refugee health, tropical medicine, or cross-cultural health.

- ◯ Strongly disagree
- ◯ Disagree
- ⊗ No opinion
- ◯ Agree
- ◯ Strongly agree

c. I feel comfortable with my fund of knowledge regarding immigrant and refugee health.

- ◯ Strongly disagree
- ◯ Disagree
- ◯ No opinion
- ⊗ Agree
- ◯ Strongly agree

d. I would like to have further training in immigrant and refugee health.

- ◯ Strongly disagree
- ⊗ Disagree
- ◯ No opinion
- ◯ Agree
- ◯ Strongly agree

SECTION C: Attitudes towards immigrant health

A.Please indicate your level of agreement with the following statements regarding immigrant and refugee health by checking the box that best represents your opinion.

a. I enjoy taking care of immigrants and refugees.

- ◯ Never
- ◯ Rarely
- ⊗ Sometimes
- ◯ Usually
- ◯ Always

b. Please indicate the reasons that you enjoy taking care of immigrants and refugees (may choose more than one).

- ☐ Tropical and other conditions not frequently diagnosed in US-born patients
- ☒ Learning about other cultures
- ☒ They don't complain as much
- ☐ Being able to hear their stories
- ☐ Their care is more complicated
- ☐ Their care is less complicated
- ☐ They are very appreciative of your help.
- ☐ They are extremely vulnerable
- ☐ Other:

c. Taking care of immigrants and refugees is more challenging than taking care of US born patients.

- ◯ Never
- ◯ Rarely
- ⊗ Sometimes
- ◯ Usually
- ◯ Always

d. Please mark all the challenges that you face as a provider when providing care to immigrants and refugees (may choose more than one):

- [x] Language barriers
- [ ] Insurance barriers
- [x] Cultural barriers
- [x] Finding a professional interpreter
- [ ] Knowing how to work with a professional interpreter
- [ ] Time constraints
- [ ] My own knowledge related to tropical and travel medicine
- [ ] Transportation problems for the patient
- [x] Patients not understanding treatment plan
- [ ] Patients not following treatment plan
- [x] My lack of knowledge regarding the patient's culture
- [x] Bias or stereotyping
- [ ] Other:

e. Please mark all of the challenges faced by immigrant and refugee populations when receiving healthcare that you have perceived or witnessed (may choose more than one):

- [x] Language barriers
- [x] Insurance barriers
- [x] Cultural barriers
- [ ] Finding a professional interpreter
- [ ] Knowing how to work with a professional interpreter
- [x] Time constraints
- [ ] Insufficiently trained health care providers
- [ ] Transportation problems for the patient
- [ ] Food insufficiency
- [x] Need for child care
- [x] Patients not understanding treatment plan
- [x] Patients not following treatment plan
- [ ] My lack of knowledge regarding the patient's culture
- [ ] Bias or stereotyping
- [x] Trust issues
- [ ] Other...

f. Rank how well immigrants and refugees understand the healthcare that you are trying to provide.

- (x) Significantly less than a US born individual
- ( ) Less than a US born individual
- ( ) Equivalent to a US born individual
- ( ) More than a US born patient
- ( ) Significantly more than a US born individual

g. Immigrants and refugees adhere to treatment plans and follow my recommendations.

- ( ) Never
- ( ) Rarely
- ( ) Sometimes
- ( ) Usually
- ( ) Always

h. Immigrants and refugees should receive the same care and insurance coverage as US born patients.

- ( ) Never
- ( ) Rarely
- ( ) Sometimes
- (x) Usually
- ( ) Always

i. Immigrants and refugees who are undocumented should receive the same care and insurance coverage as US born patients.

- ( ) Never
- ( ) Rarely
- ( ) Sometimes
- (x) Usually
- ( ) Always

j. Every physician is professionally obligated to care for immigrants and refugees if they present to your clinic or hospital.

- ( ) Strongly disagree
- ( ) Disagree
- ( ) No opinion
- ( ) Agree
- (x) Strongly agree

k. Is healthcare a human right?
- ⊗ Yes
- ○ No

B. If you wish, please tell us about what you enjoy or do not enjoy about immigrant and refugee health care and the greatest challenges you face in caring for this population.

____

SECTION D: DEMOGRAPHIC INFORMATION
Please answer the following questions by checking the box in front of the response choice that best describes you.

a. Your age?
- ○ 20 to 24
- ○ 25 to 29
- ○ 30 to 34
- ○ 35 to 39
- ○ 40 or older

b. Your gender?
- ○ Female
- ○ Male
- ○ Other

c. Are you Hispanic or Latino?
- ○ Yes
- ○ No

d. What is your race? (Select one or more responses)
- ○ American Indian or Alaska Native
- ○ Asian (Please specify):
- ○ Black or African American
- ○ Native Hawaiian or Other Pacific Islander
- ○ White
- ○ Other (Please specify):

e. Were you born in the United States?
- ○ Yes
- ○ No

g. Your residency year?
- ○ PGY1
- ○ PGY2
- ○ PGY3
- ○ PGY4
- ○ PGY5

h. How would you classify your political ideology?
- ○ Conservative
- ○ Somewhat conservative
- ○ Moderate
- ○ Somewhat liberal
- ○ Liberal
- ○ Other (Please specify):

i. Estimated level of educational debt?
- ○ None
- ○ Less than $50,000
- ○ $50,000 - $100,000
- ○ $100,000 - $200,000
- ○ $200,000 or more

j. Do you plan to subspecialize?
- ○ Yes
- ○ No

k. Languages spoken?

- ☐ English
- ☐ Spanish
- ☐ French
- ☐ Hmong
- ☐ Somali
- ☐ Japanese
- ☐ Chinese
- ☐ Russian
- ☐ Ethiopian
- ☐ Other __________

l. Are you in the Global Health Pathway?

- ◯ Yes
- ◯ No

m. Did you earn your degree in the US?

- ◯ Yes
- ◯ No

n. What residency program are you in?

- ◯ Internal Medicine
- ◯ Med-Peds
- ◯ Pediatrics
- ◯ Family Practice
- ◯ Neurology
- ◯ Psychiatry
- ◯ ObGyn
- ◯ Neurosurgery
- ◯ General Surgery
- ◯ Orthopedic Surgery
- ◯ Urology
- ◯ Surgical sub-specialty (please specify in text box below)
- ◯ Non-clinical specialty (radiology, pathology; please specify in text box below)

Medical Trainees' attitudes, knowledge, and experience with immigrant and refugee health

Response was added on 11/03/2013 11:38am.

SECTION A: Personal experience with immigrant and refugee health care.

A. Please indicate your level of agreement with the following statements regarding your personal experience with immigrant and refugee health care by checking the box that best represents your experience.

a. During my inpatient rotations, I take care of the following percentage of immigrant and refugee patients:
- ◯ None
- ◯ 0 -5%
- ⊗ 5-10%
- ◯ 10-25%
- ◯ > 25%

b. During my outpatient rotations, I take care of the following percentage of immigrant and refugee patients:
- ◯ None
- ◯ 0-10%
- ⊗ 10 -25%
- ◯ 25-50%
- ◯ 50-75%
- ◯ >75%

c. I would like to take care of more immigrant and refugee patients.
- ◯ Strongly disagree
- ◯ Disagree
- ◯ No opinion
- ⊗ Agree
- ◯ Strongly agree

d. I plan to take care of immigrants and refugees when I finish residency.
- ◯ Strongly disagree
- ◯ Disagree
- ◯ No opinion
- ⊗ Agree
- ◯ Strongly agree

e. I plan to do short term (< 6 months) international work when I finish residency.
- ◯ Strongly disagree
- ◯ Disagree
- ◯ No opinion
- ◯ Agree
- ⊗ Strongly agree

f. I plan to do long term (>6 months) international work when I finish residency.
- ◯ Strongly disagree
- ◯ Disagree
- ◯ No opinion
- ◯ Agree
- ⊗ Strongly agree

g. I plan to work in health disparities in the following way after residency:

homeless teens, international health

SECTION B: MEDICAL EDUCATION

A. Please indicate your level of agreement with the following statements regarding your medical education and knowledge about immigrants and refugees by checking the box that best represents your opinion.

a. I have received specialized training in immigrant and refugee health, tropical medicine, or cross-cultural health.

- ◯ Strongly disagree
- ◯ Disagree
- ◯ No opinion
- ⊗ Agree
- ◯ Strongly agree

b. If you have received specialized training in immigrant and refugee health, tropical medicine, or cross-cultural health, please indicate all the contexts in which you received this training:

- ☒ As an undergraduate.
- ☒ As a medical student.
- ☒ As part of my residency.
- ☐ A special program.
- ☐ As part of my fellowship.
- ☐ As part of a degree program (e.g. MPH)
- ☐ Other:

c. I feel comfortable with my fund of knowledge regarding immigrant and refugee health.

- ◯ Strongly disagree
- ⊗ Disagree
- ◯ No opinion
- ◯ Agree
- ◯ Strongly agree

d. I would like to have further training in immigrant and refugee health.

- ◯ Strongly disagree
- ◯ Disagree
- ◯ No opinion
- ⊗ Agree
- ◯ Strongly agree

e. If you agree with the above, please indicate all the contexts in which you would like to receive this training:

- ☒ As part of my residency.
- ☒ A special program.
- ☐ As part of my fellowship.

SECTION C: Attitudes towards immigrant health

A. Please indicate your level of agreement with the following statements regarding immigrant and refugee health by checking the box that best represents your opinion.

a. I enjoy taking care of immigrants and refugees.

- ◯ Never
- ◯ Rarely
- ◯ Sometimes
- ⊗ Usually
- ◯ Always

b. Please indicate the reasons that you enjoy taking care of immigrants and refugees (may choose more than one).

- ☒ Tropical and other conditions not frequently diagnosed in US-born patients
- ☒ Learning about other cultures
- ☐ They don't complain as much
- ☒ Being able to hear their stories
- ☒ Their care is more complicated
- ☐ Their care is less complicated
- ☒ They are very appreciative of your help.
- ☒ They are extremely vulnerable
- ☐ Other:

c. Taking care of immigrants and refugees is more challenging than taking care of US born patients.

- ◯ Never
- ◯ Rarely
- ◯ Sometimes
- ⊗ Usually
- ◯ Always

d. Please mark all the challenges that you face as a provider when providing care to immigrants and refugees (may choose more than one):

- ☒ Language barriers
- ☐ Insurance barriers
- ☒ Cultural barriers
- ☒ Finding a professional interpreter
- ☒ Knowing how to work with a professional interpreter
- ☒ Time constraints
- ☒ My own knowledge related to tropical and travel medicine
- ☒ Transportation problems for the patient
- ☒ Patients not understanding treatment plan
- ☐ Patients not following treatment plan
- ☒ My lack of knowledge regarding the patient's culture
- ☒ Bias or stereotyping
- ☐ Other:

e. Please mark all of the challenges faced by immigrant and refugee populations when receiving healthcare that you have perceived or witnessed (may choose more than one):

- ☒ Language barriers
- ☒ Insurance barriers
- ☒ Cultural barriers
- ☒ Finding a professional interpreter
- ☒ Knowing how to work with a professional interpreter
- ☒ Time constraints
- ☒ Insufficiently trained health care providers
- ☒ Transportation problems for the patient
- ☒ Food insufficiency
- ☒ Need for child care
- ☒ Patients not understanding treatment plan
- ☒ Patients not following treatment plan
- ☒ My lack of knowledge regarding the patient's culture
- ☒ Bias or stereotyping
- ☒ Trust issues
- ☐ Other...

f. Rank how well immigrants and refugees understand the healthcare that you are trying to provide.

- ○ Significantly less than a US born individual
- ○ Less than a US born individual
- ⊗ Equivalent to a US born individual
- ○ More than a US born patient
- ○ Significantly more than a US born individual

g. Immigrants and refugees adhere to treatment plans and follow my recommendations.

- ○ Never
- ○ Rarely
- ○ Sometimes
- ⊗ Usually
- ○ Always

h. Immigrants and refugees should receive the same care and insurance coverage as US born patients.

- ○ Never
- ○ Rarely
- ○ Sometimes
- ⊗ Usually
- ○ Always

i. Immigrants and refugees who are undocumented should receive the same care and insurance coverage as US born patients.

- ○ Never
- ○ Rarely
- ○ Sometimes
- ⊗ Usually
- ○ Always

j. Every physician is professionally obligated to care for immigrants and refugees if they present to your clinic or hospital.

- ○ Strongly disagree
- ○ Disagree
- ○ No opinion
- ○ Agree
- ⊗ Strongly agree

k. Is healthcare a human right?
⊗ Yes
◯ No

B. If you wish, please tell us about what you enjoy or do not enjoy about immigrant and refugee health care and the greatest challenges you face in caring for this population.
____

SECTION D: DEMOGRAPHIC INFORMATION
Please answer the following questions by checking the box in front of the response choice that best describes you.

a. Your age?
◯ 20 to 24
⊗ 25 to 29
◯ 30 to 34
◯ 35 to 39
◯ 40 or older

b. Your gender?
⊗ Female
◯ Male
◯ Other

c.Are you Hispanic or Latino?
◯ Yes
⊗ No

d. What is your race? (Select one or more responses)
◯ American Indian or Alaska Native
◯ Asian (Please specify):
◯ Black or African American
◯ Native Hawaiian or Other Pacific Islander
⊗ White
◯ Other (Please specify):

e.Were you born in the United States?
⊗ Yes
◯ No

g. Your residency year?
⊗ PGY1
◯ PGY2
◯ PGY3
◯ PGY4
◯ PGY5

h. How would you classify your political ideology?
◯ Conservative
◯ Somewhat conservative
⊗ Moderate
◯ Somewhat liberal
◯ Liberal
◯ Other (Please specify):

i. Estimated level of educational debt?
◯ None
◯ Less than $50,000
◯ $50,000 - $100,000
⊗ $100,000 - $200,000
◯ $200,000 or more

j.Do you plan to subspecialize?
⊗ Yes
◯ No

k. Languages spoken?

☒ English
☐ Spanish
☐ French
☐ Hmong
☐ Somali
☐ Japanese
☐ Chinese
☐ Russian
☐ Ethiopian
☐ Other ___________

l. Are you in the Global Health Pathway?

⊗ Yes
◯ No

m. Did you earn your degree in the US?

⊗ Yes
◯ No

n. What residency program are you in?

◯ Internal Medicine
◯ Med-Peds
⊗ Pediatrics
◯ Family Practice
◯ Neurology
◯ Psychiatry
◯ ObGyn
◯ Neurosurgery
◯ General Surgery
◯ Orthopedic Surgery
◯ Urology
◯ Surgical sub-specialty (please specify in text box below)
◯ Non-clinical specialty (radiology, pathology; please specify in text box below)

Medical Trainees' attitudes, knowledge, and experience with immigrant and refugee health

Response was added on 11/03/2013 12:13pm.

SECTION A: Personal experience with immigrant and refugee health care.

A. Please indicate your level of agreement with the following statements regarding your personal experience with immigrant and refugee health care by checking the box that best represents your experience.

a. During my inpatient rotations, I take care of the following percentage of immigrant and refugee patients:

- ◯ None
- ◯ 0 -5%
- ◯ 5-10%
- ⊗ 10-25%
- ◯ > 25%

b. During my outpatient rotations, I take care of the following percentage of immigrant and refugee patients:

- ◯ None
- ◯ 0-10%
- ⊗ 10 -25%
- ◯ 25-50%
- ◯ 50-75%
- ◯ >75%

c. I would like to take care of more immigrant and refugee patients.

- ◯ Strongly disagree
- ◯ Disagree
- ◯ No opinion
- ⊗ Agree
- ◯ Strongly agree

d. I plan to take care of immigrants and refugees when I finish residency.

- ◯ Strongly disagree
- ◯ Disagree
- ◯ No opinion
- ⊗ Agree
- ◯ Strongly agree

e. I plan to do short term (< 6 months) international work when I finish residency.

- ◯ Strongly disagree
- ◯ Disagree
- ◯ No opinion
- ◯ Agree
- ⊗ Strongly agree

f. I plan to do long term (>6 months) international work when I finish residency.

- ◯ Strongly disagree
- ◯ Disagree
- ◯ No opinion
- ◯ Agree
- ⊗ Strongly agree

g. I plan to work in health disparities in the following way after residency:

working rurally and also working internationally

SECTION B: MEDICAL EDUCATION

A. Please indicate your level of agreement with the following statements regarding your medical education and knowledge about immigrants and refugees by checking the box that best represents your opinion.

a. I have received specialized training in immigrant and refugee health, tropical medicine, or cross-cultural health.

- ◯ Strongly disagree
- ◯ Disagree
- ◯ No opinion
- ⊗ Agree
- ◯ Strongly agree

b. If you have received specialized training in immigrant and refugee health, tropical medicine, or cross-cultural health, please indicate all the contexts in which you received this training:

- ☒ As an undergraduate.
- ☒ As a medical student.
- ☒ As part of my residency.
- ☐ A special program.
- ☐ As part of my fellowship.
- ☒ As part of a degree program (e.g. MPH)
- ☐ Other:

c. I feel comfortable with my fund of knowledge regarding immigrant and refugee health.

- ◯ Strongly disagree
- ◯ Disagree
- ◯ No opinion
- ◯ Agree
- ⊗ Strongly agree

d. I would like to have further training in immigrant and refugee health.

- ◯ Strongly disagree
- ◯ Disagree
- ◯ No opinion
- ⊗ Agree
- ◯ Strongly agree

e. If you agree with the above, please indicate all the contexts in which you would like to receive this training:
□□

- ☒ As part of my residency.
- ☐ A special program.
- ☐ As part of my fellowship.

SECTION C: Attitudes towards immigrant health

A.□Please indicate your level of agreement with the following statements regarding immigrant and refugee health by checking the box that best represents your opinion.

a. I enjoy taking care of immigrants and refugees.

- ◯ Never
- ◯ Rarely
- ◯ Sometimes
- ◯ Usually
- ⊗ Always

b. Please indicate the reasons that you enjoy taking care of immigrants and refugees (may choose more than one).

- ☒ Tropical and other conditions not frequently diagnosed in US-born patients
- ☒ Learning about other cultures
- ☐ They don't complain as much
- ☒ Being able to hear their stories
- ☐ Their care is more complicated
- ☐ Their care is less complicated
- ☒ They are very appreciative of your help.
- ☒ They are extremely vulnerable
- ☐ Other:

c. Taking care of immigrants and refugees is more challenging than taking care of US born patients.

- ◯ Never
- ◯ Rarely
- ⊗ Sometimes
- ◯ Usually
- ◯ Always

d. Please mark all the challenges that you face as a provider when providing care to immigrants and refugees (may choose more than one):

- ☒ Language barriers
- ☐ Insurance barriers
- ☐ Cultural barriers
- ☐ Finding a professional interpreter
- ☐ Knowing how to work with a professional interpreter
- ☐ Time constraints
- ☐ My own knowledge related to tropical and travel medicine
- ☐ Transportation problems for the patient
- ☐ Patients not understanding treatment plan
- ☒ Patients not following treatment plan
- ☒ My lack of knowledge regarding the patient's culture
- ☐ Bias or stereotyping
- ☐ Other:

e. Please mark all of the challenges faced by immigrant and refugee populations when receiving healthcare that you have perceived or witnessed (may choose more than one):

- ☒ Language barriers
- ☐ Insurance barriers
- ☒ Cultural barriers
- ☐ Finding a professional interpreter
- ☒ Knowing how to work with a professional interpreter
- ☐ Time constraints
- ☐ Insufficiently trained health care providers
- ☒ Transportation problems for the patient
- ☐ Food insufficiency
- ☒ Need for child care
- ☒ Patients not understanding treatment plan
- ☒ Patients not following treatment plan
- ☐ My lack of knowledge regarding the patient's culture
- ☐ Bias or stereotyping
- ☐ Trust issues
- ☐ Other...

f. Rank how well immigrants and refugees understand the healthcare that you are trying to provide.

- ○ Significantly less than a US born individual
- ⊗ Less than a US born individual
- ○ Equivalent to a US born individual
- ○ More than a US born patient
- ○ Significantly more than a US born individual

g. Immigrants and refugees adhere to treatment plans and follow my recommendations.

- ○ Never
- ○ Rarely
- ⊗ Sometimes
- ○ Usually
- ○ Always

h. Immigrants and refugees should receive the same care and insurance coverage as US born patients.

- ○ Never
- ○ Rarely
- ○ Sometimes
- ○ Usually
- ⊗ Always

i. Immigrants and refugees who are undocumented should receive the same care and insurance coverage as US born patients.

- ○ Never
- ○ Rarely
- ○ Sometimes
- ○ Usually
- ⊗ Always

j. Every physician is professionally obligated to care for immigrants and refugees if they present to your clinic or hospital.

- ○ Strongly disagree
- ○ Disagree
- ○ No opinion
- ○ Agree
- ⊗ Strongly agree

k. Is healthcare a human right?	⊗ Yes<br>◯ No
B. If you wish, please tell us about what you enjoy or do not enjoy about immigrant and refugee health care and the greatest challenges you face in caring for this population.	learning about different cultures, values and belief systems. trying to work with them to navigate the US health system

SECTION D: DEMOGRAPHIC INFORMATION
Please answer the following questions by checking the box in front of the response choice that best describes you.

a. Your age?	◯ 20 to 24<br>◯ 25 to 29<br>◯ 30 to 34<br>⊗ 35 to 39<br>◯ 40 or older
b. Your gender?	⊗ Female<br>◯ Male<br>◯ Other
c.Are you Hispanic or Latino?	◯ Yes<br>⊗ No
d. What is your race? (Select one or more responses)	◯ American Indian or Alaska Native<br>◯ Asian (Please specify):<br>◯ Black or African American<br>◯ Native Hawaiian or Other Pacific Islander<br>◯ White<br>⊗ Other (Please specify):<br><br>asian indian
e.Were you born in the United States?	◯ Yes<br>⊗ No
f.If not, in what country were you born?	India
g. Your residency year?	◯ PGY1<br>◯ PGY2<br>⊗ PGY3<br>◯ PGY4<br>◯ PGY5
h. How would you classify your political ideology?	◯ Conservative<br>◯ Somewhat conservative<br>◯ Moderate<br>◯ Somewhat liberal<br>⊗ Liberal<br>◯ Other (Please specify):
i. Estimated level of educational debt?	◯ None<br>⊗ Less than $50,000<br>◯ $50,000 - $100,000<br>◯ $100,000 - $200,000<br>◯ $200,000 or more
j.Do you plan to subspecialize?	◯ Yes<br>⊗ No

k. Languages spoken?

☒ English
☐ Spanish
☐ French
☐ Hmong
☐ Somali
☐ Japanese
☐ Chinese
☐ Russian
☐ Ethiopian
☒ Other ___________

hindi, marathi, german

l. Are you in the Global Health Pathway?

◯ Yes
⊗ No

m. Did you earn your degree in the US?

⊗ Yes
◯ No

n. What residency program are you in?

◯ Internal Medicine
◯ Med-Peds
◯ Pediatrics
⊗ Family Practice
◯ Neurology
◯ Psychiatry
◯ ObGyn
◯ Neurosurgery
◯ General Surgery
◯ Orthopedic Surgery
◯ Urology
◯ Surgical sub-specialty (please specify in text box below)
◯ Non-clinical specialty (radiology, pathology; please specify in text box below)

Medical Trainees' attitudes, knowledge, and experience with immigrant and refugee health

Response was added on 11/03/2013 12:17pm.

SECTION A: Personal experience with immigrant and refugee health care.

A. Please indicate your level of agreement with the following statements regarding your personal experience with immigrant and refugee health care by checking the box that best represents your experience.

a. During my inpatient rotations, I take care of the following percentage of immigrant and refugee patients:

- ○ None
- ⊗ 0 -5%
- ○ 5-10%
- ○ 10-25%
- ○ > 25%

b. During my outpatient rotations, I take care of the following percentage of immigrant and refugee patients:

- ○ None
- ○ 0-10%
- ⊗ 10 -25%
- ○ 25-50%
- ○ 50-75%
- ○ >75%

c. I would like to take care of more immigrant and refugee patients.

- ○ Strongly disagree
- ○ Disagree
- ⊗ No opinion
- ○ Agree
- ○ Strongly agree

d. I plan to take care of immigrants and refugees when I finish residency.

- ○ Strongly disagree
- ○ Disagree
- ⊗ No opinion
- ○ Agree
- ○ Strongly agree

e. I plan to do short term (< 6 months) international work when I finish residency.

- ○ Strongly disagree
- ○ Disagree
- ⊗ No opinion
- ○ Agree
- ○ Strongly agree

f. I plan to do long term (>6 months) international work when I finish residency.

- ○ Strongly disagree
- ⊗ Disagree
- ○ No opinion
- ○ Agree
- ○ Strongly agree

g. I plan to work in health disparities in the following way after residency:

na

SECTION B: MEDICAL EDUCATION

A. Please indicate your level of agreement with the following statements regarding your medical education and knowledge about immigrants and refugees by checking the box that best represents your opinion.

a. I have received specialized training in immigrant and refugee health, tropical medicine, or cross-cultural health.

- ◯ Strongly disagree
- ◯ Disagree
- ◯ No opinion
- ⊗ Agree
- ◯ Strongly agree

b. If you have received specialized training in immigrant and refugee health, tropical medicine, or cross-cultural health, please indicate all the contexts in which you received this training:

- ☐ As an undergraduate.
- ☐ As a medical student.
- ☒ As part of my residency.
- ☐ A special program.
- ☐ As part of my fellowship.
- ☐ As part of a degree program (e.g. MPH)
- ☐ Other:

c. I feel comfortable with my fund of knowledge regarding immigrant and refugee health.

- ◯ Strongly disagree
- ⊗ Disagree
- ◯ No opinion
- ◯ Agree
- ◯ Strongly agree

d. I would like to have further training in immigrant and refugee health.

- ◯ Strongly disagree
- ◯ Disagree
- ◯ No opinion
- ⊗ Agree
- ◯ Strongly agree

e. If you agree with the above, please indicate all the contexts in which you would like to receive this training:
▯▯

- ☒ As part of my residency.
- ☐ A special program.
- ☐ As part of my fellowship.

SECTION C: Attitudes towards immigrant health

A.▯Please indicate your level of agreement with the following statements regarding immigrant and refugee health by checking the box that best represents your opinion.

a. I enjoy taking care of immigrants and refugees.

- ◯ Never
- ◯ Rarely
- ⊗ Sometimes
- ◯ Usually
- ◯ Always

b. Please indicate the reasons that you enjoy taking care of immigrants and refugees (may choose more than one).

- ☐ Tropical and other conditions not frequently diagnosed in US-born patients
- ☒ Learning about other cultures
- ☐ They don't complain as much
- ☐ Being able to hear their stories
- ☐ Their care is more complicated
- ☐ Their care is less complicated
- ☒ They are very appreciative of your help.
- ☐ They are extremely vulnerable
- ☐ Other:

c. Taking care of immigrants and refugees is more challenging than taking care of US born patients.

- ◯ Never
- ◯ Rarely
- ⊗ Sometimes
- ◯ Usually
- ◯ Always

d. Please mark all the challenges that you face as a provider when providing care to immigrants and refugees (may choose more than one):

- ☒ Language barriers
- ☒ Insurance barriers
- ☒ Cultural barriers
- ☒ Finding a professional interpreter
- ☐ Knowing how to work with a professional interpreter
- ☐ Time constraints
- ☒ My own knowledge related to tropical and travel medicine
- ☐ Transportation problems for the patient
- ☐ Patients not understanding treatment plan
- ☐ Patients not following treatment plan
- ☒ My lack of knowledge regarding the patient's culture
- ☐ Bias or stereotyping
- ☐ Other:

e. Please mark all of the challenges faced by immigrant and refugee populations when receiving healthcare that you have perceived or witnessed (may choose more than one):

- ☒ Language barriers
- ☐ Insurance barriers
- ☒ Cultural barriers
- ☐ Finding a professional interpreter
- ☐ Knowing how to work with a professional interpreter
- ☒ Time constraints
- ☒ Insufficiently trained health care providers
- ☐ Transportation problems for the patient
- ☐ Food insufficiency
- ☐ Need for child care
- ☒ Patients not understanding treatment plan
- ☒ Patients not following treatment plan
- ☐ My lack of knowledge regarding the patient's culture
- ☐ Bias or stereotyping
- ☒ Trust issues
- ☐ Other...

f. Rank how well immigrants and refugees understand the healthcare that you are trying to provide.

- ○ Significantly less than a US born individual
- ⊗ Less than a US born individual
- ○ Equivalent to a US born individual
- ○ More than a US born patient
- ○ Significantly more than a US born individual

g. Immigrants and refugees adhere to treatment plans and follow my recommendations.

- ○ Never
- ○ Rarely
- ⊗ Sometimes
- ○ Usually
- ○ Always

h. Immigrants and refugees should receive the same care and insurance coverage as US born patients.

- ○ Never
- ○ Rarely
- ○ Sometimes
- ⊗ Usually
- ○ Always

i. Immigrants and refugees who are undocumented should receive the same care and insurance coverage as US born patients.

- ○ Never
- ○ Rarely
- ○ Sometimes
- ⊗ Usually
- ○ Always

j. Every physician is professionally obligated to care for immigrants and refugees if they present to your clinic or hospital.

- ○ Strongly disagree
- ○ Disagree
- ○ No opinion
- ○ Agree
- ⊗ Strongly agree

k. Is healthcare a human right?
⊗ Yes
◯ No

B. If you wish, please tell us about what you enjoy or do not enjoy about immigrant and refugee health care and the greatest challenges you face in caring for this population.
____

SECTION D: DEMOGRAPHIC INFORMATION
Please answer the following questions by checking the box in front of the response choice that best describes you.

a. Your age?
◯ 20 to 24
⊗ 25 to 29
◯ 30 to 34
◯ 35 to 39
◯ 40 or older

b. Your gender?
⊗ Female
◯ Male
◯ Other

c. Are you Hispanic or Latino?
◯ Yes
⊗ No

d. What is your race? (Select one or more responses)
◯ American Indian or Alaska Native
◯ Asian (Please specify):
◯ Black or African American
◯ Native Hawaiian or Other Pacific Islander
⊗ White
◯ Other (Please specify):

e. Were you born in the United States?
⊗ Yes
◯ No

g. Your residency year?
◯ PGY1
◯ PGY2
⊗ PGY3
◯ PGY4
◯ PGY5

h. How would you classify your political ideology?
◯ Conservative
◯ Somewhat conservative
◯ Moderate
⊗ Somewhat liberal
◯ Liberal
◯ Other (Please specify):

i. Estimated level of educational debt?
◯ None
◯ Less than $50,000
◯ $50,000 - $100,000
⊗ $100,000 - $200,000
◯ $200,000 or more

j. Do you plan to subspecialize?
◯ Yes
⊗ No

k. Languages spoken?

- ☒ English
- ☐ Spanish
- ☐ French
- ☐ Hmong
- ☐ Somali
- ☐ Japanese
- ☐ Chinese
- ☐ Russian
- ☐ Ethiopian
- ☐ Other ___________

l. Are you in the Global Health Pathway?

- ◯ Yes
- ⊗ No

m. Did you earn your degree in the US?

- ⊗ Yes
- ◯ No

n. What residency program are you in?

- ◯ Internal Medicine
- ◯ Med-Peds
- ⊗ Pediatrics
- ◯ Family Practice
- ◯ Neurology
- ◯ Psychiatry
- ◯ ObGyn
- ◯ Neurosurgery
- ◯ General Surgery
- ◯ Orthopedic Surgery
- ◯ Urology
- ◯ Surgical sub-specialty (please specify in text box below)
- ◯ Non-clinical specialty (radiology, pathology; please specify in text box below)

Medical Trainees' attitudes, knowledge, and experience with immigrant and refugee health

Response was added on 11/03/2013 1:38pm.

SECTION A: Personal experience with immigrant and refugee health care.

A. Please indicate your level of agreement with the following statements regarding your personal experience with immigrant and refugee health care by checking the box that best represents your experience.

a. During my inpatient rotations, I take care of the following percentage of immigrant and refugee patients:

- ◯ None
- ⊗ 0 -5%
- ◯ 5-10%
- ◯ 10-25%
- ◯ > 25%

b. During my outpatient rotations, I take care of the following percentage of immigrant and refugee patients:

- ◯ None
- ◯ 0-10%
- ⊗ 10 -25%
- ◯ 25-50%
- ◯ 50-75%
- ◯ >75%

c. I would like to take care of more immigrant and refugee patients.

- ◯ Strongly disagree
- ◯ Disagree
- ⊗ No opinion
- ◯ Agree
- ◯ Strongly agree

d. I plan to take care of immigrants and refugees when I finish residency.

- ◯ Strongly disagree
- ◯ Disagree
- ⊗ No opinion
- ◯ Agree
- ◯ Strongly agree

e. I plan to do short term (< 6 months) international work when I finish residency.

- ◯ Strongly disagree
- ◯ Disagree
- ◯ No opinion
- ⊗ Agree
- ◯ Strongly agree

f. I plan to do long term (>6 months) international work when I finish residency.

- ◯ Strongly disagree
- ⊗ Disagree
- ◯ No opinion
- ◯ Agree
- ◯ Strongly agree

g. I plan to work in health disparities in the following way after residency:

Remote areas of east Africa most likely. Short surgical trips.

SECTION B: MEDICAL EDUCATION

A. Please indicate your level of agreement with the following statements regarding your medical education and knowledge about immigrants and refugees by checking the box that best represents your opinion.

a. I have received specialized training in immigrant and refugee health, tropical medicine, or cross-cultural health.

- ◯ Strongly disagree
- ⊗ Disagree
- ⊗ No opinion
- ◯ Agree
- ◯ Strongly agree

c. I feel comfortable with my fund of knowledge regarding immigrant and refugee health.

- ◯ Strongly disagree
- ◯ Disagree
- ⊗ No opinion
- ◯ Agree
- ◯ Strongly agree

d. I would like to have further training in immigrant and refugee health.

- ◯ Strongly disagree
- ⊗ Disagree
- ◯ No opinion
- ◯ Agree
- ◯ Strongly agree

SECTION C: Attitudes towards immigrant health

A.Please indicate your level of agreement with the following statements regarding immigrant and refugee health by checking the box that best represents your opinion.

a. I enjoy taking care of immigrants and refugees.

- ◯ Never
- ◯ Rarely
- ◯ Sometimes
- ⊗ Usually
- ◯ Always

b. Please indicate the reasons that you enjoy taking care of immigrants and refugees (may choose more than one).

- ☐ Tropical and other conditions not frequently diagnosed in US-born patients
- ☒ Learning about other cultures
- ☒ They don't complain as much
- ☒ Being able to hear their stories
- ☐ Their care is more complicated
- ☐ Their care is less complicated
- ☒ They are very appreciative of your help.
- ☐ They are extremely vulnerable
- ☐ Other:

c. Taking care of immigrants and refugees is more challenging than taking care of US born patients.

- ◯ Never
- ⊗ Rarely
- ◯ Sometimes
- ◯ Usually
- ◯ Always

d. Please mark all the challenges that you face as a provider when providing care to immigrants and refugees (may choose more than one):

- ☒ Language barriers
- ☐ Insurance barriers
- ☒ Cultural barriers
- ☒ Finding a professional interpreter
- ☐ Knowing how to work with a professional interpreter
- ☐ Time constraints
- ☐ My own knowledge related to tropical and travel medicine
- ☐ Transportation problems for the patient
- ☒ Patients not understanding treatment plan
- ☒ Patients not following treatment plan
- ☒ My lack of knowledge regarding the patient's culture
- ☐ Bias or stereotyping
- ☐ Other:

e. Please mark all of the challenges faced by immigrant and refugee populations when receiving healthcare that you have perceived or witnessed (may choose more than one):

- ☒ Language barriers
- ☐ Insurance barriers
- ☐ Cultural barriers
- ☒ Finding a professional interpreter
- ☐ Knowing how to work with a professional interpreter
- ☐ Time constraints
- ☐ Insufficiently trained health care providers
- ☐ Transportation problems for the patient
- ☒ Food insufficiency
- ☐ Need for child care
- ☒ Patients not understanding treatment plan
- ☒ Patients not following treatment plan
- ☒ My lack of knowledge regarding the patient's culture
- ☐ Bias or stereotyping
- ☐ Trust issues
- ☐ Other...

f. Rank how well immigrants and refugees understand the healthcare that you are trying to provide.

- ◯ Significantly less than a US born individual
- ⊗ Less than a US born individual
- ◯ Equivalent to a US born individual
- ◯ More than a US born patient
- ◯ Significantly more than a US born individual

g. Immigrants and refugees adhere to treatment plans and follow my recommendations.

- ◯ Never
- ◯ Rarely
- ◯ Sometimes
- ⊗ Usually
- ◯ Always

h. Immigrants and refugees should receive the same care and insurance coverage as US born patients.

- ◯ Never
- ◯ Rarely
- ◯ Sometimes
- ◯ Usually
- ⊗ Always

i. Immigrants and refugees who are undocumented should receive the same care and insurance coverage as US born patients.

- ◯ Never
- ◯ Rarely
- ◯ Sometimes
- ◯ Usually
- ⊗ Always

j. Every physician is professionally obligated to care for immigrants and refugees if they present to your clinic or hospital.

- ◯ Strongly disagree
- ◯ Disagree
- ◯ No opinion
- ◯ Agree
- ⊗ Strongly agree

k. Is healthcare a human right?

- ⊗ Yes
- ◯ No

B. If you wish, please tell us about what you enjoy or do not enjoy about immigrant and refugee health care and the greatest challenges you face in caring for this population.

____

SECTION D: DEMOGRAPHIC INFORMATION
Please answer the following questions by checking the box in front of the response choice that best describes you.

a. Your age?
- ◯ 20 to 24
- ◯ 25 to 29
- ⊗ 30 to 34
- ◯ 35 to 39
- ◯ 40 or older

b. Your gender?
- ◯ Female
- ⊗ Male
- ◯ Other

c. Are you Hispanic or Latino?
- ◯ Yes
- ⊗ No

d. What is your race? (Select one or more responses)
- ◯ American Indian or Alaska Native
- ◯ Asian (Please specify):
- ◯ Black or African American
- ◯ Native Hawaiian or Other Pacific Islander
- ⊗ White
- ◯ Other (Please specify):

e. Were you born in the United States?
- ⊗ Yes
- ◯ No

g. Your residency year?
- ⊗ PGY1
- ◯ PGY2
- ◯ PGY3
- ◯ PGY4
- ◯ PGY5

h. How would you classify your political ideology?
- ◯ Conservative
- ◯ Somewhat conservative
- ◯ Moderate
- ◯ Somewhat liberal
- ◯ Liberal
- ⊗ Other (Please specify):

Libertarian

i. Estimated level of educational debt?
- ◯ None
- ◯ Less than $50,000
- ◯ $50,000 - $100,000
- ◯ $100,000 - $200,000
- ⊗ $200,000 or more

j. Do you plan to subspecialize?
- ◯ Yes
- ⊗ No

k. Languages spoken?
- ☒ English
- ☐ Spanish
- ☐ French
- ☐ Hmong
- ☐ Somali
- ☐ Japanese
- ☐ Chinese
- ☐ Russian
- ☐ Ethiopian
- ☐ Other ___________

l. Are you in the Global Health Pathway?
- ◯ Yes
- ⊗ No

m. Did you earn your degree in the US?

⊗ Yes
◯ No

n. What residency program are you in?

◯ Internal Medicine
◯ Med-Peds
◯ Pediatrics
◯ Family Practice
◯ Neurology
◯ Psychiatry
◯ ObGyn
◯ Neurosurgery
◯ General Surgery
⊗ Orthopedic Surgery
◯ Urology
◯ Surgical sub-specialty (please specify in text box below)
◯ Non-clinical specialty (radiology, pathology; please specify in text box below)

Medical Trainees' attitudes, knowledge, and experience with immigrant and refugee health

Response was added on 11/03/2013 5:05pm.

SECTION A: Personal experience with immigrant and refugee health care.

A. Please indicate your level of agreement with the following statements regarding your personal experience with immigrant and refugee health care by checking the box that best represents your experience.

a. During my inpatient rotations, I take care of the following percentage of immigrant and refugee patients:
- ◯ None
- ◯ 0 -5%
- ◯ 5-10%
- ⊗ 10-25%
- ◯ > 25%

b. During my outpatient rotations, I take care of the following percentage of immigrant and refugee patients:
- ◯ None
- ◯ 0-10%
- ◯ 10 -25%
- ⊗ 25-50%
- ◯ 50-75%
- ◯ >75%

c. I would like to take care of more immigrant and refugee patients.
- ◯ Strongly disagree
- ◯ Disagree
- ⊗ No opinion
- ◯ Agree
- ◯ Strongly agree

d. I plan to take care of immigrants and refugees when I finish residency.
- ◯ Strongly disagree
- ◯ Disagree
- ⊗ No opinion
- ◯ Agree
- ◯ Strongly agree

e. I plan to do short term (< 6 months) international work when I finish residency.
- ◯ Strongly disagree
- ◯ Disagree
- ◯ No opinion
- ⊗ Agree
- ◯ Strongly agree

f. I plan to do long term (>6 months) international work when I finish residency.
- ◯ Strongly disagree
- ⊗ Disagree
- ◯ No opinion
- ◯ Agree
- ◯ Strongly agree

g. I plan to work in health disparities in the following way after residency:

under served area

SECTION B: MEDICAL EDUCATION

A. Please indicate your level of agreement with the following statements regarding your medical education and knowledge about immigrants and refugees by checking the box that best represents your opinion.

a. I have received specialized training in immigrant and refugee health, tropical medicine, or cross-cultural health.

- ◯ Strongly disagree
- ⊗ Disagree
- ◯ No opinion
- ◯ Agree
- ◯ Strongly agree

c. I feel comfortable with my fund of knowledge regarding immigrant and refugee health.

- ◯ Strongly disagree
- ◯ Disagree
- ⊗ No opinion
- ◯ Agree
- ◯ Strongly agree

d. I would like to have further training in immigrant and refugee health.

- ◯ Strongly disagree
- ◯ Disagree
- ⊗ No opinion
- ◯ Agree
- ◯ Strongly agree

SECTION C: Attitudes towards immigrant health

A.Please indicate your level of agreement with the following statements regarding immigrant and refugee health by checking the box that best represents your opinion.

a. I enjoy taking care of immigrants and refugees.

- ◯ Never
- ◯ Rarely
- ◯ Sometimes
- ⊗ Usually
- ◯ Always

b. Please indicate the reasons that you enjoy taking care of immigrants and refugees (may choose more than one).

- ☐ Tropical and other conditions not frequently diagnosed in US-born patients
- ☐ Learning about other cultures
- ☐ They don't complain as much
- ☐ Being able to hear their stories
- ☐ Their care is more complicated
- ☐ Their care is less complicated
- ☒ They are very appreciative of your help.
- ☐ They are extremely vulnerable
- ☐ Other:

c. Taking care of immigrants and refugees is more challenging than taking care of US born patients.

- ◯ Never
- ◯ Rarely
- ◯ Sometimes
- ⊗ Usually
- ◯ Always

d. Please mark all the challenges that you face as a provider when providing care to immigrants and refugees (may choose more than one):

- ☒ Language barriers
- ☐ Insurance barriers
- ☒ Cultural barriers
- ☐ Finding a professional interpreter
- ☐ Knowing how to work with a professional interpreter
- ☐ Time constraints
- ☐ My own knowledge related to tropical and travel medicine
- ☐ Transportation problems for the patient
- ☒ Patients not understanding treatment plan
- ☐ Patients not following treatment plan
- ☐ My lack of knowledge regarding the patient's culture
- ☐ Bias or stereotyping
- ☐ Other:

e. Please mark all of the challenges faced by immigrant and refugee populations when receiving healthcare that you have perceived or witnessed (may choose more than one):

- ☒ Language barriers
- ☐ Insurance barriers
- ☐ Cultural barriers
- ☐ Finding a professional interpreter
- ☐ Knowing how to work with a professional interpreter
- ☐ Time constraints
- ☐ Insufficiently trained health care providers
- ☐ Transportation problems for the patient
- ☐ Food insufficiency
- ☐ Need for child care
- ☐ Patients not understanding treatment plan
- ☐ Patients not following treatment plan
- ☐ My lack of knowledge regarding the patient's culture
- ☐ Bias or stereotyping
- ☐ Trust issues
- ☐ Other...

f. Rank how well immigrants and refugees understand the healthcare that you are trying to provide.

- ◯ Significantly less than a US born individual
- ⊗ Less than a US born individual
- ◯ Equivalent to a US born individual
- ◯ More than a US born patient
- ◯ Significantly more than a US born individual

g. Immigrants and refugees adhere to treatment plans and follow my recommendations.

- ◯ Never
- ◯ Rarely
- ◯ Sometimes
- ⊗ Usually
- ◯ Always

h. Immigrants and refugees should receive the same care and insurance coverage as US born patients.

- ◯ Never
- ◯ Rarely
- ◯ Sometimes
- ◯ Usually
- ⊗ Always

i. Immigrants and refugees who are undocumented should receive the same care and insurance coverage as US born patients.

- ⊗ Never
- ◯ Rarely
- ◯ Sometimes
- ◯ Usually
- ◯ Always

j. Every physician is professionally obligated to care for immigrants and refugees if they present to your clinic or hospital.

- ◯ Strongly disagree
- ◯ Disagree
- ◯ No opinion
- ◯ Agree
- ⊗ Strongly agree

k. Is healthcare a human right?

- ⊗ Yes
- ◯ No

B. If you wish, please tell us about what you enjoy or do not enjoy about immigrant and refugee health care and the greatest challenges you face in caring for this population.

____

SECTION D: DEMOGRAPHIC INFORMATION
Please answer the following questions by checking the box in front of the response choice that best describes you.

a. Your age?
- ◯ 20 to 24
- ⊗ 25 to 29
- ◯ 30 to 34
- ◯ 35 to 39
- ◯ 40 or older

b. Your gender?
- ◯ Female
- ⊗ Male
- ◯ Other

c. Are you Hispanic or Latino?
- ◯ Yes
- ⊗ No

d. What is your race? (Select one or more responses)
- ◯ American Indian or Alaska Native
- ◯ Asian (Please specify):
- ◯ Black or African American
- ◯ Native Hawaiian or Other Pacific Islander
- ⊗ White
- ◯ Other (Please specify):

e. Were you born in the United States?
- ⊗ Yes
- ◯ No

g. Your residency year?
- ◯ PGY1
- ◯ PGY2
- ◯ PGY3
- ⊗ PGY4
- ◯ PGY5

h. How would you classify your political ideology?
- ◯ Conservative
- ◯ Somewhat conservative
- ⊗ Moderate
- ◯ Somewhat liberal
- ◯ Liberal
- ◯ Other (Please specify):

i. Estimated level of educational debt?
- ◯ None
- ◯ Less than $50,000
- ◯ $50,000 - $100,000
- ◯ $100,000 - $200,000
- ⊗ $200,000 or more

j. Do you plan to subspecialize?
- ⊗ Yes
- ◯ No

k. Languages spoken?
- ☒ English
- ☐ Spanish
- ☐ French
- ☐ Hmong
- ☐ Somali
- ☐ Japanese
- ☐ Chinese
- ☐ Russian
- ☐ Ethiopian
- ☐ Other ___________

l. Are you in the Global Health Pathway?
- ◯ Yes
- ⊗ No

m. Did you earn your degree in the US?

- ⊗ Yes
- ◯ No

n. What residency program are you in?

- ◯ Internal Medicine
- ◯ Med-Peds
- ◯ Pediatrics
- ◯ Family Practice
- ◯ Neurology
- ◯ Psychiatry
- ◯ ObGyn
- ◯ Neurosurgery
- ◯ General Surgery
- ⊗ Orthopedic Surgery
- ◯ Urology
- ◯ Surgical sub-specialty (please specify in text box below)
- ◯ Non-clinical specialty (radiology, pathology; please specify in text box below)

Medical Trainees' attitudes, knowledge, and experience with immigrant and refugee health

Response was added on 11/03/2013 5:06pm.

SECTION A: Personal experience with immigrant and refugee health care.

A. Please indicate your level of agreement with the following statements regarding your personal experience with immigrant and refugee health care by checking the box that best represents your experience.

a. During my inpatient rotations, I take care of the following percentage of immigrant and refugee patients:

- ◯ None
- ⊗ 0 -5%
- ◯ 5-10%
- ◯ 10-25%
- ◯ > 25%

b. During my outpatient rotations, I take care of the following percentage of immigrant and refugee patients:

- ◯ None
- ⊗ 0-10%
- ◯ 10 -25%
- ◯ 25-50%
- ◯ 50-75%
- ◯ >75%

c. I would like to take care of more immigrant and refugee patients.

- ◯ Strongly disagree
- ◯ Disagree
- ◯ No opinion
- ⊗ Agree
- ◯ Strongly agree

d. I plan to take care of immigrants and refugees when I finish residency.

- ◯ Strongly disagree
- ◯ Disagree
- ◯ No opinion
- ⊗ Agree
- ◯ Strongly agree

e. I plan to do short term (< 6 months) international work when I finish residency.

- ◯ Strongly disagree
- ⊗ Disagree
- ◯ No opinion
- ◯ Agree
- ◯ Strongly agree

f. I plan to do long term (>6 months) international work when I finish residency.

- ⊗ Strongly disagree
- ◯ Disagree
- ◯ No opinion
- ◯ Agree
- ◯ Strongly agree

g. I plan to work in health disparities in the following way after residency:

Community based hosptials

SECTION B: MEDICAL EDUCATION

A. Please indicate your level of agreement with the following statements regarding your medical education and knowledge about immigrants and refugees by checking the box that best represents your opinion.

a. I have received specialized training in immigrant and refugee health, tropical medicine, or cross-cultural health.

- ◯ Strongly disagree
- ◯ Disagree
- ◯ No opinion
- ◯ Agree
- ⊗ Strongly agree

b. If you have received specialized training in immigrant and refugee health, tropical medicine, or cross-cultural health, please indicate all the contexts in which you received this training:

- ☒ As an undergraduate.
- ☒ As a medical student.
- ☒ As part of my residency.
- ☐ A special program.
- ☐ As part of my fellowship.
- ☐ As part of a degree program (e.g. MPH)
- ☐ Other:

c. I feel comfortable with my fund of knowledge regarding immigrant and refugee health.

- ◯ Strongly disagree
- ◯ Disagree
- ◯ No opinion
- ⊗ Agree
- ◯ Strongly agree

d. I would like to have further training in immigrant and refugee health.

- ◯ Strongly disagree
- ◯ Disagree
- ◯ No opinion
- ⊗ Agree
- ◯ Strongly agree

e. If you agree with the above, please indicate all the contexts in which you would like to receive this training:
□□

- ☒ As part of my residency.
- ☐ A special program.
- ☐ As part of my fellowship.

SECTION C: Attitudes towards immigrant health

A.□Please indicate your level of agreement with the following statements regarding immigrant and refugee health by checking the box that best represents your opinion.

a. I enjoy taking care of immigrants and refugees.

- ◯ Never
- ◯ Rarely
- ◯ Sometimes
- ⊗ Usually
- ◯ Always

b. Please indicate the reasons that you enjoy taking care of immigrants and refugees (may choose more than one).

- ☒ Tropical and other conditions not frequently diagnosed in US-born patients
- ☒ Learning about other cultures
- ☐ They don't complain as much
- ☐ Being able to hear their stories
- ☐ Their care is more complicated
- ☐ Their care is less complicated
- ☐ They are very appreciative of your help.
- ☒ They are extremely vulnerable
- ☐ Other:

c. Taking care of immigrants and refugees is more challenging than taking care of US born patients.

- ◯ Never
- ◯ Rarely
- ◯ Sometimes
- ⊗ Usually
- ◯ Always

d. Please mark all the challenges that you face as a provider when providing care to immigrants and refugees (may choose more than one):

- ☒ Language barriers
- ☒ Insurance barriers
- ☒ Cultural barriers
- ☒ Finding a professional interpreter
- ☐ Knowing how to work with a professional interpreter
- ☒ Time constraints
- ☐ My own knowledge related to tropical and travel medicine
- ☐ Transportation problems for the patient
- ☒ Patients not understanding treatment plan
- ☐ Patients not following treatment plan
- ☐ My lack of knowledge regarding the patient's culture
- ☒ Bias or stereotyping
- ☐ Other:

e. Please mark all of the challenges faced by immigrant and refugee populations when receiving healthcare that you have perceived or witnessed (may choose more than one):

- ☒ Language barriers
- ☒ Insurance barriers
- ☒ Cultural barriers
- ☐ Finding a professional interpreter
- ☒ Knowing how to work with a professional interpreter
- ☐ Time constraints
- ☐ Insufficiently trained health care providers
- ☒ Transportation problems for the patient
- ☒ Food insufficiency
- ☒ Need for child care
- ☒ Patients not understanding treatment plan
- ☐ Patients not following treatment plan
- ☐ My lack of knowledge regarding the patient's culture
- ☒ Bias or stereotyping
- ☐ Trust issues
- ☐ Other...

f. Rank how well immigrants and refugees understand the healthcare that you are trying to provide.

- ◯ Significantly less than a US born individual
- ⊗ Less than a US born individual
- ◯ Equivalent to a US born individual
- ◯ More than a US born patient
- ◯ Significantly more than a US born individual

g. Immigrants and refugees adhere to treatment plans and follow my recommendations.

- ◯ Never
- ◯ Rarely
- ⊗ Sometimes
- ◯ Usually
- ◯ Always

h. Immigrants and refugees should receive the same care and insurance coverage as US born patients.

- ◯ Never
- ◯ Rarely
- ◯ Sometimes
- ◯ Usually
- ⊗ Always

i. Immigrants and refugees who are undocumented should receive the same care and insurance coverage as US born patients.

- ◯ Never
- ◯ Rarely
- ◯ Sometimes
- ◯ Usually
- ⊗ Always

j. Every physician is professionally obligated to care for immigrants and refugees if they present to your clinic or hospital.

- ◯ Strongly disagree
- ◯ Disagree
- ◯ No opinion
- ◯ Agree
- ⊗ Strongly agree

k. Is healthcare a human right?

⊗ Yes
◯ No

B. If you wish, please tell us about what you enjoy or do not enjoy about immigrant and refugee health care and the greatest challenges you face in caring for this population.

In the current healthcare system there are significant time constraints. One of my largest struggles comes from obtaining the appropriate information then, trying to diagnose and provide treatment plan recommendations in a limited time frame-and that is true even without the added complexity of an interpreter. This is a vulnerable population and I wish schedulers and insurance companies could see the advantage of giving certain patients more time.

SECTION D: DEMOGRAPHIC INFORMATION
Please answer the following questions by checking the box in front of the response choice that best describes you.

a. Your age?

◯ 20 to 24
⊗ 25 to 29
◯ 30 to 34
◯ 35 to 39
◯ 40 or older

b. Your gender?

⊗ Female
◯ Male
◯ Other

c.Are you Hispanic or Latino?

◯ Yes
⊗ No

d. What is your race? (Select one or more responses)

◯ American Indian or Alaska Native
◯ Asian (Please specify):
◯ Black or African American
◯ Native Hawaiian or Other Pacific Islander
⊗ White
◯ Other (Please specify):

e.Were you born in the United States?

⊗ Yes
◯ No

g. Your residency year?

◯ PGY1
◯ PGY2
⊗ PGY3
◯ PGY4
◯ PGY5

h. How would you classify your political ideology?

◯ Conservative
◯ Somewhat conservative
◯ Moderate
⊗ Somewhat liberal
◯ Liberal
◯ Other (Please specify):

i. Estimated level of educational debt?

◯ None
◯ Less than $50,000
◯ $50,000 - $100,000
⊗ $100,000 - $200,000
◯ $200,000 or more

j.Do you plan to subspecialize?

- ◯ Yes
- ⊗ No

k. Languages spoken?

- ☒ English
- ☒ Spanish
- ☐ French
- ☐ Hmong
- ☐ Somali
- ☐ Japanese
- ☐ Chinese
- ☐ Russian
- ☐ Ethiopian
- ☐ Other ___________

l. Are you in the Global Health Pathway?

- ⊗ Yes
- ◯ No

m. Did you earn your degree in the US?

- ⊗ Yes
- ◯ No

n. What residency program are you in?

- ◯ Internal Medicine
- ⊗ Med-Peds
- ◯ Pediatrics
- ◯ Family Practice
- ◯ Neurology
- ◯ Psychiatry
- ◯ ObGyn
- ◯ Neurosurgery
- ◯ General Surgery
- ◯ Orthopedic Surgery
- ◯ Urology
- ◯ Surgical sub-specialty (please specify in text box below)
- ◯ Non-clinical specialty (radiology, pathology; please specify in text box below)

Medical Trainees' attitudes, knowledge, and experience with immigrant and refugee health

Response was added on 11/03/2013 6:42pm.

SECTION A: Personal experience with immigrant and refugee health care.

A. Please indicate your level of agreement with the following statements regarding your personal experience with immigrant and refugee health care by checking the box that best represents your experience.

a. During my inpatient rotations, I take care of the following percentage of immigrant and refugee patients:
- ◯ None
- ◯ 0 -5%
- ⊗ 5-10%
- ◯ 10-25%
- ◯ > 25%

b. During my outpatient rotations, I take care of the following percentage of immigrant and refugee patients:
- ◯ None
- ⊗ 0-10%
- ◯ 10 -25%
- ◯ 25-50%
- ◯ 50-75%
- ◯ >75%

c. I would like to take care of more immigrant and refugee patients.
- ◯ Strongly disagree
- ⊗ Disagree
- ◯ No opinion
- ◯ Agree
- ◯ Strongly agree

d. I plan to take care of immigrants and refugees when I finish residency.
- ◯ Strongly disagree
- ⊗ Disagree
- ◯ No opinion
- ◯ Agree
- ◯ Strongly agree

e. I plan to do short term (< 6 months) international work when I finish residency.
- ◯ Strongly disagree
- ⊗ Disagree
- ◯ No opinion
- ◯ Agree
- ◯ Strongly agree

f. I plan to do long term (>6 months) international work when I finish residency.
- ◯ Strongly disagree
- ⊗ Disagree
- ◯ No opinion
- ◯ Agree
- ◯ Strongly agree

g. I plan to work in health disparities in the following way after residency: Not in a significant fasion

SECTION B: MEDICAL EDUCATION

A. Please indicate your level of agreement with the following statements regarding your medical education and knowledge about immigrants and refugees by checking the box that best represents your opinion.

a. I have received specialized training in immigrant and refugee health, tropical medicine, or cross-cultural health.

- ◯ Strongly disagree
- ◯ Disagree
- ⊗ No opinion
- ◯ Agree
- ◯ Strongly agree

c. I feel comfortable with my fund of knowledge regarding immigrant and refugee health.

- ◯ Strongly disagree
- ◯ Disagree
- ⊗ No opinion
- ◯ Agree
- ◯ Strongly agree

d. I would like to have further training in immigrant and refugee health.

- ◯ Strongly disagree
- ⊗ Disagree
- ◯ No opinion
- ◯ Agree
- ◯ Strongly agree

SECTION C: Attitudes towards immigrant health

A.Please indicate your level of agreement with the following statements regarding immigrant and refugee health by checking the box that best represents your opinion.

a. I enjoy taking care of immigrants and refugees.

- ◯ Never
- ◯ Rarely
- ⊗ Sometimes
- ◯ Usually
- ◯ Always

b. Please indicate the reasons that you enjoy taking care of immigrants and refugees (may choose more than one).

- ☐ Tropical and other conditions not frequently diagnosed in US-born patients
- ☐ Learning about other cultures
- ☐ They don't complain as much
- ☐ Being able to hear their stories
- ☐ Their care is more complicated
- ☐ Their care is less complicated
- ☐ They are very appreciative of your help.
- ☐ They are extremely vulnerable
- ☐ Other:

c. Taking care of immigrants and refugees is more challenging than taking care of US born patients.

- ◯ Never
- ◯ Rarely
- ⊗ Sometimes
- ◯ Usually
- ◯ Always

d. Please mark all the challenges that you face as a provider when providing care to immigrants and refugees (may choose more than one):

- ☒ Language barriers
- ☒ Insurance barriers
- ☒ Cultural barriers
- ☐ Finding a professional interpreter
- ☐ Knowing how to work with a professional interpreter
- ☐ Time constraints
- ☒ My own knowledge related to tropical and travel medicine
- ☐ Transportation problems for the patient
- ☒ Patients not understanding treatment plan
- ☒ Patients not following treatment plan
- ☒ My lack of knowledge regarding the patient's culture
- ☒ Bias or stereotyping
- ☐ Other:

e. Please mark all of the challenges faced by immigrant and refugee populations when receiving healthcare that you have perceived or witnessed (may choose more than one):

- ☒ Language barriers
- ☒ Insurance barriers
- ☒ Cultural barriers
- ☒ Finding a professional interpreter
- ☒ Knowing how to work with a professional interpreter
- ☒ Time constraints
- ☒ Insufficiently trained health care providers
- ☒ Transportation problems for the patient
- ☒ Food insufficiency
- ☐ Need for child care
- ☒ Patients not understanding treatment plan
- ☒ Patients not following treatment plan
- ☒ My lack of knowledge regarding the patient's culture
- ☒ Bias or stereotyping
- ☒ Trust issues
- ☐ Other...

f. Rank how well immigrants and refugees understand the healthcare that you are trying to provide.

- ◯ Significantly less than a US born individual
- ⊗ Less than a US born individual
- ◯ Equivalent to a US born individual
- ◯ More than a US born patient
- ◯ Significantly more than a US born individual

g. Immigrants and refugees adhere to treatment plans and follow my recommendations.

- ◯ Never
- ◯ Rarely
- ◯ Sometimes
- ⊗ Usually
- ◯ Always

h. Immigrants and refugees should receive the same care and insurance coverage as US born patients.

- ◯ Never
- ◯ Rarely
- ◯ Sometimes
- ◯ Usually
- ⊗ Always

i. Immigrants and refugees who are undocumented should receive the same care and insurance coverage as US born patients.

- ◯ Never
- ◯ Rarely
- ◯ Sometimes
- ◯ Usually
- ⊗ Always

j. Every physician is professionally obligated to care for immigrants and refugees if they present to your clinic or hospital.

- ◯ Strongly disagree
- ◯ Disagree
- ◯ No opinion
- ◯ Agree
- ⊗ Strongly agree

k. Is healthcare a human right?

- ⊗ Yes
- ◯ No

B. If you wish, please tell us about what you enjoy or do not enjoy about immigrant and refugee health care and the greatest challenges you face in caring for this population.

I don't have a specific interest in caring for this population. I do feel that we have lots of great oppurtunities to learn about this often specialized care but often have the option of how in depth we want to take this learning.

SECTION D: DEMOGRAPHIC INFORMATION
Please answer the following questions by checking the box in front of the response choice that best describes you.

a. Your age?

- ◯ 20 to 24
- ⊗ 25 to 29
- ◯ 30 to 34
- ◯ 35 to 39
- ◯ 40 or older

b. Your gender?

- ⊗ Female
- ◯ Male
- ◯ Other

c. Are you Hispanic or Latino?

- ◯ Yes
- ⊗ No

d. What is your race? (Select one or more responses)

- ◯ American Indian or Alaska Native
- ◯ Asian (Please specify):
- ◯ Black or African American
- ◯ Native Hawaiian or Other Pacific Islander
- ⊗ White
- ◯ Other (Please specify):

e. Were you born in the United States?

- ⊗ Yes
- ◯ No

g. Your residency year?

- ◯ PGY1
- ◯ PGY2
- ⊗ PGY3
- ◯ PGY4
- ◯ PGY5

h. How would you classify your political ideology?

- ◯ Conservative
- ◯ Somewhat conservative
- ◯ Moderate
- ⊗ Somewhat liberal
- ◯ Liberal
- ◯ Other (Please specify):

i. Estimated level of educational debt?

- ◯ None
- ◯ Less than $50,000
- ◯ $50,000 - $100,000
- ◯ $100,000 - $200,000
- ⊗ $200,000 or more

j. Do you plan to subspecialize?

- ◯ Yes
- ⊗ No

k. Languages spoken?

- ☒ English
- ☐ Spanish
- ☐ French
- ☐ Hmong
- ☐ Somali
- ☐ Japanese
- ☐ Chinese
- ☐ Russian
- ☐ Ethiopian
- ☐ Other ___________

l. Are you in the Global Health Pathway?

- ◯ Yes
- ⊗ No

m.	Did you earn your degree in the US?

- ⊗ Yes
- ◯ No

n. What residency program are you in?

- ◯ Internal Medicine
- ◯ Med-Peds
- ⊗ Pediatrics
- ◯ Family Practice
- ◯ Neurology
- ◯ Psychiatry
- ◯ ObGyn
- ◯ Neurosurgery
- ◯ General Surgery
- ◯ Orthopedic Surgery
- ◯ Urology
- ◯ Surgical sub-specialty (please specify in text box below)
- ◯ Non-clinical specialty (radiology, pathology; please specify in text box below)

Medical Trainees' attitudes, knowledge, and experience with immigrant and refugee health

Response was added on 11/03/2013 8:41pm.

SECTION A: Personal experience with immigrant and refugee health care.

A. Please indicate your level of agreement with the following statements regarding your personal experience with immigrant and refugee health care by checking the box that best represents your experience.

a. During my inpatient rotations, I take care of the following percentage of immigrant and refugee patients:	◯ None<br>◯ 0 -5%<br>⊗ 10-25%<br>◯ > 25%
b. During my outpatient rotations, I take care of the following percentage of immigrant and refugee patients:	◯ None<br>◯ 0-10%<br>⊗ 10 -25%<br>◯ 25-50%<br>◯ 50-75%<br>◯ >75%
c. I would like to take care of more immigrant and refugee patients.	◯ Strongly disagree<br>◯ Disagree<br>⊗ No opinion<br>◯ Agree<br>◯ Strongly agree
d. I plan to take care of immigrants and refugees when I finish residency.	◯ Strongly disagree<br>◯ Disagree<br>◯ No opinion<br>⊗ Agree<br>◯ Strongly agree
e. I plan to do short term (< 6 months) international work when I finish residency.	◯ Strongly disagree<br>⊗ Disagree<br>◯ No opinion<br>◯ Agree<br>◯ Strongly agree
f. I plan to do long term (>6 months) international work when I finish residency.	◯ Strongly disagree<br>⊗ Disagree<br>◯ No opinion<br>◯ Agree<br>◯ Strongly agree
g. I plan to work in health disparities in the following way after residency:	Yes

SECTION B: MEDICAL EDUCATION

A. Please indicate your level of agreement with the following statements regarding your medical education and knowledge about immigrants and refugees by checking the box that best represents your opinion.

a. I have received specialized training in immigrant and refugee health, tropical medicine, or cross-cultural health.

- ◯ Strongly disagree
- ◯ Disagree
- ◯ No opinion
- ⊗ Agree
- ◯ Strongly agree

b. If you have received specialized training in immigrant and refugee health, tropical medicine, or cross-cultural health, please indicate all the contexts in which you received this training:

- ☐ As an undergraduate.
- ☒ As a medical student.
- ☒ As part of my residency.
- ☐ A special program.
- ☐ As part of my fellowship.
- ☐ As part of a degree program (e.g. MPH)
- ☐ Other:

c. I feel comfortable with my fund of knowledge regarding immigrant and refugee health.

- ◯ Strongly disagree
- ◯ Disagree
- ◯ No opinion
- ◯ Agree
- ◯ Strongly agree

d. I would like to have further training in immigrant and refugee health.

- ◯ Strongly disagree
- ◯ Disagree
- ◯ No opinion
- ⊗ Agree
- ◯ Strongly agree

e. If you agree with the above, please indicate all the contexts in which you would like to receive this training:
□□

- ☒ As part of my residency.
- ☒ A special program.
- ☒ As part of my fellowship.

SECTION C: Attitudes towards immigrant health

A.□Please indicate your level of agreement with the following statements regarding immigrant and refugee health by checking the box that best represents your opinion.

a. I enjoy taking care of immigrants and refugees.

- ◯ Never
- ◯ Rarely
- ◯ Sometimes
- ⊗ Usually
- ◯ Always

b. Please indicate the reasons that you enjoy taking care of immigrants and refugees (may choose more than one).

- ☒ Tropical and other conditions not frequently diagnosed in US-born patients
- ☐ Learning about other cultures
- ☐ They don't complain as much
- ☒ Being able to hear their stories
- ☐ Their care is more complicated
- ☐ Their care is less complicated
- ☒ They are very appreciative of your help.
- ☒ They are extremely vulnerable
- ☐ Other:

c. Taking care of immigrants and refugees is more challenging than taking care of US born patients.

- ◯ Never
- ◯ Rarely
- ⊗ Sometimes
- ◯ Usually
- ◯ Always

d. Please mark all the challenges that you face as a provider when providing care to immigrants and refugees (may choose more than one):

- ☒ Language barriers
- ☒ Insurance barriers
- ☐ Cultural barriers
- ☐ Finding a professional interpreter
- ☐ Knowing how to work with a professional interpreter
- ☐ Time constraints
- ☒ My own knowledge related to tropical and travel medicine
- ☐ Transportation problems for the patient
- ☐ Patients not understanding treatment plan
- ☐ Patients not following treatment plan
- ☐ My lack of knowledge regarding the patient's culture
- ☐ Bias or stereotyping
- ☐ Other:

e. Please mark all of the challenges faced by immigrant and refugee populations when receiving healthcare that you have perceived or witnessed (may choose more than one):

- ☒ Language barriers
- ☒ Insurance barriers
- ☒ Cultural barriers
- ☐ Finding a professional interpreter
- ☐ Knowing how to work with a professional interpreter
- ☐ Time constraints
- ☒ Insufficiently trained health care providers
- ☒ Transportation problems for the patient
- ☒ Food insufficiency
- ☐ Need for child care
- ☐ Patients not understanding treatment plan
- ☐ Patients not following treatment plan
- ☒ My lack of knowledge regarding the patient's culture
- ☒ Bias or stereotyping
- ☐ Trust issues
- ☐ Other...

f. Rank how well immigrants and refugees understand the healthcare that you are trying to provide.

- ○ Significantly less than a US born individual
- ⊗ Less than a US born individual
- ○ Equivalent to a US born individual
- ○ More than a US born patient
- ○ Significantly more than a US born individual

g. Immigrants and refugees adhere to treatment plans and follow my recommendations.

- ○ Never
- ○ Rarely
- ○ Sometimes
- ⊗ Usually
- ○ Always

h. Immigrants and refugees should receive the same care and insurance coverage as US born patients.

- ○ Never
- ○ Rarely
- ○ Sometimes
- ○ Usually
- ⊗ Always

i. Immigrants and refugees who are undocumented should receive the same care and insurance coverage as US born patients.

- ○ Never
- ○ Rarely
- ○ Sometimes
- ○ Usually
- ⊗ Always

j. Every physician is professionally obligated to care for immigrants and refugees if they present to your clinic or hospital.

- ○ Strongly disagree
- ○ Disagree
- ○ No opinion
- ○ Agree
- ⊗ Strongly agree

k. Is healthcare a human right?

- ⊗ Yes
- ◯ No

B. If you wish, please tell us about what you enjoy or do not enjoy about immigrant and refugee health care and the greatest challenges you face in caring for this population.

____

SECTION D: DEMOGRAPHIC INFORMATION
Please answer the following questions by checking the box in front of the response choice that best describes you.

a. Your age?

- ◯ 20 to 24
- ⊗ 25 to 29
- ◯ 30 to 34
- ◯ 35 to 39
- ◯ 40 or older

b. Your gender?

- ◯ Female
- ⊗ Male
- ◯ Other

c.Are you Hispanic or Latino?

- ◯ Yes
- ⊗ No

d. What is your race? (Select one or more responses)

- ◯ American Indian or Alaska Native
- ◯ Asian (Please specify):
- ◯ Black or African American
- ◯ Native Hawaiian or Other Pacific Islander
- ⊗ White
- ◯ Other (Please specify):

e.Were you born in the United States?

- ⊗ Yes
- ◯ No

g. Your residency year?

- ⊗ PGY1
- ◯ PGY2
- ◯ PGY3
- ◯ PGY4
- ◯ PGY5

h. How would you classify your political ideology?

- ◯ Conservative
- ◯ Somewhat conservative
- ◯ Moderate
- ◯ Somewhat liberal
- ⊗ Liberal
- ◯ Other (Please specify):

i. Estimated level of educational debt?

- ◯ None
- ◯ Less than $50,000
- ◯ $50,000 - $100,000
- ◯ $100,000 - $200,000
- ⊗ $200,000 or more

j.Do you plan to subspecialize?

- ⊗ Yes
- ◯ No

k. Languages spoken?

- ☒ English
- ☐ Spanish
- ☐ French
- ☐ Hmong
- ☐ Somali
- ☐ Japanese
- ☐ Chinese
- ☐ Russian
- ☐ Ethiopian
- ☐ Other ___________

l. Are you in the Global Health Pathway?

- ◯ Yes
- ⊗ No

m. Did you earn your degree in the US?

- ⊗ Yes
- ◯ No

n. What residency program are you in?

- ◯ Internal Medicine
- ◯ Med-Peds
- ◯ Pediatrics
- ◯ Family Practice
- ◯ Neurology
- ⊗ Psychiatry
- ◯ ObGyn
- ◯ Neurosurgery
- ◯ General Surgery
- ◯ Orthopedic Surgery
- ◯ Urology
- ◯ Surgical sub-specialty (please specify in text box below)
- ◯ Non-clinical specialty (radiology, pathology; please specify in text box below)

Medical Trainees' attitudes, knowledge, and experience with immigrant and refugee health

Response was added on 11/03/2013 8:50pm.

SECTION A: Personal experience with immigrant and refugee health care.

A. Please indicate your level of agreement with the following statements regarding your personal experience with immigrant and refugee health care by checking the box that best represents your experience.

a. During my inpatient rotations, I take care of the following percentage of immigrant and refugee patients:

- ◯ None
- ◯ 0 -5%
- ◯ 5-10%
- ⊗ 10-25%
- ◯ > 25%

b. During my outpatient rotations, I take care of the following percentage of immigrant and refugee patients:

- ◯ None
- ⊗ 0-10%
- ◯ 10 -25%
- ◯ 25-50%
- ◯ 50-75%
- ◯ >75%

c. I would like to take care of more immigrant and refugee patients.

- ◯ Strongly disagree
- ◯ Disagree
- ⊗ No opinion
- ◯ Agree
- ◯ Strongly agree

d. I plan to take care of immigrants and refugees when I finish residency.

- ◯ Strongly disagree
- ◯ Disagree
- ⊗ No opinion
- ◯ Agree
- ◯ Strongly agree

e. I plan to do short term (< 6 months) international work when I finish residency.

- ◯ Strongly disagree
- ⊗ Disagree
- ◯ No opinion
- ◯ Agree
- ◯ Strongly agree

f. I plan to do long term (>6 months) international work when I finish residency.

- ◯ Strongly disagree
- ⊗ Disagree
- ◯ No opinion
- ◯ Agree
- ◯ Strongly agree

g. I plan to work in health disparities in the following way after residency:

Inaptient - urban area

SECTION B: MEDICAL EDUCATION

A. Please indicate your level of agreement with the following statements regarding your medical education and knowledge about immigrants and refugees by checking the box that best represents your opinion.

a. I have received specialized training in immigrant and refugee health, tropical medicine, or cross-cultural health.

- ◯ Strongly disagree
- ◯ Disagree
- ◯ No opinion
- ⊗ Agree
- ◯ Strongly agree

b. If you have received specialized training in immigrant and refugee health, tropical medicine, or cross-cultural health, please indicate all the contexts in which you received this training:

- ☐ As an undergraduate.
- ☐ As a medical student.
- ☒ As part of my residency.
- ☐ A special program.
- ☐ As part of my fellowship.
- ☐ As part of a degree program (e.g. MPH)
- ☐ Other:

c. I feel comfortable with my fund of knowledge regarding immigrant and refugee health.

- ◯ Strongly disagree
- ◯ Disagree
- ◯ No opinion
- ⊗ Agree
- ◯ Strongly agree

d. I would like to have further training in immigrant and refugee health.

- ◯ Strongly disagree
- ◯ Disagree
- ◯ No opinion
- ⊗ Agree
- ◯ Strongly agree

e. If you agree with the above, please indicate all the contexts in which you would like to receive this training:
□□

- ☒ As part of my residency.
- ☐ A special program.
- ☐ As part of my fellowship.

SECTION C: Attitudes towards immigrant health

A.□Please indicate your level of agreement with the following statements regarding immigrant and refugee health by checking the box that best represents your opinion.

a. I enjoy taking care of immigrants and refugees.

- ◯ Never
- ◯ Rarely
- ⊗ Sometimes
- ◯ Usually
- ◯ Always

b. Please indicate the reasons that you enjoy taking care of immigrants and refugees (may choose more than one).

- ☐ Tropical and other conditions not frequently diagnosed in US-born patients
- ☒ Learning about other cultures
- ☐ They don't complain as much
- ☐ Being able to hear their stories
- ☐ Their care is more complicated
- ☐ Their care is less complicated
- ☐ They are very appreciative of your help.
- ☐ They are extremely vulnerable
- ☐ Other:

c. Taking care of immigrants and refugees is more challenging than taking care of US born patients.

- ◯ Never
- ◯ Rarely
- ◯ Sometimes
- ◯ Usually
- ⊗ Always

d. Please mark all the challenges that you face as a provider when providing care to immigrants and refugees (may choose more than one):

- ☒ Language barriers
- ☒ Insurance barriers
- ☒ Cultural barriers
- ☐ Finding a professional interpreter
- ☐ Knowing how to work with a professional interpreter
- ☐ Time constraints
- ☐ My own knowledge related to tropical and travel medicine
- ☐ Transportation problems for the patient
- ☒ Patients not understanding treatment plan
- ☒ Patients not following treatment plan
- ☐ My lack of knowledge regarding the patient's culture
- ☒ Bias or stereotyping
- ☐ Other:

e. Please mark all of the challenges faced by immigrant and refugee populations when receiving healthcare that you have perceived or witnessed (may choose more than one):

- ☒ Language barriers
- ☒ Insurance barriers
- ☒ Cultural barriers
- ☐ Finding a professional interpreter
- ☐ Knowing how to work with a professional interpreter
- ☐ Time constraints
- ☐ Insufficiently trained health care providers
- ☐ Transportation problems for the patient
- ☐ Food insufficiency
- ☐ Need for child care
- ☒ Patients not understanding treatment plan
- ☒ Patients not following treatment plan
- ☐ My lack of knowledge regarding the patient's culture
- ☐ Bias or stereotyping
- ☒ Trust issues
- ☐ Other...

f. Rank how well immigrants and refugees understand the healthcare that you are trying to provide.

- ⊗ Significantly less than a US born individual
- ○ Less than a US born individual
- ○ Equivalent to a US born individual
- ○ More than a US born patient
- ○ Significantly more than a US born individual

g. Immigrants and refugees adhere to treatment plans and follow my recommendations.

- ○ Never
- ⊗ Rarely
- ○ Sometimes
- ○ Usually
- ○ Always

h. Immigrants and refugees should receive the same care and insurance coverage as US born patients.

- ○ Never
- ○ Rarely
- ○ Sometimes
- ○ Usually
- ⊗ Always

i. Immigrants and refugees who are undocumented should receive the same care and insurance coverage as US born patients.

- ○ Never
- ⊗ Rarely
- ○ Sometimes
- ○ Usually
- ○ Always

j. Every physician is professionally obligated to care for immigrants and refugees if they present to your clinic or hospital.

- ○ Strongly disagree
- ○ Disagree
- ○ No opinion
- ○ Agree
- ⊗ Strongly agree

k. Is healthcare a human right?

⊗ Yes
◯ No

B. If you wish, please tell us about what you enjoy or do not enjoy about immigrant and refugee health care and the greatest challenges you face in caring for this population.

____

SECTION D: DEMOGRAPHIC INFORMATION
Please answer the following questions by checking the box in front of the response choice that best describes you.

a. Your age?

◯ 20 to 24
◯ 25 to 29
⊗ 30 to 34
◯ 35 to 39
◯ 40 or older

b. Your gender?

⊗ Female
◯ Male
◯ Other

c.Are you Hispanic or Latino?

◯ Yes
⊗ No

d. What is your race? (Select one or more responses)

◯ American Indian or Alaska Native
◯ Asian (Please specify):
◯ Black or African American
◯ Native Hawaiian or Other Pacific Islander
⊗ White
◯ Other (Please specify):

e.Were you born in the United States?

◯ Yes
⊗ No

f.If not, in what country were you born?

Romania - Eastern Europe

g. Your residency year?

◯ PGY1
⊗ PGY2
◯ PGY3
◯ PGY4
◯ PGY5

h. How would you classify your political ideology?

⊗ Conservative
◯ Somewhat conservative
◯ Moderate
◯ Somewhat liberal
◯ Liberal
◯ Other (Please specify):

i. Estimated level of educational debt?

⊗ None
◯ Less than $50,000
◯ $50,000 - $100,000
◯ $100,000 - $200,000
◯ $200,000 or more

j.Do you plan to subspecialize?

⊗ Yes
◯ No

k. Languages spoken?

- ☒ English
- ☒ Spanish
- ☒ French
- ☐ Hmong
- ☐ Somali
- ☐ Japanese
- ☐ Chinese
- ☐ Russian
- ☐ Ethiopian
- ☒ Other ___________

romanian, italian

l. Are you in the Global Health Pathway?

- ◯ Yes
- ⊗ No

m. Did you earn your degree in the US?

- ◯ Yes
- ⊗ No

n. What residency program are you in?

- ⊗ Internal Medicine
- ◯ Med-Peds
- ◯ Pediatrics
- ◯ Family Practice
- ◯ Neurology
- ◯ Psychiatry
- ◯ ObGyn
- ◯ Neurosurgery
- ◯ General Surgery
- ◯ Orthopedic Surgery
- ◯ Urology
- ◯ Surgical sub-specialty (please specify in text box below)
- ◯ Non-clinical specialty (radiology, pathology; please specify in text box below)

Medical Trainees' attitudes, knowledge, and experience with immigrant and refugee health

Response was added on 11/03/2013 9:59pm.

SECTION A: Personal experience with immigrant and refugee health care.

A. Please indicate your level of agreement with the following statements regarding your personal experience with immigrant and refugee health care by checking the box that best represents your experience.

a. During my inpatient rotations, I take care of the following percentage of immigrant and refugee patients:
- ◯ None
- ◯ 0 -5%
- ◯ 5-10%
- ◯ 10-25%
- ⊗ > 25%

b. During my outpatient rotations, I take care of the following percentage of immigrant and refugee patients:
- ◯ None
- ◯ 0-10%
- ◯ 10 -25%
- ⊗ 25-50%
- ◯ 50-75%
- ◯ >75%

c. I would like to take care of more immigrant and refugee patients.
- ◯ Strongly disagree
- ⊗ Disagree
- ◯ No opinion
- ◯ Agree
- ◯ Strongly agree

d. I plan to take care of immigrants and refugees when I finish residency.
- ◯ Strongly disagree
- ◯ Disagree
- ⊗ No opinion
- ◯ Agree
- ◯ Strongly agree

e. I plan to do short term (< 6 months) international work when I finish residency.
- ◯ Strongly disagree
- ⊗ Disagree
- ◯ No opinion
- ◯ Agree
- ◯ Strongly agree

f. I plan to do long term (>6 months) international work when I finish residency.
- ◯ Strongly disagree
- ⊗ Disagree
- ◯ No opinion
- ◯ Agree
- ◯ Strongly agree

g. I plan to work in health disparities in the following way after residency: Nope

SECTION B: MEDICAL EDUCATION

A. Please indicate your level of agreement with the following statements regarding your medical education and knowledge about immigrants and refugees by checking the box that best represents your opinion.

a. I have received specialized training in immigrant and refugee health, tropical medicine, or cross-cultural health.

- ◯ Strongly disagree
- ⊗ Disagree
- ◯ No opinion
- ◯ Agree
- ◯ Strongly agree

c. I feel comfortable with my fund of knowledge regarding immigrant and refugee health.

- ◯ Strongly disagree
- ◯ Disagree
- ◯ No opinion
- ⊗ Agree
- ◯ Strongly agree

d. I would like to have further training in immigrant and refugee health.

- ◯ Strongly disagree
- ⊗ Disagree
- ◯ No opinion
- ◯ Agree
- ◯ Strongly agree

SECTION C: Attitudes towards immigrant health

A.Please indicate your level of agreement with the following statements regarding immigrant and refugee health by checking the box that best represents your opinion.

a. I enjoy taking care of immigrants and refugees.

- ◯ Never
- ◯ Rarely
- ⊗ Sometimes
- ◯ Usually
- ◯ Always

b. Please indicate the reasons that you enjoy taking care of immigrants and refugees (may choose more than one).

- ☒ Tropical and other conditions not frequently diagnosed in US-born patients
- ☐ Learning about other cultures
- ☐ They don't complain as much
- ☐ Being able to hear their stories
- ☐ Their care is more complicated
- ☐ Their care is less complicated
- ☐ They are very appreciative of your help.
- ☐ They are extremely vulnerable
- ☐ Other:

c. Taking care of immigrants and refugees is more challenging than taking care of US born patients.

- ◯ Never
- ◯ Rarely
- ◯ Sometimes
- ⊗ Usually
- ◯ Always

d. Please mark all the challenges that you face as a provider when providing care to immigrants and refugees (may choose more than one):

- ☒ Language barriers
- ☐ Insurance barriers
- ☒ Cultural barriers
- ☒ Finding a professional interpreter
- ☐ Knowing how to work with a professional interpreter
- ☒ Time constraints
- ☐ My own knowledge related to tropical and travel medicine
- ☒ Transportation problems for the patient
- ☒ Patients not understanding treatment plan
- ☒ Patients not following treatment plan
- ☐ My lack of knowledge regarding the patient's culture
- ☐ Bias or stereotyping
- ☐ Other:

e. Please mark all of the challenges faced by immigrant and refugee populations when receiving healthcare that you have perceived or witnessed (may choose more than one):

- ☒ Language barriers
- ☐ Insurance barriers
- ☐ Cultural barriers
- ☐ Finding a professional interpreter
- ☐ Knowing how to work with a professional interpreter
- ☐ Time constraints
- ☐ Insufficiently trained health care providers
- ☐ Transportation problems for the patient
- ☐ Food insufficiency
- ☐ Need for child care
- ☐ Patients not understanding treatment plan
- ☐ Patients not following treatment plan
- ☐ My lack of knowledge regarding the patient's culture
- ☐ Bias or stereotyping
- ☐ Trust issues
- ☐ Other...

f. Rank how well immigrants and refugees understand the healthcare that you are trying to provide.

- ○ Significantly less than a US born individual
- ⊗ Less than a US born individual
- ○ Equivalent to a US born individual
- ○ More than a US born patient
- ○ Significantly more than a US born individual

g. Immigrants and refugees adhere to treatment plans and follow my recommendations.

- ○ Never
- ○ Rarely
- ⊗ Sometimes
- ○ Usually
- ○ Always

h. Immigrants and refugees should receive the same care and insurance coverage as US born patients.

- ○ Never
- ○ Rarely
- ⊗ Sometimes
- ○ Usually
- ○ Always

i. Immigrants and refugees who are undocumented should receive the same care and insurance coverage as US born patients.

- ○ Never
- ⊗ Rarely
- ○ Sometimes
- ○ Usually
- ○ Always

j. Every physician is professionally obligated to care for immigrants and refugees if they present to your clinic or hospital.

- ○ Strongly disagree
- ○ Disagree
- ○ No opinion
- ⊗ Agree
- ○ Strongly agree

k. Is healthcare a human right?

- ○ Yes
- ⊗ No

B. If you wish, please tell us about what you enjoy or do not enjoy about immigrant and refugee health care and the greatest challenges you face in caring for this population.

____

SECTION D: DEMOGRAPHIC INFORMATION
Please answer the following questions by checking the box in front of the response choice that best describes you.

a. Your age?
- ◯ 20 to 24
- ◯ 25 to 29
- ⊗ 30 to 34
- ◯ 35 to 39
- ◯ 40 or older

b. Your gender?
- ◯ Female
- ⊗ Male
- ◯ Other

c. Are you Hispanic or Latino?
- ◯ Yes
- ⊗ No

d. What is your race? (Select one or more responses)
- ◯ American Indian or Alaska Native
- ◯ Asian (Please specify):
- ◯ Black or African American
- ◯ Native Hawaiian or Other Pacific Islander
- ⊗ White
- ◯ Other (Please specify):

e. Were you born in the United States?
- ⊗ Yes
- ◯ No

g. Your residency year?
- ◯ PGY1
- ◯ PGY2
- ⊗ PGY3
- ◯ PGY4
- ◯ PGY5

h. How would you classify your political ideology?
- ◯ Conservative
- ◯ Somewhat conservative
- ⊗ Moderate
- ◯ Somewhat liberal
- ◯ Liberal
- ◯ Other (Please specify):

i. Estimated level of educational debt?
- ⊗ None
- ◯ Less than $50,000
- ◯ $50,000 - $100,000
- ◯ $100,000 - $200,000
- ◯ $200,000 or more

j. Do you plan to subspecialize?
- ⊗ Yes
- ◯ No

k. Languages spoken?
- ☒ English
- ☐ Spanish
- ☒ French
- ☐ Hmong
- ☐ Somali
- ☐ Japanese
- ☐ Chinese
- ☐ Russian
- ☐ Ethiopian
- ☐ Other ___________

l. Are you in the Global Health Pathway?
- ◯ Yes
- ⊗ No

m. Did you earn your degree in the US?

- ⊗ Yes
- ◯ No

n. What residency program are you in?

- ◯ Internal Medicine
- ◯ Med-Peds
- ◯ Pediatrics
- ◯ Family Practice
- ◯ Neurology
- ◯ Psychiatry
- ◯ ObGyn
- ◯ Neurosurgery
- ◯ General Surgery
- ◯ Orthopedic Surgery
- ◯ Urology
- ◯ Surgical sub-specialty (please specify in text box below)
- ⊗ Non-clinical specialty (radiology, pathology; please specify in text box below)

____

Medical Trainees' attitudes, knowledge, and experience with immigrant and refugee health

Response was added on 11/03/2013 10:49pm.

SECTION A: Personal experience with immigrant and refugee health care.

A. Please indicate your level of agreement with the following statements regarding your personal experience with immigrant and refugee health care by checking the box that best represents your experience.

a. During my inpatient rotations, I take care of the following percentage of immigrant and refugee patients:

- ◯ None
- ◯ 0 -5%
- ⊗ 5-10%
- ◯ 10-25%
- ◯ > 25%

b. During my outpatient rotations, I take care of the following percentage of immigrant and refugee patients:

- ⊗ None
- ◯ 0-10%
- ◯ 10 -25%
- ◯ 25-50%
- ◯ 50-75%
- ◯ >75%

c. I would like to take care of more immigrant and refugee patients.

- ◯ Strongly disagree
- ◯ Disagree
- ◯ No opinion
- ⊗ Agree
- ◯ Strongly agree

d. I plan to take care of immigrants and refugees when I finish residency.

- ◯ Strongly disagree
- ◯ Disagree
- ◯ No opinion
- ⊗ Agree
- ◯ Strongly agree

e. I plan to do short term (< 6 months) international work when I finish residency.

- ◯ Strongly disagree
- ◯ Disagree
- ◯ No opinion
- ◯ Agree
- ⊗ Strongly agree

f. I plan to do long term (>6 months) international work when I finish residency.

- ◯ Strongly disagree
- ◯ Disagree
- ◯ No opinion
- ⊗ Agree
- ◯ Strongly agree

g. I plan to work in health disparities in the following way after residency:

a

SECTION B: MEDICAL EDUCATION

A. Please indicate your level of agreement with the following statements regarding your medical education and knowledge about immigrants and refugees by checking the box that best represents your opinion.

a. I have received specialized training in immigrant and refugee health, tropical medicine, or cross-cultural health.

- ◯ Strongly disagree
- ⊗ Disagree
- ◯ No opinion
- ◯ Agree
- ◯ Strongly agree

c. I feel comfortable with my fund of knowledge regarding immigrant and refugee health.

- ◯ Strongly disagree
- ◯ Disagree
- ◯ No opinion
- ⊗ Agree
- ◯ Strongly agree

d. I would like to have further training in immigrant and refugee health.

- ◯ Strongly disagree
- ◯ Disagree
- ⊗ No opinion
- ◯ Agree
- ◯ Strongly agree

SECTION C: Attitudes towards immigrant health

A.Please indicate your level of agreement with the following statements regarding immigrant and refugee health by checking the box that best represents your opinion.

a. I enjoy taking care of immigrants and refugees.

- ◯ Never
- ◯ Rarely
- ◯ Sometimes
- ⊗ Usually
- ◯ Always

b. Please indicate the reasons that you enjoy taking care of immigrants and refugees (may choose more than one).

- ☐ Tropical and other conditions not frequently diagnosed in US-born patients
- ☒ Learning about other cultures
- ☒ They don't complain as much
- ☐ Being able to hear their stories
- ☐ Their care is more complicated
- ☐ Their care is less complicated
- ☒ They are very appreciative of your help.
- ☒ They are extremely vulnerable
- ☐ Other:

c. Taking care of immigrants and refugees is more challenging than taking care of US born patients.

- ◯ Never
- ◯ Rarely
- ⊗ Sometimes
- ◯ Usually
- ◯ Always

d. Please mark all the challenges that you face as a provider when providing care to immigrants and refugees (may choose more than one):

- ☒ Language barriers
- ☐ Insurance barriers
- ☒ Cultural barriers
- ☐ Finding a professional interpreter
- ☐ Knowing how to work with a professional interpreter
- ☒ Time constraints
- ☐ My own knowledge related to tropical and travel medicine
- ☐ Transportation problems for the patient
- ☐ Patients not understanding treatment plan
- ☐ Patients not following treatment plan
- ☐ My lack of knowledge regarding the patient's culture
- ☐ Bias or stereotyping
- ☐ Other:

e. Please mark all of the challenges faced by immigrant and refugee populations when receiving healthcare that you have perceived or witnessed (may choose more than one):

- ☒ Language barriers
- ☒ Insurance barriers
- ☒ Cultural barriers
- ☐ Finding a professional interpreter
- ☐ Knowing how to work with a professional interpreter
- ☒ Time constraints
- ☐ Insufficiently trained health care providers
- ☒ Transportation problems for the patient
- ☐ Food insufficiency
- ☐ Need for child care
- ☒ Patients not understanding treatment plan
- ☐ Patients not following treatment plan
- ☐ My lack of knowledge regarding the patient's culture
- ☐ Bias or stereotyping
- ☐ Trust issues
- ☐ Other...

f. Rank how well immigrants and refugees understand the healthcare that you are trying to provide.

- ◯ Significantly less than a US born individual
- ⊗ Less than a US born individual
- ◯ Equivalent to a US born individual
- ◯ More than a US born patient
- ◯ Significantly more than a US born individual

g. Immigrants and refugees adhere to treatment plans and follow my recommendations.

- ◯ Never
- ◯ Rarely
- ⊗ Sometimes
- ◯ Usually
- ◯ Always

h. Immigrants and refugees should receive the same care and insurance coverage as US born patients.

- ◯ Never
- ◯ Rarely
- ◯ Sometimes
- ◯ Usually
- ⊗ Always

i. Immigrants and refugees who are undocumented should receive the same care and insurance coverage as US born patients.

- ◯ Never
- ◯ Rarely
- ◯ Sometimes
- ◯ Usually
- ⊗ Always

j. Every physician is professionally obligated to care for immigrants and refugees if they present to your clinic or hospital.

- ◯ Strongly disagree
- ◯ Disagree
- ◯ No opinion
- ◯ Agree
- ⊗ Strongly agree

k. Is healthcare a human right?

- ⊗ Yes
- ◯ No

B. If you wish, please tell us about what you enjoy or do not enjoy about immigrant and refugee health care and the greatest challenges you face in caring for this population.

____

SECTION D: DEMOGRAPHIC INFORMATION
Please answer the following questions by checking the box in front of the response choice that best describes you.

a. Your age?
- ◯ 20 to 24
- ⊗ 25 to 29
- ◯ 30 to 34
- ◯ 35 to 39
- ◯ 40 or older

b. Your gender?
- ◯ Female
- ⊗ Male
- ◯ Other

c. Are you Hispanic or Latino?
- ◯ Yes
- ⊗ No

d. What is your race? (Select one or more responses)
- ◯ American Indian or Alaska Native
- ◯ Asian (Please specify):
- ◯ Black or African American
- ◯ Native Hawaiian or Other Pacific Islander
- ⊗ White
- ◯ Other (Please specify):

e. Were you born in the United States?
- ⊗ Yes
- ◯ No

g. Your residency year?
- ◯ PGY1
- ◯ PGY2
- ⊗ PGY3
- ◯ PGY4
- ◯ PGY5

h. How would you classify your political ideology?
- ◯ Conservative
- ◯ Somewhat conservative
- ⊗ Moderate
- ◯ Somewhat liberal
- ◯ Liberal
- ◯ Other (Please specify):

i. Estimated level of educational debt?
- ◯ None
- ◯ Less than $50,000
- ◯ $50,000 - $100,000
- ⊗ $100,000 - $200,000
- ◯ $200,000 or more

j. Do you plan to subspecialize?
- ⊗ Yes
- ◯ No

k. Languages spoken?
- ☒ English
- ☒ Spanish
- ☐ French
- ☐ Hmong
- ☐ Somali
- ☐ Japanese
- ☐ Chinese
- ☐ Russian
- ☐ Ethiopian
- ☐ Other ___________

l. Are you in the Global Health Pathway?
- ◯ Yes
- ⊗ No

m. Did you earn your degree in the US?

- ⊗ Yes
- ◯ No

n. What residency program are you in?

- ◯ Internal Medicine
- ◯ Med-Peds
- ◯ Pediatrics
- ◯ Family Practice
- ◯ Neurology
- ◯ Psychiatry
- ◯ ObGyn
- ◯ Neurosurgery
- ⊗ General Surgery
- ◯ Orthopedic Surgery
- ◯ Urology
- ◯ Surgical sub-specialty (please specify in text box below)
- ◯ Non-clinical specialty (radiology, pathology; please specify in text box below)

Medical Trainees' attitudes, knowledge, and experience with immigrant and refugee health

Response was added on 11/04/2013 5:19am.

SECTION A: Personal experience with immigrant and refugee health care.

A. Please indicate your level of agreement with the following statements regarding your personal experience with immigrant and refugee health care by checking the box that best represents your experience.

a. During my inpatient rotations, I take care of the following percentage of immigrant and refugee patients:

- ○ None
- ○ 0 -5%
- ○ 5-10%
- ○ 10-25%
- ⊗ > 25%

b. During my outpatient rotations, I take care of the following percentage of immigrant and refugee patients:

- ○ None
- ○ 0-10%
- ○ 10 -25%
- ○ 25-50%
- ○ 50-75%
- ⊗ >75%

c. I would like to take care of more immigrant and refugee patients.

- ○ Strongly disagree
- ○ Disagree
- ○ No opinion
- ○ Agree
- ⊗ Strongly agree

d. I plan to take care of immigrants and refugees when I finish residency.

- ○ Strongly disagree
- ○ Disagree
- ○ No opinion
- ○ Agree
- ⊗ Strongly agree

e. I plan to do short term (< 6 months) international work when I finish residency.

- ○ Strongly disagree
- ○ Disagree
- ⊗ No opinion
- ○ Agree
- ○ Strongly agree

f. I plan to do long term (>6 months) international work when I finish residency.

- ○ Strongly disagree
- ○ Disagree
- ⊗ No opinion
- ○ Agree
- ○ Strongly agree

g. I plan to work in health disparities in the following way after residency:

May consider working at community health center.

SECTION B: MEDICAL EDUCATION

A. Please indicate your level of agreement with the following statements regarding your medical education and knowledge about immigrants and refugees by checking the box that best represents your opinion.

a. I have received specialized training in immigrant and refugee health, tropical medicine, or cross-cultural health.

- ◯ Strongly disagree
- ⊗ Disagree
- ⊗ No opinion
- ◯ Agree
- ◯ Strongly agree

c. I feel comfortable with my fund of knowledge regarding immigrant and refugee health.

- ◯ Strongly disagree
- ⊗ Disagree
- ◯ No opinion
- ◯ Agree
- ◯ Strongly agree

d. I would like to have further training in immigrant and refugee health.

- ◯ Strongly disagree
- ◯ Disagree
- ◯ No opinion
- ⊗ Agree
- ◯ Strongly agree

e. If you agree with the above, please indicate all the contexts in which you would like to receive this training:

- ☒ As part of my residency.
- ☒ A special program.
- ☐ As part of my fellowship.

SECTION C: Attitudes towards immigrant health

A. Please indicate your level of agreement with the following statements regarding immigrant and refugee health by checking the box that best represents your opinion.

a. I enjoy taking care of immigrants and refugees.

- ◯ Never
- ◯ Rarely
- ◯ Sometimes
- ◯ Usually
- ⊗ Always

b. Please indicate the reasons that you enjoy taking care of immigrants and refugees (may choose more than one).

- ☒ Tropical and other conditions not frequently diagnosed in US-born patients
- ☒ Learning about other cultures
- ☐ They don't complain as much
- ☐ Being able to hear their stories
- ☐ Their care is more complicated
- ☐ Their care is less complicated
- ☒ They are very appreciative of your help.
- ☒ They are extremely vulnerable
- ☐ Other:

c. Taking care of immigrants and refugees is more challenging than taking care of US born patients.

- ◯ Never
- ◯ Rarely
- ◯ Sometimes
- ⊗ Usually
- ◯ Always

d. Please mark all the challenges that you face as a provider when providing care to immigrants and refugees (may choose more than one):

- ☒ Language barriers
- ☒ Insurance barriers
- ☒ Cultural barriers
- ☐ Finding a professional interpreter
- ☐ Knowing how to work with a professional interpreter
- ☒ Time constraints
- ☒ My own knowledge related to tropical and travel medicine
- ☒ Transportation problems for the patient
- ☒ Patients not understanding treatment plan
- ☒ Patients not following treatment plan
- ☒ My lack of knowledge regarding the patient's culture
- ☒ Bias or stereotyping
- ☐ Other:

e. Please mark all of the challenges faced by immigrant and refugee populations when receiving healthcare that you have perceived or witnessed (may choose more than one):

- ☒ Language barriers
- ☒ Insurance barriers
- ☒ Cultural barriers
- ☒ Finding a professional interpreter
- ☒ Knowing how to work with a professional interpreter
- ☒ Time constraints
- ☒ Insufficiently trained health care providers
- ☒ Transportation problems for the patient
- ☒ Food insufficiency
- ☒ Need for child care
- ☒ Patients not understanding treatment plan
- ☒ Patients not following treatment plan
- ☒ My lack of knowledge regarding the patient's culture
- ☒ Bias or stereotyping
- ☒ Trust issues
- ☐ Other...

f. Rank how well immigrants and refugees understand the healthcare that you are trying to provide.

- ○ Significantly less than a US born individual
- ⊗ Less than a US born individual
- ○ Equivalent to a US born individual
- ○ More than a US born patient
- ○ Significantly more than a US born individual

g. Immigrants and refugees adhere to treatment plans and follow my recommendations.

- ○ Never
- ○ Rarely
- ⊗ Sometimes
- ○ Usually
- ○ Always

h. Immigrants and refugees should receive the same care and insurance coverage as US born patients.

- ○ Never
- ○ Rarely
- ○ Sometimes
- ○ Usually
- ⊗ Always

i. Immigrants and refugees who are undocumented should receive the same care and insurance coverage as US born patients.

- ○ Never
- ○ Rarely
- ○ Sometimes
- ○ Usually
- ⊗ Always

j. Every physician is professionally obligated to care for immigrants and refugees if they present to your clinic or hospital.

- ○ Strongly disagree
- ○ Disagree
- ⊗ No opinion
- ○ Agree
- ○ Strongly agree

k. Is healthcare a human right?

- ◯ Yes
- ⊗ No

B. If you wish, please tell us about what you enjoy or do not enjoy about immigrant and refugee health care and the greatest challenges you face in caring for this population.

____

SECTION D: DEMOGRAPHIC INFORMATION
Please answer the following questions by checking the box in front of the response choice that best describes you.

a. Your age?

- ◯ 20 to 24
- ◯ 25 to 29
- ◯ 30 to 34
- ⊗ 35 to 39
- ◯ 40 or older

b. Your gender?

- ⊗ Female
- ◯ Male
- ◯ Other

c. Are you Hispanic or Latino?

- ◯ Yes
- ⊗ No

d. What is your race? (Select one or more responses)

- ◯ American Indian or Alaska Native
- ◯ Asian (Please specify):
- ◯ Black or African American
- ◯ Native Hawaiian or Other Pacific Islander
- ⊗ White
- ◯ Other (Please specify):

e. Were you born in the United States?

- ⊗ Yes
- ◯ No

g. Your residency year?

- ◯ PGY1
- ◯ PGY2
- ⊗ PGY3
- ◯ PGY4
- ◯ PGY5

h. How would you classify your political ideology?

- ◯ Conservative
- ◯ Somewhat conservative
- ◯ Moderate
- ⊗ Somewhat liberal
- ◯ Liberal
- ◯ Other (Please specify):

i. Estimated level of educational debt?

- ◯ None
- ◯ Less than $50,000
- ◯ $50,000 - $100,000
- ◯ $100,000 - $200,000
- ⊗ $200,000 or more

j. Do you plan to subspecialize?

- ◯ Yes
- ⊗ No

k. Languages spoken?

- ☒ English
- ☒ Spanish
- ☐ French
- ☐ Hmong
- ☐ Somali
- ☐ Japanese
- ☐ Chinese
- ☐ Russian
- ☐ Ethiopian
- ☐ Other ___________

l. Are you in the Global Health Pathway?

- ○ Yes
- ⊗ No

m. Did you earn your degree in the US?

- ⊗ Yes
- ○ No

n. What residency program are you in?

- ○ Internal Medicine
- ○ Med-Peds
- ○ Pediatrics
- ⊗ Family Practice
- ○ Neurology
- ○ Psychiatry
- ○ ObGyn
- ○ Neurosurgery
- ○ General Surgery
- ○ Orthopedic Surgery
- ○ Urology
- ○ Surgical sub-specialty (please specify in text box below)
- ○ Non-clinical specialty (radiology, pathology; please specify in text box below)

Medical Trainees' attitudes, knowledge, and experience with immigrant and refugee health

Response was added on 11/04/2013 6:33am.

SECTION A: Personal experience with immigrant and refugee health care.

A. Please indicate your level of agreement with the following statements regarding your personal experience with immigrant and refugee health care by checking the box that best represents your experience.

a. During my inpatient rotations, I take care of the following percentage of immigrant and refugee patients:

- ⊗ None
- ◯ 0 -5%
- ◯ 5-10%
- ◯ 10-25%
- ◯ > 25%

b. During my outpatient rotations, I take care of the following percentage of immigrant and refugee patients:

- ◯ None
- ⊗ 0-10%
- ◯ 10 -25%
- ◯ 25-50%
- ◯ 50-75%
- ◯ >75%

c. I would like to take care of more immigrant and refugee patients.

- ◯ Strongly disagree
- ◯ Disagree
- ⊗ No opinion
- ◯ Agree
- ◯ Strongly agree

d. I plan to take care of immigrants and refugees when I finish residency.

- ◯ Strongly disagree
- ◯ Disagree
- ⊗ No opinion
- ◯ Agree
- ◯ Strongly agree

e. I plan to do short term (< 6 months) international work when I finish residency.

- ◯ Strongly disagree
- ⊗ Disagree
- ◯ No opinion
- ◯ Agree
- ◯ Strongly agree

f. I plan to do long term (>6 months) international work when I finish residency.

- ⊗ Strongly disagree
- ◯ Disagree
- ◯ No opinion
- ◯ Agree
- ◯ Strongly agree

g. I plan to work in health disparities in the following way after residency:

Care for all patients referred to me. Many will have disproportionately low socioeconomic status
.

SECTION B: MEDICAL EDUCATION

A. Please indicate your level of agreement with the following statements regarding your medical education and knowledge about immigrants and refugees by checking the box that best represents your opinion.

a. I have received specialized training in immigrant and refugee health, tropical medicine, or cross-cultural health.

- ◯ Strongly disagree
- ⊗ Disagree
- ◯ No opinion
- ◯ Agree
- ◯ Strongly agree

c. I feel comfortable with my fund of knowledge regarding immigrant and refugee health.

- ◯ Strongly disagree
- ⊗ Disagree
- ◯ No opinion
- ◯ Agree
- ◯ Strongly agree

d. I would like to have further training in immigrant and refugee health.

- ◯ Strongly disagree
- ◯ Disagree
- ◯ No opinion
- ⊗ Agree
- ◯ Strongly agree

e. If you agree with the above, please indicate all the contexts in which you would like to receive this training:
□□

- ☒ As part of my residency.
- ☐ A special program.
- ☐ As part of my fellowship.

SECTION C: Attitudes towards immigrant health

A.□Please indicate your level of agreement with the following statements regarding immigrant and refugee health by checking the box that best represents your opinion.

a. I enjoy taking care of immigrants and refugees.

- ◯ Never
- ◯ Rarely
- ◯ Sometimes
- ⊗ Usually
- ◯ Always

b. Please indicate the reasons that you enjoy taking care of immigrants and refugees (may choose more than one).

- ☐ Tropical and other conditions not frequently diagnosed in US-born patients
- ☒ Learning about other cultures
- ☐ They don't complain as much
- ☐ Being able to hear their stories
- ☐ Their care is more complicated
- ☐ Their care is less complicated
- ☐ They are very appreciative of your help.
- ☐ They are extremely vulnerable
- ☐ Other:

c. Taking care of immigrants and refugees is more challenging than taking care of US born patients.

- ◯ Never
- ◯ Rarely
- ◯ Sometimes
- ⊗ Usually
- ◯ Always

d. Please mark all the challenges that you face as a provider when providing care to immigrants and refugees (may choose more than one):

- ☒ Language barriers
- ☐ Insurance barriers
- ☒ Cultural barriers
- ☐ Finding a professional interpreter
- ☐ Knowing how to work with a professional interpreter
- ☒ Time constraints
- ☐ My own knowledge related to tropical and travel medicine
- ☐ Transportation problems for the patient
- ☐ Patients not understanding treatment plan
- ☐ Patients not following treatment plan
- ☒ My lack of knowledge regarding the patient's culture
- ☐ Bias or stereotyping
- ☐ Other:

e. Please mark all of the challenges faced by immigrant and refugee populations when receiving healthcare that you have perceived or witnessed (may choose more than one):

- ☒ Language barriers
- ☐ Insurance barriers
- ☒ Cultural barriers
- ☐ Finding a professional interpreter
- ☐ Knowing how to work with a professional interpreter
- ☒ Time constraints
- ☒ Insufficiently trained health care providers
- ☐ Transportation problems for the patient
- ☐ Food insufficiency
- ☐ Need for child care
- ☒ Patients not understanding treatment plan
- ☐ Patients not following treatment plan
- ☐ My lack of knowledge regarding the patient's culture
- ☐ Bias or stereotyping
- ☒ Trust issues
- ☐ Other...

f. Rank how well immigrants and refugees understand the healthcare that you are trying to provide.

- ○ Significantly less than a US born individual
- ⊗ Less than a US born individual
- ○ Equivalent to a US born individual
- ○ More than a US born patient
- ○ Significantly more than a US born individual

g. Immigrants and refugees adhere to treatment plans and follow my recommendations.

- ○ Never
- ○ Rarely
- ○ Sometimes
- ⊗ Usually
- ○ Always

h. Immigrants and refugees should receive the same care and insurance coverage as US born patients.

- ○ Never
- ○ Rarely
- ○ Sometimes
- ○ Usually
- ⊗ Always

i. Immigrants and refugees who are undocumented should receive the same care and insurance coverage as US born patients.

- ○ Never
- ○ Rarely
- ○ Sometimes
- ○ Usually
- ⊗ Always

j. Every physician is professionally obligated to care for immigrants and refugees if they present to your clinic or hospital.

- ○ Strongly disagree
- ○ Disagree
- ○ No opinion
- ○ Agree
- ⊗ Strongly agree

k. Is healthcare a human right?
⊗ Yes
◯ No

B. If you wish, please tell us about what you enjoy or do not enjoy about immigrant and refugee health care and the greatest challenges you face in caring for this population.
____

SECTION D: DEMOGRAPHIC INFORMATION
Please answer the following questions by checking the box in front of the response choice that best describes you.

a. Your age?
◯ 20 to 24
◯ 25 to 29
⊗ 30 to 34
◯ 35 to 39
◯ 40 or older

b. Your gender?
◯ Female
⊗ Male
◯ Other

c. Are you Hispanic or Latino?
◯ Yes
⊗ No

d. What is your race? (Select one or more responses)
◯ American Indian or Alaska Native
◯ Asian (Please specify):
◯ Black or African American
◯ Native Hawaiian or Other Pacific Islander
⊗ White
◯ Other (Please specify):

e. Were you born in the United States?
⊗ Yes
◯ No

g. Your residency year?
◯ PGY1
⊗ PGY2
◯ PGY3
◯ PGY4
◯ PGY5

h. How would you classify your political ideology?
◯ Conservative
◯ Somewhat conservative
◯ Moderate
◯ Somewhat liberal
⊗ Liberal
◯ Other (Please specify):

i. Estimated level of educational debt?
◯ None
◯ Less than $50,000
◯ $50,000 - $100,000
⊗ $100,000 - $200,000
◯ $200,000 or more

j. Do you plan to subspecialize?
⊗ Yes
◯ No

k. Languages spoken?

- [x] English
- [ ] Spanish
- [ ] French
- [ ] Hmong
- [ ] Somali
- [ ] Japanese
- [ ] Chinese
- [ ] Russian
- [ ] Ethiopian
- [ ] Other ___________

l. Are you in the Global Health Pathway?

- ◯ Yes
- ⊗ No

m. Did you earn your degree in the US?

- ⊗ Yes
- ◯ No

n. What residency program are you in?

- ◯ Internal Medicine
- ◯ Med-Peds
- ◯ Pediatrics
- ◯ Family Practice
- ◯ Neurology
- ◯ Psychiatry
- ◯ ObGyn
- ◯ Neurosurgery
- ◯ General Surgery
- ◯ Orthopedic Surgery
- ◯ Urology
- ⊗ Surgical sub-specialty (please specify in text box below)
- ◯ Non-clinical specialty (radiology, pathology; please specify in text box below)

radiation oncology

Medical Trainees' attitudes, knowledge, and experience with immigrant and refugee health

Response was added on 11/04/2013 6:45am.

SECTION A: Personal experience with immigrant and refugee health care.

A. Please indicate your level of agreement with the following statements regarding your personal experience with immigrant and refugee health care by checking the box that best represents your experience.

a. During my inpatient rotations, I take care of the following percentage of immigrant and refugee patients:
- ◯ None
- ◯ 0 -5%
- ◯ 5-10%
- ⊗ 10-25%
- ◯ > 25%

b. During my outpatient rotations, I take care of the following percentage of immigrant and refugee patients:
- ◯ None
- ◯ 0-10%
- ◯ 10 -25%
- ⊗ 25-50%
- ◯ 50-75%
- ◯ >75%

c. I would like to take care of more immigrant and refugee patients.
- ◯ Strongly disagree
- ◯ Disagree
- ◯ No opinion
- ⊗ Agree
- ◯ Strongly agree

d. I plan to take care of immigrants and refugees when I finish residency.
- ◯ Strongly disagree
- ◯ Disagree
- ◯ No opinion
- ⊗ Agree
- ◯ Strongly agree

e. I plan to do short term (< 6 months) international work when I finish residency.
- ◯ Strongly disagree
- ◯ Disagree
- ◯ No opinion
- ◯ Agree
- ⊗ Strongly agree

f. I plan to do long term (>6 months) international work when I finish residency.
- ◯ Strongly disagree
- ⊗ Disagree
- ◯ No opinion
- ◯ Agree
- ◯ Strongly agree

g. I plan to work in health disparities in the following way after residency:

underserved community

SECTION B: MEDICAL EDUCATION

A. Please indicate your level of agreement with the following statements regarding your medical education and knowledge about immigrants and refugees by checking the box that best represents your opinion.

a. I have received specialized training in immigrant and refugee health, tropical medicine, or cross-cultural health.

- ◯ Strongly disagree
- ⊗ Disagree
- ◯ No opinion
- ◯ Agree
- ◯ Strongly agree

c. I feel comfortable with my fund of knowledge regarding immigrant and refugee health.

- ◯ Strongly disagree
- ⊗ Disagree
- ◯ No opinion
- ◯ Agree
- ◯ Strongly agree

d. I would like to have further training in immigrant and refugee health.

- ◯ Strongly disagree
- ◯ Disagree
- ◯ No opinion
- ◯ Agree
- ⊗ Strongly agree

e. If you agree with the above, please indicate all the contexts in which you would like to receive this training:

- ☒ As part of my residency.
- ☒ A special program.
- ☐ As part of my fellowship.

SECTION C: Attitudes towards immigrant health

A. Please indicate your level of agreement with the following statements regarding immigrant and refugee health by checking the box that best represents your opinion.

a. I enjoy taking care of immigrants and refugees.

- ◯ Never
- ◯ Rarely
- ◯ Sometimes
- ⊗ Usually
- ◯ Always

b. Please indicate the reasons that you enjoy taking care of immigrants and refugees (may choose more than one).

- ☒ Tropical and other conditions not frequently diagnosed in US-born patients
- ☒ Learning about other cultures
- ☐ They don't complain as much
- ☒ Being able to hear their stories
- ☐ Their care is more complicated
- ☐ Their care is less complicated
- ☐ They are very appreciative of your help.
- ☐ They are extremely vulnerable
- ☐ Other:

c. Taking care of immigrants and refugees is more challenging than taking care of US born patients.

- ◯ Never
- ◯ Rarely
- ⊗ Sometimes
- ◯ Usually
- ◯ Always

d. Please mark all the challenges that you face as a provider when providing care to immigrants and refugees (may choose more than one):

- ☒ Language barriers
- ☒ Insurance barriers
- ☒ Cultural barriers
- ☒ Finding a professional interpreter
- ☐ Knowing how to work with a professional interpreter
- ☒ Time constraints
- ☒ My own knowledge related to tropical and travel medicine
- ☒ Transportation problems for the patient
- ☒ Patients not understanding treatment plan
- ☒ Patients not following treatment plan
- ☒ My lack of knowledge regarding the patient's culture
- ☐ Bias or stereotyping
- ☐ Other:

e. Please mark all of the challenges faced by immigrant and refugee populations when receiving healthcare that you have perceived or witnessed (may choose more than one):

- ☒ Language barriers
- ☒ Insurance barriers
- ☒ Cultural barriers
- ☒ Finding a professional interpreter
- ☒ Knowing how to work with a professional interpreter
- ☒ Time constraints
- ☒ Insufficiently trained health care providers
- ☒ Transportation problems for the patient
- ☐ Food insufficiency
- ☒ Need for child care
- ☒ Patients not understanding treatment plan
- ☒ Patients not following treatment plan
- ☐ My lack of knowledge regarding the patient's culture
- ☐ Bias or stereotyping
- ☒ Trust issues
- ☐ Other...

f. Rank how well immigrants and refugees understand the healthcare that you are trying to provide.

- ◯ Significantly less than a US born individual
- ⊗ Less than a US born individual
- ◯ Equivalent to a US born individual
- ◯ More than a US born patient
- ◯ Significantly more than a US born individual

g. Immigrants and refugees adhere to treatment plans and follow my recommendations.

- ◯ Never
- ◯ Rarely
- ◯ Sometimes
- ⊗ Usually
- ◯ Always

h. Immigrants and refugees should receive the same care and insurance coverage as US born patients.

- ◯ Never
- ◯ Rarely
- ◯ Sometimes
- ◯ Usually
- ⊗ Always

i. Immigrants and refugees who are undocumented should receive the same care and insurance coverage as US born patients.

- ◯ Never
- ◯ Rarely
- ◯ Sometimes
- ◯ Usually
- ⊗ Always

j. Every physician is professionally obligated to care for immigrants and refugees if they present to your clinic or hospital.

- ◯ Strongly disagree
- ◯ Disagree
- ◯ No opinion
- ◯ Agree
- ⊗ Strongly agree

k. Is healthcare a human right?

⊗ Yes
◯ No

B. If you wish, please tell us about what you enjoy or do not enjoy about immigrant and refugee health care and the greatest challenges you face in caring for this population.

____

SECTION D: DEMOGRAPHIC INFORMATION
Please answer the following questions by checking the box in front of the response choice that best describes you.

a. Your age?

◯ 20 to 24
⊗ 25 to 29
◯ 30 to 34
◯ 35 to 39
◯ 40 or older

b. Your gender?

⊗ Female
◯ Male
◯ Other

c. Are you Hispanic or Latino?

◯ Yes
⊗ No

d. What is your race? (Select one or more responses)

◯ American Indian or Alaska Native
◯ Asian (Please specify):
◯ Black or African American
◯ Native Hawaiian or Other Pacific Islander
⊗ White
◯ Other (Please specify):

e. Were you born in the United States?

⊗ Yes
◯ No

g. Your residency year?

⊗ PGY1
◯ PGY2
◯ PGY3
◯ PGY4
◯ PGY5

h. How would you classify your political ideology?

◯ Conservative
◯ Somewhat conservative
◯ Moderate
◯ Somewhat liberal
⊗ Liberal
◯ Other (Please specify):

i. Estimated level of educational debt?

◯ None
◯ Less than $50,000
◯ $50,000 - $100,000
⊗ $100,000 - $200,000
◯ $200,000 or more

j. Do you plan to subspecialize?

◯ Yes
⊗ No

k. Languages spoken?

- [x] English
- [ ] Spanish
- [ ] French
- [ ] Hmong
- [ ] Somali
- [ ] Japanese
- [ ] Chinese
- [ ] Russian
- [ ] Ethiopian
- [ ] Other ___________

l. Are you in the Global Health Pathway?

- (⊗) Yes
- ( ) No

m. Did you earn your degree in the US?

- (⊗) Yes
- ( ) No

n. What residency program are you in?

- ( ) Internal Medicine
- ( ) Med-Peds
- ( ) Pediatrics
- (⊗) Family Practice
- ( ) Neurology
- ( ) Psychiatry
- ( ) ObGyn
- ( ) Neurosurgery
- ( ) General Surgery
- ( ) Orthopedic Surgery
- ( ) Urology
- ( ) Surgical sub-specialty (please specify in text box below)
- ( ) Non-clinical specialty (radiology, pathology; please specify in text box below)

Medical Trainees' attitudes, knowledge, and experience with immigrant and refugee health

Response was added on 11/04/2013 8:44am.

SECTION A: Personal experience with immigrant and refugee health care.

A. Please indicate your level of agreement with the following statements regarding your personal experience with immigrant and refugee health care by checking the box that best represents your experience.

a. During my inpatient rotations, I take care of the following percentage of immigrant and refugee patients:

- ◯ None
- ◯ 0 -5%
- ◯ 5-10%
- ⊗ 10-25%
- ◯ > 25%

b. During my outpatient rotations, I take care of the following percentage of immigrant and refugee patients:

- ◯ None
- ◯ 0-10%
- ⊗ 10 -25%
- ◯ 25-50%
- ◯ 50-75%
- ◯ >75%

c. I would like to take care of more immigrant and refugee patients.

- ◯ Strongly disagree
- ⊗ Disagree
- ◯ No opinion
- ◯ Agree
- ◯ Strongly agree

d. I plan to take care of immigrants and refugees when I finish residency.

- ◯ Strongly disagree
- ◯ Disagree
- ◯ No opinion
- ⊗ Agree
- ◯ Strongly agree

e. I plan to do short term (< 6 months) international work when I finish residency.

- ◯ Strongly disagree
- ⊗ Disagree
- ◯ No opinion
- ◯ Agree
- ◯ Strongly agree

f. I plan to do long term (>6 months) international work when I finish residency.

- ◯ Strongly disagree
- ⊗ Disagree
- ◯ No opinion
- ◯ Agree
- ◯ Strongly agree

g. I plan to work in health disparities in the following way after residency:

rural population

SECTION B: MEDICAL EDUCATION

A. Please indicate your level of agreement with the following statements regarding your medical education and knowledge about immigrants and refugees by checking the box that best represents your opinion.

a. I have received specialized training in immigrant and refugee health, tropical medicine, or cross-cultural health.

- ◯ Strongly disagree
- ⊗ Disagree
- ◯ No opinion
- ◯ Agree
- ◯ Strongly agree

c. I feel comfortable with my fund of knowledge regarding immigrant and refugee health.

- ◯ Strongly disagree
- ⊗ Disagree
- ◯ No opinion
- ◯ Agree
- ◯ Strongly agree

d. I would like to have further training in immigrant and refugee health.

- ◯ Strongly disagree
- ◯ Disagree
- ◯ No opinion
- ⊗ Agree
- ◯ Strongly agree

e. If you agree with the above, please indicate all the contexts in which you would like to receive this training:

- ☒ As part of my residency.
- ☐ A special program.
- ☐ As part of my fellowship.

SECTION C: Attitudes towards immigrant health

A. Please indicate your level of agreement with the following statements regarding immigrant and refugee health by checking the box that best represents your opinion.

a. I enjoy taking care of immigrants and refugees.

- ◯ Never
- ◯ Rarely
- ◯ Sometimes
- ⊗ Usually
- ◯ Always

b. Please indicate the reasons that you enjoy taking care of immigrants and refugees (may choose more than one).

- ☒ Tropical and other conditions not frequently diagnosed in US-born patients
- ☒ Learning about other cultures
- ☐ They don't complain as much
- ☒ Being able to hear their stories
- ☐ Their care is more complicated
- ☐ Their care is less complicated
- ☐ They are very appreciative of your help.
- ☐ They are extremely vulnerable
- ☐ Other:

c. Taking care of immigrants and refugees is more challenging than taking care of US born patients.

- ◯ Never
- ◯ Rarely
- ⊗ Sometimes
- ◯ Usually
- ◯ Always

d. Please mark all the challenges that you face as a provider when providing care to immigrants and refugees (may choose more than one):

- ☒ Language barriers
- ☒ Insurance barriers
- ☒ Cultural barriers
- ☒ Finding a professional interpreter
- ☐ Knowing how to work with a professional interpreter
- ☒ Time constraints
- ☒ My own knowledge related to tropical and travel medicine
- ☐ Transportation problems for the patient
- ☐ Patients not understanding treatment plan
- ☒ Patients not following treatment plan
- ☒ My lack of knowledge regarding the patient's culture
- ☐ Bias or stereotyping
- ☐ Other:

e. Please mark all of the challenges faced by immigrant and refugee populations when receiving healthcare that you have perceived or witnessed (may choose more than one):

- ☒ Language barriers
- ☒ Insurance barriers
- ☒ Cultural barriers
- ☒ Finding a professional interpreter
- ☒ Knowing how to work with a professional interpreter
- ☒ Time constraints
- ☒ Insufficiently trained health care providers
- ☒ Transportation problems for the patient
- ☒ Food insufficiency
- ☒ Need for child care
- ☒ Patients not understanding treatment plan
- ☒ Patients not following treatment plan
- ☒ My lack of knowledge regarding the patient's culture
- ☒ Bias or stereotyping
- ☒ Trust issues
- ☐ Other...

f. Rank how well immigrants and refugees understand the healthcare that you are trying to provide.

- ○ Significantly less than a US born individual
- ⊗ Less than a US born individual
- ○ Equivalent to a US born individual
- ○ More than a US born patient
- ○ Significantly more than a US born individual

g. Immigrants and refugees adhere to treatment plans and follow my recommendations.

- ○ Never
- ○ Rarely
- ⊗ Sometimes
- ○ Usually
- ○ Always

h. Immigrants and refugees should receive the same care and insurance coverage as US born patients.

- ○ Never
- ○ Rarely
- ○ Sometimes
- ○ Usually
- ⊗ Always

i. Immigrants and refugees who are undocumented should receive the same care and insurance coverage as US born patients.

- ○ Never
- ○ Rarely
- ○ Sometimes
- ⊗ Usually
- ○ Always

j. Every physician is professionally obligated to care for immigrants and refugees if they present to your clinic or hospital.

- ○ Strongly disagree
- ○ Disagree
- ○ No opinion
- ○ Agree
- ⊗ Strongly agree

k. Is healthcare a human right?
⊗ Yes
◯ No

B. If you wish, please tell us about what you enjoy or do not enjoy about immigrant and refugee health care and the greatest challenges you face in caring for this population.
____

SECTION D: DEMOGRAPHIC INFORMATION
Please answer the following questions by checking the box in front of the response choice that best describes you.

a. Your age?
◯ 20 to 24
◯ 25 to 29
⊗ 30 to 34
◯ 35 to 39
◯ 40 or older

b. Your gender?
⊗ Female
◯ Male
◯ Other

c. Are you Hispanic or Latino?
◯ Yes
⊗ No

d. What is your race? (Select one or more responses)
◯ American Indian or Alaska Native
◯ Asian (Please specify):
◯ Black or African American
◯ Native Hawaiian or Other Pacific Islander
⊗ White
◯ Other (Please specify):

e. Were you born in the United States?
⊗ Yes
◯ No

g. Your residency year?
◯ PGY1
◯ PGY2
⊗ PGY3
◯ PGY4
◯ PGY5

h. How would you classify your political ideology?
◯ Conservative
◯ Somewhat conservative
◯ Moderate
◯ Somewhat liberal
⊗ Liberal
◯ Other (Please specify):

i. Estimated level of educational debt?
◯ None
◯ Less than $50,000
◯ $50,000 - $100,000
◯ $100,000 - $200,000
⊗ $200,000 or more

j. Do you plan to subspecialize?
◯ Yes
⊗ No

k. Languages spoken?

- ☒ English
- ☐ Spanish
- ☐ French
- ☐ Hmong
- ☐ Somali
- ☐ Japanese
- ☐ Chinese
- ☐ Russian
- ☐ Ethiopian
- ☐ Other ___________

l. Are you in the Global Health Pathway?

- ○ Yes
- ⊗ No

m. Did you earn your degree in the US?

- ⊗ Yes
- ○ No

n. What residency program are you in?

- ○ Internal Medicine
- ○ Med-Peds
- ○ Pediatrics
- ⊗ Family Practice
- ○ Neurology
- ○ Psychiatry
- ○ ObGyn
- ○ Neurosurgery
- ○ General Surgery
- ○ Orthopedic Surgery
- ○ Urology
- ○ Surgical sub-specialty (please specify in text box below)
- ○ Non-clinical specialty (radiology, pathology; please specify in text box below)

Medical Trainees' attitudes, knowledge, and experience with immigrant and refugee health

Response was added on 11/04/2013 8:49am.

SECTION A: Personal experience with immigrant and refugee health care.

A. Please indicate your level of agreement with the following statements regarding your personal experience with immigrant and refugee health care by checking the box that best represents your experience.

a. During my inpatient rotations, I take care of the following percentage of immigrant and refugee patients:

- ◯ None
- ◯ 0 -5%
- ◯ 5-10%
- ⊗ 10-25%
- ◯ > 25%

b. During my outpatient rotations, I take care of the following percentage of immigrant and refugee patients:

- ◯ None
- ◯ 0-10%
- ⊗ 10 -25%
- ◯ 25-50%
- ◯ 50-75%
- ◯ >75%

c. I would like to take care of more immigrant and refugee patients.

- ◯ Strongly disagree
- ◯ Disagree
- ⊗ No opinion
- ◯ Agree
- ◯ Strongly agree

d. I plan to take care of immigrants and refugees when I finish residency.

- ◯ Strongly disagree
- ◯ Disagree
- ◯ No opinion
- ⊗ Agree
- ◯ Strongly agree

e. I plan to do short term (< 6 months) international work when I finish residency.

- ◯ Strongly disagree
- ◯ Disagree
- ◯ No opinion
- ⊗ Agree
- ◯ Strongly agree

f. I plan to do long term (>6 months) international work when I finish residency.

- ◯ Strongly disagree
- ⊗ Disagree
- ◯ No opinion
- ◯ Agree
- ◯ Strongly agree

g. I plan to work in health disparities in the following way after residency:

Clinic and international travel

SECTION B: MEDICAL EDUCATION

A. Please indicate your level of agreement with the following statements regarding your medical education and knowledge about immigrants and refugees by checking the box that best represents your opinion.

a. I have received specialized training in immigrant and refugee health, tropical medicine, or cross-cultural health.

◯ Strongly disagree
◯ Disagree
◯ No opinion
⊗ Agree
◯ Strongly agree

b. If you have received specialized training in immigrant and refugee health, tropical medicine, or cross-cultural health, please indicate all the contexts in which you received this training:

☐ As an undergraduate.
☒ As a medical student.
☒ As part of my residency.
☐ A special program.
☐ As part of my fellowship.
☐ As part of a degree program (e.g. MPH)
☐ Other:

c. I feel comfortable with my fund of knowledge regarding immigrant and refugee health.

◯ Strongly disagree
⊗ Disagree
◯ No opinion
◯ Agree
◯ Strongly agree

d. I would like to have further training in immigrant and refugee health.

◯ Strongly disagree
◯ Disagree
◯ No opinion
⊗ Agree
◯ Strongly agree

e. If you agree with the above, please indicate all the contexts in which you would like to receive this training:
□□

☒ As part of my residency.
☒ A special program.
☐ As part of my fellowship.

SECTION C: Attitudes towards immigrant health

A.□Please indicate your level of agreement with the following statements regarding immigrant and refugee health by checking the box that best represents your opinion.

a. I enjoy taking care of immigrants and refugees.

◯ Never
◯ Rarely
◯ Sometimes
⊗ Usually
◯ Always

b. Please indicate the reasons that you enjoy taking care of immigrants and refugees (may choose more than one).

☒ Tropical and other conditions not frequently diagnosed in US-born patients
☒ Learning about other cultures
☐ They don't complain as much
☒ Being able to hear their stories
☐ Their care is more complicated
☐ Their care is less complicated
☐ They are very appreciative of your help.
☐ They are extremely vulnerable
☐ Other:

c. Taking care of immigrants and refugees is more challenging than taking care of US born patients.

◯ Never
◯ Rarely
⊗ Sometimes
◯ Usually
◯ Always

d. Please mark all the challenges that you face as a provider when providing care to immigrants and refugees (may choose more than one):

- ☒ Language barriers
- ☒ Insurance barriers
- ☒ Cultural barriers
- ☐ Finding a professional interpreter
- ☐ Knowing how to work with a professional interpreter
- ☐ Time constraints
- ☐ My own knowledge related to tropical and travel medicine
- ☐ Transportation problems for the patient
- ☒ Patients not understanding treatment plan
- ☒ Patients not following treatment plan
- ☐ My lack of knowledge regarding the patient's culture
- ☐ Bias or stereotyping
- ☐ Other:

e. Please mark all of the challenges faced by immigrant and refugee populations when receiving healthcare that you have perceived or witnessed (may choose more than one):

- ☒ Language barriers
- ☐ Insurance barriers
- ☐ Cultural barriers
- ☒ Finding a professional interpreter
- ☒ Knowing how to work with a professional interpreter
- ☐ Time constraints
- ☐ Insufficiently trained health care providers
- ☒ Transportation problems for the patient
- ☐ Food insufficiency
- ☒ Need for child care
- ☐ Patients not understanding treatment plan
- ☐ Patients not following treatment plan
- ☒ My lack of knowledge regarding the patient's culture
- ☒ Bias or stereotyping
- ☐ Trust issues
- ☐ Other...

f. Rank how well immigrants and refugees understand the healthcare that you are trying to provide.

- ○ Significantly less than a US born individual
- ○ Less than a US born individual
- ⊗ Equivalent to a US born individual
- ○ More than a US born patient
- ○ Significantly more than a US born individual

g. Immigrants and refugees adhere to treatment plans and follow my recommendations.

- ○ Never
- ○ Rarely
- ⊗ Sometimes
- ○ Usually
- ○ Always

h. Immigrants and refugees should receive the same care and insurance coverage as US born patients.

- ○ Never
- ○ Rarely
- ⊗ Sometimes
- ○ Usually
- ○ Always

i. Immigrants and refugees who are undocumented should receive the same care and insurance coverage as US born patients.

- ○ Never
- ○ Rarely
- ○ Sometimes
- ⊗ Usually
- ○ Always

j. Every physician is professionally obligated to care for immigrants and refugees if they present to your clinic or hospital.

- ○ Strongly disagree
- ○ Disagree
- ○ No opinion
- ⊗ Agree
- ○ Strongly agree

k. Is healthcare a human right?
- ⊗ Yes
- ◯ No

B. If you wish, please tell us about what you enjoy or do not enjoy about immigrant and refugee health care and the greatest challenges you face in caring for this population.

____

SECTION D: DEMOGRAPHIC INFORMATION
Please answer the following questions by checking the box in front of the response choice that best describes you.

a. Your age?
- ◯ 20 to 24
- ⊗ 25 to 29
- ◯ 30 to 34
- ◯ 35 to 39
- ◯ 40 or older

b. Your gender?
- ◯ Female
- ⊗ Male
- ◯ Other

c.Are you Hispanic or Latino?
- ◯ Yes
- ⊗ No

d. What is your race? (Select one or more responses)
- ◯ American Indian or Alaska Native
- ◯ Asian (Please specify):
- ◯ Black or African American
- ◯ Native Hawaiian or Other Pacific Islander
- ⊗ White
- ◯ Other (Please specify):

e.Were you born in the United States?
- ⊗ Yes
- ◯ No

g. Your residency year?
- ◯ PGY1
- ◯ PGY2
- ⊗ PGY3
- ◯ PGY4
- ◯ PGY5

h. How would you classify your political ideology?
- ◯ Conservative
- ◯ Somewhat conservative
- ◯ Moderate
- ⊗ Somewhat liberal
- ◯ Liberal
- ◯ Other (Please specify):

i. Estimated level of educational debt?
- ◯ None
- ◯ Less than $50,000
- ◯ $50,000 - $100,000
- ◯ $100,000 - $200,000
- ⊗ $200,000 or more

j.Do you plan to subspecialize?
- ⊗ Yes
- ◯ No

k. Languages spoken?

- [x] English
- [ ] Spanish
- [ ] French
- [ ] Hmong
- [ ] Somali
- [ ] Japanese
- [ ] Chinese
- [ ] Russian
- [ ] Ethiopian
- [ ] Other ___________

l. Are you in the Global Health Pathway?

- (X) Yes
- ( ) No

m. Did you earn your degree in the US?

- (X) Yes
- ( ) No

n. What residency program are you in?

- ( ) Internal Medicine
- (X) Med-Peds
- ( ) Pediatrics
- ( ) Family Practice
- ( ) Neurology
- ( ) Psychiatry
- ( ) ObGyn
- ( ) Neurosurgery
- ( ) General Surgery
- ( ) Orthopedic Surgery
- ( ) Urology
- ( ) Surgical sub-specialty (please specify in text box below)
- ( ) Non-clinical specialty (radiology, pathology; please specify in text box below)

Medical Trainees' attitudes, knowledge, and experience with immigrant and refugee health

Response was added on 11/04/2013 11:09am.

SECTION A: Personal experience with immigrant and refugee health care.

A. Please indicate your level of agreement with the following statements regarding your personal experience with immigrant and refugee health care by checking the box that best represents your experience.

a. During my inpatient rotations, I take care of the following percentage of immigrant and refugee patients:

- ◯ None
- ◯ 0 -5%
- ⊗ 5-10%
- ◯ 10-25%
- ◯ > 25%

b. During my outpatient rotations, I take care of the following percentage of immigrant and refugee patients:

- ◯ None
- ◯ 0-10%
- ⊗ 10 -25%
- ◯ 25-50%
- ◯ 50-75%
- ◯ >75%

c. I would like to take care of more immigrant and refugee patients.

- ◯ Strongly disagree
- ◯ Disagree
- ⊗ No opinion
- ◯ Agree
- ◯ Strongly agree

d. I plan to take care of immigrants and refugees when I finish residency.

- ◯ Strongly disagree
- ◯ Disagree
- ⊗ No opinion
- ◯ Agree
- ◯ Strongly agree

e. I plan to do short term (< 6 months) international work when I finish residency.

- ◯ Strongly disagree
- ◯ Disagree
- ⊗ No opinion
- ◯ Agree
- ◯ Strongly agree

f. I plan to do long term (>6 months) international work when I finish residency.

- ◯ Strongly disagree
- ⊗ Disagree
- ◯ No opinion
- ◯ Agree
- ◯ Strongly agree

g. I plan to work in health disparities in the following way after residency:

Will likely serve an urban, underserved population as I continue my training in critical care at an urban, academic medical center.

SECTION B: MEDICAL EDUCATION

A. Please indicate your level of agreement with the following statements regarding your medical education and knowledge about immigrants and refugees by checking the box that best represents your opinion.

a. I have received specialized training in immigrant and refugee health, tropical medicine, or cross-cultural health.

- ◯ Strongly disagree
- ◯ Disagree
- ◯ No opinion
- ⊗ Agree
- ◯ Strongly agree

b. If you have received specialized training in immigrant and refugee health, tropical medicine, or cross-cultural health, please indicate all the contexts in which you received this training:

- ☐ As an undergraduate.
- ☒ As a medical student.
- ☒ As part of my residency.
- ☐ A special program.
- ☐ As part of my fellowship.
- ☐ As part of a degree program (e.g. MPH)
- ☐ Other:

c. I feel comfortable with my fund of knowledge regarding immigrant and refugee health.

- ◯ Strongly disagree
- ⊗ Disagree
- ◯ No opinion
- ◯ Agree
- ◯ Strongly agree

d. I would like to have further training in immigrant and refugee health.

- ◯ Strongly disagree
- ◯ Disagree
- ◯ No opinion
- ◯ Agree
- ⊗ Strongly agree

e. If you agree with the above, please indicate all the contexts in which you would like to receive this training:
□□

- ☒ As part of my residency.
- ☐ A special program.
- ☒ As part of my fellowship.

SECTION C: Attitudes towards immigrant health

A.□Please indicate your level of agreement with the following statements regarding immigrant and refugee health by checking the box that best represents your opinion.

a. I enjoy taking care of immigrants and refugees.

- ◯ Never
- ◯ Rarely
- ◯ Sometimes
- ⊗ Usually
- ◯ Always

b. Please indicate the reasons that you enjoy taking care of immigrants and refugees (may choose more than one).

- ☒ Tropical and other conditions not frequently diagnosed in US-born patients
- ☒ Learning about other cultures
- ☐ They don't complain as much
- ☐ Being able to hear their stories
- ☒ Their care is more complicated
- ☐ Their care is less complicated
- ☐ They are very appreciative of your help.
- ☐ They are extremely vulnerable
- ☐ Other:

c. Taking care of immigrants and refugees is more challenging than taking care of US born patients.

- ◯ Never
- ◯ Rarely
- ◯ Sometimes
- ⊗ Usually
- ◯ Always

d. Please mark all the challenges that you face as a provider when providing care to immigrants and refugees (may choose more than one):

- [x] Language barriers
- [ ] Insurance barriers
- [x] Cultural barriers
- [x] Finding a professional interpreter
- [ ] Knowing how to work with a professional interpreter
- [x] Time constraints
- [x] My own knowledge related to tropical and travel medicine
- [ ] Transportation problems for the patient
- [ ] Patients not understanding treatment plan
- [ ] Patients not following treatment plan
- [ ] My lack of knowledge regarding the patient's culture
- [ ] Bias or stereotyping
- [ ] Other:

e. Please mark all of the challenges faced by immigrant and refugee populations when receiving healthcare that you have perceived or witnessed (may choose more than one):

- [x] Language barriers
- [x] Insurance barriers
- [x] Cultural barriers
- [x] Finding a professional interpreter
- [ ] Knowing how to work with a professional interpreter
- [x] Time constraints
- [x] Insufficiently trained health care providers
- [x] Transportation problems for the patient
- [ ] Food insufficiency
- [ ] Need for child care
- [ ] Patients not understanding treatment plan
- [ ] Patients not following treatment plan
- [x] My lack of knowledge regarding the patient's culture
- [ ] Bias or stereotyping
- [ ] Trust issues
- [ ] Other...

f. Rank how well immigrants and refugees understand the healthcare that you are trying to provide.

- ( ) Significantly less than a US born individual
- ( ) Less than a US born individual
- (x) Equivalent to a US born individual
- ( ) More than a US born patient
- ( ) Significantly more than a US born individual

g. Immigrants and refugees adhere to treatment plans and follow my recommendations.

- ( ) Never
- ( ) Rarely
- ( ) Sometimes
- (x) Usually
- ( ) Always

h. Immigrants and refugees should receive the same care and insurance coverage as US born patients.

- ( ) Never
- ( ) Rarely
- ( ) Sometimes
- ( ) Usually
- (x) Always

i. Immigrants and refugees who are undocumented should receive the same care and insurance coverage as US born patients.

- ( ) Never
- ( ) Rarely
- ( ) Sometimes
- ( ) Usually
- (x) Always

j. Every physician is professionally obligated to care for immigrants and refugees if they present to your clinic or hospital.

- ( ) Strongly disagree
- ( ) Disagree
- ( ) No opinion
- ( ) Agree
- (x) Strongly agree

k. Is healthcare a human right?
⊗ Yes
○ No

B. If you wish, please tell us about what you enjoy or do not enjoy about immigrant and refugee health care and the greatest challenges you face in caring for this population.
____

SECTION D: DEMOGRAPHIC INFORMATION
Please answer the following questions by checking the box in front of the response choice that best describes you.

a. Your age?
○ 20 to 24
⊗ 25 to 29
○ 30 to 34
○ 35 to 39
○ 40 or older

b. Your gender?
⊗ Female
○ Male
○ Other

c.Are you Hispanic or Latino?
○ Yes
⊗ No

d. What is your race? (Select one or more responses)
○ American Indian or Alaska Native
○ Asian (Please specify):
○ Black or African American
○ Native Hawaiian or Other Pacific Islander
⊗ White
○ Other (Please specify):

e.Were you born in the United States?
⊗ Yes
○ No

g. Your residency year?
○ PGY1
○ PGY2
⊗ PGY3
○ PGY4
○ PGY5

h. How would you classify your political ideology?
○ Conservative
○ Somewhat conservative
⊗ Moderate
○ Somewhat liberal
○ Liberal
○ Other (Please specify):

i. Estimated level of educational debt?
○ None
○ Less than $50,000
○ $50,000 - $100,000
⊗ $100,000 - $200,000
○ $200,000 or more

j.Do you plan to subspecialize?
⊗ Yes
○ No

k. Languages spoken?

- [x] English
- [ ] Spanish
- [ ] French
- [ ] Hmong
- [ ] Somali
- [ ] Japanese
- [ ] Chinese
- [ ] Russian
- [ ] Ethiopian
- [ ] Other ___________

l. Are you in the Global Health Pathway?

- ◯ Yes
- ⊗ No

m. Did you earn your degree in the US?

- ⊗ Yes
- ◯ No

n. What residency program are you in?

- ◯ Internal Medicine
- ◯ Med-Peds
- ⊗ Pediatrics
- ◯ Family Practice
- ◯ Neurology
- ◯ Psychiatry
- ◯ ObGyn
- ◯ Neurosurgery
- ◯ General Surgery
- ◯ Orthopedic Surgery
- ◯ Urology
- ◯ Surgical sub-specialty (please specify in text box below)
- ◯ Non-clinical specialty (radiology, pathology; please specify in text box below)

Medical Trainees' attitudes, knowledge, and experience with immigrant and refugee health

Response was added on 11/04/2013 2:04pm.

SECTION A: Personal experience with immigrant and refugee health care.

A. Please indicate your level of agreement with the following statements regarding your personal experience with immigrant and refugee health care by checking the box that best represents your experience.

a. During my inpatient rotations, I take care of the following percentage of immigrant and refugee patients:

- ◯ None
- ◯ 0 -5%
- ◯ 5-10%
- ⊗ 10-25%
- ◯ > 25%

b. During my outpatient rotations, I take care of the following percentage of immigrant and refugee patients:

- ◯ None
- ◯ 0-10%
- ⊗ 10 -25%
- ◯ 25-50%
- ◯ 50-75%
- ◯ >75%

c. I would like to take care of more immigrant and refugee patients.

- ◯ Strongly disagree
- ◯ Disagree
- ⊗ No opinion
- ◯ Agree
- ◯ Strongly agree

d. I plan to take care of immigrants and refugees when I finish residency.

- ◯ Strongly disagree
- ◯ Disagree
- ◯ No opinion
- ◯ Agree
- ⊗ Strongly agree

e. I plan to do short term (< 6 months) international work when I finish residency.

- ◯ Strongly disagree
- ◯ Disagree
- ⊗ No opinion
- ◯ Agree
- ◯ Strongly agree

f. I plan to do long term (>6 months) international work when I finish residency.

- ◯ Strongly disagree
- ◯ Disagree
- ⊗ No opinion
- ◯ Agree
- ◯ Strongly agree

g. I plan to work in health disparities in the following way after residency:

Neutral

SECTION B: MEDICAL EDUCATION

A. Please indicate your level of agreement with the following statements regarding your medical education and knowledge about immigrants and refugees by checking the box that best represents your opinion.

a. I have received specialized training in immigrant and refugee health, tropical medicine, or cross-cultural health.

- ◯ Strongly disagree
- ◯ Disagree
- ⊗ No opinion
- ◯ Agree
- ◯ Strongly agree

c. I feel comfortable with my fund of knowledge regarding immigrant and refugee health.

- ◯ Strongly disagree
- ◯ Disagree
- ◯ No opinion
- ⊗ Agree
- ◯ Strongly agree

d. I would like to have further training in immigrant and refugee health.

- ◯ Strongly disagree
- ◯ Disagree
- ◯ No opinion
- ⊗ Agree
- ◯ Strongly agree

e. If you agree with the above, please indicate all the contexts in which you would like to receive this training:
□□

- ☒ As part of my residency.
- ☐ A special program.
- ☐ As part of my fellowship.

SECTION C: Attitudes towards immigrant health

A.□Please indicate your level of agreement with the following statements regarding immigrant and refugee health by checking the box that best represents your opinion.

a. I enjoy taking care of immigrants and refugees.

- ◯ Never
- ◯ Rarely
- ◯ Sometimes
- ⊗ Usually
- ◯ Always

b. Please indicate the reasons that you enjoy taking care of immigrants and refugees (may choose more than one).

- ☐ Tropical and other conditions not frequently diagnosed in US-born patients
- ☒ Learning about other cultures
- ☐ They don't complain as much
- ☐ Being able to hear their stories
- ☐ Their care is more complicated
- ☐ Their care is less complicated
- ☒ They are very appreciative of your help.
- ☐ They are extremely vulnerable
- ☐ Other:

c. Taking care of immigrants and refugees is more challenging than taking care of US born patients.

- ◯ Never
- ◯ Rarely
- ⊗ Sometimes
- ◯ Usually
- ◯ Always

d. Please mark all the challenges that you face as a provider when providing care to immigrants and refugees (may choose more than one):

- ☒ Language barriers
- ☐ Insurance barriers
- ☒ Cultural barriers
- ☒ Finding a professional interpreter
- ☒ Knowing how to work with a professional interpreter
- ☐ Time constraints
- ☐ My own knowledge related to tropical and travel medicine
- ☐ Transportation problems for the patient
- ☒ Patients not understanding treatment plan
- ☐ Patients not following treatment plan
- ☒ My lack of knowledge regarding the patient's culture
- ☐ Bias or stereotyping
- ☐ Other:

e. Please mark all of the challenges faced by immigrant and refugee populations when receiving healthcare that you have perceived or witnessed (may choose more than one):

- ☒ Language barriers
- ☒ Insurance barriers
- ☒ Cultural barriers
- ☒ Finding a professional interpreter
- ☒ Knowing how to work with a professional interpreter
- ☒ Time constraints
- ☐ Insufficiently trained health care providers
- ☐ Transportation problems for the patient
- ☐ Food insufficiency
- ☒ Need for child care
- ☒ Patients not understanding treatment plan
- ☒ Patients not following treatment plan
- ☒ My lack of knowledge regarding the patient's culture
- ☒ Bias or stereotyping
- ☐ Trust issues
- ☐ Other...

f. Rank how well immigrants and refugees understand the healthcare that you are trying to provide.

- ○ Significantly less than a US born individual
- ⊗ Less than a US born individual
- ○ Equivalent to a US born individual
- ○ More than a US born patient
- ○ Significantly more than a US born individual

g. Immigrants and refugees adhere to treatment plans and follow my recommendations.

- ○ Never
- ○ Rarely
- ○ Sometimes
- ⊗ Usually
- ○ Always

h. Immigrants and refugees should receive the same care and insurance coverage as US born patients.

- ○ Never
- ○ Rarely
- ○ Sometimes
- ○ Usually
- ⊗ Always

i. Immigrants and refugees who are undocumented should receive the same care and insurance coverage as US born patients.

- ○ Never
- ○ Rarely
- ○ Sometimes
- ○ Usually
- ⊗ Always

j. Every physician is professionally obligated to care for immigrants and refugees if they present to your clinic or hospital.

- ○ Strongly disagree
- ○ Disagree
- ○ No opinion
- ○ Agree
- ⊗ Strongly agree

k. Is healthcare a human right?

⊗ Yes
◯ No

B. If you wish, please tell us about what you enjoy or do not enjoy about immigrant and refugee health care and the greatest challenges you face in caring for this population.

____

SECTION D: DEMOGRAPHIC INFORMATION
Please answer the following questions by checking the box in front of the response choice that best describes you.

a. Your age?

◯ 20 to 24
⊗ 25 to 29
◯ 30 to 34
◯ 35 to 39
◯ 40 or older

b. Your gender?

◯ Female
⊗ Male
◯ Other

c. Are you Hispanic or Latino?

◯ Yes
⊗ No

d. What is your race? (Select one or more responses)

◯ American Indian or Alaska Native
⊗ Asian (Please specify):
◯ Black or African American
◯ Native Hawaiian or Other Pacific Islander
◯ White
◯ Other (Please specify):

e. Were you born in the United States?

⊗ Yes
◯ No

g. Your residency year?

⊗ PGY1
◯ PGY2
◯ PGY3
◯ PGY4
◯ PGY5

h. How would you classify your political ideology?

◯ Conservative
⊗ Somewhat conservative
◯ Moderate
◯ Somewhat liberal
◯ Liberal
◯ Other (Please specify):

i. Estimated level of educational debt?

◯ None
⊗ Less than $50,000
◯ $50,000 - $100,000
◯ $100,000 - $200,000
◯ $200,000 or more

j. Do you plan to subspecialize?

⊗ Yes
◯ No

k. Languages spoken?

☒ English
☐ Spanish
☐ French
☐ Hmong
☐ Somali
☐ Japanese
☐ Chinese
☐ Russian
☐ Ethiopian
☐ Other ___________

l. Are you in the Global Health Pathway?

◯ Yes
⊗ No

m.□ Did you earn your degree in the US?

⊗ Yes
◯ No

n. What residency program are you in?

◯ Internal Medicine
◯ Med-Peds
◯ Pediatrics
◯ Family Practice
◯ Neurology
◯ Psychiatry
◯ ObGyn
◯ Neurosurgery
◯ General Surgery
◯ Orthopedic Surgery
◯ Urology
⊗ Surgical sub-specialty (please specify in text box below)
◯ Non-clinical specialty (radiology, pathology; please specify in text box below)

otolaryngology

Medical Trainees' attitudes, knowledge, and experience with immigrant and refugee health

Response was added on 11/04/2013 5:01pm.

SECTION A: Personal experience with immigrant and refugee health care.

A. Please indicate your level of agreement with the following statements regarding your personal experience with immigrant and refugee health care by checking the box that best represents your experience.

a. During my inpatient rotations, I take care of the following percentage of immigrant and refugee patients:

- ○ None
- ○ 0 -5%
- ○ 5-10%
- ⊗ 10-25%
- ○ > 25%

b. During my outpatient rotations, I take care of the following percentage of immigrant and refugee patients:

- ○ None
- ○ 0-10%
- ○ 10 -25%
- ⊗ 25-50%
- ○ 50-75%
- ○ >75%

c. I would like to take care of more immigrant and refugee patients.

- ○ Strongly disagree
- ⊗ Disagree
- ○ No opinion
- ○ Agree
- ○ Strongly agree

d. I plan to take care of immigrants and refugees when I finish residency.

- ○ Strongly disagree
- ○ Disagree
- ⊗ No opinion
- ○ Agree
- ○ Strongly agree

e. I plan to do short term (< 6 months) international work when I finish residency.

- ○ Strongly disagree
- ○ Disagree
- ⊗ No opinion
- ○ Agree
- ○ Strongly agree

f. I plan to do long term (>6 months) international work when I finish residency.

- ○ Strongly disagree
- ⊗ Disagree
- ○ No opinion
- ○ Agree
- ○ Strongly agree

g. I plan to work in health disparities in the following way after residency:

Only if I am employed by a facility that provides healthcare to the socio-economically disadvantaged. I also plan to donate to charity organizations that may provide these services or encourage economic development.

SECTION B: MEDICAL EDUCATION

A. Please indicate your level of agreement with the following statements regarding your medical education and knowledge about immigrants and refugees by checking the box that best represents your opinion.

a. I have received specialized training in immigrant and refugee health, tropical medicine, or cross-cultural health.

- ◯ Strongly disagree
- ◯ Disagree
- ⊗ No opinion
- ◯ Agree
- ◯ Strongly agree

c. I feel comfortable with my fund of knowledge regarding immigrant and refugee health.

- ◯ Strongly disagree
- ◯ Disagree
- ◯ No opinion
- ⊗ Agree
- ◯ Strongly agree

d. I would like to have further training in immigrant and refugee health.

- ◯ Strongly disagree
- ◯ Disagree
- ⊗ No opinion
- ◯ Agree
- ◯ Strongly agree

SECTION C: Attitudes towards immigrant health

A.□Please indicate your level of agreement with the following statements regarding immigrant and refugee health by checking the box that best represents your opinion.

a. I enjoy taking care of immigrants and refugees.

- ◯ Never
- ◯ Rarely
- ⊗ Sometimes
- ◯ Usually
- ◯ Always

b. Please indicate the reasons that you enjoy taking care of immigrants and refugees (may choose more than one).

- ☒ Tropical and other conditions not frequently diagnosed in US-born patients
- ☒ Learning about other cultures
- ☐ They don't complain as much
- ☒ Being able to hear their stories
- ☒ Their care is more complicated
- ☐ Their care is less complicated
- ☒ They are very appreciative of your help.
- ☐ They are extremely vulnerable
- ☐ Other:

c. Taking care of immigrants and refugees is more challenging than taking care of US born patients.

- ◯ Never
- ◯ Rarely
- ⊗ Sometimes
- ◯ Usually
- ◯ Always

d. Please mark all the challenges that you face as a provider when providing care to immigrants and refugees (may choose more than one):

- ☒ Language barriers
- ☐ Insurance barriers
- ☒ Cultural barriers
- ☐ Finding a professional interpreter
- ☐ Knowing how to work with a professional interpreter
- ☐ Time constraints
- ☐ My own knowledge related to tropical and travel medicine
- ☐ Transportation problems for the patient
- ☒ Patients not understanding treatment plan
- ☒ Patients not following treatment plan
- ☐ My lack of knowledge regarding the patient's culture
- ☐ Bias or stereotyping
- ☐ Other:

e. Please mark all of the challenges faced by immigrant and refugee populations when receiving healthcare that you have perceived or witnessed (may choose more than one):

- ☒ Language barriers
- ☐ Insurance barriers
- ☒ Cultural barriers
- ☐ Finding a professional interpreter
- ☐ Knowing how to work with a professional interpreter
- ☐ Time constraints
- ☐ Insufficiently trained health care providers
- ☐ Transportation problems for the patient
- ☐ Food insufficiency
- ☐ Need for child care
- ☒ Patients not understanding treatment plan
- ☒ Patients not following treatment plan
- ☐ My lack of knowledge regarding the patient's culture
- ☐ Bias or stereotyping
- ☐ Trust issues
- ☐ Other...

f. Rank how well immigrants and refugees understand the healthcare that you are trying to provide.

- ◯ Significantly less than a US born individual
- ⊗ Less than a US born individual
- ◯ Equivalent to a US born individual
- ◯ More than a US born patient
- ◯ Significantly more than a US born individual

g. Immigrants and refugees adhere to treatment plans and follow my recommendations.

- ◯ Never
- ◯ Rarely
- ⊗ Sometimes
- ◯ Usually
- ◯ Always

h. Immigrants and refugees should receive the same care and insurance coverage as US born patients.

- ◯ Never
- ◯ Rarely
- ⊗ Sometimes
- ◯ Usually
- ◯ Always

i. Immigrants and refugees who are undocumented should receive the same care and insurance coverage as US born patients.

- ◯ Never
- ⊗ Rarely
- ◯ Sometimes
- ◯ Usually
- ◯ Always

j. Every physician is professionally obligated to care for immigrants and refugees if they present to your clinic or hospital.

- ⊗ Strongly disagree
- ◯ Disagree
- ◯ No opinion
- ◯ Agree
- ◯ Strongly agree

k. Is healthcare a human right?
◯ Yes
⊗ No

B. If you wish, please tell us about what you enjoy or do not enjoy about immigrant and refugee health care and the greatest challenges you face in caring for this population.

Different perspectives and situations are interesting. Often immigrants are extraordinarily nice.

I do not enjoy that they sometimes expect free healthcare and presume that healtcare is not their responsibility.

SECTION D: DEMOGRAPHIC INFORMATION
Please answer the following questions by checking the box in front of the response choice that best describes you.

a. Your age?
◯ 20 to 24
◯ 25 to 29
◯ 30 to 34
⊗ 35 to 39
◯ 40 or older

b. Your gender?
◯ Female
⊗ Male
◯ Other

c. Are you Hispanic or Latino?
◯ Yes
⊗ No

d. What is your race? (Select one or more responses)
◯ American Indian or Alaska Native
◯ Asian (Please specify):
◯ Black or African American
◯ Native Hawaiian or Other Pacific Islander
⊗ White
◯ Other (Please specify):

e. Were you born in the United States?
⊗ Yes
◯ No

g. Your residency year?
⊗ PGY1
◯ PGY2
◯ PGY3
◯ PGY4
◯ PGY5

h. How would you classify your political ideology?
◯ Conservative
⊗ Somewhat conservative
◯ Moderate
◯ Somewhat liberal
◯ Liberal
◯ Other (Please specify):

i. Estimated level of educational debt?
⊗ None
◯ Less than $50,000
◯ $50,000 - $100,000
◯ $100,000 - $200,000
◯ $200,000 or more

j. Do you plan to subspecialize?
⊗ Yes
◯ No

k. Languages spoken?

- ☒ English
- ☒ Spanish
- ☐ French
- ☐ Hmong
- ☐ Somali
- ☐ Japanese
- ☐ Chinese
- ☐ Russian
- ☐ Ethiopian
- ☐ Other ___________

l. Are you in the Global Health Pathway?

- ○ Yes
- ⊗ No

m. Did you earn your degree in the US?

- ⊗ Yes
- ○ No

n. What residency program are you in?

- ○ Internal Medicine
- ○ Med-Peds
- ○ Pediatrics
- ○ Family Practice
- ○ Neurology
- ○ Psychiatry
- ○ ObGyn
- ○ Neurosurgery
- ○ General Surgery
- ○ Orthopedic Surgery
- ○ Urology
- ○ Surgical sub-specialty (please specify in text box below)
- ⊗ Non-clinical specialty (radiology, pathology; please specify in text box below)

radiology

Medical Trainees' attitudes, knowledge, and experience with immigrant and refugee health

Response was added on 11/04/2013 8:10pm.

SECTION A: Personal experience with immigrant and refugee health care.

A. Please indicate your level of agreement with the following statements regarding your personal experience with immigrant and refugee health care by checking the box that best represents your experience.

a. During my inpatient rotations, I take care of the following percentage of immigrant and refugee patients:
- ◯ None
- ◯ 0 -5%
- ◯ 5-10%
- ⊗ 10-25%
- ◯ > 25%

b. During my outpatient rotations, I take care of the following percentage of immigrant and refugee patients:
- ◯ None
- ⊗ 0-10%
- ◯ 10 -25%
- ◯ 25-50%
- ◯ 50-75%
- ◯ >75%

c. I would like to take care of more immigrant and refugee patients.
- ◯ Strongly disagree
- ⊗ Disagree
- ◯ No opinion
- ◯ Agree
- ◯ Strongly agree

d. I plan to take care of immigrants and refugees when I finish residency.
- ◯ Strongly disagree
- ◯ Disagree
- ◯ No opinion
- ⊗ Agree
- ◯ Strongly agree

e. I plan to do short term (< 6 months) international work when I finish residency.
- ◯ Strongly disagree
- ◯ Disagree
- ◯ No opinion
- ⊗ Agree
- ◯ Strongly agree

f. I plan to do long term (>6 months) international work when I finish residency.
- ◯ Strongly disagree
- ◯ Disagree
- ◯ No opinion
- ⊗ Agree
- ◯ Strongly agree

g. I plan to work in health disparities in the following way after residency:

I would do abroad work

SECTION B: MEDICAL EDUCATION

A. Please indicate your level of agreement with the following statements regarding your medical education and knowledge about immigrants and refugees by checking the box that best represents your opinion.

a. I have received specialized training in immigrant and refugee health, tropical medicine, or cross-cultural health.

◯ Strongly disagree
◯ Disagree
◯ No opinion
⊗ Agree
◯ Strongly agree

b. If you have received specialized training in immigrant and refugee health, tropical medicine, or cross-cultural health, please indicate all the contexts in which you received this training:

☐ As an undergraduate.
☒ As a medical student.
☒ As part of my residency.
☐ A special program.
☐ As part of my fellowship.
☐ As part of a degree program (e.g. MPH)
☐ Other:

c. I feel comfortable with my fund of knowledge regarding immigrant and refugee health.

◯ Strongly disagree
⊗ Disagree
◯ No opinion
◯ Agree
◯ Strongly agree

d. I would like to have further training in immigrant and refugee health.

◯ Strongly disagree
◯ Disagree
◯ No opinion
⊗ Agree
◯ Strongly agree

e. If you agree with the above, please indicate all the contexts in which you would like to receive this training:
□□

☒ As part of my residency.
☐ A special program.
☐ As part of my fellowship.

SECTION C: Attitudes towards immigrant health

A.□Please indicate your level of agreement with the following statements regarding immigrant and refugee health by checking the box that best represents your opinion.

a. I enjoy taking care of immigrants and refugees.

◯ Never
◯ Rarely
⊗ Sometimes
◯ Usually
◯ Always

b. Please indicate the reasons that you enjoy taking care of immigrants and refugees (may choose more than one).

☒ Tropical and other conditions not frequently diagnosed in US-born patients
☒ Learning about other cultures
☐ They don't complain as much
☐ Being able to hear their stories
☐ Their care is more complicated
☐ Their care is less complicated
☐ They are very appreciative of your help.
☐ They are extremely vulnerable
☐ Other:

c. Taking care of immigrants and refugees is more challenging than taking care of US born patients.

◯ Never
◯ Rarely
⊗ Sometimes
◯ Usually
◯ Always

d. Please mark all the challenges that you face as a provider when providing care to immigrants and refugees (may choose more than one):

- ☒ Language barriers
- ☒ Insurance barriers
- ☒ Cultural barriers
- ☒ Finding a professional interpreter
- ☐ Knowing how to work with a professional interpreter
- ☒ Time constraints
- ☒ My own knowledge related to tropical and travel medicine
- ☐ Transportation problems for the patient
- ☐ Patients not understanding treatment plan
- ☐ Patients not following treatment plan
- ☐ My lack of knowledge regarding the patient's culture
- ☐ Bias or stereotyping
- ☐ Other:

e. Please mark all of the challenges faced by immigrant and refugee populations when receiving healthcare that you have perceived or witnessed (may choose more than one):

- ☒ Language barriers
- ☒ Insurance barriers
- ☒ Cultural barriers
- ☐ Finding a professional interpreter
- ☐ Knowing how to work with a professional interpreter
- ☐ Time constraints
- ☐ Insufficiently trained health care providers
- ☒ Transportation problems for the patient
- ☐ Food insufficiency
- ☐ Need for child care
- ☒ Patients not understanding treatment plan
- ☒ Patients not following treatment plan
- ☐ My lack of knowledge regarding the patient's culture
- ☐ Bias or stereotyping
- ☐ Trust issues
- ☐ Other...

f. Rank how well immigrants and refugees understand the healthcare that you are trying to provide.

- ◯ Significantly less than a US born individual
- ⊗ Less than a US born individual
- ◯ Equivalent to a US born individual
- ◯ More than a US born patient
- ◯ Significantly more than a US born individual

g. Immigrants and refugees adhere to treatment plans and follow my recommendations.

- ◯ Never
- ◯ Rarely
- ◯ Sometimes
- ⊗ Usually
- ◯ Always

h. Immigrants and refugees should receive the same care and insurance coverage as US born patients.

- ◯ Never
- ◯ Rarely
- ⊗ Sometimes
- ◯ Usually
- ◯ Always

i. Immigrants and refugees who are undocumented should receive the same care and insurance coverage as US born patients.

- ◯ Never
- ⊗ Rarely
- ◯ Sometimes
- ◯ Usually
- ◯ Always

j. Every physician is professionally obligated to care for immigrants and refugees if they present to your clinic or hospital.

- ◯ Strongly disagree
- ◯ Disagree
- ◯ No opinion
- ⊗ Agree
- ◯ Strongly agree

k. Is healthcare a human right?

⊗ Yes
◯ No

B. If you wish, please tell us about what you enjoy or do not enjoy about immigrant and refugee health care and the greatest challenges you face in caring for this population.

____

SECTION D: DEMOGRAPHIC INFORMATION
Please answer the following questions by checking the box in front of the response choice that best describes you.

a. Your age?

◯ 20 to 24
⊗ 25 to 29
◯ 30 to 34
◯ 35 to 39
◯ 40 or older

b. Your gender?

⊗ Female
◯ Male
◯ Other

c. Are you Hispanic or Latino?

◯ Yes
⊗ No

d. What is your race? (Select one or more responses)

◯ American Indian or Alaska Native
◯ Asian (Please specify):
◯ Black or African American
◯ Native Hawaiian or Other Pacific Islander
⊗ White
◯ Other (Please specify):

e. Were you born in the United States?

⊗ Yes
◯ No

g. Your residency year?

◯ PGY1
⊗ PGY2
◯ PGY3
◯ PGY4
◯ PGY5

h. How would you classify your political ideology?

◯ Conservative
◯ Somewhat conservative
⊗ Moderate
◯ Somewhat liberal
◯ Liberal
◯ Other (Please specify):

i. Estimated level of educational debt?

◯ None
◯ Less than $50,000
◯ $50,000 - $100,000
◯ $100,000 - $200,000
⊗ $200,000 or more

j. Do you plan to subspecialize?

⊗ Yes
◯ No

k. Languages spoken?

- [x] English
- [ ] Spanish
- [ ] French
- [ ] Hmong
- [ ] Somali
- [ ] Japanese
- [ ] Chinese
- [ ] Russian
- [ ] Ethiopian
- [ ] Other ___________

l. Are you in the Global Health Pathway?

- (X) Yes
- ( ) No

m. Did you earn your degree in the US?

- ( ) Yes
- ( ) No

n. What residency program are you in?

- ( ) Internal Medicine
- ( ) Med-Peds
- (X) Pediatrics
- ( ) Family Practice
- ( ) Neurology
- ( ) Psychiatry
- ( ) ObGyn
- ( ) Neurosurgery
- ( ) General Surgery
- ( ) Orthopedic Surgery
- ( ) Urology
- ( ) Surgical sub-specialty (please specify in text box below)
- ( ) Non-clinical specialty (radiology, pathology; please specify in text box below)

Medical Trainees' attitudes, knowledge, and experience with immigrant and refugee health

Response was added on 11/04/2013 8:36pm.

SECTION A: Personal experience with immigrant and refugee health care.

A. Please indicate your level of agreement with the following statements regarding your personal experience with immigrant and refugee health care by checking the box that best represents your experience.

a. During my inpatient rotations, I take care of the following percentage of immigrant and refugee patients:

- ◯ None
- ◯ 0 -5%
- ⊗ 5-10%
- ◯ 10-25%
- ◯ > 25%

b. During my outpatient rotations, I take care of the following percentage of immigrant and refugee patients:

- ◯ None
- ◯ 0-10%
- ◯ 10 -25%
- ⊗ 25-50%
- ◯ 50-75%
- ◯ >75%

c. I would like to take care of more immigrant and refugee patients.

- ◯ Strongly disagree
- ◯ Disagree
- ◯ No opinion
- ⊗ Agree
- ◯ Strongly agree

d. I plan to take care of immigrants and refugees when I finish residency.

- ◯ Strongly disagree
- ◯ Disagree
- ◯ No opinion
- ◯ Agree
- ⊗ Strongly agree

e. I plan to do short term (< 6 months) international work when I finish residency.

- ◯ Strongly disagree
- ◯ Disagree
- ◯ No opinion
- ◯ Agree
- ⊗ Strongly agree

f. I plan to do long term (>6 months) international work when I finish residency.

- ◯ Strongly disagree
- ◯ Disagree
- ◯ No opinion
- ◯ Agree
- ⊗ Strongly agree

g. I plan to work in health disparities in the following way after residency:

Clinic with immigrant/refugee population and work abroad for > 1year.

SECTION B: MEDICAL EDUCATION

A. Please indicate your level of agreement with the following statements regarding your medical education and knowledge about immigrants and refugees by checking the box that best represents your opinion.

a. I have received specialized training in immigrant and refugee health, tropical medicine, or cross-cultural health.

- ◯ Strongly disagree
- ◯ Disagree
- ◯ No opinion
- ◯ Agree
- ⊗ Strongly agree

b. If you have received specialized training in immigrant and refugee health, tropical medicine, or cross-cultural health, please indicate all the contexts in which you received this training:

- ☐ As an undergraduate.
- ☐ As a medical student.
- ☒ As part of my residency.
- ☐ A special program.
- ☐ As part of my fellowship.
- ☐ As part of a degree program (e.g. MPH)
- ☐ Other:

c. I feel comfortable with my fund of knowledge regarding immigrant and refugee health.

- ◯ Strongly disagree
- ◯ Disagree
- ◯ No opinion
- ⊗ Agree
- ◯ Strongly agree

d. I would like to have further training in immigrant and refugee health.

- ◯ Strongly disagree
- ⊗ Disagree
- ◯ No opinion
- ◯ Agree
- ◯ Strongly agree

SECTION C: Attitudes towards immigrant health

A.Please indicate your level of agreement with the following statements regarding immigrant and refugee health by checking the box that best represents your opinion.

a. I enjoy taking care of immigrants and refugees.

- ◯ Never
- ◯ Rarely
- ◯ Sometimes
- ⊗ Usually
- ◯ Always

b. Please indicate the reasons that you enjoy taking care of immigrants and refugees (may choose more than one).

- ☒ Tropical and other conditions not frequently diagnosed in US-born patients
- ☒ Learning about other cultures
- ☐ They don't complain as much
- ☒ Being able to hear their stories
- ☐ Their care is more complicated
- ☐ Their care is less complicated
- ☐ They are very appreciative of your help.
- ☒ They are extremely vulnerable
- ☐ Other:

c. Taking care of immigrants and refugees is more challenging than taking care of US born patients.

- ◯ Never
- ◯ Rarely
- ⊗ Sometimes
- ◯ Usually
- ◯ Always

d. Please mark all the challenges that you face as a provider when providing care to immigrants and refugees (may choose more than one):

- ☒ Language barriers
- ☒ Insurance barriers
- ☒ Cultural barriers
- ☒ Finding a professional interpreter
- ☐ Knowing how to work with a professional interpreter
- ☒ Time constraints
- ☒ My own knowledge related to tropical and travel medicine
- ☒ Transportation problems for the patient
- ☒ Patients not understanding treatment plan
- ☐ Patients not following treatment plan
- ☐ My lack of knowledge regarding the patient's culture
- ☐ Bias or stereotyping
- ☐ Other:

e. Please mark all of the challenges faced by immigrant and refugee populations when receiving healthcare that you have perceived or witnessed (may choose more than one):

- ☒ Language barriers
- ☒ Insurance barriers
- ☒ Cultural barriers
- ☒ Finding a professional interpreter
- ☒ Knowing how to work with a professional interpreter
- ☒ Time constraints
- ☐ Insufficiently trained health care providers
- ☒ Transportation problems for the patient
- ☒ Food insufficiency
- ☐ Need for child care
- ☒ Patients not understanding treatment plan
- ☒ Patients not following treatment plan
- ☐ My lack of knowledge regarding the patient's culture
- ☐ Bias or stereotyping
- ☒ Trust issues
- ☐ Other...

f. Rank how well immigrants and refugees understand the healthcare that you are trying to provide.

- ◯ Significantly less than a US born individual
- ◯ Less than a US born individual
- ⊗ Equivalent to a US born individual
- ◯ More than a US born patient
- ◯ Significantly more than a US born individual

g. Immigrants and refugees adhere to treatment plans and follow my recommendations.

- ◯ Never
- ◯ Rarely
- ◯ Sometimes
- ⊗ Usually
- ◯ Always

h. Immigrants and refugees should receive the same care and insurance coverage as US born patients.

- ◯ Never
- ◯ Rarely
- ◯ Sometimes
- ◯ Usually
- ⊗ Always

i. Immigrants and refugees who are undocumented should receive the same care and insurance coverage as US born patients.

- ◯ Never
- ◯ Rarely
- ⊗ Sometimes
- ◯ Usually
- ◯ Always

j. Every physician is professionally obligated to care for immigrants and refugees if they present to your clinic or hospital.

- ◯ Strongly disagree
- ◯ Disagree
- ◯ No opinion
- ◯ Agree
- ⊗ Strongly agree

k. Is healthcare a human right?
- ⊗ Yes
- ◯ No

B. If you wish, please tell us about what you enjoy or do not enjoy about immigrant and refugee health care and the greatest challenges you face in caring for this population.

____

SECTION D: DEMOGRAPHIC INFORMATION
Please answer the following questions by checking the box in front of the response choice that best describes you.

a. Your age?
- ◯ 20 to 24
- ◯ 25 to 29
- ⊗ 30 to 34
- ◯ 35 to 39
- ◯ 40 or older

b. Your gender?
- ⊗ Female
- ◯ Male
- ◯ Other

c. Are you Hispanic or Latino?
- ◯ Yes
- ⊗ No

d. What is your race? (Select one or more responses)
- ◯ American Indian or Alaska Native
- ◯ Asian (Please specify):
- ◯ Black or African American
- ◯ Native Hawaiian or Other Pacific Islander
- ⊗ White
- ◯ Other (Please specify):

e. Were you born in the United States?
- ⊗ Yes
- ◯ No

g. Your residency year?
- ◯ PGY1
- ◯ PGY2
- ◯ PGY3
- ⊗ PGY4
- ◯ PGY5

h. How would you classify your political ideology?
- ◯ Conservative
- ◯ Somewhat conservative
- ⊗ Moderate
- ◯ Somewhat liberal
- ◯ Liberal
- ◯ Other (Please specify):

i. Estimated level of educational debt?
- ◯ None
- ◯ Less than $50,000
- ◯ $50,000 - $100,000
- ◯ $100,000 - $200,000
- ⊗ $200,000 or more

j. Do you plan to subspecialize?
- ◯ Yes
- ⊗ No

k. Languages spoken?

- [x] English
- [x] Spanish
- [ ] French
- [ ] Hmong
- [ ] Somali
- [ ] Japanese
- [ ] Chinese
- [ ] Russian
- [ ] Ethiopian
- [ ] Other ___________

l. Are you in the Global Health Pathway?

- ⊗ Yes
- ◯ No

m. Did you earn your degree in the US?

- ⊗ Yes
- ◯ No

n. What residency program are you in?

- ◯ Internal Medicine
- ⊗ Med-Peds
- ◯ Pediatrics
- ◯ Family Practice
- ◯ Neurology
- ◯ Psychiatry
- ◯ ObGyn
- ◯ Neurosurgery
- ◯ General Surgery
- ◯ Orthopedic Surgery
- ◯ Urology
- ◯ Surgical sub-specialty (please specify in text box below)
- ◯ Non-clinical specialty (radiology, pathology; please specify in text box below)

Medical Trainees' attitudes, knowledge, and experience with immigrant and refugee health

Response was added on 11/05/2013 3:05am.

SECTION A: Personal experience with immigrant and refugee health care.

A. Please indicate your level of agreement with the following statements regarding your personal experience with immigrant and refugee health care by checking the box that best represents your experience.

a. During my inpatient rotations, I take care of the following percentage of immigrant and refugee patients:

- ◯ None
- ◯ 0 -5%
- ◯ 5-10%
- ⊗ 10-25%
- ◯ > 25%

b. During my outpatient rotations, I take care of the following percentage of immigrant and refugee patients:

- ◯ None
- ◯ 0-10%
- ⊗ 10 -25%
- ◯ 25-50%
- ◯ 50-75%
- ◯ >75%

c. I would like to take care of more immigrant and refugee patients.

- ◯ Strongly disagree
- ⊗ Disagree
- ◯ No opinion
- ◯ Agree
- ◯ Strongly agree

d. I plan to take care of immigrants and refugees when I finish residency.

- ◯ Strongly disagree
- ◯ Disagree
- ◯ No opinion
- ⊗ Agree
- ◯ Strongly agree

e. I plan to do short term (< 6 months) international work when I finish residency.

- ◯ Strongly disagree
- ◯ Disagree
- ◯ No opinion
- ⊗ Agree
- ◯ Strongly agree

f. I plan to do long term (>6 months) international work when I finish residency.

- ◯ Strongly disagree
- ◯ Disagree
- ⊗ No opinion
- ◯ Agree
- ◯ Strongly agree

g. I plan to work in health disparities in the following way after residency:

plan to continue to work with medicaid paitient, plan to continue to work with immigrant populations. likely will do volunteer or work abroad

SECTION B: MEDICAL EDUCATION

A. Please indicate your level of agreement with the following statements regarding your medical education and knowledge about immigrants and refugees by checking the box that best represents your opinion.

a. I have received specialized training in immigrant and refugee health, tropical medicine, or cross-cultural health.

- ◯ Strongly disagree
- ⊗ Disagree
- ◯ No opinion
- ◯ Agree
- ◯ Strongly agree

c. I feel comfortable with my fund of knowledge regarding immigrant and refugee health.

- ◯ Strongly disagree
- ◯ Disagree
- ⊗ No opinion
- ◯ Agree
- ◯ Strongly agree

d. I would like to have further training in immigrant and refugee health.

- ◯ Strongly disagree
- ◯ Disagree
- ◯ No opinion
- ◯ Agree
- ⊗ Strongly agree

e. If you agree with the above, please indicate all the contexts in which you would like to receive this training:

- ☒ As part of my residency.
- ☐ A special program.
- ☐ As part of my fellowship.

SECTION C: Attitudes towards immigrant health

A. Please indicate your level of agreement with the following statements regarding immigrant and refugee health by checking the box that best represents your opinion.

a. I enjoy taking care of immigrants and refugees.

- ◯ Never
- ◯ Rarely
- ⊗ Sometimes
- ◯ Usually
- ◯ Always

b. Please indicate the reasons that you enjoy taking care of immigrants and refugees (may choose more than one).

- ☒ Tropical and other conditions not frequently diagnosed in US-born patients
- ☒ Learning about other cultures
- ☐ They don't complain as much
- ☐ Being able to hear their stories
- ☒ Their care is more complicated
- ☐ Their care is less complicated
- ☒ They are very appreciative of your help.
- ☐ They are extremely vulnerable
- ☐ Other:

c. Taking care of immigrants and refugees is more challenging than taking care of US born patients.

- ◯ Never
- ◯ Rarely
- ◯ Sometimes
- ◯ Usually
- ⊗ Always

d. Please mark all the challenges that you face as a provider when providing care to immigrants and refugees (may choose more than one):

- ☒ Language barriers
- ☒ Insurance barriers
- ☒ Cultural barriers
- ☒ Finding a professional interpreter
- ☐ Knowing how to work with a professional interpreter
- ☒ Time constraints
- ☒ My own knowledge related to tropical and travel medicine
- ☒ Transportation problems for the patient
- ☒ Patients not understanding treatment plan
- ☒ Patients not following treatment plan
- ☒ My lack of knowledge regarding the patient's culture
- ☒ Bias or stereotyping
- ☐ Other:

e. Please mark all of the challenges faced by immigrant and refugee populations when receiving healthcare that you have perceived or witnessed (may choose more than one):

- ☐ Language barriers
- ☐ Insurance barriers
- ☐ Cultural barriers
- ☐ Finding a professional interpreter
- ☐ Knowing how to work with a professional interpreter
- ☐ Time constraints
- ☐ Insufficiently trained health care providers
- ☐ Transportation problems for the patient
- ☐ Food insufficiency
- ☐ Need for child care
- ☐ Patients not understanding treatment plan
- ☐ Patients not following treatment plan
- ☐ My lack of knowledge regarding the patient's culture
- ☐ Bias or stereotyping
- ☐ Trust issues
- ☐ Other...

f. Rank how well immigrants and refugees understand the healthcare that you are trying to provide.

- ○ Significantly less than a US born individual
- ○ Less than a US born individual
- ○ Equivalent to a US born individual
- ○ More than a US born patient
- ○ Significantly more than a US born individual

g. Immigrants and refugees adhere to treatment plans and follow my recommendations.

- ○ Never
- ○ Rarely
- ○ Sometimes
- ○ Usually
- ○ Always

h. Immigrants and refugees should receive the same care and insurance coverage as US born patients.

- ○ Never
- ○ Rarely
- ○ Sometimes
- ○ Usually
- ○ Always

i. Immigrants and refugees who are undocumented should receive the same care and insurance coverage as US born patients.

- ○ Never
- ○ Rarely
- ○ Sometimes
- ○ Usually
- ○ Always

j. Every physician is professionally obligated to care for immigrants and refugees if they present to your clinic or hospital.

- ○ Strongly disagree
- ○ Disagree
- ○ No opinion
- ○ Agree
- ○ Strongly agree

k. Is healthcare a human right?
◯ Yes
◯ No

B. If you wish, please tell us about what you enjoy or do not enjoy about immigrant and refugee health care and the greatest challenges you face in caring for this population. __________________________________

SECTION D: DEMOGRAPHIC INFORMATION
Please answer the following questions by checking the box in front of the response choice that best describes you.

a. Your age?
◯ 20 to 24
◯ 25 to 29
◯ 30 to 34
◯ 35 to 39
◯ 40 or older

b. Your gender?
◯ Female
◯ Male
◯ Other

c.Are you Hispanic or Latino?
◯ Yes
◯ No

d. What is your race? (Select one or more responses)
◯ American Indian or Alaska Native
◯ Asian (Please specify):
◯ Black or African American
◯ Native Hawaiian or Other Pacific Islander
◯ White
◯ Other (Please specify):

e.Were you born in the United States?
◯ Yes
◯ No

g. Your residency year?
◯ PGY1
◯ PGY2
◯ PGY3
◯ PGY4
◯ PGY5

h. How would you classify your political ideology?
◯ Conservative
◯ Somewhat conservative
◯ Moderate
◯ Somewhat liberal
◯ Liberal
◯ Other (Please specify):

i. Estimated level of educational debt?
◯ None
◯ Less than $50,000
◯ $50,000 - $100,000
◯ $100,000 - $200,000
◯ $200,000 or more

j.Do you plan to subspecialize?
◯ Yes
◯ No

k. Languages spoken?

- ☐ English
- ☐ Spanish
- ☐ French
- ☐ Hmong
- ☐ Somali
- ☐ Japanese
- ☐ Chinese
- ☐ Russian
- ☐ Ethiopian
- ☐ Other ___________

l. Are you in the Global Health Pathway?

- ◯ Yes
- ◯ No

m. Did you earn your degree in the US?

- ◯ Yes
- ◯ No

n. What residency program are you in?

- ◯ Internal Medicine
- ◯ Med-Peds
- ◯ Pediatrics
- ◯ Family Practice
- ◯ Neurology
- ◯ Psychiatry
- ◯ ObGyn
- ◯ Neurosurgery
- ◯ General Surgery
- ◯ Orthopedic Surgery
- ◯ Urology
- ◯ Surgical sub-specialty (please specify in text box below)
- ◯ Non-clinical specialty (radiology, pathology; please specify in text box below)

Medical Trainees' attitudes, knowledge, and experience with immigrant and refugee health

Response was added on 11/05/2013 3:10am.

SECTION A: Personal experience with immigrant and refugee health care.

A. Please indicate your level of agreement with the following statements regarding your personal experience with immigrant and refugee health care by checking the box that best represents your experience.

a. During my inpatient rotations, I take care of the following percentage of immigrant and refugee patients:

- ◯ None
- ◯ 0 -5%
- ◯ 5-10%
- ⊗ 10-25%
- ◯ > 25%

b. During my outpatient rotations, I take care of the following percentage of immigrant and refugee patients:

- ◯ None
- ◯ 0-10%
- ⊗ 10 -25%
- ◯ 25-50%
- ◯ 50-75%
- ◯ >75%

c. I would like to take care of more immigrant and refugee patients.

- ◯ Strongly disagree
- ⊗ Disagree
- ◯ No opinion
- ◯ Agree
- ◯ Strongly agree

d. I plan to take care of immigrants and refugees when I finish residency.

- ◯ Strongly disagree
- ◯ Disagree
- ◯ No opinion
- ⊗ Agree
- ◯ Strongly agree

e. I plan to do short term (< 6 months) international work when I finish residency.

- ◯ Strongly disagree
- ◯ Disagree
- ◯ No opinion
- ⊗ Agree
- ◯ Strongly agree

f. I plan to do long term (>6 months) international work when I finish residency.

- ◯ Strongly disagree
- ◯ Disagree
- ⊗ No opinion
- ◯ Agree
- ◯ Strongly agree

g. I plan to work in health disparities in the following way after residency:

medicaid and immigrants will be part of my practice

SECTION B: MEDICAL EDUCATION

A. Please indicate your level of agreement with the following statements regarding your medical education and knowledge about immigrants and refugees by checking the box that best represents your opinion.

a. I have received specialized training in immigrant and refugee health, tropical medicine, or cross-cultural health.

- ◯ Strongly disagree
- ⊗ Disagree
- ◯ No opinion
- ◯ Agree
- ◯ Strongly agree

c. I feel comfortable with my fund of knowledge regarding immigrant and refugee health.

- ◯ Strongly disagree
- ⊗ Disagree
- ◯ No opinion
- ◯ Agree
- ◯ Strongly agree

d. I would like to have further training in immigrant and refugee health.

- ◯ Strongly disagree
- ◯ Disagree
- ◯ No opinion
- ◯ Agree
- ⊗ Strongly agree

e. If you agree with the above, please indicate all the contexts in which you would like to receive this training:
□□

- ☒ As part of my residency.
- ☐ A special program.
- ☐ As part of my fellowship.

SECTION C: Attitudes towards immigrant health

A.□Please indicate your level of agreement with the following statements regarding immigrant and refugee health by checking the box that best represents your opinion.

a. I enjoy taking care of immigrants and refugees.

- ◯ Never
- ◯ Rarely
- ⊗ Sometimes
- ◯ Usually
- ◯ Always

b. Please indicate the reasons that you enjoy taking care of immigrants and refugees (may choose more than one).

- ☒ Tropical and other conditions not frequently diagnosed in US-born patients
- ☒ Learning about other cultures
- ☐ They don't complain as much
- ☐ Being able to hear their stories
- ☒ Their care is more complicated
- ☐ Their care is less complicated
- ☐ They are very appreciative of your help.
- ☐ They are extremely vulnerable
- ☐ Other:

c. Taking care of immigrants and refugees is more challenging than taking care of US born patients.

- ◯ Never
- ◯ Rarely
- ◯ Sometimes
- ◯ Usually
- ⊗ Always

d. Please mark all the challenges that you face as a provider when providing care to immigrants and refugees (may choose more than one):

- ☒ Language barriers
- ☒ Insurance barriers
- ☒ Cultural barriers
- ☒ Finding a professional interpreter
- ☐ Knowing how to work with a professional interpreter
- ☒ Time constraints
- ☒ My own knowledge related to tropical and travel medicine
- ☒ Transportation problems for the patient
- ☒ Patients not understanding treatment plan
- ☒ Patients not following treatment plan
- ☒ My lack of knowledge regarding the patient's culture
- ☒ Bias or stereotyping
- ☐ Other:

e. Please mark all of the challenges faced by immigrant and refugee populations when receiving healthcare that you have perceived or witnessed (may choose more than one):

- ☒ Language barriers
- ☒ Insurance barriers
- ☒ Cultural barriers
- ☒ Finding a professional interpreter
- ☒ Knowing how to work with a professional interpreter
- ☒ Time constraints
- ☒ Insufficiently trained health care providers
- ☒ Transportation problems for the patient
- ☒ Food insufficiency
- ☒ Need for child care
- ☒ Patients not understanding treatment plan
- ☒ Patients not following treatment plan
- ☒ My lack of knowledge regarding the patient's culture
- ☒ Bias or stereotyping
- ☒ Trust issues
- ☐ Other...

f. Rank how well immigrants and refugees understand the healthcare that you are trying to provide.

- ⊗ Significantly less than a US born individual
- ○ Less than a US born individual
- ○ Equivalent to a US born individual
- ○ More than a US born patient
- ○ Significantly more than a US born individual

g. Immigrants and refugees adhere to treatment plans and follow my recommendations.

- ○ Never
- ○ Rarely
- ⊗ Sometimes
- ○ Usually
- ○ Always

h. Immigrants and refugees should receive the same care and insurance coverage as US born patients.

- ○ Never
- ○ Rarely
- ○ Sometimes
- ⊗ Usually
- ○ Always

i. Immigrants and refugees who are undocumented should receive the same care and insurance coverage as US born patients.

- ○ Never
- ○ Rarely
- ○ Sometimes
- ⊗ Usually
- ○ Always

j. Every physician is professionally obligated to care for immigrants and refugees if they present to your clinic or hospital.

- ○ Strongly disagree
- ○ Disagree
- ○ No opinion
- ⊗ Agree
- ○ Strongly agree

k. Is healthcare a human right?
- ⊗ Yes
- ◯ No

B. If you wish, please tell us about what you enjoy or do not enjoy about immigrant and refugee health care and the greatest challenges you face in caring for this population.

as checked in section C #d

SECTION D: DEMOGRAPHIC INFORMATION
Please answer the following questions by checking the box in front of the response choice that best describes you.

a. Your age?
- ◯ 20 to 24
- ◯ 25 to 29
- ⊗ 30 to 34
- ◯ 35 to 39
- ◯ 40 or older

b. Your gender?
- ⊗ Female
- ◯ Male
- ◯ Other

c.Are you Hispanic or Latino?
- ◯ Yes
- ⊗ No

d. What is your race? (Select one or more responses)
- ◯ American Indian or Alaska Native
- ◯ Asian (Please specify):
- ◯ Black or African American
- ◯ Native Hawaiian or Other Pacific Islander
- ⊗ White
- ◯ Other (Please specify):

e.Were you born in the United States?
- ⊗ Yes
- ◯ No

g. Your residency year?
- ◯ PGY1
- ◯ PGY2
- ⊗ PGY3
- ◯ PGY4
- ◯ PGY5

h. How would you classify your political ideology?
- ◯ Conservative
- ◯ Somewhat conservative
- ◯ Moderate
- ◯ Somewhat liberal
- ⊗ Liberal
- ◯ Other (Please specify):

i. Estimated level of educational debt?
- ◯ None
- ◯ Less than $50,000
- ◯ $50,000 - $100,000
- ◯ $100,000 - $200,000
- ⊗ $200,000 or more

j.Do you plan to subspecialize?
- ◯ Yes
- ⊗ No

k. Languages spoken?

- ☒ English
- ☐ Spanish
- ☐ French
- ☐ Hmong
- ☐ Somali
- ☐ Japanese
- ☐ Chinese
- ☐ Russian
- ☐ Ethiopian
- ☐ Other ___________

l. Are you in the Global Health Pathway?

- ◯ Yes
- ⊗ No

m. Did you earn your degree in the US?

- ⊗ Yes
- ◯ No

n. What residency program are you in?

- ◯ Internal Medicine
- ◯ Med-Peds
- ◯ Pediatrics
- ◯ Family Practice
- ◯ Neurology
- ◯ Psychiatry
- ⊗ ObGyn
- ◯ Neurosurgery
- ◯ General Surgery
- ◯ Orthopedic Surgery
- ◯ Urology
- ◯ Surgical sub-specialty (please specify in text box below)
- ◯ Non-clinical specialty (radiology, pathology; please specify in text box below)

Medical Trainees' attitudes, knowledge, and experience with immigrant and refugee health

Response was added on 11/05/2013 12:23pm.

SECTION A: Personal experience with immigrant and refugee health care.

A. Please indicate your level of agreement with the following statements regarding your personal experience with immigrant and refugee health care by checking the box that best represents your experience.

a. During my inpatient rotations, I take care of the following percentage of immigrant and refugee patients:

- ◯ None
- ◯ 0 -5%
- ◯ 5-10%
- ⊗ 10-25%
- ◯ > 25%

b. During my outpatient rotations, I take care of the following percentage of immigrant and refugee patients:

- ◯ None
- ⊗ 0-10%
- ◯ 10 -25%
- ◯ 25-50%
- ◯ 50-75%
- ◯ >75%

c. I would like to take care of more immigrant and refugee patients.

- ◯ Strongly disagree
- ⊗ Disagree
- ◯ No opinion
- ◯ Agree
- ◯ Strongly agree

d. I plan to take care of immigrants and refugees when I finish residency.

- ◯ Strongly disagree
- ◯ Disagree
- ⊗ No opinion
- ◯ Agree
- ◯ Strongly agree

e. I plan to do short term (< 6 months) international work when I finish residency.

- ⊗ Strongly disagree
- ◯ Disagree
- ◯ No opinion
- ◯ Agree
- ◯ Strongly agree

f. I plan to do long term (>6 months) international work when I finish residency.

- ⊗ Strongly disagree
- ◯ Disagree
- ◯ No opinion
- ◯ Agree
- ◯ Strongly agree

g. I plan to work in health disparities in the following way after residency:

No specific plans.

SECTION B: MEDICAL EDUCATION

A. Please indicate your level of agreement with the following statements regarding your medical education and knowledge about immigrants and refugees by checking the box that best represents your opinion.

a. I have received specialized training in immigrant and refugee health, tropical medicine, or cross-cultural health.

- ◯ Strongly disagree
- ◯ Disagree
- ◯ No opinion
- ⊗ Agree
- ◯ Strongly agree

b. If you have received specialized training in immigrant and refugee health, tropical medicine, or cross-cultural health, please indicate all the contexts in which you received this training:

- ☐ As an undergraduate.
- ☒ As a medical student.
- ☒ As part of my residency.
- ☐ A special program.
- ☐ As part of my fellowship.
- ☐ As part of a degree program (e.g. MPH)
- ☐ Other:

c. I feel comfortable with my fund of knowledge regarding immigrant and refugee health.

- ◯ Strongly disagree
- ⊗ Disagree
- ◯ No opinion
- ◯ Agree
- ◯ Strongly agree

d. I would like to have further training in immigrant and refugee health.

- ◯ Strongly disagree
- ◯ Disagree
- ⊗ No opinion
- ◯ Agree
- ◯ Strongly agree

SECTION C: Attitudes towards immigrant health

A.Please indicate your level of agreement with the following statements regarding immigrant and refugee health by checking the box that best represents your opinion.

a. I enjoy taking care of immigrants and refugees.

- ◯ Never
- ◯ Rarely
- ⊗ Sometimes
- ◯ Usually
- ◯ Always

b. Please indicate the reasons that you enjoy taking care of immigrants and refugees (may choose more than one).

- ☐ Tropical and other conditions not frequently diagnosed in US-born patients
- ☒ Learning about other cultures
- ☐ They don't complain as much
- ☒ Being able to hear their stories
- ☐ Their care is more complicated
- ☐ Their care is less complicated
- ☐ They are very appreciative of your help.
- ☒ They are extremely vulnerable
- ☐ Other:

c. Taking care of immigrants and refugees is more challenging than taking care of US born patients.

- ◯ Never
- ◯ Rarely
- ⊗ Sometimes
- ◯ Usually
- ◯ Always

d. Please mark all the challenges that you face as a provider when providing care to immigrants and refugees (may choose more than one):

- ☒ Language barriers
- ☐ Insurance barriers
- ☒ Cultural barriers
- ☒ Finding a professional interpreter
- ☐ Knowing how to work with a professional interpreter
- ☒ Time constraints
- ☒ My own knowledge related to tropical and travel medicine
- ☒ Transportation problems for the patient
- ☐ Patients not understanding treatment plan
- ☒ Patients not following treatment plan
- ☒ My lack of knowledge regarding the patient's culture
- ☐ Bias or stereotyping
- ☐ Other:

e. Please mark all of the challenges faced by immigrant and refugee populations when receiving healthcare that you have perceived or witnessed (may choose more than one):

- ☒ Language barriers
- ☒ Insurance barriers
- ☒ Cultural barriers
- ☒ Finding a professional interpreter
- ☒ Knowing how to work with a professional interpreter
- ☒ Time constraints
- ☒ Insufficiently trained health care providers
- ☒ Transportation problems for the patient
- ☒ Food insufficiency
- ☒ Need for child care
- ☒ Patients not understanding treatment plan
- ☒ Patients not following treatment plan
- ☒ My lack of knowledge regarding the patient's culture
- ☒ Bias or stereotyping
- ☒ Trust issues
- ☐ Other...

f. Rank how well immigrants and refugees understand the healthcare that you are trying to provide.

- ○ Significantly less than a US born individual
- ⊗ Less than a US born individual
- ○ Equivalent to a US born individual
- ○ More than a US born patient
- ○ Significantly more than a US born individual

g. Immigrants and refugees adhere to treatment plans and follow my recommendations.

- ○ Never
- ○ Rarely
- ⊗ Sometimes
- ○ Usually
- ○ Always

h. Immigrants and refugees should receive the same care and insurance coverage as US born patients.

- ○ Never
- ⊗ Rarely
- ○ Sometimes
- ○ Usually
- ○ Always

i. Immigrants and refugees who are undocumented should receive the same care and insurance coverage as US born patients.

- ○ Never
- ⊗ Rarely
- ○ Sometimes
- ○ Usually
- ○ Always

j. Every physician is professionally obligated to care for immigrants and refugees if they present to your clinic or hospital.

- ○ Strongly disagree
- ○ Disagree
- ○ No opinion
- ⊗ Agree
- ○ Strongly agree

k. Is healthcare a human right?
⊗ Yes
◯ No

B. If you wish, please tell us about what you enjoy or do not enjoy about immigrant and refugee health care and the greatest challenges you face in caring for this population.
____

SECTION D: DEMOGRAPHIC INFORMATION
Please answer the following questions by checking the box in front of the response choice that best describes you.

a. Your age?
◯ 20 to 24
◯ 25 to 29
⊗ 30 to 34
◯ 35 to 39
◯ 40 or older

b. Your gender?
⊗ Female
◯ Male
◯ Other

c. Are you Hispanic or Latino?
◯ Yes
⊗ No

d. What is your race? (Select one or more responses)
◯ American Indian or Alaska Native
◯ Asian (Please specify):
◯ Black or African American
◯ Native Hawaiian or Other Pacific Islander
⊗ White
◯ Other (Please specify):

e. Were you born in the United States?
⊗ Yes
◯ No

g. Your residency year?
◯ PGY1
◯ PGY2
⊗ PGY3
◯ PGY4
◯ PGY5

h. How would you classify your political ideology?
◯ Conservative
◯ Somewhat conservative
◯ Moderate
◯ Somewhat liberal
⊗ Liberal
◯ Other (Please specify):

i. Estimated level of educational debt?
◯ None
◯ Less than $50,000
◯ $50,000 - $100,000
◯ $100,000 - $200,000
⊗ $200,000 or more

j. Do you plan to subspecialize?
◯ Yes
⊗ No

k. Languages spoken?

- ☒ English
- ☐ Spanish
- ☐ French
- ☐ Hmong
- ☐ Somali
- ☐ Japanese
- ☐ Chinese
- ☐ Russian
- ☐ Ethiopian
- ☐ Other ___________

l. Are you in the Global Health Pathway?

- ◯ Yes
- ⊗ No

m. Did you earn your degree in the US?

- ⊗ Yes
- ◯ No

n. What residency program are you in?

- ◯ Internal Medicine
- ◯ Med-Peds
- ◯ Pediatrics
- ⊗ Family Practice
- ◯ Neurology
- ◯ Psychiatry
- ◯ ObGyn
- ◯ Neurosurgery
- ◯ General Surgery
- ◯ Orthopedic Surgery
- ◯ Urology
- ◯ Surgical sub-specialty (please specify in text box below)
- ◯ Non-clinical specialty (radiology, pathology; please specify in text box below)

Medical Trainees' attitudes, knowledge, and experience with immigrant and refugee health

Response was added on 11/05/2013 3:18pm.

SECTION A: Personal experience with immigrant and refugee health care.

A. Please indicate your level of agreement with the following statements regarding your personal experience with immigrant and refugee health care by checking the box that best represents your experience.

a. During my inpatient rotations, I take care of the following percentage of immigrant and refugee patients:
- ◯ None
- ◯ 0 -5%
- ◯ 5-10%
- ⊗ 10-25%
- ◯ > 25%

b. During my outpatient rotations, I take care of the following percentage of immigrant and refugee patients:
- ◯ None
- ◯ 0-10%
- ◯ 10 -25%
- ⊗ 25-50%
- ◯ 50-75%
- ◯ >75%

c. I would like to take care of more immigrant and refugee patients.
- ◯ Strongly disagree
- ◯ Disagree
- ◯ No opinion
- ◯ Agree
- ⊗ Strongly agree

d. I plan to take care of immigrants and refugees when I finish residency.
- ◯ Strongly disagree
- ◯ Disagree
- ◯ No opinion
- ◯ Agree
- ⊗ Strongly agree

e. I plan to do short term (< 6 months) international work when I finish residency.
- ◯ Strongly disagree
- ◯ Disagree
- ◯ No opinion
- ⊗ Agree
- ◯ Strongly agree

f. I plan to do long term (>6 months) international work when I finish residency.
- ◯ Strongly disagree
- ◯ Disagree
- ◯ No opinion
- ◯ Agree
- ⊗ Strongly agree

g. I plan to work in health disparities in the following way after residency:

Working with low income, at risk populations in the US and likely working internationally long term as well

SECTION B: MEDICAL EDUCATION

A. Please indicate your level of agreement with the following statements regarding your medical education and knowledge about immigrants and refugees by checking the box that best represents your opinion.

a. I have received specialized training in immigrant and refugee health, tropical medicine, or cross-cultural health.

◯ Strongly disagree
◯ Disagree
◯ No opinion
⊗ Agree
◯ Strongly agree

b. If you have received specialized training in immigrant and refugee health, tropical medicine, or cross-cultural health, please indicate all the contexts in which you received this training:

☒ As an undergraduate.
☒ As a medical student.
☒ As part of my residency.
☐ A special program.
☐ As part of my fellowship.
☐ As part of a degree program (e.g. MPH)
☐ Other:

c. I feel comfortable with my fund of knowledge regarding immigrant and refugee health.

◯ Strongly disagree
◯ Disagree
◯ No opinion
⊗ Agree
◯ Strongly agree

d. I would like to have further training in immigrant and refugee health.

◯ Strongly disagree
◯ Disagree
◯ No opinion
◯ Agree
⊗ Strongly agree

e. If you agree with the above, please indicate all the contexts in which you would like to receive this training:
□□

☒ As part of my residency.
☐ A special program.
☐ As part of my fellowship.

SECTION C: Attitudes towards immigrant health

A.□Please indicate your level of agreement with the following statements regarding immigrant and refugee health by checking the box that best represents your opinion.

a. I enjoy taking care of immigrants and refugees.

◯ Never
◯ Rarely
◯ Sometimes
⊗ Usually
◯ Always

b. Please indicate the reasons that you enjoy taking care of immigrants and refugees (may choose more than one).

☒ Tropical and other conditions not frequently diagnosed in US-born patients
☒ Learning about other cultures
☐ They don't complain as much
☒ Being able to hear their stories
☐ Their care is more complicated
☐ Their care is less complicated
☒ They are very appreciative of your help.
☒ They are extremely vulnerable
☐ Other:

c. Taking care of immigrants and refugees is more challenging than taking care of US born patients.

◯ Never
◯ Rarely
◯ Sometimes
⊗ Usually
◯ Always

d. Please mark all the challenges that you face as a provider when providing care to immigrants and refugees (may choose more than one):

- ☒ Language barriers
- ☒ Insurance barriers
- ☒ Cultural barriers
- ☐ Finding a professional interpreter
- ☐ Knowing how to work with a professional interpreter
- ☒ Time constraints
- ☐ My own knowledge related to tropical and travel medicine
- ☒ Transportation problems for the patient
- ☒ Patients not understanding treatment plan
- ☐ Patients not following treatment plan
- ☐ My lack of knowledge regarding the patient's culture
- ☒ Bias or stereotyping
- ☐ Other:

e. Please mark all of the challenges faced by immigrant and refugee populations when receiving healthcare that you have perceived or witnessed (may choose more than one):

- ☒ Language barriers
- ☒ Insurance barriers
- ☒ Cultural barriers
- ☒ Finding a professional interpreter
- ☒ Knowing how to work with a professional interpreter
- ☒ Time constraints
- ☒ Insufficiently trained health care providers
- ☒ Transportation problems for the patient
- ☒ Food insufficiency
- ☒ Need for child care
- ☒ Patients not understanding treatment plan
- ☒ Patients not following treatment plan
- ☒ My lack of knowledge regarding the patient's culture
- ☒ Bias or stereotyping
- ☒ Trust issues
- ☐ Other...

f. Rank how well immigrants and refugees understand the healthcare that you are trying to provide.

- ◯ Significantly less than a US born individual
- ◯ Less than a US born individual
- ⊗ Equivalent to a US born individual
- ◯ More than a US born patient
- ◯ Significantly more than a US born individual

g. Immigrants and refugees adhere to treatment plans and follow my recommendations.

- ◯ Never
- ◯ Rarely
- ⊗ Sometimes
- ◯ Usually
- ◯ Always

h. Immigrants and refugees should receive the same care and insurance coverage as US born patients.

- ◯ Never
- ◯ Rarely
- ◯ Sometimes
- ◯ Usually
- ⊗ Always

i. Immigrants and refugees who are undocumented should receive the same care and insurance coverage as US born patients.

- ◯ Never
- ◯ Rarely
- ◯ Sometimes
- ◯ Usually
- ⊗ Always

j. Every physician is professionally obligated to care for immigrants and refugees if they present to your clinic or hospital.

- ◯ Strongly disagree
- ◯ Disagree
- ◯ No opinion
- ◯ Agree
- ⊗ Strongly agree

k. Is healthcare a human right?
- ⊗ Yes
- ◯ No

B. If you wish, please tell us about what you enjoy or do not enjoy about immigrant and refugee health care and the greatest challenges you face in caring for this population. ______________________________

SECTION D: DEMOGRAPHIC INFORMATION
Please answer the following questions by checking the box in front of the response choice that best describes you.

a. Your age?
- ◯ 20 to 24
- ⊗ 25 to 29
- ◯ 30 to 34
- ◯ 35 to 39
- ◯ 40 or older

b. Your gender?
- ◯ Female
- ⊗ Male
- ◯ Other

c.Are you Hispanic or Latino?
- ◯ Yes
- ⊗ No

d. What is your race? (Select one or more responses)
- ◯ American Indian or Alaska Native
- ◯ Asian (Please specify):
- ◯ Black or African American
- ◯ Native Hawaiian or Other Pacific Islander
- ⊗ White
- ◯ Other (Please specify):

e.Were you born in the United States?
- ⊗ Yes
- ◯ No

g. Your residency year?
- ⊗ PGY1
- ◯ PGY2
- ◯ PGY3
- ◯ PGY4
- ◯ PGY5

h. How would you classify your political ideology?
- ◯ Conservative
- ◯ Somewhat conservative
- ◯ Moderate
- ◯ Somewhat liberal
- ⊗ Liberal
- ◯ Other (Please specify):

i. Estimated level of educational debt?
- ◯ None
- ◯ Less than $50,000
- ◯ $50,000 - $100,000
- ◯ $100,000 - $200,000
- ⊗ $200,000 or more

j.Do you plan to subspecialize?
- ◯ Yes
- ⊗ No

k. Languages spoken?

- ☒ English
- ☒ Spanish
- ☐ French
- ☐ Hmong
- ☐ Somali
- ☐ Japanese
- ☐ Chinese
- ☐ Russian
- ☐ Ethiopian
- ☒ Other ___________

Hebrew, Swahili

l. Are you in the Global Health Pathway?

- ⊗ Yes
- ◯ No

m. Did you earn your degree in the US?

- ⊗ Yes
- ◯ No

n. What residency program are you in?

- ◯ Internal Medicine
- ⊗ Med-Peds
- ◯ Pediatrics
- ◯ Family Practice
- ◯ Neurology
- ◯ Psychiatry
- ◯ ObGyn
- ◯ Neurosurgery
- ◯ General Surgery
- ◯ Orthopedic Surgery
- ◯ Urology
- ◯ Surgical sub-specialty (please specify in text box below)
- ◯ Non-clinical specialty (radiology, pathology; please specify in text box below)

Medical Trainees' attitudes, knowledge, and experience with immigrant and refugee health

Response was added on 11/07/2013 10:56am.

SECTION A: Personal experience with immigrant and refugee health care.

A. Please indicate your level of agreement with the following statements regarding your personal experience with immigrant and refugee health care by checking the box that best represents your experience.

a. During my inpatient rotations, I take care of the following percentage of immigrant and refugee patients:
- ◯ None
- ◯ 0 -5%
- ◯ 5-10%
- ⊗ 10-25%
- ◯ > 25%

b. During my outpatient rotations, I take care of the following percentage of immigrant and refugee patients:
- ◯ None
- ◯ 0-10%
- ◯ 10 -25%
- ◯ 25-50%
- ⊗ 50-75%
- ◯ >75%

c. I would like to take care of more immigrant and refugee patients.
- ◯ Strongly disagree
- ◯ Disagree
- ⊗ No opinion
- ◯ Agree
- ◯ Strongly agree

d. I plan to take care of immigrants and refugees when I finish residency.
- ◯ Strongly disagree
- ◯ Disagree
- ◯ No opinion
- ◯ Agree
- ⊗ Strongly agree

e. I plan to do short term (< 6 months) international work when I finish residency.
- ◯ Strongly disagree
- ◯ Disagree
- ◯ No opinion
- ◯ Agree
- ⊗ Strongly agree

f. I plan to do long term (>6 months) international work when I finish residency.
- ◯ Strongly disagree
- ◯ Disagree
- ◯ No opinion
- ◯ Agree
- ⊗ Strongly agree

g. I plan to work in health disparities in the following way after residency: yes

SECTION B: MEDICAL EDUCATION

A. Please indicate your level of agreement with the following statements regarding your medical education and knowledge about immigrants and refugees by checking the box that best represents your opinion.

a. I have received specialized training in immigrant and refugee health, tropical medicine, or cross-cultural health.

○ Strongly disagree
○ Disagree
○ No opinion
○ Agree
⊗ Strongly agree

b. If you have received specialized training in immigrant and refugee health, tropical medicine, or cross-cultural health, please indicate all the contexts in which you received this training:

☐ As an undergraduate.
☒ As a medical student.
☒ As part of my residency.
☒ A special program.
☐ As part of my fellowship.
☐ As part of a degree program (e.g. MPH)
☐ Other:

c. I feel comfortable with my fund of knowledge regarding immigrant and refugee health.

○ Strongly disagree
○ Disagree
○ No opinion
⊗ Agree
○ Strongly agree

d. I would like to have further training in immigrant and refugee health.

○ Strongly disagree
○ Disagree
○ No opinion
⊗ Agree
○ Strongly agree

e. If you agree with the above, please indicate all the contexts in which you would like to receive this training:
□□

☒ As part of my residency.
☒ A special program.
☐ As part of my fellowship.

SECTION C: Attitudes towards immigrant health

A.□Please indicate your level of agreement with the following statements regarding immigrant and refugee health by checking the box that best represents your opinion.

a. I enjoy taking care of immigrants and refugees.

○ Never
○ Rarely
○ Sometimes
⊗ Usually
○ Always

b. Please indicate the reasons that you enjoy taking care of immigrants and refugees (may choose more than one).

☒ Tropical and other conditions not frequently diagnosed in US-born patients
☒ Learning about other cultures
☒ They don't complain as much
☒ Being able to hear their stories
☒ Their care is more complicated
☒ Their care is less complicated
☒ They are very appreciative of your help.
☒ They are extremely vulnerable
☐ Other:

c. Taking care of immigrants and refugees is more challenging than taking care of US born patients.

○ Never
○ Rarely
○ Sometimes
⊗ Usually
○ Always

d. Please mark all the challenges that you face as a provider when providing care to immigrants and refugees (may choose more than one):

- ☒ Language barriers
- ☐ Insurance barriers
- ☒ Cultural barriers
- ☒ Finding a professional interpreter
- ☐ Knowing how to work with a professional interpreter
- ☒ Time constraints
- ☒ My own knowledge related to tropical and travel medicine
- ☒ Transportation problems for the patient
- ☒ Patients not understanding treatment plan
- ☒ Patients not following treatment plan
- ☒ My lack of knowledge regarding the patient's culture
- ☒ Bias or stereotyping
- ☐ Other:

e. Please mark all of the challenges faced by immigrant and refugee populations when receiving healthcare that you have perceived or witnessed (may choose more than one):

- ☒ Language barriers
- ☒ Insurance barriers
- ☒ Cultural barriers
- ☒ Finding a professional interpreter
- ☒ Knowing how to work with a professional interpreter
- ☒ Time constraints
- ☒ Insufficiently trained health care providers
- ☒ Transportation problems for the patient
- ☒ Food insufficiency
- ☒ Need for child care
- ☒ Patients not understanding treatment plan
- ☒ Patients not following treatment plan
- ☒ My lack of knowledge regarding the patient's culture
- ☒ Bias or stereotyping
- ☒ Trust issues
- ☐ Other...

f. Rank how well immigrants and refugees understand the healthcare that you are trying to provide.

- ○ Significantly less than a US born individual
- ⊗ Less than a US born individual
- ○ Equivalent to a US born individual
- ○ More than a US born patient
- ○ Significantly more than a US born individual

g. Immigrants and refugees adhere to treatment plans and follow my recommendations.

- ○ Never
- ○ Rarely
- ○ Sometimes
- ⊗ Usually
- ○ Always

h. Immigrants and refugees should receive the same care and insurance coverage as US born patients.

- ○ Never
- ○ Rarely
- ○ Sometimes
- ⊗ Usually
- ○ Always

i. Immigrants and refugees who are undocumented should receive the same care and insurance coverage as US born patients.

- ○ Never
- ○ Rarely
- ○ Sometimes
- ⊗ Usually
- ○ Always

j. Every physician is professionally obligated to care for immigrants and refugees if they present to your clinic or hospital.

- ○ Strongly disagree
- ○ Disagree
- ○ No opinion
- ○ Agree
- ⊗ Strongly agree

k. Is healthcare a human right?

⊗ Yes
◯ No

B. If you wish, please tell us about what you enjoy or do not enjoy about immigrant and refugee health care and the greatest challenges you face in caring for this population.

I love working with immigrants and refugees as they tend to take a more active role in their health care, working across cultures is fun and rewarding in the stories you get to learn and the interesting people. That being said, it simply requires more time to ensure that both the doc and patient understand each other. Frequently something is miss understood which can lead to poorer care. I've wittnessed just about every reason - didn't know how to access inurance, didn't know how to do refills, wanted to do a spiritual ceremony first and this then put them out of the window to safely operate, etc. If given additional time, working with refugees/immigrants is really rewarding, when expected to do a good job but not being given the time needed to do a good job, it is frustrating.

SECTION D: DEMOGRAPHIC INFORMATION
Please answer the following questions by checking the box in front of the response choice that best describes you.

a. Your age?

◯ 20 to 24
◯ 25 to 29
⊗ 30 to 34
◯ 35 to 39
◯ 40 or older

b. Your gender?

⊗ Female
◯ Male
◯ Other

c.Are you Hispanic or Latino?

◯ Yes
⊗ No

d. What is your race? (Select one or more responses)

◯ American Indian or Alaska Native
◯ Asian (Please specify):
◯ Black or African American
◯ Native Hawaiian or Other Pacific Islander
⊗ White
◯ Other (Please specify):

e.Were you born in the United States?

⊗ Yes
◯ No

g. Your residency year?

◯ PGY1
◯ PGY2
⊗ PGY3
◯ PGY4
◯ PGY5

h. How would you classify your political ideology?

◯ Conservative
◯ Somewhat conservative
◯ Moderate
⊗ Somewhat liberal
◯ Liberal
◯ Other (Please specify):

i. Estimated level of educational debt?

- ◯ None
- ◯ Less than $50,000
- ◯ $50,000 - $100,000
- ⊗ $100,000 - $200,000
- ◯ $200,000 or more

j.Do you plan to subspecialize?

- ◯ Yes
- ⊗ No

k. Languages spoken?

- ☒ English
- ☒ Spanish
- ☐ French
- ☐ Hmong
- ☐ Somali
- ☐ Japanese
- ☐ Chinese
- ☐ Russian
- ☐ Ethiopian
- ☒ Other ___________

German and Portuguese

l. Are you in the Global Health Pathway?

- ⊗ Yes
- ◯ No

m. Did you earn your degree in the US?

- ⊗ Yes
- ◯ No

n. What residency program are you in?

- ◯ Internal Medicine
- ⊗ Med-Peds
- ◯ Pediatrics
- ◯ Family Practice
- ◯ Neurology
- ◯ Psychiatry
- ◯ ObGyn
- ◯ Neurosurgery
- ◯ General Surgery
- ◯ Orthopedic Surgery
- ◯ Urology
- ◯ Surgical sub-specialty (please specify in text box below)
- ◯ Non-clinical specialty (radiology, pathology; please specify in text box below)

Medical Trainees' attitudes, knowledge, and experience with immigrant and refugee health

Response was added on 11/07/2013 12:25pm.

SECTION A: Personal experience with immigrant and refugee health care.

A. Please indicate your level of agreement with the following statements regarding your personal experience with immigrant and refugee health care by checking the box that best represents your experience.

a. During my inpatient rotations, I take care of the following percentage of immigrant and refugee patients:

- ○ None
- ⊗ 0 -5%
- ○ 5-10%
- ○ 10-25%
- ○ > 25%

b. During my outpatient rotations, I take care of the following percentage of immigrant and refugee patients:

- ○ None
- ○ 0-10%
- ○ 10 -25%
- ○ 25-50%
- ⊗ 50-75%
- ○ >75%

c. I would like to take care of more immigrant and refugee patients.

- ○ Strongly disagree
- ○ Disagree
- ○ No opinion
- ○ Agree
- ⊗ Strongly agree

d. I plan to take care of immigrants and refugees when I finish residency.

- ○ Strongly disagree
- ○ Disagree
- ○ No opinion
- ○ Agree
- ⊗ Strongly agree

e. I plan to do short term (< 6 months) international work when I finish residency.

- ○ Strongly disagree
- ○ Disagree
- ○ No opinion
- ⊗ Agree
- ○ Strongly agree

f. I plan to do long term (>6 months) international work when I finish residency.

- ○ Strongly disagree
- ○ Disagree
- ○ No opinion
- ○ Agree
- ⊗ Strongly agree

g. I plan to work in health disparities in the following way after residency:

Would like to be a physician in Indian Health Service or Peace Corps Global Health program in the short term, and long-term community health promotion work

SECTION B: MEDICAL EDUCATION

A. Please indicate your level of agreement with the following statements regarding your medical education and knowledge about immigrants and refugees by checking the box that best represents your opinion.

a. I have received specialized training in immigrant and refugee health, tropical medicine, or cross-cultural health.

- ○ Strongly disagree
- ○ Disagree
- ○ No opinion
- ○ Agree
- ⊗ Strongly agree

b. If you have received specialized training in immigrant and refugee health, tropical medicine, or cross-cultural health, please indicate all the contexts in which you received this training:

- ☒ As an undergraduate.
- ☒ As a medical student.
- ☒ As part of my residency.
- ☒ A special program.
- ☐ As part of my fellowship.
- ☐ As part of a degree program (e.g. MPH)
- ☐ Other:

c. I feel comfortable with my fund of knowledge regarding immigrant and refugee health.

- ○ Strongly disagree
- ○ Disagree
- ○ No opinion
- ⊗ Agree
- ○ Strongly agree

d. I would like to have further training in immigrant and refugee health.

- ○ Strongly disagree
- ○ Disagree
- ○ No opinion
- ○ Agree
- ⊗ Strongly agree

e. If you agree with the above, please indicate all the contexts in which you would like to receive this training:

- ☒ As part of my residency.
- ☒ A special program.
- ☒ As part of my fellowship.

SECTION C: Attitudes towards immigrant health

A. Please indicate your level of agreement with the following statements regarding immigrant and refugee health by checking the box that best represents your opinion.

a. I enjoy taking care of immigrants and refugees.

- ○ Never
- ○ Rarely
- ○ Sometimes
- ○ Usually
- ⊗ Always

b. Please indicate the reasons that you enjoy taking care of immigrants and refugees (may choose more than one).

- ☐ Tropical and other conditions not frequently diagnosed in US-born patients
- ☒ Learning about other cultures
- ☐ They don't complain as much
- ☒ Being able to hear their stories
- ☒ Their care is more complicated
- ☐ Their care is less complicated
- ☒ They are very appreciative of your help.
- ☒ They are extremely vulnerable
- ☐ Other:

c. Taking care of immigrants and refugees is more challenging than taking care of US born patients.

- ◯ Never
- ◯ Rarely
- ⊗ Sometimes
- ◯ Usually
- ◯ Always

d. Please mark all the challenges that you face as a provider when providing care to immigrants and refugees (may choose more than one):

- ☒ Language barriers
- ☒ Insurance barriers
- ☒ Cultural barriers
- ☒ Finding a professional interpreter
- ☐ Knowing how to work with a professional interpreter
- ☒ Time constraints
- ☐ My own knowledge related to tropical and travel medicine
- ☒ Transportation problems for the patient
- ☒ Patients not understanding treatment plan
- ☐ Patients not following treatment plan
- ☒ My lack of knowledge regarding the patient's culture
- ☒ Bias or stereotyping
- ☐ Other:

e. Please mark all of the challenges faced by immigrant and refugee populations when receiving healthcare that you have perceived or witnessed (may choose more than one):

- ☒ Language barriers
- ☒ Insurance barriers
- ☒ Cultural barriers
- ☒ Finding a professional interpreter
- ☒ Knowing how to work with a professional interpreter
- ☒ Time constraints
- ☒ Insufficiently trained health care providers
- ☒ Transportation problems for the patient
- ☒ Food insufficiency
- ☒ Need for child care
- ☒ Patients not understanding treatment plan
- ☒ Patients not following treatment plan
- ☒ My lack of knowledge regarding the patient's culture
- ☒ Bias or stereotyping
- ☒ Trust issues
- ☐ Other...

f. Rank how well immigrants and refugees understand the healthcare that you are trying to provide.

- ◯ Significantly less than a US born individual
- ⊗ Less than a US born individual
- ◯ Equivalent to a US born individual
- ◯ More than a US born patient
- ◯ Significantly more than a US born individual

g. Immigrants and refugees adhere to treatment plans and follow my recommendations.

- ◯ Never
- ◯ Rarely
- ◯ Sometimes
- ⊗ Usually
- ◯ Always

h. Immigrants and refugees should receive the same care and insurance coverage as US born patients.

- ◯ Never
- ◯ Rarely
- ◯ Sometimes
- ◯ Usually
- ⊗ Always

i. Immigrants and refugees who are undocumented should receive the same care and insurance coverage as US born patients.

- ◯ Never
- ◯ Rarely
- ◯ Sometimes
- ◯ Usually
- ⊗ Always

j. Every physician is professionally obligated to care for immigrants and refugees if they present to your clinic or hospital.

- ◯ Strongly disagree
- ◯ Disagree
- ◯ No opinion
- ◯ Agree
- ⊗ Strongly agree

k. Is healthcare a human right?

- ⊗ Yes
- ◯ No

B. If you wish, please tell us about what you enjoy or do not enjoy about immigrant and refugee health care and the greatest challenges you face in caring for this population.

____

SECTION D: DEMOGRAPHIC INFORMATION
Please answer the following questions by checking the box in front of the response choice that best describes you.

a. Your age?

- ◯ 20 to 24
- ◯ 25 to 29
- ⊗ 30 to 34
- ◯ 35 to 39
- ◯ 40 or older

b. Your gender?

- ⊗ Female
- ◯ Male
- ◯ Other

c.Are you Hispanic or Latino?

- ◯ Yes
- ⊗ No

d. What is your race? (Select one or more responses)

- ◯ American Indian or Alaska Native
- ◯ Asian (Please specify):
- ◯ Black or African American
- ◯ Native Hawaiian or Other Pacific Islander
- ⊗ White
- ◯ Other (Please specify):

e.Were you born in the United States?

- ⊗ Yes
- ◯ No

g. Your residency year?

- ◯ PGY1
- ◯ PGY2
- ⊗ PGY3
- ◯ PGY4
- ◯ PGY5

h. How would you classify your political ideology?

- ◯ Conservative
- ◯ Somewhat conservative
- ◯ Moderate
- ◯ Somewhat liberal
- ⊗ Liberal
- ◯ Other (Please specify):

i. Estimated level of educational debt?

- ◯ None
- ◯ Less than $50,000
- ◯ $50,000 - $100,000
- ◯ $100,000 - $200,000
- ⊗ $200,000 or more

j.Do you plan to subspecialize?

- ◯ Yes
- ⊗ No

k. Languages spoken?

- ☒ English
- ☒ Spanish
- ☐ French
- ☐ Hmong
- ☐ Somali
- ☐ Japanese
- ☐ Chinese
- ☐ Russian
- ☐ Ethiopian
- ☐ Other ___________

l. Are you in the Global Health Pathway?

- ⊗ Yes
- ◯ No

m. Did you earn your degree in the US?

- ⊗ Yes
- ◯ No

n. What residency program are you in?

- ◯ Internal Medicine
- ⊗ Med-Peds
- ◯ Pediatrics
- ◯ Family Practice
- ◯ Neurology
- ◯ Psychiatry
- ◯ ObGyn
- ◯ Neurosurgery
- ◯ General Surgery
- ◯ Orthopedic Surgery
- ◯ Urology
- ◯ Surgical sub-specialty (please specify in text box below)
- ◯ Non-clinical specialty (radiology, pathology; please specify in text box below)

Medical Trainees' attitudes, knowledge, and experience with immigrant and refugee health

Response was added on 11/08/2013 11:21am.

SECTION A: Personal experience with immigrant and refugee health care.

A. Please indicate your level of agreement with the following statements regarding your personal experience with immigrant and refugee health care by checking the box that best represents your experience.

a. During my inpatient rotations, I take care of the following percentage of immigrant and refugee patients:
- ◯ None
- ⊗ 0 -5%
- ◯ 5-10%
- ◯ 10-25%
- ◯ > 25%

b. During my outpatient rotations, I take care of the following percentage of immigrant and refugee patients:
- ◯ None
- ⊗ 0-10%
- ◯ 10 -25%
- ◯ 25-50%
- ◯ 50-75%
- ◯ >75%

c. I would like to take care of more immigrant and refugee patients.
- ◯ Strongly disagree
- ⊗ Disagree
- ◯ No opinion
- ◯ Agree
- ◯ Strongly agree

d. I plan to take care of immigrants and refugees when I finish residency.
- ◯ Strongly disagree
- ⊗ Disagree
- ◯ No opinion
- ◯ Agree
- ◯ Strongly agree

e. I plan to do short term (< 6 months) international work when I finish residency.
- ⊗ Strongly disagree
- ◯ Disagree
- ◯ No opinion
- ◯ Agree
- ◯ Strongly agree

f. I plan to do long term (>6 months) international work when I finish residency.
- ⊗ Strongly disagree
- ◯ Disagree
- ◯ No opinion
- ◯ Agree
- ◯ Strongly agree

g. I plan to work in health disparities in the following way after residency:
As deemed necessary by my employer(s).

SECTION B: MEDICAL EDUCATION

A. Please indicate your level of agreement with the following statements regarding your medical education and knowledge about immigrants and refugees by checking the box that best represents your opinion.

a. I have received specialized training in immigrant and refugee health, tropical medicine, or cross-cultural health.

- ◯ Strongly disagree
- ⊗ Disagree
- ◯ No opinion
- ◯ Agree
- ◯ Strongly agree

c. I feel comfortable with my fund of knowledge regarding immigrant and refugee health.

- ◯ Strongly disagree
- ⊗ Disagree
- ◯ No opinion
- ◯ Agree
- ◯ Strongly agree

d. I would like to have further training in immigrant and refugee health.

- ◯ Strongly disagree
- ◯ Disagree
- ⊗ No opinion
- ◯ Agree
- ◯ Strongly agree

SECTION C: Attitudes towards immigrant health

A.Please indicate your level of agreement with the following statements regarding immigrant and refugee health by checking the box that best represents your opinion.

a. I enjoy taking care of immigrants and refugees.

- ◯ Never
- ◯ Rarely
- ⊗ Sometimes
- ◯ Usually
- ◯ Always

b. Please indicate the reasons that you enjoy taking care of immigrants and refugees (may choose more than one).

- ☐ Tropical and other conditions not frequently diagnosed in US-born patients
- ☐ Learning about other cultures
- ☐ They don't complain as much
- ☒ Being able to hear their stories
- ☐ Their care is more complicated
- ☐ Their care is less complicated
- ☐ They are very appreciative of your help.
- ☐ They are extremely vulnerable
- ☐ Other:

c. Taking care of immigrants and refugees is more challenging than taking care of US born patients.

- ◯ Never
- ◯ Rarely
- ⊗ Sometimes
- ◯ Usually
- ◯ Always

d. Please mark all the challenges that you face as a provider when providing care to immigrants and refugees (may choose more than one):

- ☒ Language barriers
- ☒ Insurance barriers
- ☒ Cultural barriers
- ☒ Finding a professional interpreter
- ☐ Knowing how to work with a professional interpreter
- ☒ Time constraints
- ☒ My own knowledge related to tropical and travel medicine
- ☐ Transportation problems for the patient
- ☒ Patients not understanding treatment plan
- ☒ Patients not following treatment plan
- ☒ My lack of knowledge regarding the patient's culture
- ☐ Bias or stereotyping
- ☐ Other:

e. Please mark all of the challenges faced by immigrant and refugee populations when receiving healthcare that you have perceived or witnessed (may choose more than one):

☒ Language barriers
☒ Insurance barriers
☒ Cultural barriers
☒ Finding a professional interpreter
☒ Knowing how to work with a professional interpreter
☒ Time constraints
☐ Insufficiently trained health care providers
☒ Transportation problems for the patient
☐ Food insufficiency
☐ Need for child care
☒ Patients not understanding treatment plan
☒ Patients not following treatment plan
☐ My lack of knowledge regarding the patient's culture
☒ Bias or stereotyping
☒ Trust issues
☐ Other...

f. Rank how well immigrants and refugees understand the healthcare that you are trying to provide.

◯ Significantly less than a US born individual
⊗ Less than a US born individual
◯ Equivalent to a US born individual
◯ More than a US born patient
◯ Significantly more than a US born individual

g. Immigrants and refugees adhere to treatment plans and follow my recommendations.

◯ Never
◯ Rarely
◯ Sometimes
⊗ Usually
◯ Always

h. Immigrants and refugees should receive the same care and insurance coverage as US born patients.

◯ Never
◯ Rarely
⊗ Sometimes
◯ Usually
◯ Always

i. Immigrants and refugees who are undocumented should receive the same care and insurance coverage as US born patients.

◯ Never
◯ Rarely
⊗ Sometimes
◯ Usually
◯ Always

j. Every physician is professionally obligated to care for immigrants and refugees if they present to your clinic or hospital.

◯ Strongly disagree
⊗ Disagree
◯ No opinion
◯ Agree
◯ Strongly agree

k. Is healthcare a human right?

◯ Yes
⊗ No

B. If you wish, please tell us about what you enjoy or do not enjoy about immigrant and refugee health care and the greatest challenges you face in caring for this population.

____

SECTION D: DEMOGRAPHIC INFORMATION
Please answer the following questions by checking the box in front of the response choice that best describes you.

a. Your age?
- ◯ 20 to 24
- ⊗ 25 to 29
- ⊗ 30 to 34
- ◯ 35 to 39
- ◯ 40 or older

b. Your gender?
- ◯ Female
- ⊗ Male
- ◯ Other

c. Are you Hispanic or Latino?
- ◯ Yes
- ⊗ No

d. What is your race? (Select one or more responses)
- ◯ American Indian or Alaska Native
- ◯ Asian (Please specify):
- ◯ Black or African American
- ◯ Native Hawaiian or Other Pacific Islander
- ⊗ White
- ◯ Other (Please specify):

e. Were you born in the United States?
- ⊗ Yes
- ◯ No

g. Your residency year?
- ◯ PGY1
- ◯ PGY2
- ◯ PGY3
- ⊗ PGY4
- ◯ PGY5

h. How would you classify your political ideology?
- ⊗ Conservative
- ◯ Somewhat conservative
- ◯ Moderate
- ◯ Somewhat liberal
- ◯ Liberal
- ◯ Other (Please specify):

i. Estimated level of educational debt?
- ◯ None
- ◯ Less than $50,000
- ◯ $50,000 - $100,000
- ⊗ $100,000 - $200,000
- ◯ $200,000 or more

j. Do you plan to subspecialize?
- ⊗ Yes
- ◯ No

k. Languages spoken?
- ☒ English
- ☐ Spanish
- ☐ French
- ☐ Hmong
- ☐ Somali
- ☐ Japanese
- ☐ Chinese
- ☐ Russian
- ☐ Ethiopian
- ☐ Other ___________

l. Are you in the Global Health Pathway?
- ◯ Yes
- ⊗ No

m. Did you earn your degree in the US?

⊗ Yes
◯ No

n. What residency program are you in?

⊗ Internal Medicine
◯ Med-Peds
◯ Pediatrics
◯ Family Practice
◯ Neurology
◯ Psychiatry
◯ ObGyn
◯ Neurosurgery
◯ General Surgery
◯ Orthopedic Surgery
◯ Urology
◯ Surgical sub-specialty (please specify in text box below)
◯ Non-clinical specialty (radiology, pathology; please specify in text box below)

Medical Trainees' attitudes, knowledge, and experience with immigrant and refugee health

Response was added on 11/10/2013 11:45am.

SECTION A: Personal experience with immigrant and refugee health care.

A. Please indicate your level of agreement with the following statements regarding your personal experience with immigrant and refugee health care by checking the box that best represents your experience.

a. During my inpatient rotations, I take care of the following percentage of immigrant and refugee patients:
- ◯ None
- ◯ 0 -5%
- ⊗ 5-10%
- ◯ 10-25%
- ◯ > 25%

b. During my outpatient rotations, I take care of the following percentage of immigrant and refugee patients:
- ◯ None
- ◯ 0-10%
- ⊗ 10 -25%
- ◯ 25-50%
- ◯ 50-75%
- ◯ >75%

c. I would like to take care of more immigrant and refugee patients.
- ◯ Strongly disagree
- ◯ Disagree
- ◯ No opinion
- ◯ Agree
- ⊗ Strongly agree

d. I plan to take care of immigrants and refugees when I finish residency.
- ◯ Strongly disagree
- ◯ Disagree
- ◯ No opinion
- ◯ Agree
- ⊗ Strongly agree

e. I plan to do short term (< 6 months) international work when I finish residency.
- ◯ Strongly disagree
- ◯ Disagree
- ◯ No opinion
- ◯ Agree
- ⊗ Strongly agree

f. I plan to do long term (>6 months) international work when I finish residency.
- ◯ Strongly disagree
- ◯ Disagree
- ⊗ No opinion
- ◯ Agree
- ◯ Strongly agree

g. I plan to work in health disparities in the following way after residency: 10

SECTION B: MEDICAL EDUCATION

A. Please indicate your level of agreement with the following statements regarding your medical education and knowledge about immigrants and refugees by checking the box that best represents your opinion.

a. I have received specialized training in immigrant and refugee health, tropical medicine, or cross-cultural health.

◯ Strongly disagree
◯ Disagree
◯ No opinion
◯ Agree
⊗ Strongly agree

b. If you have received specialized training in immigrant and refugee health, tropical medicine, or cross-cultural health, please indicate all the contexts in which you received this training:

☐ As an undergraduate.
☒ As a medical student.
☐ As part of my residency.
☐ A special program.
☐ As part of my fellowship.
☐ As part of a degree program (e.g. MPH)
☐ Other:

c. I feel comfortable with my fund of knowledge regarding immigrant and refugee health.

◯ Strongly disagree
◯ Disagree
◯ No opinion
◯ Agree
⊗ Strongly agree

d. I would like to have further training in immigrant and refugee health.

◯ Strongly disagree
◯ Disagree
◯ No opinion
◯ Agree
⊗ Strongly agree

e. If you agree with the above, please indicate all the contexts in which you would like to receive this training:

☒ As part of my residency.
☐ A special program.
☐ As part of my fellowship.

SECTION C: Attitudes towards immigrant health

A. Please indicate your level of agreement with the following statements regarding immigrant and refugee health by checking the box that best represents your opinion.

a. I enjoy taking care of immigrants and refugees.

◯ Never
◯ Rarely
◯ Sometimes
◯ Usually
⊗ Always

b. Please indicate the reasons that you enjoy taking care of immigrants and refugees (may choose more than one).

☐ Tropical and other conditions not frequently diagnosed in US-born patients
☒ Learning about other cultures
☐ They don't complain as much
☒ Being able to hear their stories
☒ Their care is more complicated
☐ Their care is less complicated
☒ They are very appreciative of your help.
☒ They are extremely vulnerable
☒ Other:

I am an immigrant as well

c. Taking care of immigrants and refugees is more challenging than taking care of US born patients.

◯ Never
◯ Rarely
◯ Sometimes
⊗ Usually
◯ Always

d. Please mark all the challenges that you face as a provider when providing care to immigrants and refugees (may choose more than one):

- ☒ Language barriers
- ☒ Insurance barriers
- ☒ Cultural barriers
- ☐ Finding a professional interpreter
- ☐ Knowing how to work with a professional interpreter
- ☒ Time constraints
- ☒ My own knowledge related to tropical and travel medicine
- ☒ Transportation problems for the patient
- ☒ Patients not understanding treatment plan
- ☒ Patients not following treatment plan
- ☐ My lack of knowledge regarding the patient's culture
- ☐ Bias or stereotyping
- ☐ Other:

e. Please mark all of the challenges faced by immigrant and refugee populations when receiving healthcare that you have perceived or witnessed (may choose more than one):

- ☒ Language barriers
- ☒ Insurance barriers
- ☒ Cultural barriers
- ☒ Finding a professional interpreter
- ☒ Knowing how to work with a professional interpreter
- ☒ Time constraints
- ☒ Insufficiently trained health care providers
- ☒ Transportation problems for the patient
- ☒ Food insufficiency
- ☒ Need for child care
- ☒ Patients not understanding treatment plan
- ☒ Patients not following treatment plan
- ☒ My lack of knowledge regarding the patient's culture
- ☒ Bias or stereotyping
- ☒ Trust issues
- ☐ Other...

f. Rank how well immigrants and refugees understand the healthcare that you are trying to provide.

- ⊗ Significantly less than a US born individual
- ◯ Less than a US born individual
- ◯ Equivalent to a US born individual
- ◯ More than a US born patient
- ◯ Significantly more than a US born individual

g. Immigrants and refugees adhere to treatment plans and follow my recommendations.

- ◯ Never
- ◯ Rarely
- ⊗ Sometimes
- ◯ Usually
- ◯ Always

h. Immigrants and refugees should receive the same care and insurance coverage as US born patients.

- ◯ Never
- ◯ Rarely
- ◯ Sometimes
- ◯ Usually
- ⊗ Always

i. Immigrants and refugees who are undocumented should receive the same care and insurance coverage as US born patients.

- ◯ Never
- ◯ Rarely
- ◯ Sometimes
- ◯ Usually
- ⊗ Always

j. Every physician is professionally obligated to care for immigrants and refugees if they present to your clinic or hospital.

- ◯ Strongly disagree
- ◯ Disagree
- ◯ No opinion
- ◯ Agree
- ⊗ Strongly agree

k. Is healthcare a human right?

⊗ Yes
◯ No

B. If you wish, please tell us about what you enjoy or do not enjoy about immigrant and refugee health care and the greatest challenges you face in caring for this population.

____

SECTION D: DEMOGRAPHIC INFORMATION
Please answer the following questions by checking the box in front of the response choice that best describes you.

a. Your age?

◯ 20 to 24
◯ 25 to 29
◯ 30 to 34
◯ 35 to 39
⊗ 40 or older

b. Your gender?

◯ Female
⊗ Male
◯ Other

c. Are you Hispanic or Latino?

◯ Yes
⊗ No

d. What is your race? (Select one or more responses)

◯ American Indian or Alaska Native
⊗ Asian (Please specify):
◯ Black or African American
◯ Native Hawaiian or Other Pacific Islander
◯ White
◯ Other (Please specify):

e. Were you born in the United States?

◯ Yes
⊗ No

f. If not, in what country were you born?

India

g. Your residency year?

◯ PGY1
⊗ PGY2
◯ PGY3
◯ PGY4
◯ PGY5

h. How would you classify your political ideology?

◯ Conservative
◯ Somewhat conservative
◯ Moderate
◯ Somewhat liberal
⊗ Liberal
◯ Other (Please specify):

i. Estimated level of educational debt?

◯ None
⊗ Less than $50,000
◯ $50,000 - $100,000
◯ $100,000 - $200,000
◯ $200,000 or more

j. Do you plan to subspecialize?

◯ Yes
⊗ No

k. Languages spoken?

- ☒ English
- ☐ Spanish
- ☐ French
- ☐ Hmong
- ☐ Somali
- ☐ Japanese
- ☐ Chinese
- ☐ Russian
- ☐ Ethiopian
- ☐ Other ___________

l. Are you in the Global Health Pathway?

- ◯ Yes
- ⊗ No

m. Did you earn your degree in the US?

- ◯ Yes
- ⊗ No

n. What residency program are you in?

- ◯ Internal Medicine
- ◯ Med-Peds
- ◯ Pediatrics
- ⊗ Family Practice
- ◯ Neurology
- ◯ Psychiatry
- ◯ ObGyn
- ◯ Neurosurgery
- ◯ General Surgery
- ◯ Orthopedic Surgery
- ◯ Urology
- ◯ Surgical sub-specialty (please specify in text box below)
- ◯ Non-clinical specialty (radiology, pathology; please specify in text box below)

Medical Trainees' attitudes, knowledge, and experience with immigrant and refugee health

Response was added on 11/12/2013 10:10am.

SECTION A: Personal experience with immigrant and refugee health care.

A. Please indicate your level of agreement with the following statements regarding your personal experience with immigrant and refugee health care by checking the box that best represents your experience.

a. During my inpatient rotations, I take care of the following percentage of immigrant and refugee patients:
- ○ None
- ⊗ 0 -5%
- ○ 5-10%
- ○ 10-25%
- ○ > 25%

b. During my outpatient rotations, I take care of the following percentage of immigrant and refugee patients:
- ○ None
- ⊗ 0-10%
- ○ 10 -25%
- ○ 25-50%
- ○ 50-75%
- ○ >75%

c. I would like to take care of more immigrant and refugee patients.
- ○ Strongly disagree
- ○ Disagree
- ⊗ No opinion
- ○ Agree
- ○ Strongly agree

d. I plan to take care of immigrants and refugees when I finish residency.
- ○ Strongly disagree
- ○ Disagree
- ⊗ No opinion
- ○ Agree
- ○ Strongly agree

e. I plan to do short term (< 6 months) international work when I finish residency.
- ○ Strongly disagree
- ⊗ Disagree
- ○ No opinion
- ○ Agree
- ○ Strongly agree

f. I plan to do long term (>6 months) international work when I finish residency.
- ○ Strongly disagree
- ⊗ Disagree
- ○ No opinion
- ○ Agree
- ○ Strongly agree

g. I plan to work in health disparities in the following way after residency:

I have no idea

SECTION B: MEDICAL EDUCATION

A. Please indicate your level of agreement with the following statements regarding your medical education and knowledge about immigrants and refugees by checking the box that best represents your opinion.

a. I have received specialized training in immigrant and refugee health, tropical medicine, or cross-cultural health.

- ◯ Strongly disagree
- ◯ Disagree
- ◯ No opinion
- ⊗ Agree
- ◯ Strongly agree

b. If you have received specialized training in immigrant and refugee health, tropical medicine, or cross-cultural health, please indicate all the contexts in which you received this training:

- ☐ As an undergraduate.
- ☒ As a medical student.
- ☒ As part of my residency.
- ☐ A special program.
- ☐ As part of my fellowship.
- ☐ As part of a degree program (e.g. MPH)
- ☐ Other:

c. I feel comfortable with my fund of knowledge regarding immigrant and refugee health.

- ◯ Strongly disagree
- ◯ Disagree
- ◯ No opinion
- ⊗ Agree
- ◯ Strongly agree

d. I would like to have further training in immigrant and refugee health.

- ◯ Strongly disagree
- ⊗ Disagree
- ◯ No opinion
- ◯ Agree
- ◯ Strongly agree

SECTION C: Attitudes towards immigrant health

A.Please indicate your level of agreement with the following statements regarding immigrant and refugee health by checking the box that best represents your opinion.

a. I enjoy taking care of immigrants and refugees.

- ◯ Never
- ◯ Rarely
- ◯ Sometimes
- ⊗ Usually
- ◯ Always

b. Please indicate the reasons that you enjoy taking care of immigrants and refugees (may choose more than one).

- ☐ Tropical and other conditions not frequently diagnosed in US-born patients
- ☒ Learning about other cultures
- ☐ They don't complain as much
- ☐ Being able to hear their stories
- ☐ Their care is more complicated
- ☐ Their care is less complicated
- ☒ They are very appreciative of your help.
- ☐ They are extremely vulnerable
- ☐ Other:

c. Taking care of immigrants and refugees is more challenging than taking care of US born patients.

- ◯ Never
- ◯ Rarely
- ◯ Sometimes
- ⊗ Usually
- ◯ Always

d. Please mark all the challenges that you face as a provider when providing care to immigrants and refugees (may choose more than one):

- ☒ Language barriers
- ☒ Insurance barriers
- ☒ Cultural barriers
- ☐ Finding a professional interpreter
- ☐ Knowing how to work with a professional interpreter
- ☒ Time constraints
- ☐ My own knowledge related to tropical and travel medicine
- ☐ Transportation problems for the patient
- ☒ Patients not understanding treatment plan
- ☐ Patients not following treatment plan
- ☒ My lack of knowledge regarding the patient's culture
- ☐ Bias or stereotyping
- ☐ Other:

e. Please mark all of the challenges faced by immigrant and refugee populations when receiving healthcare that you have perceived or witnessed (may choose more than one):

- ☒ Language barriers
- ☒ Insurance barriers
- ☒ Cultural barriers
- ☐ Finding a professional interpreter
- ☐ Knowing how to work with a professional interpreter
- ☐ Time constraints
- ☐ Insufficiently trained health care providers
- ☒ Transportation problems for the patient
- ☐ Food insufficiency
- ☐ Need for child care
- ☒ Patients not understanding treatment plan
- ☒ Patients not following treatment plan
- ☒ My lack of knowledge regarding the patient's culture
- ☐ Bias or stereotyping
- ☒ Trust issues
- ☐ Other...

f. Rank how well immigrants and refugees understand the healthcare that you are trying to provide.

- ○ Significantly less than a US born individual
- ⊗ Less than a US born individual
- ○ Equivalent to a US born individual
- ○ More than a US born patient
- ○ Significantly more than a US born individual

g. Immigrants and refugees adhere to treatment plans and follow my recommendations.

- ○ Never
- ○ Rarely
- ⊗ Sometimes
- ○ Usually
- ○ Always

h. Immigrants and refugees should receive the same care and insurance coverage as US born patients.

- ○ Never
- ○ Rarely
- ○ Sometimes
- ○ Usually
- ⊗ Always

i. Immigrants and refugees who are undocumented should receive the same care and insurance coverage as US born patients.

- ○ Never
- ○ Rarely
- ○ Sometimes
- ○ Usually
- ⊗ Always

j. Every physician is professionally obligated to care for immigrants and refugees if they present to your clinic or hospital.

- ○ Strongly disagree
- ○ Disagree
- ○ No opinion
- ○ Agree
- ⊗ Strongly agree

k. Is healthcare a human right?
- ⊗ Yes
- ◯ No

B. If you wish, please tell us about what you enjoy or do not enjoy about immigrant and refugee health care and the greatest challenges you face in caring for this population.

____

SECTION D: DEMOGRAPHIC INFORMATION
Please answer the following questions by checking the box in front of the response choice that best describes you.

a. Your age?
- ◯ 20 to 24
- ◯ 25 to 29
- ◯ 30 to 34
- ◯ 35 to 39
- ⊗ 40 or older

b. Your gender?
- ◯ Female
- ⊗ Male
- ◯ Other

c. Are you Hispanic or Latino?
- ◯ Yes
- ⊗ No

d. What is your race? (Select one or more responses)
- ◯ American Indian or Alaska Native
- ◯ Asian (Please specify):
- ◯ Black or African American
- ◯ Native Hawaiian or Other Pacific Islander
- ⊗ White
- ◯ Other (Please specify):

e. Were you born in the United States?
- ⊗ Yes
- ◯ No

g. Your residency year?
- ◯ PGY1
- ◯ PGY2
- ◯ PGY3
- ⊗ PGY4
- ◯ PGY5

h. How would you classify your political ideology?
- ◯ Conservative
- ⊗ Somewhat conservative
- ◯ Moderate
- ◯ Somewhat liberal
- ◯ Liberal
- ◯ Other (Please specify):

i. Estimated level of educational debt?
- ◯ None
- ◯ Less than $50,000
- ◯ $50,000 - $100,000
- ⊗ $100,000 - $200,000
- ◯ $200,000 or more

j. Do you plan to subspecialize?
- ⊗ Yes
- ◯ No

k. Languages spoken?

- ☒ English
- ☐ Spanish
- ☐ French
- ☐ Hmong
- ☐ Somali
- ☐ Japanese
- ☐ Chinese
- ☐ Russian
- ☐ Ethiopian
- ☐ Other ___________

l. Are you in the Global Health Pathway?

- ◯ Yes
- ⊗ No

m. Did you earn your degree in the US?

- ⊗ Yes
- ◯ No

n. What residency program are you in?

- ◯ Internal Medicine
- ◯ Med-Peds
- ◯ Pediatrics
- ◯ Family Practice
- ◯ Neurology
- ◯ Psychiatry
- ◯ ObGyn
- ◯ Neurosurgery
- ◯ General Surgery
- ◯ Orthopedic Surgery
- ◯ Urology
- ⊗ Surgical sub-specialty (please specify in text box below)
- ◯ Non-clinical specialty (radiology, pathology; please specify in text box below)

Physical Medicine and Rehab, this actually doesn't fit any of your categories listed

Medical Trainees' attitudes, knowledge, and experience with immigrant and refugee health

Response was added on 11/12/2013 2:28pm.

SECTION A: Personal experience with immigrant and refugee health care.

A. Please indicate your level of agreement with the following statements regarding your personal experience with immigrant and refugee health care by checking the box that best represents your experience.

a. During my inpatient rotations, I take care of the following percentage of immigrant and refugee patients:
- ◯ None
- ⊗ 0 -5%
- ◯ 5-10%
- ◯ 10-25%
- ◯ > 25%

b. During my outpatient rotations, I take care of the following percentage of immigrant and refugee patients:
- ◯ None
- ⊗ 0-10%
- ◯ 10 -25%
- ◯ 25-50%
- ◯ 50-75%
- ◯ >75%

c. I would like to take care of more immigrant and refugee patients.
- ◯ Strongly disagree
- ◯ Disagree
- ⊗ No opinion
- ◯ Agree
- ◯ Strongly agree

d. I plan to take care of immigrants and refugees when I finish residency.
- ◯ Strongly disagree
- ◯ Disagree
- ◯ No opinion
- ◯ Agree
- ⊗ Strongly agree

e. I plan to do short term (< 6 months) international work when I finish residency.
- ◯ Strongly disagree
- ⊗ Disagree
- ◯ No opinion
- ◯ Agree
- ◯ Strongly agree

f. I plan to do long term (>6 months) international work when I finish residency.
- ⊗ Strongly disagree
- ◯ Disagree
- ◯ No opinion
- ◯ Agree
- ◯ Strongly agree

g. I plan to work in health disparities in the following way after residency:

within community of clinic where I work

SECTION B: MEDICAL EDUCATION

A. Please indicate your level of agreement with the following statements regarding your medical education and knowledge about immigrants and refugees by checking the box that best represents your opinion.

a. I have received specialized training in immigrant and refugee health, tropical medicine, or cross-cultural health.

- ◯ Strongly disagree
- ◯ Disagree
- ◯ No opinion
- ⊗ Agree
- ◯ Strongly agree

b. If you have received specialized training in immigrant and refugee health, tropical medicine, or cross-cultural health, please indicate all the contexts in which you received this training:

- ☐ As an undergraduate.
- ☐ As a medical student.
- ☒ As part of my residency.
- ☐ A special program.
- ☐ As part of my fellowship.
- ☐ As part of a degree program (e.g. MPH)
- ☐ Other:

c. I feel comfortable with my fund of knowledge regarding immigrant and refugee health.

- ◯ Strongly disagree
- ⊗ Disagree
- ◯ No opinion
- ◯ Agree
- ◯ Strongly agree

d. I would like to have further training in immigrant and refugee health.

- ◯ Strongly disagree
- ◯ Disagree
- ⊗ No opinion
- ◯ Agree
- ◯ Strongly agree

SECTION C: Attitudes towards immigrant health

A.Please indicate your level of agreement with the following statements regarding immigrant and refugee health by checking the box that best represents your opinion.

a. I enjoy taking care of immigrants and refugees.

- ◯ Never
- ◯ Rarely
- ◯ Sometimes
- ⊗ Usually
- ◯ Always

b. Please indicate the reasons that you enjoy taking care of immigrants and refugees (may choose more than one).

- ☒ Tropical and other conditions not frequently diagnosed in US-born patients
- ☒ Learning about other cultures
- ☒ They don't complain as much
- ☒ Being able to hear their stories
- ☐ Their care is more complicated
- ☐ Their care is less complicated
- ☒ They are very appreciative of your help.
- ☒ They are extremely vulnerable
- ☐ Other:

c. Taking care of immigrants and refugees is more challenging than taking care of US born patients.

- ◯ Never
- ◯ Rarely
- ◯ Sometimes
- ⊗ Usually
- ◯ Always

d. Please mark all the challenges that you face as a provider when providing care to immigrants and refugees (may choose more than one):

☒ Language barriers
☒ Insurance barriers
☒ Cultural barriers
☐ Finding a professional interpreter
☐ Knowing how to work with a professional interpreter
☒ Time constraints
☒ My own knowledge related to tropical and travel medicine
☒ Transportation problems for the patient
☒ Patients not understanding treatment plan
☐ Patients not following treatment plan
☒ My lack of knowledge regarding the patient's culture
☒ Bias or stereotyping
☐ Other:

e. Please mark all of the challenges faced by immigrant and refugee populations when receiving healthcare that you have perceived or witnessed (may choose more than one):

☒ Language barriers
☒ Insurance barriers
☒ Cultural barriers
☒ Finding a professional interpreter
☒ Knowing how to work with a professional interpreter
☒ Time constraints
☒ Insufficiently trained health care providers
☒ Transportation problems for the patient
☒ Food insufficiency
☒ Need for child care
☒ Patients not understanding treatment plan
☒ Patients not following treatment plan
☒ My lack of knowledge regarding the patient's culture
☒ Bias or stereotyping
☒ Trust issues
☐ Other...

f. Rank how well immigrants and refugees understand the healthcare that you are trying to provide.

◯ Significantly less than a US born individual
◯ Less than a US born individual
◯ Equivalent to a US born individual
⊗ More than a US born patient
◯ Significantly more than a US born individual

g. Immigrants and refugees adhere to treatment plans and follow my recommendations.

◯ Never
◯ Rarely
◯ Sometimes
⊗ Usually
◯ Always

h. Immigrants and refugees should receive the same care and insurance coverage as US born patients.

◯ Never
◯ Rarely
◯ Sometimes
◯ Usually
⊗ Always

i. Immigrants and refugees who are undocumented should receive the same care and insurance coverage as US born patients.

◯ Never
◯ Rarely
◯ Sometimes
◯ Usually
⊗ Always

j. Every physician is professionally obligated to care for immigrants and refugees if they present to your clinic or hospital.

◯ Strongly disagree
◯ Disagree
◯ No opinion
◯ Agree
⊗ Strongly agree

k. Is healthcare a human right? | ⊗ Yes ◯ No

B. If you wish, please tell us about what you enjoy or do not enjoy about immigrant and refugee health care and the greatest challenges you face in caring for this population. ____________________

SECTION D: DEMOGRAPHIC INFORMATION
Please answer the following questions by checking the box in front of the response choice that best describes you.

a. Your age?
◯ 20 to 24
◯ 25 to 29
⊗ 30 to 34
◯ 35 to 39
◯ 40 or older

b. Your gender?
◯ Female
⊗ Male
◯ Other

c. Are you Hispanic or Latino?
◯ Yes
⊗ No

d. What is your race? (Select one or more responses)
◯ American Indian or Alaska Native
◯ Asian (Please specify):
◯ Black or African American
◯ Native Hawaiian or Other Pacific Islander
⊗ White
◯ Other (Please specify):

e. Were you born in the United States?
⊗ Yes
◯ No

g. Your residency year?
◯ PGY1
◯ PGY2
◯ PGY3
⊗ PGY4
◯ PGY5

h. How would you classify your political ideology?
◯ Conservative
◯ Somewhat conservative
◯ Moderate
⊗ Somewhat liberal
◯ Liberal
◯ Other (Please specify):

i. Estimated level of educational debt?
◯ None
◯ Less than $50,000
◯ $50,000 - $100,000
⊗ $100,000 - $200,000
◯ $200,000 or more

j. Do you plan to subspecialize?
◯ Yes
⊗ No

k. Languages spoken?

- ☒ English
- ☒ Spanish
- ☐ French
- ☐ Hmong
- ☐ Somali
- ☐ Japanese
- ☐ Chinese
- ☐ Russian
- ☐ Ethiopian
- ☐ Other ___________

l. Are you in the Global Health Pathway?

- ◯ Yes
- ⊗ No

m. Did you earn your degree in the US?

- ⊗ Yes
- ◯ No

n. What residency program are you in?

- ◯ Internal Medicine
- ⊗ Med-Peds
- ◯ Pediatrics
- ◯ Family Practice
- ◯ Neurology
- ◯ Psychiatry
- ◯ ObGyn
- ◯ Neurosurgery
- ◯ General Surgery
- ◯ Orthopedic Surgery
- ◯ Urology
- ◯ Surgical sub-specialty (please specify in text box below)
- ◯ Non-clinical specialty (radiology, pathology; please specify in text box below)

Medical Trainees' attitudes, knowledge, and experience with immigrant and refugee health

Response was added on 11/13/2013 2:50pm.

SECTION A: Personal experience with immigrant and refugee health care.

A. Please indicate your level of agreement with the following statements regarding your personal experience with immigrant and refugee health care by checking the box that best represents your experience.

a. During my inpatient rotations, I take care of the following percentage of immigrant and refugee patients:

- ◯ None
- ⊗ 0 -5%
- ◯ 5-10%
- ◯ 10-25%
- ◯ > 25%

b. During my outpatient rotations, I take care of the following percentage of immigrant and refugee patients:

- ◯ None
- ◯ 0-10%
- ◯ 10 -25%
- ◯ 25-50%
- ◯ 50-75%
- ⊗ >75%

c. I would like to take care of more immigrant and refugee patients.

- ◯ Strongly disagree
- ◯ Disagree
- ◯ No opinion
- ⊗ Agree
- ◯ Strongly agree

d. I plan to take care of immigrants and refugees when I finish residency.

- ◯ Strongly disagree
- ◯ Disagree
- ◯ No opinion
- ⊗ Agree
- ◯ Strongly agree

e. I plan to do short term (< 6 months) international work when I finish residency.

- ◯ Strongly disagree
- ◯ Disagree
- ◯ No opinion
- ⊗ Agree
- ◯ Strongly agree

f. I plan to do long term (>6 months) international work when I finish residency.

- ◯ Strongly disagree
- ◯ Disagree
- ◯ No opinion
- ◯ Agree
- ⊗ Strongly agree

g. I plan to work in health disparities in the following way after residency:

Working with an NGO, international organization or non-profit in the realm of global health. Whether that's specifically with immigrants or refugees is uncertain, however I could see myself working in that capacity.

SECTION B: MEDICAL EDUCATION

A. Please indicate your level of agreement with the following statements regarding your medical education and knowledge about immigrants and refugees by checking the box that best represents your opinion.

a. I have received specialized training in immigrant and refugee health, tropical medicine, or cross-cultural health.

- ◯ Strongly disagree
- ◯ Disagree
- ◯ No opinion
- ⊗ Agree
- ◯ Strongly agree

b. If you have received specialized training in immigrant and refugee health, tropical medicine, or cross-cultural health, please indicate all the contexts in which you received this training:

- ☐ As an undergraduate.
- ☐ As a medical student.
- ☒ As part of my residency.
- ☒ A special program.
- ☐ As part of my fellowship.
- ☐ As part of a degree program (e.g. MPH)
- ☐ Other:

c. I feel comfortable with my fund of knowledge regarding immigrant and refugee health.

- ◯ Strongly disagree
- ⊗ Disagree
- ◯ No opinion
- ◯ Agree
- ◯ Strongly agree

d. I would like to have further training in immigrant and refugee health.

- ◯ Strongly disagree
- ◯ Disagree
- ◯ No opinion
- ⊗ Agree
- ◯ Strongly agree

e. If you agree with the above, please indicate all the contexts in which you would like to receive this training:

- ☒ As part of my residency.
- ☒ A special program.
- ☒ As part of my fellowship.

SECTION C: Attitudes towards immigrant health

A. Please indicate your level of agreement with the following statements regarding immigrant and refugee health by checking the box that best represents your opinion.

a. I enjoy taking care of immigrants and refugees.

- ◯ Never
- ◯ Rarely
- ◯ Sometimes
- ◯ Usually
- ⊗ Always

b. Please indicate the reasons that you enjoy taking care of immigrants and refugees (may choose more than one).

- ☒ Tropical and other conditions not frequently diagnosed in US-born patients
- ☒ Learning about other cultures
- ☐ They don't complain as much
- ☒ Being able to hear their stories
- ☒ Their care is more complicated
- ☐ Their care is less complicated
- ☒ They are very appreciative of your help.
- ☒ They are extremely vulnerable
- ☒ Other:

Social justice

c. Taking care of immigrants and refugees is more challenging than taking care of US born patients.

- ◯ Never
- ◯ Rarely
- ⊗ Sometimes
- ◯ Usually
- ◯ Always

d. Please mark all the challenges that you face as a provider when providing care to immigrants and refugees (may choose more than one):

- ☒ Language barriers
- ☐ Insurance barriers
- ☒ Cultural barriers
- ☐ Finding a professional interpreter
- ☐ Knowing how to work with a professional interpreter
- ☒ Time constraints
- ☒ My own knowledge related to tropical and travel medicine
- ☒ Transportation problems for the patient
- ☒ Patients not understanding treatment plan
- ☒ Patients not following treatment plan
- ☐ My lack of knowledge regarding the patient's culture
- ☐ Bias or stereotyping
- ☒ Other:

Varying expectations

e. Please mark all of the challenges faced by immigrant and refugee populations when receiving healthcare that you have perceived or witnessed (may choose more than one):

- ☒ Language barriers
- ☒ Insurance barriers
- ☒ Cultural barriers
- ☐ Finding a professional interpreter
- ☒ Knowing how to work with a professional interpreter
- ☐ Time constraints
- ☒ Insufficiently trained health care providers
- ☒ Transportation problems for the patient
- ☐ Food insufficiency
- ☐ Need for child care
- ☒ Patients not understanding treatment plan
- ☒ Patients not following treatment plan
- ☐ My lack of knowledge regarding the patient's culture
- ☐ Bias or stereotyping
- ☒ Trust issues
- ☐ Other...

f. Rank how well immigrants and refugees understand the healthcare that you are trying to provide.

- ◯ Significantly less than a US born individual
- ⊗ Less than a US born individual
- ◯ Equivalent to a US born individual
- ◯ More than a US born patient
- ◯ Significantly more than a US born individual

g. Immigrants and refugees adhere to treatment plans and follow my recommendations.

- ◯ Never
- ◯ Rarely
- ⊗ Sometimes
- ◯ Usually
- ◯ Always

h. Immigrants and refugees should receive the same care and insurance coverage as US born patients.

- ◯ Never
- ◯ Rarely
- ◯ Sometimes
- ◯ Usually
- ⊗ Always

i. Immigrants and refugees who are undocumented should receive the same care and insurance coverage as US born patients.

- ◯ Never
- ◯ Rarely
- ◯ Sometimes
- ◯ Usually
- ⊗ Always

j. Every physician is professionally obligated to care for immigrants and refugees if they present to your clinic or hospital.

- ◯ Strongly disagree
- ◯ Disagree
- ◯ No opinion
- ◯ Agree
- ⊗ Strongly agree

k. Is healthcare a human right?

- ⊗ Yes
- ◯ No

B. If you wish, please tell us about what you enjoy or do not enjoy about immigrant and refugee health care and the greatest challenges you face in caring for this population.

Refugees and immigrants represent some of the least powerful people on this planet. Their lives have been displaced and after migrating to a new country and culture, stability is difficult to come by. Power grows out of being able to retain agency and autonomy. With health, that can be achieved, however if someone doesn't have that basic internal control over their lives it is difficult for them to achieve their dreams and goals. I believe health provides an excellent entry for improving someones life, especially in these populations, who commonly have had consistent structural and political violence levied against them. So I think healthcare provides them a first start at a new life, because it gives them agencies over their own bodies, and that's a start.

SECTION D: DEMOGRAPHIC INFORMATION
Please answer the following questions by checking the box in front of the response choice that best describes you.

a. Your age?

- ⊗ 20 to 24
- ◯ 25 to 29
- ◯ 30 to 34
- ◯ 35 to 39
- ◯ 40 or older

b. Your gender?

- ◯ Female
- ⊗ Male
- ◯ Other

c. Are you Hispanic or Latino?

- ◯ Yes
- ⊗ No

d. What is your race? (Select one or more responses)

- ◯ American Indian or Alaska Native
- ◯ Asian (Please specify):
- ◯ Black or African American
- ◯ Native Hawaiian or Other Pacific Islander
- ⊗ White
- ◯ Other (Please specify):

e. Were you born in the United States?

- ⊗ Yes
- ◯ No

g. Your residency year?

- ⊗ PGY1
- ◯ PGY2
- ◯ PGY3
- ◯ PGY4
- ◯ PGY5

h. How would you classify your political ideology?

- ◯ Conservative
- ◯ Somewhat conservative
- ◯ Moderate
- ⊗ Somewhat liberal
- ◯ Liberal
- ◯ Other (Please specify):

i. Estimated level of educational debt?

- ◯ None
- ◯ Less than $50,000
- ⊗ $50,000 - $100,000
- ◯ $100,000 - $200,000
- ◯ $200,000 or more

j.Do you plan to subspecialize?

- ⊗ Yes
- ◯ No

k. Languages spoken?

- ☒ English
- ☐ Spanish
- ☐ French
- ☐ Hmong
- ☐ Somali
- ☐ Japanese
- ☐ Chinese
- ☐ Russian
- ☐ Ethiopian
- ☒ Other ___________

German

l. Are you in the Global Health Pathway?

- ⊗ Yes
- ◯ No

m. Did you earn your degree in the US?

- ⊗ Yes
- ◯ No

n. What residency program are you in?

- ⊗ Internal Medicine
- ◯ Med-Peds
- ◯ Pediatrics
- ◯ Family Practice
- ◯ Neurology
- ◯ Psychiatry
- ◯ ObGyn
- ◯ Neurosurgery
- ◯ General Surgery
- ◯ Orthopedic Surgery
- ◯ Urology
- ◯ Surgical sub-specialty (please specify in text box below)
- ◯ Non-clinical specialty (radiology, pathology; please specify in text box below)

Medical Trainees' attitudes, knowledge, and experience with immigrant and refugee health

Response was added on 11/23/2013 10:13pm.

SECTION A: Personal experience with immigrant and refugee health care.

A. Please indicate your level of agreement with the following statements regarding your personal experience with immigrant and refugee health care by checking the box that best represents your experience.

a. During my inpatient rotations, I take care of the following percentage of immigrant and refugee patients:

- ◯ None
- ⊗ 0 -5%
- ◯ 5-10%
- ◯ 10-25%
- ◯ > 25%

b. During my outpatient rotations, I take care of the following percentage of immigrant and refugee patients:

- ◯ None
- ◯ 0-10%
- ⊗ 10 -25%
- ◯ 25-50%
- ◯ 50-75%
- ◯ >75%

c. I would like to take care of more immigrant and refugee patients.

- ◯ Strongly disagree
- ◯ Disagree
- ◯ No opinion
- ⊗ Agree
- ◯ Strongly agree

d. I plan to take care of immigrants and refugees when I finish residency.

- ◯ Strongly disagree
- ◯ Disagree
- ◯ No opinion
- ⊗ Agree
- ◯ Strongly agree

e. I plan to do short term (< 6 months) international work when I finish residency.

- ◯ Strongly disagree
- ◯ Disagree
- ⊗ No opinion
- ◯ Agree
- ◯ Strongly agree

f. I plan to do long term (>6 months) international work when I finish residency.

- ◯ Strongly disagree
- ◯ Disagree
- ◯ No opinion
- ◯ Agree
- ⊗ Strongly agree

g. I plan to work in health disparities in the following way after residency:

National Health Service Corps--most likely Indian Health Service placement

SECTION B: MEDICAL EDUCATION

A. Please indicate your level of agreement with the following statements regarding your medical education and knowledge about immigrants and refugees by checking the box that best represents your opinion.

a. I have received specialized training in immigrant and refugee health, tropical medicine, or cross-cultural health.

- ○ Strongly disagree
- ○ Disagree
- ○ No opinion
- ○ Agree
- ⊗ Strongly agree

b. If you have received specialized training in immigrant and refugee health, tropical medicine, or cross-cultural health, please indicate all the contexts in which you received this training:

- ☐ As an undergraduate.
- ☒ As a medical student.
- ☒ As part of my residency.
- ☒ A special program.
- ☐ As part of my fellowship.
- ☐ As part of a degree program (e.g. MPH)
- ☐ Other:

c. I feel comfortable with my fund of knowledge regarding immigrant and refugee health.

- ○ Strongly disagree
- ○ Disagree
- ○ No opinion
- ⊗ Agree
- ○ Strongly agree

d. I would like to have further training in immigrant and refugee health.

- ○ Strongly disagree
- ○ Disagree
- ○ No opinion
- ⊗ Agree
- ○ Strongly agree

e. If you agree with the above, please indicate all the contexts in which you would like to receive this training:
□□

- ☒ As part of my residency.
- ☐ A special program.
- ☐ As part of my fellowship.

SECTION C: Attitudes towards immigrant health

A.□Please indicate your level of agreement with the following statements regarding immigrant and refugee health by checking the box that best represents your opinion.

a. I enjoy taking care of immigrants and refugees.

- ○ Never
- ○ Rarely
- ○ Sometimes
- ⊗ Usually
- ○ Always

b. Please indicate the reasons that you enjoy taking care of immigrants and refugees (may choose more than one).

- ☒ Tropical and other conditions not frequently diagnosed in US-born patients
- ☒ Learning about other cultures
- ☐ They don't complain as much
- ☐ Being able to hear their stories
- ☒ Their care is more complicated
- ☐ Their care is less complicated
- ☐ They are very appreciative of your help.
- ☐ They are extremely vulnerable
- ☐ Other:

c. Taking care of immigrants and refugees is more challenging than taking care of US born patients.

- ○ Never
- ○ Rarely
- ○ Sometimes
- ⊗ Usually
- ○ Always

d. Please mark all the challenges that you face as a provider when providing care to immigrants and refugees (may choose more than one):

- ☒ Language barriers
- ☐ Insurance barriers
- ☒ Cultural barriers
- ☒ Finding a professional interpreter
- ☐ Knowing how to work with a professional interpreter
- ☒ Time constraints
- ☒ My own knowledge related to tropical and travel medicine
- ☒ Transportation problems for the patient
- ☐ Patients not understanding treatment plan
- ☐ Patients not following treatment plan
- ☐ My lack of knowledge regarding the patient's culture
- ☐ Bias or stereotyping
- ☐ Other:

e. Please mark all of the challenges faced by immigrant and refugee populations when receiving healthcare that you have perceived or witnessed (may choose more than one):

- ☒ Language barriers
- ☒ Insurance barriers
- ☒ Cultural barriers
- ☒ Finding a professional interpreter
- ☐ Knowing how to work with a professional interpreter
- ☒ Time constraints
- ☒ Insufficiently trained health care providers
- ☒ Transportation problems for the patient
- ☒ Food insufficiency
- ☐ Need for child care
- ☒ Patients not understanding treatment plan
- ☒ Patients not following treatment plan
- ☒ My lack of knowledge regarding the patient's culture
- ☒ Bias or stereotyping
- ☒ Trust issues
- ☐ Other...

f. Rank how well immigrants and refugees understand the healthcare that you are trying to provide.

- ⊗ Significantly less than a US born individual
- ○ Less than a US born individual
- ○ Equivalent to a US born individual
- ○ More than a US born patient
- ○ Significantly more than a US born individual

g. Immigrants and refugees adhere to treatment plans and follow my recommendations.

- ○ Never
- ○ Rarely
- ○ Sometimes
- ⊗ Usually
- ○ Always

h. Immigrants and refugees should receive the same care and insurance coverage as US born patients.

- ○ Never
- ○ Rarely
- ○ Sometimes
- ○ Usually
- ⊗ Always

i. Immigrants and refugees who are undocumented should receive the same care and insurance coverage as US born patients.

- ○ Never
- ○ Rarely
- ○ Sometimes
- ○ Usually
- ⊗ Always

j. Every physician is professionally obligated to care for immigrants and refugees if they present to your clinic or hospital.

- ○ Strongly disagree
- ○ Disagree
- ○ No opinion
- ⊗ Agree
- ○ Strongly agree

k. Is healthcare a human right?

⊗ Yes
◯ No

B. If you wish, please tell us about what you enjoy or do not enjoy about immigrant and refugee health care and the greatest challenges you face in caring for this population.

The needs of immigrants are different based off their personal and health histories. Much of the evidence for health screening is based on Western research and data. A medical provider must use independent thought and recognize the unique health needs of the person of which they are providing care. Immigrant health is both challenging and exciting in this way.

SECTION D: DEMOGRAPHIC INFORMATION
Please answer the following questions by checking the box in front of the response choice that best describes you.

a. Your age?

◯ 20 to 24
◯ 25 to 29
⊗ 30 to 34
◯ 35 to 39
◯ 40 or older

b. Your gender?

⊗ Female
◯ Male
◯ Other

c. Are you Hispanic or Latino?

◯ Yes
⊗ No

d. What is your race? (Select one or more responses)

◯ American Indian or Alaska Native
◯ Asian (Please specify):
◯ Black or African American
◯ Native Hawaiian or Other Pacific Islander
⊗ White
◯ Other (Please specify):

e. Were you born in the United States?

⊗ Yes
◯ No

g. Your residency year?

◯ PGY1
◯ PGY2
⊗ PGY3
◯ PGY4
◯ PGY5

h. How would you classify your political ideology?

◯ Conservative
◯ Somewhat conservative
◯ Moderate
◯ Somewhat liberal
⊗ Liberal
◯ Other (Please specify):

i. Estimated level of educational debt?

⊗ None
◯ Less than $50,000
◯ $50,000 - $100,000
◯ $100,000 - $200,000
◯ $200,000 or more

j. Do you plan to subspecialize?

◯ Yes
⊗ No

k. Languages spoken?

- ☒ English
- ☐ Spanish
- ☐ French
- ☐ Hmong
- ☐ Somali
- ☐ Japanese
- ☐ Chinese
- ☐ Russian
- ☐ Ethiopian
- ☐ Other ___________

l. Are you in the Global Health Pathway?

- ⊗ Yes
- ○ No

m. Did you earn your degree in the US?

- ⊗ Yes
- ○ No

n. What residency program are you in?

- ○ Internal Medicine
- ○ Med-Peds
- ⊗ Pediatrics
- ○ Family Practice
- ○ Neurology
- ○ Psychiatry
- ○ ObGyn
- ○ Neurosurgery
- ○ General Surgery
- ○ Orthopedic Surgery
- ○ Urology
- ○ Surgical sub-specialty (please specify in text box below)
- ○ Non-clinical specialty (radiology, pathology; please specify in text box below)

Medical Trainees' attitudes, knowledge, and experience with immigrant and refugee health

Response was added on 12/12/2013 2:48pm.

SECTION A: Personal experience with immigrant and refugee health care.

A. Please indicate your level of agreement with the following statements regarding your personal experience with immigrant and refugee health care by checking the box that best represents your experience.

a. During my inpatient rotations, I take care of the following percentage of immigrant and refugee patients:

- ◯ None
- ◯ 0 -5%
- ◯ 5-10%
- ⊗ 10-25%
- ◯ > 25%

b. During my outpatient rotations, I take care of the following percentage of immigrant and refugee patients:

- ◯ None
- ◯ 0-10%
- ◯ 10 -25%
- ⊗ 25-50%
- ◯ 50-75%
- ◯ >75%

c. I would like to take care of more immigrant and refugee patients.

- ◯ Strongly disagree
- ◯ Disagree
- ◯ No opinion
- ◯ Agree
- ⊗ Strongly agree

d. I plan to take care of immigrants and refugees when I finish residency.

- ◯ Strongly disagree
- ◯ Disagree
- ◯ No opinion
- ⊗ Agree
- ◯ Strongly agree

e. I plan to do short term (< 6 months) international work when I finish residency.

- ◯ Strongly disagree
- ◯ Disagree
- ⊗ No opinion
- ◯ Agree
- ◯ Strongly agree

f. I plan to do long term (>6 months) international work when I finish residency.

- ◯ Strongly disagree
- ◯ Disagree
- ⊗ No opinion
- ◯ Agree
- ◯ Strongly agree

g. I plan to work in health disparities in the following way after residency:

Volunteering with free clinics

SECTION B: MEDICAL EDUCATION

A. Please indicate your level of agreement with the following statements regarding your medical education and knowledge about immigrants and refugees by checking the box that best represents your opinion.

a. I have received specialized training in immigrant and refugee health, tropical medicine, or cross-cultural health.

- ◯ Strongly disagree
- ⊗ Disagree
- ◯ No opinion
- ◯ Agree
- ◯ Strongly agree

c. I feel comfortable with my fund of knowledge regarding immigrant and refugee health.

- ◯ Strongly disagree
- ⊗ Disagree
- ◯ No opinion
- ◯ Agree
- ◯ Strongly agree

d. I would like to have further training in immigrant and refugee health.

- ◯ Strongly disagree
- ◯ Disagree
- ◯ No opinion
- ◯ Agree
- ⊗ Strongly agree

e. If you agree with the above, please indicate all the contexts in which you would like to receive this training:
□□

- ☒ As part of my residency.
- ☐ A special program.
- ☐ As part of my fellowship.

SECTION C: Attitudes towards immigrant health

A.□Please indicate your level of agreement with the following statements regarding immigrant and refugee health by checking the box that best represents your opinion.

a. I enjoy taking care of immigrants and refugees.

- ◯ Never
- ◯ Rarely
- ◯ Sometimes
- ⊗ Usually
- ◯ Always

b. Please indicate the reasons that you enjoy taking care of immigrants and refugees (may choose more than one).

- ☒ Tropical and other conditions not frequently diagnosed in US-born patients
- ☒ Learning about other cultures
- ☐ They don't complain as much
- ☒ Being able to hear their stories
- ☐ Their care is more complicated
- ☐ Their care is less complicated
- ☐ They are very appreciative of your help.
- ☐ They are extremely vulnerable
- ☐ Other:

c. Taking care of immigrants and refugees is more challenging than taking care of US born patients.

- ◯ Never
- ◯ Rarely
- ⊗ Sometimes
- ◯ Usually
- ◯ Always

d. Please mark all the challenges that you face as a provider when providing care to immigrants and refugees (may choose more than one):

- ☒ Language barriers
- ☐ Insurance barriers
- ☒ Cultural barriers
- ☒ Finding a professional interpreter
- ☐ Knowing how to work with a professional interpreter
- ☒ Time constraints
- ☐ My own knowledge related to tropical and travel medicine
- ☐ Transportation problems for the patient
- ☒ Patients not understanding treatment plan
- ☐ Patients not following treatment plan
- ☒ My lack of knowledge regarding the patient's culture
- ☐ Bias or stereotyping
- ☐ Other:

e. Please mark all of the challenges faced by immigrant and refugee populations when receiving healthcare that you have perceived or witnessed (may choose more than one):

- ☒ Language barriers
- ☐ Insurance barriers
- ☒ Cultural barriers
- ☒ Finding a professional interpreter
- ☐ Knowing how to work with a professional interpreter
- ☒ Time constraints
- ☐ Insufficiently trained health care providers
- ☐ Transportation problems for the patient
- ☐ Food insufficiency
- ☐ Need for child care
- ☒ Patients not understanding treatment plan
- ☐ Patients not following treatment plan
- ☒ My lack of knowledge regarding the patient's culture
- ☐ Bias or stereotyping
- ☐ Trust issues
- ☐ Other...

f. Rank how well immigrants and refugees understand the healthcare that you are trying to provide.

- ⊗ Significantly less than a US born individual
- ◯ Less than a US born individual
- ◯ Equivalent to a US born individual
- ◯ More than a US born patient
- ◯ Significantly more than a US born individual

g. Immigrants and refugees adhere to treatment plans and follow my recommendations.

- ◯ Never
- ◯ Rarely
- ⊗ Sometimes
- ◯ Usually
- ◯ Always

h. Immigrants and refugees should receive the same care and insurance coverage as US born patients.

- ◯ Never
- ◯ Rarely
- ◯ Sometimes
- ◯ Usually
- ⊗ Always

i. Immigrants and refugees who are undocumented should receive the same care and insurance coverage as US born patients.

- ◯ Never
- ◯ Rarely
- ⊗ Sometimes
- ◯ Usually
- ◯ Always

j. Every physician is professionally obligated to care for immigrants and refugees if they present to your clinic or hospital.

- ◯ Strongly disagree
- ◯ Disagree
- ◯ No opinion
- ⊗ Agree
- ◯ Strongly agree

k. Is healthcare a human right?
- ⊗ Yes
- ◯ No

B. If you wish, please tell us about what you enjoy or do not enjoy about immigrant and refugee health care and the greatest challenges you face in caring for this population.

____

SECTION D: DEMOGRAPHIC INFORMATION
Please answer the following questions by checking the box in front of the response choice that best describes you.

a. Your age?
- ◯ 20 to 24
- ◯ 25 to 29
- ⊗ 30 to 34
- ◯ 35 to 39
- ◯ 40 or older

b. Your gender?
- ⊗ Female
- ◯ Male
- ◯ Other

c.Are you Hispanic or Latino?
- ◯ Yes
- ⊗ No

d. What is your race? (Select one or more responses)
- ◯ American Indian or Alaska Native
- ◯ Asian (Please specify):
- ◯ Black or African American
- ◯ Native Hawaiian or Other Pacific Islander
- ⊗ White
- ◯ Other (Please specify):

e.Were you born in the United States?
- ⊗ Yes
- ◯ No

g. Your residency year?
- ⊗ PGY1
- ◯ PGY2
- ◯ PGY3
- ◯ PGY4
- ◯ PGY5

h. How would you classify your political ideology?
- ◯ Conservative
- ◯ Somewhat conservative
- ◯ Moderate
- ◯ Somewhat liberal
- ⊗ Liberal
- ◯ Other (Please specify):

i. Estimated level of educational debt?
- ◯ None
- ◯ Less than $50,000
- ◯ $50,000 - $100,000
- ◯ $100,000 - $200,000
- ⊗ $200,000 or more

j.Do you plan to subspecialize?
- ◯ Yes
- ⊗ No

k. Languages spoken?

- ☒ English
- ☐ Spanish
- ☒ French
- ☐ Hmong
- ☐ Somali
- ☐ Japanese
- ☐ Chinese
- ☐ Russian
- ☐ Ethiopian
- ☐ Other ___________

l. Are you in the Global Health Pathway?

- ◯ Yes
- ⊗ No

m. Did you earn your degree in the US?

- ⊗ Yes
- ◯ No

n. What residency program are you in?

- ◯ Internal Medicine
- ◯ Med-Peds
- ⊗ Pediatrics
- ◯ Family Practice
- ◯ Neurology
- ◯ Psychiatry
- ◯ ObGyn
- ◯ Neurosurgery
- ◯ General Surgery
- ◯ Orthopedic Surgery
- ◯ Urology
- ◯ Surgical sub-specialty (please specify in text box below)
- ◯ Non-clinical specialty (radiology, pathology; please specify in text box below)

Medical Trainees' attitudes, knowledge, and experience with immigrant and refugee health

Response was added on 12/13/2013 1:45pm.

SECTION A: Personal experience with immigrant and refugee health care.

A. Please indicate your level of agreement with the following statements regarding your personal experience with immigrant and refugee health care by checking the box that best represents your experience.

a. During my inpatient rotations, I take care of the following percentage of immigrant and refugee patients:

- ◯ None
- ◯ 0 -5%
- ◯ 5-10%
- ⊗ 10-25%
- ◯ > 25%

b. During my outpatient rotations, I take care of the following percentage of immigrant and refugee patients:

- ◯ None
- ⊗ 0-10%
- ◯ 10 -25%
- ◯ 25-50%
- ◯ 50-75%
- ◯ >75%

c. I would like to take care of more immigrant and refugee patients.

- ◯ Strongly disagree
- ◯ Disagree
- ◯ No opinion
- ◯ Agree
- ⊗ Strongly agree

d. I plan to take care of immigrants and refugees when I finish residency.

- ◯ Strongly disagree
- ◯ Disagree
- ◯ No opinion
- ⊗ Agree
- ◯ Strongly agree

e. I plan to do short term (< 6 months) international work when I finish residency.

- ◯ Strongly disagree
- ◯ Disagree
- ⊗ No opinion
- ◯ Agree
- ◯ Strongly agree

f. I plan to do long term (>6 months) international work when I finish residency.

- ◯ Strongly disagree
- ◯ Disagree
- ◯ No opinion
- ⊗ Agree
- ◯ Strongly agree

g. I plan to work in health disparities in the following way after residency:

Work in a disadvantaged area

SECTION B: MEDICAL EDUCATION

A. Please indicate your level of agreement with the following statements regarding your medical education and knowledge about immigrants and refugees by checking the box that best represents your opinion.

a. I have received specialized training in immigrant and refugee health, tropical medicine, or cross-cultural health.

○ Strongly disagree
⊗ Disagree
○ No opinion
○ Agree
○ Strongly agree

c. I feel comfortable with my fund of knowledge regarding immigrant and refugee health.

○ Strongly disagree
○ Disagree
○ No opinion
⊗ Agree
○ Strongly agree

d. I would like to have further training in immigrant and refugee health.

○ Strongly disagree
○ Disagree
○ No opinion
⊗ Agree
○ Strongly agree

e. If you agree with the above, please indicate all the contexts in which you would like to receive this training:
□□

☒ As part of my residency.
☐ A special program.
☐ As part of my fellowship.

SECTION C: Attitudes towards immigrant health

A.□Please indicate your level of agreement with the following statements regarding immigrant and refugee health by checking the box that best represents your opinion.

a. I enjoy taking care of immigrants and refugees.

○ Never
○ Rarely
○ Sometimes
⊗ Usually
○ Always

b. Please indicate the reasons that you enjoy taking care of immigrants and refugees (may choose more than one).

☐ Tropical and other conditions not frequently diagnosed in US-born patients
☒ Learning about other cultures
☐ They don't complain as much
☒ Being able to hear their stories
☐ Their care is more complicated
☐ Their care is less complicated
☐ They are very appreciative of your help.
☐ They are extremely vulnerable
☐ Other:

c. Taking care of immigrants and refugees is more challenging than taking care of US born patients.

○ Never
○ Rarely
⊗ Sometimes
○ Usually
○ Always

d. Please mark all the challenges that you face as a provider when providing care to immigrants and refugees (may choose more than one):

- ☐ Language barriers
- ☐ Insurance barriers
- ☒ Cultural barriers
- ☒ Finding a professional interpreter
- ☐ Knowing how to work with a professional interpreter
- ☐ Time constraints
- ☐ My own knowledge related to tropical and travel medicine
- ☐ Transportation problems for the patient
- ☒ Patients not understanding treatment plan
- ☐ Patients not following treatment plan
- ☐ My lack of knowledge regarding the patient's culture
- ☐ Bias or stereotyping
- ☐ Other:

e. Please mark all of the challenges faced by immigrant and refugee populations when receiving healthcare that you have perceived or witnessed (may choose more than one):

- ☐ Language barriers
- ☐ Insurance barriers
- ☒ Cultural barriers
- ☐ Finding a professional interpreter
- ☐ Knowing how to work with a professional interpreter
- ☐ Time constraints
- ☐ Insufficiently trained health care providers
- ☐ Transportation problems for the patient
- ☐ Food insufficiency
- ☐ Need for child care
- ☐ Patients not understanding treatment plan
- ☐ Patients not following treatment plan
- ☐ My lack of knowledge regarding the patient's culture
- ☐ Bias or stereotyping
- ☐ Trust issues
- ☐ Other...

f. Rank how well immigrants and refugees understand the healthcare that you are trying to provide.

- ○ Significantly less than a US born individual
- ⊗ Less than a US born individual
- ○ Equivalent to a US born individual
- ○ More than a US born patient
- ○ Significantly more than a US born individual

g. Immigrants and refugees adhere to treatment plans and follow my recommendations.

- ○ Never
- ○ Rarely
- ○ Sometimes
- ⊗ Usually
- ○ Always

h. Immigrants and refugees should receive the same care and insurance coverage as US born patients.

- ○ Never
- ○ Rarely
- ○ Sometimes
- ○ Usually
- ○ Always

i. Immigrants and refugees who are undocumented should receive the same care and insurance coverage as US born patients.

- ○ Never
- ○ Rarely
- ○ Sometimes
- ○ Usually
- ⊗ Always

j. Every physician is professionally obligated to care for immigrants and refugees if they present to your clinic or hospital.

- ○ Strongly disagree
- ○ Disagree
- ○ No opinion
- ○ Agree
- ⊗ Strongly agree

k. Is healthcare a human right?

⊗ Yes
◯ No

B. If you wish, please tell us about what you enjoy or do not enjoy about immigrant and refugee health care and the greatest challenges you face in caring for this population.

____

SECTION D: DEMOGRAPHIC INFORMATION
Please answer the following questions by checking the box in front of the response choice that best describes you.

a. Your age?

◯ 20 to 24
◯ 25 to 29
◯ 30 to 34
⊗ 35 to 39
◯ 40 or older

b. Your gender?

◯ Female
⊗ Male
◯ Other

c.Are you Hispanic or Latino?

◯ Yes
⊗ No

d. What is your race? (Select one or more responses)

◯ American Indian or Alaska Native
◯ Asian (Please specify):
⊗ Black or African American
◯ Native Hawaiian or Other Pacific Islander
◯ White
◯ Other (Please specify):

e.Were you born in the United States?

◯ Yes
⊗ No

f.If not, in what country were you born?

Sudan

g. Your residency year?

◯ PGY1
◯ PGY2
◯ PGY3
⊗ PGY4
◯ PGY5

h. How would you classify your political ideology?

◯ Conservative
◯ Somewhat conservative
◯ Moderate
⊗ Somewhat liberal
◯ Liberal
◯ Other (Please specify):

i. Estimated level of educational debt?

⊗ None
◯ Less than $50,000
◯ $50,000 - $100,000
◯ $100,000 - $200,000
◯ $200,000 or more

j.Do you plan to subspecialize?

⊗ Yes
◯ No

k. Languages spoken?

☒ English
☐ Spanish
☒ French
☐ Hmong
☐ Somali
☐ Japanese
☐ Chinese
☐ Russian
☐ Ethiopian
☒ Other ___________

Arabic

l. Are you in the Global Health Pathway?

◯ Yes
⊗ No

m. Did you earn your degree in the US?

◯ Yes
⊗ No

n. What residency program are you in?

◯ Internal Medicine
◯ Med-Peds
◯ Pediatrics
◯ Family Practice
⊗ Neurology
◯ Psychiatry
◯ ObGyn
◯ Neurosurgery
◯ General Surgery
◯ Orthopedic Surgery
◯ Urology
◯ Surgical sub-specialty (please specify in text box below)
◯ Non-clinical specialty (radiology, pathology; please specify in text box below)

Medical Trainees' attitudes, knowledge, and experience with immigrant and refugee health

Response was added on 12/13/2013 1:54pm.

SECTION A: Personal experience with immigrant and refugee health care.

A. Please indicate your level of agreement with the following statements regarding your personal experience with immigrant and refugee health care by checking the box that best represents your experience.

a. During my inpatient rotations, I take care of the following percentage of immigrant and refugee patients:

- ◯ None
- ◯ 0 -5%
- ◯ 5-10%
- ⊗ 10-25%
- ◯ > 25%

b. During my outpatient rotations, I take care of the following percentage of immigrant and refugee patients:

- ◯ None
- ◯ 0-10%
- ⊗ 10 -25%
- ◯ 25-50%
- ◯ 50-75%
- ◯ >75%

c. I would like to take care of more immigrant and refugee patients.

- ◯ Strongly disagree
- ⊗ Disagree
- ◯ No opinion
- ◯ Agree
- ◯ Strongly agree

d. I plan to take care of immigrants and refugees when I finish residency.

- ◯ Strongly disagree
- ◯ Disagree
- ◯ No opinion
- ⊗ Agree
- ◯ Strongly agree

e. I plan to do short term (< 6 months) international work when I finish residency.

- ◯ Strongly disagree
- ◯ Disagree
- ◯ No opinion
- ⊗ Agree
- ◯ Strongly agree

f. I plan to do long term (>6 months) international work when I finish residency.

- ◯ Strongly disagree
- ⊗ Disagree
- ◯ No opinion
- ◯ Agree
- ◯ Strongly agree

g. I plan to work in health disparities in the following way after residency:

Practice in rural, underserved area

SECTION B: MEDICAL EDUCATION

A. Please indicate your level of agreement with the following statements regarding your medical education and knowledge about immigrants and refugees by checking the box that best represents your opinion.

a. I have received specialized training in immigrant and refugee health, tropical medicine, or cross-cultural health.

- ◯ Strongly disagree
- ⊗ Disagree
- ◯ No opinion
- ◯ Agree
- ◯ Strongly agree

c. I feel comfortable with my fund of knowledge regarding immigrant and refugee health.

- ◯ Strongly disagree
- ◯ Disagree
- ⊗ No opinion
- ◯ Agree
- ◯ Strongly agree

d. I would like to have further training in immigrant and refugee health.

- ◯ Strongly disagree
- ⊗ Disagree
- ◯ No opinion
- ◯ Agree
- ◯ Strongly agree

SECTION C: Attitudes towards immigrant health

A.Please indicate your level of agreement with the following statements regarding immigrant and refugee health by checking the box that best represents your opinion.

a. I enjoy taking care of immigrants and refugees.

- ◯ Never
- ⊗ Rarely
- ◯ Sometimes
- ◯ Usually
- ◯ Always

b. Please indicate the reasons that you enjoy taking care of immigrants and refugees (may choose more than one).

- ☒ Tropical and other conditions not frequently diagnosed in US-born patients
- ☐ Learning about other cultures
- ☐ They don't complain as much
- ☐ Being able to hear their stories
- ☐ Their care is more complicated
- ☐ Their care is less complicated
- ☐ They are very appreciative of your help.
- ☐ They are extremely vulnerable
- ☐ Other:

c. Taking care of immigrants and refugees is more challenging than taking care of US born patients.

- ◯ Never
- ◯ Rarely
- ◯ Sometimes
- ⊗ Usually
- ◯ Always

d. Please mark all the challenges that you face as a provider when providing care to immigrants and refugees (may choose more than one):

- ☒ Language barriers
- ☐ Insurance barriers
- ☒ Cultural barriers
- ☒ Finding a professional interpreter
- ☐ Knowing how to work with a professional interpreter
- ☒ Time constraints
- ☒ My own knowledge related to tropical and travel medicine
- ☒ Transportation problems for the patient
- ☒ Patients not understanding treatment plan
- ☒ Patients not following treatment plan
- ☐ My lack of knowledge regarding the patient's culture
- ☐ Bias or stereotyping
- ☐ Other:

e. Please mark all of the challenges faced by immigrant and refugee populations when receiving healthcare that you have perceived or witnessed (may choose more than one):

- ☒ Language barriers
- ☒ Insurance barriers
- ☒ Cultural barriers
- ☒ Finding a professional interpreter
- ☒ Knowing how to work with a professional interpreter
- ☐ Time constraints
- ☐ Insufficiently trained health care providers
- ☒ Transportation problems for the patient
- ☐ Food insufficiency
- ☐ Need for child care
- ☒ Patients not understanding treatment plan
- ☒ Patients not following treatment plan
- ☐ My lack of knowledge regarding the patient's culture
- ☐ Bias or stereotyping
- ☐ Trust issues
- ☐ Other...

f. Rank how well immigrants and refugees understand the healthcare that you are trying to provide.

- ⊗ Significantly less than a US born individual
- ◯ Less than a US born individual
- ◯ Equivalent to a US born individual
- ◯ More than a US born patient
- ◯ Significantly more than a US born individual

g. Immigrants and refugees adhere to treatment plans and follow my recommendations.

- ◯ Never
- ◯ Rarely
- ⊗ Sometimes
- ◯ Usually
- ◯ Always

h. Immigrants and refugees should receive the same care and insurance coverage as US born patients.

- ◯ Never
- ◯ Rarely
- ⊗ Sometimes
- ◯ Usually
- ◯ Always

i. Immigrants and refugees who are undocumented should receive the same care and insurance coverage as US born patients.

- ⊗ Never
- ◯ Rarely
- ◯ Sometimes
- ◯ Usually
- ◯ Always

j. Every physician is professionally obligated to care for immigrants and refugees if they present to your clinic or hospital.

- ◯ Strongly disagree
- ◯ Disagree
- ◯ No opinion
- ⊗ Agree
- ◯ Strongly agree

k. Is healthcare a human right?

- ⊗ Yes
- ◯ No

B. If you wish, please tell us about what you enjoy or do not enjoy about immigrant and refugee health care and the greatest challenges you face in caring for this population.

____

SECTION D: DEMOGRAPHIC INFORMATION
Please answer the following questions by checking the box in front of the response choice that best describes you.

a. Your age?
- ◯ 20 to 24
- ⊗ 25 to 29
- ◯ 30 to 34
- ◯ 35 to 39
- ◯ 40 or older

b. Your gender?
- ⊗ Female
- ◯ Male
- ◯ Other

c. Are you Hispanic or Latino?
- ◯ Yes
- ⊗ No

d. What is your race? (Select one or more responses)
- ◯ American Indian or Alaska Native
- ◯ Asian (Please specify):
- ◯ Black or African American
- ◯ Native Hawaiian or Other Pacific Islander
- ⊗ White
- ◯ Other (Please specify):

e. Were you born in the United States?
- ⊗ Yes
- ◯ No

g. Your residency year?
- ◯ PGY1
- ⊗ PGY2
- ◯ PGY3
- ◯ PGY4
- ◯ PGY5

h. How would you classify your political ideology?
- ⊗ Conservative
- ◯ Somewhat conservative
- ◯ Moderate
- ◯ Somewhat liberal
- ◯ Liberal
- ◯ Other (Please specify):

i. Estimated level of educational debt?
- ◯ None
- ◯ Less than $50,000
- ◯ $50,000 - $100,000
- ⊗ $100,000 - $200,000
- ◯ $200,000 or more

j. Do you plan to subspecialize?
- ⊗ Yes
- ◯ No

k. Languages spoken?
- ☒ English
- ☒ Spanish
- ☐ French
- ☐ Hmong
- ☐ Somali
- ☐ Japanese
- ☐ Chinese
- ☐ Russian
- ☐ Ethiopian
- ☐ Other ___________

l. Are you in the Global Health Pathway?
- ◯ Yes
- ⊗ No

m. Did you earn your degree in the US?

⊗ Yes
◯ No

n. What residency program are you in?

◯ Internal Medicine
◯ Med-Peds
◯ Pediatrics
◯ Family Practice
◯ Neurology
◯ Psychiatry
◯ ObGyn
◯ Neurosurgery
◯ General Surgery
◯ Orthopedic Surgery
◯ Urology
⊗ Surgical sub-specialty (please specify in text box below)
◯ Non-clinical specialty (radiology, pathology; please specify in text box below)

____

Medical Trainees' attitudes, knowledge, and experience with immigrant and refugee health

Response was added on 12/13/2013 2:40pm.

SECTION A: Personal experience with immigrant and refugee health care.

A. Please indicate your level of agreement with the following statements regarding your personal experience with immigrant and refugee health care by checking the box that best represents your experience.

a. During my inpatient rotations, I take care of the following percentage of immigrant and refugee patients:

- ◯ None
- ◯ 0 -5%
- ◯ 5-10%
- ⊗ 10-25%
- ◯ > 25%

b. During my outpatient rotations, I take care of the following percentage of immigrant and refugee patients:

- ◯ None
- ◯ 0-10%
- ◯ 10 -25%
- ⊗ 25-50%
- ◯ 50-75%
- ◯ >75%

c. I would like to take care of more immigrant and refugee patients.

- ◯ Strongly disagree
- ◯ Disagree
- ◯ No opinion
- ⊗ Agree
- ◯ Strongly agree

d. I plan to take care of immigrants and refugees when I finish residency.

- ◯ Strongly disagree
- ◯ Disagree
- ◯ No opinion
- ⊗ Agree
- ◯ Strongly agree

e. I plan to do short term (< 6 months) international work when I finish residency.

- ◯ Strongly disagree
- ◯ Disagree
- ◯ No opinion
- ⊗ Agree
- ◯ Strongly agree

f. I plan to do long term (>6 months) international work when I finish residency.

- ◯ Strongly disagree
- ⊗ Disagree
- ◯ No opinion
- ◯ Agree
- ◯ Strongly agree

g. I plan to work in health disparities in the following way after residency: undetermined

SECTION B: MEDICAL EDUCATION

A. Please indicate your level of agreement with the following statements regarding your medical education and knowledge about immigrants and refugees by checking the box that best represents your opinion.

a. I have received specialized training in immigrant and refugee health, tropical medicine, or cross-cultural health.
- ◯ Strongly disagree
- ◯ Disagree
- ◯ No opinion
- ⊗ Agree
- ◯ Strongly agree

b. If you have received specialized training in immigrant and refugee health, tropical medicine, or cross-cultural health, please indicate all the contexts in which you received this training:
- ☐ As an undergraduate.
- ☒ As a medical student.
- ☐ As part of my residency.
- ☐ A special program.
- ☐ As part of my fellowship.
- ☐ As part of a degree program (e.g. MPH)
- ☐ Other:

c. I feel comfortable with my fund of knowledge regarding immigrant and refugee health.
- ◯ Strongly disagree
- ◯ Disagree
- ◯ No opinion
- ⊗ Agree
- ◯ Strongly agree

d. I would like to have further training in immigrant and refugee health.
- ◯ Strongly disagree
- ◯ Disagree
- ◯ No opinion
- ◯ Agree
- ⊗ Strongly agree

e. If you agree with the above, please indicate all the contexts in which you would like to receive this training:
- ☒ As part of my residency.
- ☒ A special program.
- ☐ As part of my fellowship.

SECTION C: Attitudes towards immigrant health

A. Please indicate your level of agreement with the following statements regarding immigrant and refugee health by checking the box that best represents your opinion.

a. I enjoy taking care of immigrants and refugees.
- ◯ Never
- ◯ Rarely
- ◯ Sometimes
- ⊗ Usually
- ◯ Always

b. Please indicate the reasons that you enjoy taking care of immigrants and refugees (may choose more than one).
- ☒ Tropical and other conditions not frequently diagnosed in US-born patients
- ☒ Learning about other cultures
- ☒ They don't complain as much
- ☒ Being able to hear their stories
- ☐ Their care is more complicated
- ☐ Their care is less complicated
- ☒ They are very appreciative of your help.
- ☒ They are extremely vulnerable
- ☐ Other:

c. Taking care of immigrants and refugees is more challenging than taking care of US born patients.
- ◯ Never
- ◯ Rarely
- ◯ Sometimes
- ⊗ Usually
- ◯ Always

d. Please mark all the challenges that you face as a provider when providing care to immigrants and refugees (may choose more than one):

- ☒ Language barriers
- ☒ Insurance barriers
- ☒ Cultural barriers
- ☒ Finding a professional interpreter
- ☐ Knowing how to work with a professional interpreter
- ☐ Time constraints
- ☒ My own knowledge related to tropical and travel medicine
- ☒ Transportation problems for the patient
- ☒ Patients not understanding treatment plan
- ☒ Patients not following treatment plan
- ☒ My lack of knowledge regarding the patient's culture
- ☒ Bias or stereotyping
- ☐ Other:

e. Please mark all of the challenges faced by immigrant and refugee populations when receiving healthcare that you have perceived or witnessed (may choose more than one):

- ☒ Language barriers
- ☒ Insurance barriers
- ☒ Cultural barriers
- ☒ Finding a professional interpreter
- ☐ Knowing how to work with a professional interpreter
- ☐ Time constraints
- ☒ Insufficiently trained health care providers
- ☒ Transportation problems for the patient
- ☐ Food insufficiency
- ☐ Need for child care
- ☒ Patients not understanding treatment plan
- ☒ Patients not following treatment plan
- ☒ My lack of knowledge regarding the patient's culture
- ☒ Bias or stereotyping
- ☒ Trust issues
- ☐ Other...

f. Rank how well immigrants and refugees understand the healthcare that you are trying to provide.

- ○ Significantly less than a US born individual
- ⊗ Less than a US born individual
- ○ Equivalent to a US born individual
- ○ More than a US born patient
- ○ Significantly more than a US born individual

g. Immigrants and refugees adhere to treatment plans and follow my recommendations.

- ○ Never
- ○ Rarely
- ○ Sometimes
- ⊗ Usually
- ○ Always

h. Immigrants and refugees should receive the same care and insurance coverage as US born patients.

- ○ Never
- ○ Rarely
- ○ Sometimes
- ○ Usually
- ⊗ Always

i. Immigrants and refugees who are undocumented should receive the same care and insurance coverage as US born patients.

- ○ Never
- ○ Rarely
- ○ Sometimes
- ○ Usually
- ⊗ Always

j. Every physician is professionally obligated to care for immigrants and refugees if they present to your clinic or hospital.

- ○ Strongly disagree
- ○ Disagree
- ○ No opinion
- ○ Agree
- ⊗ Strongly agree

k. Is healthcare a human right?

⊗ Yes
◯ No

B. If you wish, please tell us about what you enjoy or do not enjoy about immigrant and refugee health care and the greatest challenges you face in caring for this population.

____

SECTION D: DEMOGRAPHIC INFORMATION
Please answer the following questions by checking the box in front of the response choice that best describes you.

a. Your age?

◯ 20 to 24
⊗ 25 to 29
◯ 30 to 34
◯ 35 to 39
◯ 40 or older

b. Your gender?

⊗ Female
◯ Male
◯ Other

c.Are you Hispanic or Latino?

◯ Yes
⊗ No

d. What is your race? (Select one or more responses)

◯ American Indian or Alaska Native
◯ Asian (Please specify):
◯ Black or African American
◯ Native Hawaiian or Other Pacific Islander
⊗ White
◯ Other (Please specify):

e.Were you born in the United States?

⊗ Yes
◯ No

g. Your residency year?

◯ PGY1
⊗ PGY2
◯ PGY3
◯ PGY4
◯ PGY5

h. How would you classify your political ideology?

◯ Conservative
◯ Somewhat conservative
◯ Moderate
◯ Somewhat liberal
⊗ Liberal
◯ Other (Please specify):

i. Estimated level of educational debt?

◯ None
◯ Less than $50,000
◯ $50,000 - $100,000
⊗ $100,000 - $200,000
◯ $200,000 or more

j.Do you plan to subspecialize?

◯ Yes
⊗ No

k. Languages spoken?

- ☒ English
- ☒ Spanish
- ☐ French
- ☐ Hmong
- ☐ Somali
- ☐ Japanese
- ☐ Chinese
- ☐ Russian
- ☐ Ethiopian
- ☐ Other ___________

l. Are you in the Global Health Pathway?

- ⊗ Yes
- ◯ No

m.□ Did you earn your degree in the US?

- ⊗ Yes
- ◯ No

n. What residency program are you in?

- ◯ Internal Medicine
- ◯ Med-Peds
- ⊗ Pediatrics
- ◯ Family Practice
- ◯ Neurology
- ◯ Psychiatry
- ◯ ObGyn
- ◯ Neurosurgery
- ◯ General Surgery
- ◯ Orthopedic Surgery
- ◯ Urology
- ◯ Surgical sub-specialty (please specify in text box below)
- ◯ Non-clinical specialty (radiology, pathology; please specify in text box below)

Medical Trainees' attitudes, knowledge, and experience with immigrant and refugee health

Response was added on 12/13/2013 2:45pm.

SECTION A: Personal experience with immigrant and refugee health care.

A. Please indicate your level of agreement with the following statements regarding your personal experience with immigrant and refugee health care by checking the box that best represents your experience.

a. During my inpatient rotations, I take care of the following percentage of immigrant and refugee patients:
- ◯ None
- ◯ 0 -5%
- ⊗ 5-10%
- ◯ 10-25%
- ◯ > 25%

b. During my outpatient rotations, I take care of the following percentage of immigrant and refugee patients:
- ◯ None
- ◯ 0-10%
- ⊗ 10 -25%
- ◯ 25-50%
- ◯ 50-75%
- ◯ >75%

c. I would like to take care of more immigrant and refugee patients.
- ⊗ Strongly disagree
- ◯ Disagree
- ◯ No opinion
- ◯ Agree
- ◯ Strongly agree

d. I plan to take care of immigrants and refugees when I finish residency.
- ⊗ Strongly disagree
- ◯ Disagree
- ◯ No opinion
- ◯ Agree
- ◯ Strongly agree

e. I plan to do short term (< 6 months) international work when I finish residency.
- ⊗ Strongly disagree
- ◯ Disagree
- ◯ No opinion
- ◯ Agree
- ◯ Strongly agree

f. I plan to do long term (>6 months) international work when I finish residency.
- ⊗ Strongly disagree
- ◯ Disagree
- ◯ No opinion
- ◯ Agree
- ◯ Strongly agree

g. I plan to work in health disparities in the following way after residency:

It's an unavoidable part of medicine.

SECTION B: MEDICAL EDUCATION

A. Please indicate your level of agreement with the following statements regarding your medical education and knowledge about immigrants and refugees by checking the box that best represents your opinion.

a. I have received specialized training in immigrant and refugee health, tropical medicine, or cross-cultural health.
- ◯ Strongly disagree
- ⊗ Disagree
- ◯ No opinion
- ◯ Agree
- ◯ Strongly agree

c. I feel comfortable with my fund of knowledge regarding immigrant and refugee health.
- ◯ Strongly disagree
- ◯ Disagree
- ⊗ No opinion
- ◯ Agree
- ◯ Strongly agree

d. I would like to have further training in immigrant and refugee health.
- ◯ Strongly disagree
- ⊗ Disagree
- ◯ No opinion
- ◯ Agree
- ◯ Strongly agree

SECTION C: Attitudes towards immigrant health

A.Please indicate your level of agreement with the following statements regarding immigrant and refugee health by checking the box that best represents your opinion.

a. I enjoy taking care of immigrants and refugees.
- ◯ Never
- ⊗ Rarely
- ◯ Sometimes
- ◯ Usually
- ◯ Always

b. Please indicate the reasons that you enjoy taking care of immigrants and refugees (may choose more than one).
- ☐ Tropical and other conditions not frequently diagnosed in US-born patients
- ☐ Learning about other cultures
- ☐ They don't complain as much
- ☐ Being able to hear their stories
- ☐ Their care is more complicated
- ☐ Their care is less complicated
- ☐ They are very appreciative of your help.
- ☐ They are extremely vulnerable
- ☐ Other:

c. Taking care of immigrants and refugees is more challenging than taking care of US born patients.
- ◯ Never
- ◯ Rarely
- ◯ Sometimes
- ⊗ Usually
- ◯ Always

d. Please mark all the challenges that you face as a provider when providing care to immigrants and refugees (may choose more than one):
- ☒ Language barriers
- ☒ Insurance barriers
- ☒ Cultural barriers
- ☒ Finding a professional interpreter
- ☒ Knowing how to work with a professional interpreter
- ☒ Time constraints
- ☒ My own knowledge related to tropical and travel medicine
- ☒ Transportation problems for the patient
- ☒ Patients not understanding treatment plan
- ☐ Patients not following treatment plan
- ☒ My lack of knowledge regarding the patient's culture
- ☒ Bias or stereotyping
- ☐ Other:

e. Please mark all of the challenges faced by immigrant and refugee populations when receiving healthcare that you have perceived or witnessed (may choose more than one):

- ☒ Language barriers
- ☐ Insurance barriers
- ☐ Cultural barriers
- ☐ Finding a professional interpreter
- ☐ Knowing how to work with a professional interpreter
- ☐ Time constraints
- ☐ Insufficiently trained health care providers
- ☐ Transportation problems for the patient
- ☐ Food insufficiency
- ☐ Need for child care
- ☐ Patients not understanding treatment plan
- ☐ Patients not following treatment plan
- ☐ My lack of knowledge regarding the patient's culture
- ☐ Bias or stereotyping
- ☐ Trust issues
- ☐ Other...

f. Rank how well immigrants and refugees understand the healthcare that you are trying to provide.

- ⊗ Significantly less than a US born individual
- ◯ Less than a US born individual
- ◯ Equivalent to a US born individual
- ◯ More than a US born patient
- ◯ Significantly more than a US born individual

g. Immigrants and refugees adhere to treatment plans and follow my recommendations.

- ◯ Never
- ◯ Rarely
- ◯ Sometimes
- ⊗ Usually
- ◯ Always

h. Immigrants and refugees should receive the same care and insurance coverage as US born patients.

- ◯ Never
- ◯ Rarely
- ⊗ Sometimes
- ◯ Usually
- ◯ Always

i. Immigrants and refugees who are undocumented should receive the same care and insurance coverage as US born patients.

- ⊗ Never
- ◯ Rarely
- ◯ Sometimes
- ◯ Usually
- ◯ Always

j. Every physician is professionally obligated to care for immigrants and refugees if they present to your clinic or hospital.

- ◯ Strongly disagree
- ◯ Disagree
- ⊗ No opinion
- ◯ Agree
- ◯ Strongly agree

k. Is healthcare a human right?

- ◯ Yes
- ⊗ No

B. If you wish, please tell us about what you enjoy or do not enjoy about immigrant and refugee health care and the greatest challenges you face in caring for this population.

____

SECTION D: DEMOGRAPHIC INFORMATION
Please answer the following questions by checking the box in front of the response choice that best describes you.

a. Your age?
- ◯ 20 to 24
- ⊗ 25 to 29
- ◯ 30 to 34
- ◯ 35 to 39
- ◯ 40 or older

b. Your gender?
- ⊗ Female
- ◯ Male
- ◯ Other

c. Are you Hispanic or Latino?
- ◯ Yes
- ⊗ No

d. What is your race? (Select one or more responses)
- ◯ American Indian or Alaska Native
- ◯ Asian (Please specify):
- ◯ Black or African American
- ◯ Native Hawaiian or Other Pacific Islander
- ⊗ White
- ◯ Other (Please specify):

e. Were you born in the United States?
- ⊗ Yes
- ◯ No

g. Your residency year?
- ◯ PGY1
- ⊗ PGY2
- ◯ PGY3
- ◯ PGY4
- ◯ PGY5

h. How would you classify your political ideology?
- ⊗ Conservative
- ◯ Somewhat conservative
- ◯ Moderate
- ◯ Somewhat liberal
- ◯ Liberal
- ◯ Other (Please specify):

i. Estimated level of educational debt?
- ◯ None
- ◯ Less than $50,000
- ◯ $50,000 - $100,000
- ◯ $100,000 - $200,000
- ⊗ $200,000 or more

j. Do you plan to subspecialize?
- ⊗ Yes
- ◯ No

k. Languages spoken?
- ☒ English
- ☐ Spanish
- ☐ French
- ☐ Hmong
- ☐ Somali
- ☐ Japanese
- ☐ Chinese
- ☐ Russian
- ☐ Ethiopian
- ☐ Other ___________

l. Are you in the Global Health Pathway?
- ◯ Yes
- ◯ No

m. Did you earn your degree in the US?

- ⊗ Yes
- ○ No

n. What residency program are you in?

- ○ Internal Medicine
- ○ Med-Peds
- ○ Pediatrics
- ○ Family Practice
- ○ Neurology
- ○ Psychiatry
- ○ ObGyn
- ○ Neurosurgery
- ○ General Surgery
- ○ Orthopedic Surgery
- ○ Urology
- ○ Surgical sub-specialty (please specify in text box below)
- ○ Non-clinical specialty (radiology, pathology; please specify in text box below)

Medical Trainees' attitudes, knowledge, and experience with immigrant and refugee health

Response was added on 12/13/2013 3:00pm.

SECTION A: Personal experience with immigrant and refugee health care.

A. Please indicate your level of agreement with the following statements regarding your personal experience with immigrant and refugee health care by checking the box that best represents your experience.

a. During my inpatient rotations, I take care of the following percentage of immigrant and refugee patients:

- ◯ None
- ◯ 0 -5%
- ◯ 5-10%
- ⊗ 10-25%
- ◯ > 25%

b. During my outpatient rotations, I take care of the following percentage of immigrant and refugee patients:

- ◯ None
- ◯ 0-10%
- ⊗ 10 -25%
- ◯ 25-50%
- ◯ 50-75%
- ◯ >75%

c. I would like to take care of more immigrant and refugee patients.

- ◯ Strongly disagree
- ◯ Disagree
- ⊗ No opinion
- ◯ Agree
- ◯ Strongly agree

d. I plan to take care of immigrants and refugees when I finish residency.

- ◯ Strongly disagree
- ◯ Disagree
- ◯ No opinion
- ⊗ Agree
- ◯ Strongly agree

e. I plan to do short term (< 6 months) international work when I finish residency.

- ◯ Strongly disagree
- ◯ Disagree
- ◯ No opinion
- ◯ Agree
- ⊗ Strongly agree

f. I plan to do long term (>6 months) international work when I finish residency.

- ◯ Strongly disagree
- ⊗ Disagree
- ◯ No opinion
- ◯ Agree
- ◯ Strongly agree

g. I plan to work in health disparities in the following way after residency:

serving abroad, serving MA patients

SECTION B: MEDICAL EDUCATION

A. Please indicate your level of agreement with the following statements regarding your medical education and knowledge about immigrants and refugees by checking the box that best represents your opinion.

a. I have received specialized training in immigrant and refugee health, tropical medicine, or cross-cultural health.

- ◯ Strongly disagree
- ◯ Disagree
- ◯ No opinion
- ◯ Agree
- ⊗ Strongly agree

b. If you have received specialized training in immigrant and refugee health, tropical medicine, or cross-cultural health, please indicate all the contexts in which you received this training:

- ☐ As an undergraduate.
- ☒ As a medical student.
- ☐ As part of my residency.
- ☒ A special program.
- ☐ As part of my fellowship.
- ☐ As part of a degree program (e.g. MPH)
- ☐ Other:

c. I feel comfortable with my fund of knowledge regarding immigrant and refugee health.

- ◯ Strongly disagree
- ◯ Disagree
- ◯ No opinion
- ⊗ Agree
- ◯ Strongly agree

d. I would like to have further training in immigrant and refugee health.

- ◯ Strongly disagree
- ◯ Disagree
- ◯ No opinion
- ⊗ Agree
- ◯ Strongly agree

e. If you agree with the above, please indicate all the contexts in which you would like to receive this training:
▯▯

- ☒ As part of my residency.
- ☒ A special program.
- ☐ As part of my fellowship.

SECTION C: Attitudes towards immigrant health

A.▯Please indicate your level of agreement with the following statements regarding immigrant and refugee health by checking the box that best represents your opinion.

a. I enjoy taking care of immigrants and refugees.

- ◯ Never
- ◯ Rarely
- ◯ Sometimes
- ⊗ Usually
- ◯ Always

b. Please indicate the reasons that you enjoy taking care of immigrants and refugees (may choose more than one).

- ☒ Tropical and other conditions not frequently diagnosed in US-born patients
- ☒ Learning about other cultures
- ☐ They don't complain as much
- ☒ Being able to hear their stories
- ☒ Their care is more complicated
- ☐ Their care is less complicated
- ☐ They are very appreciative of your help.
- ☒ They are extremely vulnerable
- ☐ Other:

c. Taking care of immigrants and refugees is more challenging than taking care of US born patients.

- ◯ Never
- ◯ Rarely
- ◯ Sometimes
- ⊗ Usually
- ◯ Always

d. Please mark all the challenges that you face as a provider when providing care to immigrants and refugees (may choose more than one):

- [x] Language barriers
- [x] Insurance barriers
- [x] Cultural barriers
- [ ] Finding a professional interpreter
- [ ] Knowing how to work with a professional interpreter
- [x] Time constraints
- [ ] My own knowledge related to tropical and travel medicine
- [x] Transportation problems for the patient
- [x] Patients not understanding treatment plan
- [x] Patients not following treatment plan
- [ ] My lack of knowledge regarding the patient's culture
- [x] Bias or stereotyping
- [ ] Other:

e. Please mark all of the challenges faced by immigrant and refugee populations when receiving healthcare that you have perceived or witnessed (may choose more than one):

- [x] Language barriers
- [x] Insurance barriers
- [x] Cultural barriers
- [ ] Finding a professional interpreter
- [ ] Knowing how to work with a professional interpreter
- [ ] Time constraints
- [x] Insufficiently trained health care providers
- [x] Transportation problems for the patient
- [x] Food insufficiency
- [x] Need for child care
- [x] Patients not understanding treatment plan
- [x] Patients not following treatment plan
- [ ] My lack of knowledge regarding the patient's culture
- [x] Bias or stereotyping
- [x] Trust issues
- [ ] Other...

f. Rank how well immigrants and refugees understand the healthcare that you are trying to provide.

- ( ) Significantly less than a US born individual
- (x) Less than a US born individual
- ( ) Equivalent to a US born individual
- ( ) More than a US born patient
- ( ) Significantly more than a US born individual

g. Immigrants and refugees adhere to treatment plans and follow my recommendations.

- ( ) Never
- ( ) Rarely
- (x) Sometimes
- ( ) Usually
- ( ) Always

h. Immigrants and refugees should receive the same care and insurance coverage as US born patients.

- ( ) Never
- ( ) Rarely
- ( ) Sometimes
- ( ) Usually
- (x) Always

i. Immigrants and refugees who are undocumented should receive the same care and insurance coverage as US born patients.

- ( ) Never
- ( ) Rarely
- ( ) Sometimes
- ( ) Usually
- (x) Always

j. Every physician is professionally obligated to care for immigrants and refugees if they present to your clinic or hospital.

- ( ) Strongly disagree
- ( ) Disagree
- ( ) No opinion
- ( ) Agree
- (x) Strongly agree

k. Is healthcare a human right?
- ⊗ Yes
- ◯ No

B. If you wish, please tell us about what you enjoy or do not enjoy about immigrant and refugee health care and the greatest challenges you face in caring for this population.

____

SECTION D: DEMOGRAPHIC INFORMATION
Please answer the following questions by checking the box in front of the response choice that best describes you.

a. Your age?
- ◯ 20 to 24
- ◯ 25 to 29
- ⊗ 30 to 34
- ◯ 35 to 39
- ◯ 40 or older

b. Your gender?
- ⊗ Female
- ◯ Male
- ◯ Other

c.Are you Hispanic or Latino?
- ◯ Yes
- ⊗ No

d. What is your race? (Select one or more responses)
- ◯ American Indian or Alaska Native
- ◯ Asian (Please specify):
- ◯ Black or African American
- ◯ Native Hawaiian or Other Pacific Islander
- ⊗ White
- ◯ Other (Please specify):

e.Were you born in the United States?
- ⊗ Yes
- ◯ No

g. Your residency year?
- ◯ PGY1
- ◯ PGY2
- ⊗ PGY3
- ◯ PGY4
- ◯ PGY5

h. How would you classify your political ideology?
- ◯ Conservative
- ◯ Somewhat conservative
- ◯ Moderate
- ⊗ Somewhat liberal
- ◯ Liberal
- ◯ Other (Please specify):

i. Estimated level of educational debt?
- ◯ None
- ◯ Less than $50,000
- ⊗ $50,000 - $100,000
- ◯ $100,000 - $200,000
- ◯ $200,000 or more

j.Do you plan to subspecialize?
- ◯ Yes
- ⊗ No

k. Languages spoken?

- ☒ English
- ☒ Spanish
- ☐ French
- ☐ Hmong
- ☐ Somali
- ☐ Japanese
- ☐ Chinese
- ☐ Russian
- ☐ Ethiopian
- ☐ Other ___________

l. Are you in the Global Health Pathway?

- ⊗ Yes
- ◯ No

m. Did you earn your degree in the US?

- ⊗ Yes
- ◯ No

n. What residency program are you in?

- ◯ Internal Medicine
- ◯ Med-Peds
- ◯ Pediatrics
- ⊗ Family Practice
- ◯ Neurology
- ◯ Psychiatry
- ◯ ObGyn
- ◯ Neurosurgery
- ◯ General Surgery
- ◯ Orthopedic Surgery
- ◯ Urology
- ◯ Surgical sub-specialty (please specify in text box below)
- ◯ Non-clinical specialty (radiology, pathology; please specify in text box below)

Medical Trainees' attitudes, knowledge, and experience with immigrant and refugee health

Response was added on 12/13/2013 3:14pm.

SECTION A: Personal experience with immigrant and refugee health care.

A. Please indicate your level of agreement with the following statements regarding your personal experience with immigrant and refugee health care by checking the box that best represents your experience.

a. During my inpatient rotations, I take care of the following percentage of immigrant and refugee patients:
- ◯ None
- ◯ 0 -5%
- ◯ 5-10%
- ⊗ 10-25%
- ◯ > 25%

b. During my outpatient rotations, I take care of the following percentage of immigrant and refugee patients:
- ◯ None
- ◯ 0-10%
- ◯ 10 -25%
- ⊗ 25-50%
- ◯ 50-75%
- ◯ >75%

c. I would like to take care of more immigrant and refugee patients.
- ◯ Strongly disagree
- ◯ Disagree
- ⊗ No opinion
- ◯ Agree
- ◯ Strongly agree

d. I plan to take care of immigrants and refugees when I finish residency.
- ◯ Strongly disagree
- ◯ Disagree
- ◯ No opinion
- ⊗ Agree
- ◯ Strongly agree

e. I plan to do short term (< 6 months) international work when I finish residency.
- ⊗ Strongly disagree
- ◯ Disagree
- ◯ No opinion
- ◯ Agree
- ◯ Strongly agree

f. I plan to do long term (>6 months) international work when I finish residency.
- ⊗ Strongly disagree
- ◯ Disagree
- ◯ No opinion
- ◯ Agree
- ◯ Strongly agree

g. I plan to work in health disparities in the following way after residency:

I'll try and work at an urban hospital setting, but I have no specific plans or goals to specifically work with immigrants/refugee's.

SECTION B: MEDICAL EDUCATION

A. Please indicate your level of agreement with the following statements regarding your medical education and knowledge about immigrants and refugees by checking the box that best represents your opinion.

a. I have received specialized training in immigrant and refugee health, tropical medicine, or cross-cultural health.

- ⊗ Strongly disagree
- ○ Disagree
- ○ No opinion
- ○ Agree
- ○ Strongly agree

c. I feel comfortable with my fund of knowledge regarding immigrant and refugee health.

- ○ Strongly disagree
- ⊗ Disagree
- ○ No opinion
- ○ Agree
- ○ Strongly agree

d. I would like to have further training in immigrant and refugee health.

- ○ Strongly disagree
- ⊗ Disagree
- ○ No opinion
- ○ Agree
- ○ Strongly agree

SECTION C: Attitudes towards immigrant health

A.Please indicate your level of agreement with the following statements regarding immigrant and refugee health by checking the box that best represents your opinion.

a. I enjoy taking care of immigrants and refugees.

- ○ Never
- ○ Rarely
- ⊗ Sometimes
- ○ Usually
- ○ Always

b. Please indicate the reasons that you enjoy taking care of immigrants and refugees (may choose more than one).

- ☐ Tropical and other conditions not frequently diagnosed in US-born patients
- ☐ Learning about other cultures
- ☐ They don't complain as much
- ☐ Being able to hear their stories
- ☐ Their care is more complicated
- ☐ Their care is less complicated
- ☐ They are very appreciative of your help.
- ☐ They are extremely vulnerable
- ☒ Other:

I enjoy taking care of all my patients

c. Taking care of immigrants and refugees is more challenging than taking care of US born patients.

- ○ Never
- ○ Rarely
- ⊗ Sometimes
- ○ Usually
- ○ Always

d. Please mark all the challenges that you face as a provider when providing care to immigrants and refugees (may choose more than one):

- ☒ Language barriers
- ☐ Insurance barriers
- ☐ Cultural barriers
- ☒ Finding a professional interpreter
- ☐ Knowing how to work with a professional interpreter
- ☐ Time constraints
- ☐ My own knowledge related to tropical and travel medicine
- ☐ Transportation problems for the patient
- ☐ Patients not understanding treatment plan
- ☐ Patients not following treatment plan
- ☐ My lack of knowledge regarding the patient's culture
- ☐ Bias or stereotyping
- ☐ Other:

e. Please mark all of the challenges faced by immigrant and refugee populations when receiving healthcare that you have perceived or witnessed (may choose more than one):

- ☒ Language barriers
- ☒ Insurance barriers
- ☐ Cultural barriers
- ☒ Finding a professional interpreter
- ☒ Knowing how to work with a professional interpreter
- ☒ Time constraints
- ☐ Insufficiently trained health care providers
- ☐ Transportation problems for the patient
- ☐ Food insufficiency
- ☐ Need for child care
- ☒ Patients not understanding treatment plan
- ☐ Patients not following treatment plan
- ☐ My lack of knowledge regarding the patient's culture
- ☐ Bias or stereotyping
- ☐ Trust issues
- ☐ Other...

f. Rank how well immigrants and refugees understand the healthcare that you are trying to provide.

- ○ Significantly less than a US born individual
- ○ Less than a US born individual
- ⊗ Equivalent to a US born individual
- ○ More than a US born patient
- ○ Significantly more than a US born individual

g. Immigrants and refugees adhere to treatment plans and follow my recommendations.

- ○ Never
- ○ Rarely
- ○ Sometimes
- ⊗ Usually
- ○ Always

h. Immigrants and refugees should receive the same care and insurance coverage as US born patients.

- ○ Never
- ○ Rarely
- ○ Sometimes
- ⊗ Usually
- ○ Always

i. Immigrants and refugees who are undocumented should receive the same care and insurance coverage as US born patients.

- ○ Never
- ○ Rarely
- ○ Sometimes
- ⊗ Usually
- ○ Always

j. Every physician is professionally obligated to care for immigrants and refugees if they present to your clinic or hospital.

- ○ Strongly disagree
- ○ Disagree
- ○ No opinion
- ○ Agree
- ⊗ Strongly agree

k. Is healthcare a human right?

- ⊗ Yes
- ◯ No

B. If you wish, please tell us about what you enjoy or do not enjoy about immigrant and refugee health care and the greatest challenges you face in caring for this population.

____

SECTION D: DEMOGRAPHIC INFORMATION
Please answer the following questions by checking the box in front of the response choice that best describes you.

a. Your age?

- ◯ 20 to 24
- ◯ 25 to 29
- ◯ 30 to 34
- ⊗ 35 to 39
- ◯ 40 or older

b. Your gender?

- ◯ Female
- ⊗ Male
- ◯ Other

c.Are you Hispanic or Latino?

- ◯ Yes
- ⊗ No

d. What is your race? (Select one or more responses)

- ◯ American Indian or Alaska Native
- ◯ Asian (Please specify):
- ◯ Black or African American
- ◯ Native Hawaiian or Other Pacific Islander
- ⊗ White
- ◯ Other (Please specify):

e.Were you born in the United States?

- ⊗ Yes
- ◯ No

g. Your residency year?

- ◯ PGY1
- ⊗ PGY2
- ◯ PGY3
- ◯ PGY4
- ◯ PGY5

h. How would you classify your political ideology?

- ◯ Conservative
- ◯ Somewhat conservative
- ◯ Moderate
- ◯ Somewhat liberal
- ⊗ Liberal
- ◯ Other (Please specify):

i. Estimated level of educational debt?

- ◯ None
- ◯ Less than $50,000
- ◯ $50,000 - $100,000
- ◯ $100,000 - $200,000
- ⊗ $200,000 or more

j.Do you plan to subspecialize?

- ⊗ Yes
- ◯ No

k. Languages spoken?

- ☒ English
- ☐ Spanish
- ☐ French
- ☐ Hmong
- ☐ Somali
- ☐ Japanese
- ☐ Chinese
- ☒ Russian
- ☐ Ethiopian
- ☐ Other ___________

l. Are you in the Global Health Pathway?

- ◯ Yes
- ⊗ No

m. Did you earn your degree in the US?

- ⊗ Yes
- ◯ No

n. What residency program are you in?

- ◯ Internal Medicine
- ◯ Med-Peds
- ⊗ Pediatrics
- ◯ Family Practice
- ◯ Neurology
- ◯ Psychiatry
- ◯ ObGyn
- ◯ Neurosurgery
- ◯ General Surgery
- ◯ Orthopedic Surgery
- ◯ Urology
- ◯ Surgical sub-specialty (please specify in text box below)
- ◯ Non-clinical specialty (radiology, pathology; please specify in text box below)

Medical Trainees' attitudes, knowledge, and experience with immigrant and refugee health

Response was added on 12/13/2013 3:51pm.

SECTION A: Personal experience with immigrant and refugee health care.

A. Please indicate your level of agreement with the following statements regarding your personal experience with immigrant and refugee health care by checking the box that best represents your experience.

a. During my inpatient rotations, I take care of the following percentage of immigrant and refugee patients:	◯ None<br>⊗ 0 -5%<br>◯ 5-10%<br>◯ 10-25%<br>◯ > 25%
b. During my outpatient rotations, I take care of the following percentage of immigrant and refugee patients:	◯ None<br>◯ 0-10%<br>⊗ 10 -25%<br>◯ 25-50%<br>◯ 50-75%<br>◯ >75%
c. I would like to take care of more immigrant and refugee patients.	◯ Strongly disagree<br>◯ Disagree<br>⊗ No opinion<br>◯ Agree<br>◯ Strongly agree
d. I plan to take care of immigrants and refugees when I finish residency.	◯ Strongly disagree<br>◯ Disagree<br>⊗ No opinion<br>◯ Agree<br>◯ Strongly agree
e. I plan to do short term (< 6 months) international work when I finish residency.	◯ Strongly disagree<br>⊗ Disagree<br>◯ No opinion<br>◯ Agree<br>◯ Strongly agree
f. I plan to do long term (>6 months) international work when I finish residency.	⊗ Strongly disagree<br>◯ Disagree<br>◯ No opinion<br>◯ Agree<br>◯ Strongly agree
g. I plan to work in health disparities in the following way after residency:	Rural under served Midwest

SECTION B: MEDICAL EDUCATION

A. Please indicate your level of agreement with the following statements regarding your medical education and knowledge about immigrants and refugees by checking the box that best represents your opinion.

a. I have received specialized training in immigrant and refugee health, tropical medicine, or cross-cultural health.

- ◯ Strongly disagree
- ◯ Disagree
- ◯ No opinion
- ⊗ Agree
- ◯ Strongly agree

b. If you have received specialized training in immigrant and refugee health, tropical medicine, or cross-cultural health, please indicate all the contexts in which you received this training:

- ☐ As an undergraduate.
- ☒ As a medical student.
- ☐ As part of my residency.
- ☐ A special program.
- ☐ As part of my fellowship.
- ☐ As part of a degree program (e.g. MPH)
- ☐ Other:

c. I feel comfortable with my fund of knowledge regarding immigrant and refugee health.

- ◯ Strongly disagree
- ◯ Disagree
- ⊗ No opinion
- ◯ Agree
- ◯ Strongly agree

d. I would like to have further training in immigrant and refugee health.

- ◯ Strongly disagree
- ◯ Disagree
- ⊗ No opinion
- ◯ Agree
- ◯ Strongly agree

SECTION C: Attitudes towards immigrant health

A.Please indicate your level of agreement with the following statements regarding immigrant and refugee health by checking the box that best represents your opinion.

a. I enjoy taking care of immigrants and refugees.

- ◯ Never
- ◯ Rarely
- ⊗ Sometimes
- ◯ Usually
- ◯ Always

b. Please indicate the reasons that you enjoy taking care of immigrants and refugees (may choose more than one).

- ☐ Tropical and other conditions not frequently diagnosed in US-born patients
- ☒ Learning about other cultures
- ☐ They don't complain as much
- ☐ Being able to hear their stories
- ☐ Their care is more complicated
- ☐ Their care is less complicated
- ☐ They are very appreciative of your help.
- ☐ They are extremely vulnerable
- ☐ Other:

c. Taking care of immigrants and refugees is more challenging than taking care of US born patients.

- ◯ Never
- ◯ Rarely
- ◯ Sometimes
- ⊗ Usually
- ◯ Always

d. Please mark all the challenges that you face as a provider when providing care to immigrants and refugees (may choose more than one):

- ☒ Language barriers
- ☐ Insurance barriers
- ☐ Cultural barriers
- ☒ Finding a professional interpreter
- ☐ Knowing how to work with a professional interpreter
- ☒ Time constraints
- ☐ My own knowledge related to tropical and travel medicine
- ☐ Transportation problems for the patient
- ☐ Patients not understanding treatment plan
- ☐ Patients not following treatment plan
- ☐ My lack of knowledge regarding the patient's culture
- ☐ Bias or stereotyping
- ☐ Other:

e. Please mark all of the challenges faced by immigrant and refugee populations when receiving healthcare that you have perceived or witnessed (may choose more than one):

- ☒ Language barriers
- ☒ Insurance barriers
- ☒ Cultural barriers
- ☐ Finding a professional interpreter
- ☐ Knowing how to work with a professional interpreter
- ☐ Time constraints
- ☐ Insufficiently trained health care providers
- ☒ Transportation problems for the patient
- ☐ Food insufficiency
- ☐ Need for child care
- ☒ Patients not understanding treatment plan
- ☐ Patients not following treatment plan
- ☐ My lack of knowledge regarding the patient's culture
- ☐ Bias or stereotyping
- ☒ Trust issues
- ☐ Other...

f. Rank how well immigrants and refugees understand the healthcare that you are trying to provide.

- ○ Significantly less than a US born individual
- ⊗ Less than a US born individual
- ○ Equivalent to a US born individual
- ○ More than a US born patient
- ○ Significantly more than a US born individual

g. Immigrants and refugees adhere to treatment plans and follow my recommendations.

- ○ Never
- ○ Rarely
- ○ Sometimes
- ⊗ Usually
- ○ Always

h. Immigrants and refugees should receive the same care and insurance coverage as US born patients.

- ○ Never
- ○ Rarely
- ○ Sometimes
- ○ Usually
- ⊗ Always

i. Immigrants and refugees who are undocumented should receive the same care and insurance coverage as US born patients.

- ○ Never
- ○ Rarely
- ⊗ Sometimes
- ○ Usually
- ○ Always

j. Every physician is professionally obligated to care for immigrants and refugees if they present to your clinic or hospital.

- ○ Strongly disagree
- ○ Disagree
- ○ No opinion
- ⊗ Agree
- ○ Strongly agree

k. Is healthcare a human right?
- ⊗ Yes
- ◯ No

B. If you wish, please tell us about what you enjoy or do not enjoy about immigrant and refugee health care and the greatest challenges you face in caring for this population.

____

SECTION D: DEMOGRAPHIC INFORMATION
Please answer the following questions by checking the box in front of the response choice that best describes you.

a. Your age?
- ◯ 20 to 24
- ⊗ 25 to 29
- ◯ 30 to 34
- ◯ 35 to 39
- ◯ 40 or older

b. Your gender?
- ⊗ Female
- ◯ Male
- ◯ Other

c. Are you Hispanic or Latino?
- ◯ Yes
- ⊗ No

d. What is your race? (Select one or more responses)
- ◯ American Indian or Alaska Native
- ◯ Asian (Please specify):
- ◯ Black or African American
- ⊗ Native Hawaiian or Other Pacific Islander
- ◯ White
- ◯ Other (Please specify):

e. Were you born in the United States?
- ⊗ Yes
- ◯ No

g. Your residency year?
- ⊗ PGY1
- ◯ PGY2
- ◯ PGY3
- ◯ PGY4
- ◯ PGY5

h. How would you classify your political ideology?
- ◯ Conservative
- ◯ Somewhat conservative
- ⊗ Moderate
- ◯ Somewhat liberal
- ◯ Liberal
- ◯ Other (Please specify):

i. Estimated level of educational debt?
- ◯ None
- ⊗ Less than $50,000
- ◯ $50,000 - $100,000
- ◯ $100,000 - $200,000
- ◯ $200,000 or more

j. Do you plan to subspecialize?
- ◯ Yes
- ⊗ No

k. Languages spoken?

- ☒ English
- ☐ Spanish
- ☐ French
- ☐ Hmong
- ☐ Somali
- ☐ Japanese
- ☐ Chinese
- ☐ Russian
- ☐ Ethiopian
- ☐ Other ___________

l. Are you in the Global Health Pathway?

- ◯ Yes
- ⊗ No

m. Did you earn your degree in the US?

- ⊗ Yes
- ◯ No

n. What residency program are you in?

- ◯ Internal Medicine
- ◯ Med-Peds
- ◯ Pediatrics
- ⊗ Family Practice
- ◯ Neurology
- ◯ Psychiatry
- ◯ ObGyn
- ◯ Neurosurgery
- ◯ General Surgery
- ◯ Orthopedic Surgery
- ◯ Urology
- ◯ Surgical sub-specialty (please specify in text box below)
- ◯ Non-clinical specialty (radiology, pathology; please specify in text box below)

Medical Trainees' attitudes, knowledge, and experience with immigrant and refugee health

Response was added on 12/13/2013 7:07pm.

SECTION A: Personal experience with immigrant and refugee health care.

A. Please indicate your level of agreement with the following statements regarding your personal experience with immigrant and refugee health care by checking the box that best represents your experience.

a. During my inpatient rotations, I take care of the following percentage of immigrant and refugee patients:

- ◯ None
- ◯ 0 -5%
- ◯ 5-10%
- ◯ 10-25%
- ◯ > 25%

b. During my outpatient rotations, I take care of the following percentage of immigrant and refugee patients:

- ◯ None
- ◯ 0-10%
- ⊗ 10 -25%
- ◯ 25-50%
- ◯ 50-75%
- ◯ >75%

c. I would like to take care of more immigrant and refugee patients.

- ◯ Strongly disagree
- ◯ Disagree
- ◯ No opinion
- ⊗ Agree
- ◯ Strongly agree

d. I plan to take care of immigrants and refugees when I finish residency.

- ◯ Strongly disagree
- ◯ Disagree
- ◯ No opinion
- ⊗ Agree
- ◯ Strongly agree

e. I plan to do short term (< 6 months) international work when I finish residency.

- ◯ Strongly disagree
- ◯ Disagree
- ◯ No opinion
- ⊗ Agree
- ◯ Strongly agree

f. I plan to do long term (>6 months) international work when I finish residency.

- ◯ Strongly disagree
- ◯ Disagree
- ⊗ No opinion
- ◯ Agree
- ◯ Strongly agree

g. I plan to work in health disparities in the following way after residency:

Volunteer at free clinics, volunteer abroad.

SECTION B: MEDICAL EDUCATION

A. Please indicate your level of agreement with the following statements regarding your medical education and knowledge about immigrants and refugees by checking the box that best represents your opinion.

a. I have received specialized training in immigrant and refugee health, tropical medicine, or cross-cultural health.

- ◯ Strongly disagree
- ⊗ Disagree
- ◯ No opinion
- ◯ Agree
- ◯ Strongly agree

c. I feel comfortable with my fund of knowledge regarding immigrant and refugee health.

- ◯ Strongly disagree
- ⊗ Disagree
- ◯ No opinion
- ◯ Agree
- ◯ Strongly agree

d. I would like to have further training in immigrant and refugee health.

- ◯ Strongly disagree
- ◯ Disagree
- ◯ No opinion
- ⊗ Agree
- ◯ Strongly agree

e. If you agree with the above, please indicate all the contexts in which you would like to receive this training:

- ☒ As part of my residency.
- ☒ A special program.
- ☐ As part of my fellowship.

SECTION C: Attitudes towards immigrant health

A. Please indicate your level of agreement with the following statements regarding immigrant and refugee health by checking the box that best represents your opinion.

a. I enjoy taking care of immigrants and refugees.

- ◯ Never
- ◯ Rarely
- ◯ Sometimes
- ⊗ Usually
- ◯ Always

b. Please indicate the reasons that you enjoy taking care of immigrants and refugees (may choose more than one).

- ☒ Tropical and other conditions not frequently diagnosed in US-born patients
- ☒ Learning about other cultures
- ☐ They don't complain as much
- ☐ Being able to hear their stories
- ☐ Their care is more complicated
- ☐ Their care is less complicated
- ☐ They are very appreciative of your help.
- ☒ They are extremely vulnerable
- ☐ Other:

c. Taking care of immigrants and refugees is more challenging than taking care of US born patients.

- ◯ Never
- ◯ Rarely
- ⊗ Sometimes
- ◯ Usually
- ◯ Always

d. Please mark all the challenges that you face as a provider when providing care to immigrants and refugees (may choose more than one):

- ☒ Language barriers
- ☒ Insurance barriers
- ☐ Cultural barriers
- ☒ Finding a professional interpreter
- ☐ Knowing how to work with a professional interpreter
- ☐ Time constraints
- ☐ My own knowledge related to tropical and travel medicine
- ☐ Transportation problems for the patient
- ☐ Patients not understanding treatment plan
- ☐ Patients not following treatment plan
- ☐ My lack of knowledge regarding the patient's culture
- ☐ Bias or stereotyping
- ☐ Other:

e. Please mark all of the challenges faced by immigrant and refugee populations when receiving healthcare that you have perceived or witnessed (may choose more than one):

- ☒ Language barriers
- ☒ Insurance barriers
- ☐ Cultural barriers
- ☐ Finding a professional interpreter
- ☒ Knowing how to work with a professional interpreter
- ☐ Time constraints
- ☐ Insufficiently trained health care providers
- ☒ Transportation problems for the patient
- ☐ Food insufficiency
- ☐ Need for child care
- ☐ Patients not understanding treatment plan
- ☐ Patients not following treatment plan
- ☐ My lack of knowledge regarding the patient's culture
- ☒ Bias or stereotyping
- ☐ Trust issues
- ☐ Other...

f. Rank how well immigrants and refugees understand the healthcare that you are trying to provide.

- ○ Significantly less than a US born individual
- ○ Less than a US born individual
- ⊗ Equivalent to a US born individual
- ○ More than a US born patient
- ○ Significantly more than a US born individual

g. Immigrants and refugees adhere to treatment plans and follow my recommendations.

- ○ Never
- ○ Rarely
- ○ Sometimes
- ⊗ Usually
- ○ Always

h. Immigrants and refugees should receive the same care and insurance coverage as US born patients.

- ○ Never
- ○ Rarely
- ○ Sometimes
- ○ Usually
- ⊗ Always

i. Immigrants and refugees who are undocumented should receive the same care and insurance coverage as US born patients.

- ○ Never
- ○ Rarely
- ○ Sometimes
- ○ Usually
- ⊗ Always

j. Every physician is professionally obligated to care for immigrants and refugees if they present to your clinic or hospital.

- ○ Strongly disagree
- ○ Disagree
- ○ No opinion
- ○ Agree
- ⊗ Strongly agree

k. Is healthcare a human right?	⊗ Yes<br>◯ No
B. If you wish, please tell us about what you enjoy or do not enjoy about immigrant and refugee health care and the greatest challenges you face in caring for this population.	I like working with immigrant populations, although they do take more time, but it's often rewarding. I have run into problems with the interpreters being racist / biased against their own people, however, which is very worrisome, and I cannot control! I hate that I don't know exactly what the interpreter is telling the patient!

SECTION D: DEMOGRAPHIC INFORMATION
Please answer the following questions by checking the box in front of the response choice that best describes you.

a. Your age?	◯ 20 to 24<br>⊗ 25 to 29<br>◯ 30 to 34<br>◯ 35 to 39<br>◯ 40 or older
b. Your gender?	⊗ Female<br>◯ Male<br>◯ Other
c. Are you Hispanic or Latino?	◯ Yes<br>⊗ No
d. What is your race? (Select one or more responses)	◯ American Indian or Alaska Native<br>◯ Asian (Please specify):<br>◯ Black or African American<br>◯ Native Hawaiian or Other Pacific Islander<br>⊗ White<br>◯ Other (Please specify):
e. Were you born in the United States?	⊗ Yes<br>◯ No
g. Your residency year?	◯ PGY1<br>◯ PGY2<br>⊗ PGY3<br>◯ PGY4<br>◯ PGY5
h. How would you classify your political ideology?	◯ Conservative<br>◯ Somewhat conservative<br>◯ Moderate<br>◯ Somewhat liberal<br>⊗ Liberal<br>◯ Other (Please specify):
i. Estimated level of educational debt?	◯ None<br>◯ Less than $50,000<br>◯ $50,000 - $100,000<br>⊗ $100,000 - $200,000<br>◯ $200,000 or more
j. Do you plan to subspecialize?	◯ Yes<br>⊗ No

k. Languages spoken?

- [x] English
- [ ] Spanish
- [ ] French
- [ ] Hmong
- [ ] Somali
- [ ] Japanese
- [ ] Chinese
- [ ] Russian
- [ ] Ethiopian
- [ ] Other ___________

l. Are you in the Global Health Pathway?

- ◯ Yes
- ⊗ No

m. Did you earn your degree in the US?

- ◯ Yes
- ◯ No

n. What residency program are you in?

- ◯ Internal Medicine
- ◯ Med-Peds
- ◯ Pediatrics
- ◯ Family Practice
- ◯ Neurology
- ◯ Psychiatry
- ◯ ObGyn
- ◯ Neurosurgery
- ◯ General Surgery
- ◯ Orthopedic Surgery
- ◯ Urology
- ⊗ Surgical sub-specialty (please specify in text box below)
- ◯ Non-clinical specialty (radiology, pathology; please specify in text box below)

Dermatology!! (Where is it on the list?!)

Medical Trainees' attitudes, knowledge, and experience with immigrant and refugee health

Response was added on 12/13/2013 7:58pm.

SECTION A: Personal experience with immigrant and refugee health care.

A. Please indicate your level of agreement with the following statements regarding your personal experience with immigrant and refugee health care by checking the box that best represents your experience.

a. During my inpatient rotations, I take care of the following percentage of immigrant and refugee patients:

- ◯ None
- ◯ 0 -5%
- ⊗ 5-10%
- ◯ 10-25%
- ◯ > 25%

b. During my outpatient rotations, I take care of the following percentage of immigrant and refugee patients:

- ⊗ None
- ◯ 0-10%
- ◯ 10 -25%
- ◯ 25-50%
- ◯ 50-75%
- ◯ >75%

c. I would like to take care of more immigrant and refugee patients.

- ◯ Strongly disagree
- ◯ Disagree
- ⊗ No opinion
- ◯ Agree
- ◯ Strongly agree

d. I plan to take care of immigrants and refugees when I finish residency.

- ◯ Strongly disagree
- ◯ Disagree
- ⊗ No opinion
- ◯ Agree
- ◯ Strongly agree

e. I plan to do short term (< 6 months) international work when I finish residency.

- ◯ Strongly disagree
- ◯ Disagree
- ◯ No opinion
- ⊗ Agree
- ◯ Strongly agree

f. I plan to do long term (>6 months) international work when I finish residency.

- ◯ Strongly disagree
- ⊗ Disagree
- ◯ No opinion
- ◯ Agree
- ◯ Strongly agree

g. I plan to work in health disparities in the following way after residency:

international health education work in short term

SECTION B: MEDICAL EDUCATION

A. Please indicate your level of agreement with the following statements regarding your medical education and knowledge about immigrants and refugees by checking the box that best represents your opinion.

a. I have received specialized training in immigrant and refugee health, tropical medicine, or cross-cultural health.

- ◯ Strongly disagree
- ⊗ Disagree
- ◯ No opinion
- ◯ Agree
- ◯ Strongly agree

c. I feel comfortable with my fund of knowledge regarding immigrant and refugee health.

- ◯ Strongly disagree
- ⊗ Disagree
- ◯ No opinion
- ◯ Agree
- ◯ Strongly agree

d. I would like to have further training in immigrant and refugee health.

- ◯ Strongly disagree
- ◯ Disagree
- ⊗ No opinion
- ◯ Agree
- ◯ Strongly agree

SECTION C: Attitudes towards immigrant health

A.Please indicate your level of agreement with the following statements regarding immigrant and refugee health by checking the box that best represents your opinion.

a. I enjoy taking care of immigrants and refugees.

- ◯ Never
- ◯ Rarely
- ⊗ Sometimes
- ◯ Usually
- ◯ Always

b. Please indicate the reasons that you enjoy taking care of immigrants and refugees (may choose more than one).

- ☐ Tropical and other conditions not frequently diagnosed in US-born patients
- ☐ Learning about other cultures
- ☐ They don't complain as much
- ☐ Being able to hear their stories
- ☐ Their care is more complicated
- ☐ Their care is less complicated
- ☒ They are very appreciative of your help.
- ☐ They are extremely vulnerable
- ☐ Other:

c. Taking care of immigrants and refugees is more challenging than taking care of US born patients.

- ◯ Never
- ◯ Rarely
- ◯ Sometimes
- ◯ Usually
- ⊗ Always

d. Please mark all the challenges that you face as a provider when providing care to immigrants and refugees (may choose more than one):

- ☒ Language barriers
- ☐ Insurance barriers
- ☒ Cultural barriers
- ☒ Finding a professional interpreter
- ☐ Knowing how to work with a professional interpreter
- ☒ Time constraints
- ☐ My own knowledge related to tropical and travel medicine
- ☒ Transportation problems for the patient
- ☐ Patients not understanding treatment plan
- ☐ Patients not following treatment plan
- ☐ My lack of knowledge regarding the patient's culture
- ☐ Bias or stereotyping
- ☐ Other:

e. Please mark all of the challenges faced by immigrant and refugee populations when receiving healthcare that you have perceived or witnessed (may choose more than one):

- [x] Language barriers
- [ ] Insurance barriers
- [x] Cultural barriers
- [ ] Finding a professional interpreter
- [ ] Knowing how to work with a professional interpreter
- [ ] Time constraints
- [ ] Insufficiently trained health care providers
- [ ] Transportation problems for the patient
- [ ] Food insufficiency
- [ ] Need for child care
- [x] Patients not understanding treatment plan
- [x] Patients not following treatment plan
- [ ] My lack of knowledge regarding the patient's culture
- [ ] Bias or stereotyping
- [ ] Trust issues
- [ ] Other...

f. Rank how well immigrants and refugees understand the healthcare that you are trying to provide.

- ◯ Significantly less than a US born individual
- ⊗ Less than a US born individual
- ◯ Equivalent to a US born individual
- ◯ More than a US born patient
- ◯ Significantly more than a US born individual

g. Immigrants and refugees adhere to treatment plans and follow my recommendations.

- ◯ Never
- ◯ Rarely
- ◯ Sometimes
- ⊗ Usually
- ◯ Always

h. Immigrants and refugees should receive the same care and insurance coverage as US born patients.

- ◯ Never
- ◯ Rarely
- ◯ Sometimes
- ◯ Usually
- ⊗ Always

i. Immigrants and refugees who are undocumented should receive the same care and insurance coverage as US born patients.

- ◯ Never
- ◯ Rarely
- ◯ Sometimes
- ◯ Usually
- ⊗ Always

j. Every physician is professionally obligated to care for immigrants and refugees if they present to your clinic or hospital.

- ◯ Strongly disagree
- ◯ Disagree
- ◯ No opinion
- ◯ Agree
- ⊗ Strongly agree

k. Is healthcare a human right?

- ◯ Yes
- ⊗ No

B. If you wish, please tell us about what you enjoy or do not enjoy about immigrant and refugee health care and the greatest challenges you face in caring for this population.

____

SECTION D: DEMOGRAPHIC INFORMATION
Please answer the following questions by checking the box in front of the response choice that best describes you.

a. Your age?

- ◯ 20 to 24
- ⊗ 25 to 29
- ◯ 30 to 34
- ◯ 35 to 39
- ◯ 40 or older

b. Your gender?

- ◯ Female
- ⊗ Male
- ◯ Other

c. Are you Hispanic or Latino?

- ◯ Yes
- ⊗ No

d. What is your race? (Select one or more responses)

- ◯ American Indian or Alaska Native
- ◯ Asian (Please specify):
- ◯ Black or African American
- ◯ Native Hawaiian or Other Pacific Islander
- ⊗ White
- ◯ Other (Please specify):

e. Were you born in the United States?

- ⊗ Yes
- ◯ No

g. Your residency year?

- ◯ PGY1
- ⊗ PGY2
- ◯ PGY3
- ◯ PGY4
- ◯ PGY5

h. How would you classify your political ideology?

- ⊗ Conservative
- ◯ Somewhat conservative
- ◯ Moderate
- ◯ Somewhat liberal
- ◯ Liberal
- ◯ Other (Please specify):

i. Estimated level of educational debt?

- ◯ None
- ◯ Less than $50,000
- ◯ $50,000 - $100,000
- ⊗ $100,000 - $200,000
- ◯ $200,000 or more

j. Do you plan to subspecialize?

- ⊗ Yes
- ◯ No

k. Languages spoken?

- ☒ English
- ☐ Spanish
- ☐ French
- ☐ Hmong
- ☐ Somali
- ☐ Japanese
- ☐ Chinese
- ☐ Russian
- ☐ Ethiopian
- ☐ Other ___________

l. Are you in the Global Health Pathway?

- ◯ Yes
- ⊗ No

m. Did you earn your degree in the US?

- ⊗ Yes
- ◯ No

n. What residency program are you in?

- ◯ Internal Medicine
- ◯ Med-Peds
- ◯ Pediatrics
- ◯ Family Practice
- ◯ Neurology
- ◯ Psychiatry
- ◯ ObGyn
- ◯ Neurosurgery
- ⊗ General Surgery
- ◯ Orthopedic Surgery
- ◯ Urology
- ◯ Surgical sub-specialty (please specify in text box below)
- ◯ Non-clinical specialty (radiology, pathology; please specify in text box below)

Medical Trainees' attitudes, knowledge, and experience with immigrant and refugee health

Response was added on 12/13/2013 9:54pm.

SECTION A: Personal experience with immigrant and refugee health care.

A. Please indicate your level of agreement with the following statements regarding your personal experience with immigrant and refugee health care by checking the box that best represents your experience.

a. During my inpatient rotations, I take care of the following percentage of immigrant and refugee patients:

- ◯ None
- ⊗ 0 -5%
- ◯ 5-10%
- ◯ 10-25%
- ◯ > 25%

b. During my outpatient rotations, I take care of the following percentage of immigrant and refugee patients:

- ◯ None
- ◯ 0-10%
- ⊗ 10 -25%
- ◯ 25-50%
- ◯ 50-75%
- ◯ >75%

c. I would like to take care of more immigrant and refugee patients.

- ◯ Strongly disagree
- ◯ Disagree
- ◯ No opinion
- ⊗ Agree
- ◯ Strongly agree

d. I plan to take care of immigrants and refugees when I finish residency.

- ◯ Strongly disagree
- ◯ Disagree
- ◯ No opinion
- ⊗ Agree
- ◯ Strongly agree

e. I plan to do short term (< 6 months) international work when I finish residency.

- ◯ Strongly disagree
- ◯ Disagree
- ◯ No opinion
- ⊗ Agree
- ◯ Strongly agree

f. I plan to do long term (>6 months) international work when I finish residency.

- ◯ Strongly disagree
- ◯ Disagree
- ⊗ No opinion
- ◯ Agree
- ◯ Strongly agree

g. I plan to work in health disparities in the following way after residency:

Rural undeserved and in Latin America

SECTION B: MEDICAL EDUCATION

A. Please indicate your level of agreement with the following statements regarding your medical education and knowledge about immigrants and refugees by checking the box that best represents your opinion.

a. I have received specialized training in immigrant and refugee health, tropical medicine, or cross-cultural health.

- ◯ Strongly disagree
- ◯ Disagree
- ◯ No opinion
- ◯ Agree
- ⊗ Strongly agree

b. If you have received specialized training in immigrant and refugee health, tropical medicine, or cross-cultural health, please indicate all the contexts in which you received this training:

- ☒ As an undergraduate.
- ☒ As a medical student.
- ☐ As part of my residency.
- ☐ A special program.
- ☐ As part of my fellowship.
- ☐ As part of a degree program (e.g. MPH)
- ☐ Other:

c. I feel comfortable with my fund of knowledge regarding immigrant and refugee health.

- ◯ Strongly disagree
- ◯ Disagree
- ◯ No opinion
- ⊗ Agree
- ◯ Strongly agree

d. I would like to have further training in immigrant and refugee health.

- ◯ Strongly disagree
- ◯ Disagree
- ◯ No opinion
- ◯ Agree
- ⊗ Strongly agree

e. If you agree with the above, please indicate all the contexts in which you would like to receive this training:

- ☒ As part of my residency.
- ☒ A special program.
- ☐ As part of my fellowship.

SECTION C: Attitudes towards immigrant health

A. Please indicate your level of agreement with the following statements regarding immigrant and refugee health by checking the box that best represents your opinion.

a. I enjoy taking care of immigrants and refugees.

- ◯ Never
- ◯ Rarely
- ◯ Sometimes
- ⊗ Usually
- ◯ Always

b. Please indicate the reasons that you enjoy taking care of immigrants and refugees (may choose more than one).

- ☒ Tropical and other conditions not frequently diagnosed in US-born patients
- ☒ Learning about other cultures
- ☐ They don't complain as much
- ☒ Being able to hear their stories
- ☐ Their care is more complicated
- ☐ Their care is less complicated
- ☐ They are very appreciative of your help.
- ☐ They are extremely vulnerable
- ☐ Other:

c. Taking care of immigrants and refugees is more challenging than taking care of US born patients.

- ◯ Never
- ◯ Rarely
- ⊗ Sometimes
- ◯ Usually
- ◯ Always

d. Please mark all the challenges that you face as a provider when providing care to immigrants and refugees (may choose more than one):

- ☒ Language barriers
- ☒ Insurance barriers
- ☒ Cultural barriers
- ☐ Finding a professional interpreter
- ☐ Knowing how to work with a professional interpreter
- ☒ Time constraints
- ☐ My own knowledge related to tropical and travel medicine
- ☒ Transportation problems for the patient
- ☒ Patients not understanding treatment plan
- ☒ Patients not following treatment plan
- ☐ My lack of knowledge regarding the patient's culture
- ☐ Bias or stereotyping
- ☐ Other:

e. Please mark all of the challenges faced by immigrant and refugee populations when receiving healthcare that you have perceived or witnessed (may choose more than one):

- ☒ Language barriers
- ☒ Insurance barriers
- ☒ Cultural barriers
- ☒ Finding a professional interpreter
- ☒ Knowing how to work with a professional interpreter
- ☒ Time constraints
- ☐ Insufficiently trained health care providers
- ☒ Transportation problems for the patient
- ☒ Food insufficiency
- ☒ Need for child care
- ☒ Patients not understanding treatment plan
- ☒ Patients not following treatment plan
- ☒ My lack of knowledge regarding the patient's culture
- ☐ Bias or stereotyping
- ☒ Trust issues
- ☐ Other...

f. Rank how well immigrants and refugees understand the healthcare that you are trying to provide.

- ◯ Significantly less than a US born individual
- ⊗ Less than a US born individual
- ◯ Equivalent to a US born individual
- ◯ More than a US born patient
- ◯ Significantly more than a US born individual

g. Immigrants and refugees adhere to treatment plans and follow my recommendations.

- ◯ Never
- ◯ Rarely
- ⊗ Sometimes
- ◯ Usually
- ◯ Always

h. Immigrants and refugees should receive the same care and insurance coverage as US born patients.

- ◯ Never
- ◯ Rarely
- ◯ Sometimes
- ◯ Usually
- ⊗ Always

i. Immigrants and refugees who are undocumented should receive the same care and insurance coverage as US born patients.

- ◯ Never
- ◯ Rarely
- ◯ Sometimes
- ◯ Usually
- ⊗ Always

j. Every physician is professionally obligated to care for immigrants and refugees if they present to your clinic or hospital.

- ◯ Strongly disagree
- ◯ Disagree
- ◯ No opinion
- ◯ Agree
- ⊗ Strongly agree

k. Is healthcare a human right?

⊗ Yes
◯ No

B. If you wish, please tell us about what you enjoy or do not enjoy about immigrant and refugee health care and the greatest challenges you face in caring for this population.

____

SECTION D: DEMOGRAPHIC INFORMATION
Please answer the following questions by checking the box in front of the response choice that best describes you.

a. Your age?

◯ 20 to 24
⊗ 25 to 29
◯ 30 to 34
◯ 35 to 39
◯ 40 or older

b. Your gender?

⊗ Female
◯ Male
◯ Other

c.Are you Hispanic or Latino?

◯ Yes
⊗ No

d. What is your race? (Select one or more responses)

◯ American Indian or Alaska Native
◯ Asian (Please specify):
◯ Black or African American
◯ Native Hawaiian or Other Pacific Islander
⊗ White
◯ Other (Please specify):

e.Were you born in the United States?

⊗ Yes
◯ No

g. Your residency year?

◯ PGY1
⊗ PGY2
◯ PGY3
◯ PGY4
◯ PGY5

h. How would you classify your political ideology?

⊗ Conservative
◯ Somewhat conservative
◯ Moderate
◯ Somewhat liberal
◯ Liberal
◯ Other (Please specify):

i. Estimated level of educational debt?

◯ None
◯ Less than $50,000
◯ $50,000 - $100,000
◯ $100,000 - $200,000
⊗ $200,000 or more

j.Do you plan to subspecialize?

◯ Yes
⊗ No

k. Languages spoken?

- ☒ English
- ☒ Spanish
- ☐ French
- ☐ Hmong
- ☐ Somali
- ☐ Japanese
- ☐ Chinese
- ☐ Russian
- ☐ Ethiopian
- ☐ Other ___________

l. Are you in the Global Health Pathway?

- ⊗ Yes
- ◯ No

m. Did you earn your degree in the US?

- ⊗ Yes
- ◯ No

n. What residency program are you in?

- ◯ Internal Medicine
- ◯ Med-Peds
- ◯ Pediatrics
- ⊗ Family Practice
- ◯ Neurology
- ◯ Psychiatry
- ◯ ObGyn
- ◯ Neurosurgery
- ◯ General Surgery
- ◯ Orthopedic Surgery
- ◯ Urology
- ◯ Surgical sub-specialty (please specify in text box below)
- ◯ Non-clinical specialty (radiology, pathology; please specify in text box below)

Medical Trainees' attitudes, knowledge, and experience with immigrant and refugee health

Response was added on 12/14/2013 3:21am.

SECTION A: Personal experience with immigrant and refugee health care.

A. Please indicate your level of agreement with the following statements regarding your personal experience with immigrant and refugee health care by checking the box that best represents your experience.

a. During my inpatient rotations, I take care of the following percentage of immigrant and refugee patients:

- ○ None
- ⊗ 0 -5%
- ○ 5-10%
- ○ 10-25%
- ○ > 25%

b. During my outpatient rotations, I take care of the following percentage of immigrant and refugee patients:

- ○ None
- ⊗ 0-10%
- ○ 10 -25%
- ○ 25-50%
- ○ 50-75%
- ○ >75%

c. I would like to take care of more immigrant and refugee patients.

- ○ Strongly disagree
- ○ Disagree
- ○ No opinion
- ⊗ Agree
- ○ Strongly agree

d. I plan to take care of immigrants and refugees when I finish residency.

- ○ Strongly disagree
- ○ Disagree
- ○ No opinion
- ⊗ Agree
- ○ Strongly agree

e. I plan to do short term (< 6 months) international work when I finish residency.

- ○ Strongly disagree
- ○ Disagree
- ○ No opinion
- ⊗ Agree
- ○ Strongly agree

f. I plan to do long term (>6 months) international work when I finish residency.

- ○ Strongly disagree
- ○ Disagree
- ⊗ No opinion
- ○ Agree
- ○ Strongly agree

g. I plan to work in health disparities in the following way after residency:

I like doing some free work with underserved populations

SECTION B: MEDICAL EDUCATION

A. Please indicate your level of agreement with the following statements regarding your medical education and knowledge about immigrants and refugees by checking the box that best represents your opinion.

a. I have received specialized training in immigrant and refugee health, tropical medicine, or cross-cultural health.

◯ Strongly disagree
◯ Disagree
◯ No opinion
◯ Agree
⊗ Strongly agree

b. If you have received specialized training in immigrant and refugee health, tropical medicine, or cross-cultural health, please indicate all the contexts in which you received this training:

☐ As an undergraduate.
☐ As a medical student.
☒ As part of my residency.
☐ A special program.
☐ As part of my fellowship.
☐ As part of a degree program (e.g. MPH)
☐ Other:

c. I feel comfortable with my fund of knowledge regarding immigrant and refugee health.

◯ Strongly disagree
◯ Disagree
◯ No opinion
⊗ Agree
◯ Strongly agree

d. I would like to have further training in immigrant and refugee health.

◯ Strongly disagree
◯ Disagree
◯ No opinion
◯ Agree
⊗ Strongly agree

e. If you agree with the above, please indicate all the contexts in which you would like to receive this training:
□□

☒ As part of my residency.
☐ A special program.
☒ As part of my fellowship.

SECTION C: Attitudes towards immigrant health

A.□Please indicate your level of agreement with the following statements regarding immigrant and refugee health by checking the box that best represents your opinion.

a. I enjoy taking care of immigrants and refugees.

◯ Never
◯ Rarely
◯ Sometimes
⊗ Usually
◯ Always

b. Please indicate the reasons that you enjoy taking care of immigrants and refugees (may choose more than one).

☒ Tropical and other conditions not frequently diagnosed in US-born patients
☒ Learning about other cultures
☐ They don't complain as much
☐ Being able to hear their stories
☐ Their care is more complicated
☐ Their care is less complicated
☐ They are very appreciative of your help.
☐ They are extremely vulnerable
☐ Other:

c. Taking care of immigrants and refugees is more challenging than taking care of US born patients.

◯ Never
◯ Rarely
◯ Sometimes
⊗ Usually
◯ Always

d. Please mark all the challenges that you face as a provider when providing care to immigrants and refugees (may choose more than one):

- ☒ Language barriers
- ☐ Insurance barriers
- ☒ Cultural barriers
- ☐ Finding a professional interpreter
- ☐ Knowing how to work with a professional interpreter
- ☒ Time constraints
- ☐ My own knowledge related to tropical and travel medicine
- ☐ Transportation problems for the patient
- ☐ Patients not understanding treatment plan
- ☐ Patients not following treatment plan
- ☐ My lack of knowledge regarding the patient's culture
- ☐ Bias or stereotyping
- ☐ Other:

e. Please mark all of the challenges faced by immigrant and refugee populations when receiving healthcare that you have perceived or witnessed (may choose more than one):

- ☒ Language barriers
- ☒ Insurance barriers
- ☒ Cultural barriers
- ☒ Finding a professional interpreter
- ☐ Knowing how to work with a professional interpreter
- ☒ Time constraints
- ☒ Insufficiently trained health care providers
- ☒ Transportation problems for the patient
- ☐ Food insufficiency
- ☐ Need for child care
- ☒ Patients not understanding treatment plan
- ☒ Patients not following treatment plan
- ☒ My lack of knowledge regarding the patient's culture
- ☒ Bias or stereotyping
- ☒ Trust issues
- ☐ Other...

f. Rank how well immigrants and refugees understand the healthcare that you are trying to provide.

- ○ Significantly less than a US born individual
- ⊗ Less than a US born individual
- ○ Equivalent to a US born individual
- ○ More than a US born patient
- ○ Significantly more than a US born individual

g. Immigrants and refugees adhere to treatment plans and follow my recommendations.

- ○ Never
- ○ Rarely
- ○ Sometimes
- ⊗ Usually
- ○ Always

h. Immigrants and refugees should receive the same care and insurance coverage as US born patients.

- ○ Never
- ○ Rarely
- ○ Sometimes
- ○ Usually
- ⊗ Always

i. Immigrants and refugees who are undocumented should receive the same care and insurance coverage as US born patients.

- ○ Never
- ○ Rarely
- ○ Sometimes
- ○ Usually
- ⊗ Always

j. Every physician is professionally obligated to care for immigrants and refugees if they present to your clinic or hospital.

- ○ Strongly disagree
- ○ Disagree
- ○ No opinion
- ○ Agree
- ⊗ Strongly agree

k. Is healthcare a human right?

⊗ Yes
◯ No

B. If you wish, please tell us about what you enjoy or do not enjoy about immigrant and refugee health care and the greatest challenges you face in caring for this population.

____

SECTION D: DEMOGRAPHIC INFORMATION
Please answer the following questions by checking the box in front of the response choice that best describes you.

a. Your age?

◯ 20 to 24
◯ 25 to 29
⊗ 30 to 34
◯ 35 to 39
◯ 40 or older

b. Your gender?

◯ Female
⊗ Male
◯ Other

c.Are you Hispanic or Latino?

◯ Yes
⊗ No

d. What is your race? (Select one or more responses)

◯ American Indian or Alaska Native
◯ Asian (Please specify):
◯ Black or African American
◯ Native Hawaiian or Other Pacific Islander
⊗ White
◯ Other (Please specify):

e.Were you born in the United States?

⊗ Yes
◯ No

g. Your residency year?

◯ PGY1
◯ PGY2
◯ PGY3
◯ PGY4
⊗ PGY5

h. How would you classify your political ideology?

◯ Conservative
◯ Somewhat conservative
◯ Moderate
◯ Somewhat liberal
⊗ Liberal
◯ Other (Please specify):

i. Estimated level of educational debt?

◯ None
◯ Less than $50,000
◯ $50,000 - $100,000
◯ $100,000 - $200,000
⊗ $200,000 or more

j.Do you plan to subspecialize?

⊗ Yes
◯ No

k. Languages spoken?

- [x] English
- [ ] Spanish
- [ ] French
- [ ] Hmong
- [ ] Somali
- [ ] Japanese
- [ ] Chinese
- [ ] Russian
- [ ] Ethiopian
- [ ] Other ___________

l. Are you in the Global Health Pathway?

- (X) Yes
- ( ) No

m. Did you earn your degree in the US?

- (X) Yes
- ( ) No

n. What residency program are you in?

- (X) Internal Medicine
- ( ) Med-Peds
- ( ) Pediatrics
- ( ) Family Practice
- ( ) Neurology
- ( ) Psychiatry
- ( ) ObGyn
- ( ) Neurosurgery
- ( ) General Surgery
- ( ) Orthopedic Surgery
- ( ) Urology
- ( ) Surgical sub-specialty (please specify in text box below)
- ( ) Non-clinical specialty (radiology, pathology; please specify in text box below)

Medical Trainees' attitudes, knowledge, and experience with immigrant and refugee health

Response was added on 12/14/2013 7:23am.

SECTION A: Personal experience with immigrant and refugee health care.

A. Please indicate your level of agreement with the following statements regarding your personal experience with immigrant and refugee health care by checking the box that best represents your experience.

a. During my inpatient rotations, I take care of the following percentage of immigrant and refugee patients:
- ◯ None
- ⊗ 0 -5%
- ◯ 5-10%
- ◯ 10-25%
- ◯ > 25%

b. During my outpatient rotations, I take care of the following percentage of immigrant and refugee patients:
- ◯ None
- ⊗ 0-10%
- ◯ 10 -25%
- ◯ 25-50%
- ◯ 50-75%
- ◯ >75%

c. I would like to take care of more immigrant and refugee patients.
- ◯ Strongly disagree
- ◯ Disagree
- ◯ No opinion
- ◯ Agree
- ⊗ Strongly agree

d. I plan to take care of immigrants and refugees when I finish residency.
- ◯ Strongly disagree
- ◯ Disagree
- ◯ No opinion
- ◯ Agree
- ⊗ Strongly agree

e. I plan to do short term (< 6 months) international work when I finish residency.
- ◯ Strongly disagree
- ◯ Disagree
- ◯ No opinion
- ◯ Agree
- ⊗ Strongly agree

f. I plan to do long term (>6 months) international work when I finish residency.
- ◯ Strongly disagree
- ⊗ Disagree
- ◯ No opinion
- ◯ Agree
- ◯ Strongly agree

g. I plan to work in health disparities in the following way after residency:

Free clinic, reduced rates

SECTION B: MEDICAL EDUCATION

A. Please indicate your level of agreement with the following statements regarding your medical education and knowledge about immigrants and refugees by checking the box that best represents your opinion.

a. I have received specialized training in immigrant and refugee health, tropical medicine, or cross-cultural health.

- ◯ Strongly disagree
- ◯ Disagree
- ◯ No opinion
- ⊗ Agree
- ◯ Strongly agree

b. If you have received specialized training in immigrant and refugee health, tropical medicine, or cross-cultural health, please indicate all the contexts in which you received this training:

- ☐ As an undergraduate.
- ☒ As a medical student.
- ☐ As part of my residency.
- ☐ A special program.
- ☐ As part of my fellowship.
- ☐ As part of a degree program (e.g. MPH)
- ☐ Other:

c. I feel comfortable with my fund of knowledge regarding immigrant and refugee health.

- ⊗ Strongly disagree
- ◯ Disagree
- ◯ No opinion
- ◯ Agree
- ◯ Strongly agree

d. I would like to have further training in immigrant and refugee health.

- ◯ Strongly disagree
- ◯ Disagree
- ◯ No opinion
- ◯ Agree
- ⊗ Strongly agree

e. If you agree with the above, please indicate all the contexts in which you would like to receive this training:

- ☒ As part of my residency.
- ☒ A special program.
- ☒ As part of my fellowship.

SECTION C: Attitudes towards immigrant health

A. Please indicate your level of agreement with the following statements regarding immigrant and refugee health by checking the box that best represents your opinion.

a. I enjoy taking care of immigrants and refugees.

- ◯ Never
- ◯ Rarely
- ◯ Sometimes
- ◯ Usually
- ⊗ Always

b. Please indicate the reasons that you enjoy taking care of immigrants and refugees (may choose more than one).

- ☒ Tropical and other conditions not frequently diagnosed in US-born patients
- ☒ Learning about other cultures
- ☐ They don't complain as much
- ☒ Being able to hear their stories
- ☒ Their care is more complicated
- ☐ Their care is less complicated
- ☐ They are very appreciative of your help.
- ☒ They are extremely vulnerable
- ☐ Other:

c. Taking care of immigrants and refugees is more challenging than taking care of US born patients.

- ◯ Never
- ◯ Rarely
- ◯ Sometimes
- ⊗ Usually
- ◯ Always

d. Please mark all the challenges that you face as a provider when providing care to immigrants and refugees (may choose more than one):

- ☒ Language barriers
- ☐ Insurance barriers
- ☒ Cultural barriers
- ☒ Finding a professional interpreter
- ☐ Knowing how to work with a professional interpreter
- ☒ Time constraints
- ☒ My own knowledge related to tropical and travel medicine
- ☐ Transportation problems for the patient
- ☒ Patients not understanding treatment plan
- ☐ Patients not following treatment plan
- ☒ My lack of knowledge regarding the patient's culture
- ☐ Bias or stereotyping
- ☐ Other:

e. Please mark all of the challenges faced by immigrant and refugee populations when receiving healthcare that you have perceived or witnessed (may choose more than one):

- ☒ Language barriers
- ☒ Insurance barriers
- ☒ Cultural barriers
- ☒ Finding a professional interpreter
- ☐ Knowing how to work with a professional interpreter
- ☐ Time constraints
- ☒ Insufficiently trained health care providers
- ☒ Transportation problems for the patient
- ☒ Food insufficiency
- ☒ Need for child care
- ☒ Patients not understanding treatment plan
- ☒ Patients not following treatment plan
- ☒ My lack of knowledge regarding the patient's culture
- ☐ Bias or stereotyping
- ☒ Trust issues
- ☐ Other...

f. Rank how well immigrants and refugees understand the healthcare that you are trying to provide.

- ○ Significantly less than a US born individual
- ⊗ Less than a US born individual
- ○ Equivalent to a US born individual
- ○ More than a US born patient
- ○ Significantly more than a US born individual

g. Immigrants and refugees adhere to treatment plans and follow my recommendations.

- ○ Never
- ○ Rarely
- ○ Sometimes
- ⊗ Usually
- ○ Always

h. Immigrants and refugees should receive the same care and insurance coverage as US born patients.

- ○ Never
- ○ Rarely
- ○ Sometimes
- ○ Usually
- ⊗ Always

i. Immigrants and refugees who are undocumented should receive the same care and insurance coverage as US born patients.

- ○ Never
- ○ Rarely
- ○ Sometimes
- ○ Usually
- ⊗ Always

j. Every physician is professionally obligated to care for immigrants and refugees if they present to your clinic or hospital.

- ○ Strongly disagree
- ○ Disagree
- ○ No opinion
- ○ Agree
- ⊗ Strongly agree

k. Is healthcare a human right?
- ⊗ Yes
- ◯ No

B. If you wish, please tell us about what you enjoy or do not enjoy about immigrant and refugee health care and the greatest challenges you face in caring for this population.

We joined medicine to help people. These people really need our help.

SECTION D: DEMOGRAPHIC INFORMATION
Please answer the following questions by checking the box in front of the response choice that best describes you.

a. Your age?
- ◯ 20 to 24
- ⊗ 25 to 29
- ◯ 30 to 34
- ◯ 35 to 39
- ◯ 40 or older

b. Your gender?
- ⊗ Female
- ◯ Male
- ◯ Other

c. Are you Hispanic or Latino?
- ◯ Yes
- ⊗ No

d. What is your race? (Select one or more responses)
- ◯ American Indian or Alaska Native
- ◯ Asian (Please specify):
- ◯ Black or African American
- ◯ Native Hawaiian or Other Pacific Islander
- ⊗ White
- ◯ Other (Please specify):

e. Were you born in the United States?
- ⊗ Yes
- ◯ No

g. Your residency year?
- ⊗ PGY1
- ◯ PGY2
- ◯ PGY3
- ◯ PGY4
- ◯ PGY5

h. How would you classify your political ideology?
- ◯ Conservative
- ◯ Somewhat conservative
- ◯ Moderate
- ◯ Somewhat liberal
- ⊗ Liberal
- ◯ Other (Please specify):

i. Estimated level of educational debt?
- ◯ None
- ◯ Less than $50,000
- ◯ $50,000 - $100,000
- ⊗ $100,000 - $200,000
- ◯ $200,000 or more

j. Do you plan to subspecialize?
- ⊗ Yes
- ◯ No

k. Languages spoken?

- ☒ English
- ☐ Spanish
- ☐ French
- ☐ Hmong
- ☐ Somali
- ☐ Japanese
- ☐ Chinese
- ☐ Russian
- ☐ Ethiopian
- ☒ Other ___________

German

l. Are you in the Global Health Pathway?

- ◯ Yes
- ⊗ No

m. Did you earn your degree in the US?

- ⊗ Yes
- ◯ No

n. What residency program are you in?

- ◯ Internal Medicine
- ◯ Med-Peds
- ◯ Pediatrics
- ◯ Family Practice
- ◯ Neurology
- ⊗ Psychiatry
- ◯ ObGyn
- ◯ Neurosurgery
- ◯ General Surgery
- ◯ Orthopedic Surgery
- ◯ Urology
- ◯ Surgical sub-specialty (please specify in text box below)
- ◯ Non-clinical specialty (radiology, pathology; please specify in text box below)

Medical Trainees' attitudes, knowledge, and experience with immigrant and refugee health

Response was added on 12/14/2013 9:25am.

SECTION A: Personal experience with immigrant and refugee health care.

A. Please indicate your level of agreement with the following statements regarding your personal experience with immigrant and refugee health care by checking the box that best represents your experience.

a. During my inpatient rotations, I take care of the following percentage of immigrant and refugee patients:
- ◯ None
- ◯ 0 -5%
- ⊗ 5-10%
- ◯ 10-25%
- ◯ > 25%

b. During my outpatient rotations, I take care of the following percentage of immigrant and refugee patients:
- ◯ None
- ⊗ 0-10%
- ◯ 10 -25%
- ◯ 25-50%
- ◯ 50-75%
- ◯ >75%

c. I would like to take care of more immigrant and refugee patients.
- ◯ Strongly disagree
- ◯ Disagree
- ◯ No opinion
- ⊗ Agree
- ◯ Strongly agree

d. I plan to take care of immigrants and refugees when I finish residency.
- ◯ Strongly disagree
- ◯ Disagree
- ⊗ No opinion
- ◯ Agree
- ◯ Strongly agree

e. I plan to do short term (< 6 months) international work when I finish residency.
- ◯ Strongly disagree
- ◯ Disagree
- ◯ No opinion
- ⊗ Agree
- ◯ Strongly agree

f. I plan to do long term (>6 months) international work when I finish residency.
- ◯ Strongly disagree
- ◯ Disagree
- ◯ No opinion
- ◯ Agree
- ⊗ Strongly agree

g. I plan to work in health disparities in the following way after residency:

Hopefully working for international NGO

SECTION B: MEDICAL EDUCATION

A. Please indicate your level of agreement with the following statements regarding your medical education and knowledge about immigrants and refugees by checking the box that best represents your opinion.

a. I have received specialized training in immigrant and refugee health, tropical medicine, or cross-cultural health.

◯ Strongly disagree
◯ Disagree
◯ No opinion
◯ Agree
⊗ Strongly agree

b. If you have received specialized training in immigrant and refugee health, tropical medicine, or cross-cultural health, please indicate all the contexts in which you received this training:

☐ As an undergraduate.
☐ As a medical student.
☐ As part of my residency.
☐ A special program.
☐ As part of my fellowship.
☒ As part of a degree program (e.g. MPH)
☐ Other:

c. I feel comfortable with my fund of knowledge regarding immigrant and refugee health.

◯ Strongly disagree
◯ Disagree
⊗ No opinion
◯ Agree
◯ Strongly agree

d. I would like to have further training in immigrant and refugee health.

◯ Strongly disagree
◯ Disagree
◯ No opinion
◯ Agree
⊗ Strongly agree

e. If you agree with the above, please indicate all the contexts in which you would like to receive this training:
□□

☒ As part of my residency.
☒ A special program.
☒ As part of my fellowship.

SECTION C: Attitudes towards immigrant health

A.□Please indicate your level of agreement with the following statements regarding immigrant and refugee health by checking the box that best represents your opinion.

a. I enjoy taking care of immigrants and refugees.

◯ Never
◯ Rarely
◯ Sometimes
⊗ Usually
◯ Always

b. Please indicate the reasons that you enjoy taking care of immigrants and refugees (may choose more than one).

☒ Tropical and other conditions not frequently diagnosed in US-born patients
☒ Learning about other cultures
☐ They don't complain as much
☒ Being able to hear their stories
☐ Their care is more complicated
☐ Their care is less complicated
☐ They are very appreciative of your help.
☐ They are extremely vulnerable
☐ Other:

c. Taking care of immigrants and refugees is more challenging than taking care of US born patients.

◯ Never
◯ Rarely
⊗ Sometimes
◯ Usually
◯ Always

d. Please mark all the challenges that you face as a provider when providing care to immigrants and refugees (may choose more than one):

- ☒ Language barriers
- ☐ Insurance barriers
- ☒ Cultural barriers
- ☒ Finding a professional interpreter
- ☐ Knowing how to work with a professional interpreter
- ☒ Time constraints
- ☒ My own knowledge related to tropical and travel medicine
- ☒ Transportation problems for the patient
- ☒ Patients not understanding treatment plan
- ☒ Patients not following treatment plan
- ☒ My lack of knowledge regarding the patient's culture
- ☒ Bias or stereotyping
- ☐ Other:

e. Please mark all of the challenges faced by immigrant and refugee populations when receiving healthcare that you have perceived or witnessed (may choose more than one):

- ☒ Language barriers
- ☒ Insurance barriers
- ☒ Cultural barriers
- ☒ Finding a professional interpreter
- ☒ Knowing how to work with a professional interpreter
- ☒ Time constraints
- ☒ Insufficiently trained health care providers
- ☒ Transportation problems for the patient
- ☐ Food insufficiency
- ☐ Need for child care
- ☒ Patients not understanding treatment plan
- ☒ Patients not following treatment plan
- ☒ My lack of knowledge regarding the patient's culture
- ☒ Bias or stereotyping
- ☒ Trust issues
- ☐ Other...

f. Rank how well immigrants and refugees understand the healthcare that you are trying to provide.

- ○ Significantly less than a US born individual
- ○ Less than a US born individual
- ⊗ Equivalent to a US born individual
- ○ More than a US born patient
- ○ Significantly more than a US born individual

g. Immigrants and refugees adhere to treatment plans and follow my recommendations.

- ○ Never
- ○ Rarely
- ○ Sometimes
- ○ Usually
- ○ Always

h. Immigrants and refugees should receive the same care and insurance coverage as US born patients.

- ○ Never
- ○ Rarely
- ○ Sometimes
- ○ Usually
- ⊗ Always

i. Immigrants and refugees who are undocumented should receive the same care and insurance coverage as US born patients.

- ○ Never
- ○ Rarely
- ○ Sometimes
- ○ Usually
- ⊗ Always

j. Every physician is professionally obligated to care for immigrants and refugees if they present to your clinic or hospital.

- ○ Strongly disagree
- ○ Disagree
- ○ No opinion
- ⊗ Agree
- ○ Strongly agree

k. Is healthcare a human right?

⊗ Yes
◯ No

B. If you wish, please tell us about what you enjoy or do not enjoy about immigrant and refugee health care and the greatest challenges you face in caring for this population.

____

SECTION D: DEMOGRAPHIC INFORMATION
Please answer the following questions by checking the box in front of the response choice that best describes you.

a. Your age?

◯ 20 to 24
◯ 25 to 29
⊗ 30 to 34
◯ 35 to 39
◯ 40 or older

b. Your gender?

⊗ Female
◯ Male
◯ Other

c.Are you Hispanic or Latino?

◯ Yes
⊗ No

d. What is your race? (Select one or more responses)

◯ American Indian or Alaska Native
◯ Asian (Please specify):
◯ Black or African American
◯ Native Hawaiian or Other Pacific Islander
⊗ White
◯ Other (Please specify):

e.Were you born in the United States?

⊗ Yes
◯ No

g. Your residency year?

⊗ PGY1
◯ PGY2
◯ PGY3
◯ PGY4
◯ PGY5

h. How would you classify your political ideology?

◯ Conservative
◯ Somewhat conservative
◯ Moderate
⊗ Somewhat liberal
◯ Liberal
◯ Other (Please specify):

i. Estimated level of educational debt?

◯ None
⊗ Less than $50,000
◯ $50,000 - $100,000
◯ $100,000 - $200,000
◯ $200,000 or more

j.Do you plan to subspecialize?

⊗ Yes
◯ No

k. Languages spoken?

- ☒ English
- ☐ Spanish
- ☐ French
- ☐ Hmong
- ☐ Somali
- ☐ Japanese
- ☐ Chinese
- ☐ Russian
- ☐ Ethiopian
- ☐ Other ___________

l. Are you in the Global Health Pathway?

- ⊗ Yes
- ◯ No

m. Did you earn your degree in the US?

- ⊗ Yes
- ◯ No

n. What residency program are you in?

- ⊗ Internal Medicine
- ◯ Med-Peds
- ◯ Pediatrics
- ◯ Family Practice
- ◯ Neurology
- ◯ Psychiatry
- ◯ ObGyn
- ◯ Neurosurgery
- ◯ General Surgery
- ◯ Orthopedic Surgery
- ◯ Urology
- ◯ Surgical sub-specialty (please specify in text box below)
- ◯ Non-clinical specialty (radiology, pathology; please specify in text box below)

Medical Trainees' attitudes, knowledge, and experience with immigrant and refugee health

Response was added on 12/14/2013 11:03am.

SECTION A: Personal experience with immigrant and refugee health care.

A. Please indicate your level of agreement with the following statements regarding your personal experience with immigrant and refugee health care by checking the box that best represents your experience.

a. During my inpatient rotations, I take care of the following percentage of immigrant and refugee patients:
- ◯ None
- ◯ 0 -5%
- ⊗ 5-10%
- ◯ 10-25%
- ◯ > 25%

b. During my outpatient rotations, I take care of the following percentage of immigrant and refugee patients:
- ◯ None
- ⊗ 0-10%
- ◯ 10 -25%
- ◯ 25-50%
- ◯ 50-75%
- ◯ >75%

c. I would like to take care of more immigrant and refugee patients.
- ◯ Strongly disagree
- ◯ Disagree
- ◯ No opinion
- ⊗ Agree
- ◯ Strongly agree

d. I plan to take care of immigrants and refugees when I finish residency.
- ◯ Strongly disagree
- ◯ Disagree
- ⊗ No opinion
- ◯ Agree
- ◯ Strongly agree

e. I plan to do short term (< 6 months) international work when I finish residency.
- ◯ Strongly disagree
- ◯ Disagree
- ⊗ No opinion
- ◯ Agree
- ◯ Strongly agree

f. I plan to do long term (>6 months) international work when I finish residency.
- ◯ Strongly disagree
- ⊗ Disagree
- ◯ No opinion
- ◯ Agree
- ◯ Strongly agree

g. I plan to work in health disparities in the following way after residency:

Focus on underserved communities, not necessarily immigrants

SECTION B: MEDICAL EDUCATION

A. Please indicate your level of agreement with the following statements regarding your medical education and knowledge about immigrants and refugees by checking the box that best represents your opinion.

a. I have received specialized training in immigrant and refugee health, tropical medicine, or cross-cultural health.

- ◯ Strongly disagree
- ◯ Disagree
- ◯ No opinion
- ⊗ Agree
- ◯ Strongly agree

b. If you have received specialized training in immigrant and refugee health, tropical medicine, or cross-cultural health, please indicate all the contexts in which you received this training:

- ☐ As an undergraduate.
- ☐ As a medical student.
- ☐ As part of my residency.
- ☐ A special program.
- ☐ As part of my fellowship.
- ☐ As part of a degree program (e.g. MPH)
- ☐ Other:

c. I feel comfortable with my fund of knowledge regarding immigrant and refugee health.

- ◯ Strongly disagree
- ⊗ Disagree
- ◯ No opinion
- ◯ Agree
- ◯ Strongly agree

d. I would like to have further training in immigrant and refugee health.

- ◯ Strongly disagree
- ◯ Disagree
- ◯ No opinion
- ⊗ Agree
- ◯ Strongly agree

e. If you agree with the above, please indicate all the contexts in which you would like to receive this training:
□□

- ☒ As part of my residency.
- ☐ A special program.
- ☐ As part of my fellowship.

SECTION C: Attitudes towards immigrant health

A.□Please indicate your level of agreement with the following statements regarding immigrant and refugee health by checking the box that best represents your opinion.

a. I enjoy taking care of immigrants and refugees.

- ◯ Never
- ◯ Rarely
- ◯ Sometimes
- ⊗ Usually
- ◯ Always

b. Please indicate the reasons that you enjoy taking care of immigrants and refugees (may choose more than one).

- ☒ Tropical and other conditions not frequently diagnosed in US-born patients
- ☐ Learning about other cultures
- ☐ They don't complain as much
- ☐ Being able to hear their stories
- ☐ Their care is more complicated
- ☐ Their care is less complicated
- ☐ They are very appreciative of your help.
- ☒ They are extremely vulnerable
- ☐ Other:

c. Taking care of immigrants and refugees is more challenging than taking care of US born patients.

- ◯ Never
- ◯ Rarely
- ◯ Sometimes
- ◯ Usually
- ⊗ Always

d. Please mark all the challenges that you face as a provider when providing care to immigrants and refugees (may choose more than one):

- [x] Language barriers
- [x] Insurance barriers
- [x] Cultural barriers
- [x] Finding a professional interpreter
- [ ] Knowing how to work with a professional interpreter
- [x] Time constraints
- [ ] My own knowledge related to tropical and travel medicine
- [ ] Transportation problems for the patient
- [x] Patients not understanding treatment plan
- [ ] Patients not following treatment plan
- [ ] My lack of knowledge regarding the patient's culture
- [ ] Bias or stereotyping
- [ ] Other:

e. Please mark all of the challenges faced by immigrant and refugee populations when receiving healthcare that you have perceived or witnessed (may choose more than one):

- [x] Language barriers
- [x] Insurance barriers
- [x] Cultural barriers
- [x] Finding a professional interpreter
- [x] Knowing how to work with a professional interpreter
- [x] Time constraints
- [x] Insufficiently trained health care providers
- [x] Transportation problems for the patient
- [ ] Food insufficiency
- [x] Need for child care
- [x] Patients not understanding treatment plan
- [x] Patients not following treatment plan
- [x] My lack of knowledge regarding the patient's culture
- [ ] Bias or stereotyping
- [x] Trust issues
- [ ] Other...

f. Rank how well immigrants and refugees understand the healthcare that you are trying to provide.

- ○ Significantly less than a US born individual
- ⊗ Less than a US born individual
- ○ Equivalent to a US born individual
- ○ More than a US born patient
- ○ Significantly more than a US born individual

g. Immigrants and refugees adhere to treatment plans and follow my recommendations.

- ○ Never
- ⊗ Rarely
- ○ Sometimes
- ○ Usually
- ○ Always

h. Immigrants and refugees should receive the same care and insurance coverage as US born patients.

- ○ Never
- ○ Rarely
- ○ Sometimes
- ⊗ Usually
- ○ Always

i. Immigrants and refugees who are undocumented should receive the same care and insurance coverage as US born patients.

- ○ Never
- ○ Rarely
- ⊗ Sometimes
- ○ Usually
- ○ Always

j. Every physician is professionally obligated to care for immigrants and refugees if they present to your clinic or hospital.

- ○ Strongly disagree
- ○ Disagree
- ○ No opinion
- ⊗ Agree
- ○ Strongly agree

k. Is healthcare a human right?
- ⊗ Yes
- ◯ No

B. If you wish, please tell us about what you enjoy or do not enjoy about immigrant and refugee health care and the greatest challenges you face in caring for this population.

____

SECTION D: DEMOGRAPHIC INFORMATION
Please answer the following questions by checking the box in front of the response choice that best describes you.

a. Your age?
- ◯ 20 to 24
- ◯ 25 to 29
- ⊗ 30 to 34
- ◯ 35 to 39
- ◯ 40 or older

b. Your gender?
- ◯ Female
- ⊗ Male
- ◯ Other

c.Are you Hispanic or Latino?
- ◯ Yes
- ⊗ No

d. What is your race? (Select one or more responses)
- ◯ American Indian or Alaska Native
- ◯ Asian (Please specify):
- ◯ Black or African American
- ◯ Native Hawaiian or Other Pacific Islander
- ⊗ White
- ◯ Other (Please specify):

e.Were you born in the United States?
- ⊗ Yes
- ◯ No

g. Your residency year?
- ◯ PGY1
- ⊗ PGY2
- ◯ PGY3
- ◯ PGY4
- ◯ PGY5

h. How would you classify your political ideology?
- ◯ Conservative
- ◯ Somewhat conservative
- ◯ Moderate
- ⊗ Somewhat liberal
- ◯ Liberal
- ◯ Other (Please specify):

i. Estimated level of educational debt?
- ◯ None
- ◯ Less than $50,000
- ◯ $50,000 - $100,000
- ◯ $100,000 - $200,000
- ⊗ $200,000 or more

j.Do you plan to subspecialize?
- ◯ Yes
- ⊗ No

k. Languages spoken?

☒ English
☐ Spanish
☐ French
☐ Hmong
☐ Somali
☐ Japanese
☐ Chinese
☐ Russian
☐ Ethiopian
☐ Other ___________

l. Are you in the Global Health Pathway?

◯ Yes
⊗ No

m. Did you earn your degree in the US?

⊗ Yes
◯ No

n. What residency program are you in?

⊗ Internal Medicine
◯ Med-Peds
◯ Pediatrics
◯ Family Practice
◯ Neurology
◯ Psychiatry
◯ ObGyn
◯ Neurosurgery
◯ General Surgery
◯ Orthopedic Surgery
◯ Urology
◯ Surgical sub-specialty (please specify in text box below)
◯ Non-clinical specialty (radiology, pathology; please specify in text box below)

Medical Trainees' attitudes, knowledge, and experience with immigrant and refugee health

Response was added on 12/14/2013 12:54pm.

SECTION A: Personal experience with immigrant and refugee health care.

A. Please indicate your level of agreement with the following statements regarding your personal experience with immigrant and refugee health care by checking the box that best represents your experience.

a. During my inpatient rotations, I take care of the following percentage of immigrant and refugee patients:
- ◯ None
- ◯ 0 -5%
- ◯ 5-10%
- ◯ 10-25%
- ⊗ > 25%

b. During my outpatient rotations, I take care of the following percentage of immigrant and refugee patients:
- ◯ None
- ◯ 0-10%
- ◯ 10 -25%
- ◯ 25-50%
- ⊗ 50-75%
- ◯ >75%

c. I would like to take care of more immigrant and refugee patients.
- ◯ Strongly disagree
- ◯ Disagree
- ◯ No opinion
- ◯ Agree
- ⊗ Strongly agree

d. I plan to take care of immigrants and refugees when I finish residency.
- ◯ Strongly disagree
- ◯ Disagree
- ◯ No opinion
- ◯ Agree
- ⊗ Strongly agree

e. I plan to do short term (< 6 months) international work when I finish residency.
- ◯ Strongly disagree
- ◯ Disagree
- ◯ No opinion
- ◯ Agree
- ⊗ Strongly agree

f. I plan to do long term (>6 months) international work when I finish residency.
- ◯ Strongly disagree
- ◯ Disagree
- ◯ No opinion
- ◯ Agree
- ⊗ Strongly agree

g. I plan to work in health disparities in the following way after residency:
Either in an underserved clinic or overseas

SECTION B: MEDICAL EDUCATION

A. Please indicate your level of agreement with the following statements regarding your medical education and knowledge about immigrants and refugees by checking the box that best represents your opinion.

a. I have received specialized training in immigrant and refugee health, tropical medicine, or cross-cultural health.

- ◯ Strongly disagree
- ◯ Disagree
- ◯ No opinion
- ◯ Agree
- ⊗ Strongly agree

b. If you have received specialized training in immigrant and refugee health, tropical medicine, or cross-cultural health, please indicate all the contexts in which you received this training:

- ☒ As an undergraduate.
- ☒ As a medical student.
- ☒ As part of my residency.
- ☒ A special program.
- ☐ As part of my fellowship.
- ☐ As part of a degree program (e.g. MPH)
- ☐ Other:

c. I feel comfortable with my fund of knowledge regarding immigrant and refugee health.

- ◯ Strongly disagree
- ◯ Disagree
- ◯ No opinion
- ⊗ Agree
- ◯ Strongly agree

d. I would like to have further training in immigrant and refugee health.

- ◯ Strongly disagree
- ◯ Disagree
- ◯ No opinion
- ◯ Agree
- ⊗ Strongly agree

e. If you agree with the above, please indicate all the contexts in which you would like to receive this training:

- ☒ As part of my residency.
- ☐ A special program.
- ☐ As part of my fellowship.

SECTION C: Attitudes towards immigrant health

A. Please indicate your level of agreement with the following statements regarding immigrant and refugee health by checking the box that best represents your opinion.

a. I enjoy taking care of immigrants and refugees.

- ◯ Never
- ◯ Rarely
- ◯ Sometimes
- ◯ Usually
- ⊗ Always

b. Please indicate the reasons that you enjoy taking care of immigrants and refugees (may choose more than one).

- ☒ Tropical and other conditions not frequently diagnosed in US-born patients
- ☒ Learning about other cultures
- ☒ They don't complain as much
- ☒ Being able to hear their stories
- ☐ Their care is more complicated
- ☐ Their care is less complicated
- ☒ They are very appreciative of your help.
- ☒ They are extremely vulnerable
- ☐ Other:

c. Taking care of immigrants and refugees is more challenging than taking care of US born patients.

- ◯ Never
- ◯ Rarely
- ◯ Sometimes
- ⊗ Usually
- ◯ Always

d. Please mark all the challenges that you face as a provider when providing care to immigrants and refugees (may choose more than one):

- ☒ Language barriers
- ☐ Insurance barriers
- ☒ Cultural barriers
- ☐ Finding a professional interpreter
- ☐ Knowing how to work with a professional interpreter
- ☒ Time constraints
- ☒ My own knowledge related to tropical and travel medicine
- ☒ Transportation problems for the patient
- ☒ Patients not understanding treatment plan
- ☐ Patients not following treatment plan
- ☒ My lack of knowledge regarding the patient's culture
- ☐ Bias or stereotyping
- ☐ Other:

e. Please mark all of the challenges faced by immigrant and refugee populations when receiving healthcare that you have perceived or witnessed (may choose more than one):

- ☒ Language barriers
- ☒ Insurance barriers
- ☒ Cultural barriers
- ☒ Finding a professional interpreter
- ☐ Knowing how to work with a professional interpreter
- ☐ Time constraints
- ☒ Insufficiently trained health care providers
- ☒ Transportation problems for the patient
- ☒ Food insufficiency
- ☒ Need for child care
- ☒ Patients not understanding treatment plan
- ☐ Patients not following treatment plan
- ☐ My lack of knowledge regarding the patient's culture
- ☐ Bias or stereotyping
- ☒ Trust issues
- ☐ Other...

f. Rank how well immigrants and refugees understand the healthcare that you are trying to provide.

- ⊗ Significantly less than a US born individual
- ○ Less than a US born individual
- ○ Equivalent to a US born individual
- ○ More than a US born patient
- ○ Significantly more than a US born individual

g. Immigrants and refugees adhere to treatment plans and follow my recommendations.

- ○ Never
- ○ Rarely
- ⊗ Sometimes
- ○ Usually
- ○ Always

h. Immigrants and refugees should receive the same care and insurance coverage as US born patients.

- ○ Never
- ○ Rarely
- ○ Sometimes
- ○ Usually
- ⊗ Always

i. Immigrants and refugees who are undocumented should receive the same care and insurance coverage as US born patients.

- ○ Never
- ○ Rarely
- ○ Sometimes
- ○ Usually
- ⊗ Always

j. Every physician is professionally obligated to care for immigrants and refugees if they present to your clinic or hospital.

- ○ Strongly disagree
- ○ Disagree
- ○ No opinion
- ○ Agree
- ⊗ Strongly agree

k. Is healthcare a human right?

⊗ Yes
◯ No

B. If you wish, please tell us about what you enjoy or do not enjoy about immigrant and refugee health care and the greatest challenges you face in caring for this population.

____

SECTION D: DEMOGRAPHIC INFORMATION
Please answer the following questions by checking the box in front of the response choice that best describes you.

a. Your age?

◯ 20 to 24
⊗ 25 to 29
◯ 30 to 34
◯ 35 to 39
◯ 40 or older

b. Your gender?

⊗ Female
◯ Male
◯ Other

c.Are you Hispanic or Latino?

◯ Yes
⊗ No

d. What is your race? (Select one or more responses)

◯ American Indian or Alaska Native
◯ Asian (Please specify):
◯ Black or African American
◯ Native Hawaiian or Other Pacific Islander
⊗ White
◯ Other (Please specify):

e.Were you born in the United States?

⊗ Yes
◯ No

g. Your residency year?

◯ PGY1
⊗ PGY2
◯ PGY3
◯ PGY4
◯ PGY5

h. How would you classify your political ideology?

◯ Conservative
◯ Somewhat conservative
◯ Moderate
⊗ Somewhat liberal
◯ Liberal
◯ Other (Please specify):

i. Estimated level of educational debt?

◯ None
◯ Less than $50,000
◯ $50,000 - $100,000
⊗ $100,000 - $200,000
◯ $200,000 or more

j.Do you plan to subspecialize?

◯ Yes
⊗ No

k. Languages spoken?
- ☒ English
- ☒ Spanish
- ☐ French
- ☐ Hmong
- ☐ Somali
- ☐ Japanese
- ☐ Chinese
- ☐ Russian
- ☐ Ethiopian
- ☐ Other ___________

l. Are you in the Global Health Pathway?
- ⊗ Yes
- ◯ No

m.▯ Did you earn your degree in the US?
- ⊗ Yes
- ◯ No

n. What residency program are you in?
- ◯ Internal Medicine
- ◯ Med-Peds
- ◯ Pediatrics
- ⊗ Family Practice
- ◯ Neurology
- ◯ Psychiatry
- ◯ ObGyn
- ◯ Neurosurgery
- ◯ General Surgery
- ◯ Orthopedic Surgery
- ◯ Urology
- ◯ Surgical sub-specialty (please specify in text box below)
- ◯ Non-clinical specialty (radiology, pathology; please specify in text box below)

Medical Trainees' attitudes, knowledge, and experience with immigrant and refugee health

Response was added on 12/14/2013 6:44pm.

SECTION A: Personal experience with immigrant and refugee health care.

A. Please indicate your level of agreement with the following statements regarding your personal experience with immigrant and refugee health care by checking the box that best represents your experience.

a. During my inpatient rotations, I take care of the following percentage of immigrant and refugee patients:

- ◯ None
- ⊗ 0 -5%
- ◯ 5-10%
- ◯ 10-25%
- ◯ > 25%

b. During my outpatient rotations, I take care of the following percentage of immigrant and refugee patients:

- ◯ None
- ⊗ 0-10%
- ◯ 10 -25%
- ◯ 25-50%
- ◯ 50-75%
- ◯ >75%

c. I would like to take care of more immigrant and refugee patients.

- ◯ Strongly disagree
- ◯ Disagree
- ◯ No opinion
- ⊗ Agree
- ◯ Strongly agree

d. I plan to take care of immigrants and refugees when I finish residency.

- ◯ Strongly disagree
- ◯ Disagree
- ◯ No opinion
- ⊗ Agree
- ◯ Strongly agree

e. I plan to do short term (< 6 months) international work when I finish residency.

- ◯ Strongly disagree
- ◯ Disagree
- ⊗ No opinion
- ◯ Agree
- ◯ Strongly agree

f. I plan to do long term (>6 months) international work when I finish residency.

- ◯ Strongly disagree
- ◯ Disagree
- ⊗ No opinion
- ◯ Agree
- ◯ Strongly agree

g. I plan to work in health disparities in the following way after residency:

Providing care to underserved and under/non-insured population

SECTION B: MEDICAL EDUCATION

A. Please indicate your level of agreement with the following statements regarding your medical education and knowledge about immigrants and refugees by checking the box that best represents your opinion.

a. I have received specialized training in immigrant and refugee health, tropical medicine, or cross-cultural health.

- ◯ Strongly disagree
- ◯ Disagree
- ◯ No opinion
- ⊗ Agree
- ◯ Strongly agree

b. If you have received specialized training in immigrant and refugee health, tropical medicine, or cross-cultural health, please indicate all the contexts in which you received this training:

- ☐ As an undergraduate.
- ☒ As a medical student.
- ☐ As part of my residency.
- ☐ A special program.
- ☐ As part of my fellowship.
- ☐ As part of a degree program (e.g. MPH)
- ☐ Other:

c. I feel comfortable with my fund of knowledge regarding immigrant and refugee health.

- ◯ Strongly disagree
- ⊗ Disagree
- ◯ No opinion
- ◯ Agree
- ◯ Strongly agree

d. I would like to have further training in immigrant and refugee health.

- ◯ Strongly disagree
- ◯ Disagree
- ◯ No opinion
- ⊗ Agree
- ◯ Strongly agree

e. If you agree with the above, please indicate all the contexts in which you would like to receive this training:
□□

- ☒ As part of my residency.
- ☒ A special program.
- ☐ As part of my fellowship.

SECTION C: Attitudes towards immigrant health

A.□Please indicate your level of agreement with the following statements regarding immigrant and refugee health by checking the box that best represents your opinion.

a. I enjoy taking care of immigrants and refugees.

- ◯ Never
- ◯ Rarely
- ◯ Sometimes
- ⊗ Usually
- ◯ Always

b. Please indicate the reasons that you enjoy taking care of immigrants and refugees (may choose more than one).

- ☒ Tropical and other conditions not frequently diagnosed in US-born patients
- ☒ Learning about other cultures
- ☐ They don't complain as much
- ☐ Being able to hear their stories
- ☒ Their care is more complicated
- ☐ Their care is less complicated
- ☐ They are very appreciative of your help.
- ☐ They are extremely vulnerable
- ☐ Other:

c. Taking care of immigrants and refugees is more challenging than taking care of US born patients.

- ◯ Never
- ◯ Rarely
- ⊗ Sometimes
- ◯ Usually
- ◯ Always

d. Please mark all the challenges that you face as a provider when providing care to immigrants and refugees (may choose more than one):

- ☒ Language barriers
- ☐ Insurance barriers
- ☒ Cultural barriers
- ☒ Finding a professional interpreter
- ☐ Knowing how to work with a professional interpreter
- ☐ Time constraints
- ☒ My own knowledge related to tropical and travel medicine
- ☐ Transportation problems for the patient
- ☒ Patients not understanding treatment plan
- ☒ Patients not following treatment plan
- ☐ My lack of knowledge regarding the patient's culture
- ☒ Bias or stereotyping
- ☐ Other:

e. Please mark all of the challenges faced by immigrant and refugee populations when receiving healthcare that you have perceived or witnessed (may choose more than one):

- ☒ Language barriers
- ☒ Insurance barriers
- ☒ Cultural barriers
- ☒ Finding a professional interpreter
- ☒ Knowing how to work with a professional interpreter
- ☐ Time constraints
- ☒ Insufficiently trained health care providers
- ☐ Transportation problems for the patient
- ☐ Food insufficiency
- ☐ Need for child care
- ☒ Patients not understanding treatment plan
- ☒ Patients not following treatment plan
- ☒ My lack of knowledge regarding the patient's culture
- ☒ Bias or stereotyping
- ☐ Trust issues
- ☐ Other...

f. Rank how well immigrants and refugees understand the healthcare that you are trying to provide.

- ○ Significantly less than a US born individual
- ⊗ Less than a US born individual
- ○ Equivalent to a US born individual
- ○ More than a US born patient
- ○ Significantly more than a US born individual

g. Immigrants and refugees adhere to treatment plans and follow my recommendations.

- ○ Never
- ○ Rarely
- ⊗ Sometimes
- ○ Usually
- ○ Always

h. Immigrants and refugees should receive the same care and insurance coverage as US born patients.

- ○ Never
- ○ Rarely
- ○ Sometimes
- ○ Usually
- ⊗ Always

i. Immigrants and refugees who are undocumented should receive the same care and insurance coverage as US born patients.

- ○ Never
- ○ Rarely
- ○ Sometimes
- ○ Usually
- ⊗ Always

j. Every physician is professionally obligated to care for immigrants and refugees if they present to your clinic or hospital.

- ○ Strongly disagree
- ○ Disagree
- ○ No opinion
- ⊗ Agree
- ○ Strongly agree

k. Is healthcare a human right?
⊗ Yes
◯ No

B. If you wish, please tell us about what you enjoy or do not enjoy about immigrant and refugee health care and the greatest challenges you face in caring for this population.
____

SECTION D: DEMOGRAPHIC INFORMATION
Please answer the following questions by checking the box in front of the response choice that best describes you.

a. Your age?
◯ 20 to 24
⊗ 25 to 29
◯ 30 to 34
◯ 35 to 39
◯ 40 or older

b. Your gender?
◯ Female
⊗ Male
◯ Other

c. Are you Hispanic or Latino?
◯ Yes
⊗ No

d. What is your race? (Select one or more responses)
◯ American Indian or Alaska Native
◯ Asian (Please specify):
◯ Black or African American
◯ Native Hawaiian or Other Pacific Islander
◯ White
⊗ Other (Please specify):

mixed asian-caucasian race

e. Were you born in the United States?
⊗ Yes
◯ No

g. Your residency year?
◯ PGY1
⊗ PGY2
◯ PGY3
◯ PGY4
◯ PGY5

h. How would you classify your political ideology?
◯ Conservative
◯ Somewhat conservative
◯ Moderate
⊗ Somewhat liberal
◯ Liberal
◯ Other (Please specify):

i. Estimated level of educational debt?
◯ None
◯ Less than $50,000
◯ $50,000 - $100,000
◯ $100,000 - $200,000
⊗ $200,000 or more

j. Do you plan to subspecialize?
⊗ Yes
◯ No

k. Languages spoken?

- ☒ English
- ☒ Spanish
- ☒ French
- ☐ Hmong
- ☐ Somali
- ☐ Japanese
- ☐ Chinese
- ☐ Russian
- ☐ Ethiopian
- ☒ Other ___________

Hindi

l. Are you in the Global Health Pathway?

- ◯ Yes
- ⊗ No

m. Did you earn your degree in the US?

- ⊗ Yes
- ◯ No

n. What residency program are you in?

- ◯ Internal Medicine
- ◯ Med-Peds
- ◯ Pediatrics
- ◯ Family Practice
- ⊗ Neurology
- ◯ Psychiatry
- ◯ ObGyn
- ◯ Neurosurgery
- ◯ General Surgery
- ◯ Orthopedic Surgery
- ◯ Urology
- ◯ Surgical sub-specialty (please specify in text box below)
- ◯ Non-clinical specialty (radiology, pathology; please specify in text box below)

Medical Trainees' attitudes, knowledge, and experience with immigrant and refugee health

Response was added on 12/14/2013 7:44pm.

SECTION A: Personal experience with immigrant and refugee health care.

A. Please indicate your level of agreement with the following statements regarding your personal experience with immigrant and refugee health care by checking the box that best represents your experience.

a. During my inpatient rotations, I take care of the following percentage of immigrant and refugee patients:

◯ None
◯ 0 -5%
◯ 5-10%
⊗ 10-25%
◯ > 25%

b. During my outpatient rotations, I take care of the following percentage of immigrant and refugee patients:

◯ None
◯ 0-10%
◯ 10 -25%
⊗ 25-50%
◯ 50-75%
◯ >75%

c. I would like to take care of more immigrant and refugee patients.

◯ Strongly disagree
◯ Disagree
◯ No opinion
⊗ Agree
◯ Strongly agree

d. I plan to take care of immigrants and refugees when I finish residency.

◯ Strongly disagree
◯ Disagree
◯ No opinion
⊗ Agree
◯ Strongly agree

e. I plan to do short term (< 6 months) international work when I finish residency.

◯ Strongly disagree
◯ Disagree
◯ No opinion
⊗ Agree
◯ Strongly agree

f. I plan to do long term (>6 months) international work when I finish residency.

◯ Strongly disagree
◯ Disagree
◯ No opinion
⊗ Agree
◯ Strongly agree

g. I plan to work in health disparities in the following way after residency:

Provide health education and advocacy for under served communities

SECTION B: MEDICAL EDUCATION

A. Please indicate your level of agreement with the following statements regarding your medical education and knowledge about immigrants and refugees by checking the box that best represents your opinion.

a. I have received specialized training in immigrant and refugee health, tropical medicine, or cross-cultural health.

- ◯ Strongly disagree
- ◯ Disagree
- ◯ No opinion
- ⊗ Agree
- ◯ Strongly agree

b. If you have received specialized training in immigrant and refugee health, tropical medicine, or cross-cultural health, please indicate all the contexts in which you received this training:

- ☐ As an undergraduate.
- ☐ As a medical student.
- ☐ As part of my residency.
- ☐ A special program.
- ☐ As part of my fellowship.
- ☒ As part of a degree program (e.g. MPH)
- ☐ Other:

c. I feel comfortable with my fund of knowledge regarding immigrant and refugee health.

- ◯ Strongly disagree
- ◯ Disagree
- ◯ No opinion
- ⊗ Agree
- ◯ Strongly agree

d. I would like to have further training in immigrant and refugee health.

- ◯ Strongly disagree
- ◯ Disagree
- ◯ No opinion
- ⊗ Agree
- ◯ Strongly agree

e. If you agree with the above, please indicate all the contexts in which you would like to receive this training:

- ☒ As part of my residency.
- ☐ A special program.
- ☐ As part of my fellowship.

SECTION C: Attitudes towards immigrant health

A. Please indicate your level of agreement with the following statements regarding immigrant and refugee health by checking the box that best represents your opinion.

a. I enjoy taking care of immigrants and refugees.

- ◯ Never
- ◯ Rarely
- ◯ Sometimes
- ⊗ Usually
- ◯ Always

b. Please indicate the reasons that you enjoy taking care of immigrants and refugees (may choose more than one).

- ☒ Tropical and other conditions not frequently diagnosed in US-born patients
- ☐ Learning about other cultures
- ☐ They don't complain as much
- ☐ Being able to hear their stories
- ☐ Their care is more complicated
- ☐ Their care is less complicated
- ☐ They are very appreciative of your help.
- ☐ They are extremely vulnerable
- ☐ Other:

c. Taking care of immigrants and refugees is more challenging than taking care of US born patients.

- ◯ Never
- ◯ Rarely
- ⊗ Sometimes
- ◯ Usually
- ◯ Always

d. Please mark all the challenges that you face as a provider when providing care to immigrants and refugees (may choose more than one):

- [x] Language barriers
- [ ] Insurance barriers
- [x] Cultural barriers
- [ ] Finding a professional interpreter
- [ ] Knowing how to work with a professional interpreter
- [ ] Time constraints
- [ ] My own knowledge related to tropical and travel medicine
- [ ] Transportation problems for the patient
- [ ] Patients not understanding treatment plan
- [ ] Patients not following treatment plan
- [ ] My lack of knowledge regarding the patient's culture
- [ ] Bias or stereotyping
- [ ] Other:

e. Please mark all of the challenges faced by immigrant and refugee populations when receiving healthcare that you have perceived or witnessed (may choose more than one):

- [x] Language barriers
- [ ] Insurance barriers
- [x] Cultural barriers
- [ ] Finding a professional interpreter
- [ ] Knowing how to work with a professional interpreter
- [x] Time constraints
- [ ] Insufficiently trained health care providers
- [ ] Transportation problems for the patient
- [ ] Food insufficiency
- [ ] Need for child care
- [ ] Patients not understanding treatment plan
- [ ] Patients not following treatment plan
- [ ] My lack of knowledge regarding the patient's culture
- [ ] Bias or stereotyping
- [ ] Trust issues
- [ ] Other...

f. Rank how well immigrants and refugees understand the healthcare that you are trying to provide.

- ○ Significantly less than a US born individual
- ⊗ Less than a US born individual
- ○ Equivalent to a US born individual
- ○ More than a US born patient
- ○ Significantly more than a US born individual

g. Immigrants and refugees adhere to treatment plans and follow my recommendations.

- ○ Never
- ○ Rarely
- ○ Sometimes
- ⊗ Usually
- ○ Always

h. Immigrants and refugees should receive the same care and insurance coverage as US born patients.

- ○ Never
- ○ Rarely
- ○ Sometimes
- ○ Usually
- ⊗ Always

i. Immigrants and refugees who are undocumented should receive the same care and insurance coverage as US born patients.

- ○ Never
- ○ Rarely
- ⊗ Sometimes
- ○ Usually
- ○ Always

j. Every physician is professionally obligated to care for immigrants and refugees if they present to your clinic or hospital.

- ○ Strongly disagree
- ○ Disagree
- ○ No opinion
- ⊗ Agree
- ○ Strongly agree

k. Is healthcare a human right?

⊗ Yes
◯ No

B. If you wish, please tell us about what you enjoy or do not enjoy about immigrant and refugee health care and the greatest challenges you face in caring for this population.

____

SECTION D: DEMOGRAPHIC INFORMATION
Please answer the following questions by checking the box in front of the response choice that best describes you.

a. Your age?

◯ 20 to 24
◯ 25 to 29
◯ 30 to 34
⊗ 35 to 39
◯ 40 or older

b. Your gender?

◯ Female
⊗ Male
◯ Other

c.Are you Hispanic or Latino?

◯ Yes
⊗ No

d. What is your race? (Select one or more responses)

◯ American Indian or Alaska Native
◯ Asian (Please specify):
⊗ Black or African American
◯ Native Hawaiian or Other Pacific Islander
◯ White
◯ Other (Please specify):

e.Were you born in the United States?

◯ Yes
⊗ No

f.If not, in what country were you born?

____

g. Your residency year?

⊗ PGY1
◯ PGY2
◯ PGY3
◯ PGY4
◯ PGY5

h. How would you classify your political ideology?

◯ Conservative
⊗ Somewhat conservative
◯ Moderate
◯ Somewhat liberal
◯ Liberal
◯ Other (Please specify):

i. Estimated level of educational debt?

◯ None
⊗ Less than $50,000
◯ $50,000 - $100,000
◯ $100,000 - $200,000
◯ $200,000 or more

j.Do you plan to subspecialize?

⊗ Yes
◯ No

k. Languages spoken?

- [x] English
- [ ] Spanish
- [ ] French
- [ ] Hmong
- [ ] Somali
- [ ] Japanese
- [ ] Chinese
- [ ] Russian
- [ ] Ethiopian
- [ ] Other ___________

l. Are you in the Global Health Pathway?

- (x) Yes
- ( ) No

m. Did you earn your degree in the US?

- ( ) Yes
- (x) No

n. What residency program are you in?

- ( ) Internal Medicine
- ( ) Med-Peds
- (x) Pediatrics
- ( ) Family Practice
- ( ) Neurology
- ( ) Psychiatry
- ( ) ObGyn
- ( ) Neurosurgery
- ( ) General Surgery
- ( ) Orthopedic Surgery
- ( ) Urology
- ( ) Surgical sub-specialty (please specify in text box below)
- ( ) Non-clinical specialty (radiology, pathology; please specify in text box below)

Medical Trainees' attitudes, knowledge, and experience with immigrant and refugee health

Response was added on 12/14/2013 9:37pm.

SECTION A: Personal experience with immigrant and refugee health care.

A. Please indicate your level of agreement with the following statements regarding your personal experience with immigrant and refugee health care by checking the box that best represents your experience.

a. During my inpatient rotations, I take care of the following percentage of immigrant and refugee patients:

- ◯ None
- ◯ 0 -5%
- ⊗ 5-10%
- ◯ 10-25%
- ◯ > 25%

b. During my outpatient rotations, I take care of the following percentage of immigrant and refugee patients:

- ◯ None
- ◯ 0-10%
- ⊗ 10 -25%
- ◯ 25-50%
- ◯ 50-75%
- ◯ >75%

c. I would like to take care of more immigrant and refugee patients.

- ◯ Strongly disagree
- ◯ Disagree
- ⊗ No opinion
- ◯ Agree
- ◯ Strongly agree

d. I plan to take care of immigrants and refugees when I finish residency.

- ◯ Strongly disagree
- ◯ Disagree
- ◯ No opinion
- ⊗ Agree
- ◯ Strongly agree

e. I plan to do short term (< 6 months) international work when I finish residency.

- ◯ Strongly disagree
- ⊗ Disagree
- ◯ No opinion
- ◯ Agree
- ◯ Strongly agree

f. I plan to do long term (>6 months) international work when I finish residency.

- ◯ Strongly disagree
- ⊗ Disagree
- ◯ No opinion
- ◯ Agree
- ◯ Strongly agree

g. I plan to work in health disparities in the following way after residency:

Addiction medicine: closing intergenerational health status gap associated with poverty, child and domestic abuse, and substance use

SECTION B: MEDICAL EDUCATION

A. Please indicate your level of agreement with the following statements regarding your medical education and knowledge about immigrants and refugees by checking the box that best represents your opinion.

a. I have received specialized training in immigrant and refugee health, tropical medicine, or cross-cultural health.

- ◯ Strongly disagree
- ⊗ Disagree
- ◯ No opinion
- ◯ Agree
- ◯ Strongly agree

c. I feel comfortable with my fund of knowledge regarding immigrant and refugee health.

- ◯ Strongly disagree
- ◯ Disagree
- ⊗ No opinion
- ◯ Agree
- ◯ Strongly agree

d. I would like to have further training in immigrant and refugee health.

- ◯ Strongly disagree
- ◯ Disagree
- ◯ No opinion
- ⊗ Agree
- ◯ Strongly agree

e. If you agree with the above, please indicate all the contexts in which you would like to receive this training:
□□

- ☒ As part of my residency.
- ☐ A special program.
- ☒ As part of my fellowship.

SECTION C: Attitudes towards immigrant health

A.□Please indicate your level of agreement with the following statements regarding immigrant and refugee health by checking the box that best represents your opinion.

a. I enjoy taking care of immigrants and refugees.

- ◯ Never
- ◯ Rarely
- ◯ Sometimes
- ◯ Usually
- ⊗ Always

b. Please indicate the reasons that you enjoy taking care of immigrants and refugees (may choose more than one).

- ☐ Tropical and other conditions not frequently diagnosed in US-born patients
- ☒ Learning about other cultures
- ☐ They don't complain as much
- ☒ Being able to hear their stories
- ☒ Their care is more complicated
- ☐ Their care is less complicated
- ☒ They are very appreciative of your help.
- ☒ They are extremely vulnerable
- ☐ Other:

c. Taking care of immigrants and refugees is more challenging than taking care of US born patients.

- ◯ Never
- ◯ Rarely
- ⊗ Sometimes
- ◯ Usually
- ◯ Always

d. Please mark all the challenges that you face as a provider when providing care to immigrants and refugees (may choose more than one):

- [x] Language barriers
- [ ] Insurance barriers
- [x] Cultural barriers
- [ ] Finding a professional interpreter
- [ ] Knowing how to work with a professional interpreter
- [x] Time constraints
- [x] My own knowledge related to tropical and travel medicine
- [x] Transportation problems for the patient
- [ ] Patients not understanding treatment plan
- [x] Patients not following treatment plan
- [ ] My lack of knowledge regarding the patient's culture
- [ ] Bias or stereotyping
- [ ] Other:

e. Please mark all of the challenges faced by immigrant and refugee populations when receiving healthcare that you have perceived or witnessed (may choose more than one):

- [x] Language barriers
- [x] Insurance barriers
- [x] Cultural barriers
- [ ] Finding a professional interpreter
- [x] Knowing how to work with a professional interpreter
- [x] Time constraints
- [ ] Insufficiently trained health care providers
- [x] Transportation problems for the patient
- [ ] Food insufficiency
- [x] Need for child care
- [x] Patients not understanding treatment plan
- [x] Patients not following treatment plan
- [x] My lack of knowledge regarding the patient's culture
- [ ] Bias or stereotyping
- [ ] Trust issues
- [ ] Other...

f. Rank how well immigrants and refugees understand the healthcare that you are trying to provide.

- (x) Significantly less than a US born individual
- ( ) Less than a US born individual
- ( ) Equivalent to a US born individual
- ( ) More than a US born patient
- ( ) Significantly more than a US born individual

g. Immigrants and refugees adhere to treatment plans and follow my recommendations.

- ( ) Never
- ( ) Rarely
- (x) Sometimes
- ( ) Usually
- ( ) Always

h. Immigrants and refugees should receive the same care and insurance coverage as US born patients.

- ( ) Never
- ( ) Rarely
- ( ) Sometimes
- ( ) Usually
- (x) Always

i. Immigrants and refugees who are undocumented should receive the same care and insurance coverage as US born patients.

- ( ) Never
- ( ) Rarely
- ( ) Sometimes
- (x) Usually
- ( ) Always

j. Every physician is professionally obligated to care for immigrants and refugees if they present to your clinic or hospital.

- ( ) Strongly disagree
- ( ) Disagree
- ( ) No opinion
- ( ) Agree
- (x) Strongly agree

k. Is healthcare a human right?
- ⊗ Yes
- ◯ No

B. If you wish, please tell us about what you enjoy or do not enjoy about immigrant and refugee health care and the greatest challenges you face in caring for this population.

____

SECTION D: DEMOGRAPHIC INFORMATION
Please answer the following questions by checking the box in front of the response choice that best describes you.

a. Your age?
- ◯ 20 to 24
- ◯ 25 to 29
- ⊗ 30 to 34
- ◯ 35 to 39
- ◯ 40 or older

b. Your gender?
- ◯ Female
- ⊗ Male
- ◯ Other

c. Are you Hispanic or Latino?
- ◯ Yes
- ⊗ No

d. What is your race? (Select one or more responses)
- ◯ American Indian or Alaska Native
- ◯ Asian (Please specify):
- ◯ Black or African American
- ◯ Native Hawaiian or Other Pacific Islander
- ⊗ White
- ◯ Other (Please specify):

e. Were you born in the United States?
- ⊗ Yes
- ◯ No

g. Your residency year?
- ◯ PGY1
- ◯ PGY2
- ◯ PGY3
- ⊗ PGY4
- ◯ PGY5

h. How would you classify your political ideology?
- ◯ Conservative
- ◯ Somewhat conservative
- ◯ Moderate
- ⊗ Somewhat liberal
- ◯ Liberal
- ◯ Other (Please specify):

i. Estimated level of educational debt?
- ⊗ None
- ◯ Less than $50,000
- ◯ $50,000 - $100,000
- ◯ $100,000 - $200,000
- ◯ $200,000 or more

j. Do you plan to subspecialize?
- ⊗ Yes
- ◯ No

k. Languages spoken?

- ☒ English
- ☐ Spanish
- ☐ French
- ☐ Hmong
- ☐ Somali
- ☐ Japanese
- ☐ Chinese
- ☐ Russian
- ☐ Ethiopian
- ☐ Other ___________

l. Are you in the Global Health Pathway?

- ◯ Yes
- ⊗ No

m. Did you earn your degree in the US?

- ⊗ Yes
- ◯ No

n. What residency program are you in?

- ◯ Internal Medicine
- ⊗ Med-Peds
- ◯ Pediatrics
- ◯ Family Practice
- ◯ Neurology
- ◯ Psychiatry
- ◯ ObGyn
- ◯ Neurosurgery
- ◯ General Surgery
- ◯ Orthopedic Surgery
- ◯ Urology
- ◯ Surgical sub-specialty (please specify in text box below)
- ◯ Non-clinical specialty (radiology, pathology; please specify in text box below)

Medical Trainees' attitudes, knowledge, and experience with immigrant and refugee health

Response was added on 12/15/2013 10:05am.

SECTION A: Personal experience with immigrant and refugee health care.

A. Please indicate your level of agreement with the following statements regarding your personal experience with immigrant and refugee health care by checking the box that best represents your experience.

a. During my inpatient rotations, I take care of the following percentage of immigrant and refugee patients:
- ◯ None
- ◯ 0 -5%
- ◯ 5-10%
- ◯ 10-25%
- ⊗ > 25%

b. During my outpatient rotations, I take care of the following percentage of immigrant and refugee patients:
- ◯ None
- ◯ 0-10%
- ◯ 10 -25%
- ◯ 25-50%
- ⊗ 50-75%
- ◯ >75%

c. I would like to take care of more immigrant and refugee patients.
- ◯ Strongly disagree
- ◯ Disagree
- ◯ No opinion
- ◯ Agree
- ⊗ Strongly agree

d. I plan to take care of immigrants and refugees when I finish residency.
- ◯ Strongly disagree
- ◯ Disagree
- ◯ No opinion
- ⊗ Agree
- ◯ Strongly agree

e. I plan to do short term (< 6 months) international work when I finish residency.
- ◯ Strongly disagree
- ◯ Disagree
- ⊗ No opinion
- ◯ Agree
- ◯ Strongly agree

f. I plan to do long term (>6 months) international work when I finish residency.
- ◯ Strongly disagree
- ⊗ Disagree
- ◯ No opinion
- ◯ Agree
- ◯ Strongly agree

g. I plan to work in health disparities in the following way after residency:
Working with Native Americans

SECTION B: MEDICAL EDUCATION

A. Please indicate your level of agreement with the following statements regarding your medical education and knowledge about immigrants and refugees by checking the box that best represents your opinion.

a. I have received specialized training in immigrant and refugee health, tropical medicine, or cross-cultural health.

- ◯ Strongly disagree
- ◯ Disagree
- ◯ No opinion
- ⊗ Agree
- ◯ Strongly agree

b. If you have received specialized training in immigrant and refugee health, tropical medicine, or cross-cultural health, please indicate all the contexts in which you received this training:

- ☐ As an undergraduate.
- ☐ As a medical student.
- ☒ As part of my residency.
- ☐ A special program.
- ☐ As part of my fellowship.
- ☐ As part of a degree program (e.g. MPH)
- ☐ Other:

c. I feel comfortable with my fund of knowledge regarding immigrant and refugee health.

- ◯ Strongly disagree
- ◯ Disagree
- ◯ No opinion
- ⊗ Agree
- ◯ Strongly agree

d. I would like to have further training in immigrant and refugee health.

- ◯ Strongly disagree
- ◯ Disagree
- ◯ No opinion
- ◯ Agree
- ⊗ Strongly agree

e. If you agree with the above, please indicate all the contexts in which you would like to receive this training:
□□

- ☒ As part of my residency.
- ☒ A special program.
- ☐ As part of my fellowship.

SECTION C: Attitudes towards immigrant health

A.□Please indicate your level of agreement with the following statements regarding immigrant and refugee health by checking the box that best represents your opinion.

a. I enjoy taking care of immigrants and refugees.

- ◯ Never
- ◯ Rarely
- ◯ Sometimes
- ◯ Usually
- ⊗ Always

b. Please indicate the reasons that you enjoy taking care of immigrants and refugees (may choose more than one).

- ☐ Tropical and other conditions not frequently diagnosed in US-born patients
- ☒ Learning about other cultures
- ☐ They don't complain as much
- ☒ Being able to hear their stories
- ☒ Their care is more complicated
- ☐ Their care is less complicated
- ☒ They are very appreciative of your help.
- ☐ They are extremely vulnerable
- ☐ Other:

c. Taking care of immigrants and refugees is more challenging than taking care of US born patients.

- ◯ Never
- ◯ Rarely
- ◯ Sometimes
- ⊗ Usually
- ◯ Always

d. Please mark all the challenges that you face as a provider when providing care to immigrants and refugees (may choose more than one):

- ☒ Language barriers
- ☒ Insurance barriers
- ☒ Cultural barriers
- ☒ Finding a professional interpreter
- ☐ Knowing how to work with a professional interpreter
- ☒ Time constraints
- ☒ My own knowledge related to tropical and travel medicine
- ☒ Transportation problems for the patient
- ☒ Patients not understanding treatment plan
- ☒ Patients not following treatment plan
- ☒ My lack of knowledge regarding the patient's culture
- ☐ Bias or stereotyping
- ☐ Other:

e. Please mark all of the challenges faced by immigrant and refugee populations when receiving healthcare that you have perceived or witnessed (may choose more than one):

- ☒ Language barriers
- ☒ Insurance barriers
- ☒ Cultural barriers
- ☒ Finding a professional interpreter
- ☐ Knowing how to work with a professional interpreter
- ☐ Time constraints
- ☒ Insufficiently trained health care providers
- ☒ Transportation problems for the patient
- ☒ Food insufficiency
- ☒ Need for child care
- ☒ Patients not understanding treatment plan
- ☒ Patients not following treatment plan
- ☐ My lack of knowledge regarding the patient's culture
- ☐ Bias or stereotyping
- ☐ Trust issues
- ☐ Other...

f. Rank how well immigrants and refugees understand the healthcare that you are trying to provide.

- ⊗ Significantly less than a US born individual
- ◯ Less than a US born individual
- ◯ Equivalent to a US born individual
- ◯ More than a US born patient
- ◯ Significantly more than a US born individual

g. Immigrants and refugees adhere to treatment plans and follow my recommendations.

- ◯ Never
- ◯ Rarely
- ◯ Sometimes
- ⊗ Usually
- ◯ Always

h. Immigrants and refugees should receive the same care and insurance coverage as US born patients.

- ◯ Never
- ◯ Rarely
- ◯ Sometimes
- ◯ Usually
- ⊗ Always

i. Immigrants and refugees who are undocumented should receive the same care and insurance coverage as US born patients.

- ◯ Never
- ◯ Rarely
- ⊗ Sometimes
- ◯ Usually
- ◯ Always

j. Every physician is professionally obligated to care for immigrants and refugees if they present to your clinic or hospital.

- ◯ Strongly disagree
- ◯ Disagree
- ◯ No opinion
- ◯ Agree
- ⊗ Strongly agree

k. Is healthcare a human right?

⊗ Yes
◯ No

B. If you wish, please tell us about what you enjoy or do not enjoy about immigrant and refugee health care and the greatest challenges you face in caring for this population.

____

SECTION D: DEMOGRAPHIC INFORMATION
Please answer the following questions by checking the box in front of the response choice that best describes you.

a. Your age?

◯ 20 to 24
⊗ 25 to 29
◯ 30 to 34
◯ 35 to 39
◯ 40 or older

b. Your gender?

⊗ Female
◯ Male
◯ Other

c.Are you Hispanic or Latino?

◯ Yes
⊗ No

d. What is your race? (Select one or more responses)

◯ American Indian or Alaska Native
◯ Asian (Please specify):
◯ Black or African American
◯ Native Hawaiian or Other Pacific Islander
⊗ White
◯ Other (Please specify):

e.Were you born in the United States?

⊗ Yes
◯ No

g. Your residency year?

◯ PGY1
◯ PGY2
⊗ PGY3
◯ PGY4
◯ PGY5

h. How would you classify your political ideology?

◯ Conservative
◯ Somewhat conservative
◯ Moderate
⊗ Somewhat liberal
◯ Liberal
◯ Other (Please specify):

i. Estimated level of educational debt?

◯ None
◯ Less than $50,000
◯ $50,000 - $100,000
◯ $100,000 - $200,000
⊗ $200,000 or more

j.Do you plan to subspecialize?

◯ Yes
⊗ No

k. Languages spoken?

- ☒ English
- ☐ Spanish
- ☐ French
- ☐ Hmong
- ☐ Somali
- ☐ Japanese
- ☐ Chinese
- ☐ Russian
- ☐ Ethiopian
- ☐ Other ___________

l. Are you in the Global Health Pathway?

- ◯ Yes
- ⊗ No

m. Did you earn your degree in the US?

- ⊗ Yes
- ◯ No

n. What residency program are you in?

- ◯ Internal Medicine
- ◯ Med-Peds
- ◯ Pediatrics
- ⊗ Family Practice
- ◯ Neurology
- ◯ Psychiatry
- ◯ ObGyn
- ◯ Neurosurgery
- ◯ General Surgery
- ◯ Orthopedic Surgery
- ◯ Urology
- ◯ Surgical sub-specialty (please specify in text box below)
- ◯ Non-clinical specialty (radiology, pathology; please specify in text box below)

Medical Trainees' attitudes, knowledge, and experience with immigrant and refugee health

Response was added on 12/15/2013 2:22pm.

SECTION A: Personal experience with immigrant and refugee health care.

A. Please indicate your level of agreement with the following statements regarding your personal experience with immigrant and refugee health care by checking the box that best represents your experience.

a. During my inpatient rotations, I take care of the following percentage of immigrant and refugee patients:

- ◯ None
- ◯ 0 -5%
- ⊗ 5-10%
- ◯ 10-25%
- ◯ > 25%

b. During my outpatient rotations, I take care of the following percentage of immigrant and refugee patients:

- ◯ None
- ⊗ 0-10%
- ◯ 10 -25%
- ◯ 25-50%
- ◯ 50-75%
- ◯ >75%

c. I would like to take care of more immigrant and refugee patients.

- ◯ Strongly disagree
- ◯ Disagree
- ⊗ No opinion
- ◯ Agree
- ◯ Strongly agree

d. I plan to take care of immigrants and refugees when I finish residency.

- ◯ Strongly disagree
- ◯ Disagree
- ⊗ No opinion
- ◯ Agree
- ◯ Strongly agree

e. I plan to do short term (< 6 months) international work when I finish residency.

- ◯ Strongly disagree
- ⊗ Disagree
- ◯ No opinion
- ◯ Agree
- ◯ Strongly agree

f. I plan to do long term (>6 months) international work when I finish residency.

- ◯ Strongly disagree
- ⊗ Disagree
- ◯ No opinion
- ◯ Agree
- ◯ Strongly agree

g. I plan to work in health disparities in the following way after residency:

I don't know yet.

SECTION B: MEDICAL EDUCATION

A. Please indicate your level of agreement with the following statements regarding your medical education and knowledge about immigrants and refugees by checking the box that best represents your opinion.

a. I have received specialized training in immigrant and refugee health, tropical medicine, or cross-cultural health.

- ⊗ Strongly disagree
- ◯ Disagree
- ◯ No opinion
- ◯ Agree
- ◯ Strongly agree

c. I feel comfortable with my fund of knowledge regarding immigrant and refugee health.

- ◯ Strongly disagree
- ⊗ Disagree
- ◯ No opinion
- ◯ Agree
- ◯ Strongly agree

d. I would like to have further training in immigrant and refugee health.

- ◯ Strongly disagree
- ◯ Disagree
- ◯ No opinion
- ⊗ Agree
- ◯ Strongly agree

e. If you agree with the above, please indicate all the contexts in which you would like to receive this training:
□□

- ☒ As part of my residency.
- ☐ A special program.
- ☐ As part of my fellowship.

SECTION C: Attitudes towards immigrant health

A.□Please indicate your level of agreement with the following statements regarding immigrant and refugee health by checking the box that best represents your opinion.

a. I enjoy taking care of immigrants and refugees.

- ◯ Never
- ◯ Rarely
- ◯ Sometimes
- ⊗ Usually
- ◯ Always

b. Please indicate the reasons that you enjoy taking care of immigrants and refugees (may choose more than one).

- ☒ Tropical and other conditions not frequently diagnosed in US-born patients
- ☒ Learning about other cultures
- ☐ They don't complain as much
- ☐ Being able to hear their stories
- ☐ Their care is more complicated
- ☐ Their care is less complicated
- ☐ They are very appreciative of your help.
- ☐ They are extremely vulnerable
- ☐ Other:

c. Taking care of immigrants and refugees is more challenging than taking care of US born patients.

- ◯ Never
- ◯ Rarely
- ⊗ Sometimes
- ◯ Usually
- ◯ Always

d. Please mark all the challenges that you face as a provider when providing care to immigrants and refugees (may choose more than one):

- ☒ Language barriers
- ☒ Insurance barriers
- ☒ Cultural barriers
- ☐ Finding a professional interpreter
- ☐ Knowing how to work with a professional interpreter
- ☒ Time constraints
- ☒ My own knowledge related to tropical and travel medicine
- ☐ Transportation problems for the patient
- ☒ Patients not understanding treatment plan
- ☒ Patients not following treatment plan
- ☐ My lack of knowledge regarding the patient's culture
- ☐ Bias or stereotyping
- ☐ Other:

e. Please mark all of the challenges faced by immigrant and refugee populations when receiving healthcare that you have perceived or witnessed (may choose more than one):

- ☒ Language barriers
- ☒ Insurance barriers
- ☐ Cultural barriers
- ☐ Finding a professional interpreter
- ☐ Knowing how to work with a professional interpreter
- ☒ Time constraints
- ☐ Insufficiently trained health care providers
- ☒ Transportation problems for the patient
- ☒ Food insufficiency
- ☐ Need for child care
- ☒ Patients not understanding treatment plan
- ☒ Patients not following treatment plan
- ☐ My lack of knowledge regarding the patient's culture
- ☐ Bias or stereotyping
- ☒ Trust issues
- ☐ Other...

f. Rank how well immigrants and refugees understand the healthcare that you are trying to provide.

- ○ Significantly less than a US born individual
- ⊗ Less than a US born individual
- ○ Equivalent to a US born individual
- ○ More than a US born patient
- ○ Significantly more than a US born individual

g. Immigrants and refugees adhere to treatment plans and follow my recommendations.

- ○ Never
- ○ Rarely
- ○ Sometimes
- ⊗ Usually
- ○ Always

h. Immigrants and refugees should receive the same care and insurance coverage as US born patients.

- ○ Never
- ○ Rarely
- ○ Sometimes
- ○ Usually
- ⊗ Always

i. Immigrants and refugees who are undocumented should receive the same care and insurance coverage as US born patients.

- ○ Never
- ○ Rarely
- ○ Sometimes
- ○ Usually
- ⊗ Always

j. Every physician is professionally obligated to care for immigrants and refugees if they present to your clinic or hospital.

- ○ Strongly disagree
- ○ Disagree
- ○ No opinion
- ○ Agree
- ⊗ Strongly agree

k. Is healthcare a human right?

⊗ Yes
◯ No

B. If you wish, please tell us about what you enjoy or do not enjoy about immigrant and refugee health care and the greatest challenges you face in caring for this population.

____

SECTION D: DEMOGRAPHIC INFORMATION
Please answer the following questions by checking the box in front of the response choice that best describes you.

a. Your age?

◯ 20 to 24
◯ 25 to 29
◯ 30 to 34
◯ 35 to 39
⊗ 40 or older

b. Your gender?

⊗ Female
◯ Male
◯ Other

c. Are you Hispanic or Latino?

◯ Yes
⊗ No

d. What is your race? (Select one or more responses)

◯ American Indian or Alaska Native
◯ Asian (Please specify):
◯ Black or African American
◯ Native Hawaiian or Other Pacific Islander
⊗ White
◯ Other (Please specify):

e. Were you born in the United States?

◯ Yes
⊗ No

f. If not, in what country were you born?

Germany

g. Your residency year?

◯ PGY1
⊗ PGY2
◯ PGY3
◯ PGY4
◯ PGY5

h. How would you classify your political ideology?

◯ Conservative
◯ Somewhat conservative
◯ Moderate
◯ Somewhat liberal
⊗ Liberal
◯ Other (Please specify):

i. Estimated level of educational debt?

⊗ None
◯ Less than $50,000
◯ $50,000 - $100,000
◯ $100,000 - $200,000
◯ $200,000 or more

j. Do you plan to subspecialize?

⊗ Yes
◯ No

k. Languages spoken?

- ☒ English
- ☐ Spanish
- ☐ French
- ☐ Hmong
- ☐ Somali
- ☐ Japanese
- ☐ Chinese
- ☐ Russian
- ☐ Ethiopian
- ☒ Other __________

German

l. Are you in the Global Health Pathway?

- ◯ Yes
- ⊗ No

m. Did you earn your degree in the US?

- ◯ Yes
- ⊗ No

n. What residency program are you in?

- ◯ Internal Medicine
- ◯ Med-Peds
- ⊗ Pediatrics
- ◯ Family Practice
- ◯ Neurology
- ◯ Psychiatry
- ◯ ObGyn
- ◯ Neurosurgery
- ◯ General Surgery
- ◯ Orthopedic Surgery
- ◯ Urology
- ◯ Surgical sub-specialty (please specify in text box below)
- ◯ Non-clinical specialty (radiology, pathology; please specify in text box below)

Medical Trainees' attitudes, knowledge, and experience with immigrant and refugee health

Response was added on 12/15/2013 5:39pm.

SECTION A: Personal experience with immigrant and refugee health care.

A. Please indicate your level of agreement with the following statements regarding your personal experience with immigrant and refugee health care by checking the box that best represents your experience.

a. During my inpatient rotations, I take care of the following percentage of immigrant and refugee patients:

- ◯ None
- ◯ 0 -5%
- ◯ 5-10%
- ⊗ 10-25%
- ◯ > 25%

b. During my outpatient rotations, I take care of the following percentage of immigrant and refugee patients:

- ◯ None
- ◯ 0-10%
- ◯ 10 -25%
- ⊗ 25-50%
- ◯ 50-75%
- ◯ >75%

c. I would like to take care of more immigrant and refugee patients.

- ◯ Strongly disagree
- ◯ Disagree
- ◯ No opinion
- ◯ Agree
- ⊗ Strongly agree

d. I plan to take care of immigrants and refugees when I finish residency.

- ◯ Strongly disagree
- ◯ Disagree
- ⊗ No opinion
- ◯ Agree
- ◯ Strongly agree

e. I plan to do short term (< 6 months) international work when I finish residency.

- ◯ Strongly disagree
- ⊗ Disagree
- ◯ No opinion
- ◯ Agree
- ◯ Strongly agree

f. I plan to do long term (>6 months) international work when I finish residency.

- ◯ Strongly disagree
- ⊗ Disagree
- ◯ No opinion
- ◯ Agree
- ◯ Strongly agree

g. I plan to work in health disparities in the following way after residency:

Will be doing fellowship after residency so not clear this question is pertinent to me.

SECTION B: MEDICAL EDUCATION

A. Please indicate your level of agreement with the following statements regarding your medical education and knowledge about immigrants and refugees by checking the box that best represents your opinion.

a. I have received specialized training in immigrant and refugee health, tropical medicine, or cross-cultural health.

- ◯ Strongly disagree
- ◯ Disagree
- ◯ No opinion
- ⊗ Agree
- ◯ Strongly agree

b. If you have received specialized training in immigrant and refugee health, tropical medicine, or cross-cultural health, please indicate all the contexts in which you received this training:

- ☐ As an undergraduate.
- ☐ As a medical student.
- ☒ As part of my residency.
- ☐ A special program.
- ☐ As part of my fellowship.
- ☐ As part of a degree program (e.g. MPH)
- ☐ Other:

c. I feel comfortable with my fund of knowledge regarding immigrant and refugee health.

- ◯ Strongly disagree
- ◯ Disagree
- ◯ No opinion
- ⊗ Agree
- ◯ Strongly agree

d. I would like to have further training in immigrant and refugee health.

- ◯ Strongly disagree
- ◯ Disagree
- ◯ No opinion
- ◯ Agree
- ⊗ Strongly agree

e. If you agree with the above, please indicate all the contexts in which you would like to receive this training:
▯▯

- ☒ As part of my residency.
- ☐ A special program.
- ☐ As part of my fellowship.

SECTION C: Attitudes towards immigrant health

A.▯Please indicate your level of agreement with the following statements regarding immigrant and refugee health by checking the box that best represents your opinion.

a. I enjoy taking care of immigrants and refugees.

- ◯ Never
- ◯ Rarely
- ◯ Sometimes
- ◯ Usually
- ⊗ Always

b. Please indicate the reasons that you enjoy taking care of immigrants and refugees (may choose more than one).

- ☐ Tropical and other conditions not frequently diagnosed in US-born patients
- ☒ Learning about other cultures
- ☐ They don't complain as much
- ☒ Being able to hear their stories
- ☐ Their care is more complicated
- ☐ Their care is less complicated
- ☒ They are very appreciative of your help.
- ☒ They are extremely vulnerable
- ☐ Other:

c. Taking care of immigrants and refugees is more challenging than taking care of US born patients.

- ◯ Never
- ◯ Rarely
- ⊗ Sometimes
- ◯ Usually
- ◯ Always

d. Please mark all the challenges that you face as a provider when providing care to immigrants and refugees (may choose more than one):

- ☒ Language barriers
- ☒ Insurance barriers
- ☐ Cultural barriers
- ☐ Finding a professional interpreter
- ☐ Knowing how to work with a professional interpreter
- ☒ Time constraints
- ☒ My own knowledge related to tropical and travel medicine
- ☒ Transportation problems for the patient
- ☒ Patients not understanding treatment plan
- ☒ Patients not following treatment plan
- ☒ My lack of knowledge regarding the patient's culture
- ☐ Bias or stereotyping
- ☐ Other:

e. Please mark all of the challenges faced by immigrant and refugee populations when receiving healthcare that you have perceived or witnessed (may choose more than one):

- ☒ Language barriers
- ☒ Insurance barriers
- ☐ Cultural barriers
- ☐ Finding a professional interpreter
- ☐ Knowing how to work with a professional interpreter
- ☐ Time constraints
- ☐ Insufficiently trained health care providers
- ☒ Transportation problems for the patient
- ☒ Food insufficiency
- ☐ Need for child care
- ☒ Patients not understanding treatment plan
- ☐ Patients not following treatment plan
- ☒ My lack of knowledge regarding the patient's culture
- ☐ Bias or stereotyping
- ☐ Trust issues
- ☐ Other...

f. Rank how well immigrants and refugees understand the healthcare that you are trying to provide.

- ⊗ Significantly less than a US born individual
- ○ Less than a US born individual
- ○ Equivalent to a US born individual
- ○ More than a US born patient
- ○ Significantly more than a US born individual

g. Immigrants and refugees adhere to treatment plans and follow my recommendations.

- ○ Never
- ○ Rarely
- ⊗ Sometimes
- ○ Usually
- ○ Always

h. Immigrants and refugees should receive the same care and insurance coverage as US born patients.

- ○ Never
- ○ Rarely
- ○ Sometimes
- ○ Usually
- ⊗ Always

i. Immigrants and refugees who are undocumented should receive the same care and insurance coverage as US born patients.

- ○ Never
- ○ Rarely
- ○ Sometimes
- ○ Usually
- ⊗ Always

j. Every physician is professionally obligated to care for immigrants and refugees if they present to your clinic or hospital.

- ○ Strongly disagree
- ○ Disagree
- ○ No opinion
- ○ Agree
- ⊗ Strongly agree

k. Is healthcare a human right?
- ⊗ Yes
- ◯ No

B. If you wish, please tell us about what you enjoy or do not enjoy about immigrant and refugee health care and the greatest challenges you face in caring for this population.

I enjoy taking care of immigrants because I, as a foreign born, know how hard it is to navigate the health care system. I would like to help them get the best care they could.

The greatest challenge is difficulty in communication. Despite the presence of professional interpretor, I found that many times, they do not understand treatment plan (or I did not effectively communicate with patients).

SECTION D: DEMOGRAPHIC INFORMATION
Please answer the following questions by checking the box in front of the response choice that best describes you.

a. Your age?
- ◯ 20 to 24
- ◯ 25 to 29
- ⊗ 30 to 34
- ◯ 35 to 39
- ◯ 40 or older

b. Your gender?
- ◯ Female
- ⊗ Male
- ◯ Other

c. Are you Hispanic or Latino?
- ◯ Yes
- ⊗ No

d. What is your race? (Select one or more responses)
- ◯ American Indian or Alaska Native
- ⊗ Asian (Please specify):
- ◯ Black or African American
- ◯ Native Hawaiian or Other Pacific Islander
- ◯ White
- ◯ Other (Please specify):

e. Were you born in the United States?
- ◯ Yes
- ⊗ No

f. If not, in what country were you born?

South Korea

g. Your residency year?
- ◯ PGY1
- ◯ PGY2
- ⊗ PGY3
- ◯ PGY4
- ◯ PGY5

h. How would you classify your political ideology?
- ◯ Conservative
- ◯ Somewhat conservative
- ⊗ Moderate
- ◯ Somewhat liberal
- ◯ Liberal
- ◯ Other (Please specify):

i. Estimated level of educational debt?
- ⊗ None
- ◯ Less than $50,000
- ◯ $50,000 - $100,000
- ◯ $100,000 - $200,000
- ◯ $200,000 or more

j.Do you plan to subspecialize?

- ⊗ Yes
- ◯ No

k. Languages spoken?

- ☐ English
- ☐ Spanish
- ☐ French
- ☐ Hmong
- ☐ Somali
- ☐ Japanese
- ☐ Chinese
- ☐ Russian
- ☐ Ethiopian
- ☒ Other ___________

Korean

l. Are you in the Global Health Pathway?

- ◯ Yes
- ⊗ No

m. Did you earn your degree in the US?

- ◯ Yes
- ⊗ No

n. What residency program are you in?

- ⊗ Internal Medicine
- ◯ Med-Peds
- ◯ Pediatrics
- ◯ Family Practice
- ◯ Neurology
- ◯ Psychiatry
- ◯ ObGyn
- ◯ Neurosurgery
- ◯ General Surgery
- ◯ Orthopedic Surgery
- ◯ Urology
- ◯ Surgical sub-specialty (please specify in text box below)
- ◯ Non-clinical specialty (radiology, pathology; please specify in text box below)

Medical Trainees' attitudes, knowledge, and experience with immigrant and refugee health

Response was added on 12/15/2013 6:18pm.

SECTION A: Personal experience with immigrant and refugee health care.

A. Please indicate your level of agreement with the following statements regarding your personal experience with immigrant and refugee health care by checking the box that best represents your experience.

a. During my inpatient rotations, I take care of the following percentage of immigrant and refugee patients:

- ◯ None
- ◯ 0 -5%
- ⊗ 5-10%
- ◯ 10-25%
- ◯ > 25%

b. During my outpatient rotations, I take care of the following percentage of immigrant and refugee patients:

- ◯ None
- ⊗ 0-10%
- ◯ 10 -25%
- ◯ 25-50%
- ◯ 50-75%
- ◯ >75%

c. I would like to take care of more immigrant and refugee patients.

- ◯ Strongly disagree
- ◯ Disagree
- ◯ No opinion
- ⊗ Agree
- ◯ Strongly agree

d. I plan to take care of immigrants and refugees when I finish residency.

- ◯ Strongly disagree
- ◯ Disagree
- ◯ No opinion
- ⊗ Agree
- ◯ Strongly agree

e. I plan to do short term (< 6 months) international work when I finish residency.

- ◯ Strongly disagree
- ⊗ Disagree
- ◯ No opinion
- ◯ Agree
- ◯ Strongly agree

f. I plan to do long term (>6 months) international work when I finish residency.

- ⊗ Strongly disagree
- ◯ Disagree
- ◯ No opinion
- ◯ Agree
- ◯ Strongly agree

g. I plan to work in health disparities in the following way after residency:

Would like to take care of low income patients.

SECTION B: MEDICAL EDUCATION

A. Please indicate your level of agreement with the following statements regarding your medical education and knowledge about immigrants and refugees by checking the box that best represents your opinion.

a. I have received specialized training in immigrant and refugee health, tropical medicine, or cross-cultural health.

○ Strongly disagree
○ Disagree
○ No opinion
⊗ Agree
○ Strongly agree

b. If you have received specialized training in immigrant and refugee health, tropical medicine, or cross-cultural health, please indicate all the contexts in which you received this training:

☐ As an undergraduate.
☒ As a medical student.
☒ As part of my residency.
☐ A special program.
☐ As part of my fellowship.
☐ As part of a degree program (e.g. MPH)
☐ Other:

c. I feel comfortable with my fund of knowledge regarding immigrant and refugee health.

○ Strongly disagree
⊗ Disagree
○ No opinion
○ Agree
○ Strongly agree

d. I would like to have further training in immigrant and refugee health.

○ Strongly disagree
○ Disagree
○ No opinion
⊗ Agree
○ Strongly agree

e. If you agree with the above, please indicate all the contexts in which you would like to receive this training:
□□

☒ As part of my residency.
☐ A special program.
☐ As part of my fellowship.

SECTION C: Attitudes towards immigrant health

A.□Please indicate your level of agreement with the following statements regarding immigrant and refugee health by checking the box that best represents your opinion.

a. I enjoy taking care of immigrants and refugees.

○ Never
○ Rarely
○ Sometimes
⊗ Usually
○ Always

b. Please indicate the reasons that you enjoy taking care of immigrants and refugees (may choose more than one).

☒ Tropical and other conditions not frequently diagnosed in US-born patients
☒ Learning about other cultures
☐ They don't complain as much
☐ Being able to hear their stories
☐ Their care is more complicated
☐ Their care is less complicated
☐ They are very appreciative of your help.
☐ They are extremely vulnerable
☐ Other:

c. Taking care of immigrants and refugees is more challenging than taking care of US born patients.

○ Never
○ Rarely
⊗ Sometimes
○ Usually
○ Always

d. Please mark all the challenges that you face as a provider when providing care to immigrants and refugees (may choose more than one):

- ☒ Language barriers
- ☐ Insurance barriers
- ☒ Cultural barriers
- ☐ Finding a professional interpreter
- ☐ Knowing how to work with a professional interpreter
- ☐ Time constraints
- ☒ My own knowledge related to tropical and travel medicine
- ☒ Transportation problems for the patient
- ☒ Patients not understanding treatment plan
- ☒ Patients not following treatment plan
- ☒ My lack of knowledge regarding the patient's culture
- ☐ Bias or stereotyping
- ☐ Other:

e. Please mark all of the challenges faced by immigrant and refugee populations when receiving healthcare that you have perceived or witnessed (may choose more than one):

- ☒ Language barriers
- ☒ Insurance barriers
- ☒ Cultural barriers
- ☐ Finding a professional interpreter
- ☒ Knowing how to work with a professional interpreter
- ☐ Time constraints
- ☐ Insufficiently trained health care providers
- ☒ Transportation problems for the patient
- ☐ Food insufficiency
- ☒ Need for child care
- ☒ Patients not understanding treatment plan
- ☒ Patients not following treatment plan
- ☒ My lack of knowledge regarding the patient's culture
- ☒ Bias or stereotyping
- ☒ Trust issues
- ☐ Other...

f. Rank how well immigrants and refugees understand the healthcare that you are trying to provide.

- ○ Significantly less than a US born individual
- ○ Less than a US born individual
- ⊗ Equivalent to a US born individual
- ○ More than a US born patient
- ○ Significantly more than a US born individual

g. Immigrants and refugees adhere to treatment plans and follow my recommendations.

- ○ Never
- ○ Rarely
- ⊗ Sometimes
- ○ Usually
- ○ Always

h. Immigrants and refugees should receive the same care and insurance coverage as US born patients.

- ○ Never
- ○ Rarely
- ○ Sometimes
- ⊗ Usually
- ○ Always

i. Immigrants and refugees who are undocumented should receive the same care and insurance coverage as US born patients.

- ○ Never
- ○ Rarely
- ○ Sometimes
- ⊗ Usually
- ○ Always

j. Every physician is professionally obligated to care for immigrants and refugees if they present to your clinic or hospital.

- ○ Strongly disagree
- ○ Disagree
- ○ No opinion
- ○ Agree
- ⊗ Strongly agree

k. Is healthcare a human right?

- ⊗ Yes
- ◯ No

B. If you wish, please tell us about what you enjoy or do not enjoy about immigrant and refugee health care and the greatest challenges you face in caring for this population.

____

SECTION D: DEMOGRAPHIC INFORMATION
Please answer the following questions by checking the box in front of the response choice that best describes you.

a. Your age?

- ◯ 20 to 24
- ⊗ 25 to 29
- ◯ 30 to 34
- ◯ 35 to 39
- ◯ 40 or older

b. Your gender?

- ⊗ Female
- ◯ Male
- ◯ Other

c.Are you Hispanic or Latino?

- ◯ Yes
- ⊗ No

d. What is your race? (Select one or more responses)

- ◯ American Indian or Alaska Native
- ◯ Asian (Please specify):
- ◯ Black or African American
- ◯ Native Hawaiian or Other Pacific Islander
- ⊗ White
- ◯ Other (Please specify):

e.Were you born in the United States?

- ⊗ Yes
- ◯ No

g. Your residency year?

- ◯ PGY1
- ◯ PGY2
- ⊗ PGY3
- ◯ PGY4
- ◯ PGY5

h. How would you classify your political ideology?

- ◯ Conservative
- ◯ Somewhat conservative
- ◯ Moderate
- ◯ Somewhat liberal
- ⊗ Liberal
- ◯ Other (Please specify):

i. Estimated level of educational debt?

- ◯ None
- ◯ Less than $50,000
- ◯ $50,000 - $100,000
- ⊗ $100,000 - $200,000
- ◯ $200,000 or more

j.Do you plan to subspecialize?

- ⊗ Yes
- ◯ No

k. Languages spoken?

☒ English
☒ Spanish
☐ French
☐ Hmong
☐ Somali
☐ Japanese
☐ Chinese
☐ Russian
☐ Ethiopian
☐ Other __________

l. Are you in the Global Health Pathway?

○ Yes
⊗ No

m. Did you earn your degree in the US?

⊗ Yes
○ No

n. What residency program are you in?

○ Internal Medicine
○ Med-Peds
○ Pediatrics
○ Family Practice
⊗ Neurology
○ Psychiatry
○ ObGyn
○ Neurosurgery
○ General Surgery
○ Orthopedic Surgery
○ Urology
○ Surgical sub-specialty (please specify in text box below)
○ Non-clinical specialty (radiology, pathology; please specify in text box below)

Medical Trainees' attitudes, knowledge, and experience with immigrant and refugee health

Response was added on 12/16/2013 10:41am.

SECTION A: Personal experience with immigrant and refugee health care.

A. Please indicate your level of agreement with the following statements regarding your personal experience with immigrant and refugee health care by checking the box that best represents your experience.

a. During my inpatient rotations, I take care of the following percentage of immigrant and refugee patients:
- ◯ None
- ◯ 0 -5%
- ◯ 5-10%
- ⊗ 10-25%
- ◯ > 25%

b. During my outpatient rotations, I take care of the following percentage of immigrant and refugee patients:
- ◯ None
- ◯ 0-10%
- ◯ 10 -25%
- ⊗ 25-50%
- ◯ 50-75%
- ◯ >75%

c. I would like to take care of more immigrant and refugee patients.
- ◯ Strongly disagree
- ◯ Disagree
- ⊗ No opinion
- ◯ Agree
- ◯ Strongly agree

d. I plan to take care of immigrants and refugees when I finish residency.
- ◯ Strongly disagree
- ◯ Disagree
- ◯ No opinion
- ⊗ Agree
- ◯ Strongly agree

e. I plan to do short term (< 6 months) international work when I finish residency.
- ◯ Strongly disagree
- ◯ Disagree
- ◯ No opinion
- ⊗ Agree
- ◯ Strongly agree

f. I plan to do long term (>6 months) international work when I finish residency.
- ◯ Strongly disagree
- ◯ Disagree
- ⊗ No opinion
- ◯ Agree
- ◯ Strongly agree

g. I plan to work in health disparities in the following way after residency:

To work on under-served areas of the world, for example in developiing countries.

SECTION B: MEDICAL EDUCATION

A. Please indicate your level of agreement with the following statements regarding your medical education and knowledge about immigrants and refugees by checking the box that best represents your opinion.

a. I have received specialized training in immigrant and refugee health, tropical medicine, or cross-cultural health.

- ◯ Strongly disagree
- ⊗ Disagree
- ◯ No opinion
- ◯ Agree
- ◯ Strongly agree

c. I feel comfortable with my fund of knowledge regarding immigrant and refugee health.

- ◯ Strongly disagree
- ◯ Disagree
- ◯ No opinion
- ⊗ Agree
- ◯ Strongly agree

d. I would like to have further training in immigrant and refugee health.

- ◯ Strongly disagree
- ◯ Disagree
- ◯ No opinion
- ⊗ Agree
- ◯ Strongly agree

e. If you agree with the above, please indicate all the contexts in which you would like to receive this training:

- ☒ As part of my residency.
- ☐ A special program.
- ☐ As part of my fellowship.

SECTION C: Attitudes towards immigrant health

A. Please indicate your level of agreement with the following statements regarding immigrant and refugee health by checking the box that best represents your opinion.

a. I enjoy taking care of immigrants and refugees.

- ◯ Never
- ◯ Rarely
- ◯ Sometimes
- ⊗ Usually
- ◯ Always

b. Please indicate the reasons that you enjoy taking care of immigrants and refugees (may choose more than one).

- ☐ Tropical and other conditions not frequently diagnosed in US-born patients
- ☒ Learning about other cultures
- ☐ They don't complain as much
- ☐ Being able to hear their stories
- ☐ Their care is more complicated
- ☐ Their care is less complicated
- ☒ They are very appreciative of your help.
- ☐ They are extremely vulnerable
- ☐ Other:

c. Taking care of immigrants and refugees is more challenging than taking care of US born patients.

- ◯ Never
- ◯ Rarely
- ⊗ Sometimes
- ◯ Usually
- ◯ Always

d. Please mark all the challenges that you face as a provider when providing care to immigrants and refugees (may choose more than one):

- ☒ Language barriers
- ☒ Insurance barriers
- ☒ Cultural barriers
- ☐ Finding a professional interpreter
- ☒ Knowing how to work with a professional interpreter
- ☐ Time constraints
- ☐ My own knowledge related to tropical and travel medicine
- ☐ Transportation problems for the patient
- ☐ Patients not understanding treatment plan
- ☐ Patients not following treatment plan
- ☐ My lack of knowledge regarding the patient's culture
- ☐ Bias or stereotyping
- ☐ Other:

e. Please mark all of the challenges faced by immigrant and refugee populations when receiving healthcare that you have perceived or witnessed (may choose more than one):

- ☐ Language barriers
- ☒ Insurance barriers
- ☒ Cultural barriers
- ☐ Finding a professional interpreter
- ☐ Knowing how to work with a professional interpreter
- ☐ Time constraints
- ☐ Insufficiently trained health care providers
- ☐ Transportation problems for the patient
- ☐ Food insufficiency
- ☐ Need for child care
- ☐ Patients not understanding treatment plan
- ☐ Patients not following treatment plan
- ☐ My lack of knowledge regarding the patient's culture
- ☐ Bias or stereotyping
- ☐ Trust issues
- ☐ Other...

f. Rank how well immigrants and refugees understand the healthcare that you are trying to provide.

- ○ Significantly less than a US born individual
- ○ Less than a US born individual
- ○ Equivalent to a US born individual
- ⊗ More than a US born patient
- ○ Significantly more than a US born individual

g. Immigrants and refugees adhere to treatment plans and follow my recommendations.

- ○ Never
- ○ Rarely
- ○ Sometimes
- ⊗ Usually
- ○ Always

h. Immigrants and refugees should receive the same care and insurance coverage as US born patients.

- ○ Never
- ○ Rarely
- ○ Sometimes
- ⊗ Usually
- ○ Always

i. Immigrants and refugees who are undocumented should receive the same care and insurance coverage as US born patients.

- ○ Never
- ○ Rarely
- ○ Sometimes
- ⊗ Usually
- ○ Always

j. Every physician is professionally obligated to care for immigrants and refugees if they present to your clinic or hospital.

- ○ Strongly disagree
- ○ Disagree
- ○ No opinion
- ⊗ Agree
- ○ Strongly agree

k. Is healthcare a human right?

⊗ Yes
◯ No

B. If you wish, please tell us about what you enjoy or do not enjoy about immigrant and refugee health care and the greatest challenges you face in caring for this population.

____

SECTION D: DEMOGRAPHIC INFORMATION
Please answer the following questions by checking the box in front of the response choice that best describes you.

a. Your age?

◯ 20 to 24
◯ 25 to 29
◯ 30 to 34
⊗ 35 to 39
◯ 40 or older

b. Your gender?

◯ Female
⊗ Male
◯ Other

c. Are you Hispanic or Latino?

◯ Yes
⊗ No

d. What is your race? (Select one or more responses)

◯ American Indian or Alaska Native
◯ Asian (Please specify):
◯ Black or African American
◯ Native Hawaiian or Other Pacific Islander
⊗ White
◯ Other (Please specify):

e. Were you born in the United States?

◯ Yes
⊗ No

f. If not, in what country were you born?

Iran

g. Your residency year?

◯ PGY1
⊗ PGY2
◯ PGY3
◯ PGY4
◯ PGY5

h. How would you classify your political ideology?

◯ Conservative
◯ Somewhat conservative
⊗ Moderate
◯ Somewhat liberal
◯ Liberal
◯ Other (Please specify):

i. Estimated level of educational debt?

⊗ None
◯ Less than $50,000
◯ $50,000 - $100,000
◯ $100,000 - $200,000
◯ $200,000 or more

j. Do you plan to subspecialize?

⊗ Yes
◯ No

k. Languages spoken?

☒ English
☐ Spanish
☒ French
☐ Hmong
☐ Somali
☐ Japanese
☐ Chinese
☐ Russian
☐ Ethiopian
☒ Other ___________

Farsi

l. Are you in the Global Health Pathway?

◯ Yes
⊗ No

m. Did you earn your degree in the US?

◯ Yes
⊗ No

n. What residency program are you in?

◯ Internal Medicine
◯ Med-Peds
◯ Pediatrics
◯ Family Practice
◯ Neurology
◯ Psychiatry
◯ ObGyn
◯ Neurosurgery
◯ General Surgery
◯ Orthopedic Surgery
◯ Urology
◯ Surgical sub-specialty (please specify in text box below)
⊗ Non-clinical specialty (radiology, pathology; please specify in text box below)

Radiology

Medical Trainees' attitudes, knowledge, and experience with immigrant and refugee health

Response was added on 12/16/2013 11:18am.

SECTION A: Personal experience with immigrant and refugee health care.

A. Please indicate your level of agreement with the following statements regarding your personal experience with immigrant and refugee health care by checking the box that best represents your experience.

a. During my inpatient rotations, I take care of the following percentage of immigrant and refugee patients:

- ◯ None
- ◯ 0 -5%
- ◯ 5-10%
- ⊗ 10-25%
- ◯ > 25%

b. During my outpatient rotations, I take care of the following percentage of immigrant and refugee patients:

- ◯ None
- ◯ 0-10%
- ◯ 10 -25%
- ◯ 25-50%
- ⊗ 50-75%
- ◯ >75%

c. I would like to take care of more immigrant and refugee patients.

- ◯ Strongly disagree
- ◯ Disagree
- ◯ No opinion
- ◯ Agree
- ⊗ Strongly agree

d. I plan to take care of immigrants and refugees when I finish residency.

- ◯ Strongly disagree
- ◯ Disagree
- ◯ No opinion
- ◯ Agree
- ⊗ Strongly agree

e. I plan to do short term (< 6 months) international work when I finish residency.

- ◯ Strongly disagree
- ◯ Disagree
- ◯ No opinion
- ◯ Agree
- ⊗ Strongly agree

f. I plan to do long term (>6 months) international work when I finish residency.

- ◯ Strongly disagree
- ◯ Disagree
- ⊗ No opinion
- ◯ Agree
- ◯ Strongly agree

g. I plan to work in health disparities in the following way after residency:

minority health problems in hematology (specifically, sickle cell disease and thalassemias)

SECTION B: MEDICAL EDUCATION

A. Please indicate your level of agreement with the following statements regarding your medical education and knowledge about immigrants and refugees by checking the box that best represents your opinion.

a. I have received specialized training in immigrant and refugee health, tropical medicine, or cross-cultural health.

◯ Strongly disagree
◯ Disagree
◯ No opinion
◯ Agree
⊗ Strongly agree

b. If you have received specialized training in immigrant and refugee health, tropical medicine, or cross-cultural health, please indicate all the contexts in which you received this training:

☐ As an undergraduate.
☐ As a medical student.
☒ As part of my residency.
☒ A special program.
☐ As part of my fellowship.
☐ As part of a degree program (e.g. MPH)
☐ Other:

c. I feel comfortable with my fund of knowledge regarding immigrant and refugee health.

◯ Strongly disagree
◯ Disagree
◯ No opinion
⊗ Agree
◯ Strongly agree

d. I would like to have further training in immigrant and refugee health.

◯ Strongly disagree
◯ Disagree
◯ No opinion
◯ Agree
⊗ Strongly agree

e. If you agree with the above, please indicate all the contexts in which you would like to receive this training:
□□

☒ As part of my residency.
☐ A special program.
☐ As part of my fellowship.

SECTION C: Attitudes towards immigrant health

A.□Please indicate your level of agreement with the following statements regarding immigrant and refugee health by checking the box that best represents your opinion.

a. I enjoy taking care of immigrants and refugees.

◯ Never
◯ Rarely
◯ Sometimes
◯ Usually
⊗ Always

b. Please indicate the reasons that you enjoy taking care of immigrants and refugees (may choose more than one).

☒ Tropical and other conditions not frequently diagnosed in US-born patients
☒ Learning about other cultures
☐ They don't complain as much
☒ Being able to hear their stories
☐ Their care is more complicated
☐ Their care is less complicated
☒ They are very appreciative of your help.
☐ They are extremely vulnerable
☐ Other:

c. Taking care of immigrants and refugees is more challenging than taking care of US born patients.

◯ Never
◯ Rarely
⊗ Sometimes
◯ Usually
◯ Always

d. Please mark all the challenges that you face as a provider when providing care to immigrants and refugees (may choose more than one):

- ☒ Language barriers
- ☒ Insurance barriers
- ☒ Cultural barriers
- ☒ Finding a professional interpreter
- ☒ Knowing how to work with a professional interpreter
- ☒ Time constraints
- ☒ My own knowledge related to tropical and travel medicine
- ☒ Transportation problems for the patient
- ☒ Patients not understanding treatment plan
- ☒ Patients not following treatment plan
- ☒ My lack of knowledge regarding the patient's culture
- ☐ Bias or stereotyping
- ☐ Other:

e. Please mark all of the challenges faced by immigrant and refugee populations when receiving healthcare that you have perceived or witnessed (may choose more than one):

- ☒ Language barriers
- ☒ Insurance barriers
- ☒ Cultural barriers
- ☒ Finding a professional interpreter
- ☒ Knowing how to work with a professional interpreter
- ☒ Time constraints
- ☒ Insufficiently trained health care providers
- ☒ Transportation problems for the patient
- ☒ Food insufficiency
- ☒ Need for child care
- ☒ Patients not understanding treatment plan
- ☒ Patients not following treatment plan
- ☒ My lack of knowledge regarding the patient's culture
- ☒ Bias or stereotyping
- ☒ Trust issues
- ☐ Other...

f. Rank how well immigrants and refugees understand the healthcare that you are trying to provide.

- ○ Significantly less than a US born individual
- ⊗ Less than a US born individual
- ○ Equivalent to a US born individual
- ○ More than a US born patient
- ○ Significantly more than a US born individual

g. Immigrants and refugees adhere to treatment plans and follow my recommendations.

- ○ Never
- ○ Rarely
- ⊗ Sometimes
- ○ Usually
- ○ Always

h. Immigrants and refugees should receive the same care and insurance coverage as US born patients.

- ○ Never
- ○ Rarely
- ○ Sometimes
- ○ Usually
- ⊗ Always

i. Immigrants and refugees who are undocumented should receive the same care and insurance coverage as US born patients.

- ○ Never
- ○ Rarely
- ○ Sometimes
- ○ Usually
- ⊗ Always

j. Every physician is professionally obligated to care for immigrants and refugees if they present to your clinic or hospital.

- ○ Strongly disagree
- ○ Disagree
- ○ No opinion
- ○ Agree
- ⊗ Strongly agree

k. Is healthcare a human right?
- ⊗ Yes
- ◯ No

B. If you wish, please tell us about what you enjoy or do not enjoy about immigrant and refugee health care and the greatest challenges you face in caring for this population.

as the child of immigrants, i am sometimes more comfortable taking care of refugees and immigrants than i am taking care of native Minnesotans - i can relate to my immigrant and refugee patients better, and i feel that they trust me more and do not judge me based on race.

SECTION D: DEMOGRAPHIC INFORMATION
Please answer the following questions by checking the box in front of the response choice that best describes you.

a. Your age?
- ◯ 20 to 24
- ◯ 25 to 29
- ⊗ 30 to 34
- ◯ 35 to 39
- ◯ 40 or older

b. Your gender?
- ⊗ Female
- ◯ Male
- ◯ Other

c. Are you Hispanic or Latino?
- ◯ Yes
- ⊗ No

d. What is your race? (Select one or more responses)
- ◯ American Indian or Alaska Native
- ⊗ Asian (Please specify):
- ◯ Black or African American
- ◯ Native Hawaiian or Other Pacific Islander
- ◯ White
- ◯ Other (Please specify):

e. Were you born in the United States?
- ⊗ Yes
- ◯ No

g. Your residency year?
- ⊗ PGY1
- ◯ PGY2
- ◯ PGY3
- ◯ PGY4
- ◯ PGY5

h. How would you classify your political ideology?
- ◯ Conservative
- ◯ Somewhat conservative
- ◯ Moderate
- ◯ Somewhat liberal
- ⊗ Liberal
- ◯ Other (Please specify):

i. Estimated level of educational debt?
- ◯ None
- ⊗ Less than $50,000
- ◯ $50,000 - $100,000
- ◯ $100,000 - $200,000
- ◯ $200,000 or more

j. Do you plan to subspecialize?
- ⊗ Yes
- ◯ No

k. Languages spoken?

☒ English
☐ Spanish
☒ French
☐ Hmong
☐ Somali
☐ Japanese
☐ Chinese
☐ Russian
☐ Ethiopian
☒ Other __________

Burmese

l. Are you in the Global Health Pathway?

⊗ Yes
○ No

m. Did you earn your degree in the US?

⊗ Yes
○ No

n. What residency program are you in?

○ Internal Medicine
⊗ Med-Peds
○ Pediatrics
○ Family Practice
○ Neurology
○ Psychiatry
○ ObGyn
○ Neurosurgery
○ General Surgery
○ Orthopedic Surgery
○ Urology
○ Surgical sub-specialty (please specify in text box below)
○ Non-clinical specialty (radiology, pathology; please specify in text box below)

Medical Trainees' attitudes, knowledge, and experience with immigrant and refugee health

Response was added on 12/17/2013 10:43am.

SECTION A: Personal experience with immigrant and refugee health care.

A. Please indicate your level of agreement with the following statements regarding your personal experience with immigrant and refugee health care by checking the box that best represents your experience.

a. During my inpatient rotations, I take care of the following percentage of immigrant and refugee patients:
- ◯ None
- ◯ 0 -5%
- ⊗ 5-10%
- ◯ 10-25%
- ◯ > 25%

b. During my outpatient rotations, I take care of the following percentage of immigrant and refugee patients:
- ◯ None
- ⊗ 0-10%
- ◯ 10 -25%
- ◯ 25-50%
- ◯ 50-75%
- ◯ >75%

c. I would like to take care of more immigrant and refugee patients.
- ◯ Strongly disagree
- ◯ Disagree
- ⊗ No opinion
- ◯ Agree
- ◯ Strongly agree

d. I plan to take care of immigrants and refugees when I finish residency.
- ◯ Strongly disagree
- ◯ Disagree
- ⊗ No opinion
- ◯ Agree
- ◯ Strongly agree

e. I plan to do short term (< 6 months) international work when I finish residency.
- ◯ Strongly disagree
- ◯ Disagree
- ◯ No opinion
- ◯ Agree
- ⊗ Strongly agree

f. I plan to do long term (>6 months) international work when I finish residency.
- ◯ Strongly disagree
- ⊗ Disagree
- ◯ No opinion
- ◯ Agree
- ◯ Strongly agree

g. I plan to work in health disparities in the following way after residency:
Through public health organizations

SECTION B: MEDICAL EDUCATION

A. Please indicate your level of agreement with the following statements regarding your medical education and knowledge about immigrants and refugees by checking the box that best represents your opinion.

a. I have received specialized training in immigrant and refugee health, tropical medicine, or cross-cultural health.

- ◯ Strongly disagree
- ◯ Disagree
- ◯ No opinion
- ⊗ Agree
- ◯ Strongly agree

b. If you have received specialized training in immigrant and refugee health, tropical medicine, or cross-cultural health, please indicate all the contexts in which you received this training:

- ☐ As an undergraduate.
- ☐ As a medical student.
- ☒ As part of my residency.
- ☐ A special program.
- ☐ As part of my fellowship.
- ☒ As part of a degree program (e.g. MPH)
- ☐ Other:

c. I feel comfortable with my fund of knowledge regarding immigrant and refugee health.

- ◯ Strongly disagree
- ⊗ Disagree
- ◯ No opinion
- ◯ Agree
- ◯ Strongly agree

d. I would like to have further training in immigrant and refugee health.

- ◯ Strongly disagree
- ◯ Disagree
- ◯ No opinion
- ⊗ Agree
- ◯ Strongly agree

e. If you agree with the above, please indicate all the contexts in which you would like to receive this training:

- ☒ As part of my residency.
- ☐ A special program.
- ☒ As part of my fellowship.

SECTION C: Attitudes towards immigrant health

A. Please indicate your level of agreement with the following statements regarding immigrant and refugee health by checking the box that best represents your opinion.

a. I enjoy taking care of immigrants and refugees.

- ◯ Never
- ◯ Rarely
- ◯ Sometimes
- ⊗ Usually
- ◯ Always

b. Please indicate the reasons that you enjoy taking care of immigrants and refugees (may choose more than one).

- ☒ Tropical and other conditions not frequently diagnosed in US-born patients
- ☐ Learning about other cultures
- ☐ They don't complain as much
- ☒ Being able to hear their stories
- ☐ Their care is more complicated
- ☐ Their care is less complicated
- ☒ They are very appreciative of your help.
- ☐ They are extremely vulnerable
- ☐ Other:

c. Taking care of immigrants and refugees is more challenging than taking care of US born patients.

- ◯ Never
- ◯ Rarely
- ◯ Sometimes
- ⊗ Usually
- ◯ Always

d. Please mark all the challenges that you face as a provider when providing care to immigrants and refugees (may choose more than one):

- ☒ Language barriers
- ☐ Insurance barriers
- ☒ Cultural barriers
- ☒ Finding a professional interpreter
- ☐ Knowing how to work with a professional interpreter
- ☐ Time constraints
- ☒ My own knowledge related to tropical and travel medicine
- ☐ Transportation problems for the patient
- ☐ Patients not understanding treatment plan
- ☐ Patients not following treatment plan
- ☒ My lack of knowledge regarding the patient's culture
- ☐ Bias or stereotyping
- ☐ Other:

e. Please mark all of the challenges faced by immigrant and refugee populations when receiving healthcare that you have perceived or witnessed (may choose more than one):

- ☒ Language barriers
- ☐ Insurance barriers
- ☒ Cultural barriers
- ☒ Finding a professional interpreter
- ☒ Knowing how to work with a professional interpreter
- ☒ Time constraints
- ☒ Insufficiently trained health care providers
- ☒ Transportation problems for the patient
- ☐ Food insufficiency
- ☐ Need for child care
- ☒ Patients not understanding treatment plan
- ☒ Patients not following treatment plan
- ☒ My lack of knowledge regarding the patient's culture
- ☒ Bias or stereotyping
- ☒ Trust issues
- ☐ Other...

f. Rank how well immigrants and refugees understand the healthcare that you are trying to provide.

- ○ Significantly less than a US born individual
- ⊗ Less than a US born individual
- ○ Equivalent to a US born individual
- ○ More than a US born patient
- ○ Significantly more than a US born individual

g. Immigrants and refugees adhere to treatment plans and follow my recommendations.

- ○ Never
- ○ Rarely
- ○ Sometimes
- ⊗ Usually
- ○ Always

h. Immigrants and refugees should receive the same care and insurance coverage as US born patients.

- ○ Never
- ○ Rarely
- ○ Sometimes
- ○ Usually
- ⊗ Always

i. Immigrants and refugees who are undocumented should receive the same care and insurance coverage as US born patients.

- ○ Never
- ○ Rarely
- ⊗ Sometimes
- ○ Usually
- ○ Always

j. Every physician is professionally obligated to care for immigrants and refugees if they present to your clinic or hospital.

- ○ Strongly disagree
- ○ Disagree
- ○ No opinion
- ○ Agree
- ⊗ Strongly agree

k. Is healthcare a human right?
- ⊗ Yes
- ◯ No

B. If you wish, please tell us about what you enjoy or do not enjoy about immigrant and refugee health care and the greatest challenges you face in caring for this population. ____

SECTION D: DEMOGRAPHIC INFORMATION
Please answer the following questions by checking the box in front of the response choice that best describes you.

a. Your age?
- ◯ 20 to 24
- ◯ 25 to 29
- ⊗ 30 to 34
- ◯ 35 to 39
- ◯ 40 or older

b. Your gender?
- ⊗ Female
- ◯ Male
- ◯ Other

c. Are you Hispanic or Latino?
- ◯ Yes
- ◯ No

d. What is your race? (Select one or more responses)
- ◯ American Indian or Alaska Native
- ◯ Asian (Please specify):
- ◯ Black or African American
- ◯ Native Hawaiian or Other Pacific Islander
- ⊗ White
- ◯ Other (Please specify):

e. Were you born in the United States?
- ⊗ Yes
- ◯ No

g. Your residency year?
- ◯ PGY1
- ◯ PGY2
- ⊗ PGY3
- ◯ PGY4
- ◯ PGY5

h. How would you classify your political ideology?
- ◯ Conservative
- ◯ Somewhat conservative
- ◯ Moderate
- ◯ Somewhat liberal
- ⊗ Liberal
- ◯ Other (Please specify):

i. Estimated level of educational debt?
- ◯ None
- ◯ Less than $50,000
- ◯ $50,000 - $100,000
- ◯ $100,000 - $200,000
- ⊗ $200,000 or more

j. Do you plan to subspecialize?
- ⊗ Yes
- ◯ No

k. Languages spoken?

- ☒ English
- ☐ Spanish
- ☒ French
- ☐ Hmong
- ☐ Somali
- ☐ Japanese
- ☐ Chinese
- ☐ Russian
- ☐ Ethiopian
- ☐ Other ___________

l. Are you in the Global Health Pathway?

- ◯ Yes
- ⊗ No

m. Did you earn your degree in the US?

- ⊗ Yes
- ◯ No

n. What residency program are you in?

- ◯ Internal Medicine
- ◯ Med-Peds
- ⊗ Pediatrics
- ◯ Family Practice
- ◯ Neurology
- ◯ Psychiatry
- ◯ ObGyn
- ◯ Neurosurgery
- ◯ General Surgery
- ◯ Orthopedic Surgery
- ◯ Urology
- ◯ Surgical sub-specialty (please specify in text box below)
- ◯ Non-clinical specialty (radiology, pathology; please specify in text box below)

Medical Trainees' attitudes, knowledge, and experience with immigrant and refugee health

Response was added on 12/17/2013 10:07pm.

SECTION A: Personal experience with immigrant and refugee health care.

A. Please indicate your level of agreement with the following statements regarding your personal experience with immigrant and refugee health care by checking the box that best represents your experience.

a. During my inpatient rotations, I take care of the following percentage of immigrant and refugee patients:

- ◯ None
- ◯ 0 -5%
- ⊗ 5-10%
- ◯ 10-25%
- ◯ > 25%

b. During my outpatient rotations, I take care of the following percentage of immigrant and refugee patients:

- ◯ None
- ⊗ 0-10%
- ◯ 10 -25%
- ◯ 25-50%
- ◯ 50-75%
- ◯ >75%

c. I would like to take care of more immigrant and refugee patients.

- ◯ Strongly disagree
- ⊗ Disagree
- ◯ No opinion
- ◯ Agree
- ◯ Strongly agree

d. I plan to take care of immigrants and refugees when I finish residency.

- ◯ Strongly disagree
- ⊗ Disagree
- ◯ No opinion
- ◯ Agree
- ◯ Strongly agree

e. I plan to do short term (< 6 months) international work when I finish residency.

- ◯ Strongly disagree
- ⊗ Disagree
- ◯ No opinion
- ◯ Agree
- ◯ Strongly agree

f. I plan to do long term (>6 months) international work when I finish residency.

- ◯ Strongly disagree
- ⊗ Disagree
- ◯ No opinion
- ◯ Agree
- ◯ Strongly agree

g. I plan to work in health disparities in the following way after residency:

No set plans. Likely work in outer Twin Citis suburb.

SECTION B: MEDICAL EDUCATION

A. Please indicate your level of agreement with the following statements regarding your medical education and knowledge about immigrants and refugees by checking the box that best represents your opinion.

a. I have received specialized training in immigrant and refugee health, tropical medicine, or cross-cultural health.

- ◯ Strongly disagree
- ⊗ Disagree
- ◯ No opinion
- ◯ Agree
- ◯ Strongly agree

c. I feel comfortable with my fund of knowledge regarding immigrant and refugee health.

- ⊗ Strongly disagree
- ◯ Disagree
- ◯ No opinion
- ◯ Agree
- ◯ Strongly agree

d. I would like to have further training in immigrant and refugee health.

- ◯ Strongly disagree
- ◯ Disagree
- ⊗ No opinion
- ◯ Agree
- ◯ Strongly agree

SECTION C: Attitudes towards immigrant health

A.Please indicate your level of agreement with the following statements regarding immigrant and refugee health by checking the box that best represents your opinion.

a. I enjoy taking care of immigrants and refugees.

- ◯ Never
- ◯ Rarely
- ◯ Sometimes
- ⊗ Usually
- ◯ Always

b. Please indicate the reasons that you enjoy taking care of immigrants and refugees (may choose more than one).

- ☐ Tropical and other conditions not frequently diagnosed in US-born patients
- ☒ Learning about other cultures
- ☐ They don't complain as much
- ☐ Being able to hear their stories
- ☐ Their care is more complicated
- ☐ Their care is less complicated
- ☐ They are very appreciative of your help.
- ☒ They are extremely vulnerable
- ☐ Other:

c. Taking care of immigrants and refugees is more challenging than taking care of US born patients.

- ◯ Never
- ◯ Rarely
- ⊗ Sometimes
- ◯ Usually
- ◯ Always

d. Please mark all the challenges that you face as a provider when providing care to immigrants and refugees (may choose more than one):

- ☒ Language barriers
- ☒ Insurance barriers
- ☒ Cultural barriers
- ☒ Finding a professional interpreter
- ☐ Knowing how to work with a professional interpreter
- ☐ Time constraints
- ☒ My own knowledge related to tropical and travel medicine
- ☐ Transportation problems for the patient
- ☒ Patients not understanding treatment plan
- ☒ Patients not following treatment plan
- ☒ My lack of knowledge regarding the patient's culture
- ☒ Bias or stereotyping
- ☐ Other:

e. Please mark all of the challenges faced by immigrant and refugee populations when receiving healthcare that you have perceived or witnessed (may choose more than one):

- ☒ Language barriers
- ☒ Insurance barriers
- ☒ Cultural barriers
- ☒ Finding a professional interpreter
- ☒ Knowing how to work with a professional interpreter
- ☐ Time constraints
- ☒ Insufficiently trained health care providers
- ☒ Transportation problems for the patient
- ☐ Food insufficiency
- ☐ Need for child care
- ☒ Patients not understanding treatment plan
- ☐ Patients not following treatment plan
- ☒ My lack of knowledge regarding the patient's culture
- ☒ Bias or stereotyping
- ☒ Trust issues
- ☐ Other...

f. Rank how well immigrants and refugees understand the healthcare that you are trying to provide.

- ◯ Significantly less than a US born individual
- ⊗ Less than a US born individual
- ◯ Equivalent to a US born individual
- ◯ More than a US born patient
- ◯ Significantly more than a US born individual

g. Immigrants and refugees adhere to treatment plans and follow my recommendations.

- ◯ Never
- ◯ Rarely
- ⊗ Sometimes
- ◯ Usually
- ◯ Always

h. Immigrants and refugees should receive the same care and insurance coverage as US born patients.

- ◯ Never
- ◯ Rarely
- ◯ Sometimes
- ⊗ Usually
- ◯ Always

i. Immigrants and refugees who are undocumented should receive the same care and insurance coverage as US born patients.

- ◯ Never
- ◯ Rarely
- ◯ Sometimes
- ⊗ Usually
- ◯ Always

j. Every physician is professionally obligated to care for immigrants and refugees if they present to your clinic or hospital.

- ◯ Strongly disagree
- ◯ Disagree
- ◯ No opinion
- ⊗ Agree
- ◯ Strongly agree

k. Is healthcare a human right?

- ⊗ Yes
- ◯ No

B. If you wish, please tell us about what you enjoy or do not enjoy about immigrant and refugee health care and the greatest challenges you face in caring for this population.

____

SECTION D: DEMOGRAPHIC INFORMATION
Please answer the following questions by checking the box in front of the response choice that best describes you.

a. Your age?	◯ 20 to 24<br>◯ 25 to 29<br>⊗ 30 to 34<br>◯ 35 to 39<br>◯ 40 or older
b. Your gender?	⊗ Female<br>◯ Male<br>◯ Other
c. Are you Hispanic or Latino?	◯ Yes<br>⊗ No
d. What is your race? (Select one or more responses)	◯ American Indian or Alaska Native<br>◯ Asian (Please specify):<br>◯ Black or African American<br>◯ Native Hawaiian or Other Pacific Islander<br>⊗ White<br>◯ Other (Please specify):
e. Were you born in the United States?	⊗ Yes<br>◯ No
g. Your residency year?	◯ PGY1<br>⊗ PGY2<br>◯ PGY3<br>◯ PGY4<br>◯ PGY5
h. How would you classify your political ideology?	◯ Conservative<br>◯ Somewhat conservative<br>⊗ Moderate<br>◯ Somewhat liberal<br>◯ Liberal<br>◯ Other (Please specify):
i. Estimated level of educational debt?	◯ None<br>◯ Less than $50,000<br>◯ $50,000 - $100,000<br>⊗ $100,000 - $200,000<br>◯ $200,000 or more
j. Do you plan to subspecialize?	◯ Yes<br>⊗ No
k. Languages spoken?	☒ English<br>☐ Spanish<br>☐ French<br>☐ Hmong<br>☐ Somali<br>☐ Japanese<br>☐ Chinese<br>☐ Russian<br>☐ Ethiopian<br>☐ Other ___________
l. Are you in the Global Health Pathway?	◯ Yes<br>⊗ No

m. Did you earn your degree in the US?

⊗ Yes
◯ No

n. What residency program are you in?

◯ Internal Medicine
◯ Med-Peds
⊗ Pediatrics
◯ Family Practice
◯ Neurology
◯ Psychiatry
◯ ObGyn
◯ Neurosurgery
◯ General Surgery
◯ Orthopedic Surgery
◯ Urology
◯ Surgical sub-specialty (please specify in text box below)
◯ Non-clinical specialty (radiology, pathology; please specify in text box below)

Medical Trainees' attitudes, knowledge, and experience with immigrant and refugee health

Response was added on 12/18/2013 10:01am.

SECTION A: Personal experience with immigrant and refugee health care.

A. Please indicate your level of agreement with the following statements regarding your personal experience with immigrant and refugee health care by checking the box that best represents your experience.

a. During my inpatient rotations, I take care of the following percentage of immigrant and refugee patients:

- ◯ None
- ◯ 0 -5%
- ◯ 5-10%
- ⊗ 10-25%
- ◯ > 25%

b. During my outpatient rotations, I take care of the following percentage of immigrant and refugee patients:

- ◯ None
- ◯ 0-10%
- ◯ 10 -25%
- ⊗ 25-50%
- ◯ 50-75%
- ◯ >75%

c. I would like to take care of more immigrant and refugee patients.

- ◯ Strongly disagree
- ⊗ Disagree
- ◯ No opinion
- ◯ Agree
- ◯ Strongly agree

d. I plan to take care of immigrants and refugees when I finish residency.

- ◯ Strongly disagree
- ◯ Disagree
- ◯ No opinion
- ⊗ Agree
- ◯ Strongly agree

e. I plan to do short term (< 6 months) international work when I finish residency.

- ◯ Strongly disagree
- ◯ Disagree
- ◯ No opinion
- ◯ Agree
- ⊗ Strongly agree

f. I plan to do long term (>6 months) international work when I finish residency.

- ◯ Strongly disagree
- ◯ Disagree
- ◯ No opinion
- ⊗ Agree
- ◯ Strongly agree

g. I plan to work in health disparities in the following way after residency:

I have done a lot of work in West Africa and hope to return to the region for short-term work initially and then long-term in my later years.

SECTION B: MEDICAL EDUCATION

A. Please indicate your level of agreement with the following statements regarding your medical education and knowledge about immigrants and refugees by checking the box that best represents your opinion.

a. I have received specialized training in immigrant and refugee health, tropical medicine, or cross-cultural health.

◯ Strongly disagree
◯ Disagree
◯ No opinion
⊗ Agree
◯ Strongly agree

b. If you have received specialized training in immigrant and refugee health, tropical medicine, or cross-cultural health, please indicate all the contexts in which you received this training:

☒ As an undergraduate.
☒ As a medical student.
☒ As part of my residency.
☐ A special program.
☐ As part of my fellowship.
☒ As part of a degree program (e.g. MPH)
☐ Other:

c. I feel comfortable with my fund of knowledge regarding immigrant and refugee health.

◯ Strongly disagree
◯ Disagree
◯ No opinion
⊗ Agree
◯ Strongly agree

d. I would like to have further training in immigrant and refugee health.

◯ Strongly disagree
◯ Disagree
⊗ No opinion
◯ Agree
◯ Strongly agree

SECTION C: Attitudes towards immigrant health

A.Please indicate your level of agreement with the following statements regarding immigrant and refugee health by checking the box that best represents your opinion.

a. I enjoy taking care of immigrants and refugees.

◯ Never
◯ Rarely
◯ Sometimes
⊗ Usually
◯ Always

b. Please indicate the reasons that you enjoy taking care of immigrants and refugees (may choose more than one).

☒ Tropical and other conditions not frequently diagnosed in US-born patients
☒ Learning about other cultures
☐ They don't complain as much
☐ Being able to hear their stories
☐ Their care is more complicated
☐ Their care is less complicated
☐ They are very appreciative of your help.
☒ They are extremely vulnerable
☐ Other:

c. Taking care of immigrants and refugees is more challenging than taking care of US born patients.

◯ Never
◯ Rarely
◯ Sometimes
⊗ Usually
◯ Always

d. Please mark all the challenges that you face as a provider when providing care to immigrants and refugees (may choose more than one):

- ☒ Language barriers
- ☒ Insurance barriers
- ☒ Cultural barriers
- ☐ Finding a professional interpreter
- ☐ Knowing how to work with a professional interpreter
- ☒ Time constraints
- ☐ My own knowledge related to tropical and travel medicine
- ☒ Transportation problems for the patient
- ☒ Patients not understanding treatment plan
- ☒ Patients not following treatment plan
- ☐ My lack of knowledge regarding the patient's culture
- ☐ Bias or stereotyping
- ☐ Other:

e. Please mark all of the challenges faced by immigrant and refugee populations when receiving healthcare that you have perceived or witnessed (may choose more than one):

- ☒ Language barriers
- ☒ Insurance barriers
- ☒ Cultural barriers
- ☐ Finding a professional interpreter
- ☐ Knowing how to work with a professional interpreter
- ☐ Time constraints
- ☐ Insufficiently trained health care providers
- ☒ Transportation problems for the patient
- ☐ Food insufficiency
- ☐ Need for child care
- ☒ Patients not understanding treatment plan
- ☒ Patients not following treatment plan
- ☐ My lack of knowledge regarding the patient's culture
- ☐ Bias or stereotyping
- ☐ Trust issues
- ☐ Other...

f. Rank how well immigrants and refugees understand the healthcare that you are trying to provide.

- ○ Significantly less than a US born individual
- ⊗ Less than a US born individual
- ○ Equivalent to a US born individual
- ○ More than a US born patient
- ○ Significantly more than a US born individual

g. Immigrants and refugees adhere to treatment plans and follow my recommendations.

- ○ Never
- ○ Rarely
- ⊗ Sometimes
- ○ Usually
- ○ Always

h. Immigrants and refugees should receive the same care and insurance coverage as US born patients.

- ○ Never
- ○ Rarely
- ⊗ Sometimes
- ○ Usually
- ○ Always

i. Immigrants and refugees who are undocumented should receive the same care and insurance coverage as US born patients.

- ○ Never
- ○ Rarely
- ○ Sometimes
- ⊗ Usually
- ○ Always

j. Every physician is professionally obligated to care for immigrants and refugees if they present to your clinic or hospital.

- ○ Strongly disagree
- ○ Disagree
- ○ No opinion
- ⊗ Agree
- ○ Strongly agree

k. Is healthcare a human right?	◯ Yes<br>⊗ No
B. If you wish, please tell us about what you enjoy or do not enjoy about immigrant and refugee health care and the greatest challenges you face in caring for this population.	__________________________________

SECTION D: DEMOGRAPHIC INFORMATION
Please answer the following questions by checking the box in front of the response choice that best describes you.

a. Your age?	◯ 20 to 24<br>◯ 25 to 29<br>⊗ 30 to 34<br>◯ 35 to 39<br>◯ 40 or older
b. Your gender?	⊗ Female<br>◯ Male<br>◯ Other
c. Are you Hispanic or Latino?	◯ Yes<br>⊗ No
d. What is your race? (Select one or more responses)	◯ American Indian or Alaska Native<br>◯ Asian (Please specify):<br>◯ Black or African American<br>◯ Native Hawaiian or Other Pacific Islander<br>⊗ White<br>◯ Other (Please specify):
e. Were you born in the United States?	⊗ Yes<br>◯ No
g. Your residency year?	⊗ PGY1<br>◯ PGY2<br>◯ PGY3<br>◯ PGY4<br>◯ PGY5
h. How would you classify your political ideology?	◯ Conservative<br>◯ Somewhat conservative<br>⊗ Moderate<br>◯ Somewhat liberal<br>◯ Liberal<br>◯ Other (Please specify):
i. Estimated level of educational debt?	◯ None<br>◯ Less than $50,000<br>◯ $50,000 - $100,000<br>◯ $100,000 - $200,000<br>⊗ $200,000 or more
j. Do you plan to subspecialize?	⊗ Yes<br>◯ No

k. Languages spoken?

- ☒ English
- ☐ Spanish
- ☒ French
- ☐ Hmong
- ☐ Somali
- ☐ Japanese
- ☐ Chinese
- ☐ Russian
- ☐ Ethiopian
- ☐ Other ___________

l. Are you in the Global Health Pathway?

- ⊗ Yes
- ○ No

m.[illegible] Did you earn your degree in the US?

- ⊗ Yes
- ○ No

n. What residency program are you in?

- ○ Internal Medicine
- ○ Med-Peds
- ⊗ Pediatrics
- ○ Family Practice
- ○ Neurology
- ○ Psychiatry
- ○ ObGyn
- ○ Neurosurgery
- ○ General Surgery
- ○ Orthopedic Surgery
- ○ Urology
- ○ Surgical sub-specialty (please specify in text box below)
- ○ Non-clinical specialty (radiology, pathology; please specify in text box below)

Medical Trainees' attitudes, knowledge, and experience with immigrant and refugee health

Response was added on 12/18/2013 8:21pm.

SECTION A: Personal experience with immigrant and refugee health care.

A. Please indicate your level of agreement with the following statements regarding your personal experience with immigrant and refugee health care by checking the box that best represents your experience.

a. During my inpatient rotations, I take care of the following percentage of immigrant and refugee patients:

- ○ None
- ○ 0 -5%
- ⊗ 5-10%
- ○ 10-25%
- ○ > 25%

b. During my outpatient rotations, I take care of the following percentage of immigrant and refugee patients:

- ○ None
- ⊗ 0-10%
- ○ 10 -25%
- ○ 25-50%
- ○ 50-75%
- ○ >75%

c. I would like to take care of more immigrant and refugee patients.

- ○ Strongly disagree
- ○ Disagree
- ○ No opinion
- ⊗ Agree
- ○ Strongly agree

d. I plan to take care of immigrants and refugees when I finish residency.

- ○ Strongly disagree
- ○ Disagree
- ○ No opinion
- ⊗ Agree
- ○ Strongly agree

e. I plan to do short term (< 6 months) international work when I finish residency.

- ○ Strongly disagree
- ○ Disagree
- ○ No opinion
- ⊗ Agree
- ○ Strongly agree

f. I plan to do long term (>6 months) international work when I finish residency.

- ○ Strongly disagree
- ⊗ Disagree
- ○ No opinion
- ○ Agree
- ○ Strongly agree

g. I plan to work in health disparities in the following way after residency:

Continue actively seeking to care for patients with healtcare discrepancies.

SECTION B: MEDICAL EDUCATION

A. Please indicate your level of agreement with the following statements regarding your medical education and knowledge about immigrants and refugees by checking the box that best represents your opinion.

a. I have received specialized training in immigrant and refugee health, tropical medicine, or cross-cultural health.

◯ Strongly disagree
◯ Disagree
◯ No opinion
◯ Agree
⊗ Strongly agree

b. If you have received specialized training in immigrant and refugee health, tropical medicine, or cross-cultural health, please indicate all the contexts in which you received this training:

☒ As an undergraduate.
☒ As a medical student.
☒ As part of my residency.
☒ A special program.
☐ As part of my fellowship.
☐ As part of a degree program (e.g. MPH)
☐ Other:

c. I feel comfortable with my fund of knowledge regarding immigrant and refugee health.

◯ Strongly disagree
◯ Disagree
◯ No opinion
⊗ Agree
◯ Strongly agree

d. I would like to have further training in immigrant and refugee health.

◯ Strongly disagree
◯ Disagree
◯ No opinion
◯ Agree
⊗ Strongly agree

e. If you agree with the above, please indicate all the contexts in which you would like to receive this training:

☒ As part of my residency.
☒ A special program.
☐ As part of my fellowship.

SECTION C: Attitudes towards immigrant health

A. Please indicate your level of agreement with the following statements regarding immigrant and refugee health by checking the box that best represents your opinion.

a. I enjoy taking care of immigrants and refugees.

◯ Never
◯ Rarely
◯ Sometimes
◯ Usually
⊗ Always

b. Please indicate the reasons that you enjoy taking care of immigrants and refugees (may choose more than one).

☒ Tropical and other conditions not frequently diagnosed in US-born patients
☒ Learning about other cultures
☐ They don't complain as much
☒ Being able to hear their stories
☐ Their care is more complicated
☐ Their care is less complicated
☐ They are very appreciative of your help.
☒ They are extremely vulnerable
☐ Other:

c. Taking care of immigrants and refugees is more challenging than taking care of US born patients.

◯ Never
◯ Rarely
◯ Sometimes
⊗ Usually
◯ Always

d. Please mark all the challenges that you face as a provider when providing care to immigrants and refugees (may choose more than one):

- ☒ Language barriers
- ☐ Insurance barriers
- ☒ Cultural barriers
- ☐ Finding a professional interpreter
- ☐ Knowing how to work with a professional interpreter
- ☐ Time constraints
- ☒ My own knowledge related to tropical and travel medicine
- ☒ Transportation problems for the patient
- ☐ Patients not understanding treatment plan
- ☐ Patients not following treatment plan
- ☐ My lack of knowledge regarding the patient's culture
- ☐ Bias or stereotyping
- ☐ Other:

e. Please mark all of the challenges faced by immigrant and refugee populations when receiving healthcare that you have perceived or witnessed (may choose more than one):

- ☒ Language barriers
- ☒ Insurance barriers
- ☒ Cultural barriers
- ☒ Finding a professional interpreter
- ☒ Knowing how to work with a professional interpreter
- ☒ Time constraints
- ☒ Insufficiently trained health care providers
- ☒ Transportation problems for the patient
- ☐ Food insufficiency
- ☐ Need for child care
- ☒ Patients not understanding treatment plan
- ☐ Patients not following treatment plan
- ☐ My lack of knowledge regarding the patient's culture
- ☒ Bias or stereotyping
- ☒ Trust issues
- ☐ Other...

f. Rank how well immigrants and refugees understand the healthcare that you are trying to provide.

- ○ Significantly less than a US born individual
- ⊗ Less than a US born individual
- ○ Equivalent to a US born individual
- ○ More than a US born patient
- ○ Significantly more than a US born individual

g. Immigrants and refugees adhere to treatment plans and follow my recommendations.

- ○ Never
- ○ Rarely
- ○ Sometimes
- ⊗ Usually
- ○ Always

h. Immigrants and refugees should receive the same care and insurance coverage as US born patients.

- ○ Never
- ○ Rarely
- ○ Sometimes
- ○ Usually
- ⊗ Always

i. Immigrants and refugees who are undocumented should receive the same care and insurance coverage as US born patients.

- ○ Never
- ○ Rarely
- ⊗ Sometimes
- ○ Usually
- ○ Always

j. Every physician is professionally obligated to care for immigrants and refugees if they present to your clinic or hospital.

- ○ Strongly disagree
- ○ Disagree
- ○ No opinion
- ○ Agree
- ⊗ Strongly agree

k. Is healthcare a human right?
- ⊗ Yes
- ◯ No

B. If you wish, please tell us about what you enjoy or do not enjoy about immigrant and refugee health care and the greatest challenges you face in caring for this population. ____

SECTION D: DEMOGRAPHIC INFORMATION
Please answer the following questions by checking the box in front of the response choice that best describes you.

a. Your age?
- ◯ 20 to 24
- ⊗ 25 to 29
- ◯ 30 to 34
- ◯ 35 to 39
- ◯ 40 or older

b. Your gender?
- ⊗ Female
- ◯ Male
- ◯ Other

c.Are you Hispanic or Latino?
- ◯ Yes
- ⊗ No

d. What is your race? (Select one or more responses)
- ◯ American Indian or Alaska Native
- ◯ Asian (Please specify):
- ◯ Black or African American
- ◯ Native Hawaiian or Other Pacific Islander
- ⊗ White
- ◯ Other (Please specify):

e.Were you born in the United States?
- ⊗ Yes
- ◯ No

g. Your residency year?
- ◯ PGY1
- ⊗ PGY2
- ◯ PGY3
- ◯ PGY4
- ◯ PGY5

h. How would you classify your political ideology?
- ◯ Conservative
- ◯ Somewhat conservative
- ⊗ Moderate
- ◯ Somewhat liberal
- ◯ Liberal
- ◯ Other (Please specify):

i. Estimated level of educational debt?
- ◯ None
- ◯ Less than $50,000
- ◯ $50,000 - $100,000
- ◯ $100,000 - $200,000
- ⊗ $200,000 or more

j.Do you plan to subspecialize?
- ⊗ Yes
- ◯ No

k. Languages spoken?

- ☒ English
- ☒ Spanish
- ☐ French
- ☐ Hmong
- ☐ Somali
- ☐ Japanese
- ☐ Chinese
- ☐ Russian
- ☐ Ethiopian
- ☐ Other ___________

l. Are you in the Global Health Pathway?

- ⊗ Yes
- ◯ No

m. Did you earn your degree in the US?

- ⊗ Yes
- ◯ No

n. What residency program are you in?

- ⊗ Internal Medicine
- ◯ Med-Peds
- ◯ Pediatrics
- ◯ Family Practice
- ◯ Neurology
- ◯ Psychiatry
- ◯ ObGyn
- ◯ Neurosurgery
- ◯ General Surgery
- ◯ Orthopedic Surgery
- ◯ Urology
- ◯ Surgical sub-specialty (please specify in text box below)
- ◯ Non-clinical specialty (radiology, pathology; please specify in text box below)

Medical Trainees' attitudes, knowledge, and experience with immigrant and refugee health

Response was added on 12/19/2013 11:31am.

SECTION A: Personal experience with immigrant and refugee health care.

A. Please indicate your level of agreement with the following statements regarding your personal experience with immigrant and refugee health care by checking the box that best represents your experience.

a. During my inpatient rotations, I take care of the following percentage of immigrant and refugee patients:	◯ None<br>◯ 0 -5%<br>⊗ 5-10%<br>⊗ 10-25%<br>◯ > 25%
b. During my outpatient rotations, I take care of the following percentage of immigrant and refugee patients:	◯ None<br>◯ 0-10%<br>◯ 10 -25%<br>⊗ 25-50%<br>◯ 50-75%<br>◯ >75%
c. I would like to take care of more immigrant and refugee patients.	◯ Strongly disagree<br>◯ Disagree<br>⊗ No opinion<br>◯ Agree<br>◯ Strongly agree
d. I plan to take care of immigrants and refugees when I finish residency.	◯ Strongly disagree<br>◯ Disagree<br>⊗ No opinion<br>◯ Agree<br>◯ Strongly agree
e. I plan to do short term (< 6 months) international work when I finish residency.	◯ Strongly disagree<br>◯ Disagree<br>◯ No opinion<br>⊗ Agree<br>◯ Strongly agree
f. I plan to do long term (>6 months) international work when I finish residency.	◯ Strongly disagree<br>◯ Disagree<br>◯ No opinion<br>⊗ Agree<br>◯ Strongly agree
g. I plan to work in health disparities in the following way after residency:	?

SECTION B: MEDICAL EDUCATION

A. Please indicate your level of agreement with the following statements regarding your medical education and knowledge about immigrants and refugees by checking the box that best represents your opinion.

a. I have received specialized training in immigrant and refugee health, tropical medicine, or cross-cultural health.

- ◯ Strongly disagree
- ◯ Disagree
- ◯ No opinion
- ⊗ Agree
- ◯ Strongly agree

b. If you have received specialized training in immigrant and refugee health, tropical medicine, or cross-cultural health, please indicate all the contexts in which you received this training:

- ☐ As an undergraduate.
- ☒ As a medical student.
- ☒ As part of my residency.
- ☐ A special program.
- ☐ As part of my fellowship.
- ☐ As part of a degree program (e.g. MPH)
- ☐ Other:

c. I feel comfortable with my fund of knowledge regarding immigrant and refugee health.

- ◯ Strongly disagree
- ◯ Disagree
- ◯ No opinion
- ⊗ Agree
- ◯ Strongly agree

d. I would like to have further training in immigrant and refugee health.

- ◯ Strongly disagree
- ◯ Disagree
- ⊗ No opinion
- ◯ Agree
- ◯ Strongly agree

SECTION C: Attitudes towards immigrant health

A.Please indicate your level of agreement with the following statements regarding immigrant and refugee health by checking the box that best represents your opinion.

a. I enjoy taking care of immigrants and refugees.

- ◯ Never
- ◯ Rarely
- ⊗ Sometimes
- ◯ Usually
- ◯ Always

b. Please indicate the reasons that you enjoy taking care of immigrants and refugees (may choose more than one).

- ☒ Tropical and other conditions not frequently diagnosed in US-born patients
- ☒ Learning about other cultures
- ☐ They don't complain as much
- ☒ Being able to hear their stories
- ☒ Their care is more complicated
- ☐ Their care is less complicated
- ☐ They are very appreciative of your help.
- ☐ They are extremely vulnerable
- ☐ Other:

c. Taking care of immigrants and refugees is more challenging than taking care of US born patients.

- ◯ Never
- ◯ Rarely
- ⊗ Sometimes
- ◯ Usually
- ◯ Always

d. Please mark all the challenges that you face as a provider when providing care to immigrants and refugees (may choose more than one):

- [x] Language barriers
- [x] Insurance barriers
- [x] Cultural barriers
- [x] Finding a professional interpreter
- [ ] Knowing how to work with a professional interpreter
- [x] Time constraints
- [x] My own knowledge related to tropical and travel medicine
- [x] Transportation problems for the patient
- [x] Patients not understanding treatment plan
- [x] Patients not following treatment plan
- [ ] My lack of knowledge regarding the patient's culture
- [ ] Bias or stereotyping
- [ ] Other:

e. Please mark all of the challenges faced by immigrant and refugee populations when receiving healthcare that you have perceived or witnessed (may choose more than one):

- [x] Language barriers
- [x] Insurance barriers
- [x] Cultural barriers
- [ ] Finding a professional interpreter
- [x] Knowing how to work with a professional interpreter
- [x] Time constraints
- [ ] Insufficiently trained health care providers
- [x] Transportation problems for the patient
- [x] Food insufficiency
- [x] Need for child care
- [x] Patients not understanding treatment plan
- [x] Patients not following treatment plan
- [ ] My lack of knowledge regarding the patient's culture
- [x] Bias or stereotyping
- [x] Trust issues
- [ ] Other...

f. Rank how well immigrants and refugees understand the healthcare that you are trying to provide.

- ◯ Significantly less than a US born individual
- ⊗ Less than a US born individual
- ◯ Equivalent to a US born individual
- ◯ More than a US born patient
- ◯ Significantly more than a US born individual

g. Immigrants and refugees adhere to treatment plans and follow my recommendations.

- ◯ Never
- ◯ Rarely
- ⊗ Sometimes
- ◯ Usually
- ◯ Always

h. Immigrants and refugees should receive the same care and insurance coverage as US born patients.

- ◯ Never
- ◯ Rarely
- ◯ Sometimes
- ⊗ Usually
- ◯ Always

i. Immigrants and refugees who are undocumented should receive the same care and insurance coverage as US born patients.

- ◯ Never
- ◯ Rarely
- ◯ Sometimes
- ⊗ Usually
- ◯ Always

j. Every physician is professionally obligated to care for immigrants and refugees if they present to your clinic or hospital.

- ◯ Strongly disagree
- ◯ Disagree
- ◯ No opinion
- ⊗ Agree
- ◯ Strongly agree

k. Is healthcare a human right?
- ⊗ Yes
- ◯ No

B. If you wish, please tell us about what you enjoy or do not enjoy about immigrant and refugee health care and the greatest challenges you face in caring for this population.

____

SECTION D: DEMOGRAPHIC INFORMATION
Please answer the following questions by checking the box in front of the response choice that best describes you.

a. Your age?
- ◯ 20 to 24
- ⊗ 25 to 29
- ◯ 30 to 34
- ◯ 35 to 39
- ◯ 40 or older

b. Your gender?
- ◯ Female
- ⊗ Male
- ◯ Other

c.Are you Hispanic or Latino?
- ◯ Yes
- ⊗ No

d. What is your race? (Select one or more responses)
- ◯ American Indian or Alaska Native
- ◯ Asian (Please specify):
- ◯ Black or African American
- ◯ Native Hawaiian or Other Pacific Islander
- ⊗ White
- ◯ Other (Please specify):

e.Were you born in the United States?
- ⊗ Yes
- ◯ No

g. Your residency year?
- ⊗ PGY1
- ◯ PGY2
- ◯ PGY3
- ◯ PGY4
- ◯ PGY5

h. How would you classify your political ideology?
- ◯ Conservative
- ◯ Somewhat conservative
- ◯ Moderate
- ⊗ Somewhat liberal
- ◯ Liberal
- ◯ Other (Please specify):

i. Estimated level of educational debt?
- ◯ None
- ◯ Less than $50,000
- ◯ $50,000 - $100,000
- ⊗ $100,000 - $200,000
- ◯ $200,000 or more

j.Do you plan to subspecialize?
- ⊗ Yes
- ◯ No

k. Languages spoken?

- ☒ English
- ☒ Spanish
- ☐ French
- ☐ Hmong
- ☐ Somali
- ☐ Japanese
- ☐ Chinese
- ☐ Russian
- ☐ Ethiopian
- ☐ Other ___________

l. Are you in the Global Health Pathway?

- ◯ Yes
- ⊗ No

m. Did you earn your degree in the US?

- ⊗ Yes
- ◯ No

n. What residency program are you in?

- ◯ Internal Medicine
- ◯ Med-Peds
- ◯ Pediatrics
- ◯ Family Practice
- ◯ Neurology
- ◯ Psychiatry
- ◯ ObGyn
- ◯ Neurosurgery
- ◯ General Surgery
- ◯ Orthopedic Surgery
- ◯ Urology
- ◯ Surgical sub-specialty (please specify in text box below)
- ⊗ Non-clinical specialty (radiology, pathology; please specify in text box below)

____

Medical Trainees' attitudes, knowledge, and experience with immigrant and refugee health

Response was added on 12/19/2013 8:11pm.

SECTION A: Personal experience with immigrant and refugee health care.

A. Please indicate your level of agreement with the following statements regarding your personal experience with immigrant and refugee health care by checking the box that best represents your experience.

a. During my inpatient rotations, I take care of the following percentage of immigrant and refugee patients:

- ◯ None
- ⊗ 0 -5%
- ◯ 5-10%
- ◯ 10-25%
- ◯ > 25%

b. During my outpatient rotations, I take care of the following percentage of immigrant and refugee patients:

- ◯ None
- ⊗ 0-10%
- ◯ 10 -25%
- ◯ 25-50%
- ◯ 50-75%
- ◯ >75%

c. I would like to take care of more immigrant and refugee patients.

- ◯ Strongly disagree
- ◯ Disagree
- ◯ No opinion
- ◯ Agree
- ⊗ Strongly agree

d. I plan to take care of immigrants and refugees when I finish residency.

- ◯ Strongly disagree
- ◯ Disagree
- ◯ No opinion
- ◯ Agree
- ⊗ Strongly agree

e. I plan to do short term (< 6 months) international work when I finish residency.

- ◯ Strongly disagree
- ◯ Disagree
- ◯ No opinion
- ◯ Agree
- ⊗ Strongly agree

f. I plan to do long term (>6 months) international work when I finish residency.

- ◯ Strongly disagree
- ◯ Disagree
- ◯ No opinion
- ◯ Agree
- ⊗ Strongly agree

g. I plan to work in health disparities in the following way after residency:

I plan to spend multiple months a year in a clinic I fund abroad.

SECTION B: MEDICAL EDUCATION

A. Please indicate your level of agreement with the following statements regarding your medical education and knowledge about immigrants and refugees by checking the box that best represents your opinion.

a. I have received specialized training in immigrant and refugee health, tropical medicine, or cross-cultural health.

- ◯ Strongly disagree
- ⊗ Disagree
- ◯ No opinion
- ◯ Agree
- ◯ Strongly agree

c. I feel comfortable with my fund of knowledge regarding immigrant and refugee health.

- ◯ Strongly disagree
- ⊗ Disagree
- ◯ No opinion
- ◯ Agree
- ◯ Strongly agree

d. I would like to have further training in immigrant and refugee health.

- ◯ Strongly disagree
- ◯ Disagree
- ◯ No opinion
- ◯ Agree
- ⊗ Strongly agree

e. If you agree with the above, please indicate all the contexts in which you would like to receive this training:

- ☒ As part of my residency.
- ☐ A special program.
- ☐ As part of my fellowship.

SECTION C: Attitudes towards immigrant health

A. Please indicate your level of agreement with the following statements regarding immigrant and refugee health by checking the box that best represents your opinion.

a. I enjoy taking care of immigrants and refugees.

- ◯ Never
- ◯ Rarely
- ◯ Sometimes
- ◯ Usually
- ⊗ Always

b. Please indicate the reasons that you enjoy taking care of immigrants and refugees (may choose more than one).

- ☐ Tropical and other conditions not frequently diagnosed in US-born patients
- ☒ Learning about other cultures
- ☐ They don't complain as much
- ☐ Being able to hear their stories
- ☒ Their care is more complicated
- ☐ Their care is less complicated
- ☒ They are very appreciative of your help.
- ☒ They are extremely vulnerable
- ☐ Other:

c. Taking care of immigrants and refugees is more challenging than taking care of US born patients.

- ◯ Never
- ◯ Rarely
- ⊗ Sometimes
- ◯ Usually
- ◯ Always

d. Please mark all the challenges that you face as a provider when providing care to immigrants and refugees (may choose more than one):

- ☒ Language barriers
- ☒ Insurance barriers
- ☒ Cultural barriers
- ☒ Finding a professional interpreter
- ☒ Knowing how to work with a professional interpreter
- ☐ Time constraints
- ☒ My own knowledge related to tropical and travel medicine
- ☐ Transportation problems for the patient
- ☒ Patients not understanding treatment plan
- ☐ Patients not following treatment plan
- ☒ My lack of knowledge regarding the patient's culture
- ☒ Bias or stereotyping
- ☐ Other:

e. Please mark all of the challenges faced by immigrant and refugee populations when receiving healthcare that you have perceived or witnessed (may choose more than one):

- ☒ Language barriers
- ☐ Insurance barriers
- ☒ Cultural barriers
- ☐ Finding a professional interpreter
- ☐ Knowing how to work with a professional interpreter
- ☐ Time constraints
- ☒ Insufficiently trained health care providers
- ☐ Transportation problems for the patient
- ☒ Food insufficiency
- ☐ Need for child care
- ☐ Patients not understanding treatment plan
- ☒ Patients not following treatment plan
- ☐ My lack of knowledge regarding the patient's culture
- ☐ Bias or stereotyping
- ☐ Trust issues
- ☐ Other...

f. Rank how well immigrants and refugees understand the healthcare that you are trying to provide.

- ○ Significantly less than a US born individual
- ⊗ Less than a US born individual
- ○ Equivalent to a US born individual
- ○ More than a US born patient
- ○ Significantly more than a US born individual

g. Immigrants and refugees adhere to treatment plans and follow my recommendations.

- ○ Never
- ○ Rarely
- ⊗ Sometimes
- ○ Usually
- ○ Always

h. Immigrants and refugees should receive the same care and insurance coverage as US born patients.

- ○ Never
- ○ Rarely
- ○ Sometimes
- ○ Usually
- ⊗ Always

i. Immigrants and refugees who are undocumented should receive the same care and insurance coverage as US born patients.

- ○ Never
- ○ Rarely
- ○ Sometimes
- ○ Usually
- ⊗ Always

j. Every physician is professionally obligated to care for immigrants and refugees if they present to your clinic or hospital.

- ○ Strongly disagree
- ○ Disagree
- ○ No opinion
- ○ Agree
- ⊗ Strongly agree

k. Is healthcare a human right?
- ⊗ Yes
- ◯ No

B. If you wish, please tell us about what you enjoy or do not enjoy about immigrant and refugee health care and the greatest challenges you face in caring for this population.

No Comment

SECTION D: DEMOGRAPHIC INFORMATION
Please answer the following questions by checking the box in front of the response choice that best describes you.

a. Your age?
- ◯ 20 to 24
- ⊗ 25 to 29
- ◯ 30 to 34
- ◯ 35 to 39
- ◯ 40 or older

b. Your gender?
- ◯ Female
- ⊗ Male
- ◯ Other

c. Are you Hispanic or Latino?
- ◯ Yes
- ⊗ No

d. What is your race? (Select one or more responses)
- ◯ American Indian or Alaska Native
- ◯ Asian (Please specify):
- ◯ Black or African American
- ◯ Native Hawaiian or Other Pacific Islander
- ⊗ White
- ◯ Other (Please specify):

e. Were you born in the United States?
- ⊗ Yes
- ◯ No

g. Your residency year?
- ⊗ PGY1
- ◯ PGY2
- ◯ PGY3
- ◯ PGY4
- ◯ PGY5

h. How would you classify your political ideology?
- ◯ Conservative
- ◯ Somewhat conservative
- ◯ Moderate
- ⊗ Somewhat liberal
- ◯ Liberal
- ◯ Other (Please specify):

i. Estimated level of educational debt?
- ◯ None
- ◯ Less than $50,000
- ◯ $50,000 - $100,000
- ◯ $100,000 - $200,000
- ⊗ $200,000 or more

j. Do you plan to subspecialize?
- ◯ Yes
- ⊗ No

k. Languages spoken?

- ☒ English
- ☐ Spanish
- ☐ French
- ☐ Hmong
- ☐ Somali
- ☐ Japanese
- ☐ Chinese
- ☐ Russian
- ☐ Ethiopian
- ☐ Other ___________

l. Are you in the Global Health Pathway?

- ⊗ Yes
- ◯ No

m. Did you earn your degree in the US?

- ⊗ Yes
- ◯ No

n. What residency program are you in?

- ⊗ Internal Medicine
- ◯ Med-Peds
- ◯ Pediatrics
- ◯ Family Practice
- ◯ Neurology
- ◯ Psychiatry
- ◯ ObGyn
- ◯ Neurosurgery
- ◯ General Surgery
- ◯ Orthopedic Surgery
- ◯ Urology
- ◯ Surgical sub-specialty (please specify in text box below)
- ◯ Non-clinical specialty (radiology, pathology; please specify in text box below)

Medical Trainees' attitudes, knowledge, and experience with immigrant and refugee health

Response was added on 12/29/2013 3:46pm.

SECTION A: Personal experience with immigrant and refugee health care.

A. Please indicate your level of agreement with the following statements regarding your personal experience with immigrant and refugee health care by checking the box that best represents your experience.

a. During my inpatient rotations, I take care of the following percentage of immigrant and refugee patients:

- ◯ None
- ◯ 0 -5%
- ◯ 5-10%
- ⊗ 10-25%
- ◯ > 25%

b. During my outpatient rotations, I take care of the following percentage of immigrant and refugee patients:

- ◯ None
- ◯ 0-10%
- ⊗ 10 -25%
- ◯ 25-50%
- ◯ 50-75%
- ◯ >75%

c. I would like to take care of more immigrant and refugee patients.

- ◯ Strongly disagree
- ◯ Disagree
- ◯ No opinion
- ⊗ Agree
- ◯ Strongly agree

d. I plan to take care of immigrants and refugees when I finish residency.

- ◯ Strongly disagree
- ◯ Disagree
- ◯ No opinion
- ⊗ Agree
- ◯ Strongly agree

e. I plan to do short term (< 6 months) international work when I finish residency.

- ◯ Strongly disagree
- ◯ Disagree
- ⊗ No opinion
- ◯ Agree
- ◯ Strongly agree

f. I plan to do long term (>6 months) international work when I finish residency.

- ◯ Strongly disagree
- ◯ Disagree
- ⊗ No opinion
- ◯ Agree
- ◯ Strongly agree

g. I plan to work in health disparities in the following way after residency:

By working in an area with less access to healthcare and/or working with under-/un-insured populations.

SECTION B: MEDICAL EDUCATION

A. Please indicate your level of agreement with the following statements regarding your medical education and knowledge about immigrants and refugees by checking the box that best represents your opinion.

a. I have received specialized training in immigrant and refugee health, tropical medicine, or cross-cultural health.

◯ Strongly disagree
⊗ Disagree
◯ No opinion
◯ Agree
◯ Strongly agree

c. I feel comfortable with my fund of knowledge regarding immigrant and refugee health.

◯ Strongly disagree
⊗ Disagree
◯ No opinion
◯ Agree
◯ Strongly agree

d. I would like to have further training in immigrant and refugee health.

◯ Strongly disagree
◯ Disagree
◯ No opinion
◯ Agree
⊗ Strongly agree

e. If you agree with the above, please indicate all the contexts in which you would like to receive this training:

☒ As part of my residency.
☐ A special program.
☐ As part of my fellowship.

SECTION C: Attitudes towards immigrant health

A. Please indicate your level of agreement with the following statements regarding immigrant and refugee health by checking the box that best represents your opinion.

a. I enjoy taking care of immigrants and refugees.

◯ Never
◯ Rarely
◯ Sometimes
◯ Usually
⊗ Always

b. Please indicate the reasons that you enjoy taking care of immigrants and refugees (may choose more than one).

☒ Tropical and other conditions not frequently diagnosed in US-born patients
☒ Learning about other cultures
☐ They don't complain as much
☒ Being able to hear their stories
☐ Their care is more complicated
☐ Their care is less complicated
☒ They are very appreciative of your help.
☒ They are extremely vulnerable
☐ Other:

c. Taking care of immigrants and refugees is more challenging than taking care of US born patients.

◯ Never
◯ Rarely
◯ Sometimes
⊗ Usually
◯ Always

d. Please mark all the challenges that you face as a provider when providing care to immigrants and refugees (may choose more than one):

- ☒ Language barriers
- ☒ Insurance barriers
- ☒ Cultural barriers
- ☒ Finding a professional interpreter
- ☒ Knowing how to work with a professional interpreter
- ☒ Time constraints
- ☒ My own knowledge related to tropical and travel medicine
- ☒ Transportation problems for the patient
- ☒ Patients not understanding treatment plan
- ☒ Patients not following treatment plan
- ☒ My lack of knowledge regarding the patient's culture
- ☒ Bias or stereotyping
- ☐ Other:

e. Please mark all of the challenges faced by immigrant and refugee populations when receiving healthcare that you have perceived or witnessed (may choose more than one):

- ☒ Language barriers
- ☒ Insurance barriers
- ☒ Cultural barriers
- ☒ Finding a professional interpreter
- ☒ Knowing how to work with a professional interpreter
- ☒ Time constraints
- ☒ Insufficiently trained health care providers
- ☒ Transportation problems for the patient
- ☒ Food insufficiency
- ☒ Need for child care
- ☒ Patients not understanding treatment plan
- ☒ Patients not following treatment plan
- ☒ My lack of knowledge regarding the patient's culture
- ☒ Bias or stereotyping
- ☒ Trust issues
- ☐ Other...

f. Rank how well immigrants and refugees understand the healthcare that you are trying to provide.

- ○ Significantly less than a US born individual
- ⊗ Less than a US born individual
- ○ Equivalent to a US born individual
- ○ More than a US born patient
- ○ Significantly more than a US born individual

g. Immigrants and refugees adhere to treatment plans and follow my recommendations.

- ○ Never
- ○ Rarely
- ⊗ Sometimes
- ○ Usually
- ○ Always

h. Immigrants and refugees should receive the same care and insurance coverage as US born patients.

- ○ Never
- ○ Rarely
- ○ Sometimes
- ○ Usually
- ⊗ Always

i. Immigrants and refugees who are undocumented should receive the same care and insurance coverage as US born patients.

- ○ Never
- ○ Rarely
- ○ Sometimes
- ○ Usually
- ⊗ Always

j. Every physician is professionally obligated to care for immigrants and refugees if they present to your clinic or hospital.

- ○ Strongly disagree
- ○ Disagree
- ○ No opinion
- ○ Agree
- ⊗ Strongly agree

k. Is healthcare a human right?

⊗ Yes
◯ No

B. If you wish, please tell us about what you enjoy or do not enjoy about immigrant and refugee health care and the greatest challenges you face in caring for this population.

I enjoy the learning opportunity, especially being able to learn about people and cultures that are different than my own. I think the greatest challenges are my own ignorance about cultural issues and patient perspective and also time.

SECTION D: DEMOGRAPHIC INFORMATION
Please answer the following questions by checking the box in front of the response choice that best describes you.

a. Your age?

◯ 20 to 24
⊗ 25 to 29
◯ 30 to 34
◯ 35 to 39
◯ 40 or older

b. Your gender?

⊗ Female
◯ Male
◯ Other

c. Are you Hispanic or Latino?

◯ Yes
⊗ No

d. What is your race? (Select one or more responses)

◯ American Indian or Alaska Native
◯ Asian (Please specify):
◯ Black or African American
◯ Native Hawaiian or Other Pacific Islander
⊗ White
◯ Other (Please specify):

e. Were you born in the United States?

⊗ Yes
◯ No

g. Your residency year?

⊗ PGY1
◯ PGY2
◯ PGY3
◯ PGY4
◯ PGY5

h. How would you classify your political ideology?

◯ Conservative
◯ Somewhat conservative
⊗ Moderate
◯ Somewhat liberal
◯ Liberal
◯ Other (Please specify):

i. Estimated level of educational debt?

◯ None
◯ Less than $50,000
◯ $50,000 - $100,000
◯ $100,000 - $200,000
⊗ $200,000 or more

j. Do you plan to subspecialize?

◯ Yes
⊗ No

k. Languages spoken?

☒ English
☒ Spanish
☐ French
☐ Hmong
☐ Somali
☐ Japanese
☐ Chinese
☐ Russian
☐ Ethiopian
☐ Other ___________

l. Are you in the Global Health Pathway?

⊗ Yes
◯ No

m. Did you earn your degree in the US?

⊗ Yes
◯ No

n. What residency program are you in?

◯ Internal Medicine
◯ Med-Peds
◯ Pediatrics
⊗ Family Practice
◯ Neurology
◯ Psychiatry
◯ ObGyn
◯ Neurosurgery
◯ General Surgery
◯ Orthopedic Surgery
◯ Urology
◯ Surgical sub-specialty (please specify in text box below)
◯ Non-clinical specialty (radiology, pathology; please specify in text box below)

Medical Trainees' attitudes, knowledge, and experience with immigrant and refugee health

Response was added on 05/01/2014 7:56am.

SECTION A: Personal experience with immigrant and refugee health care.

A. Please indicate your level of agreement with the following statements regarding your personal experience with immigrant and refugee health care by checking the box that best represents your experience.

a. During my inpatient rotations, I take care of the following percentage of immigrant and refugee patients:

- ◯ None
- ⊗ 0 -5%
- ◯ 5-10%
- ◯ 10-25%
- ◯ > 25%

b. During my outpatient rotations, I take care of the following percentage of immigrant and refugee patients:

- ◯ None
- ⊗ 0-10%
- ◯ 10 -25%
- ◯ 25-50%
- ◯ 50-75%
- ◯ >75%

c. I would like to take care of more immigrant and refugee patients.

- ◯ Strongly disagree
- ◯ Disagree
- ⊗ No opinion
- ◯ Agree
- ◯ Strongly agree

d. I plan to take care of immigrants and refugees when I finish residency.

- ◯ Strongly disagree
- ◯ Disagree
- ⊗ No opinion
- ◯ Agree
- ◯ Strongly agree

e. I plan to do short term (< 6 months) international work when I finish residency.

- ⊗ Strongly disagree
- ◯ Disagree
- ◯ No opinion
- ◯ Agree
- ◯ Strongly agree

f. I plan to do long term (>6 months) international work when I finish residency.

- ⊗ Strongly disagree
- ◯ Disagree
- ◯ No opinion
- ◯ Agree
- ◯ Strongly agree

g. I plan to work in health disparities in the following way after residency:

Serving under served populations as ae

SECTION B: MEDICAL EDUCATION

A. Please indicate your level of agreement with the following statements regarding your medical education and knowledge about immigrants and refugees by checking the box that best represents your opinion.

a. I have received specialized training in immigrant and refugee health, tropical medicine, or cross-cultural health.

- ⊗ Strongly disagree
- ○ Disagree
- ○ No opinion
- ○ Agree
- ○ Strongly agree

c. I feel comfortable with my fund of knowledge regarding immigrant and refugee health.

- ⊗ Strongly disagree
- ○ Disagree
- ○ No opinion
- ○ Agree
- ○ Strongly agree

d. I would like to have further training in immigrant and refugee health.

- ○ Strongly disagree
- ○ Disagree
- ○ No opinion
- ⊗ Agree
- ○ Strongly agree

e. If you agree with the above, please indicate all the contexts in which you would like to receive this training:

- ☒ As part of my residency.
- ☐ A special program.
- ☐ As part of my fellowship.

SECTION C: Attitudes towards immigrant health

A. Please indicate your level of agreement with the following statements regarding immigrant and refugee health by checking the box that best represents your opinion.

a. I enjoy taking care of immigrants and refugees.

- ○ Never
- ○ Rarely
- ○ Sometimes
- ⊗ Usually
- ○ Always

b. Please indicate the reasons that you enjoy taking care of immigrants and refugees (may choose more than one).

- ☒ Tropical and other conditions not frequently diagnosed in US-born patients
- ☐ Learning about other cultures
- ☐ They don't complain as much
- ☐ Being able to hear their stories
- ☐ Their care is more complicated
- ☐ Their care is less complicated
- ☐ They are very appreciative of your help.
- ☒ They are extremely vulnerable
- ☐ Other:

c. Taking care of immigrants and refugees is more challenging than taking care of US born patients.

- ○ Never
- ○ Rarely
- ○ Sometimes
- ○ Usually
- ⊗ Always

d. Please mark all the challenges that you face as a provider when providing care to immigrants and refugees (may choose more than one):

- ☒ Language barriers
- ☐ Insurance barriers
- ☒ Cultural barriers
- ☒ Finding a professional interpreter
- ☐ Knowing how to work with a professional interpreter
- ☒ Time constraints
- ☒ My own knowledge related to tropical and travel medicine
- ☒ Transportation problems for the patient
- ☐ Patients not understanding treatment plan
- ☐ Patients not following treatment plan
- ☒ My lack of knowledge regarding the patient's culture
- ☐ Bias or stereotyping
- ☐ Other:

e. Please mark all of the challenges faced by immigrant and refugee populations when receiving healthcare that you have perceived or witnessed (may choose more than one):

- ☒ Language barriers
- ☐ Insurance barriers
- ☒ Cultural barriers
- ☒ Finding a professional interpreter
- ☐ Knowing how to work with a professional interpreter
- ☒ Time constraints
- ☐ Insufficiently trained health care providers
- ☐ Transportation problems for the patient
- ☐ Food insufficiency
- ☐ Need for child care
- ☐ Patients not understanding treatment plan
- ☐ Patients not following treatment plan
- ☒ My lack of knowledge regarding the patient's culture
- ☐ Bias or stereotyping
- ☐ Trust issues
- ☐ Other...

f. Rank how well immigrants and refugees understand the healthcare that you are trying to provide.

- ⊗ Significantly less than a US born individual
- ○ Less than a US born individual
- ○ Equivalent to a US born individual
- ○ More than a US born patient
- ○ Significantly more than a US born individual

g. Immigrants and refugees adhere to treatment plans and follow my recommendations.

- ○ Never
- ○ Rarely
- ○ Sometimes
- ⊗ Usually
- ○ Always

h. Immigrants and refugees should receive the same care and insurance coverage as US born patients.

- ○ Never
- ○ Rarely
- ○ Sometimes
- ○ Usually
- ⊗ Always

i. Immigrants and refugees who are undocumented should receive the same care and insurance coverage as US born patients.

- ○ Never
- ○ Rarely
- ○ Sometimes
- ○ Usually
- ⊗ Always

j. Every physician is professionally obligated to care for immigrants and refugees if they present to your clinic or hospital.

- ○ Strongly disagree
- ○ Disagree
- ○ No opinion
- ○ Agree
- ⊗ Strongly agree

k. Is healthcare a human right?

⊗ Yes
◯ No

B. If you wish, please tell us about what you enjoy or do not enjoy about immigrant and refugee health care and the greatest challenges you face in caring for this population.

____

SECTION D: DEMOGRAPHIC INFORMATION
Please answer the following questions by checking the box in front of the response choice that best describes you.

a. Your age?

◯ 20 to 24
⊗ 25 to 29
◯ 30 to 34
◯ 35 to 39
◯ 40 or older

b. Your gender?

⊗ Female
◯ Male
◯ Other

c. Are you Hispanic or Latino?

◯ Yes
⊗ No

d. What is your race? (Select one or more responses)

◯ American Indian or Alaska Native
◯ Asian (Please specify):
◯ Black or African American
◯ Native Hawaiian or Other Pacific Islander
⊗ White
◯ Other (Please specify):

e. Were you born in the United States?

⊗ Yes
◯ No

g. Your residency year?

◯ PGY1
⊗ PGY2
◯ PGY3
◯ PGY4
◯ PGY5

h. How would you classify your political ideology?

◯ Conservative
◯ Somewhat conservative
◯ Moderate
◯ Somewhat liberal
⊗ Liberal
◯ Other (Please specify):

i. Estimated level of educational debt?

◯ None
◯ Less than $50,000
◯ $50,000 - $100,000
◯ $100,000 - $200,000
⊗ $200,000 or more

j. Do you plan to subspecialize?

◯ Yes
⊗ No

k. Languages spoken?

- ☒ English
- ☐ Spanish
- ☐ French
- ☐ Hmong
- ☐ Somali
- ☐ Japanese
- ☐ Chinese
- ☐ Russian
- ☐ Ethiopian
- ☐ Other ___________

l. Are you in the Global Health Pathway?

- ◯ Yes
- ⊗ No

m. Did you earn your degree in the US?

- ⊗ Yes
- ◯ No

n. What residency program are you in?

- ⊗ Internal Medicine
- ◯ Med-Peds
- ◯ Pediatrics
- ◯ Family Practice
- ◯ Neurology
- ◯ Psychiatry
- ◯ ObGyn
- ◯ Neurosurgery
- ◯ General Surgery
- ◯ Orthopedic Surgery
- ◯ Urology
- ◯ Surgical sub-specialty (please specify in text box below)
- ◯ Non-clinical specialty (radiology, pathology; please specify in text box below)

Medical Trainees' attitudes, knowledge, and experience with immigrant and refugee health

Response was added on 05/01/2014 8:00am.

SECTION A: Personal experience with immigrant and refugee health care.

A. Please indicate your level of agreement with the following statements regarding your personal experience with immigrant and refugee health care by checking the box that best represents your experience.

a. During my inpatient rotations, I take care of the following percentage of immigrant and refugee patients:	◯ None<br>◯ 0 -5%<br>⊗ 5-10%<br>◯ 10-25%<br>◯ > 25%
b. During my outpatient rotations, I take care of the following percentage of immigrant and refugee patients:	⊗ None<br>◯ 0-10%<br>◯ 10 -25%<br>◯ 25-50%<br>◯ 50-75%<br>◯ >75%
c. I would like to take care of more immigrant and refugee patients.	◯ Strongly disagree<br>⊗ Disagree<br>◯ No opinion<br>◯ Agree<br>◯ Strongly agree
d. I plan to take care of immigrants and refugees when I finish residency.	◯ Strongly disagree<br>◯ Disagree<br>⊗ No opinion<br>◯ Agree<br>◯ Strongly agree
e. I plan to do short term (< 6 months) international work when I finish residency.	⊗ Strongly disagree<br>◯ Disagree<br>◯ No opinion<br>◯ Agree<br>◯ Strongly agree
f. I plan to do long term (>6 months) international work when I finish residency.	⊗ Strongly disagree<br>◯ Disagree<br>◯ No opinion<br>◯ Agree<br>◯ Strongly agree
g. I plan to work in health disparities in the following way after residency:	Plan to work at the VA, which is an under served population

SECTION B: MEDICAL EDUCATION

A. Please indicate your level of agreement with the following statements regarding your medical education and knowledge about immigrants and refugees by checking the box that best represents your opinion.

a. I have received specialized training in immigrant and refugee health, tropical medicine, or cross-cultural health.

- ○ Strongly disagree
- ⊗ Disagree
- ○ No opinion
- ○ Agree
- ○ Strongly agree

c. I feel comfortable with my fund of knowledge regarding immigrant and refugee health.

- ○ Strongly disagree
- ⊗ Disagree
- ○ No opinion
- ○ Agree
- ○ Strongly agree

d. I would like to have further training in immigrant and refugee health.

- ⊗ Strongly disagree
- ○ Disagree
- ○ No opinion
- ○ Agree
- ○ Strongly agree

SECTION C: Attitudes towards immigrant health

A. Please indicate your level of agreement with the following statements regarding immigrant and refugee health by checking the box that best represents your opinion.

a. I enjoy taking care of immigrants and refugees.

- ○ Never
- ⊗ Rarely
- ○ Sometimes
- ○ Usually
- ○ Always

b. Please indicate the reasons that you enjoy taking care of immigrants and refugees (may choose more than one).

- ☐ Tropical and other conditions not frequently diagnosed in US-born patients
- ☐ Learning about other cultures
- ☐ They don't complain as much
- ☐ Being able to hear their stories
- ☐ Their care is more complicated
- ☐ Their care is less complicated
- ☐ They are very appreciative of your help.
- ☐ They are extremely vulnerable
- ☐ Other:

c. Taking care of immigrants and refugees is more challenging than taking care of US born patients.

- ○ Never
- ○ Rarely
- ○ Sometimes
- ⊗ Usually
- ○ Always

d. Please mark all the challenges that you face as a provider when providing care to immigrants and refugees (may choose more than one):

- ☒ Language barriers
- ☐ Insurance barriers
- ☒ Cultural barriers
- ☐ Finding a professional interpreter
- ☐ Knowing how to work with a professional interpreter
- ☐ Time constraints
- ☒ My own knowledge related to tropical and travel medicine
- ☐ Transportation problems for the patient
- ☒ Patients not understanding treatment plan
- ☒ Patients not following treatment plan
- ☐ My lack of knowledge regarding the patient's culture
- ☐ Bias or stereotyping
- ☐ Other:

e. Please mark all of the challenges faced by immigrant and refugee populations when receiving healthcare that you have perceived or witnessed (may choose more than one):

- ☐ Language barriers
- ☐ Insurance barriers
- ☐ Cultural barriers
- ☐ Finding a professional interpreter
- ☐ Knowing how to work with a professional interpreter
- ☐ Time constraints
- ☐ Insufficiently trained health care providers
- ☐ Transportation problems for the patient
- ☐ Food insufficiency
- ☐ Need for child care
- ☐ Patients not understanding treatment plan
- ☐ Patients not following treatment plan
- ☐ My lack of knowledge regarding the patient's culture
- ☐ Bias or stereotyping
- ☐ Trust issues
- ☐ Other...

f. Rank how well immigrants and refugees understand the healthcare that you are trying to provide.

- ◯ Significantly less than a US born individual
- ⊗ Less than a US born individual
- ◯ Equivalent to a US born individual
- ◯ More than a US born patient
- ◯ Significantly more than a US born individual

g. Immigrants and refugees adhere to treatment plans and follow my recommendations.

- ◯ Never
- ◯ Rarely
- ⊗ Sometimes
- ◯ Usually
- ◯ Always

h. Immigrants and refugees should receive the same care and insurance coverage as US born patients.

- ◯ Never
- ◯ Rarely
- ◯ Sometimes
- ◯ Usually
- ⊗ Always

i. Immigrants and refugees who are undocumented should receive the same care and insurance coverage as US born patients.

- ◯ Never
- ◯ Rarely
- ◯ Sometimes
- ◯ Usually
- ⊗ Always

j. Every physician is professionally obligated to care for immigrants and refugees if they present to your clinic or hospital.

- ◯ Strongly disagree
- ◯ Disagree
- ◯ No opinion
- ◯ Agree
- ⊗ Strongly agree

k. Is healthcare a human right?

- ⊗ Yes
- ◯ No

B. If you wish, please tell us about what you enjoy or do not enjoy about immigrant and refugee health care and the greatest challenges you face in caring for this population.

____

SECTION D: DEMOGRAPHIC INFORMATION
Please answer the following questions by checking the box in front of the response choice that best describes you.

a. Your age?	◯ 20 to 24<br>⊗ 25 to 29<br>◯ 30 to 34<br>◯ 35 to 39<br>◯ 40 or older
b. Your gender?	◯ Female<br>⊗ Male<br>◯ Other
c.Are you Hispanic or Latino?	◯ Yes<br>⊗ No
d. What is your race? (Select one or more responses)	◯ American Indian or Alaska Native<br>◯ Asian (Please specify):<br>◯ Black or African American<br>◯ Native Hawaiian or Other Pacific Islander<br>⊗ White<br>◯ Other (Please specify):
e.Were you born in the United States?	⊗ Yes<br>◯ No
g. Your residency year?	◯ PGY1<br>◯ PGY2<br>⊗ PGY3<br>◯ PGY4<br>◯ PGY5
h. How would you classify your political ideology?	◯ Conservative<br>◯ Somewhat conservative<br>◯ Moderate<br>◯ Somewhat liberal<br>⊗ Liberal<br>◯ Other (Please specify):
i. Estimated level of educational debt?	◯ None<br>◯ Less than $50,000<br>◯ $50,000 - $100,000<br>⊗ $100,000 - $200,000<br>◯ $200,000 or more
j.Do you plan to subspecialize?	⊗ Yes<br>◯ No
k. Languages spoken?	☒ English<br>☐ Spanish<br>☐ French<br>☐ Hmong<br>☐ Somali<br>☐ Japanese<br>☐ Chinese<br>☐ Russian<br>☐ Ethiopian<br>☐ Other ___________
l. Are you in the Global Health Pathway?	◯ Yes<br>⊗ No

m. Did you earn your degree in the US?

- ⊗ Yes
- ○ No

n. What residency program are you in?

- ⊗ Internal Medicine
- ○ Med-Peds
- ○ Pediatrics
- ○ Family Practice
- ○ Neurology
- ○ Psychiatry
- ○ ObGyn
- ○ Neurosurgery
- ○ General Surgery
- ○ Orthopedic Surgery
- ○ Urology
- ○ Surgical sub-specialty (please specify in text box below)
- ○ Non-clinical specialty (radiology, pathology; please specify in text box below)

Medical Trainees' attitudes, knowledge, and experience with immigrant and refugee health

Response was added on 05/01/2014 8:40am.

SECTION A: Personal experience with immigrant and refugee health care.

A. Please indicate your level of agreement with the following statements regarding your personal experience with immigrant and refugee health care by checking the box that best represents your experience.

a. During my inpatient rotations, I take care of the following percentage of immigrant and refugee patients:

- ◯ None
- ◯ 0 -5%
- ◯ 5-10%
- ⊗ 10-25%
- ◯ > 25%

b. During my outpatient rotations, I take care of the following percentage of immigrant and refugee patients:

- ◯ None
- ⊗ 0-10%
- ◯ 10 -25%
- ◯ 25-50%
- ◯ 50-75%
- ◯ >75%

c. I would like to take care of more immigrant and refugee patients.

- ◯ Strongly disagree
- ◯ Disagree
- ◯ No opinion
- ⊗ Agree
- ◯ Strongly agree

d. I plan to take care of immigrants and refugees when I finish residency.

- ◯ Strongly disagree
- ◯ Disagree
- ⊗ No opinion
- ◯ Agree
- ◯ Strongly agree

e. I plan to do short term (< 6 months) international work when I finish residency.

- ⊗ Strongly disagree
- ◯ Disagree
- ◯ No opinion
- ◯ Agree
- ◯ Strongly agree

f. I plan to do long term (>6 months) international work when I finish residency.

- ⊗ Strongly disagree
- ◯ Disagree
- ◯ No opinion
- ◯ Agree
- ◯ Strongly agree

g. I plan to work in health disparities in the following way after residency:

I want to focus on racial disparities in hematology but not necessarily focused on immigrants

SECTION B: MEDICAL EDUCATION

A. Please indicate your level of agreement with the following statements regarding your medical education and knowledge about immigrants and refugees by checking the box that best represents your opinion.

a. I have received specialized training in immigrant and refugee health, tropical medicine, or cross-cultural health.

- ◯ Strongly disagree
- ⊗ Disagree
- ⊗ No opinion
- ◯ Agree
- ◯ Strongly agree

c. I feel comfortable with my fund of knowledge regarding immigrant and refugee health.

- ◯ Strongly disagree
- ⊗ Disagree
- ◯ No opinion
- ◯ Agree
- ◯ Strongly agree

d. I would like to have further training in immigrant and refugee health.

- ◯ Strongly disagree
- ◯ Disagree
- ◯ No opinion
- ⊗ Agree
- ◯ Strongly agree

e. If you agree with the above, please indicate all the contexts in which you would like to receive this training:

- ☒ As part of my residency.
- ☐ A special program.
- ☐ As part of my fellowship.

SECTION C: Attitudes towards immigrant health

A. Please indicate your level of agreement with the following statements regarding immigrant and refugee health by checking the box that best represents your opinion.

a. I enjoy taking care of immigrants and refugees.

- ◯ Never
- ◯ Rarely
- ◯ Sometimes
- ⊗ Usually
- ◯ Always

b. Please indicate the reasons that you enjoy taking care of immigrants and refugees (may choose more than one).

- ☐ Tropical and other conditions not frequently diagnosed in US-born patients
- ☒ Learning about other cultures
- ☐ They don't complain as much
- ☐ Being able to hear their stories
- ☐ Their care is more complicated
- ☐ Their care is less complicated
- ☐ They are very appreciative of your help.
- ☒ They are extremely vulnerable
- ☐ Other:

c. Taking care of immigrants and refugees is more challenging than taking care of US born patients.

- ◯ Never
- ◯ Rarely
- ◯ Sometimes
- ⊗ Usually
- ◯ Always

d. Please mark all the challenges that you face as a provider when providing care to immigrants and refugees (may choose more than one):

- ☒ Language barriers
- ☒ Insurance barriers
- ☒ Cultural barriers
- ☐ Finding a professional interpreter
- ☐ Knowing how to work with a professional interpreter
- ☒ Time constraints
- ☐ My own knowledge related to tropical and travel medicine
- ☐ Transportation problems for the patient
- ☐ Patients not understanding treatment plan
- ☐ Patients not following treatment plan
- ☒ My lack of knowledge regarding the patient's culture
- ☐ Bias or stereotyping
- ☐ Other:

e. Please mark all of the challenges faced by immigrant and refugee populations when receiving healthcare that you have perceived or witnessed (may choose more than one):

- ☒ Language barriers
- ☒ Insurance barriers
- ☒ Cultural barriers
- ☐ Finding a professional interpreter
- ☐ Knowing how to work with a professional interpreter
- ☐ Time constraints
- ☒ Insufficiently trained health care providers
- ☒ Transportation problems for the patient
- ☐ Food insufficiency
- ☐ Need for child care
- ☐ Patients not understanding treatment plan
- ☐ Patients not following treatment plan
- ☐ My lack of knowledge regarding the patient's culture
- ☒ Bias or stereotyping
- ☒ Trust issues
- ☐ Other...

f. Rank how well immigrants and refugees understand the healthcare that you are trying to provide.

- ○ Significantly less than a US born individual
- ⊗ Less than a US born individual
- ○ Equivalent to a US born individual
- ○ More than a US born patient
- ○ Significantly more than a US born individual

g. Immigrants and refugees adhere to treatment plans and follow my recommendations.

- ○ Never
- ○ Rarely
- ⊗ Sometimes
- ○ Usually
- ○ Always

h. Immigrants and refugees should receive the same care and insurance coverage as US born patients.

- ○ Never
- ○ Rarely
- ○ Sometimes
- ⊗ Usually
- ○ Always

i. Immigrants and refugees who are undocumented should receive the same care and insurance coverage as US born patients.

- ○ Never
- ○ Rarely
- ⊗ Sometimes
- ○ Usually
- ○ Always

j. Every physician is professionally obligated to care for immigrants and refugees if they present to your clinic or hospital.

- ○ Strongly disagree
- ○ Disagree
- ○ No opinion
- ⊗ Agree
- ○ Strongly agree

k. Is healthcare a human right?

⊗ Yes
◯ No

B. If you wish, please tell us about what you enjoy or do not enjoy about immigrant and refugee health care and the greatest challenges you face in caring for this population.

____

SECTION D: DEMOGRAPHIC INFORMATION
Please answer the following questions by checking the box in front of the response choice that best describes you.

a. Your age?

◯ 20 to 24
⊗ 25 to 29
◯ 30 to 34
◯ 35 to 39
◯ 40 or older

b. Your gender?

◯ Female
⊗ Male
◯ Other

c.Are you Hispanic or Latino?

◯ Yes
⊗ No

d. What is your race? (Select one or more responses)

◯ American Indian or Alaska Native
◯ Asian (Please specify):
◯ Black or African American
◯ Native Hawaiian or Other Pacific Islander
⊗ White
◯ Other (Please specify):

e.Were you born in the United States?

⊗ Yes
◯ No

g. Your residency year?

◯ PGY1
⊗ PGY2
◯ PGY3
◯ PGY4
◯ PGY5

h. How would you classify your political ideology?

◯ Conservative
◯ Somewhat conservative
◯ Moderate
⊗ Somewhat liberal
◯ Liberal
◯ Other (Please specify):

i. Estimated level of educational debt?

◯ None
◯ Less than $50,000
⊗ $50,000 - $100,000
◯ $100,000 - $200,000
◯ $200,000 or more

j.Do you plan to subspecialize?

⊗ Yes
◯ No

k. Languages spoken?

☒ English
☐ Spanish
☐ French
☐ Hmong
☐ Somali
☐ Japanese
☐ Chinese
☐ Russian
☐ Ethiopian
☐ Other ___________

l. Are you in the Global Health Pathway?

◯ Yes
⊗ No

m. Did you earn your degree in the US?

⊗ Yes
◯ No

n. What residency program are you in?

◯ Internal Medicine
⊗ Med-Peds
◯ Pediatrics
◯ Family Practice
◯ Neurology
◯ Psychiatry
◯ ObGyn
◯ Neurosurgery
◯ General Surgery
◯ Orthopedic Surgery
◯ Urology
◯ Surgical sub-specialty (please specify in text box below)
◯ Non-clinical specialty (radiology, pathology; please specify in text box below)

Medical Trainees' attitudes, knowledge, and experience with immigrant and refugee health

Response was added on 05/01/2014 10:11am.

SECTION A: Personal experience with immigrant and refugee health care.

A. Please indicate your level of agreement with the following statements regarding your personal experience with immigrant and refugee health care by checking the box that best represents your experience.

a. During my inpatient rotations, I take care of the following percentage of immigrant and refugee patients:	◯ None<br>◯ 0 -5%<br>⊗ 5-10%<br>◯ 10-25%<br>◯ > 25%
b. During my outpatient rotations, I take care of the following percentage of immigrant and refugee patients:	⊗ None<br>◯ 0-10%<br>◯ 10 -25%<br>◯ 25-50%<br>◯ 50-75%<br>◯ >75%
c. I would like to take care of more immigrant and refugee patients.	◯ Strongly disagree<br>◯ Disagree<br>◯ No opinion<br>◯ Agree<br>⊗ Strongly agree
d. I plan to take care of immigrants and refugees when I finish residency.	◯ Strongly disagree<br>◯ Disagree<br>◯ No opinion<br>⊗ Agree<br>◯ Strongly agree
e. I plan to do short term (< 6 months) international work when I finish residency.	◯ Strongly disagree<br>◯ Disagree<br>◯ No opinion<br>◯ Agree<br>⊗ Strongly agree
f. I plan to do long term (>6 months) international work when I finish residency.	◯ Strongly disagree<br>◯ Disagree<br>◯ No opinion<br>⊗ Agree<br>◯ Strongly agree
g. I plan to work in health disparities in the following way after residency:	Not sure

SECTION B: MEDICAL EDUCATION

A. Please indicate your level of agreement with the following statements regarding your medical education and knowledge about immigrants and refugees by checking the box that best represents your opinion.

a. I have received specialized training in immigrant and refugee health, tropical medicine, or cross-cultural health.

- ◯ Strongly disagree
- ◯ Disagree
- ◯ No opinion
- ⊗ Agree
- ◯ Strongly agree

b. If you have received specialized training in immigrant and refugee health, tropical medicine, or cross-cultural health, please indicate all the contexts in which you received this training:

- ☐ As an undergraduate.
- ☐ As a medical student.
- ☒ As part of my residency.
- ☒ A special program.
- ☐ As part of my fellowship.
- ☐ As part of a degree program (e.g. MPH)
- ☐ Other:

c. I feel comfortable with my fund of knowledge regarding immigrant and refugee health.

- ◯ Strongly disagree
- ◯ Disagree
- ◯ No opinion
- ⊗ Agree
- ◯ Strongly agree

d. I would like to have further training in immigrant and refugee health.

- ◯ Strongly disagree
- ◯ Disagree
- ◯ No opinion
- ◯ Agree
- ⊗ Strongly agree

e. If you agree with the above, please indicate all the contexts in which you would like to receive this training:
□□

- ☒ As part of my residency.
- ☒ A special program.
- ☐ As part of my fellowship.

SECTION C: Attitudes towards immigrant health

A.□Please indicate your level of agreement with the following statements regarding immigrant and refugee health by checking the box that best represents your opinion.

a. I enjoy taking care of immigrants and refugees.

- ◯ Never
- ◯ Rarely
- ◯ Sometimes
- ⊗ Usually
- ◯ Always

b. Please indicate the reasons that you enjoy taking care of immigrants and refugees (may choose more than one).

- ☒ Tropical and other conditions not frequently diagnosed in US-born patients
- ☒ Learning about other cultures
- ☐ They don't complain as much
- ☐ Being able to hear their stories
- ☐ Their care is more complicated
- ☐ Their care is less complicated
- ☐ They are very appreciative of your help.
- ☒ They are extremely vulnerable
- ☐ Other:

c. Taking care of immigrants and refugees is more challenging than taking care of US born patients.

- ◯ Never
- ◯ Rarely
- ◯ Sometimes
- ◯ Usually
- ⊗ Always

d. Please mark all the challenges that you face as a provider when providing care to immigrants and refugees (may choose more than one):

- ☒ Language barriers
- ☐ Insurance barriers
- ☒ Cultural barriers
- ☒ Finding a professional interpreter
- ☐ Knowing how to work with a professional interpreter
- ☒ Time constraints
- ☐ My own knowledge related to tropical and travel medicine
- ☐ Transportation problems for the patient
- ☒ Patients not understanding treatment plan
- ☒ Patients not following treatment plan
- ☒ My lack of knowledge regarding the patient's culture
- ☐ Bias or stereotyping
- ☐ Other:

e. Please mark all of the challenges faced by immigrant and refugee populations when receiving healthcare that you have perceived or witnessed (may choose more than one):

- ☒ Language barriers
- ☒ Insurance barriers
- ☒ Cultural barriers
- ☒ Finding a professional interpreter
- ☒ Knowing how to work with a professional interpreter
- ☒ Time constraints
- ☒ Insufficiently trained health care providers
- ☒ Transportation problems for the patient
- ☒ Food insufficiency
- ☒ Need for child care
- ☒ Patients not understanding treatment plan
- ☒ Patients not following treatment plan
- ☒ My lack of knowledge regarding the patient's culture
- ☒ Bias or stereotyping
- ☒ Trust issues
- ☐ Other...

f. Rank how well immigrants and refugees understand the healthcare that you are trying to provide.

- ○ Significantly less than a US born individual
- ⊗ Less than a US born individual
- ○ Equivalent to a US born individual
- ○ More than a US born patient
- ○ Significantly more than a US born individual

g. Immigrants and refugees adhere to treatment plans and follow my recommendations.

- ○ Never
- ○ Rarely
- ⊗ Sometimes
- ○ Usually
- ○ Always

h. Immigrants and refugees should receive the same care and insurance coverage as US born patients.

- ○ Never
- ○ Rarely
- ○ Sometimes
- ○ Usually
- ⊗ Always

i. Immigrants and refugees who are undocumented should receive the same care and insurance coverage as US born patients.

- ○ Never
- ○ Rarely
- ○ Sometimes
- ○ Usually
- ⊗ Always

j. Every physician is professionally obligated to care for immigrants and refugees if they present to your clinic or hospital.

- ○ Strongly disagree
- ○ Disagree
- ○ No opinion
- ○ Agree
- ⊗ Strongly agree

k. Is healthcare a human right?	⊗ Yes<br>○ No
B. If you wish, please tell us about what you enjoy or do not enjoy about immigrant and refugee health care and the greatest challenges you face in caring for this population.	Bigest challenge hands down is time constraints - finding interpreter, the doubled time that working with an interpreter requires, the time working around cultural restaraints and figuring out different expressions of disease symptoms, etc

SECTION D: DEMOGRAPHIC INFORMATION
Please answer the following questions by checking the box in front of the response choice that best describes you.

a. Your age?	○ 20 to 24<br>○ 25 to 29<br>⊗ 30 to 34<br>○ 35 to 39<br>○ 40 or older
b. Your gender?	○ Female<br>⊗ Male<br>○ Other
c. Are you Hispanic or Latino?	○ Yes<br>⊗ No
d. What is your race? (Select one or more responses)	○ American Indian or Alaska Native<br>○ Asian (Please specify):<br>○ Black or African American<br>○ Native Hawaiian or Other Pacific Islander<br>⊗ White<br>○ Other (Please specify):
e. Were you born in the United States?	⊗ Yes<br>○ No
g. Your residency year?	○ PGY1<br>○ PGY2<br>⊗ PGY3<br>○ PGY4<br>○ PGY5
h. How would you classify your political ideology?	○ Conservative<br>○ Somewhat conservative<br>○ Moderate<br>⊗ Somewhat liberal<br>○ Liberal<br>○ Other (Please specify):
i. Estimated level of educational debt?	○ None<br>○ Less than $50,000<br>○ $50,000 - $100,000<br>○ $100,000 - $200,000<br>⊗ $200,000 or more
j. Do you plan to subspecialize?	⊗ Yes<br>○ No

k. Languages spoken?

- ☒ English
- ☐ Spanish
- ☐ French
- ☐ Hmong
- ☐ Somali
- ☐ Japanese
- ☐ Chinese
- ☐ Russian
- ☐ Ethiopian
- ☐ Other ___________

l. Are you in the Global Health Pathway?

- ◯ Yes
- ⊗ No

m. Did you earn your degree in the US?

- ⊗ Yes
- ◯ No

n. What residency program are you in?

- ⊗ Internal Medicine
- ◯ Med-Peds
- ◯ Pediatrics
- ◯ Family Practice
- ◯ Neurology
- ◯ Psychiatry
- ◯ ObGyn
- ◯ Neurosurgery
- ◯ General Surgery
- ◯ Orthopedic Surgery
- ◯ Urology
- ◯ Surgical sub-specialty (please specify in text box below)
- ◯ Non-clinical specialty (radiology, pathology; please specify in text box below)

Medical Trainees' attitudes, knowledge, and experience with immigrant and refugee health

Response was added on 05/01/2014 3:52pm.

SECTION A: Personal experience with immigrant and refugee health care.

A. Please indicate your level of agreement with the following statements regarding your personal experience with immigrant and refugee health care by checking the box that best represents your experience.

a. During my inpatient rotations, I take care of the following percentage of immigrant and refugee patients:
- ◯ None
- ⊗ 0 -5%
- ◯ 5-10%
- ◯ 10-25%
- ◯ > 25%

b. During my outpatient rotations, I take care of the following percentage of immigrant and refugee patients:
- ◯ None
- ◯ 0-10%
- ⊗ 10 -25%
- ◯ 25-50%
- ◯ 50-75%
- ◯ >75%

c. I would like to take care of more immigrant and refugee patients.
- ◯ Strongly disagree
- ⊗ Disagree
- ◯ No opinion
- ◯ Agree
- ◯ Strongly agree

d. I plan to take care of immigrants and refugees when I finish residency.
- ◯ Strongly disagree
- ◯ Disagree
- ◯ No opinion
- ⊗ Agree
- ◯ Strongly agree

e. I plan to do short term (< 6 months) international work when I finish residency.
- ◯ Strongly disagree
- ⊗ Disagree
- ◯ No opinion
- ◯ Agree
- ◯ Strongly agree

f. I plan to do long term (>6 months) international work when I finish residency.
- ⊗ Strongly disagree
- ◯ Disagree
- ◯ No opinion
- ◯ Agree
- ◯ Strongly agree

g. I plan to work in health disparities in the following way after residency: health policy

SECTION B: MEDICAL EDUCATION

A. Please indicate your level of agreement with the following statements regarding your medical education and knowledge about immigrants and refugees by checking the box that best represents your opinion.

a. I have received specialized training in immigrant and refugee health, tropical medicine, or cross-cultural health.

◯ Strongly disagree
◯ Disagree
◯ No opinion
⊗ Agree
◯ Strongly agree

b. If you have received specialized training in immigrant and refugee health, tropical medicine, or cross-cultural health, please indicate all the contexts in which you received this training:

☐ As an undergraduate.
☒ As a medical student.
☒ As part of my residency.
☐ A special program.
☐ As part of my fellowship.
☐ As part of a degree program (e.g. MPH)
☐ Other:

c. I feel comfortable with my fund of knowledge regarding immigrant and refugee health.

◯ Strongly disagree
⊗ Disagree
◯ No opinion
◯ Agree
◯ Strongly agree

d. I would like to have further training in immigrant and refugee health.

◯ Strongly disagree
◯ Disagree
◯ No opinion
⊗ Agree
◯ Strongly agree

e. If you agree with the above, please indicate all the contexts in which you would like to receive this training:

☒ As part of my residency.
☐ A special program.
☐ As part of my fellowship.

SECTION C: Attitudes towards immigrant health

A. Please indicate your level of agreement with the following statements regarding immigrant and refugee health by checking the box that best represents your opinion.

a. I enjoy taking care of immigrants and refugees.

◯ Never
◯ Rarely
◯ Sometimes
⊗ Usually
◯ Always

b. Please indicate the reasons that you enjoy taking care of immigrants and refugees (may choose more than one).

☒ Tropical and other conditions not frequently diagnosed in US-born patients
☐ Learning about other cultures
☐ They don't complain as much
☐ Being able to hear their stories
☐ Their care is more complicated
☐ Their care is less complicated
☐ They are very appreciative of your help.
☒ They are extremely vulnerable
☐ Other:

c. Taking care of immigrants and refugees is more challenging than taking care of US born patients.

◯ Never
◯ Rarely
◯ Sometimes
⊗ Usually
◯ Always

d. Please mark all the challenges that you face as a provider when providing care to immigrants and refugees (may choose more than one):

- ☒ Language barriers
- ☒ Insurance barriers
- ☐ Cultural barriers
- ☐ Finding a professional interpreter
- ☐ Knowing how to work with a professional interpreter
- ☐ Time constraints
- ☐ My own knowledge related to tropical and travel medicine
- ☒ Transportation problems for the patient
- ☒ Patients not understanding treatment plan
- ☒ Patients not following treatment plan
- ☐ My lack of knowledge regarding the patient's culture
- ☐ Bias or stereotyping
- ☐ Other:

e. Please mark all of the challenges faced by immigrant and refugee populations when receiving healthcare that you have perceived or witnessed (may choose more than one):

- ☒ Language barriers
- ☒ Insurance barriers
- ☐ Cultural barriers
- ☐ Finding a professional interpreter
- ☒ Knowing how to work with a professional interpreter
- ☐ Time constraints
- ☐ Insufficiently trained health care providers
- ☒ Transportation problems for the patient
- ☒ Food insufficiency
- ☒ Need for child care
- ☒ Patients not understanding treatment plan
- ☒ Patients not following treatment plan
- ☐ My lack of knowledge regarding the patient's culture
- ☐ Bias or stereotyping
- ☐ Trust issues
- ☐ Other...

f. Rank how well immigrants and refugees understand the healthcare that you are trying to provide.

- ○ Significantly less than a US born individual
- ⊗ Less than a US born individual
- ○ Equivalent to a US born individual
- ○ More than a US born patient
- ○ Significantly more than a US born individual

g. Immigrants and refugees adhere to treatment plans and follow my recommendations.

- ○ Never
- ○ Rarely
- ⊗ Sometimes
- ○ Usually
- ○ Always

h. Immigrants and refugees should receive the same care and insurance coverage as US born patients.

- ○ Never
- ○ Rarely
- ○ Sometimes
- ○ Usually
- ⊗ Always

i. Immigrants and refugees who are undocumented should receive the same care and insurance coverage as US born patients.

- ○ Never
- ○ Rarely
- ○ Sometimes
- ⊗ Usually
- ○ Always

j. Every physician is professionally obligated to care for immigrants and refugees if they present to your clinic or hospital.

- ○ Strongly disagree
- ○ Disagree
- ○ No opinion
- ○ Agree
- ⊗ Strongly agree

k. Is healthcare a human right?

- ⊗ Yes
- ◯ No

B. If you wish, please tell us about what you enjoy or do not enjoy about immigrant and refugee health care and the greatest challenges you face in caring for this population.

____

SECTION D: DEMOGRAPHIC INFORMATION
Please answer the following questions by checking the box in front of the response choice that best describes you.

a. Your age?

- ◯ 20 to 24
- ⊗ 25 to 29
- ◯ 30 to 34
- ◯ 35 to 39
- ◯ 40 or older

b. Your gender?

- ◯ Female
- ⊗ Male
- ◯ Other

c.Are you Hispanic or Latino?

- ◯ Yes
- ⊗ No

d. What is your race? (Select one or more responses)

- ◯ American Indian or Alaska Native
- ◯ Asian (Please specify):
- ◯ Black or African American
- ◯ Native Hawaiian or Other Pacific Islander
- ⊗ White
- ◯ Other (Please specify):

e.Were you born in the United States?

- ⊗ Yes
- ◯ No

g. Your residency year?

- ◯ PGY1
- ◯ PGY2
- ⊗ PGY3
- ◯ PGY4
- ◯ PGY5

h. How would you classify your political ideology?

- ◯ Conservative
- ◯ Somewhat conservative
- ◯ Moderate
- ◯ Somewhat liberal
- ⊗ Liberal
- ◯ Other (Please specify):

i. Estimated level of educational debt?

- ◯ None
- ◯ Less than $50,000
- ◯ $50,000 - $100,000
- ◯ $100,000 - $200,000
- ⊗ $200,000 or more

j.Do you plan to subspecialize?

- ◯ Yes
- ⊗ No

k. Languages spoken?

- ☒ English
- ☐ Spanish
- ☐ French
- ☐ Hmong
- ☐ Somali
- ☐ Japanese
- ☐ Chinese
- ☐ Russian
- ☐ Ethiopian
- ☐ Other ___________

l. Are you in the Global Health Pathway?

- ◯ Yes
- ⊗ No

m. Did you earn your degree in the US?

- ⊗ Yes
- ◯ No

n. What residency program are you in?

- ◯ Internal Medicine
- ⊗ Med-Peds
- ◯ Pediatrics
- ◯ Family Practice
- ◯ Neurology
- ◯ Psychiatry
- ◯ ObGyn
- ◯ Neurosurgery
- ◯ General Surgery
- ◯ Orthopedic Surgery
- ◯ Urology
- ◯ Surgical sub-specialty (please specify in text box below)
- ◯ Non-clinical specialty (radiology, pathology; please specify in text box below)

Medical Trainees' attitudes, knowledge, and experience with immigrant and refugee health

Response was added on 05/01/2014 5:34pm.

SECTION A: Personal experience with immigrant and refugee health care.

A. Please indicate your level of agreement with the following statements regarding your personal experience with immigrant and refugee health care by checking the box that best represents your experience.

a. During my inpatient rotations, I take care of the following percentage of immigrant and refugee patients:	◯ None<br>◯ 0 -5%<br>⊗ 5-10%<br>◯ 10-25%<br>◯ > 25%
b. During my outpatient rotations, I take care of the following percentage of immigrant and refugee patients:	◯ None<br>◯ 0-10%<br>◯ 10 -25%<br>◯ 25-50%<br>◯ 50-75%<br>⊗ >75%
c. I would like to take care of more immigrant and refugee patients.	◯ Strongly disagree<br>◯ Disagree<br>◯ No opinion<br>◯ Agree<br>⊗ Strongly agree
d. I plan to take care of immigrants and refugees when I finish residency.	◯ Strongly disagree<br>◯ Disagree<br>◯ No opinion<br>◯ Agree<br>⊗ Strongly agree
e. I plan to do short term (< 6 months) international work when I finish residency.	◯ Strongly disagree<br>◯ Disagree<br>◯ No opinion<br>◯ Agree<br>⊗ Strongly agree
f. I plan to do long term (>6 months) international work when I finish residency.	◯ Strongly disagree<br>◯ Disagree<br>◯ No opinion<br>◯ Agree<br>⊗ Strongly agree
g. I plan to work in health disparities in the following way after residency:	Working with IO's, NGO's. Potentially public health or policy issues in developing nations or refugee care.

SECTION B: MEDICAL EDUCATION

A. Please indicate your level of agreement with the following statements regarding your medical education and knowledge about immigrants and refugees by checking the box that best represents your opinion.

a. I have received specialized training in immigrant and refugee health, tropical medicine, or cross-cultural health.

- ◯ Strongly disagree
- ◯ Disagree
- ◯ No opinion
- ⊗ Agree
- ◯ Strongly agree

b. If you have received specialized training in immigrant and refugee health, tropical medicine, or cross-cultural health, please indicate all the contexts in which you received this training:

- ☐ As an undergraduate.
- ☐ As a medical student.
- ☒ As part of my residency.
- ☒ A special program.
- ☐ As part of my fellowship.
- ☐ As part of a degree program (e.g. MPH)
- ☐ Other:

c. I feel comfortable with my fund of knowledge regarding immigrant and refugee health.

- ◯ Strongly disagree
- ⊗ Disagree
- ◯ No opinion
- ◯ Agree
- ◯ Strongly agree

d. I would like to have further training in immigrant and refugee health.

- ◯ Strongly disagree
- ◯ Disagree
- ◯ No opinion
- ◯ Agree
- ⊗ Strongly agree

e. If you agree with the above, please indicate all the contexts in which you would like to receive this training:

- ☒ As part of my residency.
- ☒ A special program.
- ☒ As part of my fellowship.

SECTION C: Attitudes towards immigrant health

A. Please indicate your level of agreement with the following statements regarding immigrant and refugee health by checking the box that best represents your opinion.

a. I enjoy taking care of immigrants and refugees.

- ◯ Never
- ◯ Rarely
- ◯ Sometimes
- ◯ Usually
- ⊗ Always

b. Please indicate the reasons that you enjoy taking care of immigrants and refugees (may choose more than one).

- ☒ Tropical and other conditions not frequently diagnosed in US-born patients
- ☐ Learning about other cultures
- ☐ They don't complain as much
- ☒ Being able to hear their stories
- ☐ Their care is more complicated
- ☐ Their care is less complicated
- ☒ They are very appreciative of your help.
- ☒ They are extremely vulnerable
- ☐ Other:

c. Taking care of immigrants and refugees is more challenging than taking care of US born patients.

- ◯ Never
- ◯ Rarely
- ⊗ Sometimes
- ◯ Usually
- ◯ Always

d. Please mark all the challenges that you face as a provider when providing care to immigrants and refugees (may choose more than one):

- ☒ Language barriers
- ☐ Insurance barriers
- ☒ Cultural barriers
- ☐ Finding a professional interpreter
- ☐ Knowing how to work with a professional interpreter
- ☒ Time constraints
- ☒ My own knowledge related to tropical and travel medicine
- ☒ Transportation problems for the patient
- ☒ Patients not understanding treatment plan
- ☒ Patients not following treatment plan
- ☐ My lack of knowledge regarding the patient's culture
- ☐ Bias or stereotyping
- ☐ Other:

e. Please mark all of the challenges faced by immigrant and refugee populations when receiving healthcare that you have perceived or witnessed (may choose more than one):

- ☒ Language barriers
- ☒ Insurance barriers
- ☒ Cultural barriers
- ☐ Finding a professional interpreter
- ☐ Knowing how to work with a professional interpreter
- ☒ Time constraints
- ☒ Insufficiently trained health care providers
- ☒ Transportation problems for the patient
- ☒ Food insufficiency
- ☒ Need for child care
- ☒ Patients not understanding treatment plan
- ☒ Patients not following treatment plan
- ☐ My lack of knowledge regarding the patient's culture
- ☐ Bias or stereotyping
- ☒ Trust issues
- ☐ Other...

f. Rank how well immigrants and refugees understand the healthcare that you are trying to provide.

- ◯ Significantly less than a US born individual
- ⊗ Less than a US born individual
- ◯ Equivalent to a US born individual
- ◯ More than a US born patient
- ◯ Significantly more than a US born individual

g. Immigrants and refugees adhere to treatment plans and follow my recommendations.

- ◯ Never
- ◯ Rarely
- ◯ Sometimes
- ⊗ Usually
- ◯ Always

h. Immigrants and refugees should receive the same care and insurance coverage as US born patients.

- ◯ Never
- ◯ Rarely
- ◯ Sometimes
- ◯ Usually
- ⊗ Always

i. Immigrants and refugees who are undocumented should receive the same care and insurance coverage as US born patients.

- ◯ Never
- ◯ Rarely
- ◯ Sometimes
- ⊗ Usually
- ◯ Always

j. Every physician is professionally obligated to care for immigrants and refugees if they present to your clinic or hospital.

- ◯ Strongly disagree
- ◯ Disagree
- ◯ No opinion
- ◯ Agree
- ⊗ Strongly agree

k. Is healthcare a human right?

⊗ Yes
○ No

B. If you wish, please tell us about what you enjoy or do not enjoy about immigrant and refugee health care and the greatest challenges you face in caring for this population.

I enjoy hearing their stories but also being part of it. These folks have gone through so much, and have been victims of both willful and passive neglect. I think giving them another opportunity is incredibly satisfying, but also difficult. I don't want to slight them again, so making sure to be holistic and understanding requires even more energy and knowledge than I'd anticipate in a US born patient.

SECTION D: DEMOGRAPHIC INFORMATION
Please answer the following questions by checking the box in front of the response choice that best describes you.

a. Your age?

○ 20 to 24
⊗ 25 to 29
○ 30 to 34
○ 35 to 39
○ 40 or older

b. Your gender?

○ Female
⊗ Male
○ Other

c. Are you Hispanic or Latino?

○ Yes
⊗ No

d. What is your race? (Select one or more responses)

○ American Indian or Alaska Native
○ Asian (Please specify):
○ Black or African American
○ Native Hawaiian or Other Pacific Islander
⊗ White
○ Other (Please specify):

e. Were you born in the United States?

⊗ Yes
○ No

g. Your residency year?

⊗ PGY1
○ PGY2
○ PGY3
○ PGY4
○ PGY5

h. How would you classify your political ideology?

○ Conservative
○ Somewhat conservative
○ Moderate
⊗ Somewhat liberal
○ Liberal
○ Other (Please specify):

i. Estimated level of educational debt?

○ None
○ Less than $50,000
⊗ $50,000 - $100,000
○ $100,000 - $200,000
○ $200,000 or more

j. Do you plan to subspecialize?

⊗ Yes
○ No

k. Languages spoken?

☒ English
☐ Spanish
☐ French
☐ Hmong
☐ Somali
☐ Japanese
☐ Chinese
☐ Russian
☐ Ethiopian
☒ Other ___________

German

l. Are you in the Global Health Pathway?

⊗ Yes
◯ No

m. Did you earn your degree in the US?

⊗ Yes
◯ No

n. What residency program are you in?

⊗ Internal Medicine
◯ Med-Peds
◯ Pediatrics
◯ Family Practice
◯ Neurology
◯ Psychiatry
◯ ObGyn
◯ Neurosurgery
◯ General Surgery
◯ Orthopedic Surgery
◯ Urology
◯ Surgical sub-specialty (please specify in text box below)
◯ Non-clinical specialty (radiology, pathology; please specify in text box below)

Medical Trainees' attitudes, knowledge, and experience with immigrant and refugee health

Response was added on 05/01/2014 7:55pm.

SECTION A: Personal experience with immigrant and refugee health care.

A. Please indicate your level of agreement with the following statements regarding your personal experience with immigrant and refugee health care by checking the box that best represents your experience.

a. During my inpatient rotations, I take care of the following percentage of immigrant and refugee patients:	◯ None<br>◯ 0 -5%<br>⊗ 10-25%<br>◯ 5-10%<br>◯ > 25%
b. During my outpatient rotations, I take care of the following percentage of immigrant and refugee patients:	◯ None<br>◯ 0-10%<br>⊗ 10 -25%<br>◯ 25-50%<br>◯ 50-75%<br>◯ >75%
c. I would like to take care of more immigrant and refugee patients.	◯ Strongly disagree<br>◯ Disagree<br>⊗ No opinion<br>◯ Agree<br>◯ Strongly agree
d. I plan to take care of immigrants and refugees when I finish residency.	◯ Strongly disagree<br>◯ Disagree<br>⊗ No opinion<br>◯ Agree<br>◯ Strongly agree
e. I plan to do short term (< 6 months) international work when I finish residency.	◯ Strongly disagree<br>◯ Disagree<br>◯ No opinion<br>⊗ Agree<br>◯ Strongly agree
f. I plan to do long term (>6 months) international work when I finish residency.	◯ Strongly disagree<br>◯ Disagree<br>⊗ No opinion<br>◯ Agree<br>◯ Strongly agree
g. I plan to work in health disparities in the following way after residency:	possibly

SECTION B: MEDICAL EDUCATION

A. Please indicate your level of agreement with the following statements regarding your medical education and knowledge about immigrants and refugees by checking the box that best represents your opinion.

a. I have received specialized training in immigrant and refugee health, tropical medicine, or cross-cultural health.

- ◯ Strongly disagree
- ◯ Disagree
- ◯ No opinion
- ⊗ Agree
- ◯ Strongly agree

b. If you have received specialized training in immigrant and refugee health, tropical medicine, or cross-cultural health, please indicate all the contexts in which you received this training:

- ☐ As an undergraduate.
- ☒ As a medical student.
- ☒ As part of my residency.
- ☐ A special program.
- ☐ As part of my fellowship.
- ☐ As part of a degree program (e.g. MPH)
- ☐ Other:

c. I feel comfortable with my fund of knowledge regarding immigrant and refugee health.

- ◯ Strongly disagree
- ◯ Disagree
- ⊗ No opinion
- ◯ Agree
- ◯ Strongly agree

d. I would like to have further training in immigrant and refugee health.

- ◯ Strongly disagree
- ◯ Disagree
- ◯ No opinion
- ⊗ Agree
- ◯ Strongly agree

e. If you agree with the above, please indicate all the contexts in which you would like to receive this training:

- ☒ As part of my residency.
- ☐ A special program.
- ☐ As part of my fellowship.

SECTION C: Attitudes towards immigrant health

A. Please indicate your level of agreement with the following statements regarding immigrant and refugee health by checking the box that best represents your opinion.

a. I enjoy taking care of immigrants and refugees.

- ◯ Never
- ◯ Rarely
- ◯ Sometimes
- ⊗ Usually
- ◯ Always

b. Please indicate the reasons that you enjoy taking care of immigrants and refugees (may choose more than one).

- ☒ Tropical and other conditions not frequently diagnosed in US-born patients
- ☒ Learning about other cultures
- ☐ They don't complain as much
- ☒ Being able to hear their stories
- ☐ Their care is more complicated
- ☐ Their care is less complicated
- ☐ They are very appreciative of your help.
- ☐ They are extremely vulnerable
- ☐ Other:

c. Taking care of immigrants and refugees is more challenging than taking care of US born patients.

- ◯ Never
- ◯ Rarely
- ⊗ Sometimes
- ◯ Usually
- ◯ Always

d. Please mark all the challenges that you face as a provider when providing care to immigrants and refugees (may choose more than one):

- [x] Language barriers
- [ ] Insurance barriers
- [ ] Cultural barriers
- [ ] Finding a professional interpreter
- [ ] Knowing how to work with a professional interpreter
- [x] Time constraints
- [x] My own knowledge related to tropical and travel medicine
- [ ] Transportation problems for the patient
- [x] Patients not understanding treatment plan
- [ ] Patients not following treatment plan
- [ ] My lack of knowledge regarding the patient's culture
- [ ] Bias or stereotyping
- [ ] Other:

e. Please mark all of the challenges faced by immigrant and refugee populations when receiving healthcare that you have perceived or witnessed (may choose more than one):

- [x] Language barriers
- [ ] Insurance barriers
- [x] Cultural barriers
- [ ] Finding a professional interpreter
- [ ] Knowing how to work with a professional interpreter
- [ ] Time constraints
- [ ] Insufficiently trained health care providers
- [ ] Transportation problems for the patient
- [ ] Food insufficiency
- [x] Need for child care
- [x] Patients not understanding treatment plan
- [x] Patients not following treatment plan
- [x] My lack of knowledge regarding the patient's culture
- [ ] Bias or stereotyping
- [x] Trust issues
- [ ] Other...

f. Rank how well immigrants and refugees understand the healthcare that you are trying to provide.

- ○ Significantly less than a US born individual
- ⊗ Less than a US born individual
- ○ Equivalent to a US born individual
- ○ More than a US born patient
- ○ Significantly more than a US born individual

g. Immigrants and refugees adhere to treatment plans and follow my recommendations.

- ○ Never
- ○ Rarely
- ⊗ Sometimes
- ○ Usually
- ○ Always

h. Immigrants and refugees should receive the same care and insurance coverage as US born patients.

- ○ Never
- ○ Rarely
- ○ Sometimes
- ○ Usually
- ⊗ Always

i. Immigrants and refugees who are undocumented should receive the same care and insurance coverage as US born patients.

- ○ Never
- ○ Rarely
- ⊗ Sometimes
- ○ Usually
- ○ Always

j. Every physician is professionally obligated to care for immigrants and refugees if they present to your clinic or hospital.

- ○ Strongly disagree
- ○ Disagree
- ○ No opinion
- ○ Agree
- ⊗ Strongly agree

k. Is healthcare a human right?
- ⊗ Yes
- ◯ No

B. If you wish, please tell us about what you enjoy or do not enjoy about immigrant and refugee health care and the greatest challenges you face in caring for this population.

____

SECTION D: DEMOGRAPHIC INFORMATION

Please answer the following questions by checking the box in front of the response choice that best describes you.

a. Your age?
- ◯ 20 to 24
- ⊗ 25 to 29
- ◯ 30 to 34
- ◯ 35 to 39
- ◯ 40 or older

b. Your gender?
- ⊗ Female
- ◯ Male
- ◯ Other

c. Are you Hispanic or Latino?
- ◯ Yes
- ⊗ No

d. What is your race? (Select one or more responses)
- ◯ American Indian or Alaska Native
- ◯ Asian (Please specify):
- ◯ Black or African American
- ◯ Native Hawaiian or Other Pacific Islander
- ⊗ White
- ◯ Other (Please specify):

e. Were you born in the United States?
- ⊗ Yes
- ◯ No

g. Your residency year?
- ⊗ PGY1
- ◯ PGY2
- ◯ PGY3
- ◯ PGY4
- ◯ PGY5

h. How would you classify your political ideology?
- ◯ Conservative
- ◯ Somewhat conservative
- ⊗ Moderate
- ◯ Somewhat liberal
- ◯ Liberal
- ◯ Other (Please specify):

i. Estimated level of educational debt?
- ◯ None
- ◯ Less than $50,000
- ◯ $50,000 - $100,000
- ◯ $100,000 - $200,000
- ⊗ $200,000 or more

j. Do you plan to subspecialize?
- ⊗ Yes
- ◯ No

k. Languages spoken?

- [x] English
- [ ] Spanish
- [ ] French
- [ ] Hmong
- [ ] Somali
- [ ] Japanese
- [ ] Chinese
- [ ] Russian
- [ ] Ethiopian
- [ ] Other ___________

l. Are you in the Global Health Pathway?

- ◯ Yes
- ⊗ No

m. Did you earn your degree in the US?

- ⊗ Yes
- ◯ No

n. What residency program are you in?

- ⊗ Internal Medicine
- ◯ Med-Peds
- ◯ Pediatrics
- ◯ Family Practice
- ◯ Neurology
- ◯ Psychiatry
- ◯ ObGyn
- ◯ Neurosurgery
- ◯ General Surgery
- ◯ Orthopedic Surgery
- ◯ Urology
- ◯ Surgical sub-specialty (please specify in text box below)
- ◯ Non-clinical specialty (radiology, pathology; please specify in text box below)

Medical Trainees' attitudes, knowledge, and experience with immigrant and refugee health

Response was added on 05/01/2014 8:02pm.

SECTION A: Personal experience with immigrant and refugee health care.

A. Please indicate your level of agreement with the following statements regarding your personal experience with immigrant and refugee health care by checking the box that best represents your experience.

a. During my inpatient rotations, I take care of the following percentage of immigrant and refugee patients:

- ◯ None
- ◯ 0 -5%
- ◯ 5-10%
- ⊗ 10-25%
- ◯ > 25%

b. During my outpatient rotations, I take care of the following percentage of immigrant and refugee patients:

- ◯ None
- ◯ 0-10%
- ⊗ 10 -25%
- ◯ 25-50%
- ◯ 50-75%
- ◯ >75%

c. I would like to take care of more immigrant and refugee patients.

- ◯ Strongly disagree
- ◯ Disagree
- ◯ No opinion
- ⊗ Agree
- ◯ Strongly agree

d. I plan to take care of immigrants and refugees when I finish residency.

- ◯ Strongly disagree
- ◯ Disagree
- ◯ No opinion
- ⊗ Agree
- ◯ Strongly agree

e. I plan to do short term (< 6 months) international work when I finish residency.

- ◯ Strongly disagree
- ⊗ Disagree
- ◯ No opinion
- ◯ Agree
- ◯ Strongly agree

f. I plan to do long term (>6 months) international work when I finish residency.

- ◯ Strongly disagree
- ⊗ Disagree
- ◯ No opinion
- ◯ Agree
- ◯ Strongly agree

g. I plan to work in health disparities in the following way after residency:

Work with disparities among african american and native american population

SECTION B: MEDICAL EDUCATION

A. Please indicate your level of agreement with the following statements regarding your medical education and knowledge about immigrants and refugees by checking the box that best represents your opinion.

a. I have received specialized training in immigrant and refugee health, tropical medicine, or cross-cultural health.

- ◯ Strongly disagree
- ◯ Disagree
- ◯ No opinion
- ⊗ Agree
- ◯ Strongly agree

b. If you have received specialized training in immigrant and refugee health, tropical medicine, or cross-cultural health, please indicate all the contexts in which you received this training:

- ☒ As an undergraduate.
- ☒ As a medical student.
- ☒ As part of my residency.
- ☐ A special program.
- ☐ As part of my fellowship.
- ☐ As part of a degree program (e.g. MPH)
- ☐ Other:

c. I feel comfortable with my fund of knowledge regarding immigrant and refugee health.

- ◯ Strongly disagree
- ◯ Disagree
- ◯ No opinion
- ⊗ Agree
- ◯ Strongly agree

d. I would like to have further training in immigrant and refugee health.

- ◯ Strongly disagree
- ◯ Disagree
- ◯ No opinion
- ◯ Agree
- ◯ Strongly agree

SECTION C: Attitudes towards immigrant health

A.Please indicate your level of agreement with the following statements regarding immigrant and refugee health by checking the box that best represents your opinion.

a. I enjoy taking care of immigrants and refugees.

- ◯ Never
- ◯ Rarely
- ◯ Sometimes
- ⊗ Usually
- ◯ Always

b. Please indicate the reasons that you enjoy taking care of immigrants and refugees (may choose more than one).

- ☐ Tropical and other conditions not frequently diagnosed in US-born patients
- ☒ Learning about other cultures
- ☒ They don't complain as much
- ☒ Being able to hear their stories
- ☐ Their care is more complicated
- ☒ Their care is less complicated
- ☒ They are very appreciative of your help.
- ☐ They are extremely vulnerable
- ☐ Other:

c. Taking care of immigrants and refugees is more challenging than taking care of US born patients.

- ◯ Never
- ◯ Rarely
- ⊗ Sometimes
- ◯ Usually
- ◯ Always

d. Please mark all the challenges that you face as a provider when providing care to immigrants and refugees (may choose more than one):

- ☒ Language barriers
- ☒ Insurance barriers
- ☒ Cultural barriers
- ☒ Finding a professional interpreter
- ☐ Knowing how to work with a professional interpreter
- ☒ Time constraints
- ☒ My own knowledge related to tropical and travel medicine
- ☒ Transportation problems for the patient
- ☒ Patients not understanding treatment plan
- ☒ Patients not following treatment plan
- ☐ My lack of knowledge regarding the patient's culture
- ☐ Bias or stereotyping
- ☐ Other:

e. Please mark all of the challenges faced by immigrant and refugee populations when receiving healthcare that you have perceived or witnessed (may choose more than one):

- ☒ Language barriers
- ☒ Insurance barriers
- ☒ Cultural barriers
- ☒ Finding a professional interpreter
- ☒ Knowing how to work with a professional interpreter
- ☒ Time constraints
- ☒ Insufficiently trained health care providers
- ☒ Transportation problems for the patient
- ☒ Food insufficiency
- ☒ Need for child care
- ☒ Patients not understanding treatment plan
- ☒ Patients not following treatment plan
- ☒ My lack of knowledge regarding the patient's culture
- ☒ Bias or stereotyping
- ☒ Trust issues
- ☐ Other...

f. Rank how well immigrants and refugees understand the healthcare that you are trying to provide.

- ○ Significantly less than a US born individual
- ⊗ Less than a US born individual
- ○ Equivalent to a US born individual
- ○ More than a US born patient
- ○ Significantly more than a US born individual

g. Immigrants and refugees adhere to treatment plans and follow my recommendations.

- ○ Never
- ○ Rarely
- ⊗ Sometimes
- ○ Usually
- ○ Always

h. Immigrants and refugees should receive the same care and insurance coverage as US born patients.

- ○ Never
- ○ Rarely
- ○ Sometimes
- ○ Usually
- ⊗ Always

i. Immigrants and refugees who are undocumented should receive the same care and insurance coverage as US born patients.

- ○ Never
- ○ Rarely
- ⊗ Sometimes
- ○ Usually
- ○ Always

j. Every physician is professionally obligated to care for immigrants and refugees if they present to your clinic or hospital.

- ○ Strongly disagree
- ○ Disagree
- ○ No opinion
- ○ Agree
- ⊗ Strongly agree

k. Is healthcare a human right?

- ⊗ Yes
- ◯ No

B. If you wish, please tell us about what you enjoy or do not enjoy about immigrant and refugee health care and the greatest challenges you face in caring for this population.

____

SECTION D: DEMOGRAPHIC INFORMATION
Please answer the following questions by checking the box in front of the response choice that best describes you.

a. Your age?

- ◯ 20 to 24
- ⊗ 25 to 29
- ◯ 30 to 34
- ◯ 35 to 39
- ◯ 40 or older

b. Your gender?

- ◯ Female
- ⊗ Male
- ◯ Other

c. Are you Hispanic or Latino?

- ◯ Yes
- ⊗ No

d. What is your race? (Select one or more responses)

- ◯ American Indian or Alaska Native
- ◯ Asian (Please specify):
- ◯ Black or African American
- ◯ Native Hawaiian or Other Pacific Islander
- ⊗ White
- ◯ Other (Please specify):

e. Were you born in the United States?

- ⊗ Yes
- ◯ No

g. Your residency year?

- ◯ PGY1
- ◯ PGY2
- ⊗ PGY3
- ◯ PGY4
- ◯ PGY5

h. How would you classify your political ideology?

- ◯ Conservative
- ◯ Somewhat conservative
- ◯ Moderate
- ◯ Somewhat liberal
- ⊗ Liberal
- ◯ Other (Please specify):

i. Estimated level of educational debt?

- ◯ None
- ◯ Less than $50,000
- ◯ $50,000 - $100,000
- ◯ $100,000 - $200,000
- ⊗ $200,000 or more

j. Do you plan to subspecialize?

- ◯ Yes
- ⊗ No

k. Languages spoken?

- [x] English
- [ ] Spanish
- [ ] French
- [ ] Hmong
- [ ] Somali
- [ ] Japanese
- [ ] Chinese
- [ ] Russian
- [ ] Ethiopian
- [ ] Other ___________

l. Are you in the Global Health Pathway?

- ◯ Yes
- ⊗ No

m. Did you earn your degree in the US?

- ⊗ Yes
- ◯ No

n. What residency program are you in?

- ◯ Internal Medicine
- ⊗ Med-Peds
- ◯ Pediatrics
- ◯ Family Practice
- ◯ Neurology
- ◯ Psychiatry
- ◯ ObGyn
- ◯ Neurosurgery
- ◯ General Surgery
- ◯ Orthopedic Surgery
- ◯ Urology
- ◯ Surgical sub-specialty (please specify in text box below)
- ◯ Non-clinical specialty (radiology, pathology; please specify in text box below)

Medical Trainees' attitudes, knowledge, and experience with immigrant and refugee health

Response was added on 05/01/2014 8:08pm.

SECTION A: Personal experience with immigrant and refugee health care.

A. Please indicate your level of agreement with the following statements regarding your personal experience with immigrant and refugee health care by checking the box that best represents your experience.

a. During my inpatient rotations, I take care of the following percentage of immigrant and refugee patients:

- ◯ None
- ◯ 0 -5%
- ⊗ 5-10%
- ◯ 10-25%
- ◯ > 25%

b. During my outpatient rotations, I take care of the following percentage of immigrant and refugee patients:

- ◯ None
- ◯ 0-10%
- ⊗ 10 -25%
- ◯ 25-50%
- ◯ 50-75%
- ◯ >75%

c. I would like to take care of more immigrant and refugee patients.

- ◯ Strongly disagree
- ◯ Disagree
- ⊗ No opinion
- ◯ Agree
- ◯ Strongly agree

d. I plan to take care of immigrants and refugees when I finish residency.

- ◯ Strongly disagree
- ◯ Disagree
- ◯ No opinion
- ⊗ Agree
- ◯ Strongly agree

e. I plan to do short term (< 6 months) international work when I finish residency.

- ◯ Strongly disagree
- ⊗ Disagree
- ◯ No opinion
- ◯ Agree
- ◯ Strongly agree

f. I plan to do long term (>6 months) international work when I finish residency.

- ◯ Strongly disagree
- ⊗ Disagree
- ◯ No opinion
- ◯ Agree
- ◯ Strongly agree

g. I plan to work in health disparities in the following way after residency:

Addiction medicine with a focus on super utilizers. Lots of social inequality feeds into that type of medicine.

SECTION B: MEDICAL EDUCATION

A. Please indicate your level of agreement with the following statements regarding your medical education and knowledge about immigrants and refugees by checking the box that best represents your opinion.

a. I have received specialized training in immigrant and refugee health, tropical medicine, or cross-cultural health.

- ◯ Strongly disagree
- ◯ Disagree
- ◯ No opinion
- ⊗ Agree
- ◯ Strongly agree

b. If you have received specialized training in immigrant and refugee health, tropical medicine, or cross-cultural health, please indicate all the contexts in which you received this training:

- ☐ As an undergraduate.
- ☐ As a medical student.
- ☒ As part of my residency.
- ☐ A special program.
- ☐ As part of my fellowship.
- ☐ As part of a degree program (e.g. MPH)
- ☐ Other:

c. I feel comfortable with my fund of knowledge regarding immigrant and refugee health.

- ◯ Strongly disagree
- ⊗ Disagree
- ◯ No opinion
- ◯ Agree
- ◯ Strongly agree

d. I would like to have further training in immigrant and refugee health.

- ◯ Strongly disagree
- ◯ Disagree
- ◯ No opinion
- ⊗ Agree
- ◯ Strongly agree

e. If you agree with the above, please indicate all the contexts in which you would like to receive this training:
□□

- ☒ As part of my residency.
- ☐ A special program.
- ☐ As part of my fellowship.

SECTION C: Attitudes towards immigrant health

A.□Please indicate your level of agreement with the following statements regarding immigrant and refugee health by checking the box that best represents your opinion.

a. I enjoy taking care of immigrants and refugees.

- ◯ Never
- ◯ Rarely
- ◯ Sometimes
- ⊗ Usually
- ◯ Always

b. Please indicate the reasons that you enjoy taking care of immigrants and refugees (may choose more than one).

- ☐ Tropical and other conditions not frequently diagnosed in US-born patients
- ☒ Learning about other cultures
- ☐ They don't complain as much
- ☒ Being able to hear their stories
- ☒ Their care is more complicated
- ☐ Their care is less complicated
- ☒ They are very appreciative of your help.
- ☒ They are extremely vulnerable
- ☐ Other:

c. Taking care of immigrants and refugees is more challenging than taking care of US born patients.

- ◯ Never
- ◯ Rarely
- ◯ Sometimes
- ⊗ Usually
- ◯ Always

d. Please mark all the challenges that you face as a provider when providing care to immigrants and refugees (may choose more than one):

- ☒ Language barriers
- ☐ Insurance barriers
- ☒ Cultural barriers
- ☐ Finding a professional interpreter
- ☐ Knowing how to work with a professional interpreter
- ☒ Time constraints
- ☒ My own knowledge related to tropical and travel medicine
- ☒ Transportation problems for the patient
- ☒ Patients not understanding treatment plan
- ☒ Patients not following treatment plan
- ☒ My lack of knowledge regarding the patient's culture
- ☒ Bias or stereotyping
- ☐ Other:

e. Please mark all of the challenges faced by immigrant and refugee populations when receiving healthcare that you have perceived or witnessed (may choose more than one):

- ☒ Language barriers
- ☐ Insurance barriers
- ☒ Cultural barriers
- ☒ Finding a professional interpreter
- ☒ Knowing how to work with a professional interpreter
- ☒ Time constraints
- ☐ Insufficiently trained health care providers
- ☒ Transportation problems for the patient
- ☒ Food insufficiency
- ☒ Need for child care
- ☒ Patients not understanding treatment plan
- ☒ Patients not following treatment plan
- ☒ My lack of knowledge regarding the patient's culture
- ☒ Bias or stereotyping
- ☒ Trust issues
- ☐ Other...

f. Rank how well immigrants and refugees understand the healthcare that you are trying to provide.

- ○ Significantly less than a US born individual
- ○ Less than a US born individual
- ⊗ Equivalent to a US born individual
- ○ More than a US born patient
- ○ Significantly more than a US born individual

g. Immigrants and refugees adhere to treatment plans and follow my recommendations.

- ○ Never
- ○ Rarely
- ⊗ Sometimes
- ○ Usually
- ○ Always

h. Immigrants and refugees should receive the same care and insurance coverage as US born patients.

- ○ Never
- ○ Rarely
- ○ Sometimes
- ○ Usually
- ⊗ Always

i. Immigrants and refugees who are undocumented should receive the same care and insurance coverage as US born patients.

- ○ Never
- ○ Rarely
- ○ Sometimes
- ○ Usually
- ⊗ Always

j. Every physician is professionally obligated to care for immigrants and refugees if they present to your clinic or hospital.

- ○ Strongly disagree
- ○ Disagree
- ○ No opinion
- ○ Agree
- ⊗ Strongly agree

k. Is healthcare a human right?

⊗ Yes
◯ No

B. If you wish, please tell us about what you enjoy or do not enjoy about immigrant and refugee health care and the greatest challenges you face in caring for this population.

Side note:
Aa is difficult to answer because it asks me to rate multiple domains of patient care. I have received training in all domains but feel markedly more comfortable with cross cultural care than tropical and travel medicine.

Cf is also hard to answer. The SES, race, and cultural norms of a US born individual vary widely. I have a hard time conceiving of the "average" US born patient.

SECTION D: DEMOGRAPHIC INFORMATION
Please answer the following questions by checking the box in front of the response choice that best describes you.

a. Your age?

◯ 20 to 24
◯ 25 to 29
⊗ 30 to 34
◯ 35 to 39
◯ 40 or older

b. Your gender?

◯ Female
⊗ Male
◯ Other

c. Are you Hispanic or Latino?

◯ Yes
⊗ No

d. What is your race? (Select one or more responses)

◯ American Indian or Alaska Native
◯ Asian (Please specify):
◯ Black or African American
◯ Native Hawaiian or Other Pacific Islander
⊗ White
◯ Other (Please specify):

e. Were you born in the United States?

⊗ Yes
◯ No

g. Your residency year?

◯ PGY1
◯ PGY2
◯ PGY3
⊗ PGY4
◯ PGY5

h. How would you classify your political ideology?

◯ Conservative
◯ Somewhat conservative
◯ Moderate
⊗ Somewhat liberal
◯ Liberal
◯ Other (Please specify):

i. Estimated level of educational debt?

⊗ None
◯ Less than $50,000
◯ $50,000 - $100,000
◯ $100,000 - $200,000
◯ $200,000 or more

j. Do you plan to subspecialize?
- ⊗ Yes
- ◯ No

k. Languages spoken?
- ☒ English
- ☐ Spanish
- ☐ French
- ☐ Hmong
- ☐ Somali
- ☐ Japanese
- ☐ Chinese
- ☐ Russian
- ☐ Ethiopian
- ☐ Other ___________

l. Are you in the Global Health Pathway?
- ◯ Yes
- ⊗ No

m. Did you earn your degree in the US?
- ⊗ Yes
- ◯ No

n. What residency program are you in?
- ◯ Internal Medicine
- ⊗ Med-Peds
- ◯ Pediatrics
- ◯ Family Practice
- ◯ Neurology
- ◯ Psychiatry
- ◯ ObGyn
- ◯ Neurosurgery
- ◯ General Surgery
- ◯ Orthopedic Surgery
- ◯ Urology
- ◯ Surgical sub-specialty (please specify in text box below)
- ◯ Non-clinical specialty (radiology, pathology; please specify in text box below)

Medical Trainees' attitudes, knowledge, and experience with immigrant and refugee health

Response was added on 05/01/2014 8:09pm.

SECTION A: Personal experience with immigrant and refugee health care.

A. Please indicate your level of agreement with the following statements regarding your personal experience with immigrant and refugee health care by checking the box that best represents your experience.

a. During my inpatient rotations, I take care of the following percentage of immigrant and refugee patients:
- ◯ None
- ◯ 0 -5%
- ⊗ 5-10%
- ◯ 10-25%
- ◯ > 25%

b. During my outpatient rotations, I take care of the following percentage of immigrant and refugee patients:
- ◯ None
- ⊗ 0-10%
- ◯ 10 -25%
- ◯ 25-50%
- ◯ 50-75%
- ◯ >75%

c. I would like to take care of more immigrant and refugee patients.
- ◯ Strongly disagree
- ◯ Disagree
- ⊗ No opinion
- ◯ Agree
- ◯ Strongly agree

d. I plan to take care of immigrants and refugees when I finish residency.
- ◯ Strongly disagree
- ◯ Disagree
- ◯ No opinion
- ⊗ Agree
- ◯ Strongly agree

e. I plan to do short term (< 6 months) international work when I finish residency.
- ⊗ Strongly disagree
- ◯ Disagree
- ◯ No opinion
- ◯ Agree
- ◯ Strongly agree

f. I plan to do long term (>6 months) international work when I finish residency.
- ⊗ Strongly disagree
- ◯ Disagree
- ◯ No opinion
- ◯ Agree
- ◯ Strongly agree

g. I plan to work in health disparities in the following way after residency: unsure

SECTION B: MEDICAL EDUCATION

A. Please indicate your level of agreement with the following statements regarding your medical education and knowledge about immigrants and refugees by checking the box that best represents your opinion.

a. I have received specialized training in immigrant and refugee health, tropical medicine, or cross-cultural health.

- ◯ Strongly disagree
- ⊗ Disagree
- ◯ No opinion
- ◯ Agree
- ◯ Strongly agree

c. I feel comfortable with my fund of knowledge regarding immigrant and refugee health.

- ◯ Strongly disagree
- ◯ Disagree
- ⊗ No opinion
- ◯ Agree
- ◯ Strongly agree

d. I would like to have further training in immigrant and refugee health.

- ◯ Strongly disagree
- ◯ Disagree
- ◯ No opinion
- ⊗ Agree
- ◯ Strongly agree

e. If you agree with the above, please indicate all the contexts in which you would like to receive this training:
□□

- ☒ As part of my residency.
- ☒ A special program.
- ☐ As part of my fellowship.

SECTION C: Attitudes towards immigrant health

A.□Please indicate your level of agreement with the following statements regarding immigrant and refugee health by checking the box that best represents your opinion.

a. I enjoy taking care of immigrants and refugees.

- ◯ Never
- ◯ Rarely
- ◯ Sometimes
- ◯ Usually
- ⊗ Always

b. Please indicate the reasons that you enjoy taking care of immigrants and refugees (may choose more than one).

- ☒ Tropical and other conditions not frequently diagnosed in US-born patients
- ☒ Learning about other cultures
- ☐ They don't complain as much
- ☒ Being able to hear their stories
- ☐ Their care is more complicated
- ☐ Their care is less complicated
- ☐ They are very appreciative of your help.
- ☒ They are extremely vulnerable
- ☐ Other:

c. Taking care of immigrants and refugees is more challenging than taking care of US born patients.

- ◯ Never
- ◯ Rarely
- ⊗ Sometimes
- ◯ Usually
- ◯ Always

d. Please mark all the challenges that you face as a provider when providing care to immigrants and refugees (may choose more than one):

- ☒ Language barriers
- ☐ Insurance barriers
- ☒ Cultural barriers
- ☐ Finding a professional interpreter
- ☐ Knowing how to work with a professional interpreter
- ☒ Time constraints
- ☒ My own knowledge related to tropical and travel medicine
- ☐ Transportation problems for the patient
- ☐ Patients not understanding treatment plan
- ☐ Patients not following treatment plan
- ☒ My lack of knowledge regarding the patient's culture
- ☐ Bias or stereotyping
- ☐ Other:

e. Please mark all of the challenges faced by immigrant and refugee populations when receiving healthcare that you have perceived or witnessed (may choose more than one):

- ☒ Language barriers
- ☒ Insurance barriers
- ☒ Cultural barriers
- ☐ Finding a professional interpreter
- ☐ Knowing how to work with a professional interpreter
- ☒ Time constraints
- ☒ Insufficiently trained health care providers
- ☒ Transportation problems for the patient
- ☐ Food insufficiency
- ☐ Need for child care
- ☐ Patients not understanding treatment plan
- ☐ Patients not following treatment plan
- ☒ My lack of knowledge regarding the patient's culture
- ☐ Bias or stereotyping
- ☒ Trust issues
- ☐ Other...

f. Rank how well immigrants and refugees understand the healthcare that you are trying to provide.

- ○ Significantly less than a US born individual
- ⊗ Less than a US born individual
- ○ Equivalent to a US born individual
- ○ More than a US born patient
- ○ Significantly more than a US born individual

g. Immigrants and refugees adhere to treatment plans and follow my recommendations.

- ○ Never
- ○ Rarely
- ○ Sometimes
- ⊗ Usually
- ○ Always

h. Immigrants and refugees should receive the same care and insurance coverage as US born patients.

- ○ Never
- ○ Rarely
- ○ Sometimes
- ○ Usually
- ⊗ Always

i. Immigrants and refugees who are undocumented should receive the same care and insurance coverage as US born patients.

- ○ Never
- ○ Rarely
- ⊗ Sometimes
- ○ Usually
- ○ Always

j. Every physician is professionally obligated to care for immigrants and refugees if they present to your clinic or hospital.

- ○ Strongly disagree
- ○ Disagree
- ○ No opinion
- ○ Agree
- ⊗ Strongly agree

k. Is healthcare a human right?
- ⊗ Yes
- ◯ No

B. If you wish, please tell us about what you enjoy or do not enjoy about immigrant and refugee health care and the greatest challenges you face in caring for this population.

____

SECTION D: DEMOGRAPHIC INFORMATION
Please answer the following questions by checking the box in front of the response choice that best describes you.

a. Your age?
- ◯ 20 to 24
- ◯ 25 to 29
- ⊗ 30 to 34
- ◯ 35 to 39
- ◯ 40 or older

b. Your gender?
- ◯ Female
- ⊗ Male
- ◯ Other

c.Are you Hispanic or Latino?
- ◯ Yes
- ⊗ No

d. What is your race? (Select one or more responses)
- ◯ American Indian or Alaska Native
- ◯ Asian (Please specify):
- ◯ Black or African American
- ◯ Native Hawaiian or Other Pacific Islander
- ⊗ White
- ◯ Other (Please specify):

e.Were you born in the United States?
- ⊗ Yes
- ◯ No

g. Your residency year?
- ◯ PGY1
- ◯ PGY2
- ⊗ PGY3
- ◯ PGY4
- ◯ PGY5

h. How would you classify your political ideology?
- ◯ Conservative
- ◯ Somewhat conservative
- ◯ Moderate
- ⊗ Somewhat liberal
- ◯ Liberal
- ◯ Other (Please specify):

i. Estimated level of educational debt?
- ⊗ None
- ◯ Less than $50,000
- ◯ $50,000 - $100,000
- ◯ $100,000 - $200,000
- ◯ $200,000 or more

j.Do you plan to subspecialize?
- ⊗ Yes
- ◯ No

k. Languages spoken?

- ☒ English
- ☐ Spanish
- ☐ French
- ☐ Hmong
- ☐ Somali
- ☐ Japanese
- ☐ Chinese
- ☐ Russian
- ☐ Ethiopian
- ☐ Other ___________

l. Are you in the Global Health Pathway?

- ○ Yes
- ⊗ No

m. Did you earn your degree in the US?

- ⊗ Yes
- ○ No

n. What residency program are you in?

- ⊗ Internal Medicine
- ○ Med-Peds
- ○ Pediatrics
- ○ Family Practice
- ○ Neurology
- ○ Psychiatry
- ○ ObGyn
- ○ Neurosurgery
- ○ General Surgery
- ○ Orthopedic Surgery
- ○ Urology
- ○ Surgical sub-specialty (please specify in text box below)
- ○ Non-clinical specialty (radiology, pathology; please specify in text box below)

Medical Trainees' attitudes, knowledge, and experience with immigrant and refugee health

Response was added on 05/01/2014 9:34pm.

SECTION A: Personal experience with immigrant and refugee health care.

A. Please indicate your level of agreement with the following statements regarding your personal experience with immigrant and refugee health care by checking the box that best represents your experience.

a. During my inpatient rotations, I take care of the following percentage of immigrant and refugee patients:
- ◯ None
- ◯ 0 -5%
- ⊗ 5-10%
- ◯ 10-25%
- ◯ > 25%

b. During my outpatient rotations, I take care of the following percentage of immigrant and refugee patients:
- ◯ None
- ◯ 0-10%
- ◯ 10 -25%
- ⊗ 25-50%
- ◯ 50-75%
- ◯ >75%

c. I would like to take care of more immigrant and refugee patients.
- ◯ Strongly disagree
- ◯ Disagree
- ⊗ No opinion
- ◯ Agree
- ◯ Strongly agree

d. I plan to take care of immigrants and refugees when I finish residency.
- ◯ Strongly disagree
- ◯ Disagree
- ◯ No opinion
- ◯ Agree
- ⊗ Strongly agree

e. I plan to do short term (< 6 months) international work when I finish residency.
- ◯ Strongly disagree
- ◯ Disagree
- ◯ No opinion
- ◯ Agree
- ⊗ Strongly agree

f. I plan to do long term (>6 months) international work when I finish residency.
- ◯ Strongly disagree
- ◯ Disagree
- ⊗ No opinion
- ◯ Agree
- ◯ Strongly agree

g. I plan to work in health disparities in the following way after residency:

care of patients from underserved communities and research on problems of particular interest in resource-limited settings

SECTION B: MEDICAL EDUCATION

A. Please indicate your level of agreement with the following statements regarding your medical education and knowledge about immigrants and refugees by checking the box that best represents your opinion.

a. I have received specialized training in immigrant and refugee health, tropical medicine, or cross-cultural health.

- ◯ Strongly disagree
- ◯ Disagree
- ◯ No opinion
- ◯ Agree
- ⊗ Strongly agree

b. If you have received specialized training in immigrant and refugee health, tropical medicine, or cross-cultural health, please indicate all the contexts in which you received this training:

- ☐ As an undergraduate.
- ☐ As a medical student.
- ☒ As part of my residency.
- ☐ A special program.
- ☐ As part of my fellowship.
- ☒ As part of a degree program (e.g. MPH)
- ☐ Other:

c. I feel comfortable with my fund of knowledge regarding immigrant and refugee health.

- ◯ Strongly disagree
- ◯ Disagree
- ◯ No opinion
- ⊗ Agree
- ◯ Strongly agree

d. I would like to have further training in immigrant and refugee health.

- ◯ Strongly disagree
- ◯ Disagree
- ◯ No opinion
- ⊗ Agree
- ◯ Strongly agree

e. If you agree with the above, please indicate all the contexts in which you would like to receive this training:

- ☒ As part of my residency.
- ☐ A special program.
- ☒ As part of my fellowship.

SECTION C: Attitudes towards immigrant health

A. Please indicate your level of agreement with the following statements regarding immigrant and refugee health by checking the box that best represents your opinion.

a. I enjoy taking care of immigrants and refugees.

- ◯ Never
- ◯ Rarely
- ◯ Sometimes
- ⊗ Usually
- ◯ Always

b. Please indicate the reasons that you enjoy taking care of immigrants and refugees (may choose more than one).

- ☒ Tropical and other conditions not frequently diagnosed in US-born patients
- ☒ Learning about other cultures
- ☐ They don't complain as much
- ☒ Being able to hear their stories
- ☐ Their care is more complicated
- ☐ Their care is less complicated
- ☒ They are very appreciative of your help.
- ☒ They are extremely vulnerable
- ☐ Other:

c. Taking care of immigrants and refugees is more challenging than taking care of US born patients.

- ◯ Never
- ◯ Rarely
- ⊗ Sometimes
- ◯ Usually
- ◯ Always

d. Please mark all the challenges that you face as a provider when providing care to immigrants and refugees (may choose more than one):

- ☒ Language barriers
- ☐ Insurance barriers
- ☒ Cultural barriers
- ☐ Finding a professional interpreter
- ☐ Knowing how to work with a professional interpreter
- ☒ Time constraints
- ☐ My own knowledge related to tropical and travel medicine
- ☒ Transportation problems for the patient
- ☐ Patients not understanding treatment plan
- ☐ Patients not following treatment plan
- ☐ My lack of knowledge regarding the patient's culture
- ☐ Bias or stereotyping
- ☐ Other:

e. Please mark all of the challenges faced by immigrant and refugee populations when receiving healthcare that you have perceived or witnessed (may choose more than one):

- ☒ Language barriers
- ☒ Insurance barriers
- ☒ Cultural barriers
- ☒ Finding a professional interpreter
- ☒ Knowing how to work with a professional interpreter
- ☐ Time constraints
- ☒ Insufficiently trained health care providers
- ☒ Transportation problems for the patient
- ☒ Food insufficiency
- ☒ Need for child care
- ☒ Patients not understanding treatment plan
- ☐ Patients not following treatment plan
- ☐ My lack of knowledge regarding the patient's culture
- ☒ Bias or stereotyping
- ☒ Trust issues
- ☐ Other...

f. Rank how well immigrants and refugees understand the healthcare that you are trying to provide.

- ◯ Significantly less than a US born individual
- ◯ Less than a US born individual
- ⊗ Equivalent to a US born individual
- ◯ More than a US born patient
- ◯ Significantly more than a US born individual

g. Immigrants and refugees adhere to treatment plans and follow my recommendations.

- ◯ Never
- ◯ Rarely
- ⊗ Sometimes
- ◯ Usually
- ◯ Always

h. Immigrants and refugees should receive the same care and insurance coverage as US born patients.

- ◯ Never
- ◯ Rarely
- ◯ Sometimes
- ◯ Usually
- ⊗ Always

i. Immigrants and refugees who are undocumented should receive the same care and insurance coverage as US born patients.

- ◯ Never
- ◯ Rarely
- ◯ Sometimes
- ◯ Usually
- ⊗ Always

j. Every physician is professionally obligated to care for immigrants and refugees if they present to your clinic or hospital.

- ◯ Strongly disagree
- ◯ Disagree
- ◯ No opinion
- ◯ Agree
- ⊗ Strongly agree

k. Is healthcare a human right?

⊗ Yes
◯ No

B. If you wish, please tell us about what you enjoy or do not enjoy about immigrant and refugee health care and the greatest challenges you face in caring for this population.

____

SECTION D: DEMOGRAPHIC INFORMATION
Please answer the following questions by checking the box in front of the response choice that best describes you.

a. Your age?

◯ 20 to 24
◯ 25 to 29
⊗ 30 to 34
◯ 35 to 39
◯ 40 or older

b. Your gender?

⊗ Female
◯ Male
◯ Other

c. Are you Hispanic or Latino?

◯ Yes
⊗ No

d. What is your race? (Select one or more responses)

◯ American Indian or Alaska Native
◯ Asian (Please specify):
◯ Black or African American
◯ Native Hawaiian or Other Pacific Islander
⊗ White
◯ Other (Please specify):

e. Were you born in the United States?

⊗ Yes
◯ No

g. Your residency year?

◯ PGY1
⊗ PGY2
◯ PGY3
◯ PGY4
◯ PGY5

h. How would you classify your political ideology?

◯ Conservative
◯ Somewhat conservative
◯ Moderate
◯ Somewhat liberal
⊗ Liberal
◯ Other (Please specify):

i. Estimated level of educational debt?

⊗ None
◯ Less than $50,000
◯ $50,000 - $100,000
◯ $100,000 - $200,000
◯ $200,000 or more

j. Do you plan to subspecialize?

⊗ Yes
◯ No

k. Languages spoken?

- ☒ English
- ☐ Spanish
- ☐ French
- ☐ Hmong
- ☐ Somali
- ☐ Japanese
- ☐ Chinese
- ☒ Russian
- ☐ Ethiopian
- ☐ Other ___________

l. Are you in the Global Health Pathway?

- ⊗ Yes
- ◯ No

m. Did you earn your degree in the US?

- ⊗ Yes
- ◯ No

n. What residency program are you in?

- ◯ Internal Medicine
- ⊗ Med-Peds
- ◯ Pediatrics
- ◯ Family Practice
- ◯ Neurology
- ◯ Psychiatry
- ◯ ObGyn
- ◯ Neurosurgery
- ◯ General Surgery
- ◯ Orthopedic Surgery
- ◯ Urology
- ◯ Surgical sub-specialty (please specify in text box below)
- ◯ Non-clinical specialty (radiology, pathology; please specify in text box below)

Medical Trainees' attitudes, knowledge, and experience with immigrant and refugee health

Response was added on 05/01/2014 10:41pm.

SECTION A: Personal experience with immigrant and refugee health care.

A. Please indicate your level of agreement with the following statements regarding your personal experience with immigrant and refugee health care by checking the box that best represents your experience.

a. During my inpatient rotations, I take care of the following percentage of immigrant and refugee patients:	◯ None<br>⊗ 0 -5%<br>◯ 5-10%<br>◯ 10-25%<br>◯ > 25%
b. During my outpatient rotations, I take care of the following percentage of immigrant and refugee patients:	◯ None<br>⊗ 0-10%<br>◯ 10 -25%<br>◯ 25-50%<br>◯ 50-75%<br>◯ >75%
c. I would like to take care of more immigrant and refugee patients.	◯ Strongly disagree<br>◯ Disagree<br>⊗ No opinion<br>◯ Agree<br>◯ Strongly agree
d. I plan to take care of immigrants and refugees when I finish residency.	◯ Strongly disagree<br>◯ Disagree<br>◯ No opinion<br>⊗ Agree<br>◯ Strongly agree
e. I plan to do short term (< 6 months) international work when I finish residency.	◯ Strongly disagree<br>◯ Disagree<br>⊗ No opinion<br>◯ Agree<br>◯ Strongly agree
f. I plan to do long term (>6 months) international work when I finish residency.	⊗ Strongly disagree<br>◯ Disagree<br>◯ No opinion<br>◯ Agree<br>◯ Strongly agree
g. I plan to work in health disparities in the following way after residency:	Not sure

SECTION B: MEDICAL EDUCATION

A. Please indicate your level of agreement with the following statements regarding your medical education and knowledge about immigrants and refugees by checking the box that best represents your opinion.

a. I have received specialized training in immigrant and refugee health, tropical medicine, or cross-cultural health.

◯ Strongly disagree
◯ Disagree
◯ No opinion
⊗ Agree
◯ Strongly agree

b. If you have received specialized training in immigrant and refugee health, tropical medicine, or cross-cultural health, please indicate all the contexts in which you received this training:

☐ As an undergraduate.
☒ As a medical student.
☒ As part of my residency.
☐ A special program.
☐ As part of my fellowship.
☐ As part of a degree program (e.g. MPH)
☐ Other:

c. I feel comfortable with my fund of knowledge regarding immigrant and refugee health.

◯ Strongly disagree
◯ Disagree
⊗ No opinion
◯ Agree
◯ Strongly agree

d. I would like to have further training in immigrant and refugee health.

◯ Strongly disagree
◯ Disagree
⊗ No opinion
◯ Agree
◯ Strongly agree

SECTION C: Attitudes towards immigrant health

A.Please indicate your level of agreement with the following statements regarding immigrant and refugee health by checking the box that best represents your opinion.

a. I enjoy taking care of immigrants and refugees.

◯ Never
◯ Rarely
◯ Sometimes
⊗ Usually
◯ Always

b. Please indicate the reasons that you enjoy taking care of immigrants and refugees (may choose more than one).

☐ Tropical and other conditions not frequently diagnosed in US-born patients
☐ Learning about other cultures
☐ They don't complain as much
☐ Being able to hear their stories
☐ Their care is more complicated
☐ Their care is less complicated
☐ They are very appreciative of your help.
☐ They are extremely vulnerable
☐ Other:

c. Taking care of immigrants and refugees is more challenging than taking care of US born patients.

◯ Never
◯ Rarely
⊗ Sometimes
◯ Usually
◯ Always

d. Please mark all the challenges that you face as a provider when providing care to immigrants and refugees (may choose more than one):

- ☐ Language barriers
- ☐ Insurance barriers
- ☒ Cultural barriers
- ☐ Finding a professional interpreter
- ☐ Knowing how to work with a professional interpreter
- ☒ Time constraints
- ☐ My own knowledge related to tropical and travel medicine
- ☐ Transportation problems for the patient
- ☐ Patients not understanding treatment plan
- ☐ Patients not following treatment plan
- ☐ My lack of knowledge regarding the patient's culture
- ☐ Bias or stereotyping
- ☐ Other:

e. Please mark all of the challenges faced by immigrant and refugee populations when receiving healthcare that you have perceived or witnessed (may choose more than one):

- ☒ Language barriers
- ☒ Insurance barriers
- ☒ Cultural barriers
- ☐ Finding a professional interpreter
- ☐ Knowing how to work with a professional interpreter
- ☐ Time constraints
- ☐ Insufficiently trained health care providers
- ☐ Transportation problems for the patient
- ☐ Food insufficiency
- ☐ Need for child care
- ☐ Patients not understanding treatment plan
- ☐ Patients not following treatment plan
- ☐ My lack of knowledge regarding the patient's culture
- ☐ Bias or stereotyping
- ☐ Trust issues
- ☐ Other...

f. Rank how well immigrants and refugees understand the healthcare that you are trying to provide.

- ○ Significantly less than a US born individual
- ○ Less than a US born individual
- ○ Equivalent to a US born individual
- ⊗ More than a US born patient
- ○ Significantly more than a US born individual

g. Immigrants and refugees adhere to treatment plans and follow my recommendations.

- ○ Never
- ○ Rarely
- ○ Sometimes
- ⊗ Usually
- ○ Always

h. Immigrants and refugees should receive the same care and insurance coverage as US born patients.

- ○ Never
- ○ Rarely
- ○ Sometimes
- ⊗ Usually
- ○ Always

i. Immigrants and refugees who are undocumented should receive the same care and insurance coverage as US born patients.

- ○ Never
- ○ Rarely
- ○ Sometimes
- ⊗ Usually
- ○ Always

j. Every physician is professionally obligated to care for immigrants and refugees if they present to your clinic or hospital.

- ○ Strongly disagree
- ○ Disagree
- ○ No opinion
- ⊗ Agree
- ○ Strongly agree

k. Is healthcare a human right?

- ⊗ Yes
- ◯ No

B. If you wish, please tell us about what you enjoy or do not enjoy about immigrant and refugee health care and the greatest challenges you face in caring for this population. ____

SECTION D: DEMOGRAPHIC INFORMATION
Please answer the following questions by checking the box in front of the response choice that best describes you.

a. Your age?

- ◯ 20 to 24
- ◯ 25 to 29
- ⊗ 30 to 34
- ◯ 35 to 39
- ◯ 40 or older

b. Your gender?

- ◯ Female
- ⊗ Male
- ◯ Other

c. Are you Hispanic or Latino?

- ◯ Yes
- ⊗ No

d. What is your race? (Select one or more responses)

- ◯ American Indian or Alaska Native
- ◯ Asian (Please specify):
- ◯ Black or African American
- ◯ Native Hawaiian or Other Pacific Islander
- ⊗ White
- ◯ Other (Please specify):

e. Were you born in the United States?

- ⊗ Yes
- ◯ No

g. Your residency year?

- ◯ PGY1
- ◯ PGY2
- ⊗ PGY3
- ◯ PGY4
- ◯ PGY5

h. How would you classify your political ideology?

- ◯ Conservative
- ◯ Somewhat conservative
- ⊗ Moderate
- ◯ Somewhat liberal
- ◯ Liberal
- ◯ Other (Please specify):

i. Estimated level of educational debt?

- ◯ None
- ◯ Less than $50,000
- ◯ $50,000 - $100,000
- ⊗ $100,000 - $200,000
- ◯ $200,000 or more

j. Do you plan to subspecialize?

- ⊗ Yes
- ◯ No

k. Languages spoken?

- ☒ English
- ☒ Spanish
- ☐ French
- ☐ Hmong
- ☐ Somali
- ☐ Japanese
- ☐ Chinese
- ☐ Russian
- ☐ Ethiopian
- ☐ Other ___________

l. Are you in the Global Health Pathway?

- ◯ Yes
- ⊗ No

m. Did you earn your degree in the US?

- ⊗ Yes
- ◯ No

n. What residency program are you in?

- ⊗ Internal Medicine
- ◯ Med-Peds
- ◯ Pediatrics
- ◯ Family Practice
- ◯ Neurology
- ◯ Psychiatry
- ◯ ObGyn
- ◯ Neurosurgery
- ◯ General Surgery
- ◯ Orthopedic Surgery
- ◯ Urology
- ◯ Surgical sub-specialty (please specify in text box below)
- ◯ Non-clinical specialty (radiology, pathology; please specify in text box below)

Medical Trainees' attitudes, knowledge, and experience with immigrant and refugee health

Response was added on 05/02/2014 1:10am.

SECTION A: Personal experience with immigrant and refugee health care.

A. Please indicate your level of agreement with the following statements regarding your personal experience with immigrant and refugee health care by checking the box that best represents your experience.

a. During my inpatient rotations, I take care of the following percentage of immigrant and refugee patients:
- ◯ None
- ◯ 0 -5%
- ⊗ 5-10%
- ◯ 10-25%
- ◯ > 25%

b. During my outpatient rotations, I take care of the following percentage of immigrant and refugee patients:
- ◯ None
- ◯ 0-10%
- ◯ 10 -25%
- ⊗ 25-50%
- ◯ 50-75%
- ◯ >75%

c. I would like to take care of more immigrant and refugee patients.
- ◯ Strongly disagree
- ◯ Disagree
- ◯ No opinion
- ⊗ Agree
- ◯ Strongly agree

d. I plan to take care of immigrants and refugees when I finish residency.
- ◯ Strongly disagree
- ◯ Disagree
- ◯ No opinion
- ◯ Agree
- ⊗ Strongly agree

e. I plan to do short term (< 6 months) international work when I finish residency.
- ◯ Strongly disagree
- ◯ Disagree
- ◯ No opinion
- ◯ Agree
- ⊗ Strongly agree

f. I plan to do long term (>6 months) international work when I finish residency.
- ◯ Strongly disagree
- ◯ Disagree
- ⊗ No opinion
- ◯ Agree
- ◯ Strongly agree

g. I plan to work in health disparities in the following way after residency:

Continue to provide high-quality care to refugees and educate colleagues re: appropriate, culturally competent care

SECTION B: MEDICAL EDUCATION

A. Please indicate your level of agreement with the following statements regarding your medical education and knowledge about immigrants and refugees by checking the box that best represents your opinion.

a. I have received specialized training in immigrant and refugee health, tropical medicine, or cross-cultural health.

- ◯ Strongly disagree
- ◯ Disagree
- ◯ No opinion
- ◯ Agree
- ⊗ Strongly agree

b. If you have received specialized training in immigrant and refugee health, tropical medicine, or cross-cultural health, please indicate all the contexts in which you received this training:

- ☐ As an undergraduate.
- ☒ As a medical student.
- ☒ As part of my residency.
- ☒ A special program.
- ☐ As part of my fellowship.
- ☐ As part of a degree program (e.g. MPH)
- ☐ Other:

c. I feel comfortable with my fund of knowledge regarding immigrant and refugee health.

- ◯ Strongly disagree
- ◯ Disagree
- ◯ No opinion
- ⊗ Agree
- ◯ Strongly agree

d. I would like to have further training in immigrant and refugee health.

- ◯ Strongly disagree
- ◯ Disagree
- ◯ No opinion
- ◯ Agree
- ⊗ Strongly agree

e. If you agree with the above, please indicate all the contexts in which you would like to receive this training:

- ☒ As part of my residency.
- ☒ A special program.
- ☐ As part of my fellowship.

SECTION C: Attitudes towards immigrant health

A. Please indicate your level of agreement with the following statements regarding immigrant and refugee health by checking the box that best represents your opinion.

a. I enjoy taking care of immigrants and refugees.

- ◯ Never
- ◯ Rarely
- ◯ Sometimes
- ⊗ Usually
- ◯ Always

b. Please indicate the reasons that you enjoy taking care of immigrants and refugees (may choose more than one).

- ☒ Tropical and other conditions not frequently diagnosed in US-born patients
- ☒ Learning about other cultures
- ☐ They don't complain as much
- ☒ Being able to hear their stories
- ☐ Their care is more complicated
- ☐ Their care is less complicated
- ☒ They are very appreciative of your help.
- ☒ They are extremely vulnerable
- ☐ Other:

c. Taking care of immigrants and refugees is more challenging than taking care of US born patients.

- ◯ Never
- ◯ Rarely
- ◯ Sometimes
- ⊗ Usually
- ◯ Always

d. Please mark all the challenges that you face as a provider when providing care to immigrants and refugees (may choose more than one):

- ☒ Language barriers
- ☒ Insurance barriers
- ☒ Cultural barriers
- ☐ Finding a professional interpreter
- ☐ Knowing how to work with a professional interpreter
- ☐ Time constraints
- ☐ My own knowledge related to tropical and travel medicine
- ☒ Transportation problems for the patient
- ☐ Patients not understanding treatment plan
- ☐ Patients not following treatment plan
- ☒ My lack of knowledge regarding the patient's culture
- ☐ Bias or stereotyping
- ☐ Other:

e. Please mark all of the challenges faced by immigrant and refugee populations when receiving healthcare that you have perceived or witnessed (may choose more than one):

- ☒ Language barriers
- ☒ Insurance barriers
- ☒ Cultural barriers
- ☒ Finding a professional interpreter
- ☒ Knowing how to work with a professional interpreter
- ☒ Time constraints
- ☒ Insufficiently trained health care providers
- ☒ Transportation problems for the patient
- ☒ Food insufficiency
- ☒ Need for child care
- ☒ Patients not understanding treatment plan
- ☐ Patients not following treatment plan
- ☒ My lack of knowledge regarding the patient's culture
- ☒ Bias or stereotyping
- ☒ Trust issues
- ☐ Other...

f. Rank how well immigrants and refugees understand the healthcare that you are trying to provide.

- ○ Significantly less than a US born individual
- ⊗ Less than a US born individual
- ○ Equivalent to a US born individual
- ○ More than a US born patient
- ○ Significantly more than a US born individual

g. Immigrants and refugees adhere to treatment plans and follow my recommendations.

- ○ Never
- ○ Rarely
- ⊗ Sometimes
- ○ Usually
- ○ Always

h. Immigrants and refugees should receive the same care and insurance coverage as US born patients.

- ○ Never
- ○ Rarely
- ○ Sometimes
- ○ Usually
- ⊗ Always

i. Immigrants and refugees who are undocumented should receive the same care and insurance coverage as US born patients.

- ○ Never
- ○ Rarely
- ⊗ Sometimes
- ○ Usually
- ○ Always

j. Every physician is professionally obligated to care for immigrants and refugees if they present to your clinic or hospital.

- ○ Strongly disagree
- ○ Disagree
- ○ No opinion
- ○ Agree
- ⊗ Strongly agree

k. Is healthcare a human right?
- ⊗ Yes
- ◯ No

B. If you wish, please tell us about what you enjoy or do not enjoy about immigrant and refugee health care and the greatest challenges you face in caring for this population.

____

SECTION D: DEMOGRAPHIC INFORMATION
Please answer the following questions by checking the box in front of the response choice that best describes you.

a. Your age?
- ◯ 20 to 24
- ⊗ 25 to 29
- ◯ 30 to 34
- ◯ 35 to 39
- ◯ 40 or older

b. Your gender?
- ⊗ Female
- ◯ Male
- ◯ Other

c.Are you Hispanic or Latino?
- ◯ Yes
- ⊗ No

d. What is your race? (Select one or more responses)
- ◯ American Indian or Alaska Native
- ◯ Asian (Please specify):
- ◯ Black or African American
- ◯ Native Hawaiian or Other Pacific Islander
- ⊗ White
- ◯ Other (Please specify):

e.Were you born in the United States?
- ⊗ Yes
- ◯ No

g. Your residency year?
- ◯ PGY1
- ⊗ PGY2
- ◯ PGY3
- ◯ PGY4
- ◯ PGY5

h. How would you classify your political ideology?
- ◯ Conservative
- ◯ Somewhat conservative
- ⊗ Moderate
- ◯ Somewhat liberal
- ◯ Liberal
- ◯ Other (Please specify):

i. Estimated level of educational debt?
- ◯ None
- ◯ Less than $50,000
- ◯ $50,000 - $100,000
- ⊗ $100,000 - $200,000
- ◯ $200,000 or more

j.Do you plan to subspecialize?
- ⊗ Yes
- ◯ No

k. Languages spoken?

- ☒ English
- ☐ Spanish
- ☐ French
- ☐ Hmong
- ☐ Somali
- ☐ Japanese
- ☐ Chinese
- ☐ Russian
- ☐ Ethiopian
- ☐ Other ___________

l. Are you in the Global Health Pathway?

- ⊗ Yes
- ◯ No

m. Did you earn your degree in the US?

- ⊗ Yes
- ◯ No

n. What residency program are you in?

- ⊗ Internal Medicine
- ◯ Med-Peds
- ◯ Pediatrics
- ◯ Family Practice
- ◯ Neurology
- ◯ Psychiatry
- ◯ ObGyn
- ◯ Neurosurgery
- ◯ General Surgery
- ◯ Orthopedic Surgery
- ◯ Urology
- ◯ Surgical sub-specialty (please specify in text box below)
- ◯ Non-clinical specialty (radiology, pathology; please specify in text box below)

Medical Trainees' attitudes, knowledge, and experience with immigrant and refugee health

Response was added on 05/02/2014 2:26am.

SECTION A: Personal experience with immigrant and refugee health care.

A. Please indicate your level of agreement with the following statements regarding your personal experience with immigrant and refugee health care by checking the box that best represents your experience.

a. During my inpatient rotations, I take care of the following percentage of immigrant and refugee patients:	◯ None<br>◯ 0 -5%<br>⊗ 5-10%<br>◯ 10-25%<br>◯ > 25%
b. During my outpatient rotations, I take care of the following percentage of immigrant and refugee patients:	◯ None<br>◯ 0-10%<br>⊗ 10 -25%<br>◯ 25-50%<br>◯ 50-75%<br>◯ >75%
c. I would like to take care of more immigrant and refugee patients.	◯ Strongly disagree<br>◯ Disagree<br>◯ No opinion<br>⊗ Agree<br>◯ Strongly agree
d. I plan to take care of immigrants and refugees when I finish residency.	◯ Strongly disagree<br>◯ Disagree<br>◯ No opinion<br>⊗ Agree<br>◯ Strongly agree
e. I plan to do short term (< 6 months) international work when I finish residency.	◯ Strongly disagree<br>⊗ Disagree<br>◯ No opinion<br>◯ Agree<br>◯ Strongly agree
f. I plan to do long term (>6 months) international work when I finish residency.	◯ Strongly disagree<br>⊗ Disagree<br>◯ No opinion<br>◯ Agree<br>◯ Strongly agree
g. I plan to work in health disparities in the following way after residency:	n/a

SECTION B: MEDICAL EDUCATION

A. Please indicate your level of agreement with the following statements regarding your medical education and knowledge about immigrants and refugees by checking the box that best represents your opinion.

a. I have received specialized training in immigrant and refugee health, tropical medicine, or cross-cultural health.

- ◯ Strongly disagree
- ◯ Disagree
- ◯ No opinion
- ⊗ Agree
- ◯ Strongly agree

b. If you have received specialized training in immigrant and refugee health, tropical medicine, or cross-cultural health, please indicate all the contexts in which you received this training:

- ☐ As an undergraduate.
- ☐ As a medical student.
- ☐ As part of my residency.
- ☐ A special program.
- ☐ As part of my fellowship.
- ☐ As part of a degree program (e.g. MPH)
- ☒ Other:

____

c. I feel comfortable with my fund of knowledge regarding immigrant and refugee health.

- ◯ Strongly disagree
- ◯ Disagree
- ◯ No opinion
- ⊗ Agree
- ◯ Strongly agree

d. I would like to have further training in immigrant and refugee health.

- ◯ Strongly disagree
- ◯ Disagree
- ◯ No opinion
- ⊗ Agree
- ◯ Strongly agree

e. If you agree with the above, please indicate all the contexts in which you would like to receive this training:
□□

- ☐ As part of my residency.
- ☒ A special program.
- ☐ As part of my fellowship.

SECTION C: Attitudes towards immigrant health

A.□Please indicate your level of agreement with the following statements regarding immigrant and refugee health by checking the box that best represents your opinion.

a. I enjoy taking care of immigrants and refugees.

- ◯ Never
- ◯ Rarely
- ◯ Sometimes
- ◯ Usually
- ⊗ Always

b. Please indicate the reasons that you enjoy taking care of immigrants and refugees (may choose more than one).

- ☒ Tropical and other conditions not frequently diagnosed in US-born patients
- ☒ Learning about other cultures
- ☐ They don't complain as much
- ☐ Being able to hear their stories
- ☒ Their care is more complicated
- ☐ Their care is less complicated
- ☒ They are very appreciative of your help.
- ☐ They are extremely vulnerable
- ☐ Other:

c. Taking care of immigrants and refugees is more challenging than taking care of US born patients.

- ◯ Never
- ◯ Rarely
- ◯ Sometimes
- ⊗ Usually
- ◯ Always

d. Please mark all the challenges that you face as a provider when providing care to immigrants and refugees (may choose more than one):

☒ Language barriers
☒ Insurance barriers
☒ Cultural barriers
☒ Finding a professional interpreter
☒ Knowing how to work with a professional interpreter
☒ Time constraints
☒ My own knowledge related to tropical and travel medicine
☐ Transportation problems for the patient
☒ Patients not understanding treatment plan
☒ Patients not following treatment plan
☒ My lack of knowledge regarding the patient's culture
☒ Bias or stereotyping
☒ Other:

____

e. Please mark all of the challenges faced by immigrant and refugee populations when receiving healthcare that you have perceived or witnessed (may choose more than one):

☒ Language barriers
☒ Insurance barriers
☒ Cultural barriers
☒ Finding a professional interpreter
☒ Knowing how to work with a professional interpreter
☒ Time constraints
☒ Insufficiently trained health care providers
☐ Transportation problems for the patient
☐ Food insufficiency
☐ Need for child care
☒ Patients not understanding treatment plan
☒ Patients not following treatment plan
☒ My lack of knowledge regarding the patient's culture
☒ Bias or stereotyping
☒ Trust issues
☐ Other...

f. Rank how well immigrants and refugees understand the healthcare that you are trying to provide.

○ Significantly less than a US born individual
⊗ Less than a US born individual
○ Equivalent to a US born individual
○ More than a US born patient
○ Significantly more than a US born individual

g. Immigrants and refugees adhere to treatment plans and follow my recommendations.

○ Never
○ Rarely
⊗ Sometimes
○ Usually
○ Always

h. Immigrants and refugees should receive the same care and insurance coverage as US born patients.

○ Never
○ Rarely
○ Sometimes
⊗ Usually
○ Always

i. Immigrants and refugees who are undocumented should receive the same care and insurance coverage as US born patients.

○ Never
○ Rarely
○ Sometimes
⊗ Usually
○ Always

j. Every physician is professionally obligated to care for immigrants and refugees if they present to your clinic or hospital.

- ◯ Strongly disagree
- ◯ Disagree
- ◯ No opinion
- ⊗ Agree
- ◯ Strongly agree

k. Is healthcare a human right?

- ⊗ Yes
- ◯ No

B. If you wish, please tell us about what you enjoy or do not enjoy about immigrant and refugee health care and the greatest challenges you face in caring for this population.

____

SECTION D: DEMOGRAPHIC INFORMATION
Please answer the following questions by checking the box in front of the response choice that best describes you.

a. Your age?

- ◯ 20 to 24
- ◯ 25 to 29
- ◯ 30 to 34
- ⊗ 35 to 39
- ◯ 40 or older

b. Your gender?

- ◯ Female
- ⊗ Male
- ◯ Other

c. Are you Hispanic or Latino?

- ◯ Yes
- ⊗ No

d. What is your race? (Select one or more responses)

- ◯ American Indian or Alaska Native
- ⊗ Asian (Please specify):
- ◯ Black or African American
- ◯ Native Hawaiian or Other Pacific Islander
- ◯ White
- ◯ Other (Please specify):

e. Were you born in the United States?

- ◯ Yes
- ⊗ No

f. If not, in what country were you born?

____

g. Your residency year?

- ⊗ PGY1
- ◯ PGY2
- ◯ PGY3
- ◯ PGY4
- ◯ PGY5

h. How would you classify your political ideology?

- ◯ Conservative
- ◯ Somewhat conservative
- ⊗ Moderate
- ◯ Somewhat liberal
- ◯ Liberal
- ◯ Other (Please specify):

i. Estimated level of educational debt?

- ◯ None
- ⊗ Less than $50,000
- ◯ $50,000 - $100,000
- ◯ $100,000 - $200,000
- ◯ $200,000 or more

j.Do you plan to subspecialize?
- ⊗ Yes
- ○ No

k. Languages spoken?
- ☒ English
- ☐ Spanish
- ☐ French
- ☐ Hmong
- ☐ Somali
- ☐ Japanese
- ☐ Chinese
- ☐ Russian
- ☐ Ethiopian
- ☒ Other ___________

____

l. Are you in the Global Health Pathway?
- ○ Yes
- ⊗ No

m. Did you earn your degree in the US?
- ○ Yes
- ⊗ No

n. What residency program are you in?
- ⊗ Internal Medicine
- ○ Med-Peds
- ○ Pediatrics
- ○ Family Practice
- ○ Neurology
- ○ Psychiatry
- ○ ObGyn
- ○ Neurosurgery
- ○ General Surgery
- ○ Orthopedic Surgery
- ○ Urology
- ○ Surgical sub-specialty (please specify in text box below)
- ○ Non-clinical specialty (radiology, pathology; please specify in text box below)

Medical Trainees' attitudes, knowledge, and experience with immigrant and refugee health

Response was added on 05/02/2014 7:40am.

SECTION A: Personal experience with immigrant and refugee health care.

A. Please indicate your level of agreement with the following statements regarding your personal experience with immigrant and refugee health care by checking the box that best represents your experience.

a. During my inpatient rotations, I take care of the following percentage of immigrant and refugee patients:	◯ None<br>◯ 0 -5%<br>⊗ 5-10%<br>◯ 10-25%<br>◯ > 25%
b. During my outpatient rotations, I take care of the following percentage of immigrant and refugee patients:	◯ None<br>⊗ 0-10%<br>◯ 10 -25%<br>◯ 25-50%<br>◯ 50-75%<br>◯ >75%
c. I would like to take care of more immigrant and refugee patients.	◯ Strongly disagree<br>◯ Disagree<br>⊗ No opinion<br>◯ Agree<br>◯ Strongly agree
d. I plan to take care of immigrants and refugees when I finish residency.	◯ Strongly disagree<br>◯ Disagree<br>⊗ No opinion<br>◯ Agree<br>◯ Strongly agree
e. I plan to do short term (< 6 months) international work when I finish residency.	◯ Strongly disagree<br>◯ Disagree<br>◯ No opinion<br>⊗ Agree<br>◯ Strongly agree
f. I plan to do long term (>6 months) international work when I finish residency.	◯ Strongly disagree<br>⊗ Disagree<br>◯ No opinion<br>◯ Agree<br>◯ Strongly agree
g. I plan to work in health disparities in the following way after residency:	not specifically but if the opportunity arises I will.

SECTION B: MEDICAL EDUCATION

A. Please indicate your level of agreement with the following statements regarding your medical education and knowledge about immigrants and refugees by checking the box that best represents your opinion.

a. I have received specialized training in immigrant and refugee health, tropical medicine, or cross-cultural health.

- ○ Strongly disagree
- ⊗ Disagree
- ○ No opinion
- ○ Agree
- ○ Strongly agree

c. I feel comfortable with my fund of knowledge regarding immigrant and refugee health.

- ○ Strongly disagree
- ⊗ Disagree
- ○ No opinion
- ○ Agree
- ○ Strongly agree

d. I would like to have further training in immigrant and refugee health.

- ○ Strongly disagree
- ○ Disagree
- ○ No opinion
- ⊗ Agree
- ○ Strongly agree

e. If you agree with the above, please indicate all the contexts in which you would like to receive this training:
□□

- ☒ As part of my residency.
- ☐ A special program.
- ☐ As part of my fellowship.

SECTION C: Attitudes towards immigrant health

A.□Please indicate your level of agreement with the following statements regarding immigrant and refugee health by checking the box that best represents your opinion.

a. I enjoy taking care of immigrants and refugees.

- ○ Never
- ○ Rarely
- ○ Sometimes
- ⊗ Usually
- ○ Always

b. Please indicate the reasons that you enjoy taking care of immigrants and refugees (may choose more than one).

- ☒ Tropical and other conditions not frequently diagnosed in US-born patients
- ☒ Learning about other cultures
- ☒ They don't complain as much
- ☐ Being able to hear their stories
- ☒ Their care is more complicated
- ☐ Their care is less complicated
- ☒ They are very appreciative of your help.
- ☐ They are extremely vulnerable
- ☐ Other:

c. Taking care of immigrants and refugees is more challenging than taking care of US born patients.

- ○ Never
- ○ Rarely
- ○ Sometimes
- ⊗ Usually
- ○ Always

d. Please mark all the challenges that you face as a provider when providing care to immigrants and refugees (may choose more than one):

- ☒ Language barriers
- ☐ Insurance barriers
- ☒ Cultural barriers
- ☒ Finding a professional interpreter
- ☐ Knowing how to work with a professional interpreter
- ☒ Time constraints
- ☒ My own knowledge related to tropical and travel medicine
- ☐ Transportation problems for the patient
- ☒ Patients not understanding treatment plan
- ☐ Patients not following treatment plan
- ☒ My lack of knowledge regarding the patient's culture
- ☐ Bias or stereotyping
- ☐ Other:

e. Please mark all of the challenges faced by immigrant and refugee populations when receiving healthcare that you have perceived or witnessed (may choose more than one):

- ☒ Language barriers
- ☒ Insurance barriers
- ☒ Cultural barriers
- ☒ Finding a professional interpreter
- ☐ Knowing how to work with a professional interpreter
- ☐ Time constraints
- ☐ Insufficiently trained health care providers
- ☐ Transportation problems for the patient
- ☐ Food insufficiency
- ☐ Need for child care
- ☒ Patients not understanding treatment plan
- ☐ Patients not following treatment plan
- ☒ My lack of knowledge regarding the patient's culture
- ☐ Bias or stereotyping
- ☒ Trust issues
- ☐ Other...

f. Rank how well immigrants and refugees understand the healthcare that you are trying to provide.

- ○ Significantly less than a US born individual
- ⊗ Less than a US born individual
- ○ Equivalent to a US born individual
- ○ More than a US born patient
- ○ Significantly more than a US born individual

g. Immigrants and refugees adhere to treatment plans and follow my recommendations.

- ○ Never
- ○ Rarely
- ○ Sometimes
- ⊗ Usually
- ○ Always

h. Immigrants and refugees should receive the same care and insurance coverage as US born patients.

- ○ Never
- ○ Rarely
- ○ Sometimes
- ○ Usually
- ⊗ Always

i. Immigrants and refugees who are undocumented should receive the same care and insurance coverage as US born patients.

- ⊗ Never
- ○ Rarely
- ○ Sometimes
- ○ Usually
- ○ Always

j. Every physician is professionally obligated to care for immigrants and refugees if they present to your clinic or hospital.

- ○ Strongly disagree
- ○ Disagree
- ○ No opinion
- ⊗ Agree
- ○ Strongly agree

k. Is healthcare a human right?
◯ Yes
⊗ No

B. If you wish, please tell us about what you enjoy or do not enjoy about immigrant and refugee health care and the greatest challenges you face in caring for this population.

I enjoy taking care of immigrant patients, however the aspect that makes caring for them most difficult is the language barrier. I find it is difficult to use the interpreter phones, or the MARTI, and waiting for an interpreter is often disruptive to rounds.

SECTION D: DEMOGRAPHIC INFORMATION
Please answer the following questions by checking the box in front of the response choice that best describes you.

a. Your age?
◯ 20 to 24
⊗ 25 to 29
◯ 30 to 34
◯ 35 to 39
◯ 40 or older

b. Your gender?
◯ Female
⊗ Male
◯ Other

c. Are you Hispanic or Latino?
◯ Yes
⊗ No

d. What is your race? (Select one or more responses)
◯ American Indian or Alaska Native
◯ Asian (Please specify):
◯ Black or African American
◯ Native Hawaiian or Other Pacific Islander
⊗ White
◯ Other (Please specify):

e. Were you born in the United States?
⊗ Yes
◯ No

g. Your residency year?
⊗ PGY1
◯ PGY2
◯ PGY3
◯ PGY4
◯ PGY5

h. How would you classify your political ideology?
◯ Conservative
⊗ Somewhat conservative
◯ Moderate
◯ Somewhat liberal
◯ Liberal
◯ Other (Please specify):

i. Estimated level of educational debt?
◯ None
◯ Less than $50,000
◯ $50,000 - $100,000
⊗ $100,000 - $200,000
◯ $200,000 or more

j. Do you plan to subspecialize?
⊗ Yes
◯ No

k. Languages spoken?

- ☒ English
- ☒ Spanish
- ☐ French
- ☐ Hmong
- ☐ Somali
- ☐ Japanese
- ☐ Chinese
- ☐ Russian
- ☐ Ethiopian
- ☐ Other ___________

l. Are you in the Global Health Pathway?

- ◯ Yes
- ⊗ No

m. Did you earn your degree in the US?

- ⊗ Yes
- ◯ No

n. What residency program are you in?

- ⊗ Internal Medicine
- ◯ Med-Peds
- ◯ Pediatrics
- ◯ Family Practice
- ◯ Neurology
- ◯ Psychiatry
- ◯ ObGyn
- ◯ Neurosurgery
- ◯ General Surgery
- ◯ Orthopedic Surgery
- ◯ Urology
- ◯ Surgical sub-specialty (please specify in text box below)
- ◯ Non-clinical specialty (radiology, pathology; please specify in text box below)

Medical Trainees' attitudes, knowledge, and experience with immigrant and refugee health

Response was added on 05/02/2014 8:11am.

SECTION A: Personal experience with immigrant and refugee health care.

A. Please indicate your level of agreement with the following statements regarding your personal experience with immigrant and refugee health care by checking the box that best represents your experience.

a. During my inpatient rotations, I take care of the following percentage of immigrant and refugee patients:

- ◯ None
- ◯ 0 -5%
- ⊗ 5-10%
- ◯ 10-25%
- ◯ > 25%

b. During my outpatient rotations, I take care of the following percentage of immigrant and refugee patients:

- ◯ None
- ⊗ 0-10%
- ◯ 10 -25%
- ◯ 25-50%
- ◯ 50-75%
- ◯ >75%

c. I would like to take care of more immigrant and refugee patients.

- ◯ Strongly disagree
- ◯ Disagree
- ◯ No opinion
- ⊗ Agree
- ◯ Strongly agree

d. I plan to take care of immigrants and refugees when I finish residency.

- ◯ Strongly disagree
- ◯ Disagree
- ◯ No opinion
- ⊗ Agree
- ◯ Strongly agree

e. I plan to do short term (< 6 months) international work when I finish residency.

- ◯ Strongly disagree
- ◯ Disagree
- ◯ No opinion
- ⊗ Agree
- ◯ Strongly agree

f. I plan to do long term (>6 months) international work when I finish residency.

- ◯ Strongly disagree
- ◯ Disagree
- ◯ No opinion
- ⊗ Agree
- ◯ Strongly agree

g. I plan to work in health disparities in the following way after residency:

Working with an underserved, under-insured population
Working in underserved international countries

SECTION B: MEDICAL EDUCATION

A. Please indicate your level of agreement with the following statements regarding your medical education and knowledge about immigrants and refugees by checking the box that best represents your opinion.

a. I have received specialized training in immigrant and refugee health, tropical medicine, or cross-cultural health.

- ◯ Strongly disagree
- ⊗ Disagree
- ◯ No opinion
- ◯ Agree
- ◯ Strongly agree

c. I feel comfortable with my fund of knowledge regarding immigrant and refugee health.

- ◯ Strongly disagree
- ⊗ Disagree
- ◯ No opinion
- ◯ Agree
- ◯ Strongly agree

d. I would like to have further training in immigrant and refugee health.

- ◯ Strongly disagree
- ◯ Disagree
- ◯ No opinion
- ⊗ Agree
- ◯ Strongly agree

e. If you agree with the above, please indicate all the contexts in which you would like to receive this training:
□□

- ☒ As part of my residency.
- ☐ A special program.
- ☐ As part of my fellowship.

SECTION C: Attitudes towards immigrant health

A.□Please indicate your level of agreement with the following statements regarding immigrant and refugee health by checking the box that best represents your opinion.

a. I enjoy taking care of immigrants and refugees.

- ◯ Never
- ◯ Rarely
- ◯ Sometimes
- ⊗ Usually
- ◯ Always

b. Please indicate the reasons that you enjoy taking care of immigrants and refugees (may choose more than one).

- ☒ Tropical and other conditions not frequently diagnosed in US-born patients
- ☒ Learning about other cultures
- ☐ They don't complain as much
- ☒ Being able to hear their stories
- ☐ Their care is more complicated
- ☐ Their care is less complicated
- ☒ They are very appreciative of your help.
- ☐ They are extremely vulnerable
- ☐ Other:

c. Taking care of immigrants and refugees is more challenging than taking care of US born patients.

- ◯ Never
- ◯ Rarely
- ⊗ Sometimes
- ◯ Usually
- ◯ Always

d. Please mark all the challenges that you face as a provider when providing care to immigrants and refugees (may choose more than one):

- ☒ Language barriers
- ☒ Insurance barriers
- ☒ Cultural barriers
- ☒ Finding a professional interpreter
- ☐ Knowing how to work with a professional interpreter
- ☐ Time constraints
- ☒ My own knowledge related to tropical and travel medicine
- ☐ Transportation problems for the patient
- ☒ Patients not understanding treatment plan
- ☒ Patients not following treatment plan
- ☒ My lack of knowledge regarding the patient's culture
- ☒ Bias or stereotyping
- ☐ Other:

e. Please mark all of the challenges faced by immigrant and refugee populations when receiving healthcare that you have perceived or witnessed (may choose more than one):

- ☒ Language barriers
- ☒ Insurance barriers
- ☒ Cultural barriers
- ☒ Finding a professional interpreter
- ☒ Knowing how to work with a professional interpreter
- ☐ Time constraints
- ☒ Insufficiently trained health care providers
- ☐ Transportation problems for the patient
- ☐ Food insufficiency
- ☐ Need for child care
- ☒ Patients not understanding treatment plan
- ☒ Patients not following treatment plan
- ☒ My lack of knowledge regarding the patient's culture
- ☒ Bias or stereotyping
- ☒ Trust issues
- ☐ Other...

f. Rank how well immigrants and refugees understand the healthcare that you are trying to provide.

- ○ Significantly less than a US born individual
- ⊗ Less than a US born individual
- ○ Equivalent to a US born individual
- ○ More than a US born patient
- ○ Significantly more than a US born individual

g. Immigrants and refugees adhere to treatment plans and follow my recommendations.

- ○ Never
- ○ Rarely
- ⊗ Sometimes
- ○ Usually
- ○ Always

h. Immigrants and refugees should receive the same care and insurance coverage as US born patients.

- ○ Never
- ○ Rarely
- ○ Sometimes
- ○ Usually
- ⊗ Always

i. Immigrants and refugees who are undocumented should receive the same care and insurance coverage as US born patients.

- ○ Never
- ○ Rarely
- ○ Sometimes
- ○ Usually
- ⊗ Always

j. Every physician is professionally obligated to care for immigrants and refugees if they present to your clinic or hospital.

- ○ Strongly disagree
- ○ Disagree
- ○ No opinion
- ○ Agree
- ⊗ Strongly agree

k. Is healthcare a human right?

⊗ Yes
◯ No

B. If you wish, please tell us about what you enjoy or do not enjoy about immigrant and refugee health care and the greatest challenges you face in caring for this population.

____

SECTION D: DEMOGRAPHIC INFORMATION
Please answer the following questions by checking the box in front of the response choice that best describes you.

a. Your age?

◯ 20 to 24
⊗ 25 to 29
◯ 30 to 34
◯ 35 to 39
◯ 40 or older

b. Your gender?

◯ Female
⊗ Male
◯ Other

c. Are you Hispanic or Latino?

◯ Yes
⊗ No

d. What is your race? (Select one or more responses)

◯ American Indian or Alaska Native
◯ Asian (Please specify):
◯ Black or African American
◯ Native Hawaiian or Other Pacific Islander
⊗ White
◯ Other (Please specify):

e. Were you born in the United States?

⊗ Yes
◯ No

g. Your residency year?

◯ PGY1
⊗ PGY2
◯ PGY3
◯ PGY4
◯ PGY5

h. How would you classify your political ideology?

◯ Conservative
◯ Somewhat conservative
◯ Moderate
⊗ Somewhat liberal
◯ Liberal
◯ Other (Please specify):

i. Estimated level of educational debt?

◯ None
◯ Less than $50,000
◯ $50,000 - $100,000
◯ $100,000 - $200,000
⊗ $200,000 or more

j. Do you plan to subspecialize?

⊗ Yes
◯ No

k. Languages spoken?

- ☒ English
- ☒ Spanish
- ☐ French
- ☐ Hmong
- ☐ Somali
- ☐ Japanese
- ☐ Chinese
- ☐ Russian
- ☐ Ethiopian
- ☐ Other ___________

l. Are you in the Global Health Pathway?

- ◯ Yes
- ⊗ No

m. Did you earn your degree in the US?

- ⊗ Yes
- ◯ No

n. What residency program are you in?

- ⊗ Internal Medicine
- ◯ Med-Peds
- ◯ Pediatrics
- ◯ Family Practice
- ◯ Neurology
- ◯ Psychiatry
- ◯ ObGyn
- ◯ Neurosurgery
- ◯ General Surgery
- ◯ Orthopedic Surgery
- ◯ Urology
- ◯ Surgical sub-specialty (please specify in text box below)
- ◯ Non-clinical specialty (radiology, pathology; please specify in text box below)

Medical Trainees' attitudes, knowledge, and experience with immigrant and refugee health

Response was added on 05/02/2014 12:19pm.

SECTION A: Personal experience with immigrant and refugee health care.

A. Please indicate your level of agreement with the following statements regarding your personal experience with immigrant and refugee health care by checking the box that best represents your experience.

a. During my inpatient rotations, I take care of the following percentage of immigrant and refugee patients:

- ◯ None
- ⊗ 0 -5%
- ◯ 5-10%
- ◯ 10-25%
- ◯ > 25%

b. During my outpatient rotations, I take care of the following percentage of immigrant and refugee patients:

- ⊗ None
- ◯ 0-10%
- ◯ 10 -25%
- ◯ 25-50%
- ◯ 50-75%
- ◯ >75%

c. I would like to take care of more immigrant and refugee patients.

- ◯ Strongly disagree
- ◯ Disagree
- ◯ No opinion
- ⊗ Agree
- ◯ Strongly agree

d. I plan to take care of immigrants and refugees when I finish residency.

- ◯ Strongly disagree
- ◯ Disagree
- ⊗ No opinion
- ◯ Agree
- ◯ Strongly agree

e. I plan to do short term (< 6 months) international work when I finish residency.

- ◯ Strongly disagree
- ◯ Disagree
- ⊗ No opinion
- ◯ Agree
- ◯ Strongly agree

f. I plan to do long term (>6 months) international work when I finish residency.

- ◯ Strongly disagree
- ⊗ Disagree
- ◯ No opinion
- ◯ Agree
- ◯ Strongly agree

g. I plan to work in health disparities in the following way after residency:

Possibly

SECTION B: MEDICAL EDUCATION

A. Please indicate your level of agreement with the following statements regarding your medical education and knowledge about immigrants and refugees by checking the box that best represents your opinion.

a. I have received specialized training in immigrant and refugee health, tropical medicine, or cross-cultural health.

- ◯ Strongly disagree
- ⊗ Disagree
- ◯ No opinion
- ◯ Agree
- ◯ Strongly agree

c. I feel comfortable with my fund of knowledge regarding immigrant and refugee health.

- ◯ Strongly disagree
- ⊗ Disagree
- ◯ No opinion
- ◯ Agree
- ◯ Strongly agree

d. I would like to have further training in immigrant and refugee health.

- ◯ Strongly disagree
- ◯ Disagree
- ◯ No opinion
- ⊗ Agree
- ◯ Strongly agree

e. If you agree with the above, please indicate all the contexts in which you would like to receive this training:
□□

- ☒ As part of my residency.
- ☐ A special program.
- ☐ As part of my fellowship.

SECTION C: Attitudes towards immigrant health

A.□Please indicate your level of agreement with the following statements regarding immigrant and refugee health by checking the box that best represents your opinion.

a. I enjoy taking care of immigrants and refugees.

- ◯ Never
- ◯ Rarely
- ⊗ Sometimes
- ◯ Usually
- ◯ Always

b. Please indicate the reasons that you enjoy taking care of immigrants and refugees (may choose more than one).

- ☒ Tropical and other conditions not frequently diagnosed in US-born patients
- ☒ Learning about other cultures
- ☐ They don't complain as much
- ☐ Being able to hear their stories
- ☐ Their care is more complicated
- ☐ Their care is less complicated
- ☒ They are very appreciative of your help.
- ☐ They are extremely vulnerable
- ☐ Other:

c. Taking care of immigrants and refugees is more challenging than taking care of US born patients.

- ◯ Never
- ⊗ Rarely
- ◯ Sometimes
- ◯ Usually
- ◯ Always

d. Please mark all the challenges that you face as a provider when providing care to immigrants and refugees (may choose more than one):

- ☒ Language barriers
- ☐ Insurance barriers
- ☒ Cultural barriers
- ☒ Finding a professional interpreter
- ☐ Knowing how to work with a professional interpreter
- ☒ Time constraints
- ☒ My own knowledge related to tropical and travel medicine
- ☐ Transportation problems for the patient
- ☐ Patients not understanding treatment plan
- ☐ Patients not following treatment plan
- ☒ My lack of knowledge regarding the patient's culture
- ☐ Bias or stereotyping
- ☐ Other:

e. Please mark all of the challenges faced by immigrant and refugee populations when receiving healthcare that you have perceived or witnessed (may choose more than one):

- ☐ Language barriers
- ☒ Insurance barriers
- ☐ Cultural barriers
- ☒ Finding a professional interpreter
- ☒ Knowing how to work with a professional interpreter
- ☒ Time constraints
- ☐ Insufficiently trained health care providers
- ☒ Transportation problems for the patient
- ☐ Food insufficiency
- ☐ Need for child care
- ☒ Patients not understanding treatment plan
- ☐ Patients not following treatment plan
- ☐ My lack of knowledge regarding the patient's culture
- ☒ Bias or stereotyping
- ☒ Trust issues
- ☐ Other...

f. Rank how well immigrants and refugees understand the healthcare that you are trying to provide.

- ◯ Significantly less than a US born individual
- ◯ Less than a US born individual
- ⊗ Equivalent to a US born individual
- ◯ More than a US born patient
- ◯ Significantly more than a US born individual

g. Immigrants and refugees adhere to treatment plans and follow my recommendations.

- ◯ Never
- ◯ Rarely
- ◯ Sometimes
- ⊗ Usually
- ◯ Always

h. Immigrants and refugees should receive the same care and insurance coverage as US born patients.

- ◯ Never
- ◯ Rarely
- ◯ Sometimes
- ◯ Usually
- ⊗ Always

i. Immigrants and refugees who are undocumented should receive the same care and insurance coverage as US born patients.

- ◯ Never
- ◯ Rarely
- ◯ Sometimes
- ◯ Usually
- ⊗ Always

j. Every physician is professionally obligated to care for immigrants and refugees if they present to your clinic or hospital.

- ◯ Strongly disagree
- ◯ Disagree
- ◯ No opinion
- ◯ Agree
- ⊗ Strongly agree

k. Is healthcare a human right?

⊗ Yes
○ No

B. If you wish, please tell us about what you enjoy or do not enjoy about immigrant and refugee health care and the greatest challenges you face in caring for this population.

In the inpatient setting (and some hospitals do this better than others), getting an interpreter and having family members all coordinated to be there with the interpreter is the most challenging and time consuming part of the process.

SECTION D: DEMOGRAPHIC INFORMATION
Please answer the following questions by checking the box in front of the response choice that best describes you.

a. Your age?

○ 20 to 24
⊗ 25 to 29
○ 30 to 34
○ 35 to 39
○ 40 or older

b. Your gender?

⊗ Female
○ Male
○ Other

c. Are you Hispanic or Latino?

○ Yes
⊗ No

d. What is your race? (Select one or more responses)

○ American Indian or Alaska Native
⊗ Asian (Please specify):
○ Black or African American
○ Native Hawaiian or Other Pacific Islander
○ White
○ Other (Please specify):

e. Were you born in the United States?

○ Yes
⊗ No

f. If not, in what country were you born?

South Korea

g. Your residency year?

⊗ PGY1
○ PGY2
○ PGY3
○ PGY4
○ PGY5

h. How would you classify your political ideology?

○ Conservative
⊗ Somewhat conservative
○ Moderate
○ Somewhat liberal
○ Liberal
○ Other (Please specify):

i. Estimated level of educational debt?

○ None
○ Less than $50,000
○ $50,000 - $100,000
○ $100,000 - $200,000
⊗ $200,000 or more

j. Do you plan to subspecialize?

⊗ Yes
○ No

k. Languages spoken?

- ☒ English
- ☐ Spanish
- ☐ French
- ☐ Hmong
- ☐ Somali
- ☐ Japanese
- ☐ Chinese
- ☐ Russian
- ☐ Ethiopian
- ☐ Other ___________

l. Are you in the Global Health Pathway?

- ◯ Yes
- ⊗ No

m. Did you earn your degree in the US?

- ◯ Yes
- ⊗ No

n. What residency program are you in?

- ⊗ Internal Medicine
- ◯ Med-Peds
- ◯ Pediatrics
- ◯ Family Practice
- ◯ Neurology
- ◯ Psychiatry
- ◯ ObGyn
- ◯ Neurosurgery
- ◯ General Surgery
- ◯ Orthopedic Surgery
- ◯ Urology
- ◯ Surgical sub-specialty (please specify in text box below)
- ◯ Non-clinical specialty (radiology, pathology; please specify in text box below)

Medical Trainees' attitudes, knowledge, and experience with immigrant and refugee health

Response was added on 05/02/2014 3:25pm.

SECTION A: Personal experience with immigrant and refugee health care.

A. Please indicate your level of agreement with the following statements regarding your personal experience with immigrant and refugee health care by checking the box that best represents your experience.

a. During my inpatient rotations, I take care of the following percentage of immigrant and refugee patients:

- ◯ None
- ◯ 0 -5%
- ◯ 5-10%
- ⊗ 10-25%
- ◯ > 25%

b. During my outpatient rotations, I take care of the following percentage of immigrant and refugee patients:

- ◯ None
- ◯ 0-10%
- ◯ 10 -25%
- ◯ 25-50%
- ⊗ 50-75%
- ◯ >75%

c. I would like to take care of more immigrant and refugee patients.

- ◯ Strongly disagree
- ◯ Disagree
- ◯ No opinion
- ⊗ Agree
- ◯ Strongly agree

d. I plan to take care of immigrants and refugees when I finish residency.

- ◯ Strongly disagree
- ◯ Disagree
- ◯ No opinion
- ⊗ Agree
- ◯ Strongly agree

e. I plan to do short term (< 6 months) international work when I finish residency.

- ◯ Strongly disagree
- ◯ Disagree
- ◯ No opinion
- ◯ Agree
- ⊗ Strongly agree

f. I plan to do long term (>6 months) international work when I finish residency.

- ◯ Strongly disagree
- ◯ Disagree
- ⊗ No opinion
- ◯ Agree
- ◯ Strongly agree

g. I plan to work in health disparities in the following way after residency:

Work in urban underserved areas focusing on community-based initiatives domestically and abroad working in low-resource settings striving for improving infrastructure and accessibility.

SECTION B: MEDICAL EDUCATION

A. Please indicate your level of agreement with the following statements regarding your medical education and knowledge about immigrants and refugees by checking the box that best represents your opinion.

a. I have received specialized training in immigrant and refugee health, tropical medicine, or cross-cultural health.

- ◯ Strongly disagree
- ◯ Disagree
- ◯ No opinion
- ⊗ Agree
- ◯ Strongly agree

b. If you have received specialized training in immigrant and refugee health, tropical medicine, or cross-cultural health, please indicate all the contexts in which you received this training:

- ☐ As an undergraduate.
- ☐ As a medical student.
- ☒ As part of my residency.
- ☐ A special program.
- ☐ As part of my fellowship.
- ☒ As part of a degree program (e.g. MPH)
- ☐ Other:

c. I feel comfortable with my fund of knowledge regarding immigrant and refugee health.

- ◯ Strongly disagree
- ⊗ Disagree
- ◯ No opinion
- ◯ Agree
- ◯ Strongly agree

d. I would like to have further training in immigrant and refugee health.

- ◯ Strongly disagree
- ◯ Disagree
- ◯ No opinion
- ◯ Agree
- ⊗ Strongly agree

e. If you agree with the above, please indicate all the contexts in which you would like to receive this training:
□□

- ☒ As part of my residency.
- ☒ A special program.
- ☐ As part of my fellowship.

SECTION C: Attitudes towards immigrant health

A.□Please indicate your level of agreement with the following statements regarding immigrant and refugee health by checking the box that best represents your opinion.

a. I enjoy taking care of immigrants and refugees.

- ◯ Never
- ◯ Rarely
- ◯ Sometimes
- ◯ Usually
- ⊗ Always

b. Please indicate the reasons that you enjoy taking care of immigrants and refugees (may choose more than one).

- ☒ Tropical and other conditions not frequently diagnosed in US-born patients
- ☒ Learning about other cultures
- ☐ They don't complain as much
- ☒ Being able to hear their stories
- ☒ Their care is more complicated
- ☐ Their care is less complicated
- ☒ They are very appreciative of your help.
- ☒ They are extremely vulnerable
- ☐ Other:

c. Taking care of immigrants and refugees is more challenging than taking care of US born patients.

- ◯ Never
- ◯ Rarely
- ⊗ Sometimes
- ◯ Usually
- ◯ Always

d. Please mark all the challenges that you face as a provider when providing care to immigrants and refugees (may choose more than one):

- ☒ Language barriers
- ☒ Insurance barriers
- ☒ Cultural barriers
- ☒ Finding a professional interpreter
- ☐ Knowing how to work with a professional interpreter
- ☒ Time constraints
- ☒ My own knowledge related to tropical and travel medicine
- ☒ Transportation problems for the patient
- ☒ Patients not understanding treatment plan
- ☒ Patients not following treatment plan
- ☒ My lack of knowledge regarding the patient's culture
- ☒ Bias or stereotyping
- ☐ Other:

e. Please mark all of the challenges faced by immigrant and refugee populations when receiving healthcare that you have perceived or witnessed (may choose more than one):

- ☒ Language barriers
- ☒ Insurance barriers
- ☒ Cultural barriers
- ☒ Finding a professional interpreter
- ☒ Knowing how to work with a professional interpreter
- ☒ Time constraints
- ☒ Insufficiently trained health care providers
- ☒ Transportation problems for the patient
- ☒ Food insufficiency
- ☒ Need for child care
- ☒ Patients not understanding treatment plan
- ☒ Patients not following treatment plan
- ☒ My lack of knowledge regarding the patient's culture
- ☒ Bias or stereotyping
- ☒ Trust issues
- ☐ Other...

f. Rank how well immigrants and refugees understand the healthcare that you are trying to provide.

- ◯ Significantly less than a US born individual
- ⊗ Less than a US born individual
- ◯ Equivalent to a US born individual
- ◯ More than a US born patient
- ◯ Significantly more than a US born individual

g. Immigrants and refugees adhere to treatment plans and follow my recommendations.

- ◯ Never
- ◯ Rarely
- ⊗ Sometimes
- ◯ Usually
- ◯ Always

h. Immigrants and refugees should receive the same care and insurance coverage as US born patients.

- ◯ Never
- ◯ Rarely
- ◯ Sometimes
- ◯ Usually
- ⊗ Always

i. Immigrants and refugees who are undocumented should receive the same care and insurance coverage as US born patients.

- ◯ Never
- ◯ Rarely
- ◯ Sometimes
- ◯ Usually
- ⊗ Always

j. Every physician is professionally obligated to care for immigrants and refugees if they present to your clinic or hospital.

- ◯ Strongly disagree
- ◯ Disagree
- ◯ No opinion
- ◯ Agree
- ⊗ Strongly agree

k. Is healthcare a human right?

- ⊗ Yes
- ◯ No

B. If you wish, please tell us about what you enjoy or do not enjoy about immigrant and refugee health care and the greatest challenges you face in caring for this population.

____

SECTION D: DEMOGRAPHIC INFORMATION
Please answer the following questions by checking the box in front of the response choice that best describes you.

a. Your age?

- ◯ 20 to 24
- ⊗ 25 to 29
- ◯ 30 to 34
- ◯ 35 to 39
- ◯ 40 or older

b. Your gender?

- ◯ Female
- ⊗ Male
- ◯ Other

c. Are you Hispanic or Latino?

- ◯ Yes
- ⊗ No

d. What is your race? (Select one or more responses)

- ◯ American Indian or Alaska Native
- ◯ Asian (Please specify):
- ◯ Black or African American
- ◯ Native Hawaiian or Other Pacific Islander
- ◯ White
- ⊗ Other (Please specify):

South Asian

e. Were you born in the United States?

- ◯ Yes
- ⊗ No

f. If not, in what country were you born?

Ireland

g. Your residency year?

- ◯ PGY1
- ⊗ PGY2
- ◯ PGY3
- ◯ PGY4
- ◯ PGY5

h. How would you classify your political ideology?

- ◯ Conservative
- ◯ Somewhat conservative
- ◯ Moderate
- ◯ Somewhat liberal
- ⊗ Liberal
- ◯ Other (Please specify):

i. Estimated level of educational debt?

- ◯ None
- ◯ Less than $50,000
- ◯ $50,000 - $100,000
- ⊗ $100,000 - $200,000
- ◯ $200,000 or more

j.Do you plan to subspecialize?

- ◯ Yes
- ⊗ No

k. Languages spoken?

- ☒ English
- ☒ Spanish
- ☐ French
- ☐ Hmong
- ☐ Somali
- ☐ Japanese
- ☐ Chinese
- ☐ Russian
- ☐ Ethiopian
- ☐ Other ___________

l. Are you in the Global Health Pathway?

- ⊗ Yes
- ◯ No

m. Did you earn your degree in the US?

- ⊗ Yes
- ◯ No

n. What residency program are you in?

- ◯ Internal Medicine
- ⊗ Med-Peds
- ◯ Pediatrics
- ◯ Family Practice
- ◯ Neurology
- ◯ Psychiatry
- ◯ ObGyn
- ◯ Neurosurgery
- ◯ General Surgery
- ◯ Orthopedic Surgery
- ◯ Urology
- ◯ Surgical sub-specialty (please specify in text box below)
- ◯ Non-clinical specialty (radiology, pathology; please specify in text box below)

Medical Trainees' attitudes, knowledge, and experience with immigrant and refugee health

Response was added on 05/03/2014 8:41am.

SECTION A: Personal experience with immigrant and refugee health care.

A. Please indicate your level of agreement with the following statements regarding your personal experience with immigrant and refugee health care by checking the box that best represents your experience.

a. During my inpatient rotations, I take care of the following percentage of immigrant and refugee patients:

- ◯ None
- ◯ 0 -5%
- ⊗ 5-10%
- ◯ 10-25%
- ◯ > 25%

b. During my outpatient rotations, I take care of the following percentage of immigrant and refugee patients:

- ◯ None
- ⊗ 0-10%
- ◯ 10 -25%
- ◯ 25-50%
- ◯ 50-75%
- ◯ >75%

c. I would like to take care of more immigrant and refugee patients.

- ◯ Strongly disagree
- ◯ Disagree
- ⊗ No opinion
- ◯ Agree
- ◯ Strongly agree

d. I plan to take care of immigrants and refugees when I finish residency.

- ◯ Strongly disagree
- ◯ Disagree
- ◯ No opinion
- ⊗ Agree
- ◯ Strongly agree

e. I plan to do short term (< 6 months) international work when I finish residency.

- ◯ Strongly disagree
- ⊗ Disagree
- ◯ No opinion
- ◯ Agree
- ◯ Strongly agree

f. I plan to do long term (>6 months) international work when I finish residency.

- ⊗ Strongly disagree
- ◯ Disagree
- ◯ No opinion
- ◯ Agree
- ◯ Strongly agree

g. I plan to work in health disparities in the following way after residency:

safety net hospital

SECTION B: MEDICAL EDUCATION

A. Please indicate your level of agreement with the following statements regarding your medical education and knowledge about immigrants and refugees by checking the box that best represents your opinion.

a. I have received specialized training in immigrant and refugee health, tropical medicine, or cross-cultural health.

- ◯ Strongly disagree
- ⊗ Disagree
- ◯ No opinion
- ◯ Agree
- ◯ Strongly agree

c. I feel comfortable with my fund of knowledge regarding immigrant and refugee health.

- ◯ Strongly disagree
- ⊗ Disagree
- ◯ No opinion
- ◯ Agree
- ◯ Strongly agree

d. I would like to have further training in immigrant and refugee health.

- ◯ Strongly disagree
- ⊗ Disagree
- ◯ No opinion
- ◯ Agree
- ◯ Strongly agree

SECTION C: Attitudes towards immigrant health

A.Please indicate your level of agreement with the following statements regarding immigrant and refugee health by checking the box that best represents your opinion.

a. I enjoy taking care of immigrants and refugees.

- ◯ Never
- ◯ Rarely
- ◯ Sometimes
- ⊗ Usually
- ◯ Always

b. Please indicate the reasons that you enjoy taking care of immigrants and refugees (may choose more than one).

- ☒ Tropical and other conditions not frequently diagnosed in US-born patients
- ☒ Learning about other cultures
- ☐ They don't complain as much
- ☐ Being able to hear their stories
- ☐ Their care is more complicated
- ☐ Their care is less complicated
- ☒ They are very appreciative of your help.
- ☐ They are extremely vulnerable
- ☐ Other:

c. Taking care of immigrants and refugees is more challenging than taking care of US born patients.

- ◯ Never
- ◯ Rarely
- ⊗ Sometimes
- ◯ Usually
- ◯ Always

d. Please mark all the challenges that you face as a provider when providing care to immigrants and refugees (may choose more than one):

- ☒ Language barriers
- ☐ Insurance barriers
- ☒ Cultural barriers
- ☐ Finding a professional interpreter
- ☐ Knowing how to work with a professional interpreter
- ☐ Time constraints
- ☒ My own knowledge related to tropical and travel medicine
- ☐ Transportation problems for the patient
- ☐ Patients not understanding treatment plan
- ☐ Patients not following treatment plan
- ☒ My lack of knowledge regarding the patient's culture
- ☐ Bias or stereotyping
- ☐ Other:

e. Please mark all of the challenges faced by immigrant and refugee populations when receiving healthcare that you have perceived or witnessed (may choose more than one):

- ☐ Language barriers
- ☒ Insurance barriers
- ☒ Cultural barriers
- ☐ Finding a professional interpreter
- ☐ Knowing how to work with a professional interpreter
- ☐ Time constraints
- ☐ Insufficiently trained health care providers
- ☐ Transportation problems for the patient
- ☐ Food insufficiency
- ☐ Need for child care
- ☒ Patients not understanding treatment plan
- ☒ Patients not following treatment plan
- ☐ My lack of knowledge regarding the patient's culture
- ☐ Bias or stereotyping
- ☐ Trust issues
- ☐ Other...

f. Rank how well immigrants and refugees understand the healthcare that you are trying to provide.

- ◯ Significantly less than a US born individual
- ⊗ Less than a US born individual
- ◯ Equivalent to a US born individual
- ◯ More than a US born patient
- ◯ Significantly more than a US born individual

g. Immigrants and refugees adhere to treatment plans and follow my recommendations.

- ◯ Never
- ◯ Rarely
- ◯ Sometimes
- ⊗ Usually
- ◯ Always

h. Immigrants and refugees should receive the same care and insurance coverage as US born patients.

- ◯ Never
- ◯ Rarely
- ◯ Sometimes
- ◯ Usually
- ⊗ Always

i. Immigrants and refugees who are undocumented should receive the same care and insurance coverage as US born patients.

- ◯ Never
- ◯ Rarely
- ◯ Sometimes
- ⊗ Usually
- ◯ Always

j. Every physician is professionally obligated to care for immigrants and refugees if they present to your clinic or hospital.

- ◯ Strongly disagree
- ◯ Disagree
- ◯ No opinion
- ⊗ Agree
- ◯ Strongly agree

k. Is healthcare a human right?

- ⊗ Yes
- ◯ No

B. If you wish, please tell us about what you enjoy or do not enjoy about immigrant and refugee health care and the greatest challenges you face in caring for this population.

____

SECTION D: DEMOGRAPHIC INFORMATION
Please answer the following questions by checking the box in front of the response choice that best describes you.

a. Your age?

- ◯ 20 to 24
- ⊗ 25 to 29
- ◯ 30 to 34
- ◯ 35 to 39
- ◯ 40 or older

b. Your gender?

- ⊗ Female
- ◯ Male
- ◯ Other

c. Are you Hispanic or Latino?

- ◯ Yes
- ⊗ No

d. What is your race? (Select one or more responses)

- ◯ American Indian or Alaska Native
- ◯ Asian (Please specify):
- ◯ Black or African American
- ◯ Native Hawaiian or Other Pacific Islander
- ⊗ White
- ◯ Other (Please specify):

e. Were you born in the United States?

- ⊗ Yes
- ◯ No

g. Your residency year?

- ◯ PGY1
- ◯ PGY2
- ⊗ PGY3
- ◯ PGY4
- ◯ PGY5

h. How would you classify your political ideology?

- ◯ Conservative
- ◯ Somewhat conservative
- ◯ Moderate
- ⊗ Somewhat liberal
- ◯ Liberal
- ◯ Other (Please specify):

i. Estimated level of educational debt?

- ◯ None
- ⊗ Less than $50,000
- ◯ $50,000 - $100,000
- ◯ $100,000 - $200,000
- ◯ $200,000 or more

j. Do you plan to subspecialize?

- ⊗ Yes
- ◯ No

k. Languages spoken?

- ☒ English
- ☐ Spanish
- ☐ French
- ☐ Hmong
- ☐ Somali
- ☐ Japanese
- ☐ Chinese
- ☐ Russian
- ☐ Ethiopian
- ☐ Other ___________

l. Are you in the Global Health Pathway?

- ◯ Yes
- ⊗ No

m. Did you earn your degree in the US?

- ⊗ Yes
- ○ No

n. What residency program are you in?

- ⊗ Internal Medicine
- ○ Med-Peds
- ○ Pediatrics
- ○ Family Practice
- ○ Neurology
- ○ Psychiatry
- ○ ObGyn
- ○ Neurosurgery
- ○ General Surgery
- ○ Orthopedic Surgery
- ○ Urology
- ○ Surgical sub-specialty (please specify in text box below)
- ○ Non-clinical specialty (radiology, pathology; please specify in text box below)

Medical Trainees' attitudes, knowledge, and experience with immigrant and refugee health

Response was added on 05/03/2014 4:37pm.

SECTION A: Personal experience with immigrant and refugee health care.

A. Please indicate your level of agreement with the following statements regarding your personal experience with immigrant and refugee health care by checking the box that best represents your experience.

a. During my inpatient rotations, I take care of the following percentage of immigrant and refugee patients:

- ◯ None
- ◯ 0 -5%
- ⊗ 5-10%
- ◯ 10-25%
- ◯ > 25%

b. During my outpatient rotations, I take care of the following percentage of immigrant and refugee patients:

- ⊗ None
- ◯ 0-10%
- ◯ 10 -25%
- ◯ 25-50%
- ◯ 50-75%
- ◯ >75%

c. I would like to take care of more immigrant and refugee patients.

- ◯ Strongly disagree
- ⊗ Disagree
- ◯ No opinion
- ◯ Agree
- ◯ Strongly agree

d. I plan to take care of immigrants and refugees when I finish residency.

- ⊗ Strongly disagree
- ◯ Disagree
- ◯ No opinion
- ◯ Agree
- ◯ Strongly agree

e. I plan to do short term (< 6 months) international work when I finish residency.

- ⊗ Strongly disagree
- ◯ Disagree
- ◯ No opinion
- ◯ Agree
- ◯ Strongly agree

f. I plan to do long term (>6 months) international work when I finish residency.

- ⊗ Strongly disagree
- ◯ Disagree
- ◯ No opinion
- ◯ Agree
- ◯ Strongly agree

g. I plan to work in health disparities in the following way after residency:

undecided

SECTION B: MEDICAL EDUCATION

A. Please indicate your level of agreement with the following statements regarding your medical education and knowledge about immigrants and refugees by checking the box that best represents your opinion.

a. I have received specialized training in immigrant and refugee health, tropical medicine, or cross-cultural health.

- ◯ Strongly disagree
- ⊗ Disagree
- ⊗ No opinion
- ◯ Agree
- ◯ Strongly agree

c. I feel comfortable with my fund of knowledge regarding immigrant and refugee health.

- ◯ Strongly disagree
- ◯ Disagree
- ⊗ No opinion
- ◯ Agree
- ◯ Strongly agree

d. I would like to have further training in immigrant and refugee health.

- ◯ Strongly disagree
- ⊗ Disagree
- ◯ No opinion
- ◯ Agree
- ◯ Strongly agree

SECTION C: Attitudes towards immigrant health

A.Please indicate your level of agreement with the following statements regarding immigrant and refugee health by checking the box that best represents your opinion.

a. I enjoy taking care of immigrants and refugees.

- ◯ Never
- ⊗ Rarely
- ◯ Sometimes
- ◯ Usually
- ◯ Always

b. Please indicate the reasons that you enjoy taking care of immigrants and refugees (may choose more than one).

- ☐ Tropical and other conditions not frequently diagnosed in US-born patients
- ☐ Learning about other cultures
- ☐ They don't complain as much
- ☐ Being able to hear their stories
- ☐ Their care is more complicated
- ☐ Their care is less complicated
- ☐ They are very appreciative of your help.
- ☐ They are extremely vulnerable
- ☐ Other:

c. Taking care of immigrants and refugees is more challenging than taking care of US born patients.

- ◯ Never
- ◯ Rarely
- ◯ Sometimes
- ⊗ Usually
- ◯ Always

d. Please mark all the challenges that you face as a provider when providing care to immigrants and refugees (may choose more than one):

- ☒ Language barriers
- ☐ Insurance barriers
- ☒ Cultural barriers
- ☒ Finding a professional interpreter
- ☐ Knowing how to work with a professional interpreter
- ☒ Time constraints
- ☒ My own knowledge related to tropical and travel medicine
- ☒ Transportation problems for the patient
- ☒ Patients not understanding treatment plan
- ☒ Patients not following treatment plan
- ☒ My lack of knowledge regarding the patient's culture
- ☐ Bias or stereotyping
- ☐ Other:

e. Please mark all of the challenges faced by immigrant and refugee populations when receiving healthcare that you have perceived or witnessed (may choose more than one):

- ☒ Language barriers
- ☒ Insurance barriers
- ☒ Cultural barriers
- ☒ Finding a professional interpreter
- ☐ Knowing how to work with a professional interpreter
- ☐ Time constraints
- ☒ Insufficiently trained health care providers
- ☒ Transportation problems for the patient
- ☐ Food insufficiency
- ☐ Need for child care
- ☐ Patients not understanding treatment plan
- ☐ Patients not following treatment plan
- ☐ My lack of knowledge regarding the patient's culture
- ☐ Bias or stereotyping
- ☐ Trust issues
- ☐ Other...

f. Rank how well immigrants and refugees understand the healthcare that you are trying to provide.

- ⊗ Significantly less than a US born individual
- ◯ Less than a US born individual
- ◯ Equivalent to a US born individual
- ◯ More than a US born patient
- ◯ Significantly more than a US born individual

g. Immigrants and refugees adhere to treatment plans and follow my recommendations.

- ◯ Never
- ◯ Rarely
- ◯ Sometimes
- ⊗ Usually
- ◯ Always

h. Immigrants and refugees should receive the same care and insurance coverage as US born patients.

- ◯ Never
- ◯ Rarely
- ◯ Sometimes
- ⊗ Usually
- ◯ Always

i. Immigrants and refugees who are undocumented should receive the same care and insurance coverage as US born patients.

- ◯ Never
- ◯ Rarely
- ◯ Sometimes
- ⊗ Usually
- ◯ Always

j. Every physician is professionally obligated to care for immigrants and refugees if they present to your clinic or hospital.

- ◯ Strongly disagree
- ◯ Disagree
- ◯ No opinion
- ◯ Agree
- ⊗ Strongly agree

k. Is healthcare a human right?

- ⊗ Yes
- ◯ No

B. If you wish, please tell us about what you enjoy or do not enjoy about immigrant and refugee health care and the greatest challenges you face in caring for this population.

____

SECTION D: DEMOGRAPHIC INFORMATION
Please answer the following questions by checking the box in front of the response choice that best describes you.

a. Your age?
- ◯ 20 to 24
- ⊗ 25 to 29
- ◯ 30 to 34
- ◯ 35 to 39
- ◯ 40 or older

b. Your gender?
- ◯ Female
- ⊗ Male
- ◯ Other

c. Are you Hispanic or Latino?
- ◯ Yes
- ⊗ No

d. What is your race? (Select one or more responses)
- ◯ American Indian or Alaska Native
- ◯ Asian (Please specify):
- ◯ Black or African American
- ◯ Native Hawaiian or Other Pacific Islander
- ⊗ White
- ◯ Other (Please specify):

e. Were you born in the United States?
- ⊗ Yes
- ◯ No

g. Your residency year?
- ⊗ PGY1
- ◯ PGY2
- ◯ PGY3
- ◯ PGY4
- ◯ PGY5

h. How would you classify your political ideology?
- ◯ Conservative
- ◯ Somewhat conservative
- ◯ Moderate
- ◯ Somewhat liberal
- ⊗ Liberal
- ◯ Other (Please specify):

i. Estimated level of educational debt?
- ◯ None
- ◯ Less than $50,000
- ◯ $50,000 - $100,000
- ◯ $100,000 - $200,000
- ⊗ $200,000 or more

j. Do you plan to subspecialize?
- ⊗ Yes
- ◯ No

k. Languages spoken?
- ☒ English
- ☐ Spanish
- ☐ French
- ☐ Hmong
- ☐ Somali
- ☐ Japanese
- ☐ Chinese
- ☐ Russian
- ☐ Ethiopian
- ☐ Other ___________

l. Are you in the Global Health Pathway?
- ◯ Yes
- ⊗ No

m. Did you earn your degree in the US?

- ⊗ Yes
- ◯ No

n. What residency program are you in?

- ⊗ Internal Medicine
- ◯ Med-Peds
- ◯ Pediatrics
- ◯ Family Practice
- ◯ Neurology
- ◯ Psychiatry
- ◯ ObGyn
- ◯ Neurosurgery
- ◯ General Surgery
- ◯ Orthopedic Surgery
- ◯ Urology
- ◯ Surgical sub-specialty (please specify in text box below)
- ◯ Non-clinical specialty (radiology, pathology; please specify in text box below)

Medical Trainees' attitudes, knowledge, and experience with immigrant and refugee health

Response was added on 05/04/2014 12:31pm.

SECTION A: Personal experience with immigrant and refugee health care.

A. Please indicate your level of agreement with the following statements regarding your personal experience with immigrant and refugee health care by checking the box that best represents your experience.

a. During my inpatient rotations, I take care of the following percentage of immigrant and refugee patients:	◯ None<br>◯ 0 -5%<br>⊗ 5-10%<br>◯ 10-25%<br>◯ > 25%
b. During my outpatient rotations, I take care of the following percentage of immigrant and refugee patients:	⊗ None<br>◯ 0-10%<br>◯ 10 -25%<br>◯ 25-50%<br>◯ 50-75%<br>◯ >75%
c. I would like to take care of more immigrant and refugee patients.	◯ Strongly disagree<br>◯ Disagree<br>◯ No opinion<br>⊗ Agree<br>◯ Strongly agree
d. I plan to take care of immigrants and refugees when I finish residency.	◯ Strongly disagree<br>◯ Disagree<br>⊗ No opinion<br>◯ Agree<br>◯ Strongly agree
e. I plan to do short term (< 6 months) international work when I finish residency.	◯ Strongly disagree<br>⊗ Disagree<br>◯ No opinion<br>◯ Agree<br>◯ Strongly agree
f. I plan to do long term (>6 months) international work when I finish residency.	◯ Strongly disagree<br>⊗ Disagree<br>◯ No opinion<br>◯ Agree<br>◯ Strongly agree
g. I plan to work in health disparities in the following way after residency:	Urban, academic center

SECTION B: MEDICAL EDUCATION

A. Please indicate your level of agreement with the following statements regarding your medical education and knowledge about immigrants and refugees by checking the box that best represents your opinion.

a. I have received specialized training in immigrant and refugee health, tropical medicine, or cross-cultural health.

- ◯ Strongly disagree
- ⊗ Disagree
- ◯ No opinion
- ◯ Agree
- ◯ Strongly agree

c. I feel comfortable with my fund of knowledge regarding immigrant and refugee health.

- ◯ Strongly disagree
- ⊗ Disagree
- ◯ No opinion
- ◯ Agree
- ◯ Strongly agree

d. I would like to have further training in immigrant and refugee health.

- ◯ Strongly disagree
- ◯ Disagree
- ◯ No opinion
- ⊗ Agree
- ◯ Strongly agree

e. If you agree with the above, please indicate all the contexts in which you would like to receive this training:
□□

- ☒ As part of my residency.
- ☐ A special program.
- ☐ As part of my fellowship.

SECTION C: Attitudes towards immigrant health

A.□Please indicate your level of agreement with the following statements regarding immigrant and refugee health by checking the box that best represents your opinion.

a. I enjoy taking care of immigrants and refugees.

- ◯ Never
- ◯ Rarely
- ⊗ Sometimes
- ◯ Usually
- ◯ Always

b. Please indicate the reasons that you enjoy taking care of immigrants and refugees (may choose more than one).

- ☐ Tropical and other conditions not frequently diagnosed in US-born patients
- ☐ Learning about other cultures
- ☐ They don't complain as much
- ☐ Being able to hear their stories
- ☐ Their care is more complicated
- ☐ Their care is less complicated
- ☐ They are very appreciative of your help.
- ☒ They are extremely vulnerable
- ☐ Other:

c. Taking care of immigrants and refugees is more challenging than taking care of US born patients.

- ◯ Never
- ◯ Rarely
- ◯ Sometimes
- ⊗ Usually
- ◯ Always

d. Please mark all the challenges that you face as a provider when providing care to immigrants and refugees (may choose more than one):

- ☒ Language barriers
- ☐ Insurance barriers
- ☒ Cultural barriers
- ☐ Finding a professional interpreter
- ☐ Knowing how to work with a professional interpreter
- ☐ Time constraints
- ☐ My own knowledge related to tropical and travel medicine
- ☐ Transportation problems for the patient
- ☐ Patients not understanding treatment plan
- ☒ Patients not following treatment plan
- ☐ My lack of knowledge regarding the patient's culture
- ☐ Bias or stereotyping
- ☐ Other:

e. Please mark all of the challenges faced by immigrant and refugee populations when receiving healthcare that you have perceived or witnessed (may choose more than one):

- ☐ Language barriers
- ☐ Insurance barriers
- ☐ Cultural barriers
- ☐ Finding a professional interpreter
- ☐ Knowing how to work with a professional interpreter
- ☐ Time constraints
- ☐ Insufficiently trained health care providers
- ☐ Transportation problems for the patient
- ☒ Food insufficiency
- ☒ Need for child care
- ☐ Patients not understanding treatment plan
- ☐ Patients not following treatment plan
- ☐ My lack of knowledge regarding the patient's culture
- ☐ Bias or stereotyping
- ☐ Trust issues
- ☐ Other...

f. Rank how well immigrants and refugees understand the healthcare that you are trying to provide.

- ◯ Significantly less than a US born individual
- ⊗ Less than a US born individual
- ◯ Equivalent to a US born individual
- ◯ More than a US born patient
- ◯ Significantly more than a US born individual

g. Immigrants and refugees adhere to treatment plans and follow my recommendations.

- ◯ Never
- ◯ Rarely
- ⊗ Sometimes
- ◯ Usually
- ◯ Always

h. Immigrants and refugees should receive the same care and insurance coverage as US born patients.

- ◯ Never
- ◯ Rarely
- ⊗ Sometimes
- ◯ Usually
- ◯ Always

i. Immigrants and refugees who are undocumented should receive the same care and insurance coverage as US born patients.

- ◯ Never
- ⊗ Rarely
- ◯ Sometimes
- ◯ Usually
- ◯ Always

j. Every physician is professionally obligated to care for immigrants and refugees if they present to your clinic or hospital.

- ◯ Strongly disagree
- ◯ Disagree
- ◯ No opinion
- ⊗ Agree
- ◯ Strongly agree

k. Is healthcare a human right?
- ⊗ Yes
- ◯ No

B. If you wish, please tell us about what you enjoy or do not enjoy about immigrant and refugee health care and the greatest challenges you face in caring for this population.

____

SECTION D: DEMOGRAPHIC INFORMATION
Please answer the following questions by checking the box in front of the response choice that best describes you.

a. Your age?
- ◯ 20 to 24
- ⊗ 25 to 29
- ◯ 30 to 34
- ◯ 35 to 39
- ◯ 40 or older

b. Your gender?
- ◯ Female
- ⊗ Male
- ◯ Other

c.Are you Hispanic or Latino?
- ◯ Yes
- ⊗ No

d. What is your race? (Select one or more responses)
- ◯ American Indian or Alaska Native
- ⊗ Asian (Please specify):
- ◯ Black or African American
- ◯ Native Hawaiian or Other Pacific Islander
- ◯ White
- ◯ Other (Please specify):

e.Were you born in the United States?
- ⊗ Yes
- ◯ No

g. Your residency year?
- ◯ PGY1
- ⊗ PGY2
- ◯ PGY3
- ◯ PGY4
- ◯ PGY5

h. How would you classify your political ideology?
- ◯ Conservative
- ◯ Somewhat conservative
- ◯ Moderate
- ◯ Somewhat liberal
- ⊗ Liberal
- ◯ Other (Please specify):

i. Estimated level of educational debt?
- ◯ None
- ⊗ Less than $50,000
- ◯ $50,000 - $100,000
- ◯ $100,000 - $200,000
- ◯ $200,000 or more

j.Do you plan to subspecialize?
- ⊗ Yes
- ◯ No

k. Languages spoken?

- ☒ English
- ☐ Spanish
- ☐ French
- ☐ Hmong
- ☐ Somali
- ☐ Japanese
- ☐ Chinese
- ☐ Russian
- ☐ Ethiopian
- ☐ Other ___________

l. Are you in the Global Health Pathway?

- ◯ Yes
- ◯ No

m. Did you earn your degree in the US?

- ⊗ Yes
- ◯ No

n. What residency program are you in?

- ⊗ Internal Medicine
- ◯ Med-Peds
- ◯ Pediatrics
- ◯ Family Practice
- ◯ Neurology
- ◯ Psychiatry
- ◯ ObGyn
- ◯ Neurosurgery
- ◯ General Surgery
- ◯ Orthopedic Surgery
- ◯ Urology
- ◯ Surgical sub-specialty (please specify in text box below)
- ◯ Non-clinical specialty (radiology, pathology; please specify in text box below)

Medical Trainees' attitudes, knowledge, and experience with immigrant and refugee health

Response was added on 05/04/2014 4:15pm.

SECTION A: Personal experience with immigrant and refugee health care.

A. Please indicate your level of agreement with the following statements regarding your personal experience with immigrant and refugee health care by checking the box that best represents your experience.

a. During my inpatient rotations, I take care of the following percentage of immigrant and refugee patients:

- ◯ None
- ◯ 0 -5%
- ◯ 5-10%
- ⊗ 10-25%
- ◯ > 25%

b. During my outpatient rotations, I take care of the following percentage of immigrant and refugee patients:

- ◯ None
- ◯ 0-10%
- ◯ 10 -25%
- ◯ 25-50%
- ⊗ 50-75%
- ◯ >75%

c. I would like to take care of more immigrant and refugee patients.

- ◯ Strongly disagree
- ◯ Disagree
- ◯ No opinion
- ◯ Agree
- ⊗ Strongly agree

d. I plan to take care of immigrants and refugees when I finish residency.

- ◯ Strongly disagree
- ◯ Disagree
- ◯ No opinion
- ◯ Agree
- ⊗ Strongly agree

e. I plan to do short term (< 6 months) international work when I finish residency.

- ◯ Strongly disagree
- ◯ Disagree
- ◯ No opinion
- ◯ Agree
- ⊗ Strongly agree

f. I plan to do long term (>6 months) international work when I finish residency.

- ◯ Strongly disagree
- ◯ Disagree
- ⊗ No opinion
- ◯ Agree
- ◯ Strongly agree

g. I plan to work in health disparities in the following way after residency:

I would like to work in a community/immigrant-based clinic and also to participate in mission trips abroad.

SECTION B: MEDICAL EDUCATION

A. Please indicate your level of agreement with the following statements regarding your medical education and knowledge about immigrants and refugees by checking the box that best represents your opinion.

a. I have received specialized training in immigrant and refugee health, tropical medicine, or cross-cultural health.

- ◯ Strongly disagree
- ◯ Disagree
- ◯ No opinion
- ◯ Agree
- ⊗ Strongly agree

b. If you have received specialized training in immigrant and refugee health, tropical medicine, or cross-cultural health, please indicate all the contexts in which you received this training:

- ☐ As an undergraduate.
- ☐ As a medical student.
- ☒ As part of my residency.
- ☐ A special program.
- ☐ As part of my fellowship.
- ☐ As part of a degree program (e.g. MPH)
- ☐ Other:

c. I feel comfortable with my fund of knowledge regarding immigrant and refugee health.

- ◯ Strongly disagree
- ◯ Disagree
- ◯ No opinion
- ⊗ Agree
- ◯ Strongly agree

d. I would like to have further training in immigrant and refugee health.

- ◯ Strongly disagree
- ◯ Disagree
- ◯ No opinion
- ⊗ Agree
- ◯ Strongly agree

e. If you agree with the above, please indicate all the contexts in which you would like to receive this training:

- ☐ As part of my residency.
- ☒ A special program.
- ☐ As part of my fellowship.

SECTION C: Attitudes towards immigrant health

A. Please indicate your level of agreement with the following statements regarding immigrant and refugee health by checking the box that best represents your opinion.

a. I enjoy taking care of immigrants and refugees.

- ◯ Never
- ◯ Rarely
- ◯ Sometimes
- ◯ Usually
- ⊗ Always

b. Please indicate the reasons that you enjoy taking care of immigrants and refugees (may choose more than one).

- ☒ Tropical and other conditions not frequently diagnosed in US-born patients
- ☒ Learning about other cultures
- ☐ They don't complain as much
- ☒ Being able to hear their stories
- ☒ Their care is more complicated
- ☐ Their care is less complicated
- ☐ They are very appreciative of your help.
- ☐ They are extremely vulnerable
- ☐ Other:

c. Taking care of immigrants and refugees is more challenging than taking care of US born patients.

- ◯ Never
- ◯ Rarely
- ◯ Sometimes
- ⊗ Usually
- ◯ Always

d. Please mark all the challenges that you face as a provider when providing care to immigrants and refugees (may choose more than one):

- ☒ Language barriers
- ☒ Insurance barriers
- ☒ Cultural barriers
- ☒ Finding a professional interpreter
- ☐ Knowing how to work with a professional interpreter
- ☒ Time constraints
- ☒ My own knowledge related to tropical and travel medicine
- ☐ Transportation problems for the patient
- ☒ Patients not understanding treatment plan
- ☒ Patients not following treatment plan
- ☒ My lack of knowledge regarding the patient's culture
- ☐ Bias or stereotyping
- ☐ Other:

e. Please mark all of the challenges faced by immigrant and refugee populations when receiving healthcare that you have perceived or witnessed (may choose more than one):

- ☒ Language barriers
- ☒ Insurance barriers
- ☒ Cultural barriers
- ☒ Finding a professional interpreter
- ☐ Knowing how to work with a professional interpreter
- ☒ Time constraints
- ☒ Insufficiently trained health care providers
- ☐ Transportation problems for the patient
- ☐ Food insufficiency
- ☐ Need for child care
- ☒ Patients not understanding treatment plan
- ☒ Patients not following treatment plan
- ☒ My lack of knowledge regarding the patient's culture
- ☒ Bias or stereotyping
- ☒ Trust issues
- ☐ Other...

f. Rank how well immigrants and refugees understand the healthcare that you are trying to provide.

- ○ Significantly less than a US born individual
- ⊗ Less than a US born individual
- ○ Equivalent to a US born individual
- ○ More than a US born patient
- ○ Significantly more than a US born individual

g. Immigrants and refugees adhere to treatment plans and follow my recommendations.

- ○ Never
- ○ Rarely
- ⊗ Sometimes
- ○ Usually
- ○ Always

h. Immigrants and refugees should receive the same care and insurance coverage as US born patients.

- ○ Never
- ○ Rarely
- ○ Sometimes
- ○ Usually
- ⊗ Always

i. Immigrants and refugees who are undocumented should receive the same care and insurance coverage as US born patients.

- ○ Never
- ○ Rarely
- ○ Sometimes
- ○ Usually
- ⊗ Always

j. Every physician is professionally obligated to care for immigrants and refugees if they present to your clinic or hospital.

- ○ Strongly disagree
- ○ Disagree
- ○ No opinion
- ○ Agree
- ⊗ Strongly agree

k. Is healthcare a human right?
- ⊗ Yes
- ◯ No

B. If you wish, please tell us about what you enjoy or do not enjoy about immigrant and refugee health care and the greatest challenges you face in caring for this population.

____

SECTION D: DEMOGRAPHIC INFORMATION
Please answer the following questions by checking the box in front of the response choice that best describes you.

a. Your age?
- ◯ 20 to 24
- ◯ 25 to 29
- ⊗ 30 to 34
- ◯ 35 to 39
- ◯ 40 or older

b. Your gender?
- ⊗ Female
- ◯ Male
- ◯ Other

c.Are you Hispanic or Latino?
- ◯ Yes
- ⊗ No

d. What is your race? (Select one or more responses)
- ◯ American Indian or Alaska Native
- ⊗ Asian (Please specify):
- ◯ Black or African American
- ◯ Native Hawaiian or Other Pacific Islander
- ◯ White
- ◯ Other (Please specify):

e.Were you born in the United States?
- ◯ Yes
- ⊗ No

f.If not, in what country were you born?
Vietnam

g. Your residency year?
- ◯ PGY1
- ◯ PGY2
- ◯ PGY3
- ⊗ PGY4
- ◯ PGY5

h. How would you classify your political ideology?
- ◯ Conservative
- ◯ Somewhat conservative
- ◯ Moderate
- ◯ Somewhat liberal
- ⊗ Liberal
- ◯ Other (Please specify):

i. Estimated level of educational debt?
- ◯ None
- ◯ Less than $50,000
- ◯ $50,000 - $100,000
- ⊗ $100,000 - $200,000
- ◯ $200,000 or more

j.Do you plan to subspecialize?
- ◯ Yes
- ⊗ No

k. Languages spoken?

- [x] English
- [ ] Spanish
- [ ] French
- [ ] Hmong
- [ ] Somali
- [ ] Japanese
- [ ] Chinese
- [ ] Russian
- [ ] Ethiopian
- [ ] Other ___________

l. Are you in the Global Health Pathway?

- (x) Yes
- ( ) No

m. Did you earn your degree in the US?

- (x) Yes
- ( ) No

n. What residency program are you in?

- ( ) Internal Medicine
- (x) Med-Peds
- ( ) Pediatrics
- ( ) Family Practice
- ( ) Neurology
- ( ) Psychiatry
- ( ) ObGyn
- ( ) Neurosurgery
- ( ) General Surgery
- ( ) Orthopedic Surgery
- ( ) Urology
- ( ) Surgical sub-specialty (please specify in text box below)
- ( ) Non-clinical specialty (radiology, pathology; please specify in text box below)

Medical Trainees' attitudes, knowledge, and experience with immigrant and refugee health

Response was added on 05/05/2014 8:55am.

SECTION A: Personal experience with immigrant and refugee health care.

A. Please indicate your level of agreement with the following statements regarding your personal experience with immigrant and refugee health care by checking the box that best represents your experience.

a. During my inpatient rotations, I take care of the following percentage of immigrant and refugee patients:	◯ None<br>⊗ 0 -5%<br>◯ 5-10%<br>◯ 10-25%<br>◯ > 25%
b. During my outpatient rotations, I take care of the following percentage of immigrant and refugee patients:	◯ None<br>⊗ 0-10%<br>◯ 10 -25%<br>◯ 25-50%<br>◯ 50-75%<br>◯ >75%
c. I would like to take care of more immigrant and refugee patients.	◯ Strongly disagree<br>◯ Disagree<br>⊗ No opinion<br>◯ Agree<br>◯ Strongly agree
d. I plan to take care of immigrants and refugees when I finish residency.	◯ Strongly disagree<br>◯ Disagree<br>⊗ No opinion<br>◯ Agree<br>◯ Strongly agree
e. I plan to do short term (< 6 months) international work when I finish residency.	⊗ Strongly disagree<br>◯ Disagree<br>◯ No opinion<br>◯ Agree<br>◯ Strongly agree
f. I plan to do long term (>6 months) international work when I finish residency.	⊗ Strongly disagree<br>◯ Disagree<br>◯ No opinion<br>◯ Agree<br>◯ Strongly agree
g. I plan to work in health disparities in the following way after residency:	Working in hospital caring for all patients, consider medical trips abroad

SECTION B: MEDICAL EDUCATION

A. Please indicate your level of agreement with the following statements regarding your medical education and knowledge about immigrants and refugees by checking the box that best represents your opinion.

a. I have received specialized training in immigrant and refugee health, tropical medicine, or cross-cultural health.

- ◯ Strongly disagree
- ⊗ Disagree
- ◯ No opinion
- ◯ Agree
- ◯ Strongly agree

c. I feel comfortable with my fund of knowledge regarding immigrant and refugee health.

- ◯ Strongly disagree
- ⊗ Disagree
- ◯ No opinion
- ◯ Agree
- ◯ Strongly agree

d. I would like to have further training in immigrant and refugee health.

- ◯ Strongly disagree
- ◯ Disagree
- ◯ No opinion
- ⊗ Agree
- ◯ Strongly agree

e. If you agree with the above, please indicate all the contexts in which you would like to receive this training:
□□

- ☒ As part of my residency.
- ☐ A special program.
- ☐ As part of my fellowship.

SECTION C: Attitudes towards immigrant health

A.□Please indicate your level of agreement with the following statements regarding immigrant and refugee health by checking the box that best represents your opinion.

a. I enjoy taking care of immigrants and refugees.

- ◯ Never
- ◯ Rarely
- ⊗ Sometimes
- ◯ Usually
- ◯ Always

b. Please indicate the reasons that you enjoy taking care of immigrants and refugees (may choose more than one).

- ☒ Tropical and other conditions not frequently diagnosed in US-born patients
- ☐ Learning about other cultures
- ☐ They don't complain as much
- ☐ Being able to hear their stories
- ☐ Their care is more complicated
- ☐ Their care is less complicated
- ☒ They are very appreciative of your help.
- ☒ They are extremely vulnerable
- ☐ Other:

c. Taking care of immigrants and refugees is more challenging than taking care of US born patients.

- ◯ Never
- ◯ Rarely
- ◯ Sometimes
- ⊗ Usually
- ◯ Always

d. Please mark all the challenges that you face as a provider when providing care to immigrants and refugees (may choose more than one):

- [x] Language barriers
- [ ] Insurance barriers
- [x] Cultural barriers
- [x] Finding a professional interpreter
- [ ] Knowing how to work with a professional interpreter
- [x] Time constraints
- [x] My own knowledge related to tropical and travel medicine
- [ ] Transportation problems for the patient
- [x] Patients not understanding treatment plan
- [x] Patients not following treatment plan
- [ ] My lack of knowledge regarding the patient's culture
- [ ] Bias or stereotyping
- [ ] Other:

e. Please mark all of the challenges faced by immigrant and refugee populations when receiving healthcare that you have perceived or witnessed (may choose more than one):

- [x] Language barriers
- [x] Insurance barriers
- [x] Cultural barriers
- [x] Finding a professional interpreter
- [x] Knowing how to work with a professional interpreter
- [ ] Time constraints
- [ ] Insufficiently trained health care providers
- [ ] Transportation problems for the patient
- [ ] Food insufficiency
- [ ] Need for child care
- [x] Patients not understanding treatment plan
- [ ] Patients not following treatment plan
- [ ] My lack of knowledge regarding the patient's culture
- [ ] Bias or stereotyping
- [ ] Trust issues
- [ ] Other...

f. Rank how well immigrants and refugees understand the healthcare that you are trying to provide.

- ( ) Significantly less than a US born individual
- (x) Less than a US born individual
- ( ) Equivalent to a US born individual
- ( ) More than a US born patient
- ( ) Significantly more than a US born individual

g. Immigrants and refugees adhere to treatment plans and follow my recommendations.

- ( ) Never
- ( ) Rarely
- ( ) Sometimes
- (x) Usually
- ( ) Always

h. Immigrants and refugees should receive the same care and insurance coverage as US born patients.

- ( ) Never
- ( ) Rarely
- ( ) Sometimes
- ( ) Usually
- (x) Always

i. Immigrants and refugees who are undocumented should receive the same care and insurance coverage as US born patients.

- ( ) Never
- ( ) Rarely
- ( ) Sometimes
- ( ) Usually
- (x) Always

j. Every physician is professionally obligated to care for immigrants and refugees if they present to your clinic or hospital.

- ( ) Strongly disagree
- ( ) Disagree
- ( ) No opinion
- ( ) Agree
- (x) Strongly agree

k. Is healthcare a human right?
⊗ Yes
◯ No

B. If you wish, please tell us about what you enjoy or do not enjoy about immigrant and refugee health care and the greatest challenges you face in caring for this population.
____

SECTION D: DEMOGRAPHIC INFORMATION
Please answer the following questions by checking the box in front of the response choice that best describes you.

a. Your age?
◯ 20 to 24
⊗ 25 to 29
◯ 30 to 34
◯ 35 to 39
◯ 40 or older

b. Your gender?
◯ Female
⊗ Male
◯ Other

c.Are you Hispanic or Latino?
◯ Yes
⊗ No

d. What is your race? (Select one or more responses)
◯ American Indian or Alaska Native
◯ Asian (Please specify):
◯ Black or African American
◯ Native Hawaiian or Other Pacific Islander
⊗ White
◯ Other (Please specify):

e.Were you born in the United States?
⊗ Yes
◯ No

g. Your residency year?
◯ PGY1
⊗ PGY2
◯ PGY3
◯ PGY4
◯ PGY5

h. How would you classify your political ideology?
◯ Conservative
◯ Somewhat conservative
◯ Moderate
⊗ Somewhat liberal
◯ Liberal
◯ Other (Please specify):

i. Estimated level of educational debt?
◯ None
◯ Less than $50,000
◯ $50,000 - $100,000
◯ $100,000 - $200,000
⊗ $200,000 or more

j.Do you plan to subspecialize?
◯ Yes
⊗ No

k. Languages spoken?

- ☒ English
- ☐ Spanish
- ☐ French
- ☐ Hmong
- ☐ Somali
- ☐ Japanese
- ☐ Chinese
- ☐ Russian
- ☐ Ethiopian
- ☐ Other ___________

l. Are you in the Global Health Pathway?

- ○ Yes
- ⊗ No

m. Did you earn your degree in the US?

- ⊗ Yes
- ○ No

n. What residency program are you in?

- ⊗ Internal Medicine
- ○ Med-Peds
- ○ Pediatrics
- ○ Family Practice
- ○ Neurology
- ○ Psychiatry
- ○ ObGyn
- ○ Neurosurgery
- ○ General Surgery
- ○ Orthopedic Surgery
- ○ Urology
- ○ Surgical sub-specialty (please specify in text box below)
- ○ Non-clinical specialty (radiology, pathology; please specify in text box below)

Medical Trainees' attitudes, knowledge, and experience with immigrant and refugee health

Response was added on 05/07/2014 10:32am.

SECTION A: Personal experience with immigrant and refugee health care.

A. Please indicate your level of agreement with the following statements regarding your personal experience with immigrant and refugee health care by checking the box that best represents your experience.

a. During my inpatient rotations, I take care of the following percentage of immigrant and refugee patients:

- ◯ None
- ◯ 0 -5%
- ⊗ 5-10%
- ◯ 10-25%
- ◯ > 25%

b. During my outpatient rotations, I take care of the following percentage of immigrant and refugee patients:

- ◯ None
- ◯ 0-10%
- ⊗ 10 -25%
- ◯ 25-50%
- ◯ 50-75%
- ◯ >75%

c. I would like to take care of more immigrant and refugee patients.

- ◯ Strongly disagree
- ◯ Disagree
- ⊗ No opinion
- ◯ Agree
- ◯ Strongly agree

d. I plan to take care of immigrants and refugees when I finish residency.

- ◯ Strongly disagree
- ◯ Disagree
- ◯ No opinion
- ⊗ Agree
- ◯ Strongly agree

e. I plan to do short term (< 6 months) international work when I finish residency.

- ⊗ Strongly disagree
- ◯ Disagree
- ◯ No opinion
- ◯ Agree
- ◯ Strongly agree

f. I plan to do long term (>6 months) international work when I finish residency.

- ⊗ Strongly disagree
- ◯ Disagree
- ◯ No opinion
- ◯ Agree
- ◯ Strongly agree

g. I plan to work in health disparities in the following way after residency:

All physicians should be working to address health disparities in their patient panel.

SECTION B: MEDICAL EDUCATION

A. Please indicate your level of agreement with the following statements regarding your medical education and knowledge about immigrants and refugees by checking the box that best represents your opinion.

a. I have received specialized training in immigrant and refugee health, tropical medicine, or cross-cultural health.

- ◯ Strongly disagree
- ⊗ Disagree
- ◯ No opinion
- ◯ Agree
- ◯ Strongly agree

c. I feel comfortable with my fund of knowledge regarding immigrant and refugee health.

- ◯ Strongly disagree
- ⊗ Disagree
- ◯ No opinion
- ◯ Agree
- ◯ Strongly agree

d. I would like to have further training in immigrant and refugee health.

- ◯ Strongly disagree
- ◯ Disagree
- ◯ No opinion
- ⊗ Agree
- ◯ Strongly agree

e. If you agree with the above, please indicate all the contexts in which you would like to receive this training:

- ☒ As part of my residency.
- ☒ A special program.
- ☐ As part of my fellowship.

SECTION C: Attitudes towards immigrant health

A. Please indicate your level of agreement with the following statements regarding immigrant and refugee health by checking the box that best represents your opinion.

a. I enjoy taking care of immigrants and refugees.

- ◯ Never
- ◯ Rarely
- ◯ Sometimes
- ⊗ Usually
- ◯ Always

b. Please indicate the reasons that you enjoy taking care of immigrants and refugees (may choose more than one).

- ☐ Tropical and other conditions not frequently diagnosed in US-born patients
- ☒ Learning about other cultures
- ☐ They don't complain as much
- ☒ Being able to hear their stories
- ☒ Their care is more complicated
- ☐ Their care is less complicated
- ☒ They are very appreciative of your help.
- ☒ They are extremely vulnerable
- ☐ Other:

c. Taking care of immigrants and refugees is more challenging than taking care of US born patients.

- ◯ Never
- ◯ Rarely
- ⊗ Sometimes
- ◯ Usually
- ◯ Always

d. Please mark all the challenges that you face as a provider when providing care to immigrants and refugees (may choose more than one):

- ☒ Language barriers
- ☒ Insurance barriers
- ☒ Cultural barriers
- ☐ Finding a professional interpreter
- ☐ Knowing how to work with a professional interpreter
- ☒ Time constraints
- ☒ My own knowledge related to tropical and travel medicine
- ☐ Transportation problems for the patient
- ☐ Patients not understanding treatment plan
- ☐ Patients not following treatment plan
- ☒ My lack of knowledge regarding the patient's culture
- ☒ Bias or stereotyping
- ☐ Other:

e. Please mark all of the challenges faced by immigrant and refugee populations when receiving healthcare that you have perceived or witnessed (may choose more than one):

- ☒ Language barriers
- ☒ Insurance barriers
- ☒ Cultural barriers
- ☐ Finding a professional interpreter
- ☐ Knowing how to work with a professional interpreter
- ☒ Time constraints
- ☒ Insufficiently trained health care providers
- ☒ Transportation problems for the patient
- ☐ Food insufficiency
- ☒ Need for child care
- ☒ Patients not understanding treatment plan
- ☐ Patients not following treatment plan
- ☒ My lack of knowledge regarding the patient's culture
- ☒ Bias or stereotyping
- ☒ Trust issues
- ☐ Other...

f. Rank how well immigrants and refugees understand the healthcare that you are trying to provide.

- ◯ Significantly less than a US born individual
- ⊗ Less than a US born individual
- ◯ Equivalent to a US born individual
- ◯ More than a US born patient
- ◯ Significantly more than a US born individual

g. Immigrants and refugees adhere to treatment plans and follow my recommendations.

- ◯ Never
- ◯ Rarely
- ◯ Sometimes
- ⊗ Usually
- ◯ Always

h. Immigrants and refugees should receive the same care and insurance coverage as US born patients.

- ◯ Never
- ◯ Rarely
- ◯ Sometimes
- ◯ Usually
- ⊗ Always

i. Immigrants and refugees who are undocumented should receive the same care and insurance coverage as US born patients.

- ◯ Never
- ◯ Rarely
- ◯ Sometimes
- ◯ Usually
- ⊗ Always

j. Every physician is professionally obligated to care for immigrants and refugees if they present to your clinic or hospital.

- ◯ Strongly disagree
- ◯ Disagree
- ◯ No opinion
- ◯ Agree
- ⊗ Strongly agree

k. Is healthcare a human right?

- ⊗ Yes
- ◯ No

B. If you wish, please tell us about what you enjoy or do not enjoy about immigrant and refugee health care and the greatest challenges you face in caring for this population. ________________________________

SECTION D: DEMOGRAPHIC INFORMATION
Please answer the following questions by checking the box in front of the response choice that best describes you.

a. Your age?

- ◯ 20 to 24
- ◯ 25 to 29
- ⊗ 30 to 34
- ◯ 35 to 39
- ◯ 40 or older

b. Your gender?

- ⊗ Female
- ◯ Male
- ◯ Other

c. Are you Hispanic or Latino?

- ◯ Yes
- ⊗ No

d. What is your race? (Select one or more responses)

- ◯ American Indian or Alaska Native
- ◯ Asian (Please specify):
- ◯ Black or African American
- ◯ Native Hawaiian or Other Pacific Islander
- ⊗ White
- ◯ Other (Please specify):

e. Were you born in the United States?

- ⊗ Yes
- ◯ No

g. Your residency year?

- ◯ PGY1
- ◯ PGY2
- ⊗ PGY3
- ◯ PGY4
- ◯ PGY5

h. How would you classify your political ideology?

- ◯ Conservative
- ◯ Somewhat conservative
- ◯ Moderate
- ⊗ Somewhat liberal
- ◯ Liberal
- ◯ Other (Please specify):

i. Estimated level of educational debt?

- ◯ None
- ◯ Less than $50,000
- ◯ $50,000 - $100,000
- ⊗ $100,000 - $200,000
- ◯ $200,000 or more

j. Do you plan to subspecialize?

- ◯ Yes
- ⊗ No

k. Languages spoken?

- ☒ English
- ☒ Spanish
- ☐ French
- ☐ Hmong
- ☐ Somali
- ☐ Japanese
- ☐ Chinese
- ☐ Russian
- ☐ Ethiopian
- ☐ Other ___________

l. Are you in the Global Health Pathway?

- ◯ Yes
- ⊗ No

m. Did you earn your degree in the US?

- ⊗ Yes
- ◯ No

n. What residency program are you in?

- ◯ Internal Medicine
- ⊗ Med-Peds
- ◯ Pediatrics
- ◯ Family Practice
- ◯ Neurology
- ◯ Psychiatry
- ◯ ObGyn
- ◯ Neurosurgery
- ◯ General Surgery
- ◯ Orthopedic Surgery
- ◯ Urology
- ◯ Surgical sub-specialty (please specify in text box below)
- ◯ Non-clinical specialty (radiology, pathology; please specify in text box below)

Medical Trainees' attitudes, knowledge, and experience with immigrant and refugee health

Response was added on 05/07/2014 9:53pm.

SECTION A: Personal experience with immigrant and refugee health care.

A. Please indicate your level of agreement with the following statements regarding your personal experience with immigrant and refugee health care by checking the box that best represents your experience.

a. During my inpatient rotations, I take care of the following percentage of immigrant and refugee patients:	◯ None<br>◯ 0 -5%<br>⊗ 5-10%<br>◯ 10-25%<br>◯ > 25%
b. During my outpatient rotations, I take care of the following percentage of immigrant and refugee patients:	⊗ None<br>◯ 0-10%<br>◯ 10 -25%<br>◯ 25-50%<br>◯ 50-75%<br>◯ >75%
c. I would like to take care of more immigrant and refugee patients.	◯ Strongly disagree<br>◯ Disagree<br>⊗ No opinion<br>◯ Agree<br>◯ Strongly agree
d. I plan to take care of immigrants and refugees when I finish residency.	◯ Strongly disagree<br>◯ Disagree<br>⊗ No opinion<br>◯ Agree<br>◯ Strongly agree
e. I plan to do short term (< 6 months) international work when I finish residency.	◯ Strongly disagree<br>⊗ Disagree<br>◯ No opinion<br>◯ Agree<br>◯ Strongly agree
f. I plan to do long term (>6 months) international work when I finish residency.	◯ Strongly disagree<br>⊗ Disagree<br>◯ No opinion<br>◯ Agree<br>◯ Strongly agree
g. I plan to work in health disparities in the following way after residency:	unsure

SECTION B: MEDICAL EDUCATION

A. Please indicate your level of agreement with the following statements regarding your medical education and knowledge about immigrants and refugees by checking the box that best represents your opinion.

a. I have received specialized training in immigrant and refugee health, tropical medicine, or cross-cultural health.

⊗ Strongly disagree
◯ Disagree
◯ No opinion
◯ Agree
◯ Strongly agree

c. I feel comfortable with my fund of knowledge regarding immigrant and refugee health.

⊗ Strongly disagree
◯ Disagree
◯ No opinion
◯ Agree
◯ Strongly agree

d. I would like to have further training in immigrant and refugee health.

◯ Strongly disagree
◯ Disagree
◯ No opinion
◯ Agree
⊗ Strongly agree

e. If you agree with the above, please indicate all the contexts in which you would like to receive this training:

☒ As part of my residency.
☐ A special program.
☐ As part of my fellowship.

SECTION C: Attitudes towards immigrant health

A. Please indicate your level of agreement with the following statements regarding immigrant and refugee health by checking the box that best represents your opinion.

a. I enjoy taking care of immigrants and refugees.

◯ Never
◯ Rarely
◯ Sometimes
⊗ Usually
◯ Always

b. Please indicate the reasons that you enjoy taking care of immigrants and refugees (may choose more than one).

☒ Tropical and other conditions not frequently diagnosed in US-born patients
☒ Learning about other cultures
☐ They don't complain as much
☐ Being able to hear their stories
☐ Their care is more complicated
☐ Their care is less complicated
☒ They are very appreciative of your help.
☐ They are extremely vulnerable
☐ Other:

c. Taking care of immigrants and refugees is more challenging than taking care of US born patients.

◯ Never
◯ Rarely
◯ Sometimes
⊗ Usually
◯ Always

d. Please mark all the challenges that you face as a provider when providing care to immigrants and refugees (may choose more than one):

- ☒ Language barriers
- ☒ Insurance barriers
- ☒ Cultural barriers
- ☒ Finding a professional interpreter
- ☒ Knowing how to work with a professional interpreter
- ☒ Time constraints
- ☒ My own knowledge related to tropical and travel medicine
- ☐ Transportation problems for the patient
- ☒ Patients not understanding treatment plan
- ☒ Patients not following treatment plan
- ☒ My lack of knowledge regarding the patient's culture
- ☒ Bias or stereotyping
- ☐ Other:

e. Please mark all of the challenges faced by immigrant and refugee populations when receiving healthcare that you have perceived or witnessed (may choose more than one):

- ☒ Language barriers
- ☒ Insurance barriers
- ☒ Cultural barriers
- ☒ Finding a professional interpreter
- ☒ Knowing how to work with a professional interpreter
- ☐ Time constraints
- ☒ Insufficiently trained health care providers
- ☒ Transportation problems for the patient
- ☐ Food insufficiency
- ☒ Need for child care
- ☒ Patients not understanding treatment plan
- ☒ Patients not following treatment plan
- ☒ My lack of knowledge regarding the patient's culture
- ☒ Bias or stereotyping
- ☒ Trust issues
- ☐ Other...

f. Rank how well immigrants and refugees understand the healthcare that you are trying to provide.

- ○ Significantly less than a US born individual
- ⊗ Less than a US born individual
- ○ Equivalent to a US born individual
- ○ More than a US born patient
- ○ Significantly more than a US born individual

g. Immigrants and refugees adhere to treatment plans and follow my recommendations.

- ○ Never
- ○ Rarely
- ○ Sometimes
- ⊗ Usually
- ○ Always

h. Immigrants and refugees should receive the same care and insurance coverage as US born patients.

- ○ Never
- ○ Rarely
- ○ Sometimes
- ○ Usually
- ⊗ Always

i. Immigrants and refugees who are undocumented should receive the same care and insurance coverage as US born patients.

- ○ Never
- ○ Rarely
- ○ Sometimes
- ○ Usually
- ⊗ Always

j. Every physician is professionally obligated to care for immigrants and refugees if they present to your clinic or hospital.

- ○ Strongly disagree
- ○ Disagree
- ○ No opinion
- ○ Agree
- ⊗ Strongly agree

k. Is healthcare a human right?
⊗ Yes
◯ No

B. If you wish, please tell us about what you enjoy or do not enjoy about immigrant and refugee health care and the greatest challenges you face in caring for this population.
interesting pathology

SECTION D: DEMOGRAPHIC INFORMATION
Please answer the following questions by checking the box in front of the response choice that best describes you.

a. Your age?
◯ 20 to 24
⊗ 25 to 29
◯ 30 to 34
◯ 35 to 39
◯ 40 or older

b. Your gender?
◯ Female
⊗ Male
◯ Other

c. Are you Hispanic or Latino?
◯ Yes
⊗ No

d. What is your race? (Select one or more responses)
◯ American Indian or Alaska Native
◯ Asian (Please specify):
◯ Black or African American
◯ Native Hawaiian or Other Pacific Islander
◯ White
⊗ Other (Please specify):

Asian-white

e. Were you born in the United States?
⊗ Yes
◯ No

g. Your residency year?
⊗ PGY1
◯ PGY2
◯ PGY3
◯ PGY4
◯ PGY5

h. How would you classify your political ideology?
◯ Conservative
◯ Somewhat conservative
⊗ Moderate
◯ Somewhat liberal
◯ Liberal
◯ Other (Please specify):

i. Estimated level of educational debt?
◯ None
◯ Less than $50,000
⊗ $50,000 - $100,000
◯ $100,000 - $200,000
◯ $200,000 or more

j. Do you plan to subspecialize?
⊗ Yes
◯ No

k. Languages spoken?

- ☒ English
- ☐ Spanish
- ☐ French
- ☐ Hmong
- ☐ Somali
- ☐ Japanese
- ☐ Chinese
- ☐ Russian
- ☐ Ethiopian
- ☐ Other ___________

l. Are you in the Global Health Pathway?

- ○ Yes
- ⊗ No

m. Did you earn your degree in the US?

- ⊗ Yes
- ○ No

n. What residency program are you in?

- ⊗ Internal Medicine
- ○ Med-Peds
- ○ Pediatrics
- ○ Family Practice
- ○ Neurology
- ○ Psychiatry
- ○ ObGyn
- ○ Neurosurgery
- ○ General Surgery
- ○ Orthopedic Surgery
- ○ Urology
- ○ Surgical sub-specialty (please specify in text box below)
- ○ Non-clinical specialty (radiology, pathology; please specify in text box below)

Medical Trainees' attitudes, knowledge, and experience with immigrant and refugee health

Response was added on 06/01/2014 2:01pm.

SECTION A: Personal experience with immigrant and refugee health care.

A. Please indicate your level of agreement with the following statements regarding your personal experience with immigrant and refugee health care by checking the box that best represents your experience.

a. During my inpatient rotations, I take care of the following percentage of immigrant and refugee patients:

- ◯ None
- ◯ 0 -5%
- ⊗ 5-10%
- ◯ 10-25%
- ◯ > 25%

b. During my outpatient rotations, I take care of the following percentage of immigrant and refugee patients:

- ◯ None
- ◯ 0-10%
- ⊗ 10 -25%
- ◯ 25-50%
- ◯ 50-75%
- ◯ >75%

c. I would like to take care of more immigrant and refugee patients.

- ◯ Strongly disagree
- ◯ Disagree
- ◯ No opinion
- ⊗ Agree
- ◯ Strongly agree

d. I plan to take care of immigrants and refugees when I finish residency.

- ◯ Strongly disagree
- ◯ Disagree
- ⊗ No opinion
- ◯ Agree
- ◯ Strongly agree

e. I plan to do short term (< 6 months) international work when I finish residency.

- ◯ Strongly disagree
- ◯ Disagree
- ⊗ No opinion
- ◯ Agree
- ◯ Strongly agree

f. I plan to do long term (>6 months) international work when I finish residency.

- ◯ Strongly disagree
- ◯ Disagree
- ⊗ No opinion
- ◯ Agree
- ◯ Strongly agree

g. I plan to work in health disparities in the following way after residency:

No plan to work in health disparities at this time, but may do so after my fellowship.

SECTION B: MEDICAL EDUCATION

A. Please indicate your level of agreement with the following statements regarding your medical education and knowledge about immigrants and refugees by checking the box that best represents your opinion.

a. I have received specialized training in immigrant and refugee health, tropical medicine, or cross-cultural health.

- ◯ Strongly disagree
- ◯ Disagree
- ◯ No opinion
- ⊗ Agree
- ◯ Strongly agree

b. If you have received specialized training in immigrant and refugee health, tropical medicine, or cross-cultural health, please indicate all the contexts in which you received this training:

- ☐ As an undergraduate.
- ☐ As a medical student.
- ☒ As part of my residency.
- ☐ A special program.
- ☐ As part of my fellowship.
- ☐ As part of a degree program (e.g. MPH)
- ☐ Other:

c. I feel comfortable with my fund of knowledge regarding immigrant and refugee health.

- ◯ Strongly disagree
- ◯ Disagree
- ◯ No opinion
- ⊗ Agree
- ◯ Strongly agree

d. I would like to have further training in immigrant and refugee health.

- ◯ Strongly disagree
- ⊗ Disagree
- ◯ No opinion
- ◯ Agree
- ◯ Strongly agree

SECTION C: Attitudes towards immigrant health

A.Please indicate your level of agreement with the following statements regarding immigrant and refugee health by checking the box that best represents your opinion.

a. I enjoy taking care of immigrants and refugees.

- ◯ Never
- ◯ Rarely
- ◯ Sometimes
- ◯ Usually
- ⊗ Always

b. Please indicate the reasons that you enjoy taking care of immigrants and refugees (may choose more than one).

- ☒ Tropical and other conditions not frequently diagnosed in US-born patients
- ☒ Learning about other cultures
- ☐ They don't complain as much
- ☒ Being able to hear their stories
- ☐ Their care is more complicated
- ☐ Their care is less complicated
- ☒ They are very appreciative of your help.
- ☒ They are extremely vulnerable
- ☐ Other:

c. Taking care of immigrants and refugees is more challenging than taking care of US born patients.

- ◯ Never
- ◯ Rarely
- ⊗ Sometimes
- ◯ Usually
- ◯ Always

d. Please mark all the challenges that you face as a provider when providing care to immigrants and refugees (may choose more than one):

- [x] Language barriers
- [ ] Insurance barriers
- [x] Cultural barriers
- [x] Finding a professional interpreter
- [ ] Knowing how to work with a professional interpreter
- [x] Time constraints
- [ ] My own knowledge related to tropical and travel medicine
- [ ] Transportation problems for the patient
- [x] Patients not understanding treatment plan
- [x] Patients not following treatment plan
- [x] My lack of knowledge regarding the patient's culture
- [ ] Bias or stereotyping
- [ ] Other:

e. Please mark all of the challenges faced by immigrant and refugee populations when receiving healthcare that you have perceived or witnessed (may choose more than one):

- [x] Language barriers
- [ ] Insurance barriers
- [x] Cultural barriers
- [ ] Finding a professional interpreter
- [x] Knowing how to work with a professional interpreter
- [ ] Time constraints
- [x] Insufficiently trained health care providers
- [ ] Transportation problems for the patient
- [ ] Food insufficiency
- [ ] Need for child care
- [x] Patients not understanding treatment plan
- [x] Patients not following treatment plan
- [x] My lack of knowledge regarding the patient's culture
- [ ] Bias or stereotyping
- [ ] Trust issues
- [ ] Other...

f. Rank how well immigrants and refugees understand the healthcare that you are trying to provide.

- ○ Significantly less than a US born individual
- ○ Less than a US born individual
- ⊗ Equivalent to a US born individual
- ○ More than a US born patient
- ○ Significantly more than a US born individual

g. Immigrants and refugees adhere to treatment plans and follow my recommendations.

- ○ Never
- ○ Rarely
- ⊗ Sometimes
- ○ Usually
- ○ Always

h. Immigrants and refugees should receive the same care and insurance coverage as US born patients.

- ○ Never
- ○ Rarely
- ○ Sometimes
- ○ Usually
- ⊗ Always

i. Immigrants and refugees who are undocumented should receive the same care and insurance coverage as US born patients.

- ○ Never
- ○ Rarely
- ○ Sometimes
- ○ Usually
- ⊗ Always

j. Every physician is professionally obligated to care for immigrants and refugees if they present to your clinic or hospital.

- ○ Strongly disagree
- ○ Disagree
- ○ No opinion
- ○ Agree
- ⊗ Strongly agree

k. Is healthcare a human right?

⊗ Yes
◯ No

B. If you wish, please tell us about what you enjoy or do not enjoy about immigrant and refugee health care and the greatest challenges you face in caring for this population.

They are vulnerable in many ways, which makes it very important to provide great care for them. However, taking care of this population poses great challenges to health care providers due to cultural difference and language barrier.

SECTION D: DEMOGRAPHIC INFORMATION
Please answer the following questions by checking the box in front of the response choice that best describes you.

a. Your age?

◯ 20 to 24
◯ 25 to 29
⊗ 30 to 34
◯ 35 to 39
◯ 40 or older

b. Your gender?

◯ Female
⊗ Male
◯ Other

c.Are you Hispanic or Latino?

◯ Yes
⊗ No

d. What is your race? (Select one or more responses)

◯ American Indian or Alaska Native
⊗ Asian (Please specify):
◯ Black or African American
◯ Native Hawaiian or Other Pacific Islander
◯ White
◯ Other (Please specify):

e.Were you born in the United States?

◯ Yes
⊗ No

f.If not, in what country were you born?

Korea

g. Your residency year?

◯ PGY1
◯ PGY2
⊗ PGY3
◯ PGY4
◯ PGY5

h. How would you classify your political ideology?

◯ Conservative
◯ Somewhat conservative
⊗ Moderate
◯ Somewhat liberal
◯ Liberal
◯ Other (Please specify):

i. Estimated level of educational debt?

⊗ None
◯ Less than $50,000
◯ $50,000 - $100,000
◯ $100,000 - $200,000
◯ $200,000 or more

j.Do you plan to subspecialize?

⊗ Yes
◯ No

k. Languages spoken?

☐ English
☐ Spanish
☐ French
☐ Hmong
☐ Somali
☐ Japanese
☐ Chinese
☐ Russian
☐ Ethiopian
☒ Other ___________

Korean

l. Are you in the Global Health Pathway?

◯ Yes
⊗ No

m. Did you earn your degree in the US?

◯ Yes
⊗ No

n. What residency program are you in?

⊗ Internal Medicine
◯ Med-Peds
◯ Pediatrics
◯ Family Practice
◯ Neurology
◯ Psychiatry
◯ ObGyn
◯ Neurosurgery
◯ General Surgery
◯ Orthopedic Surgery
◯ Urology
◯ Surgical sub-specialty (please specify in text box below)
◯ Non-clinical specialty (radiology, pathology; please specify in text box below)

Medical Trainees' attitudes, knowledge, and experience with immigrant and refugee health

Response was added on 06/15/2014 3:08pm.

SECTION A: Personal experience with immigrant and refugee health care.

A. Please indicate your level of agreement with the following statements regarding your personal experience with immigrant and refugee health care by checking the box that best represents your experience.

a. During my inpatient rotations, I take care of the following percentage of immigrant and refugee patients:	◯ None<br>◯ 0 -5%<br>⊗ 5-10%<br>⊗ 10-25%<br>◯ > 25%
b. During my outpatient rotations, I take care of the following percentage of immigrant and refugee patients:	◯ None<br>◯ 0-10%<br>◯ 10 -25%<br>⊗ 25-50%<br>◯ 50-75%<br>◯ >75%
c. I would like to take care of more immigrant and refugee patients.	◯ Strongly disagree<br>◯ Disagree<br>◯ No opinion<br>⊗ Agree<br>◯ Strongly agree
d. I plan to take care of immigrants and refugees when I finish residency.	◯ Strongly disagree<br>◯ Disagree<br>◯ No opinion<br>⊗ Agree<br>◯ Strongly agree
e. I plan to do short term (< 6 months) international work when I finish residency.	◯ Strongly disagree<br>◯ Disagree<br>◯ No opinion<br>◯ Agree<br>⊗ Strongly agree
f. I plan to do long term (>6 months) international work when I finish residency.	◯ Strongly disagree<br>◯ Disagree<br>◯ No opinion<br>⊗ Agree<br>◯ Strongly agree
g. I plan to work in health disparities in the following way after residency:	Primary Care for underserved populations. Considering working for IHS.

SECTION B: MEDICAL EDUCATION

A. Please indicate your level of agreement with the following statements regarding your medical education and knowledge about immigrants and refugees by checking the box that best represents your opinion.

a. I have received specialized training in immigrant and refugee health, tropical medicine, or cross-cultural health.

- ◯ Strongly disagree
- ◯ Disagree
- ◯ No opinion
- ⊗ Agree
- ◯ Strongly agree

b. If you have received specialized training in immigrant and refugee health, tropical medicine, or cross-cultural health, please indicate all the contexts in which you received this training:

- ☒ As an undergraduate.
- ☒ As a medical student.
- ☒ As part of my residency.
- ☒ A special program.
- ☐ As part of my fellowship.
- ☐ As part of a degree program (e.g. MPH)
- ☐ Other:

c. I feel comfortable with my fund of knowledge regarding immigrant and refugee health.

- ◯ Strongly disagree
- ⊗ Disagree
- ◯ No opinion
- ◯ Agree
- ◯ Strongly agree

d. I would like to have further training in immigrant and refugee health.

- ◯ Strongly disagree
- ◯ Disagree
- ◯ No opinion
- ⊗ Agree
- ◯ Strongly agree

e. If you agree with the above, please indicate all the contexts in which you would like to receive this training:

- ☒ As part of my residency.
- ☒ A special program.
- ☐ As part of my fellowship.

SECTION C: Attitudes towards immigrant health

A. Please indicate your level of agreement with the following statements regarding immigrant and refugee health by checking the box that best represents your opinion.

a. I enjoy taking care of immigrants and refugees.

- ◯ Never
- ◯ Rarely
- ◯ Sometimes
- ⊗ Usually
- ◯ Always

b. Please indicate the reasons that you enjoy taking care of immigrants and refugees (may choose more than one).

- ☒ Tropical and other conditions not frequently diagnosed in US-born patients
- ☒ Learning about other cultures
- ☐ They don't complain as much
- ☐ Being able to hear their stories
- ☐ Their care is more complicated
- ☐ Their care is less complicated
- ☒ They are very appreciative of your help.
- ☐ They are extremely vulnerable
- ☐ Other:

c. Taking care of immigrants and refugees is more challenging than taking care of US born patients.

- ◯ Never
- ◯ Rarely
- ◯ Sometimes
- ⊗ Usually
- ◯ Always

d. Please mark all the challenges that you face as a provider when providing care to immigrants and refugees (may choose more than one):

- ☒ Language barriers
- ☒ Insurance barriers
- ☒ Cultural barriers
- ☒ Finding a professional interpreter
- ☒ Knowing how to work with a professional interpreter
- ☒ Time constraints
- ☒ My own knowledge related to tropical and travel medicine
- ☒ Transportation problems for the patient
- ☒ Patients not understanding treatment plan
- ☒ Patients not following treatment plan
- ☒ My lack of knowledge regarding the patient's culture
- ☒ Bias or stereotyping
- ☐ Other:

e. Please mark all of the challenges faced by immigrant and refugee populations when receiving healthcare that you have perceived or witnessed (may choose more than one):

- ☒ Language barriers
- ☒ Insurance barriers
- ☒ Cultural barriers
- ☒ Finding a professional interpreter
- ☒ Knowing how to work with a professional interpreter
- ☒ Time constraints
- ☒ Insufficiently trained health care providers
- ☒ Transportation problems for the patient
- ☒ Food insufficiency
- ☒ Need for child care
- ☒ Patients not understanding treatment plan
- ☒ Patients not following treatment plan
- ☒ My lack of knowledge regarding the patient's culture
- ☒ Bias or stereotyping
- ☒ Trust issues
- ☐ Other...

f. Rank how well immigrants and refugees understand the healthcare that you are trying to provide.

- ◯ Significantly less than a US born individual
- ⊗ Less than a US born individual
- ◯ Equivalent to a US born individual
- ◯ More than a US born patient
- ◯ Significantly more than a US born individual

g. Immigrants and refugees adhere to treatment plans and follow my recommendations.

- ◯ Never
- ◯ Rarely
- ⊗ Sometimes
- ◯ Usually
- ◯ Always

h. Immigrants and refugees should receive the same care and insurance coverage as US born patients.

- ◯ Never
- ◯ Rarely
- ◯ Sometimes
- ⊗ Usually
- ◯ Always

i. Immigrants and refugees who are undocumented should receive the same care and insurance coverage as US born patients.

- ◯ Never
- ◯ Rarely
- ◯ Sometimes
- ⊗ Usually
- ◯ Always

j. Every physician is professionally obligated to care for immigrants and refugees if they present to your clinic or hospital.

- ◯ Strongly disagree
- ◯ Disagree
- ◯ No opinion
- ◯ Agree
- ⊗ Strongly agree

k. Is healthcare a human right?

⊗ Yes
◯ No

B. If you wish, please tell us about what you enjoy or do not enjoy about immigrant and refugee health care and the greatest challenges you face in caring for this population.

____

SECTION D: DEMOGRAPHIC INFORMATION
Please answer the following questions by checking the box in front of the response choice that best describes you.

a. Your age?

◯ 20 to 24
⊗ 25 to 29
◯ 30 to 34
◯ 35 to 39
◯ 40 or older

b. Your gender?

⊗ Female
◯ Male
◯ Other

c. Are you Hispanic or Latino?

◯ Yes
⊗ No

d. What is your race? (Select one or more responses)

◯ American Indian or Alaska Native
◯ Asian (Please specify):
◯ Black or African American
◯ Native Hawaiian or Other Pacific Islander
⊗ White
◯ Other (Please specify):

e. Were you born in the United States?

⊗ Yes
◯ No

g. Your residency year?

⊗ PGY1
◯ PGY2
◯ PGY3
◯ PGY4
◯ PGY5

h. How would you classify your political ideology?

◯ Conservative
◯ Somewhat conservative
◯ Moderate
◯ Somewhat liberal
⊗ Liberal
◯ Other (Please specify):

i. Estimated level of educational debt?

◯ None
◯ Less than $50,000
◯ $50,000 - $100,000
◯ $100,000 - $200,000
⊗ $200,000 or more

j. Do you plan to subspecialize?

◯ Yes
⊗ No

k. Languages spoken?

- ☒ English
- ☒ Spanish
- ☐ French
- ☐ Hmong
- ☐ Somali
- ☐ Japanese
- ☐ Chinese
- ☐ Russian
- ☐ Ethiopian
- ☐ Other ___________

l. Are you in the Global Health Pathway?

- ⊗ Yes
- ◯ No

m. Did you earn your degree in the US?

- ⊗ Yes
- ◯ No

n. What residency program are you in?

- ◯ Internal Medicine
- ⊗ Med-Peds
- ◯ Pediatrics
- ◯ Family Practice
- ◯ Neurology
- ◯ Psychiatry
- ◯ ObGyn
- ◯ Neurosurgery
- ◯ General Surgery
- ◯ Orthopedic Surgery
- ◯ Urology
- ◯ Surgical sub-specialty (please specify in text box below)
- ◯ Non-clinical specialty (radiology, pathology; please specify in text box below)

Medical Trainees' attitudes, knowledge, and experience with immigrant and refugee health

Response was added on 06/23/2014 5:50pm.

SECTION A: Personal experience with immigrant and refugee health care.

A. Please indicate your level of agreement with the following statements regarding your personal experience with immigrant and refugee health care by checking the box that best represents your experience.

a. During my inpatient rotations, I take care of the following percentage of immigrant and refugee patients:
- ◯ None
- ◯ 0 -5%
- ⊗ 5-10%
- ◯ 10-25%
- ◯ > 25%

b. During my outpatient rotations, I take care of the following percentage of immigrant and refugee patients:
- ◯ None
- ◯ 0-10%
- ◯ 10 -25%
- ⊗ 25-50%
- ◯ 50-75%
- ◯ >75%

c. I would like to take care of more immigrant and refugee patients.
- ◯ Strongly disagree
- ◯ Disagree
- ⊗ No opinion
- ◯ Agree
- ◯ Strongly agree

d. I plan to take care of immigrants and refugees when I finish residency.
- ◯ Strongly disagree
- ◯ Disagree
- ◯ No opinion
- ⊗ Agree
- ◯ Strongly agree

e. I plan to do short term (< 6 months) international work when I finish residency.
- ◯ Strongly disagree
- ◯ Disagree
- ◯ No opinion
- ⊗ Agree
- ◯ Strongly agree

f. I plan to do long term (>6 months) international work when I finish residency.
- ◯ Strongly disagree
- ⊗ Disagree
- ◯ No opinion
- ◯ Agree
- ◯ Strongly agree

g. I plan to work in health disparities in the following way after residency:

Yes, global health and refugee/immigrant populations in US

SECTION B: MEDICAL EDUCATION

A. Please indicate your level of agreement with the following statements regarding your medical education and knowledge about immigrants and refugees by checking the box that best represents your opinion.

a. I have received specialized training in immigrant and refugee health, tropical medicine, or cross-cultural health.

- ◯ Strongly disagree
- ◯ Disagree
- ◯ No opinion
- ⊗ Agree
- ◯ Strongly agree

b. If you have received specialized training in immigrant and refugee health, tropical medicine, or cross-cultural health, please indicate all the contexts in which you received this training:

- ☐ As an undergraduate.
- ☐ As a medical student.
- ☐ As part of my residency.
- ☐ A special program.
- ☐ As part of my fellowship.
- ☐ As part of a degree program (e.g. MPH)
- ☐ Other:

c. I feel comfortable with my fund of knowledge regarding immigrant and refugee health.

- ◯ Strongly disagree
- ◯ Disagree
- ◯ No opinion
- ⊗ Agree
- ◯ Strongly agree

d. I would like to have further training in immigrant and refugee health.

- ◯ Strongly disagree
- ◯ Disagree
- ◯ No opinion
- ⊗ Agree
- ◯ Strongly agree

e. If you agree with the above, please indicate all the contexts in which you would like to receive this training:
□□

- ☐ As part of my residency.
- ☐ A special program.
- ☐ As part of my fellowship.

SECTION C: Attitudes towards immigrant health

A.□Please indicate your level of agreement with the following statements regarding immigrant and refugee health by checking the box that best represents your opinion.

a. I enjoy taking care of immigrants and refugees.

- ◯ Never
- ◯ Rarely
- ◯ Sometimes
- ⊗ Usually
- ◯ Always

b. Please indicate the reasons that you enjoy taking care of immigrants and refugees (may choose more than one).

- ☒ Tropical and other conditions not frequently diagnosed in US-born patients
- ☒ Learning about other cultures
- ☐ They don't complain as much
- ☐ Being able to hear their stories
- ☒ Their care is more complicated
- ☐ Their care is less complicated
- ☒ They are very appreciative of your help.
- ☒ They are extremely vulnerable
- ☐ Other:

c. Taking care of immigrants and refugees is more challenging than taking care of US born patients.

- ◯ Never
- ◯ Rarely
- ⊗ Sometimes
- ◯ Usually
- ◯ Always

d. Please mark all the challenges that you face as a provider when providing care to immigrants and refugees (may choose more than one):

- ☐ Language barriers
- ☒ Insurance barriers
- ☐ Cultural barriers
- ☐ Finding a professional interpreter
- ☐ Knowing how to work with a professional interpreter
- ☐ Time constraints
- ☐ My own knowledge related to tropical and travel medicine
- ☐ Transportation problems for the patient
- ☐ Patients not understanding treatment plan
- ☒ Patients not following treatment plan
- ☐ My lack of knowledge regarding the patient's culture
- ☐ Bias or stereotyping
- ☐ Other:

e. Please mark all of the challenges faced by immigrant and refugee populations when receiving healthcare that you have perceived or witnessed (may choose more than one):

- ☐ Language barriers
- ☒ Insurance barriers
- ☒ Cultural barriers
- ☐ Finding a professional interpreter
- ☐ Knowing how to work with a professional interpreter
- ☐ Time constraints
- ☐ Insufficiently trained health care providers
- ☐ Transportation problems for the patient
- ☐ Food insufficiency
- ☐ Need for child care
- ☐ Patients not understanding treatment plan
- ☒ Patients not following treatment plan
- ☐ My lack of knowledge regarding the patient's culture
- ☐ Bias or stereotyping
- ☒ Trust issues
- ☐ Other...

f. Rank how well immigrants and refugees understand the healthcare that you are trying to provide.

- ◯ Significantly less than a US born individual
- ⊗ Less than a US born individual
- ◯ Equivalent to a US born individual
- ◯ More than a US born patient
- ◯ Significantly more than a US born individual

g. Immigrants and refugees adhere to treatment plans and follow my recommendations.

- ◯ Never
- ◯ Rarely
- ⊗ Sometimes
- ◯ Usually
- ◯ Always

h. Immigrants and refugees should receive the same care and insurance coverage as US born patients.

- ◯ Never
- ◯ Rarely
- ⊗ Sometimes
- ◯ Usually
- ◯ Always

i. Immigrants and refugees who are undocumented should receive the same care and insurance coverage as US born patients.

- ◯ Never
- ◯ Rarely
- ⊗ Sometimes
- ◯ Usually
- ◯ Always

j. Every physician is professionally obligated to care for immigrants and refugees if they present to your clinic or hospital.

- ◯ Strongly disagree
- ◯ Disagree
- ◯ No opinion
- ⊗ Agree
- ◯ Strongly agree

k. Is healthcare a human right?

⊗ Yes
◯ No

B. If you wish, please tell us about what you enjoy or do not enjoy about immigrant and refugee health care and the greatest challenges you face in caring for this population.

____

SECTION D: DEMOGRAPHIC INFORMATION
Please answer the following questions by checking the box in front of the response choice that best describes you.

a. Your age?

◯ 20 to 24
⊗ 25 to 29
◯ 30 to 34
◯ 35 to 39
◯ 40 or older

b. Your gender?

◯ Female
⊗ Male
◯ Other

c. Are you Hispanic or Latino?

◯ Yes
⊗ No

d. What is your race? (Select one or more responses)

◯ American Indian or Alaska Native
◯ Asian (Please specify):
◯ Black or African American
◯ Native Hawaiian or Other Pacific Islander
⊗ White
◯ Other (Please specify):

e. Were you born in the United States?

⊗ Yes
◯ No

g. Your residency year?

◯ PGY1
⊗ PGY2
◯ PGY3
◯ PGY4
◯ PGY5

h. How would you classify your political ideology?

◯ Conservative
◯ Somewhat conservative
◯ Moderate
◯ Somewhat liberal
⊗ Liberal
◯ Other (Please specify):

i. Estimated level of educational debt?

◯ None
◯ Less than $50,000
◯ $50,000 - $100,000
⊗ $100,000 - $200,000
◯ $200,000 or more

j. Do you plan to subspecialize?

⊗ Yes
◯ No

k. Languages spoken?

☒ English
☒ Spanish
☐ French
☐ Hmong
☐ Somali
☐ Japanese
☐ Chinese
☐ Russian
☐ Ethiopian
☒ Other ___________

AMerican Sign Language, Hindi

l. Are you in the Global Health Pathway?

⊗ Yes
◯ No

m. Did you earn your degree in the US?

⊗ Yes
◯ No

n. What residency program are you in?

⊗ Internal Medicine
◯ Med-Peds
◯ Pediatrics
◯ Family Practice
◯ Neurology
◯ Psychiatry
◯ ObGyn
◯ Neurosurgery
◯ General Surgery
◯ Orthopedic Surgery
◯ Urology
◯ Surgical sub-specialty (please specify in text box below)
◯ Non-clinical specialty (radiology, pathology; please specify in text box below)
